# α,γ-Dioxygenated amides via tandem Brook rearrangement/radical oxygenation reactions and their application to syntheses of γ-lactams

**DOI:** 10.3762/bjoc.17.58

**Published:** 2021-03-09

**Authors:** Mikhail K Klychnikov, Radek Pohl, Ivana Císařová, Ullrich Jahn

**Affiliations:** 1 Institute of Organic Chemistry and Biochemistry of the Czech Academy of Sciences, Flemingovo náměstí 2, 16610 Prague 6, Czech Republic; 2 Department of Inorganic Chemistry, Faculty of Science, Charles University in Prague, Hlavova 2030/8, 12843 Prague 2, Czech Republic

**Keywords:** Brook rearrangement, cyclization, electron transfer, γ-lactams, tandem reactions

## Abstract

Pyrrolidones are common heterocyclic fragments in various biologically active compounds. Here, a two-step radical-based approach to γ-lactams bearing three to four stereocenters starting from epoxides, *N*-allylic silylacetamides and TEMPO is reported. The sequence starts with a new tandem nucleophilic substitution/Brook rearrangement/single electron transfer-induced radical oxygenation furnishing orthogonally protected α,γ-dioxygenated *N*-allylamides with wide scope, mostly good yields, and partly good diastereo- and enantioselectivity for defined combinations of chiral epoxides and chiral amides. This represents a very rare example of an oxidative geminal C–C/C–O difunctionalization next to carbonyl groups. The resulting dioxygenated allylic amides are subsequently subjected to persistent radical effect-based 5-*exo*-*trig* radical cyclization reactions providing functionalized pyrrolidones in high yields as diastereomeric mixtures. They converge to 3,4-*trans-*γ-lactams by base-mediated equilibration, which can be easily further diversified. Stereochemical models for both reaction types were developed.

## Introduction

**Figure 1:**
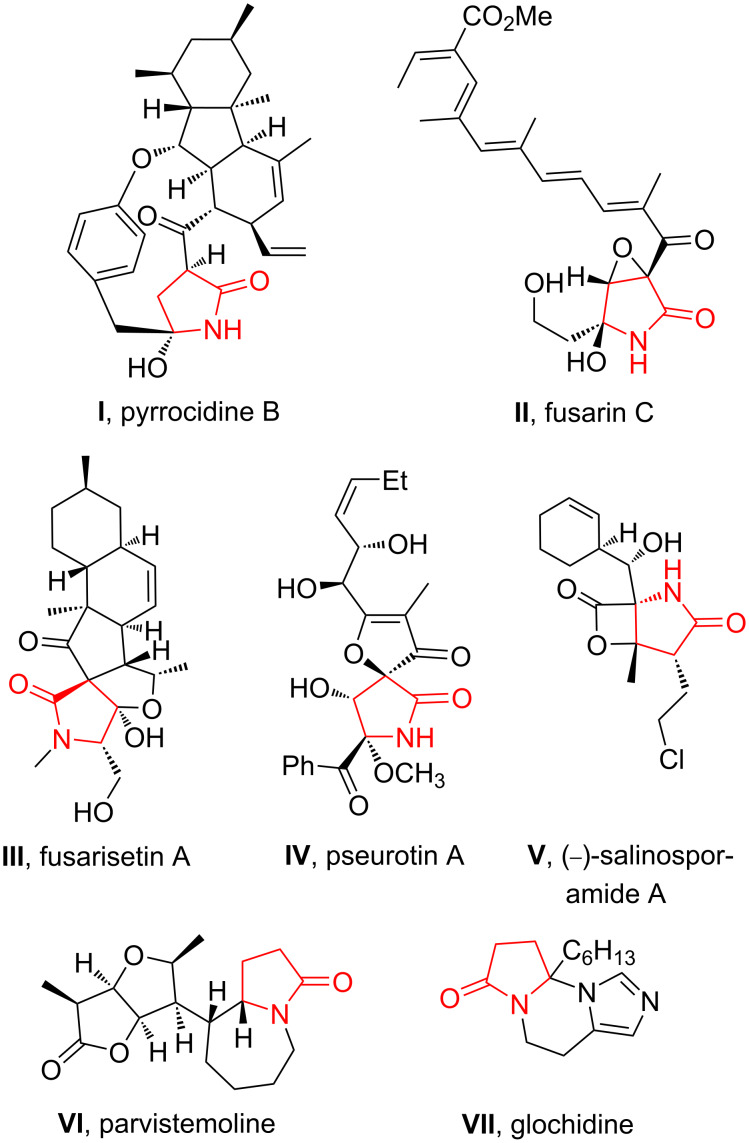
Selected alkaloids containing the pyrrolidone motif.

Nitrogen-containing heterocycles are widely distributed in biologically active compounds [1–4]. Saturated nitrogen heterocycles such as pyrrolidines [5–9], piperidines, pyrrolizidines or indolizidines [10–16] are central fragments in various natural products, which are often synthesized from lactams by reduction. This makes them important building blocks in the total syntheses of alkaloids and their non-natural analogs [17–21]. However, the γ-lactam substructure itself is a central fragment of numerous bioactive alkaloids, such as pyrrocidine B (**I**) [22], fusarin C (**II**) [23], fusarisetin A (**III**) [24], pseurotin A (**IV**) [25], (−)-salinosporamide A (**V**) [26], parvistemoline (**VI**) [27], glochidine (**VII**) [28], and other alkaloids [29–34] (Figure 1). Moreover, functionalized synthetic γ-lactams are important lead compounds, e.g., derivatives with antibacterial activity were discovered, what gains importance with respect to the increasing bacterial resistance toward traditional β-lactam antibiotics [35–39].

**Scheme 1:**
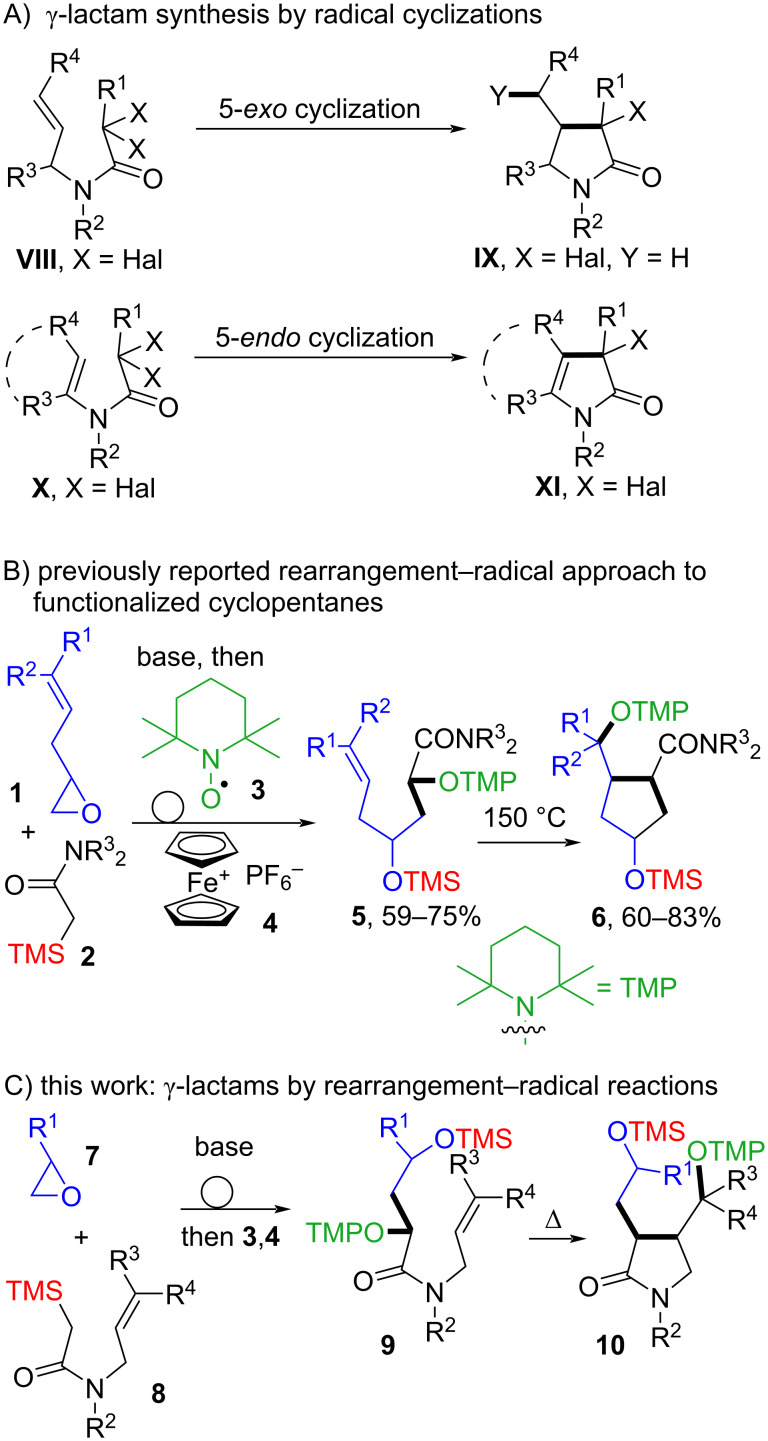
A) Classical γ-lactam synthesis by atom transfer radical cyclizations; B) previously developed tandem epoxide opening/Brook rearrangement/radical oxygenation and radical carbocyclizations; C) proposed approach to functionalized γ-lactams **10** by Brook rearrangement/radical oxygenation and subsequent 5-*exo* cyclization reactions.

Various synthetic pathways can be applied for the construction of the γ-lactam scaffold [40–44]. The pyrrolidinone fragment is often synthesized by transition metal- [45–50] or Lewis acid-catalyzed cyclization reactions [51–54]. The Diels–Alder reaction can also be used for the preparation of functionalized γ-lactams in a single step [55]. Radical 5-*exo* or 5-*endo* cyclizations of substituted *N*-allyl or *N*-vinyl α-halo amides **VIII** [56–61] or **X** [62–66] using atom transfer and other chain reactions, as well as non-chain methods [67–73] have been used to approach diverse γ-lactam-containing skeletons of the general structure **IX** or **XI**, respectively (Scheme 1A).

Recently, we became interested in merging sigmatropic rearrangements with radical oxygenation reactions since profound changes in the connectivity patterns during both reaction modes will potentially significantly simplify the access to complex target molecules [74–75]. The principle is illustrated for a merger of nucleophilic opening of allylepoxides **1** with silylacetamides **2**/Brook rearrangement [76–78] and oxygenation with TEMPO (**3**) leading to γ-(silyloxy)-α-(aminoxy)amides **5**, which can be subsequently subjected to thermal radical cyclization reactions according to the persistent radical effect [79–80] forming functionalized cyclopentanes **6** (Scheme 1B).

Based on this sequence various other reaction pathways can be envisaged. Among them we hypothesized that the nucleophilic ring opening of simple epoxides **7** by *N*-allylic 2-silylacetamides would provide an intermediate alkoxide from which the Brook rearrangement and subsequent oxygenation could proceed (Scheme 1C). This represents in the event a geminal C–C/C–O difunctionalization of amide **8** and results in the α,γ-dioxygenated *N*-allylic amides **9**. Thermal radical cyclizations to lactams of type **10** based on the persistent radical effect (PRE) are unknown and may provide a simple access to 3,4-disubstituted γ-lactams.

We report here that tandem nucleophilic epoxide ring-opening/Brook rearrangement/radical oxygenation reactions are indeed very effective for the synthesis of diverse *N*-allylic α-(aminoxy)amides **9** from various epoxides **7** and a range of *N*-allylic α-silylamides **8**. α-(Aminoxy)amides **9** serve well for the synthesis of polysubstituted γ-lactams **10** with moderate diastereoselectivities. The stereochemistry of the initial cyclization products can be however simplified by further useful reaction steps.

## Results

### Tandem nucleophilic substitution/Brook rearrangement/radical α-oxygenation reactions

The *N*-allylic α-(trimethylsilyl)acetamides **8a**–**m** were efficiently prepared by a two-step sequence. First, *N*-allyl acetamides **11a–m** were synthesized by N-acetylation of the corresponding acyclic or cyclic allylic amines in very good yields (see Supporting Information File 1 for details). Their subsequent α-deprotonation by LDA followed by treatment with chlorotrimethylsilane at −78 °C [81] resulted in clean C-silylation of the corresponding enolate providing silylacetamides **8a–m** in good to very good yields (Table 1, entries 1–13).

**Table 1:** Preparation of tertiary *N*-allylic α-(trimethylsilyl)amides **8**^a^.

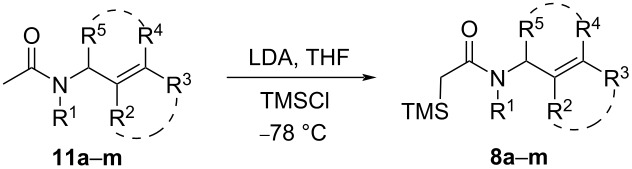							

entry	**11**	R^1^	R^2^	R^3^	R^4^	R^5^	**8**, %

1	**a**	allyl	H	H	H	H	**a**, 76
2	**b**	CH_3_	H	H	H	H	**b**, 70
3	**c**	Bn	CH_3_	H	H	H	**c**, 75
4	**d**	Bn	H	CH_3_	CH_3_	H	**d**, 72
5	**e**	(*S*)-PhCHCH_3_	H	H	H	H	**e**, 89
6	**f**	(*S*)-β-NapCHCH_3_^b^	H	H	H	H	**f**, 95
7	**g**	(*S*)-PhCHCH_3_	H	CH_3_	CH_3_	H	**g**, 88
8	**h**	(*S*)-PhCHCH_3_	CH_3_	H	H	H	**h**, 93
9	**i**	(*S*)-PhCHCH_3_	-(CH_2_)_3_-		H	H	**i**, 84
10	**j**	Bn	-(CH_2_)_3_-		H	H	**j**, 82
11	**k**	Bn	-(CH_2_)_4_-		H	H	**k**, 85
12	**l**	Bn	H	H	-(CH_2_)_2_-		**l**, 91
13	**m**	Bn	H	H	(*R*)-(CH_2_)_3_-		**m**, 76

^a^General conditions: **11** (1 equiv), iPr_2_NH (1.1 equiv), *n-*BuLi (1.1 equiv), TMSCl (1.05 equiv), −78 °C, 1 h; ^b^Nap = naphthyl.

For the synthesis of the targeted orthogonally protected α-(aminoxy)-γ-(silyloxy)amides **9** α-silylacetamides **8a**–**k** were deprotonated by *s-*BuLi and treated with commercially available racemic epoxides **7a**–**d**,**f** (Table 2, entries 1–7 and 13–15) or with enantiomerically pure epoxides (*S*)-**7b**, (*R*)-**7b**, or (*S*)-**7e** (Table 2, entries 8–12) at 0 °C. The epoxide opening/Brook rearrangement steps were typically complete after an hour, except for cyclohexene oxide **7f** for which the nucleophilic opening and Brook rearrangement steps took 24 h (Table 2, entry 13). Ferrocenium hexafluorophosphate (**4**) and TEMPO (**3**) were subsequently added to trigger the single electron oxidation of the formed amide enolates and radical oxygenation affording α-(aminoxy)amides **9a**–**n** in good 51–77% isolated yields. Cyclic units in the allylic N-substituent (Table 2, entries 14 and 15) and the epoxide (Table 2, entry 13) are tolerated. Most dioxygenated amides **9b**–**h**,**m**,**n** were isolated as inseparable 1–1.2:1 *anti*/*syn* mixtures of unassigned diastereomers (Table 2, entries 2–8, 14 and 15), thus the silyloxy group in γ-position did not influence the face selectivity of radical coupling with TEMPO (**3**). However, since for all dioxygenated amides **9** the stereocenter at the alkoxyamine unit will be destroyed in the subsequent radical reaction, the low diastereoselectivity at that center is not of concern. In contrast, in the reactions of silylamides **8e**–**g** bearing enantiomerically pure 1-arylethyl substituents with enantiomerically pure (*S*)-propylene oxide ((*S*)-**7b**) the dioxygenated amides (2*R,*4*S*)*-***9i–k** were predominately formed with 3:1 *anti*/*syn* diastereoselectivity in the radical coupling with TEMPO irrespective of the aryl substituent (Table 2, entries 9, 11, and 12), whereas the sequence of amide **8e** with (*R*)-propylene oxide ((*R*)-**7b**) provided the product (2*S,*4*R*)-**9i** in a much better 8:1 *anti*/*syn* coupling diastereoselectivity (Table 2, entry 10).

**Table 2:** Tandem nucleophilic ring opening/Brook rearrangement/radical oxygenation^a^.

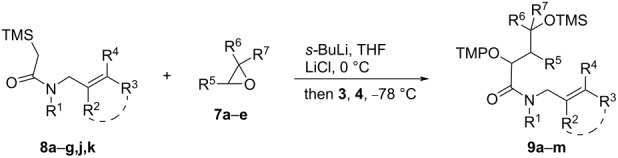											

entry	**8**	**7**^b^	R^1^	R^2^	R^3^	R^4^	R^5^	R^6^	R^7^	**9**, %	*anti*/*syn*

1	**a**	**a**	allyl	H	H	H	H	CH_3_	CH_3_	**a**, 61	–
2	**a**	**b**	allyl	H	H	H	H	CH_3_	H	**b**, 77	1.1:1
3	**a**	**c**	allyl	H	H	H	H	C_4_H_9_	H	**c**, 71	1:1
4	**a**	**d**	allyl	H	H	H	H	Ph	H	**d**, 56	1.1:1
5	**b**	**b**	CH_3_	H	H	H	H	CH_3_	H	**e**, 68	1.1:1
6	**c**	**b**	Bn	CH_3_	H	H	H	CH_3_	H	**f**, 51	1:1
7	**d**	**b**	Bn	H	CH_3_	CH_3_	H	CH_3_	H	**g**, 53	1:1
8	**a**	(*S*)-**e**	allyl	H	H	H	H	A^c^	H	**h**, 65	1.1:1
9	**e**	(*S*)-**b**	B^c^	H	H	H	H	CH_3_	H	**i**, 64	3:1
10	**e**	(*R*)-**b**	B^c^	H	H	H	H	CH_3_	H	**i**, 63	8:1
11	**f**	(*S*)-**b**	C^c^	H	H	H	H	CH_3_	H	**j**, 62	3:1
12	**g**	(*S*)-**b**	B^c^	H	CH_3_	CH_3_	H	CH_3_	H	**k**, 61	3:1
13^d^	**a**	**f**	allyl	H	H	H	-(CH_2_)_4_-		H	**l**, 63	7:1
14	**j**	**b**	Bn	-(CH_2_)_3_-		H	H	CH_3_	H	**m**, 61	1.2:1
15	**k**	**b**	Bn	-(CH_2_)_4_-		H	H	CH_3_	H	**n**, 62	1.1:1

^a^General conditions: *s-*BuLi (1.1 equiv), **8** (1 equiv), LiCl (6 equiv), 0 °C, 30 min, **7** (1.05 equiv), 0 °C, then **3** (1.05 equiv), **4** (1.2 equiv), −78 °C; ^b^epoxides **7** are racemic unless indicated otherwise; ^c^A = (*S*)-CH_2_OBn, B = (*S*)-PhCHCH_3,_ C = (*S*)-β-NapCHCH_3_; ^d^nucleophilic opening of **7e** complete after 24 h at room temperature.

**Figure 2:**
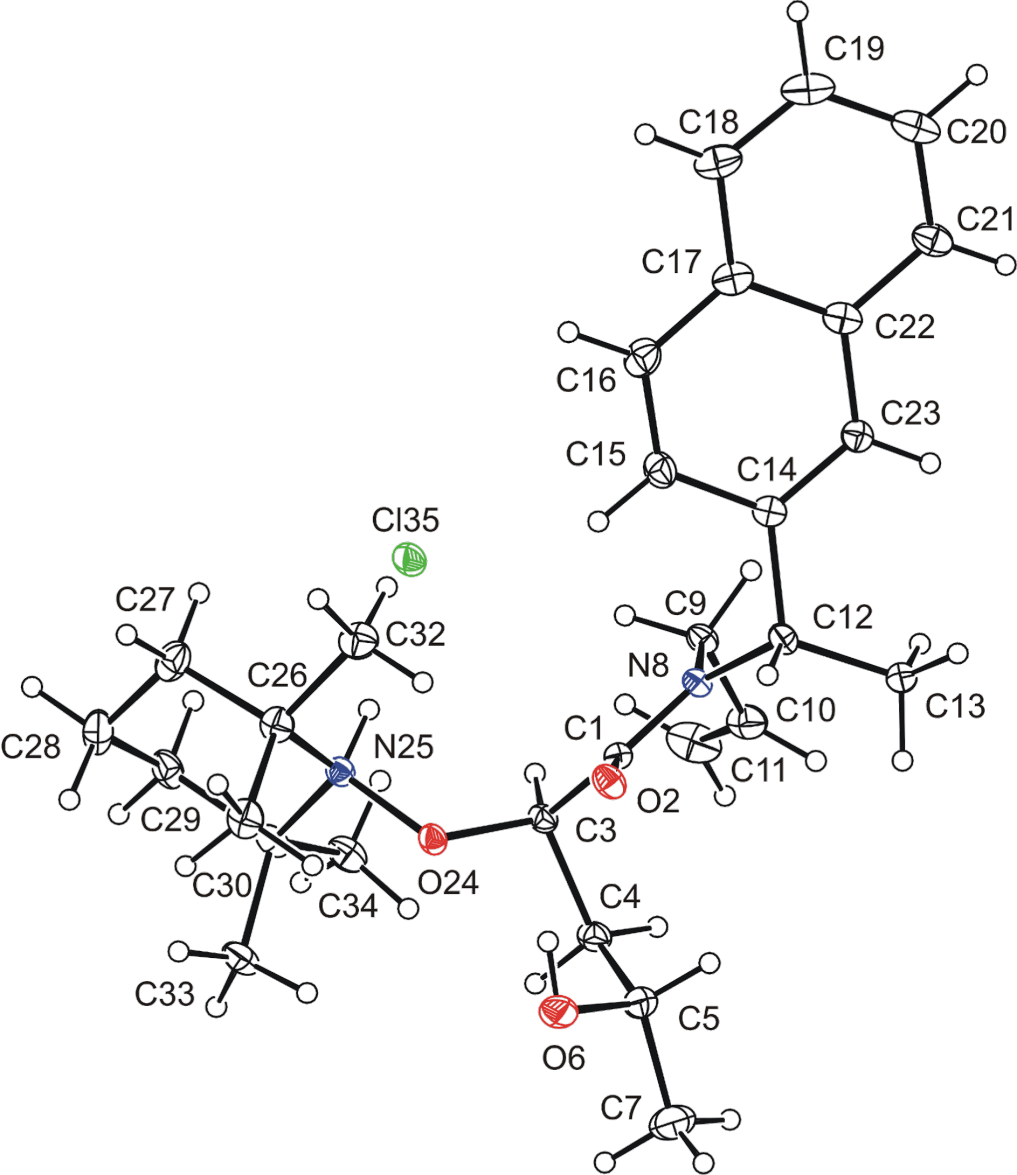
X-ray crystal structure of the major (2*R,*4*S*)-alkoxyamine hydrochloride derived from **9j**. Displacement ellipsoids at 30% probability level and a disordered CHCl_3_ solvent molecule is not shown for clarity.

The configuration of the major *anti-*diastereomer of alkoxyamine **9j** was determined by X-ray crystallography after desilylation and hydrochloride formation (see Supporting Information File 1 for details). The (*R*)-configuration at C2 as well as the (*S*)-configuration at both, C4 and the *N*-arylethyl group were established (Figure 2).

A good diastereoselectivity was also observed for the ring-opening/Brook rearrangement/oxygenation sequence with cyclohexene oxide **7f**, which furnished the dioxygenated amide **9l** with a 7:1 *anti*/*syn* diastereoselectivity for the radical coupling (Table 2, entry 13). When the reaction was quenched after completion of the Brook rearrangement, *N*-allyl-*N*-propyl-2-(2-((trimethylsilyl)oxy)cyclohexyl)acetamide was obtained as a single diastereomer because of the stereospecific epoxide ring-opening in 80% yield (not shown, see Supporting Information File 1 for details).

**Scheme 2:**
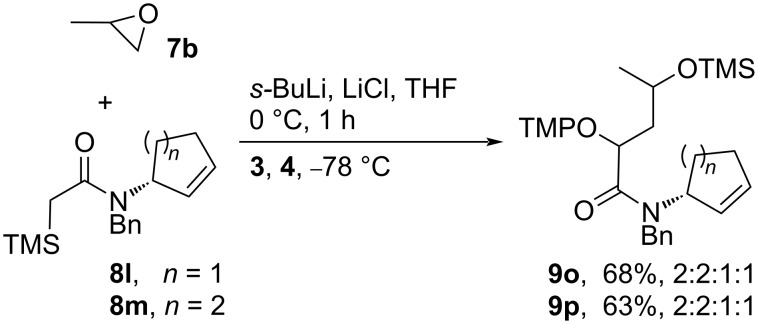
Formation of the α-(aminoxy)amides **9o**,**p**.

*N*-Cyclopent-2-enyl and *N*-cyclohex-2-enylamides **8l**,**m** provided the oxygenated products **9o**,**p** in 68% and 63% yields, respectively, as 2:2:1:1 mixture of diastereomers (Scheme 2). Thus, the chiral cyclic amide substituent on the nitrogen atom influences the selectivity of the radical coupling with TEMPO to some extent, though less than the 1-arylethyl groups.

Somewhat surprisingly, amides **8h**,**i** did not react with propylene oxide **7b** neither at 0 °C nor at room temperature. In order to confirm enolate formation from **8h**,**i** with *s-*BuLi, a deuterium quenching experiment with D_2_O was performed. Analysis by ^1^H NMR spectroscopy revealed 87 and 91% deuterium incorporation, respectively, indicating that a deprotonation occurred, but the epoxide opening was hampered by the combination of a sterically more demanding branched amide substituent and the R^2^ substituent at the internal carbon of the allylic unit.

### Transformation of α-(aminoxy)amides **9** to lactams **12** by persistent radical effect-based cyclization reactions

The acyclic α-(aminoxy)amides **9a**–**k** are suitable precursors for thermal radical cyclization reactions based on the persistent radical effect. Heating them to 150 °C in *tert*-butanol provided diverse 1,3,4-trisubstituted pyrrolidones (Table 3). For stability reasons the initially obtained silyl-protected lactams **10a**–**k** were deprotected without isolation by TBAF in THF affording hydroxy lactams **12a**–**k** in 66–93% yields. The thermal cyclization of α-(aminoxy)amide **9a** provided two diastereomers of lactam **12a** in a 2:1 *trans*/*cis* ratio (Table 3, entry 1). The *N*-allylic amides **9b–f** provided lactams **12b**–**f** as mixtures of four inseparable diastereomers in which those with *trans* orientation of the substituents at C3 and C4 of the formed 2-pyrrolidone ring predominated with moderate selectivity (Table 3, entries 2–7). The diastereomeric ratio is more or less independent of the nature of the amide substituent R^1^ or the substituent R^4^. Amide **9f** with a methyl group at the internal position of the alkene unit cyclized exclusively in the 5-*exo*-*trig* mode and provided the pyrrolidone **12f** with a quaternary center at C4 in moderate yield and similar diastereoselectivity as for **12a**–**d** (Table 3, entry 7); a product of potentially competing 6-*endo* cyclization was not detected. Amides **9g**,**k** with trisubstituted alkene units furnished 4-isopropenylpyrrolidones **12g**,**k** in very good yields as mixtures of inseparable diastereomers (Table 3, entries 7 and 11); no alkoxyamine-containing products were isolated. Lactam **12h** with a defined hydroxy group configuration in the side chain at C3 of the lactam as well as pyrrolidones **12i**–**k** bearing configurationally defined 1-arylethyl groups at the amide nitrogen and the hydroxy group in the C3 side chain were obtained as inseparable mixtures of four diastereomers (Table 3, entries 8–11). The two possible *trans*-diastereomers predominated with moderate selectivity. These results indicate negligible asymmetric inductions from the chiral centers, both at the exocyclic hydroxy substituent in the C3 side chain (Table 3, entry 8) and/or of the 1-arylethyl group during the radical cyclization under the reaction conditions (Table 3, entries 9–11). The size of the arylethyl substituent at the nitrogen atom also plays essentially no role for the diastereoselectivity of the cyclization (Table 3, entry 10 vs entry 9).

**Table 3:** Hydroxyalkyl-γ-lactams **12** by PRE-based radical 5-*exo* cyclizations^a^.

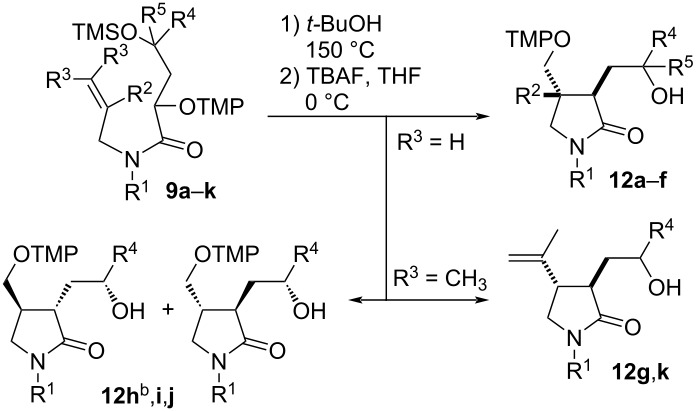								

entry	**9**	R^1^	R^2^	R^3^	R^4^	R^5^	**12**, %	dr

1	**a**	allyl	H	H	CH_3_	CH_3_	**a**, 91	2:1
2	**b**	allyl	H	H	CH_3_	H	**b**, 87	2.5:2.5:1:1
3	**c**	allyl	H	H	C_4_H_9_	H	**c**, 86	2:2:1:1
4	**d**	allyl	H	H	Ph	H	**d**, 86	3:3:1:1
5	**e**	CH_3_	H	H	CH_3_	H	**e**, 82	2:2:1:1
6	**f**	Bn	CH_3_	H	CH_3_	H	**f**, 66	3:3:1:1
7	**g**	Bn	H	CH_3_	CH_3_	H	**g**, 93	2:2:1:1
8	**h**	allyl	H	H	(*S*)-CH_2_OBn	H	**h**, 72	4:4:1:1^b^
9	**i**	(*S*)-PhCHCH_3_	H	H	(*S*)-CH_3_	H	**i**, 82	2:2:1:1
10	**j**	(*S*)-β-NapCHCH_3_	H	H	(*S*)-CH_3_	H	**j**, 77	2:2:1:1
11	**k**	*(S*)-PhCHCH_3_	H	CH_3_	(*S*)-CH_3_	H	**k**, 92	2:2:1:1

^a^General conditions: 1) **9** (1 equiv), *t-*BuOH, 150 °C, 1 h; 2) TBAF (1.2 equiv), THF, 0 °C, 30 min; ^b^the enantiomeric product is shown for clarity and simplicity.

**Figure 3:**
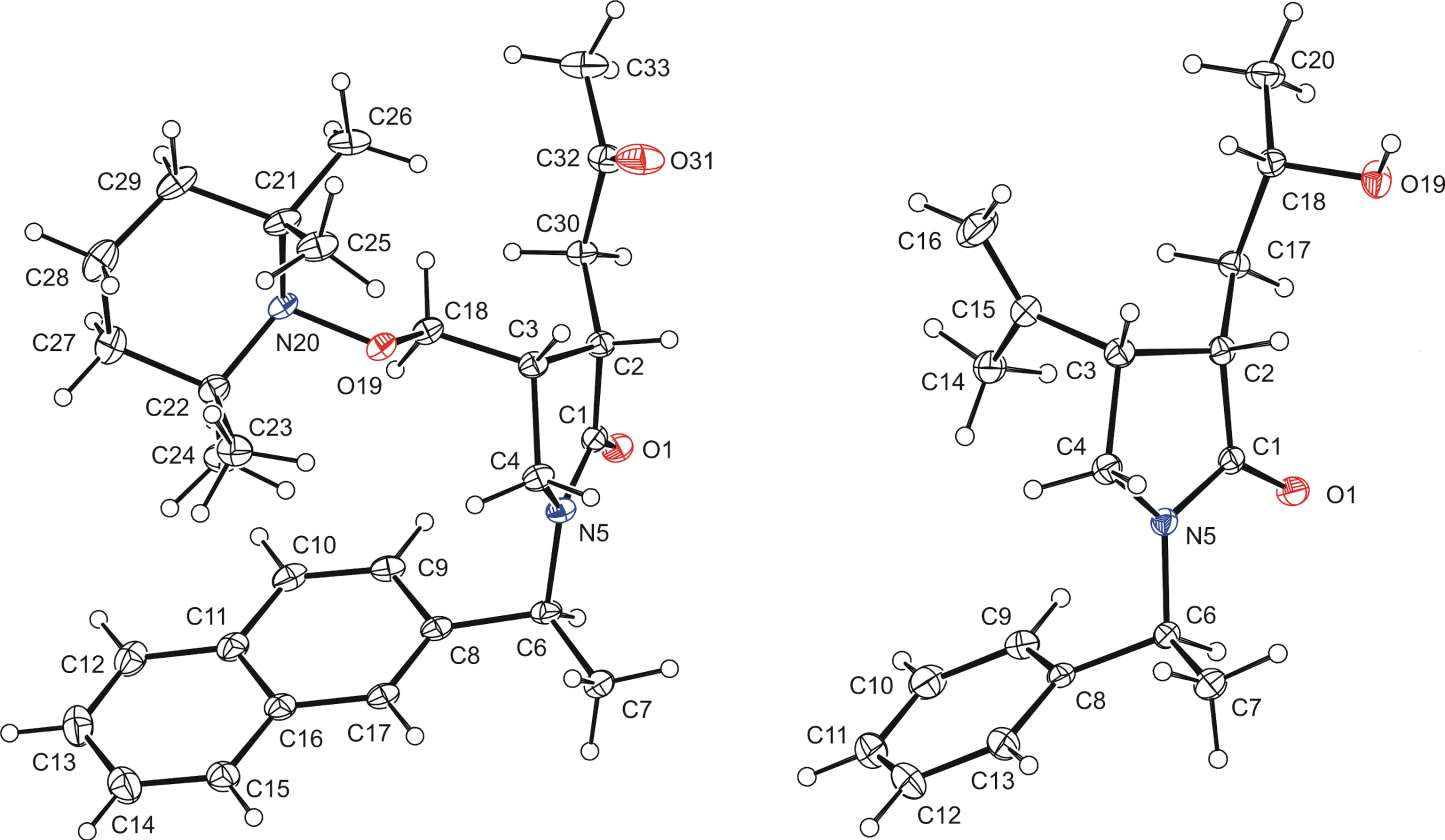
X-ray crystal structure of the minor *cis*-diastereomers of the keto lactam **13j** (left) and the hydroxy lactam **12k** (right). Displacement ellipsoids are drawn at the 30% probability level.

The minor *cis*-diastereomer of *N*-(1-β-naphthylethyl)pyrrolidone **12j** crystallized after oxidation to ketone **13j** and its configuration was unequivocally established by X-ray crystallography (Figure 3, vide infra). Similarly, the minor *cis*-diastereomer of hydroxy lactam **12k** crystallized and its configuration was confirmed. The configuration of the other lactams was assigned by analogy, by base-mediated equilibration and oxidation experiments (vide infra).

**Scheme 3:**
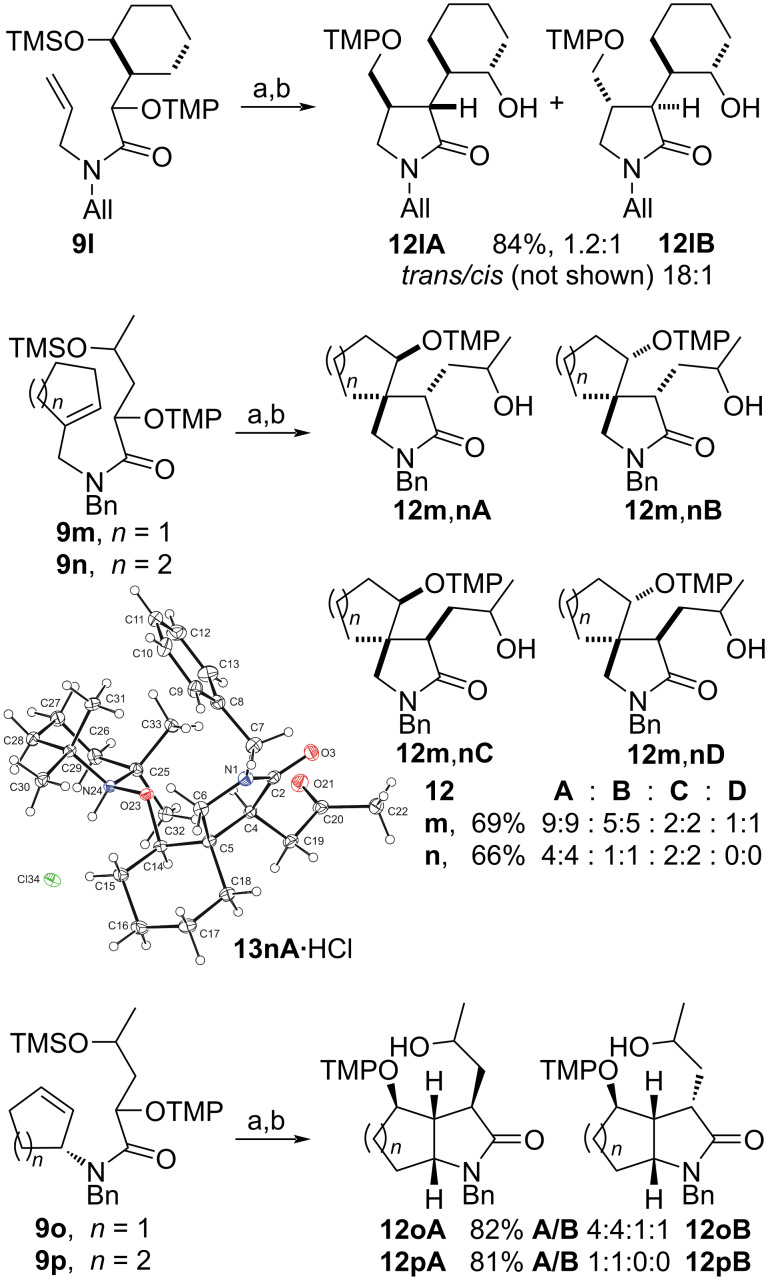
Thermal radical cyclization reactions of amides **9l**–**p** bearing cyclic units. Conditions: a) *t-*BuOH, 150 °C, 1 h; b) TBAF, THF, 0 °C. All = allyl.

The α-(aminoxy)-γ-(silyloxy)amides **9l–p** with cyclic units are also suitable precursors for radical cyclization reactions (Scheme 3). 3-(2-Hydroxycyclohexyl)-2-pyrrolidone **12l** was obtained by the thermal cyclization of α-(aminoxy)cyclohexylacetamide **9l** as a mixture of two major *trans* isomers **12lA** and **12lB**, which were accompanied by traces of a C3–C4 *cis*-diastereomer (not shown). The relative configuration of pyrrolidone **12l** was assigned by NOE experiments of 3-(2-oxocyclohexyl)lactams **13l** prepared from **12l** by Dess–Martin oxidation (vide infra). The thermal cyclization of compounds **9m**,**n** with *N*-cycloalkenylmethyl substituents provided spirolactams **12m**,**n** in good yields, but with overall low diastereoselectivity. In the cyclization of **9m** lactams **12mA** and **12mB** with *trans* orientation at C4 and the newly introduced aminoxy group at C5 were the major diastereomers, which results in an overall 4.5:1 *trans*/*cis* cyclization diastereoselectivity. The radical coupling with TEMPO (**3**) proceeded with moderate 2:1 diastereoselectivity for both pairs of the cyclized diastereomers. In the case of azaspiro[4,5]decanone **12n** with a spirocyclohexyl substituent the cyclization diastereoselectivity was with 2.5:1 lower than that for **12m**. However, the coupling diastereoselectivity with TEMPO (**3**) amounted to 4:1 for the *trans* diastereomers **12nA**,**B** and exclusive for the *cis* isomer **12nC**. The relative configuration of diastereomer **12nA** was unequivocally established by X-ray crystallography of the hydrochloride adduct of the keto lactam **13nA** (Scheme 3, insert). The relative configuration of the compounds **12m**,**n** was determined by analogy, by ROESY investigations for the keto lactams **13m**,**n**, and by isomerization experiments for **12n** (vide infra). Amides **9o**,**p** with cycloalkenyl substituents on the nitrogen were transformed to fused lactams **12o**,**p** with good diastereoselectivity. Azabicyclo[3.3.0]octanone **12o** prepared from amide **9o** with a racemic cyclopent-2-enyl group on nitrogen was obtained as an inseparable mixture of four diastereomers **12oA** and **12oB**. In major **12oA** the hydroxypropyl and tetramethylpiperidinyloxy groups reside on the convex face of the bicyclic system. Amide **9p** with an enantiomerically enriched cyclohex-2-enyl substituent cyclized with exclusive diastereoselectivity and only two diastereomers **12pA** differing in the orientation of the hydroxy group were obtained in high yield. The radical coupling with TEMPO also occurred exclusively at the convex face of the bicyclic system. The configurations of the fused lactams were assigned by chemical derivatization and NOE experiments (vide infra).

### Functionalization reactions of lactams **12**

#### Base-mediated isomerization reactions

Lactams **12** are mixtures of two, four, six or eight diastereomers (vide supra) and the analysis and further application of such diastereomeric mixtures is problematic. To improve the diastereomeric ratio and to establish the relative configuration, lactam mixtures **12** were subjected to epimerization at C3 (Table 4). Indeed, 50 mol % KO*t-*Bu in *t-*BuOH under thermodynamic equilibration conditions at room temperature or at 50 °C for 24 h induced conversion of the minor *cis* isomers to the corresponding *trans* diastereomers in good yield (Table 4, entries 1–4, and 8). Resubjecting several *trans*-enriched compounds to the reaction conditions did not improve the results, indicating that the reactions are at thermodynamic equilibrium after 24 h. Surprisingly, for *N*-methyl-substituted lactam **12e** the diastereomeric enrichment remained rather moderate despite variations of temperature and time (Table 4, entry 5), whereas the diastereomeric ratio of lactam **12f** with a quaternary center at C4 did not change at all, neither at room temperature nor at 50 °C (Table 4, entry 6). Lactam **12g** with a benzyl group as well as pyrrolidones **12i**–**k** bearing branched 1-arylethyl groups on the nitrogen required warming to 50 °C to induce epimerization at C3 (Table 4, entries 9–11). Thus, the nitrogen substituent exerted a significant influence on the facility and position of the *trans*/*cis* equilibrium of the lactams **12**. The influence of an isopropenyl or tetramethylpiperidinyloxymethyl group at C4 has in contrast only little influence on the thermodynamic equilibrium (Table 4, entries 7 and 11 vs entries 1 and 9).

**Table 4:** Isomerization of lactams **12** using KO*t-*Bu in *t-*BuOH^a^.

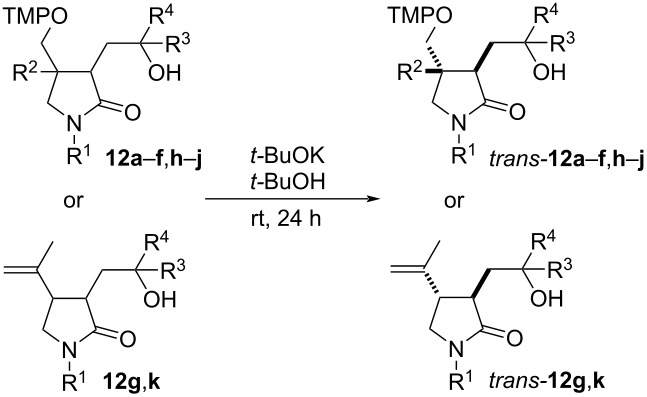							

entry	**12**	R^1^	R^2^	R^3^	R^4^	*trans-***12**	dr

1	**a**	allyl	H	CH_3_	CH_3_	**a**	17:1
2	**b**	allyl	H	CH_3_	H	**b**	12:12:1:1
3	**c**	allyl	H	C_4_H_9_	H	**c**	16:16:1:1
4	**d**	allyl	H	Ph	H	**d**	13:13:1:1
5	**e**	CH_3_	H	CH_3_	H	**e**	4:4:1:1
6^b^	**f**	Bn	CH_3_	CH_3_	H	**f**	3:3:1:1
7^b^	**g**	Bn	H	CH_3_	H	**g**	7:7:1:0
8^b^	**h**	allyl	H	(*S*)-CH_2_OBn	H	**h**	17:17:1:1
9^b^	**i**	(*S*)-PhCHCH_3_	H	(*S*)-CH_3_	H	**i**	1:1:0:0
10^b^	**j**	(*S*)-β-NapCHCH_3_	H	(*S*)-CH_3_	H	**j**	4:4:1:1
11^b^	**k**	*(S*)-PhCHCH_3_	H	(*S*)-CH_3_	H	**k**	1:1:0:0

^a^General conditions: **12** (1 equiv), KO*t-*Bu (0.5 equiv), *t-*BuOH, room temperature, 24 h; ^b^reaction at 50 °C.

Attempts to influence the diastereomeric ratio of the cyclization products by irreversible stoichiometric deprotonation of the lactams **12d**,**f**,**i** by LDA at −78 °C and subsequent protonation by methanol did not lead to substantial changes of the initial diastereomeric ratios. To confirm enolate formation, lactam **12f** was deprotonated by LDA at −78 °C and quenched with D_2_O, resulting in lactam **12f** with 86% deuterium incorporation, but no change in the diastereomeric ratio.

**Scheme 4:**
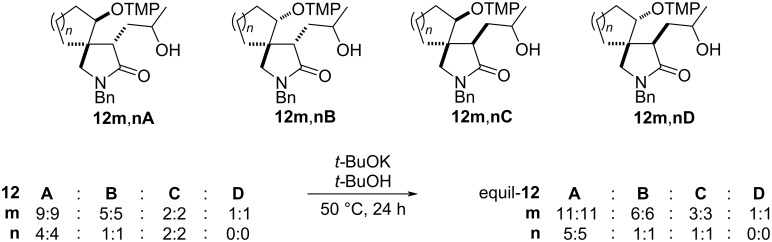
Epimerization of spirolactams **12m**,**n**.

Spirolactams **12m**,**n** were also subjected to epimerization (Scheme 4). The change of the diastereomeric ratio **A**–**D** was not significant for **12m**, whereas during equilibration of **12n** the content of the *cis* isomers **12nC** in the mixture decreased.

#### Oxidation of the side chain hydroxy group at C3

Hydroxy lactams **12** can be transformed to keto pyrrolidones **13** by a Dess–Martin oxidation (Table 5). For the isomerized ketones *trans*-**12b–d** this leads essentially to single keto lactams **13b–d** (Table 5, entries 1, 3, and 4). The non-equilibrated ketones can also be used as exemplified for **12b** providing an unchanged 2.5:1 *trans*/*cis* diastereomeric mixture (Table 5, entry 2, cf. Table 3, entry 2). The *trans* and *cis* orientation of the substituents at C3 and C4 of the pyrrolidone ring of **13b** was confirmed by NOE experiments (see Supporting Information File 1 for details). The *trans*/*cis* mixture of **12f** was similarly oxidized providing the pyrrolidone diastereomers **13f** in good yield and unchanged ratio (Table 5, entry 5). The diastereomerically enriched lactams *trans-***12i**,**j** with 1-arylethyl substituents also provided the unchanged diastereomeric mixtures **13i,j** (Table 5, entries 6 and 7), thus confirming that they have opposite *trans* arrangement at C3 and C4 based on the fixed (*S*)-configuration of the 1-arylethyl substituents. One of the minor *cis* diastereomers of **13j** crystallized and its configuration was unequivocally established by X-ray crystallography (Figure 3, vide supra).

**Table 5:** Oxidation of hydroxy lactams *trans-***12** to keto lactams **13** by the Dess–Martin periodinane^a^.

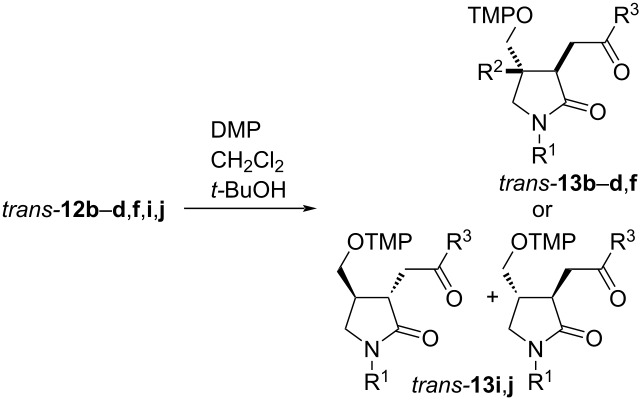						

entry	*trans-***12**	R^1^	R^2^	R^3^	**13**	dr

1	**b**	allyl	H	CH_3_	**b** 88	1:0
2^b^	**b**	allyl	H	CH_3_	**b** 86	2.5:1
3	**c**	allyl	H	C_4_H_9_	**c** 87	1:0
4	**d**	allyl	H	Ph	**d** 80	1:0
5	**f**	Bn	CH_3_	CH_3_	**f** 90	3:1
6	**i**	(*S*)-PhCHCH_3_	H	(*S*)-CH_3_	**i** 77	1:1:0:0
7	**j**	(*S*)-β-NapCHCH_3_	H	(*S*)-CH_3_	**j** 81	4:4:1:1

^a^General conditions: *trans-***12** (1 equiv), DMP (1.3 equiv), *t-*BuOH (10 mol %), CH_2_Cl_2_, room temperature, 1 h; ^b^non-isomerized **12b** was used.

**Scheme 5:**
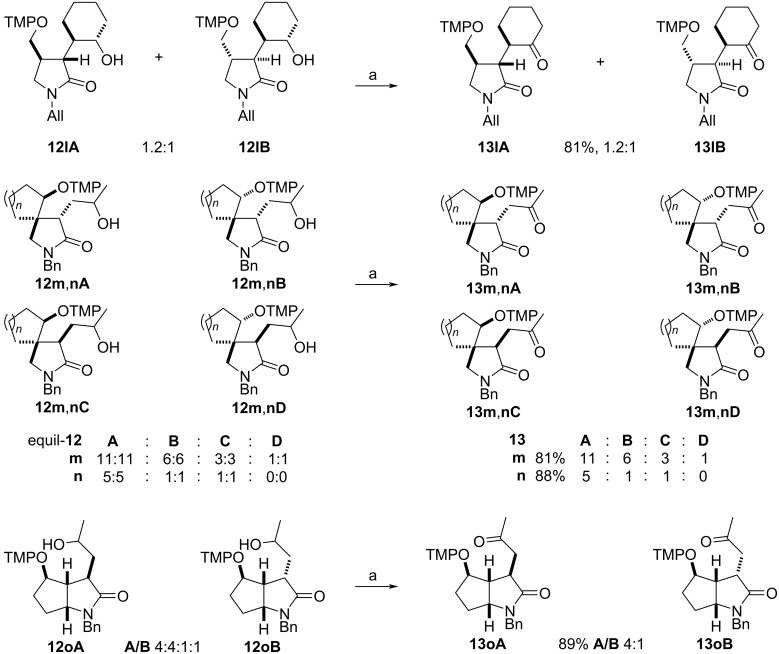
The Dess–Martin oxidation of lactams **12l**–**o**. Conditions: a) DMP (1.3 equiv), *t-*BuOH (10 mol %), CH_2_Cl_2_, room temperature, 1 h.

Lactams **12l**–**o** with cyclic subunits were also subjected to a Dess–Martin oxidation to confirm their relative configuration (Scheme 5). The diastereomeric mixture of lactam **12lA**,**B** gave two *trans*-diastereomers of **13lA**,**B** in an unchanged 1.2:1 ratio. Their opposite relative configuration at C3 and C4 was established by NOE experiments (see Supporting Information File 1). The rather complex diastereomeric mixture of spirolactams *equil-***12m**,**nA–D** simplified on oxidation to partly separable mixtures of four and three diastereomers of keto lactams **13m**,**n**, respectively. During purification of lactam **13m** a major fraction was isolated in a 1.8:1 ratio, whereas the minor consisted of a 3:1 ratio, reflecting the initial **13mA**,**B**:**13mC**,**D** ratio. The mixture of keto lactams **13nA**–**C** was similarly separated to a 5:1 mixture of the lactams **13nA**,**B** and the minor **13nC**, respectively. This lends support to the C3–C4 *trans* arrangement for **13m**,**nA** and **13m**,**nB** as well as to the respective *cis* orientation in **13m**,**nC** and **13mD**. The oxidation of the hydroxy group in the bicyclic annulated racemic compound **12o** reduced the number of diastereomers as expected to two, **13oA** and **13oB**, in a 4:1 ratio. The relative configurations of the major and the minor diastereomers were determined by NOE experiments (see Supporting Information File 1).

#### Further functionalization of lactams **12**

**Scheme 6:**
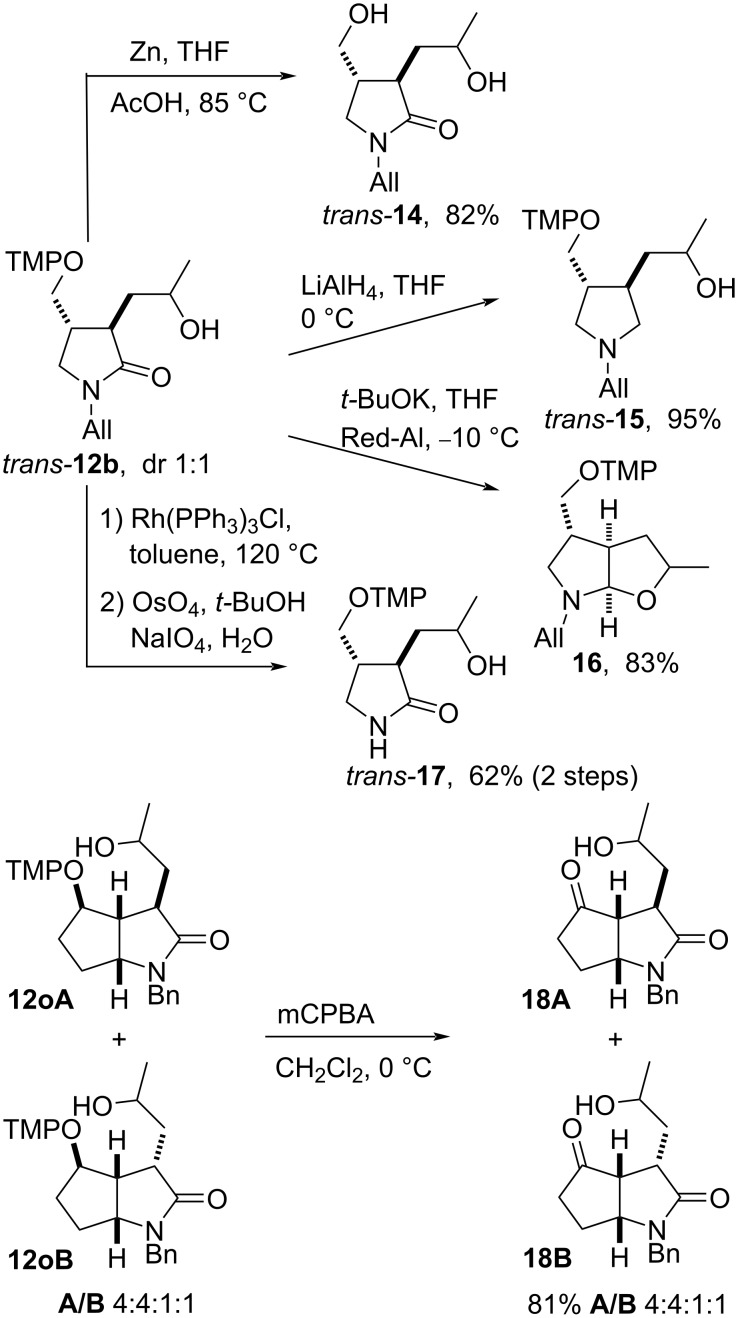
Selected transformations of the lactams *trans-***12b** and **12o**.

Derivatizations of lactams **12** lead to valuable compound classes for further elaboration. The alkoxyamine functionality of lactam *trans-***12b** was reductively cleaved by excess zinc in the presence of acetic acid at elevated temperature (Scheme 6); the dihydroxy lactam **14** was obtained in 82% yield. In the presence of LiAlH_4_ pyrrolidone *trans-***12b** was reduced to pyrrolidine *trans-***15** in 95% yield, whereas no reaction was observed with DIBAL-H, NaBH_4_ or LiHBEt_3_ as the reducing agents; the starting material was typically recovered in 95% yield. However, the reduction of *trans-***12b** by Red-Al in the presence of KO*t-*Bu was effective for the reduction of the lactam function to the hemiaminal followed by a nucleophilic exchange providing bicyclic hemiaminal **16** as 1:1 mixture of diastereomers in 83% yield. The stereochemical assignment of **16** is based on the relative configuration of the starting material. The *N*-allyl group in *trans*-**12b** can be also easily deprotected by a rhodium-catalyzed isomerization to the corresponding *N*-propenyl lactam followed by osmium tetroxide-catalyzed oxidative cleavage, providing lactam *trans-***17** in 62% yield. Additionally, the diastereomeric mixture of lactam **12o** was subjected to oxidative N–O bond cleavage by mCPBA furnishing bicyclo[3.3.0]octandiones **18**. The isolation of a mixture of four diastereomers as in the starting material indicates that radical coupling by TEMPO proceeds with exclusive diastereoselectivity (vide infra).

## Discussion

**Scheme 7:**
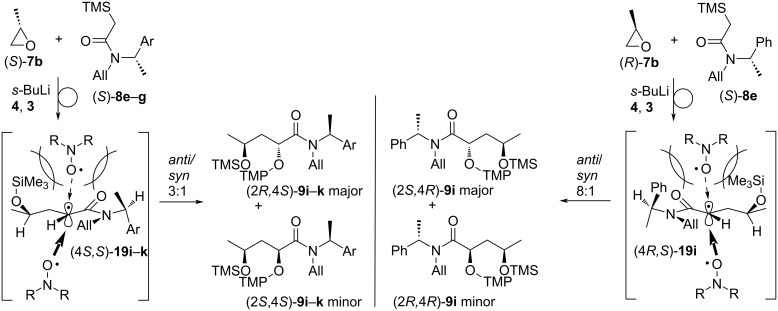
Diastereoselectivity for the formation of α-(aminoxy)amides **9i**–**k**.

The sequence nucleophilic epoxide opening/Brook rearrangement/single electron transfer-induced radical oxygenation proceeds efficiently with silylacetamides **8a**–**g**,**j**,**k** giving α,γ-dioxygenated amides **9a**–**p** in good yields (cf*.* Table 2). This transformation represents a rare geminal C–C/C–O functionalization of the starting silylacetamides **8**. However, neither the silyloxy group in γ-position nor the size of the N-substituent influence the diastereoselectivity of radical coupling with TEMPO (**3**). In contrast, the chiral *N*-(1-phenylethyl)- and *N*-(1-naphthylethyl)-substituted amides **9i**–**k** were obtained from (*S*)-propylene oxide (*S*)-**7b** with moderate 3:1 *anti*/*syn*-diastereoselectivity and from (*R*)-propylene oxide (*R*)-**7b** with good 8:1 *anti*/*syn*-diastereoselectivity. This makes the following oxygenation course most likely (Scheme 7). Silylacetamides **8e**–**g** exist as approximately 4:1 rotameric mixtures of (*Z/E*)-isomers as determined by ^1^H NMR spectroscopy at room temperature and ROESY investigations (see Supporting Information File 1). This corresponds to the previously reported data for *N*-benzylacetamides [82]. It can be assumed that the amide enolates after the Brook rearrangement and the α-amide radicals (4*S,S*)-**19i**–**k** and (4*R,S*)-**19i**, respectively, resulting after SET oxidation have a similarly preferred (*Z*)-orientation, since the environment around the amide does not change significantly during these elementary steps. A zig-zag conformation of the main chain places the bulky silyloxy and the methyl group of the 1-arylethyl unit in (4*S,S*)-**19i**–**k**, but the silyloxy group and the sterically more demanding phenyl ring of the 1-arylethyl group in (4*R,S*)-**19i** at the β-face shielding it in both radicals for the approach of TEMPO (**3**), but significantly more effectively in (4*R,S*)-**19i** (Scheme 7). The α-face is in contrast much less crowded and allows smooth radical coupling providing a good 8:1 *anti*/*syn-*diastereoselectivity for (2*S,*4*R*)-**9i**, but only 3:1 for (2*R,*4*S*)-**9i**–**k**. Thus, the configurations of both, the epoxide **7** and the silylacetamide **8** are important in the nucleophilic ring opening/Brook rearrangement/radical oxygenation sequence for obtaining optimal *anti*-diastereoselectivity. Opposite absolute configurations in both components, **7** and **8**, are displaying a synergistic effect for optimal stereocontrol in the radical oxygenation step with TEMPO (**3**).

**Scheme 8:**
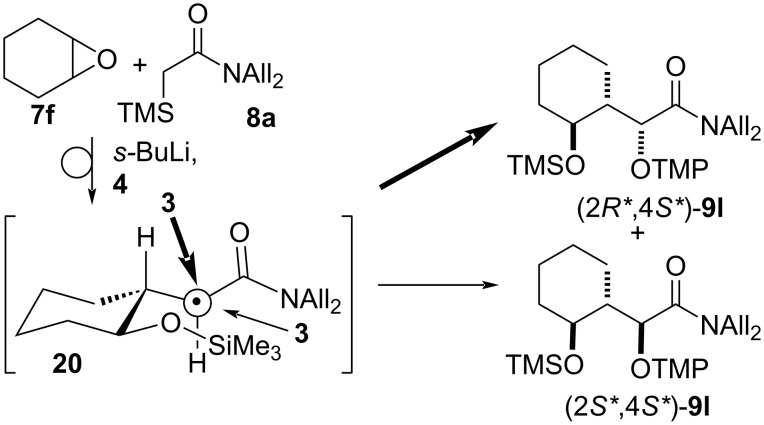
Rationalization of the diastereoselectivity for the formation of the α-(aminoxy)amide **9l**.

A good diastereoselectivity of the oxygenation was also observed for the formation of **9l** (cf. Table 2). Assuming a preferred conformation in radical **20**, in which the interactions of the carbonyl group and the cyclohexane ring are minimized, the β-face at the radical center is significantly blocked by the silyloxy group hindering the approach of TEMPO (**3**), whereas the α-face is free for radical coupling resulting in the formation of (2R*,4S*)-**9l** as the major product (Scheme 8).

**Scheme 9:**
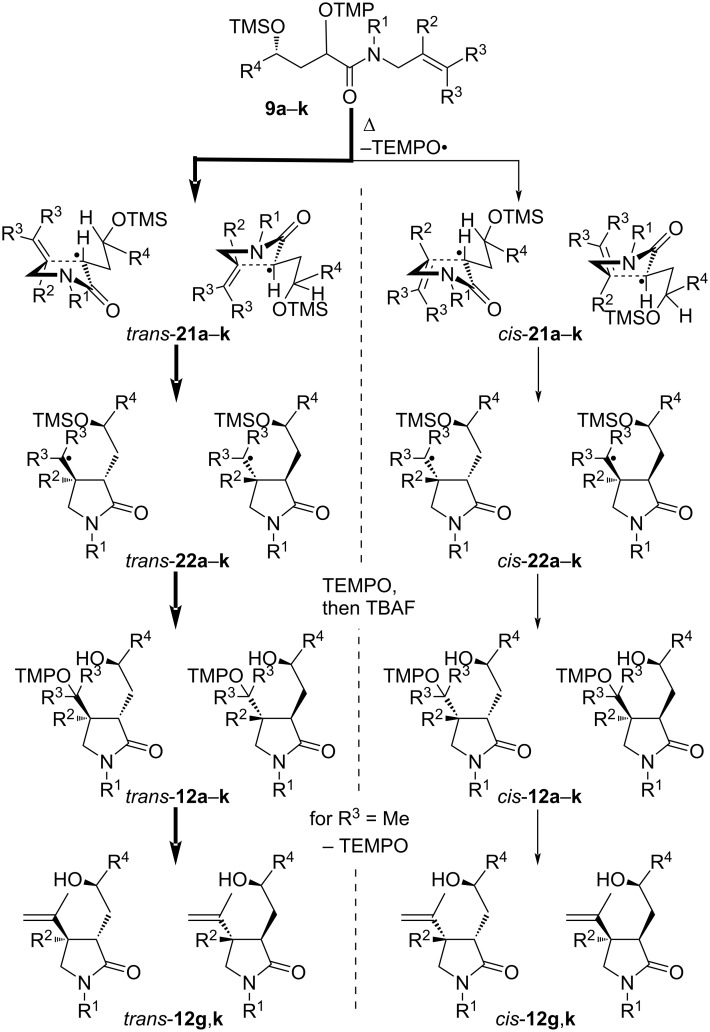
Rationalization of the thermal radical cyclization diastereoselectivity of alkoxyamines **9a**–**k**. (*S*)-Configuration at silyl ether function displayed as for **9i–k** and **12i**–**k**.

The outcome of the thermal radical cyclizations of the dioxygenated amides **9** is dependent on the structure of the alkene unit, but the general trend is similar (Scheme 9). Amides with a terminal alkene unit furnished dioxygenated pyrrolidones **12a–f**,**h**–**j** as products, amides **9g**,**k** with trisubstituted alkene units exclusively lead to isopropenylpyrrolidones **12g**,**k**. A similar reactivity was observed before in thermal radical cyclizations leading to cyclopentane derivatives [74–75]. All γ-silyloxy amides **9a**–**k** cyclize in the 5-*exo* mode via envelope transition states **21a**–**k** in which both the amide resonance and the resonance of the radical with the carbonyl group are disturbed [83–84]. Reactions via transition states *trans-***21a**–**k** are energetically favored over the corresponding sterically more hindered *cis*-oriented transition states *cis*-**21a**–**k**, however, the diastereoselectivity remains moderate under the thermal conditions. This is in line with the previously reported radical cyclization reactions to pyrrolidones [38,85–88]. There is apparently no energy difference between the pairs of *trans*-**21** or *cis*-**21**, thus the γ-silyloxy group exerts no influence. The cyclized radicals **22** couple subsequently with TEMPO providing lactams **12a**–**k** after deprotection of the TMS groups. The cyclized tertiary alkoxyamines (R^3^ = Me) are known to be thermally labile [89–92] and consequently 4-isopropenylpyrrolidones **12g**,**k** are isolated as the exclusive products. The cyclization of the 2-silyloxycyclohexyl-substituted amide **9l** proceeds similarly providing almost equal amounts of two *trans* diastereomers **12l** as single isomers because of the constrained conformation of radical **20**, which allows a cyclization essentially only from the α-face (not shown, cf. Scheme 8).

**Scheme 10:**
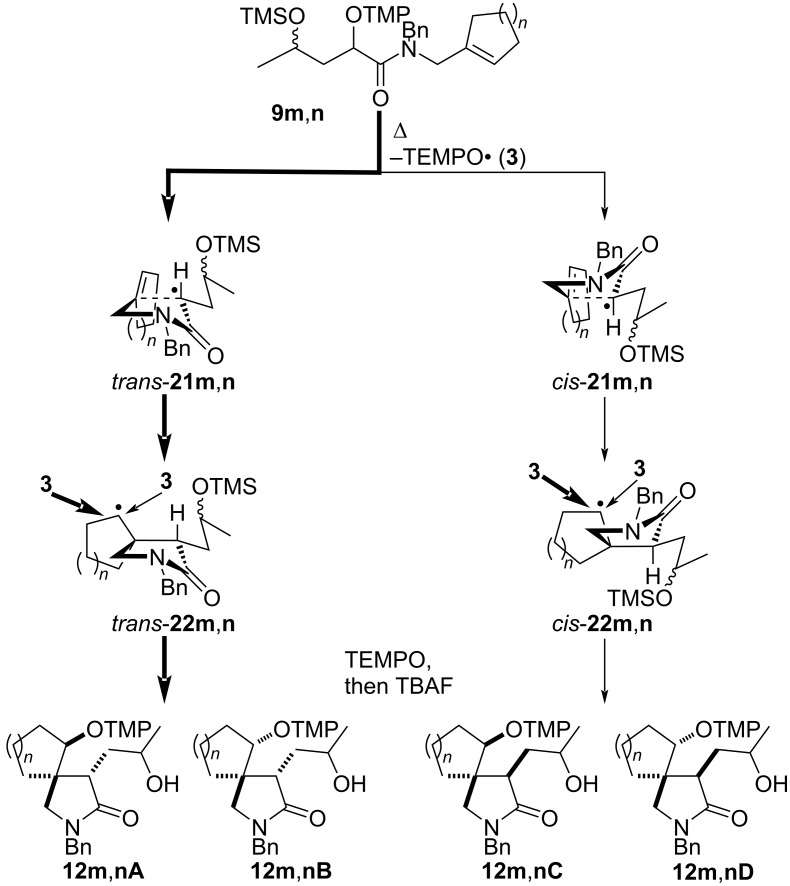
The stereochemical course for the formation of products **12m**,**n** by thermal radical cyclization of alkoxyamines **9m**,**n**.

Amides **9m**,**n** with cyclopent-1-en-1-ylmethyl or cyclohex-1-en-1-ylmethyl substituents on nitrogen similarly cyclize via envelope transition states *trans*-**21m**,**n** and *cis*-**21m**,**n** with preferred *trans* orientation of the olefin unit and the silyloxy-bearing side chain (Scheme 10). Thus, similarly as for **12a**–**k**, two cyclized diastereomeric radicals *trans*-**22m**,**n** and two radicals *cis*-**22m**,**n** result, which differ in their orientation with respect to the racemic silyloxy group (cf. Scheme 9). The situation in the azaspiro[4,4] and azaspiro[4–5] radicals **22m**,**n** is, however, more complex since they are prochiral, and thus eight diastereomers result. The coupling of **22m**,**n** with TEMPO (**3**) occurs predominately from the more accessible β-face of the cyclopentyl or cyclohexyl radicals, since the α-face is partially blocked by the oxygenated alkyl chain at C4. However, the diastereoselectivity is also dependent on the ring size of the spirocyclic radical and proved to be better for the cyclohexyl radicals **22n**, where coupling with TEMPO proceeded with a 4:1 **12nA/12nB** selectivity and exclusive diastereoselectivity for **12nC** (cf. Scheme 3).

**Scheme 11:**
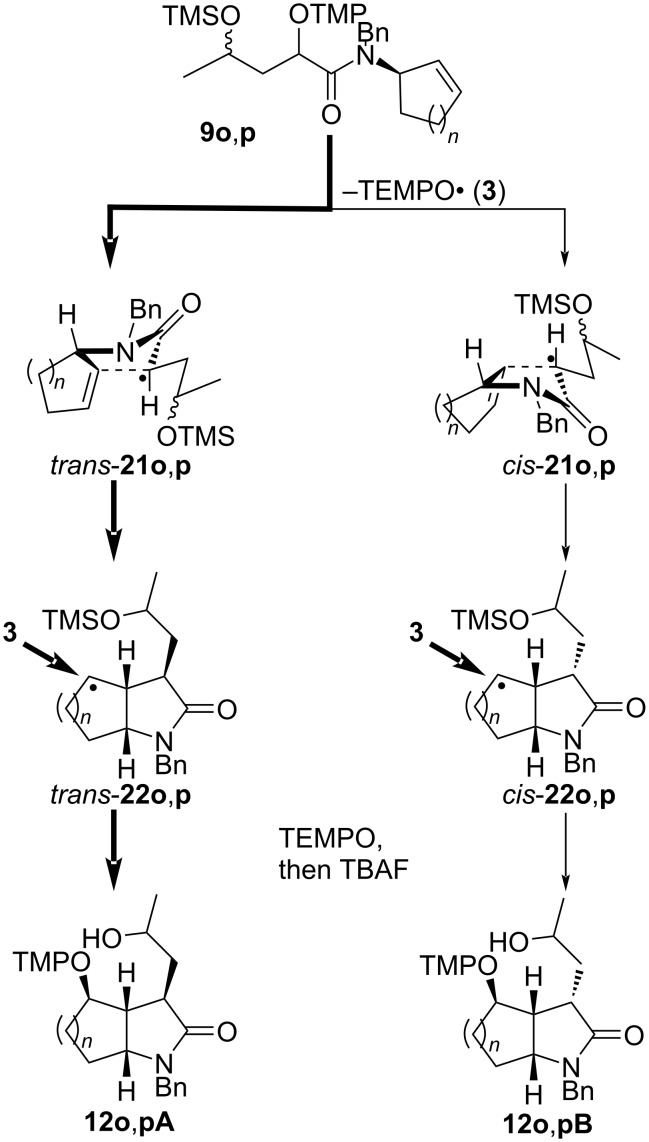
Formation of bicycles **12o**,**p**.

Fused lactams **12o**,**p** were obtained with good to excellent diastereoselectivity from *N-*cyclopent-2-enyl or *N*-cyclohex-2-enyl amides **9o**,**p** (cf. Scheme 3). The 5-*exo* cyclization step of radicals **21o**,**p** proceeds with good to exclusive *trans* diastereoselectivity forming the azabicyclo[3.3.0]octyl or azabicyclo[4.3.0]nonyl radicals **22o**,**p** (Scheme 11). The subsequent coupling of **22o**,**p** with TEMPO (**3**) occurs exclusively from the accessible convex face of the bicyclic radicals providing the lactams **12o**,**p**.

The diastereomeric mixtures resulting from the thermal radical cyclization converge easily to the corresponding *trans* isomers by base-mediated isomerization reactions, if C4 is not disubstituted.

## Conclusion

We developed a two-step methodology for the synthesis of diverse γ-lactam scaffolds. A tandem reaction combining nucleophilic epoxide opening by *N*-allylic silylacetamides, Brook rearrangement, and radical oxygenation serves for the preparation of *N*-allylic α-(aminoxy)-γ-(silyloxy)amides **9**, which represents an oxidative C–C/C–O difunctionalization at the α-position of the amides. With correct configuration combination of chiral epoxides **7** and chiral amides **8**, 2,4-dioxygenated amides **9** can be obtained with good *anti*-diastereoselectivity and enantioselectivity. Dioxygenated amides **9** are convenient precursors for radical 5-*exo* cyclization reactions based on the persistent radical effect. The *N*,3,4-trisubstituted lactams **12** were obtained in good yields, but with moderate *trans*/*cis* diastereoselectivity. The use of *N*-cycloalkenyl amides enables access to fused and spirolactams. The diastereomeric mixtures resulting from thermal radical cyclization converge to *trans*-3,4-disubstituted lactams by basic epimerization in 3-position of the lactam under thermodynamic conditions. The pyrrolidones can be easily further diversified by oxidation and reduction reactions. Thus, this methodology is suitable for the synthesis of functionalized γ-lactams, which can be used as building blocks for the synthesis of natural products or biologically active compounds.

## Experimental

### Tandem nucleophilic epoxide opening/Brook rearrangement/α-oxygenation (general procedure)

In a similar manner as described in [74]: LiCl (252 mg, 6 mmol) was added to a round-bottomed flask containing a stirring bar, which was sealed with a septum, and dried under vacuum by a heat gun. Dry THF (8 mL) and amide **8** (1.0 mmol) were added under argon. The mixture was cooled to 0 °C in an ice/water bath, *sec*-butyllithium (1.4 M solution in cyclohexane, 0.8 mL, 1.1 mmol) was added dropwise by a syringe, and the mixture was stirred at 0 °C for 15 min. Then, the epoxide **7** (1.05 mmol) was added at once by syringe and the reaction mixture was stirred at 0 °C for 1 h. The reaction mixture was cooled to −78 °C, diluted with dry THF (8 mL), and TEMPO (**3**, 164 mg, 1.05 mmol) was added as a solid in a single portion. Ferrocenium hexafluorophosphate (**4**, 397 mg, 1.2 mmol) was added in small portions with vigorous stirring until a dark blue-green color of the reaction mixture persisted for 20 min. The reaction mixture was quenched by saturated NH_4_Cl solution (5 drops), diluted with diethyl ether (10 mL), and filtered through a pad of silica gel, which was washed with a fresh portion of diethyl ether. The filtrate was evaporated, and the crude inhomogeneous mixture was purified by flash chromatography (gradient, hexanes/EtOAc 20:1 to 5:1) to give pure α-(aminoxy)amides **9**.

### Thermal radical cyclization of compounds **9** and further deprotection (general procedure)

The α-(aminoxy)amide **9** (0.65 mmol) was heated in *t-*BuOH (6 mL) in a microwave reactor at 150 °C for 1 h. The reaction mixture was diluted with diethyl ether (5 mL), transferred into a round-bottomed Schlenk flask and evaporated. The crude residue was dissolved in THF (5 mL), the reaction mixture was cooled to 0 °C in an ice/water bath, tetrabutylammonium fluoride (1 M solution in THF, 0.96 mL, 0.96 mmol) was added and the mixture was stirred at this temperature for 30 min. The reaction was quenched by saturated NH_4_Cl solution, diluted with water (5 mL) and diethyl ether (5 mL), the organic layer separated, and the aqueous layer extracted with diethyl ether (2 × 5 mL). The combined organic extracts were dried over MgSO_4_, filtered, and evaporated. The crude mixture was purified by column chromatography (gradient, hexanes/EtOAc 5:1 to 1:1) to give pure lactams **12** as diastereomeric mixtures.

### Equilibration of lactams **12** (general procedure)

A solution of KO*t-*Bu (1 M in THF, 0.25 mL, 0.25 mmol) was added to a stirred solution of hydroxy lactam **12** (0.50 mmol) in *t-*BuOH (3 mL) at room temperature or 50 °C and the reaction mixture was stirred for 24 h. The reaction was quenched by the addition of saturated NH_4_Cl solution and diluted with water (3 mL) and diethyl ether (5 mL). The organic layer was separated and the aqueous layer was extracted with diethyl ether (3 × 5 mL). The combined organic extracts were dried over MgSO_4_ and filtered. The filtrate was evaporated and the diastereomeric ratio was determined by ^1^H NMR spectroscopy. The crude mixture was purified by flash chromatography (gradient, hexanes/EtOAc 10:1 to 1:1) to give lactam *trans***-12**.

### Dess–Martin oxidation of hydroxylactams **12** (general procedure)

A solution of hydroxy lactam **12** or *trans***-12** (0.7 mmol) in dichloromethane (4 mL) was added to a stirred solution of Dess–Martin periodinane (386 mg, 0.9 mmol) and *t-*BuOH (0.1 mL, 1.1 mmol) in dichloromethane (4 mL) at room temperature. After 30 min, saturated Na_2_CO_3_ solution (2 mL) and saturated Na_2_S_2_O_3_ solution (2 mL) were added. After 5 min of vigorous stirring, the mixture was diluted with dichloromethane (10 mL), the organic layer was separated, washed with brine, dried over MgSO_4_, and the solvent was evaporated. The residue was purified by column chromatography (gradient, hexanes/EtOAc 10:1 to 1:1) to give keto lactam **13**.

## Supporting Information

File 1 Experimental details and spectral data.
