# Supplementary material for: α,γ-Dioxygenated amides via tandem Brook rearrangement/radical oxygenation reactions and their application to syntheses of γ-lactams
Source: Beilstein J Org Chem. 2021 Mar 9;17:688–704. doi: 10.3762/bjoc.17.58 (PMC7961876; doi:10.3762/bjoc.17.58)
Supplement: File 1 — Experimental details and spectral data. [file Beilstein_J_Org_Chem-17-688-s001.pdf]

BEILSTEIN JOURNAL OF ORGANIC CHEMISTRY

Supporting Information

for

α,γ-Dioxygenated amides via tandem Brook rearrangement/ radical oxygenation reactions and their application to syntheses of γ-lactams

Mikhail K. Klychnikov, Radek Pohl, Ivana Císařová and Ullrich Jahn

Beilstein J. Org. Chem. **2021,** *17,* 688–704. doi:10.3762/bjoc.17.58

Experimental details and spectral data

License and Terms: This is a supporting information file under the terms of the Creative Commons Attribution License (https://creativecommons.org/licenses/by/4.0). Please note that the reuse, redistribution and reproduction in particular requires that the author(s) and source are credited and that individual graphics may be subject to special legal provisions.
The license is subject to the *Beilstein Journal of Organic Chemistry* terms and conditions: (https://www.beilstein-journals.org/bjoc/terms)

Table of contents

1. Experimental procedures and analytical data S2
2. X-Ray crystallography S108
3. References S110
4. ^{1}H and ^{13}C NMR spectra S111

1. Experimental procedures and analytical data

General experimental conditions

Reactions not involving aqueous conditions were performed in flame-dried glassware under an argon atmosphere. Solvents and additives were dried prior to use according to standard procedures. TLC analyses were performed on POLYGRAM SIL G/UV254 plates. Chromatographic separations were carried out on silica gel 60 (Fluka, 230–400 mesh). IR spectra were measured on a Bruker ALPHA-FT-IR spectrometer as neat samples using an ATR device. The optical rotation was measured on an automatic polarimeter Autopol IV, Rudolph Research Analytical. ^{1}H and ^{13}C NMR spectra were recorded on Bruker Avance 400, 500, or 600 spectrometers at working frequencies of 400, 500, or 600 MHz for ^{1}H NMR spectra and 100.1, 125.7 or 150.9 MHz for ^{13}C NMR spectra. Connectivities were determined by ^{1}H-^{1}H COSY and ^{1}H-^{13}C HMBC experiments, ^{1}H-^{13}C assignments were obtained from ^{1}H-^{13}C HSQC measurements. The relative configurations were determined by H,H-ROESY experiments. *N*,*N*-Diallylamine (**S1**) and the epoxides **7a**–**f** are commercially available. Compounds benzyl-2-methylprop-2-en-1-amine (**S2**),[1] *N*-benzyl-3-methylbut-2-en-1-amine (**S3**),[2] (*S*)-*N*-(1-phenylethyl)prop-2-en-1-amine (**S4**),[3] (*S*)-2-methyl-*N*-(1-phenylethyl)prop-2-en-1-amine (**S5**),[4] (*S*)-*N*-(cyclopent-1-en-1-ylmethyl)-1-phenylethan-1-amine (**S6**),[5] *N*-benzyl-1-(cyclohex-1-en-1-yl)methanamine (**S7**),[2] *N*-benzylcyclopent-2-en-1-amine (**S8**),[6] (*R*)-*N*-benzylcyclohex-2-en-1-amine (**S9**),[6] *N*,*N*-diallylacetamide (**11a**),[7] *N*-allyl-*N*-methylacetamide (**11b**)[8] were prepared according to the literature. Their spectral and physical data matched those reported.

(*S*)-*N*-Allyl-*N*-(1-(naphthalen-2-yl)ethyl)amine (S10):

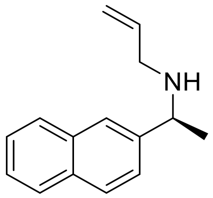

n-Butyllithium (1.6 M in hexane, 11.0 mL, 17.5 mmol) was added to a stirred solution of (*S*)-1-(naphth-2-yl)ethyl-1-amine (3.0 g, 7.5 mmol) in THF (56 mL) at −78 °C. After 40 min, a solution of allyl bromide (1.4 mL, 16.6 mmol) in dry THF (5 mL) was added dropwise and stirring was continued for 3 h. The reaction was quenched by saturated NH_4Cl solution and diluted with water (30 mL) and diethyl ether (50 mL). The organic layer was separated and the

aqueous phase was extracted with diethyl ether (3 × 20 mL). The combined organic layers were dried over $MgSO_4$ and filtered. The filtrate was evaporated and the crude product was purified by flash chromatography (gradient, hexanes/EtOAc + 1% Et_3N 20:1 to 4:1) to give 2.9 g (78%) **S10**.

[R_f (hexanes/EtOAc 1:1) = 0.44]; IR (film); ν [cm^{-1}]: 3336 (w), 3054 (w), 3010 (w), 2972 (m), 2924 (w), 2866 (w), 2816 (w), 1624 (w), 1601 (w), 1507 (w), 1452 (w), 1416 (m), 1370 (w), 1270 (w), 1177 (m), 1130 (w), 1062 (w), 1018 (w), 994 (m), 917 (w), 856 (m), 819 (s), 747 (s), 689 (w), 618 (w); MS (+ESI) m/z, (%): 212 (10, [$M+H^+$]), 155 (100, [$M–CH_2=CHCH_2NH]^+$); HRMS (+ESI) m/z [$C_{15}H_{18}N^+$]: calcd. 212.1434; found 212.1435; $[\alpha]_D^{20}$: –54.5 (c 1.016, $CHCl_3$); ^{1}H NMR (400 MHz, $CDCl_3$): δ 1.45 (d, *J* = 6.7 Hz, 3H, CH_3), 1.52 (br. s, 1H, NH), 3.14 (dt, *J* = 6.0, 1.5 Hz, 2H, $C\underline{H}_2CH=$), 3.99 (q, *J* = 6.6 Hz, 1H, $C\underline{H}CH_3$), 5.06-5.18 (m, 2H, $CH=C\underline{H}_2$), 5.92 (ddt, *J* = 17.2, 10.1, 5.9 Hz, 1H, $C\underline{H}=CH_2$), 7.41-7.55 (m, 3H, Ar$\underline{H}$), 7.74 (s, 1H, Ar$\underline{H}$), 7.80-7.85 (m, 3H, Ar$\underline{H}$); ^{13}C NMR (101 MHz, $CDCl_3$): δ 24.4 (q, CH_3), 50.4 (t, $\underline{C}H_2CH=$), 57.8 (d, $\underline{C}HCH_3$), 115.9 (t, $CH=\underline{C}H_2$), 125.0 (d, CH_{Ar}), 125.4 (d, CH_{Ar}), 125.6 (d, CH_{Ar}), 126.1 (d, CH_{Ar}), 127.8 (d, CH_{Ar}), 127.9 (d, CH_{Ar}), 128.4 (d, CH_{Ar}), 133.0 (s, C_{Ar}), 133.6 (s, C_{Ar}), 137.1 (d, $\underline{C}H=CH_2$), 143.0 (s, C_{Ar}).

***N*-Benzyl-*N*-(cyclopent-1-en-1-ylmethyl)amine (S11):**

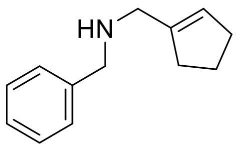

A solution of 1-(bromomethyl)cyclopent-1-ene (4.1 g, 25.5 mmol) in MeCN (5 mL) was added to a solution of benzylamine (8.2 g, 76.6 mmol) and K_2CO_3 (4.2 g, 30.6 mmol) in MeCN (40 mL) and the reaction mixture was stirred at room temperature overnight. The reaction mixture was diluted with water (50 mL) and extracted with EtOAc (3 × 20 mL). The combined organic layers were dried over $MgSO_4$ and filtered. The filtrate was evaporated and the crude product was purified by flash column chromatography (hexane/EtOAc + 1% Et_3N 5:1 to 2:1) to give 3.6 g (75%) **S11**.

HRMS (+ESI) m/z [$C_{13}H_{18}N^+$]: calcd. 188.1434; found 188.1434.

The analytical data are in agreement with those in the literature.[9]

(*S*)-3-Methyl-*N*-(1-phenylethyl)but-2-en-1-amine (S12):

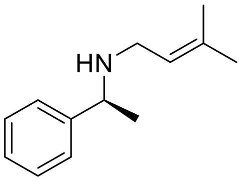

Prepared according to the procedure for **S11**, yield 2.3 g (73%).

[R_f (hexanes/EtOAc 2:1) = 0.26]; IR (film); ν [cm^{-1}]: 3334 (br), 3062 (w), 3025 (w), 2967 (m), 2926 (m), 2857 (w), 1492 (w), 1450 (m), 1375 (w), 1351 (w), 1302 (w), 1187 (w), 1116 (w), 1074 (w), 1055 (w), 1027 (w), 986 (w), 835 (w), 760 (m), 700 (s); MS (+ESI) m/z, (%): 212 (65, $[M+Na^+]$), 190 (100, $[M+H^+]$); HRMS (+ESI) m/z $[C_{13}H_{20}N^+]$: calcd. 190.1596; found 190.1594; $[\alpha]_D^{20}$: –69.9 (c 1.000, $CHCl_3$); ^{1}H NMR (400 MHz, $CDCl_3$): δ 1.22 (br. s, 1H, NH), 1.30 (d, *J* = 6.6 Hz, 3H, $CHC\underline{H}_3$), 1.48 (s, 3H, $CH=C(C\underline{H}_3)_2$), 1.65 (s, 3H, $CH=C(C\underline{H}_3)_2$), 2.99 (d, *J* = 7.0 Hz, 2H, $C\underline{H}_2CH=$), 3.73 (q, *J* = 6.6 Hz, 1H, $C\underline{H}CH_3$), 5.20 (tquint, *J* = 7.0, 1.4 Hz, 1H, $C\underline{H}=C(CH_3)_2$), 7.15-7.22 (m, 1H, $Ar\underline{H}$), 7.24-7.31 (m, 4H, $Ar\underline{H}$); ^{13}C NMR (101 MHz, $CDCl_3$): δ 18.0 (q, $CH=C(\underline{C}H_3)_2$), 24.5 (q, $CH\underline{C}H_3$), 25.9 (q, $CH=C(\underline{C}H_3)_2$), 45.4 (t, $\underline{C}H_2CH=$), 123.3 (d, $\underline{C}H=C(CH_3)_2$), 126.7 (d, CH_{Ar}), 127.0 (d, CH_{Ar}), 128.5 (d, CH_{Ar}), 134.4 (s, $CH=\underline{C}(CH_3)_2$), 145.9 (s, C_{Ar}).

General procedure for preparation of amides 11:

Acetyl chloride (1.6 g, 20.5 mmol) was added to a stirred solution of amines **S1–S12** (17 mmol) and triethylamine (2.4 mL, 17 mmol) in dry dichloromethane (15 mL) at 0 °C. After 30 min, the reaction was quenched by saturated NH_4Cl solution. After diluting with water (20 mL) and dichloromethane (30 mL), the organic layer was separated and the aqueous was extracted with dichloromethane (3 × 10 mL). The combined organic layers were dried over $MgSO_4$ and filtered. The filtrate was evaporated and the crude product was purified by flash chromatography (gradient, hexanes/EtOAc 20:1 to 2:1) to give pure amides **11**.

_N_-Benzyl-_N_-(2-methylallyl)acetamide (11c):

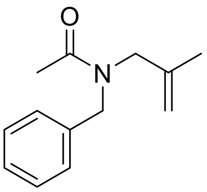

Prepared according to the general procedure, yield 3.4 g (98%) as a 1.7:1 mixture of rotamers. [R_f (hexanes/EtOAc 3:1) = 0.46]; IR (film); ν [cm^{-1}]: 3032 (w), 2974 (w), 2939 (w), 1636 (s), 1497 (m), 1448 (s), 1404 (w), 1376 (w), 1322 (w), 1299 (w), 1267 (w), 1237 (w), 1208 (m), 1183 (m), 1049 (w), 1012 (w), 905 (m), 782 (m), 747 (m), 698 (s); MS (+ESI) m/z, (%): 226 (60, [$M+Na^+$]), 204 (100, [$M+H^+$]); HRMS (+ESI) m/z [$C_{13}H_{18}NO^+$]: calcd. 204.1388; found 204.1390; ^{1}H NMR (400 MHz, $CDCl_3$): δ 1.69/1.70 (s, 3H, CH_3C=), 2.14/2.17 (s, 3H, CH_3CO), 3.70/3.98 (s, 2H, NCH_2C=), 4.48/4.58 (s, 2H, $C\underline{H}_2Ph$), 4.73/4.83 (dsext, *J* = 1.6, 0.8 Hz/dquint, *J* = 1.7, 0.9 Hz, 1H, C=CH_2), 4.90/4.96 (sept, *J* = 1.4 Hz/quint, *J* = 1.5 Hz, 1H, C=CH_2), 7.14-7.42 (m, 5H, Ar$\underline{H}$); ^{13}C NMR (101 MHz, $CDCl_3$): δ 20.2 (q, $\underline{C}H_3C$=), 21.5/21.8 (q, $\underline{C}H_3CO$), 48.2/50.7 (t, $\underline{C}H_2Ph$), 50.4/53.1 (t, $N\underline{C}H_2C$=), 111.6/112.5 (t, C=$\underline{C}H_2$), 126.4/127.7 (d, CH_{Ar}), 127.5/128.4 (d, CH_{Ar}), 128.7/129.1 (d, CH_{Ar}), 137.7 (s, C_{Ar}), 139.8 (s, $\underline{C}$=CH_2), 171.4/177.4 (s, C=O).

_N_-Benzyl-_N_-(3-methylbut-2-en-1-yl)acetamide (11d):

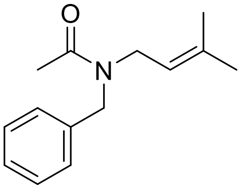

Prepared according to the general procedure, yield 3.6 g (97%) as a 1.3:1 mixture of rotamers. [R_f (hexanes/EtOAc 3:1) = 0.44]; IR (film); ν [cm^{-1}]: 2977 (w), 2929 (w), 1636 (s), 1492 (w), 1449 (m), 1415 (m), 1372 (w), 1354 (w), 1339 (w), 1301 (w), 1263 (w), 1204 (w), 1173 (w), 1055 (w), 1030 (w), 989 (w), 964 (w), 842 (w), 788 (w), 771 (w), 761 (m), 736 (w), 699 (m); MS (+ESI) m/z, (%): 240 (55, [$M+Na^+$]), 218 (100, [$M+H^+$]); HRMS (+ESI) m/z [$C_{14}H_{20}NO^+$]: calcd. 218.1545; found 218.1548; ^{1}H NMR (400 MHz, $CDCl_3$): δ 1.54/1.70 (s, 3H, CH=C($C\underline{H}_3$)$_2$), 1.58/1.72 (s, 3H, CH=C($C\underline{H}_3$)$_2$), 2.13/2.17 (s, 3H, CH_3CO), 3.77/3.99 (d, *J* = 6.8 Hz/*J* = 6.6 Hz, 2H, $C\underline{H}_2CH$=), 4.47/4.56 (s, 2H, $C\underline{H}_2Ph$), 5.06-5.11/5.14-5.19 (m, 1H, C$\underline{H}$=C(CH_3)$_2$), 7.14-7.41 (m, 5H, Ar$\underline{H}$); ^{13}C NMR (101 MHz, $CDCl_3$): δ 17.9/18.0 (q,

$CH=C(\underline{C}H_3)_2$), 21.8/21.9 (q, $\underline{C}H_3CO$), 25.8/25.9 (q, $CH=C(\underline{C}H_3)_2$), 42.9/45.8 (t, $\underline{C}H_2CH=$), 48.0/51.0 (t, $\underline{C}H_2Ph$), 119.79/119.82 (d, $CH_2\underline{C}H=$), 126.5/127.6 (d, CH_{Ar}), 127.4/128.3 (d, CH_{Ar}), 128.6/129.0 (d, CH_{Ar}), 136.1/136.6 (s, $CH=\underline{C}(CH_3)_2$), 137.1/137.9 (s, C_{Ar}), 170.7/170.9 (s, C=O).

(*S*)-*N*-Allyl-*N*-(1-phenylethyl)acetamide (11e):

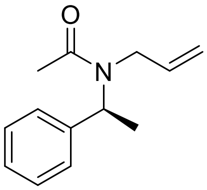

Prepared according to the general procedure, yield 3.4 g (98%) as a 2.7:1 mixture of rotamers. HRMS (+CI) m/z [$C_{13}H_{18}NO^+$]: calcd. 204.1388; found 204.1389; $[\alpha]_D^{20}$: –162.7 (c 1.000, $CHCl_3$). The analytical data are in agreement with those in the literature.[10]

(*S*)-*N*-Allyl-*N*-(1-(naphthalen-2-yl)ethyl)acetamide (11f):

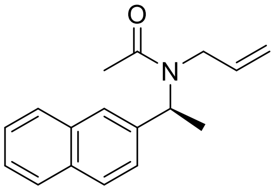

Prepared according to the general procedure, yield 4.3 g (99%) as a 3.1:1 mixture of rotamers. [R_f (hexanes/EtOAc 2:1) = 0.44]; IR (film); ν [cm^{-1}]: 3055 (w), 2977 (w), 2936 (w), 1631 (s), 1406 (s), 1318 (w), 1259 (w), 1189 (m), 1032 (w), 963 (w), 920 (w), 821 (m), 749 (m), 630 (w), 602 (w); MS (+ESI) m/z, (%): 529 (20, [$2M+Na^+$]), 276 (100, [$M+Na^+$]); HRMS (+ESI) m/z [$C_{17}H_{19}NONa^+$]: calcd. 276.1359; found 276.1362; $[\alpha]_D^{20}$: –222.0 (c 1.009, $CHCl_3$); ^1H NMR (400 MHz, $CDCl_3$): δ 1.61/1.73 (d, *J* = 7.1 Hz/*J* = 7.0 Hz, 3H, $CHC\underline{H}_3$), 2.15/2.28 (s, 3H, CH_3CO), 3.47/3.59 (dd, *J* = 15.4, 6.4 Hz/*J* = 17.9, 5.2 Hz, 1H, $C\underline{H}_2CH=$), 3.71/4.18 (dd, *J* = 17.9, 5.1 Hz/*J* = 15.4, 4.8 Hz, 1H, $C\underline{H}_2CH=$), 4.95-5.10 (m, 2H, $CH=C\underline{H}_2$), 5.25/6.28 (q, *J* = 6.9 Hz/*J* = 7.2 Hz, 1H, $C\underline{H}CH_3$), 5.51-5.63/5.73-5.85 (m, 1H, $C\underline{H}=CH_2$), 7.32-7.53 (m, 3H, $Ar\underline{H}$), 7.68/7.74 (s, 1H, $Ar\underline{H}$), 7.76-7.87 (m, 3H, $Ar\underline{H}$); ^{13}C NMR (101 MHz, $CDCl_3$): δ 16.8/19.0 (q, $CH\underline{C}H_3$), 22.4 (q, $\underline{C}H_3CO$), 45.5/46.8 (t, $\underline{C}H_2CH=$), 51.0/56.8 (d, $\underline{C}HCH_3$), 116.2/116.6 (t, $CH=\underline{C}H_2$), 125.1/125.8 (d, CH_{Ar}), 125.2/126.1 (d, CH_{Ar}), 126.3/126.4 (d, CH_{Ar}), 126.5/126.6 (d, CH_{Ar}), 127.69/127.73 (d, CH_{Ar}), 128.05/128.09 (d, CH_{Ar}), 128.3/128.7 (d, CH_{Ar}), 132.78/132.82 (s, C_{Ar}), 133.27/133.33 (s, C_{Ar}), 135.2 (d, $\underline{C}H=CH_2$), 138.2/138.6 (s, C_{Ar}), 170.7/171.6 (s, C=O).

(*S*)-*N*-(3-Methylbut-2-en-1-yl)-*N*-(1-phenylethyl)acetamide (11g):

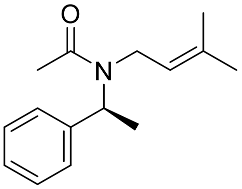

Prepared according to the general procedure, yield 3.9 g (99%) as a 2.9:1 mixture of rotamers. [R_f (hexanes/EtOAc 2:1) = 0.23]; IR (film); ν [cm^{-1}]: 2974 (w), 2932 (w), 1637 (s), 1495 (w), 1447 (m), 1412 (m), 1377 (w), 1363 (w), 1333 (w), 1306 (w), 1259 (w), 1210 (w), 1170 (w), 1056 (w), 1029 (w), 988 (w), 965 (w), 843 (w), 786 (w), 770 (w), 760 (m), 732 (w), 700 (m), 595 (w); MS (+ESI) m/z, (%): 254 (40, [$M+Na^+$]), 232 (100, [$M+H^+$]); HRMS (+ESI) m/z [$C_{15}H_{22}NO^+$]: calcd. 232.1701; found 232.1699; $[\alpha]_D^{20}$: –115.9 (c 1.000, $CHCl_3$); ^{1}H NMR (400 MHz, $CDCl_3$): δ 1.51/1.52 (d, *J* = 7.0 Hz, 3H, $CHC\underline{H}_3$), 1.53/1.64 (s, 3H, $CH=C(C\underline{H}_3)_2$), 1.63/1.66 (s, 3H, $CH=C(C\underline{H}_3)_2$), 2.12/2.23 (s, 3H, CH_3CO), 3.46/3.53 (dd, *J* = 15.5, 6.0 Hz/*J* = 17.3, 5.8 Hz, 1H, $C\underline{H}_2CH=$), 3.69/4.07 (dd, *J* = 17.2, 6.2 Hz/*J* = 15.4, 6.0 Hz, 1H, $C\underline{H}_2CH=$), 4.85-4.97/5.05-5.15 (m, 1H, $C\underline{H}=C(CH_3)_2$), 5.10/6.09 (q, *J* = 7.0 Hz, 1H, $C\underline{H}CH_3$), 7.25-7.41 (m, 5H, $Ar\underline{H}$); ^{13}C NMR (101 MHz, $CDCl_3$): δ 16.7/17.9 (q, $CH\underline{C}H_3$), 18.7 (q, $CH=C(\underline{C}H_3)_2$), 22.2 (q, $\underline{C}H_3CO$), 25.7 (q, $CH=C(\underline{C}H_3)_2$), 41.1/42.7 (t, $\underline{C}H_2CH=$), 50.9/56.4 (d, $\underline{C}HCH_3$), 122.6/122.8 (d, $CH_2\underline{C}H=$), 126.8/127.4 (d, CH_{Ar}), 127.6/127.7 (d, CH_{Ar}), 128.5/128.8 (d, CH_{Ar}), 133.7 (s, $CH=\underline{C}(CH_3)_2$), 141.3 (s, C_{Ar}), 167.8 (s, C=O).

(*S*)-*N*-(2-Methylallyl)-*N*-(1-phenylethyl)acetamide (11h):

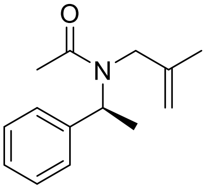

Prepared according to the general procedure, yield 3.6 g (97%) as a 3.8:1 mixture of rotamers. [R_f (hexanes/EtOAc 2:1) = 0.38]; IR (film); ν [cm^{-1}]: 3030 (w), 2976 (w), 2936 (w), 1637 (s), 1495 (m), 1448 (s), 1404 (w), 1376 (w), 1322 (w), 1299 (w), 1267 (w), 1237 (w), 1208 (m), 1183 (m), 1049 (w), 1030 (w), 1009 (w), 965 (w), 902 (m), 787 (m), 742 (m), 699 (s); MS (+ESI) m/z, (%): 240 (45, [$M+Na^+$]), 218 (100, [$M+H^+$]); HRMS (+ESI) m/z [$C_{14}H_{20}NO^+$]: calcd. 218.1467; found 218.1472; $[\alpha]_D^{20}$: –157.1 (c 1.000, $CHCl_3$); ^{1}H NMR (400 MHz, $CDCl_3$): δ 1.49 (d, *J* = 7.2 Hz, 3H, $CHC\underline{H}_3$), 1.63/1.69 (s, 3H, $CH_3C=$), 2.11/2.24 (s, 3H, CH_3CO),

3.33/3.43 (d, J = 17.0 Hz/J = 18.6 Hz, 1H, CH_2C=), 3.61/4.26 (d, J = 18.7 Hz/J = 17.0 Hz, 1H, CH_2C=), 4.67/4.81 (d, J = 2.9 Hz, 1H, C=CH_2), 4.79/4.90 (d, J = 2.9 Hz, 1H, C=CH_2), 5.14/6.11 (q, J = 7.1 Hz/J = 7.2 Hz, 1H, C$\underline{H}CH_3$), 7.23-7.41 (m, 5H, Ar$\underline{H}$); ^{13}C NMR (101 MHz, $CDCl_3$): δ 16.7/18.7 (q, CH$\underline{C}H_3$), 20.3/20.5 (q, $\underline{C}H_3C$=), 22.0/22.3 (q, $\underline{C}H_3CO$), 48.0/49.7 (t, $\underline{C}H_2C$=), 51.2/56.7 (d, $\underline{C}HCH_3$), 110.2/110.9 (t, C=$\underline{C}H_2$), 126.4/127.3 (d, CH_{Ar}), 127.5 (d, CH_{Ar}), 128.4/128.7 (d, CH_{Ar}), 133.1/133.2 (s, C_{Ar}), 141.2/141.3 (s, $\underline{C}$=CH_2), 171.9 (s, C=O).

(*S*)-*N*-(Cyclopent-1-en-1-ylmethyl)-*N*-(1-phenylethyl)acetamide (11i):

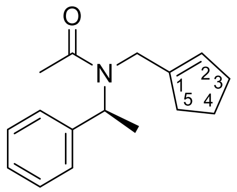

Prepared according to the general procedure, yield 3.4 g (83%) as a 3.2:1 mixture of rotamers. [R_f (hexanes/EtOAc 3:1) = 0.34]; IR (film); ν [cm^{-1}]: 3030 (w), 2936 (w), 2846 (w), 1642 (s), 1495 (w), 1451 (m), 1411 (w), 1377 (w), 1362 (w), 1327 (w), 1311 (w), 1265 (w), 1210 (w), 1182 (w), 1057 (w), 1029 (w), 995 (w), 786 (w), 737 (w), 700 (m), 642 (w); MS (+ESI) m/z, (%): 266 (50, [M+Na$^+$]), 244 (100, [M+H$^+$]); HRMS (+ESI) m/z [$C_{16}H_{22}NO^+$]: calcd. 244.1623; found 244.1622; $[\alpha]_D^{20}$: –132.5 (c 0.200, $CHCl_3$); ^{1}H NMR (400 MHz, $CDCl_3$): δ 1.48/1.60 (d, J = 7.1 Hz, 3H, CHC$\underline{H}_3$), 1.83 (quint, J = 7.8 Hz, 2H, H4), 2.05-2.09/2.15-2.20 (m, 2H, H5), 2.10/2.22 (s, 3H, CH_3CO), 2.23-2.30 (m, 2H, H3), 3.49/3.53 (d, J = 18.0 Hz/J = 16.6 Hz, 1H, CH_2C=), 3.69/4.22 (d, J = 18.0 Hz/J = 16.6 Hz, 1H, CH_2C=), 5.10/6.07 (q, J = 7.2 Hz, 1H, C$\underline{H}CH_3$), 5.27-5.30/5.40 (m/quint, J = 2.1 Hz, 1H, H2), 7.20-7.39 (m, 5H, Ar$\underline{H}$); ^{13}C NMR (101 MHz, $CDCl_3$): δ 16.9/18.8 (q, CH$\underline{C}H_3$), 22.3/22.4 (q, $\underline{C}H_3CO$), 23.5 (t, C4), 32.4 (t, C3), 33.6/33.8 (t, C5), 43.2/45.4 (t, $\underline{C}H_2C$=), 51.2/56.6 (d, $\underline{C}HCH_3$), 125.5/126.4 (d, C2), 126.7/127.4 (d, CH_{Ar}), 127.6/127.7 (d, CH_{Ar}), 128.4/128.8 (d, CH_{Ar}), 137.5/137.6 (s, C_{Ar}), 141.25/141.30 (s, C1), 171.60/171.63 (s, C=O).

***N*-Benzyl-*N*-(cyclopent-1-en-1-ylmethyl)acetamide (11j):**

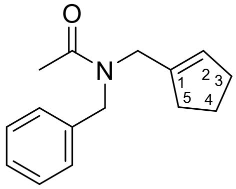

Prepared according to the general procedure, yield 3.9 g (99%) as a 1.8:1 mixture of rotamers. [R_f (hexanes/EtOAc 3:1) = 0.44]; IR (film); ν [cm^{-1}]: 3031 (w), 2928 (w), 2847 (w), 1645 (s), 1495 (w), 1469 (w), 1420 (m), 1359 (w), 1241 (m), 1170 (w), 1066 (w), 1029 (w), 986 (m), 944 (w), 821 (w), 774 (w), 733 (m), 700 (m), 600 (w); MS (+ESI) m/z, (%): 481 (15, [$2M+Na^+$]), 459 (15, [$2M+H^+$]), 252 (30, [$M+Na^+$]), 230 (100, [$M+H^+$]); HRMS (+ESI) m/z [$C_{15}H_{20}NO^+$]: calcd. 230.1539; found 230.1539; ^{1}H NMR (400 MHz, $CDCl_3$): δ 1.86-1.98 (m, 2H, H4), 2.16/2.17 (s, 3H, CH_3CO), 2.19-2.27 (m, 2H, H3), 2.30-2.40 (m, 2H, H5), 3.82/4.09 (s, 2H, NCH_2C=), 4.49/4.59 (s, 2H, C$\underline{H}_2$Ph), 5.46/5.53 (quint, *J* = 1.8 Hz/*J* = 2.1 Hz, 1H, H2), 7.16-7.39 (m, 5H, Ar$\underline{H}$); ^{13}C NMR (101 MHz, $CDCl_3$): δ 21.6/21.7 (q, $\underline{C}H_3CO$), 23.5 (t, C4), 32.3/32.4 (t, C5), 33.3/33.5 (t, C3), 45.2/48.4 (t, N$\underline{C}H_2$C=), 48.2/50.9 (t, $\underline{C}H_2$Ph), 126.4/127.4 (d, CH_{Ar}), 127.5/127.6 (d, C2), 128.3 (d, CH_{Ar}), 128.6/129.0 (d, CH_{Ar}), 136.9/137.8 (s, C_{Ar}), 139.5/140.0 (s, C1), 171.0/171.1 (s, C=O).

***N*-Benzyl-*N*-(cyclohex-1-en-1-ylmethyl)acetamide (11k):**

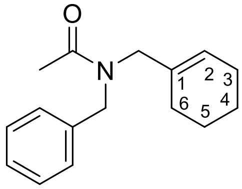

Prepared according to the general procedure, yield 4.3 g (99%) as a 1.7:1 mixture of rotamers. [R_f (hexanes/EtOAc 3:1) = 0.32]; IR (film); ν [cm^{-1}]: 2925 (m), 2857 (w), 2836 (w), 1644 (s), 1495 (w), 1468 (w), 1421 (m), 1359 (w), 1237 (w), 1177 (m), 1067 (w), 1030 (w), 985 (m), 921 (w), 802 (w), 732 (m), 699 (m), 628 (w), 604 (w); MS (+ESI) m/z, (%): 509 (15, [$2M+Na^+$]), 266 (100, [$M+Na^+$]), 244 (25, [$M+H^+$]); HRMS (+ESI) m/z [$C_{16}H_{21}NONa^+$]: calcd. 266.1515; found 266.1511; ^{1}H NMR (400 MHz, $CDCl_3$): δ 1.52-1.68 (m, 4H, H4, H5), 1.81-1.86/1.87-1.91 (m, 2H, H6), 1.97-2.06 (m, 2H, H3), 2.15 (s, 3H, CH_3CO), 3.66/3.93 (s, 2H, NCH_2C=), 4.45/4.55 (s, 2H, C$\underline{H}_2$Ph), 5.44/5.52 (sept, *J* = 1.5 Hz/*J* = 1.7 Hz, 1H, H2), 7.14-7.39 (m, 5H, Ar$\underline{H}$); ^{13}C NMR (101 MHz, $CDCl_3$): δ 21.7/21.9 (q, $\underline{C}H_3CO$), 22.5 (t, C4), 22.6/22.7 (t, C5),

25.1/25.2 (t, C3), 26.39/26.43 (t, C6), 48.0/50.5 (t, CH_2Ph), 50.7/53.6 (t, NCH_2C=), 123.8/124.6 (d, C2), 126.4/127.6 (d, CH_{Ar}), 127.3/128.4 (d, CH_{Ar}), 128.6/129.0 (d, CH_{Ar}), 132.3/133.2 (s, C1), 137.0/137.9 (s, C_{Ar}), 171.2/171.3 (s, C=O).

***N*-Benzyl-*N*-(cyclopent-2-en-1-yl)acetamide (11l):**

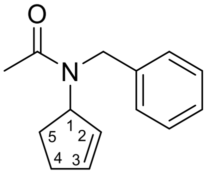

Prepared according to the general procedure, yield 3.6 g (98%) as a 1.3:1 mixture of rotamers.
[R_f (hexanes/EtOAc 2:1) = 0.39]; IR (film); ν [cm^{-1}]: 3063 (w), 3031 (w), 2939 (w), 2850 (w), 1644 (s), 1495 (w), 1413 (m), 1363 (w), 1328 (w), 1297 (w), 1264 (w), 1206 (w), 1178 (w), 1028 (w), 997 (w), 976 (w), 729 (m), 700 (m); MS (+ESI) m/z, (%): 238 (100, [$M+Na^+$]), 216 (30, [$M+H^+$]); HRMS (+ESI) m/z [$C_{14}H_{17}NONa^+$]: calcd. 238.1202; found 238.1199; ^1H NMR (400 MHz, $CDCl_3$): δ 1.49-1.58/1.59-1.68 (m, 1H, H5), 2.00/2.27 (s, 3H, CH_3), 2.21-2.39 (m, 3H, H5, H4), 4.38 (d, *J* = 15.5 Hz, 1H, CH_2Ph)/4.40 (s, 2H, CH_2Ph)/4.52 (d, *J* = 15.5 Hz, 1H, CH_2Ph), 5.01-5.07/5.79-5.85 (m, 1H, H1), 5.52 (dd, *J* = 5.7, 2.2 Hz, 1H, H2), 5.92 (ddd, *J* = 5.6, 4.6, 2.3 Hz, 1H, H3), 7.14-7.22 (m, 2H, ArH), 7.24-7.29 (m, 2H, ArH), 7.31-7.37 (m, 1H, ArH); ^{13}C NMR (101 MHz, $CDCl_3$): δ 22.4/22.6 (q, CH_3), 28.6/29.1 (t, C5), 31.5 (t, C4), 45.4/47.7 (t, CH_2Ph), 60.8/65.2 (d, C1), 125.8/126.7 (d, CH_{Ar}), 127.2/127.3 (d, CH_{Ar}), 128.4/128.9 (d, CH_{Ar}), 130.2/130.4 (d, C2), 135.2/135.6 (d, C3), 138.8/139.6 (s, C_{Ar}), 170.9/171.9 (s, C=O).

(*R*)-*N*-Benzyl-*N*-(cyclohex-2-en-1-yl)acetamide (11m):

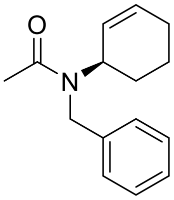

Prepared according to the general procedure from enantiomerically enriched amine **S9** (85% ee, see p. S2), yield 3.9 g (99%, 85% ee) as a 1.9:1 mixture of rotamers.
HRMS (+CI) m/z [$C_{15}H_{20}NO^+$]: calcd. 230.1545; found 230.1543; $[\alpha]_D^{20}$: +49.6 (c 0.498, $CHCl_3$).
The analytical data are in agreement with those in the literature.[8]

General procedure for the α-silylation of amides 11:

n-Butyllithium (1.6 M in hexane, 14.9 mL, 23.8 mmol) was added dropwise by syringe to a stirred solution of dry diisopropylamine (3.6 mL, 23.8 mmol) in anhydrous THF (30 mL) at −78 °C. After 30 min, a solution of the amide **11** (21.6 mmol) in anhydrous THF (5 mL) was added dropwise and the mixture was stirred for 30 min. Then, TMSCl (3.3 mL, 22.7 mmol) was rapidly added at −78 °C and the mixture was stirred at this temperature for 1 h. The reaction was quenched by saturated NH_4Cl solution after diluting with water (30 mL) and diethyl ether (50 mL), the organic layer was separated and the aqueous was extracted with diethyl ether (3 × 10 mL). The combined organic layers were dried over $MgSO_4$ and filtered. The filtrate was evaporated and the crude product was purified by flash chromatography (gradient, hexanes/EtOAc 20:1 to 5:1) to give α-trimethylsilylamides **8**.

***N*,*N*-Diallyl-2-(trimethylsilyl)acetamide (8a):**

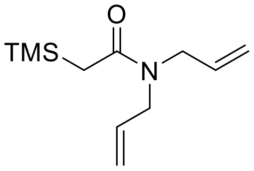

Prepared according to the general procedure, yield 3.4 g (76%).

[R*f* (hexanes/EtOAc 1:1) = 0.57]; IR (film); ν [cm^{-1}]: 3081 (w), 2955 (w), 1627 (s), 1443 (m), 1415 (m), 1395 (m), 1282 (w), 1247 (s), 1195 (w), 1134 (w), 1104 (w), 1025 (w), 993 (w), 920 (m), 851 (s), 700 (w), 611 (w); MS (+ESI) m/z, (%): 234 (60, [$M+Na^+$]), 212 (100, [$M+H^+$]); HRMS (+ESI) m/z [$C_{11}H_{22}NOSi^+$]: calcd. 212.1392; found 212.1396; ^{1}H NMR (400 MHz, $CDCl_3$): δ 0.10 (s, 9H, $Si(CH_3)_3$), 1.94 (s, 2H, CH_2CO), 3.80 (d, *J* = 5.4 Hz, 2H, C<u>H</u>$_2$CH=), 3.95 (d, *J* = 6.0 Hz, 2H, C<u>H</u>$_2$CH=), 5.03-5.22 (m, 4H, CH=C<u>H</u>$_2$), 5.67-5.80 (m, 2H, C<u>H</u>=CH$_2$); ^{13}C NMR (101 MHz, $CDCl_3$): δ –0.8 (q, $Si(CH_3)_3$), 25.4 (t, <u>C</u>H$_2$CO), 47.6 (t, <u>C</u>H$_2$CH=), 50.1 (t, <u>C</u>H$_2$CH=), 116.7 (t, CH=<u>C</u>H$_2$), 117.1 (t, CH=<u>C</u>H$_2$), 133.1 (d, <u>C</u>H=CH$_2$), 133.9 (d, <u>C</u>H=CH$_2$), 172.6 (s, C=O).

***N*-Allyl-*N*-methyl-2-(trimethylsilyl)acetamide (8b):**

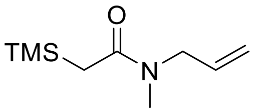

Prepared according to the general procedure, yield 4.1 g (70%) as a 1.3:1 mixture of rotamers. [R_f (hexanes/EtOAc 3:1) = 0.23]; IR (film); ν [cm^{-1}]: 2955 (w), 1624 (s), 1419 (w), 1383 (m), 1278 (w), 1247 (m), 1136 (w), 1072 (m), 991 (w), 920 (w), 848 (s), 722 (w), 700 (w), 609 (w); MS (+ESI) m/z, (%): 208 (55, [$M+Na^+$]), 186 (100, [$M+H^+$]); HRMS (+ESI) m/z [$C_9H_{20}NOSi^+$]: calcd. 186.1314; found 186.1316; ^{1}H NMR (400 MHz, $CDCl_3$): δ 0.11/0.12 (s, 9H, $Si(CH_3)_3$), 1.93/1.99 (s, 2H, CH_2CO), 2.89/2.90 (s, 3H, CH_3), 3.84/3.98 (d, *J* = 5.0 Hz/*J* = 6.1 Hz, 2H, $C\underline{H}_2CH=$), 5.07-5.23 (m, 2H, $CH=C\underline{H}_2$), 5.65-5.83 (m, 1H, $C\underline{H}=CH_2$); ^{13}C NMR (101 MHz, $CDCl_3$): δ –0.8 (q, $Si(CH_3)_3$), 25.2/25.7 (t, $\underline{C}H_2CO$), 33.5/36.0 (q, CH_3), 50.0/53.4 (t, $\underline{C}H_2CH=$), 116.8/117.1 (t, $CH=\underline{C}H_2$), 132.9/133.8 (d, $\underline{C}H=CH_2$), 172.4/172.9 (s, C=O).

***N*-Benzyl-*N*-(2-methylallyl)-2-(trimethylsilyl)acetamide (8c):**

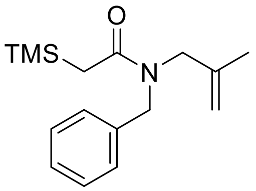

Prepared according to the general procedure, yield 4.2 g (75%) as a 1.4:1 mixture of rotamers. [R_f (hexanes/EtOAc 3:1) = 0.53]; IR (film); ν [cm^{-1}]: 3066 (w), 3030 (w), 2954 (w), 1628 (s), 1495 (w), 1440 (m), 1395 (m), 1248 (m), 1204 (w), 1139 (w), 1119 (w), 1077 (w), 1051 (w), 1030 (w), 942 (w), 897 (w), 852 (s), 722 (w), 701 (m), 604 (w); MS (+ESI) m/z, (%): 573 (50, [$2M+Na^+$]), 298 (100, [$M+Na^+$]), 276 (30, [$M+H^+$]); HRMS (+ESI) m/z [$C_{16}H_{25}NOSiNa^+$]: calcd. 298.1598; found 298.1595; ^{1}H NMR (400 MHz, $CDCl_3$): δ 0.13/0.15 (s, 9H, $Si(CH_3)_3$), 1.70 (s, 3H, CH_3), 2.00/2.06 (s, 2H, CH_2CO), 3.65/3.98 (s, 2H, $NCH_2C=$), 4.45/4.57 (s, 2H, $C\underline{H}_2Ph$), 4.74/4.83 (d, *J* = 2.9 Hz, 1H, $C=CH_2$), 4.89/4.96 (d, *J* = 2.9 Hz, 1H, $C=CH_2$), 7.16-7.39 (m, 5H, $Ar\underline{H}$); ^{13}C NMR (101 MHz, $CDCl_3$): δ –0.62/–0.59 (q, $Si(CH_3)_3$), 20.28/20.34 (q, CH_3), 25.4/25.8 (t, $\underline{C}H_2CO$), 48.0/50.6 (t, $\underline{C}H_2Ph$), 50.2/53.1 (t, $N\underline{C}H_2C=$), 111.8/112.4 (t, $C=\underline{C}H_2$), 126.5/127.6 (d, CH_{Ar}), 127.3/128.5 (d, CH_{Ar}), 128.6/129.0 (d, CH_{Ar}), 137.1/138.3 (s, C_{Ar}), 140.0/141.2 (s, $\underline{C}=CH_2$), 173.1/173.3 (s, C=O).

_N_-Benzyl-_N_-(3-methylbut-2-en-1-yl)-2-(trimethylsilyl)acetamide (8d):

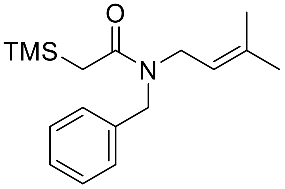

Prepared according to the general procedure, yield 4.5 g (72%) as a 1.4:1 mixture of rotamers. [R_f (hexanes/EtOAc 3:1) = 0.58]; IR (film); ν [cm^{-1}]: 2955 (w), 2867 (w), 1623 (s), 1495 (w), 1440 (m), 1397 (m), 1361 (w), 1245 (m), 1138 (w), 1112 (w), 1076 (w), 1033 (w), 943 (w), 846 (s), 772 (w), 733 (w), 699 (m), 625 (w), 603 (w); MS (+ESI) m/z, (%): 601 (20, [2M+Na$^+$]), 312 (100, [M+Na$^+$]), 290 (80, [M+H$^+$]); HRMS (+ESI) m/z [$C_{17}H_{27}NOSiNa^+$]: calcd. 312.1754; found 312.1752; ^{1}H NMR (400 MHz, $CDCl_3$): δ 0.15/0.16 (s, 9H, Si(CH_3)$_3$), 1.55/1.59 (s, 3H, CH_3), 1.72/1.74 (s, 3H, CH_3), 2.03/2.06 (s, 2H, CH_2CO), 3.75/4.00 (d, *J* = 6.8 Hz/*J* = 7.0 Hz, 2H, C$\underline{H}_2$CH=), 4.44/4.58 (s, 2H, C$\underline{H}_2$Ph), 5.09-5.13/5.14-5.18 (m, 1H, C$\underline{H}$=C(CH_3)$_2$), 7.18-7.22 (m, 2H, Ar$\underline{H}$), 7.23-7.39 (m, 3H, Ar$\underline{H}$); ^{13}C NMR (101 MHz, $CDCl_3$): δ –0.73/–0.65 (q, Si(CH_3)$_3$), 17.9/18.0 (q, CH_3), 25.5/25.7 (t, $\underline{C}H_2$CO), 25.81/25.83 (q, CH_3), 42.6/45.9 (t, $\underline{C}H_2$CH=), 47.9/51.1 (t, $\underline{C}H_2$Ph), 120.36/120.40 (d, $\underline{C}$H=C(CH_3)$_2$), 126.6/127.5 (d, CH_{Ar}), 127.2/128.4 (d, CH_{Ar}), 128.5/128.9 (d, CH_{Ar}), 135.6/136.1 (s, CH=$\underline{C}$(CH_3)$_2$), 137.5/138.5 (s, C_{Ar}), 172.7/172.8 (s, C=O).

(_S_)-_N_-Allyl-_N_-(1-phenylethyl)-2-(trimethylsilyl)acetamide (8e):

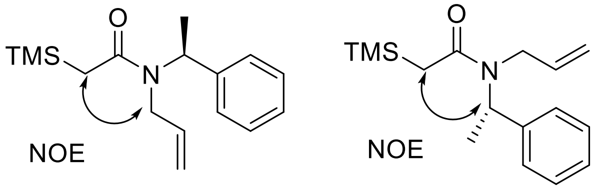

Major rotamer   Minor rotamer

Prepared according to the general procedure, yield 5.3 g (89%) as a 3.6:1 mixture of rotamers. [R_f (hexanes/EtOAc 3:1) = 0.55]; IR (film); ν [cm^{-1}]: 3064 (w), 3030 (w), 2955 (w), 1623 (s), 1495 (w), 1431 (m), 1390 (m), 1315 (w), 1248 (m), 1209 (w), 1183 (w), 1096 (w), 985 (w), 918 (w), 854 (s), 787 (w), 744 (w), 700 (m), 622 (w); MS (+ESI) m/z, (%): 573 (10, [2M+Na$^+$]), 298 (100, [M+Na$^+$]); HRMS (+ESI) m/z [$C_{16}H_{25}NOSiNa^+$]: calcd. 298.1598; found 298.1593; $[\alpha]_D^{20}$: –124.1 (c 0.998, $CHCl_3$); ^{1}H NMR (400 MHz, $CDCl_3$): δ 0.13/0.15 (s, 9H, Si(CH_3)$_3$), 1.47/1.62 (d, *J* = 7.2 Hz/*J* = 7.1 Hz, 3H, CH_3), 1.96/2.15 (d, *J* = 13.0 Hz/*J* = 12.8 Hz, 1H, CH_2CO),

2.01/2.15 (d, *J* = 13.0 Hz/*J* = 12.8 Hz, 1H, CH_2CO), 3.40/3.55 (dd, *J* = 15.6, 6.5 Hz/*J* = 17.9, 5.3 Hz, 1H, $C\underline{H}_2CH=$), 3.66/4.25 (dd, *J* = 17.9, 5.3 Hz/*J* = 15.6, 4.7 Hz, 1H, $C\underline{H}_2CH=$), 4.99-5.07 (m, 2H, $CH=C\underline{H}_2$), 5.10/6.14 (q, *J* = 7.2 Hz, 1H, $C\underline{H}CH_3$), 5.55/5.76-5.89 (ddt, *J* = 17.2, 10.5, 5.3 Hz/m, 1H, $C\underline{H}=CH_2$), 7.21-7.40 (m, 5H. Ar$\underline{H}$); ^{13}C NMR (101 MHz, $CDCl_3$): δ –0.6/–0.5 (q, $Si(CH_3)_3$), 16.9/19.6 (q, CH_3), 25.7/26.1 (t, $\underline{C}H_2CO$), 45.6/46.7 (t, $\underline{C}H_2CH=$), 50.8/56.7 (d, $\underline{C}HCH_3$), 115.7/116.4 (t, $CH=\underline{C}H_2$), 126.6/127.9 (d, CH_{Ar}), 127.4/127.5 (d, CH_{Ar}), 128.4/128.8 (d, CH_{Ar}), 135.8/135.9 (d, $\underline{C}H=CH_2$), 141.5 (s, C_{Ar}), 173.4 (s, C=O).

(*S*)-*N*-Allyl-*N*-(1-(naphthalen-2-yl)ethyl)-2-(trimethylsilyl)acetamide (8f):

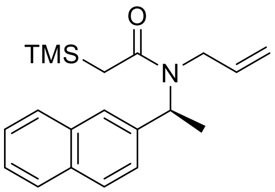

Prepared according to the general procedure, yield 6.6 g (95%) as a 4:1 mixture of rotamers.

[R_f (hexanes/EtOAc 2:1) = 0.68]; IR (film); ν [cm^{-1}]: 3056 (w), 2954 (w), 2895 (w), 1621 (s), 1431 (m), 1389 (m), 1315 (w), 1248 (m), 1190 (w), 1125 (w), 986 (w), 921 (w), 855 (s), 822 (m), 749 (m), 724 (w), 702 (w); MS (+ESI) m/z, (%): 673 (10, [2M+Na$^+$]), 348 (40, [M+Na$^+$]), 326 (100, [M+H$^+$]); HRMS (+ESI) m/z [$C_{20}H_{28}NOSi^+$]: calcd. 326.1935; found 326.1933; $[\alpha]_D^{20}$: –184.7 (c 1.000, $CHCl_3$); ^{1}H NMR (400 MHz, $CDCl_3$): δ 0.16/0.18 (s, 9H, $Si(CH_3)_3$), 1.59/1.73 (d, *J* = 7.1 Hz/*J* = 7.0 Hz, 3H, CH_3), 1.85/2.00 (d, *J* = 13.0 Hz/*J* = 13.2 Hz, 1H, CH_2CO), 2.06/2.22 (d, *J* = 13.0 Hz/*J* = 13.2 Hz, 1H, CH_2CO), 3.44/3.56 (dd, *J* = 15.5, 6.4 Hz/*J* = 17.7, 5.4 Hz, 1H, $C\underline{H}_2CH=$), 3.68/4.32 (dd, *J* = 17.7, 5.1 Hz/*J* = 15.5, 4.6 Hz, 1H, $C\underline{H}_2CH=$), 4.95-5.11 (m, 2H, $CH=C\underline{H}_2$), 5.25/6.31 (q, *J* = 7.1 Hz, 1H, $C\underline{H}CH_3$), 5.50-5.63/5.79-5.93 (m, 1H, $C\underline{H}=CH_2$), 7.34-7.51 (m, 3H, Ar$\underline{H}$), 7.66/7.74 (s, 1H, Ar$\underline{H}$), 7.76-7.85 (m, 3H, Ar$\underline{H}$); ^{13}C NMR (101 MHz, $CDCl_3$): δ –0.6/–0.5 (q, $Si(CH_3)_3$), 16.9/19.6 (q, CH_3), 25.8/26.2 (t, $\underline{C}H_2CO$), 45.6/46.7 (t, $\underline{C}H_2CH=$), 50.8/56.8 (d, $\underline{C}HCH_3$), 115.8/116.5 (t, $CH=\underline{C}H_2$), 125.0/125.8 (d, CH_{Ar}), 126.0 (d, CH_{Ar}), 126.2/126.3 (d, CH_{Ar}), 126.6/126.9 (d, CH_{Ar}), 127.7 (d, CH_{Ar}), 128.0/128.1 (d, CH_{Ar}), 128.2/128.6 (d, CH_{Ar}), 132.7/132.8 (s, C_{Ar}), 133.3/133.4 (s, C_{Ar}), 135.8/135.9 (d, $\underline{C}H=CH_2$), 138.9/139.1 (s, C_{Ar}), 173.0/173.5 (s, C=O).

(*S*)-*N*-(3-Methylbut-2-en-1-yl)-*N*-(1-phenylethyl)-2-(trimethylsilyl)acetamide (8g):

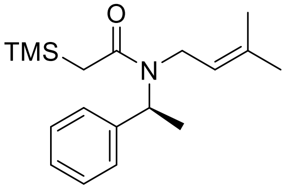

Prepared according to the general procedure, yield 5.7 g (88%) as a 3.9:1 mixture of rotamers. [R_f (hexanes/EtOAc 3:1) = 0.67]; IR (film); ν [cm^{-1}]: 2954 (w), 2932 (w), 1623 (s), 1494 (w), 1449 (m), 1431 (w), 1393 (m), 1377 (m), 1333 (w), 1305 (w), 1248 (m), 1211 (w), 1172 (w), 1111 (w), 1095 (w), 1068 (w), 1029 (w), 983 (w), 855 (s), 785 (w), 720 (w), 700 (m), 618 (w); MS (+ESI) m/z, (%): 629 (10, [$2M+Na^+$]), 326 (100, [$M+Na^+$]), 304 (95, [$M+H^+$]); HRMS (+ESI) m/z [$C_{18}H_{29}NOSiNa^+$]: calcd. 326.1911; found 326.1908; $[\alpha]_D^{20}$: –96.7 (c 1.000, $CHCl_3$); ^{1}H NMR (400 MHz, $CDCl_3$): δ 0.13/0.15 (s, 9H, $Si(CH_3)_3$), 1.47/1.60 (d, *J* = 7.2 Hz/*J* = 7.1 Hz, 3H, $CHC\underline{H}_3$), 1.50 (s, 3H, $CH=C(C\underline{H}_3)_2$), 1.63 (s, 3H, $CH=C(C\underline{H}_3)_2$), 1.92/1.99 (d, *J* = 12.8 Hz/*J* = 13.3 Hz, 1H, CH_2CO), 1.97/2.13 (d, *J* = 12.8 Hz/*J* = 13.3 Hz, 1H, CH_2CO), 3.42/3.48 (dd, *J* = 17.5, 6.1 Hz/*J* = 15.8, 4.6 Hz, 1H, $C\underline{H}_2CH=$), 3.61/4.17 (dd, *J* = 17.5, 6.2 Hz/*J* = 15.8, 4.5 Hz, 1H, $C\underline{H}_2CH=$), 4.85-4.91/5.10-5.14 (m, 1H, $C\underline{H}=C(CH_3)_2$), 5.06/6.12 (q, *J* = 7.1 Hz/*J* = 7.0 Hz, 1H, $C\underline{H}CH_3$), 7.21-7.28 (m, 3H, Ar$\underline{H}$), 7.29-7.37 (m, 2H, Ar$\underline{H}$); ^{13}C NMR (101 MHz, $CDCl_3$): δ –0.6 (q, $Si(CH_3)_3$), 16.8/19.4 (q, $CH\underline{C}H_3$), 17.9 (q, $CH=C(\underline{C}H_3)_2$), 25.7 (q, $CH=C(\underline{C}H_3)_2$), 25.8/26.8 (t, $\underline{C}H_2CO$), 41.2/42.5 (t, $\underline{C}H_2CH=$), 50.6/56.4 (d, $\underline{C}HCH_3$), 123.3/123.4 (d, $\underline{C}H=C(CH_3)_3$), 126.6/127.8 (d, CH_{Ar}), 127.3/127.4 (d, CH_{Ar}), 128.4/128.7 (d, CH_{Ar}), 133.0/133.1 (s, $CH=\underline{C}(CH_3)_2$), 141.4/141.9 (s, C_{Ar}), 172.6/173.1 (s, C=O).

(*S*)-*N*-(2-Methylallyl)-*N*-(1-phenylethyl)-2-(trimethylsilyl)acetamide (8h):

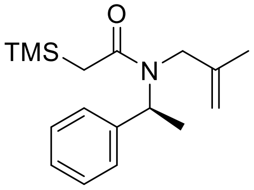

Prepared according to the general procedure, yield 5.8 g (93%) as a 3.9:1 mixture of rotamers. [R_f (hexanes/EtOAc 3:1) = 0.57]; IR (film); ν [cm^{-1}]: 3087 (w), 3031 (w), 2954 (w), 1625 (s), 1495 (w), 1449 (m), 1431 (m), 1388 (m), 1322 (w), 1248 (m), 1209 (w), 1186 (w), 1126 (w), 1097 (w), 1070 (w), 1047 (w), 1030 (w), 980 (w), 855 (s), 787 (w), 746 (w), 701 (m), 619 (w); MS (+ESI) m/z, (%): 601 (40, [$2M+Na^+$]), 312 (100, [$M+Na^+$]), 290 (25, [$M+H^+$]); HRMS

(+ESI) m/z [$C_{17}H_{27}NOSiNa^+$]: calcd. 312.1754; found 312.1752; $[\alpha]_D^{20}$: –106.4 (c 1.004, $CHCl_3$); ^{1}H NMR (400 MHz, $CDCl_3$): δ 0.11/0.16 (s, 9H, $Si(CH_3)_3$), 1.44 (d, *J* = 7.1 Hz, 3H, CHC<u>H</u>3), 1.59/1.69 (s, 3H, CH_3C=), 1.90/1.98 (d, *J* = 13.2 Hz/*J* = 13.3 Hz, 1H, CH_2CO), 1.96/2.17 (d, *J* = 13.2 Hz/*J* = 13.3 Hz, 1H, CH_2CO), 3.25/3.37 (d, *J* = 16.8 Hz/*J* = 18.7 Hz, 1H, CH_2C=), 3.53/4.34 (d, *J* = 18.7 Hz/*J* = 16.8 Hz, 1H, CH_2C=), 4.70/4.79 (d, *J* = 1.9 Hz, 1H, C=CH_2), 4.86 (d, *J* = 1.9 Hz, 1H, C=CH_2), 5.12/6.12 (q, *J* = 6.9 Hz/*J* = 7.1 Hz, 1H, C<u>H</u>CH_3), 7.19-7.41 (m, 5H, Ar<u>H</u>); ^{13}C NMR (101 MHz, $CDCl_3$): δ –0.6/–0.5 (q, $Si(CH_3)_3$), 16.9/19.3 (q, CH<u>C</u>H_3), 20.5/20.8 (q, <u>C</u>H_3C=), 26.1/26.4 (t, <u>C</u>H_2CO), 48.4/49.6 (t, <u>C</u>H_2C=), 51.1/56.8 (d, <u>C</u>HCH_3), 110.0/111.3 (t, C=<u>C</u>H_2), 126.5/127.9 (d, CH_{Ar}), 127.4/127.5 (d, CH_{Ar}), 128.4/128.8 (d, CH_{Ar}), 133.7 (s, C_{Ar}), 141.6/141.8 (s, <u>C</u>=CH_2), 173.66/173.74 (s, C=O).

(*S*)-*N*-(Cyclopent-1-en-1-ylmethyl)-*N*-(1-phenylethyl)-2-(trimethylsilyl)acetamide (8i):

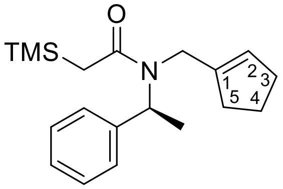

Prepared according to the general procedure, yield 5.7 g (84%) as a 4.9:1 mixture of rotamers. [R_f (hexanes/EtOAc 3:1) = 0.54]; IR (film); ν [cm^{-1}]: 2952 (w), 2896 (w), 2846 (w), 1624 (s), 1495 (w), 1459 (m), 1430 (m), 1392 (w), 1375 (w), 1327 (w), 1310 (m), 1248 (w), 1210 (w), 1184 (w), 1140 (w), 1121 (w), 1096 (w), 1069 (w), 1029 (w), 985 (w), 855 (s), 787 (w), 700 (m), 622 (w); MS (+ESI) m/z, (%): 653 (10, [2M+Na^+]), 338 (100, [M+Na^+]), 316 (15, [M+H^+]); HRMS (+ESI) m/z [$C_{19}H_{29}NOSiNa^+$]: calcd. 338.1911; found 338.1909; $[\alpha]_D^{20}$: –82.3 (c 1.000, $CHCl_3$); ^{1}H NMR (400 MHz, $CDCl_3$): δ 0.11/0.15 (s, 9H, $Si(CH_3)_3$), 1.45/1.60 (d, *J* = 7.1 Hz, 3H, CHC<u>H</u>3), 1.76-1.89 (m, 2H, H4), 1.94/1.98 (d, *J* = 13.1 Hz, 1H, CH_2CO), 2.01/2.14 (d, *J* = 13.1 Hz, 1H, CH_2CO), 2.03-2.10 (m, 2H, H3), 2.18-2.30 (m, 2H, H5), 3.44/3.50 (d, *J* = 15.4 Hz/*J* = 18.0 Hz, 1H, CH_2C=), 3.63/4.35 (d, *J* = 18.0 Hz/*J* = 15.4 Hz, 1H, CH_2C=), 5.09/6.10 (q, *J* = 7.2 Hz, 1H, C<u>H</u>CH_3), 5.29-5.33/5.38 (m/quint, *J* = 2.3 Hz, 1H, H2), 7.20-7.38 (m, 5H, Ar<u>H</u>); ^{13}C NMR (101 MHz, $CDCl_3$): δ –0.58/–0.56 (q, $Si(CH_3)_3$), 16.9/19.3 (q, CH<u>C</u>H_3), 23.47/23.50 (t, C4), 26.0/26.3 (t, <u>C</u>H_2CO), 32.36/32.42 (t, C5), 33.6/34.0 (t, C3), 43.4/45.1 (t, <u>C</u>H_2C=), 51.0/56.7 (d, <u>C</u>HCH_3), 125.1/126.5 (d, C2), 126.6/127.3 (d, CH_{Ar}), 127.4/127.9 (d, CH_{Ar}), 128.3/128.7 (d, CH_{Ar}), 141.5/141.7 (s, C1, C_{Ar}), 173.0/173.4 (s, C=O).

***N*-Benzyl-*N*-(cyclopent-1-en-1-ylmethyl)-2-(trimethylsilyl)acetamide (8j):**

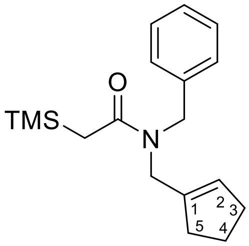

Prepared according to the general procedure, yield 4.5 g (82%) as a 1.5:1 mixture of rotamers. [R_f (hexanes/EtOAc 3:1) = 0.61]; IR (film); ν [cm^{-1}]: 3030 (w), 2952 (w), 2897 (w), 2847 (w), 1625 (s), 1495 (w), 1441 (m), 1397 (m), 1357 (w), 1247 (m), 1138 (w), 1113 (w), 1076 (w), 1044 (w), 1028 (w), 946 (w), 849 (s), 725 (w), 700 (m), 616 (w); MS (+ESI) m/z, (%): 625 (10, [$2M+Na^+$]), 324 (100, [$M+Na^+$]), 302 (25, [$M+H^+$]); HRMS (+ESI) m/z [$C_{18}H_{27}NOSiNa^+$]: calcd. 324.1754; found 324.1758; ^{1}H NMR (400 MHz, $CDCl_3$): δ 0.13/0.14 (s, 9H, $Si(CH_3)_3$), 1.82-1.98 (m, 2H, H4), 2.02/2.03 (s, 2H, CH_2CO), 2.17-2.26 (m, 2H, H3), 2.29-2.39 (m, 2H, H5), 3.76/4.08 (s, 2H, NCH_2C=), 4.45/4.58 (s, 2H, C$\underline{H}_2$Ph), 5.45/5.52 (quint, *J* = 1.7 Hz/*J* = 1.9 Hz, 1H, H2), 7.15-7.25 (m, 2H, Ar$\underline{H}$), 7.26-7.38 (m, 3H, Ar$\underline{H}$); ^{13}C NMR (101 MHz, $CDCl_3$): δ –0.64/–0.63 (q, $Si(CH_3)_3$), 23.5/23.7 (t, C4), 25.5/25.8 (t, $\underline{C}H_2CO$), 32.36/32.43 (t, C5), 33.4/33.7 (t, C3), 44.9/48.4 (t, N$\underline{C}H_2$C=), 48.0/50.9 (t, $\underline{C}H_2$Ph), 126.5/127.6 (d, CH_{Ar}), 127.3/128.5 (d, CH_{Ar}), 127.39/127.43 (d, C2), 128.5/128.9 (d, CH_{Ar}), 137.2/138.5 (s, C_{Ar}), 139.8/140.5 (s, C1), 172.9/173.0 (s, C=O).

***N*-Benzyl-*N*-(cyclohex-1-en-1-ylmethyl)-2-(trimethylsilyl)acetamide (8k):**

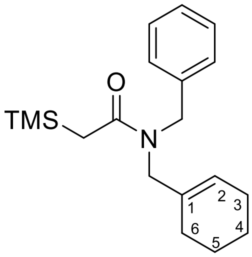

Prepared according to the general procedure, yield 5.7 g (85%) as a 1.5:1 mixture of rotamers. [R_f (hexanes/EtOAc 3:1) = 0.63]; IR (film); ν [cm^{-1}]: 3029 (w), 2927 (w), 2858 (w), 2836 (w), 1625 (s), 1495 (w), 1439 (m), 1398 (m), 1374 (w), 1359 (w), 1247 (m), 1137 (w), 1116 (w), 1076 (w), 1028 (w), 852 (w), 728 (w), 700 (m), 624 (w), 603 (w); MS (+ESI) m/z, (%): 653 (10, [$2M+Na^+$]), 338 (15, [$M+Na^+$]), 316 (100, [$M+H^+$]); HRMS (+ESI) m/z [$C_{19}H_{30}NOSi^+$]: calcd. 316.2091; found 316.2089; ^{1}H NMR (400 MHz, $CDCl_3$): δ 0.12/0.14 (s, 9H, $Si(CH_3)_3$), 1.53-

1.66 (m, 4H, H4, H5), 1.81-1.86/1.87-1.92 (m, 2H, H3), 1.98-2.06 (m, 2H, H6), 2.02/2.03 (s, 2H, CH_2CO), 3.61/3.93 (s, 2H, $NCH_2C=$), 4.42/4.55 (s, 2H, C<u>H</u>$_2$Ph), 5.44/5.52 (tt, J = 3.7, 1.7 Hz/J = 3.8, 1.7 Hz, 1H, H2), 7.15-7.25 (m, 2H, Ar<u>H</u>), 7.26-7.37 (m, 3H, Ar<u>H</u>); ^{13}C NMR (101 MHz, $CDCl_3$): δ –0.61/–0.60 (q, $Si(CH_3)_3$), 22.5 (t, C5), 22.6/22.7 (t, C4), 25.1/25.2 (t, C6), 25.6/25.9 (t, <u>C</u>H_2CO), 26.5/26.6 (t, C3), 47.8/50.4 (t, N<u>C</u>H_2C=), 50.5/53.6 (t, <u>C</u>H_2Ph), 123.8/124.7 (d, C2), 126.5/127.5 (d, CH_{Ar}), 127.2/128.5 (d, CH_{Ar}), 128.6/128.9 (d, CH_{Ar}), 132.6/133.7 (s, C1), 137.3/138.5 (s, C_{Ar}), 173.1/173.2 (s, C=O).

***N*-Benzyl-*N*-(cyclopent-2-en-1-yl)-2-(trimethylsilyl)acetamide (8l):**

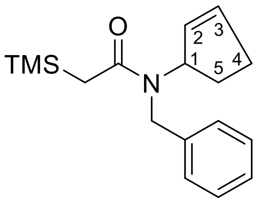

Prepared according to the general procedure, yield 5.6 g (91%) as a 1.5:1 mixture of rotamers. [R_f (hexanes/EtOAc 3:1) = 0.47]; IR (film); ν [cm^{-1}]: 3062 (w), 3030 (w), 2953 (w), 2853 (w), 1625 (s), 1496 (w), 1454 (m), 1435 (m), 1394 (m), 1365 (w), 1350 (w), 1326 (w), 1295 (w), 1249 (m), 1208 (w), 1179 (w), 1138 (w), 1076 (w), 1028 (w), 852 (s), 726 (m), 699 (m), 618 (w); MS (+ESI) m/z, (%): 310 (40, [M+Na$^+$]), 288 (100, [M+H$^+$]); HRMS (+ESI) m/z [$C_{17}H_{26}NOSi^+$]: calcd. 288.1778; found 288.1776; ^{1}H NMR (400 MHz, $CDCl_3$): δ 0.10/0.17 (s, 9H, $Si(CH_3)_3$), 1.42-1.55/1.57-1.69 (m, 1H, H5), 1.87/2.11 (d, J = 13.5 Hz/J = 13.0 Hz, 1H, CH_2CO), 1.90/2.22 (d, J = 13.0 Hz/J = 13.5 Hz, 1H, CH_2CO), 2.17-2.44 (m, 3H, H4, H5), 4.34 (d, J = 15.4 Hz, 1H, C<u>H</u>$_2$Ph)/4.35 (s, 2H, C<u>H</u>$_2$Ph)/4.53 (d, J = 15.4 Hz, 1H, C<u>H</u>$_2$Ph), 4.99-5.06/5.82-5.88 (m, 1H, H1), 5.43-5.55 (m, 1H, H2), 5.79-5.95 (m, 1H, H3), 7.14-7.28 (m, 3H, Ar<u>H</u>), 7.30-7.38 (m, 2H, Ar<u>H</u>); ^{13}C NMR (101 MHz, $CDCl_3$): δ –0.69/–0.66 (q, $Si(CH_3)_3$), 26.1/26.7 (t, CH_2CO), 28.8/29.0 (t, C5), 31.4/31.5 (t, C4), 45.3/47.6 (t, <u>C</u>H_2Ph), 60.6/65.4 (d, C1), 126.0/127.1 (d, CH_{Ar}), 126.5/127.4 (d, CH_{Ar}), 128.2/128.8 (d, CH_{Ar}), 130.6/130.8 (d, C2), 134.9/135.1 (d, C3), 139.1/140.2 (s, C_{Ar}), 172.8/173.8 (s, C=O).

(*R*)-*N*-Benzyl-*N*-(cyclohex-2-en-1-yl)-2-(trimethylsilyl)acetamide (8m):

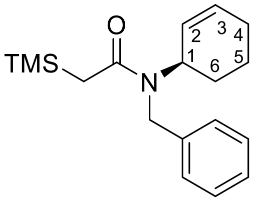

Prepared according to the general procedure, yield 5.0 g (76%) as a 2.1:1 mixture of rotamers. [R_f (hexanes/EtOAc 3:1) = 0.59]; IR (film); ν [cm^{-1}]: 3028 (w), 2948 (w), 2863 (w), 1626 (s), 1496 (w), 1452 (m), 1431 (w), 1396 (w), 1361 (w), 1313 (w), 1294 (w), 1249 (m), 1224 (w), 1203 (w), 1172 (w), 1138 (w), 1116 (w), 1076 (w), 1055 (w), 1029 (w), 989 (w), 854 (s), 727 (m), 700 (m), 619 (w); MS (+ESI) m/z, (%): 324 (40, $[M+Na]^+$), 302 (100, $[M+H]^+$); HRMS (+ESI) m/z [$C_{18}H_{28}NOSi^+$]: calcd. 302.1940; found 302.1942; $[\alpha]_D^{20}$: +44.1 (c 0.503, $CHCl_3$); ^{1}H NMR (400 MHz, $CDCl_3$): δ 0.11/0.17 (s, 9H, $Si(CH_3)_3$), 1.29-1.43/1.52-1.58 (m, 1H, H6), 1.59-1.79 (m, 2H, H5), 1.80-1.85/1.89-1.92 (m, 1H, H6), 1.93-2.01 (m, 2H, H4), 2.03/2.09 (d, *J* = 13.1 Hz/*J* = 13.0 Hz, 1H, CH_2CO), 2.16/2.22 (d, *J* = 13.0 Hz/*J* = 13.1 Hz, 1H, CH_2CO), 4.28-4.35/5.35-5.43 (m, 1H, H1), 4.39/4.40 (d, *J* = 15.3 Hz/*J* = 17.8 Hz, 1H, C$\underline{H}_2$Ph), 4.48/4.61 (d, *J* = 17.8 Hz/*J* = 15.3 Hz, 1H, C$\underline{H}_2$Ph), 5.06-5.24/5.35-5.48 (m, 1H, H2), 5.68-5.78/5.79-5.89 (m, 1H, H3), 7.17-7.37 (m, 5H, Ar$\underline{H}$); ^{13}C NMR (101 MHz, $CDCl_3$): δ –0.7/–0.6 (q, $Si(CH_3)_3$), 21.6/21.8 (t, C5), 24.6/24.8 (t, C4), 26.0/26.8 (t, $\underline{C}H_2CO$), 28.1/29.0 (t, C6), 46.2/48.1 (t, $\underline{C}H_2$Ph), 51.4/56.4 (d, C1), 126.0/126.5 (d, CH_{Ar}), 127.1/127.3 (d, CH_{Ar}), 128.48/128.53 (d, C2), 128.8 (d, CH_{Ar}), 131.7/131.9 (d, C3), 139.3/140.2 (s, C_{Ar}), 173.3/174.0 (s, C=O).

General procedure for the tandem nucleophilic epoxide opening/Brook rearrangement/ α-oxygenation:

In a similar manner as described in [11] LiCl (252 mg, 6 mmol) was added to a round-bottomed flask containing a stirring bar, which was sealed with a septum, and dried under vacuum by a heat gun. Dry THF (8 mL) and the amide **8** (1.0 mmol) were added under argon. The mixture was cooled to 0 °C in an ice/water bath, *sec*-butyllithium (1.4 M solution in cyclohexane, 0.8 mL, 1.1 mmol) was added dropwise by syringe and the mixture was stirred at 0 °C for 15 min. Then, epoxide **7** (1.05 mmol) was added at once by syringe and the reaction mixture was stirred at 0 °C for 1 h. The reaction mixture was cooled to −78 °C, diluted with dry THF (8 mL) and TEMPO (**3**; 164 mg, 1.05 mmol) was added as a solid in a single portion. Ferrocenium hexafluorophosphate (**4**; 397 mg, 1.2 mmol) was added in small portions with vigorous stirring until a dark blue-green color of the reaction mixture persisted for 20 min. The reaction mixture was quenched by saturated NH_4Cl solution (5 drops), diluted with diethyl ether (10 mL) and filtered through a pad of silica gel, which was washed with a fresh portion of diethyl ether. The filtrate was evaporated and the crude inhomogeneous mixture was purified by flash chromatography (gradient, hexanes/EtOAc 20:1 to 5:1) to give pure α-(aminoxy)amides **9**.

***N,N*-Diallyl-4-methyl-2-((2,2,6,6-tetramethylpiperidin-1-yl)oxy)-4-((trimethylsilyl)oxy)pentanamide (9a):**

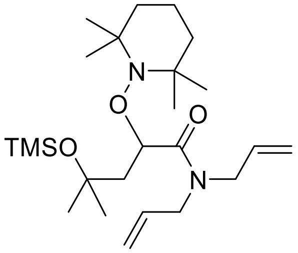

Prepared according to the general procedure, yield 313 mg (61%).

[R_f (hexanes/EtOAc 10:1) = 0.41]; IR (film); ν [cm^{-1}]: 2972 (w), 2927 (m), 1655 (m), 1450 (w), 1415 (w), 1363 (w), 1249 (m), 1230 (w), 1179 (w), 1134 (m), 1033 (m), 988 (w), 921 (m), 863 (m), 838 (s), 753 (w), 684 (w); MS (+ESI) m/z, (%): 461 (60, [$M+Na^+$]), 439 (100, [$M+H^+$]); HRMS (+ESI) m/z [$C_{24}H_{47}N_2O_3Si^+$]: calcd. 439.3351; found 439.3354; ^1H NMR (400 MHz, $CDCl_3$): δ 0.08 (s, 9H, $Si(CH_3)_3$), 0.99 (s, 3H, $NCCH_3$), 1.04 (s, 3H, $NCCH_3$), 1.08 (s, 3H, CCH_3), 1.11 (s, 3H, $NCCH_3$), 1.22 (s, 3H, $NCCH_3$), 1.24-1.33 (m, 1H, piperidine-H4), 1.29 (s, 3H, CCH_3), 1.35-1.48 (m, 4H, piperidine-H3, H5), 1.49-1.60 (m, 1H, piperidine-H4), 1.96 (dd, *J*

= 13.4, 2.1 Hz, 1H, $\mathrm{C\underline{H}_2}$CHOTMP), 2.30 (dd, *J* = 13.4, 10.6 Hz, 1H, $\mathrm{C\underline{H}_2}$CHOTMP), 3.81-4.00 (m, 3H, $\mathrm{C\underline{H}_2}$CH=), 4.51-4.59 (m, 1H, $\mathrm{C\underline{H}_2}$CH=), 4.77 (dd, *J* = 10.6, 2.1 Hz, 1H, $\mathrm{C\underline{H}}$OTMP), 5.12-5.26 (m, 4H, CH=$\mathrm{C\underline{H}_2}$), 5.77 (ddt, *J* = 16.9, 10.0, 6.6 Hz, 1H, $\mathrm{C\underline{H}}$=$\mathrm{CH_2}$), 6.03 (ddt, *J* = 16.4, 10.1, 6.2 Hz, 1H, $\mathrm{C\underline{H}}$=$\mathrm{CH_2}$); ^{13}C NMR (101 MHz, $\mathrm{CDCl_3}$): δ 2.7 (q, $\mathrm{Si(CH_3)_3}$), 17.3 (t, piperidine-C4), 20.3 (q, $\mathrm{NC\underline{C}H_3}$), 20.6 (q, $\mathrm{NC\underline{C}H_3}$), 29.5 (q, $\mathrm{C\underline{C}H_3}$), 31.9 (q, $\mathrm{C\underline{C}H_3}$), 33.0 (q, $\mathrm{NC\underline{C}H_3}$), 33.9 (q, $\mathrm{NC\underline{C}H_3}$), 40.4 (t, piperidine-C3), 40.6 (t, piperidine-C5), 46.6 (t, $\mathrm{\underline{C}H_2}$CHOTMP), 48.1 (t, $\mathrm{\underline{C}H_2}$CH=), 49.5 (t, $\mathrm{\underline{C}H_2}$CH=), 59.4 (s, CNO), 60.6 (s, CNO), 72.8 (s, $\mathrm{\underline{C}}$OTMS), 78.5 (d, $\mathrm{\underline{C}}$HOTMP), 118.1 (t, CH=$\mathrm{\underline{C}H_2}$), 118.3 (t, CH=$\mathrm{\underline{C}H_2}$), 133.2 (d, $\mathrm{\underline{C}H}$=$\mathrm{CH_2}$), 134.3 (d, $\mathrm{\underline{C}H}$=$\mathrm{CH_2}$), 172.8 (s, C=O).

***N,N*-Diallyl-2-((2,2,6,6-tetramethylpiperidin-1-yl)oxy)-4-((trimethylsilyl)oxy)pentanamide (9b):**

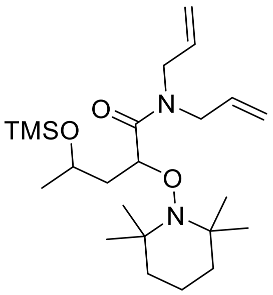

Prepared according to the general procedure, yield 326 mg (77%) as an inseparable 1.1:1 mixture of diastereomers.

[R_f (hexanes/EtOAc 5:1) = 0.63]; IR (film); ν [cm^{-1}]: 2967 (w), 2955 (w), 1651 (s), 1461 (w), 1438 (w), 1411 (m), 1376 (w), 1281 (w), 1249 (m), 1215 (w), 1194 (w), 1137 (w), 1085 (m), 1047 (w), 1018 (w), 985 (m), 921 (m), 891 (w), 869 (w), 836 (s), 758 (m), 683 (w); MS (+ESI) m/z, (%): 447 (15, [M+Na$^+$]), 425 (100, [M+H$^+$]); HRMS (+ESI) m/z [$\mathrm{C_{23}H_{45}N_2O_3Si^+}$]: calcd. 425.3194; found 425.3193.

First diastereomer: ^{1}H NMR (400 MHz, $\mathrm{CDCl_3}$): δ 0.09 (s, 9H, $\mathrm{Si(CH_3)_3}$), 1.06 (s, 6H, $\mathrm{NCCH_3}$), 1.11 (s, 3H, $\mathrm{NCCH_3}$), 1.13 (s, 3H, $\mathrm{NCCH_3}$), 1.19 (d, *J* = 6.1 Hz, 3H, $\mathrm{C\underline{H}_3}$CHOTMS), 1.24-1.34 (m, 1H, piperidine-H4), 1.35-1.49 (m, 4H, piperidine-H3, H5), 1.50-1.61 (m, 1H, piperidine-H4), 1.91-2.09 (m, 2H, $\mathrm{C\underline{H}_2}$CHOTMP), 3.70-3.77 (m, 1H, $\mathrm{C\underline{H}}$OTMS), 3.83-3.90 (m, 1H, $\mathrm{C\underline{H}_2}$CH=), 3.91-3.98 (m, 2H, $\mathrm{C\underline{H}_2}$CH=), 4.07 (dd, *J* = 15.4, 6.0 Hz, 1H, $\mathrm{C\underline{H}_2}$CH=), 4.61-4.72 (m, 1H, $\mathrm{C\underline{H}}$OTMP), 5.12-5.18 (m, 4H, CH=$\mathrm{C\underline{H}_2}$), 5.71-5.83 (m, 2H, $\mathrm{C\underline{H}}$=$\mathrm{CH_2}$); ^{13}C NMR (101 MHz, $\mathrm{CDCl_3}$): δ 0.4 (q, $\mathrm{Si(CH_3)_3}$), 17.1 (t, piperidine-C4), 20.1 (q, 2C, $\mathrm{NC\underline{C}H_3}$), 24.6 (q,

$\underline{C}H_3$CHOTMS), 33.49 (q, NC$\underline{C}H_3$), 33.53 (q, NC$\underline{C}H_3$), 40.4 (t, piperidine-C3, C5), 47.7 (t, $\underline{C}H_2$CH=), 48.2 (t, $\underline{C}H_2$CH=), 59.3 (s, CNO), 60.6 (s, CNO), 65.7 (d, $\underline{C}$HOTMS), 78.7 (d, $\underline{C}$HOTMP), 118.0 (t, CH=$\underline{C}H_2$), 118.2 (t, CH=$\underline{C}H_2$), 133.07 (d, $\underline{C}$H=CH_2), 133.10 (d, $\underline{C}$H=CH_2), 172.6 (s, C=O).

Second diastereomer: ^{1}H NMR (400 MHz, $CDCl_3$): δ 0.10 (s, 9H, $Si(CH_3)_3$), 1.02 (s, 3H, $NCCH_3$), 1.03 (s, 3H, $NCCH_3$), 1.15 (d, J = 6.2 Hz, 3H, C$\underline{H}_3$CHOTMS), 1.20 (s, 3H, $NCCH_3$), 1.22 (s, 3H, $NCCH_3$), 1.24-1.34 (m, 1H, piperidine-H4), 1.35-1.49 (m, 4H, piperidine-H3, H5), 1.50-1.61 (m, 1H, piperidine-H4), 1.91-2.09 (m, 2H, C$\underline{H}_2$CHOTMP), 3.76-3.82 (m, 1H, C$\underline{H}_2$CH=), 3.87-3.92 (m, 1H, C$\underline{H}$OTMS), 3.99-4.11 (m, 1H, C$\underline{H}_2$CH=), 4.36 (dd, J = 16.5, 6.0 Hz, 1H, C$\underline{H}_2$CH=), 4.59 (dd, J = 7.8, 5.9 Hz, 1H, C$\underline{H}$OTMP), 4.62-4.71 (m, 1H, C$\underline{H}_2$CH=), 5.19-5.24 (m, 4H, CH=C$\underline{H}_2$), 5.84-6.01 (m, 2H, C$\underline{H}$=CH_2); ^{13}C NMR (101 MHz, $CDCl_3$): δ 0.6 (q, $Si(CH_3)_3$), 17.0 (t, piperidine-C4), 20.4 (q, NC$\underline{C}H_3$), 20.5 (q, NC$\underline{C}H_3$), 23.8 (q, $\underline{C}H_3$CHOTMS), 32.8 (q, NC$\underline{C}H_3$), 33.0 (q, NC$\underline{C}H_3$), 40.3 (t, piperidine-C3), 40.5 (t, piperidine-C3), 48.9 (t, $\underline{C}H_2$CH=), 49.1 (t, $\underline{C}H_2$CH=), 59.4 (s, CNO), 60.4 (s, CNO), 65.3 (d, $\underline{C}$HOTMS), 78.1 (d, $\underline{C}$HOTMP), 117.3 (t, CH=$\underline{C}H_2$), 117.6 (t, CH=$\underline{C}H_2$), 133.7 (d, $\underline{C}$H=CH_2), 134.1 (d, $\underline{C}$H=CH_2), 172.1 (s, C=O).

***N,N*-Diallyl-2-((2,2,6,6-tetramethylpiperidin-1-yl)oxy)-4-((trimethylsilyl)oxy)octanamide (9c):**

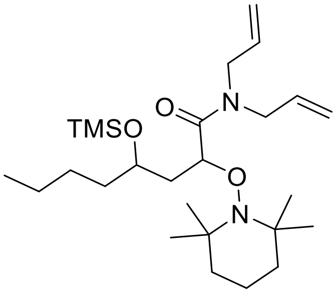

Prepared according to the general procedure, yield 336 mg (71%) as an inseparable 1:1 mixture of diastereomers.

[R_f (hexanes/EtOAc 10:1) = 0.42]; IR (film); ν [cm^{-1}]: 3007 (w), 2956 (m), 2930 (s), 2872 (m), 1653 (s), 1457 (m), 1418 (m), 1377 (m), 1362 (w), 1348 (w), 1250 (m), 1232 (w), 1184 (w), 1132 (w), 1061 (m), 990 (m), 956 (w), 921 (w), 840 (s), 754 (m), 714 (m), 684 (w), 613 (w); MS (+ESI) m/z, (%): 955 (35, [2M+Na^+]), 489 (100, [M+Na^+]), 467 (5, [M+H^+]); HRMS (+ESI) m/z [$C_{26}H_{50}N_2O_3SiNa^+$]: calcd. 489.3483; found 489.3484.

First diastereomer: ^{1}H NMR (400 MHz, $CDCl_3$): δ 0.09 (s, 9H, $Si(CH_3)_3$), 0.84-0.92 (m, 3H, $C\underline{H}_3CH_2$), 1.01 (s, 3H, $NCCH_3$), 1.04 (s, 3H, $NCCH_3$), 1.12 (s, 6H, $NCCH_3$), 1.24-1.35 (m, 5H, $CH_3C\underline{H}_2C\underline{H}_2$, piperidine-H4), 1.40-1.50 (m, 6H, $CH_2C\underline{H}_2$CHOTMS, piperidine-H3, H5), 1.53-1.60 (m, 1H, piperidine-H4), 1.95-2.10 (m, 2H, $C\underline{H}_2$CHOTMP), 3.58-3.69 (m, 1H, $C\underline{H}$OTMS), 3.80 (dd, J = 14.4, 7.4 Hz, 1H, $C\underline{H}_2CH$=), 3.91-3.96 (m, 2H, $C\underline{H}_2CH$=), 4.04 (dd, J = 14.4, 6.0 Hz, 1H, $C\underline{H}_2CH$=), 4.62-4.66 (m, 1H, $C\underline{H}$OTMP), 5.12-5.18 (m, 4H, $CH=C\underline{H}_2$), 5.71-5.84 (m, 2H, $C\underline{H}=CH_2$); ^{13}C NMR (101 MHz, $CDCl_3$): δ 0.8 (q, $Si(CH_3)_3$), 14.2 (q, $\underline{C}H_3CH_2$), 17.18 (t, piperidine-C4), 20.2 (q, $NC\underline{C}H_3$), 20.5 (q, $NC\underline{C}H_3$), 22.89 (t, $CH_3\underline{C}H_2CH_2$), 27.5 (t, $CH_3CH_2\underline{C}H_2$), 33.0 (q, $NC\underline{C}H_3$), 33.7 (q, $NC\underline{C}H_3$), 37.8 (t, $CH_2\underline{C}H_2$CHOTMS), 39.8 (t, $\underline{C}H_2$CHOTMP), 40.5 (t, piperidine-C3, C5), 47.9 (t, $\underline{C}H_2CH$=), 48.4 (t, $\underline{C}H_2CH$=), 57.0 (s, CNO), 59.4 (s, CNO), 69.8 (d, $\underline{C}$HOTMS), 79.2 (d, $\underline{C}$HOTMP), 118.1 (t, $CH=\underline{C}H_2$), 118.4 (t, $CH=\underline{C}H_2$), 133.20 (d, $\underline{C}H=CH_2$), 133.24 (d, $\underline{C}H=CH_2$), 172.2 (s, C=O).

Second diastereomer: δ 0.10 (s, 9H, $Si(CH_3)_3$), 0.84-0.92 (m, 3H, $C\underline{H}_3CH_2$), 1.06 (s, 3H, $NCCH_3$), 1.07 (s, 3H, $NCCH_3$), 1.18 (s, 3H, $NCCH_3$), 1.21 (s, 3H, $NCCH_3$), 1.24-1.35 (m, 5H, $CH_3C\underline{H}_2C\underline{H}_2$, piperidine-H4), 1.36-1.40 (m, 2H, $CH_2C\underline{H}_2$CHOTMS), 1.40-1.50 (m, 4H, piperidine-H3, H5), 1.53-1.60 (m, 1H, piperidine-H4), 1.95-2.10 (m, 2H, $C\underline{H}_2$CHOTMP), 3.58-3.69 (m, 1H, $C\underline{H}$OTMS), 3.87 (dd, J = 16.0, 6.0 Hz, 1H, $C\underline{H}_2CH$=), 3.95-4.03 (m, 1H, $C\underline{H}_2CH$=), 4.40 (dd, J = 16.0, 6.0 Hz, 1H, $C\underline{H}_2CH$=), 4.58 (dd, J = 8.2, 5.7 Hz, 1H, $C\underline{H}$OTMP), 4.64-4.70 (m, 1H, $C\underline{H}_2CH$=), 5.19-5.23 (m, 4H, $CH=C\underline{H}_2$), 5.85-5.98 (m, 2H, $C\underline{H}=CH_2$); ^{13}C NMR (101 MHz, $CDCl_3$): δ 0.6 (q, $Si(CH_3)_3$), 14.1 (q, $\underline{C}H_3CH_2$), 17.22 (t, piperidine-C4), 20.3 (q, $NC\underline{C}H_3$), 20.6 (q, $NC\underline{C}H_3$), 22.86 (t, $CH_3\underline{C}H_2CH_2$), 27.9 (t, $CH_3CH_2\underline{C}H_2$), 33.1 (q, $NC\underline{C}H_3$), 33.6 (q, $NC\underline{C}H_3$), 36.9 (t, $CH_2\underline{C}H_2$CHOTMS), 40.2 (t, $\underline{C}H_2$CHOTMP), 40.6 (t, piperidine-C3, C5), 49.1 (t, $\underline{C}H_2CH$=), 49.2 (t, $\underline{C}H_2CH$=), 57.1 (s, CNO), 59.6 (s, CNO), 69.5 (d, $\underline{C}$HOTMS), 77.7 (d, $\underline{C}$HOTMP), 117.4 (t, $CH=\underline{C}H_2$), 117.6 (t, $CH=\underline{C}H_2$), 133.9 (d, $\underline{C}H=CH_2$), 134.2 (d, $\underline{C}H=CH_2$), 172.7 (s, C=O).

***N*,*N*-Diallyl-4-phenyl-2-((2,2,6,6-tetramethylpiperidin-1-yl)oxy)-4-((trimethylsilyl)oxy)butanamide (9d):**

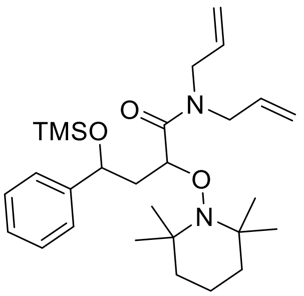

Prepared according to the general procedure, yield 265 mg (56%) as an inseparable 1.1:1 mixture of diastereomers.

[R_f (hexanes/EtOAc 10:1) = 0.51]; IR (film); ν [cm^{-1}]: 3084 (w), 3006 (w), 2930 (m), 2872 (w), 1654 (s), 1493 (w), 1453 (m), 1415 (m), 1377 (w), 1362 (w), 1250 (m), 1183 (w), 1133 (w), 1090 (m), 994 (w), 960 (w), 921 (w), 865 (m), 842 (s), 752 (m), 701 (m), 685 (w), 614 (w); MS (+ESI) m/z, (%): 509 (95, [$M+Na^+$]), 487 (100, [$M+H^+$]); HRMS (+ESI) m/z [$C_{28}H_{46}N_2O_3SiNa^+$]: calcd. 509.3170; found 509.3167.

First diastereomer: ^{1}H NMR (400 MHz, $CDCl_3$): δ –0.05 (s, 9H, $Si(CH_3)_3$), 1.06 (s, 12H, $NCCH_3$), 1.25-1.34 (m, 1H, piperidine-H4), 1.37-1.48 (m, 4H, piperidine-H3, H5), 1.49-1.56 (m, 1H, piperidine-H4), 2.21-2.39 (m, 2H, C$\underline{H}_2$CHOTMP), 3.82 (dd, *J* = 16.0, 5.0 Hz, 1H, C$\underline{H}_2$CH=), 4.11 (dd, *J* = 16.4, 5.7 Hz, 1H, C$\underline{H}_2$CH=), 4.33 (dd, *J* = 16.4, 5.6 Hz, 1H, C$\underline{H}_2$CH=), 4.38 (dd, *J* = 16.0, 5.0 Hz, 1H, C$\underline{H}_2$CH=), 4.56 (dd, *J* = 9.7, 2.9 Hz, 1H, C$\underline{H}$OTMS), 4.80 (dd, *J* = 10.6, 3.2 Hz, 1H, C$\underline{H}$OTMP), 5.12-5.27 (m, 4H, CH=C$\underline{H}_2$), 5.70-5.85 (m, 2H, C$\underline{H}$=CH_2), 7.20-7.35 (m, 5H, Ar$\underline{H}$); ^{13}C NMR (101 MHz, $CDCl_3$): δ 0.1 (q, $Si(CH_3)_3$), 16.9 (t, piperidine-C4), 20.0 (q, 2C, NC$\underline{C}H_3$), 32.7 (q, 2C, NC$\underline{C}H_3$), 40.2 (t, piperidine-C3, C5), 42.3 (t, $\underline{C}H_2$CHOTMP), 48.8 (t, $\underline{C}H_2$CH=), 49.1 (t, $\underline{C}H_2$CH=), 59.2 (s, CNO), 60.1 (s, CNO), 71.9 (d, $\underline{C}$HOTMS), 79.3 (d, $\underline{C}$HOTMP), 117.8 (t, 2C, CH=$\underline{C}H_2$), 126.1 (d, CH_{Ar}), 127.0 (d, CH_{Ar}), 127.9 (d, CH_{Ar}), 132.99 (d, $\underline{C}$H=CH_2), 133.04 (d, $\underline{C}$H=CH_2), 143.7 (s, C_{Ar}), 171.5 (s, C=O).

Second diastereomer: ^{1}H NMR (400 MHz, $CDCl_3$): δ –0.02 (s, 9H, $Si(CH_3)_3$), 0.97 (s, 3H, $NCCH_3$), 1.10 (s, 3H, $NCCH_3$), 1.15 (s, 6H, $NCCH_3$), 1.25-1.34 (m, 1H, piperidine-H4), 1.37-1.48 (m, 4H, piperidine-H3, H5), 1.49-1.56 (m, 1H, piperidine-H4), 2.21-2.39 (m, 2H, C$\underline{H}_2$CHOTMP), 3.86 (dd, *J* = 13.4, 6.6 Hz, 1H, C$\underline{H}_2$CH=), 3.92 (dd, *J* = 13.4, 6.6 Hz, 1H, C$\underline{H}_2$CH=), 3.95 (dd, *J* = 14.6, 6.7 Hz, 1H, C$\underline{H}_2$CH=), 4.05 (dd, *J* = 14.6, 6.7 Hz, 1H, C$\underline{H}_2$CH=), 4.45-4.50 (m, 1H, C$\underline{H}$OTMP), 4.75 (t, *J* = 6.6 Hz, 1H, C$\underline{H}$OTMS), 5.12-5.27 (m, 4H, CH=C$\underline{H}_2$),

5.78-5.88 (m, 1H, $C\underline{H}=CH_2$), 5.96 (ddt, *J* = 17.6, 9.9, 6.5 Hz, 1H, $C\underline{H}=CH_2$), 7.20-7.35 (m, 5H, $Ar\underline{H}$); ^{13}C NMR (101 MHz, $CDCl_3$): δ 0.2 (q, $Si(CH_3)_3$), 17.0 (t, piperidine-C4), 20.3 (q, $NC\underline{C}H_3$), 20.4 (q, $NC\underline{C}H_3$), 33.2 (q, $NC\underline{C}H_3$), 33.4 (q, $NC\underline{C}H_3$), 40.1 (t, piperidine-C3), 40.3 (t, piperidine-C5), 42.5 (t, $\underline{C}H_2CHOTMP$), 47.4 (t, $\underline{C}H_2CH=$), 47.7 (t, $\underline{C}H_2CH=$), 59.4 (s, CNO), 60.4 (s, CNO), 72.0 (d, $\underline{C}HOTMS$), 78.0 (d, $\underline{C}HOTMP$), 117.4 (t, $CH=\underline{C}H_2$), 118.0 (t, $CH=\underline{C}H_2$), 126.7 (d, CH_{Ar}), 127.3 (d, CH_{Ar}), 128.0 (d, CH_{Ar}), 133.6 (d, $\underline{C}H=CH_2$), 134.0 (d, $\underline{C}H=CH_2$), 144.8 (s, C_{Ar}), 171.9 (s, C=O).

***N*-Allyl-*N*-methyl-2-((2,2,6,6-tetramethylpiperidin-1-yl)oxy)-4-((trimethylsilyl)oxy)pentanamide (9e):**

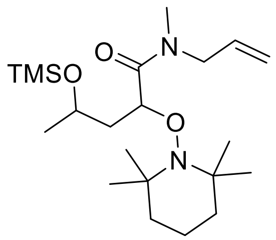

Prepared according to the general procedure, yield 230 mg (68%) as a partly separable 1.1:1 mixture of diastereomers. Ratio of rotamers is 1.5:1 for each diastereomer.

[R_f (hexanes/EtOAc 5:1) = 0.51]; IR (film); ν [cm^{-1}]: 2967 (m), 2930 (m), 2873 (w), 1655 (s), 1456 (w), 1403 (w), 1376 (w), 1362 (w), 1250 (m), 1143 (w), 1134 (w), 1100 (w), 1083 (w), 1057 (w), 1021 (w), 990 (w), 974 (w), 956 (w), 922 (w), 899 (w), 841 (s), 750 (w), 708 (w); MS (+ESI) m/z, (%): 819 (20, [$2M+Na^+$]), 421 (100, [$M+Na^+$]), 399 (65, [$M+H^+$]); HRMS (+ESI) m/z [$C_{21}H_{42}N_2O_3SiNa^+$]: calcd. 421.2857; found 421.2856.

Less polar diastereomer: ^{1}H NMR (400 MHz, $CDCl_3$): δ 0.06/0.07 (s, 9H, $Si(CH_3)_3$), 1.00 (s, 3H, $NCCH_3$), 1.05 (s, 3H, $NCCH_3$), 1.10 (s, 3H, $NCCH_3$), 1.16 (d, *J* = 6.1 Hz, 3H, $C\underline{H}_3CHOTMS$), 1.19/1.22 (s, 3H, $NCCH_3$), 1.24-1.33 (m, 1H, piperidine-H4), 1.35-1.47 (m, 4H, piperidine-H3, H5), 1.48-1.58 (m, 1H, piperidine-H4), 1.88-1.98/1.99-2.08 (m, 2H, $C\underline{H}_2CHOTMP$), 2.85/3.14 (s, 3H, NCH_3), 3.66/3.73 (ddq, *J* = 9.5, 6.1, 3.3 Hz/*J* = 11.7, 6.0, 3.0 Hz, 1H, $C\underline{H}OTMS$), 3.91/4.06 (dd, *J* = 14.4, 6.4 Hz/*J* = 16.4, 5.9 Hz, 1H, $C\underline{H}_2CH=$), 4.03/4.37 (dd, *J* = 14.4, 6.4 Hz/*J* = 16.4, 5.9 Hz, 1H, $C\underline{H}_2CH=$), 4.67/4.75 (dd, *J* = 10.0, 3.8 Hz/*J* = 10.8, 3.4 Hz, 1H, $C\underline{H}OTMP$), 5.14-5.23 (m, 2H, $CH=C\underline{H}_2$), 5.73/5.92 (ddt, *J* = 16.6, 10.0, 6.4 Hz, 1H, $C\underline{H}=CH_2$); ^{13}C NMR (101 MHz, $CDCl_3$): δ 0.7 (q, $Si(CH_3)_3$), 17.24 (t, piperidine-C4), 20.1/20.24 (q, $NC\underline{C}H_3$), 20.5/20.7 (q, $NC\underline{C}H_3$), 24.6/24.8 (q, $\underline{C}H_3CHOTMS$), 32.56/33.0 (q, $NC\underline{C}H_3$), 33.4/35.0 (q,

NCH$_3$), 33.6/33.7 (q, NC$\underline{C}$H$_3$), 40.4/40.59 (t, piperidine-C3, C5), 42.0/42.1 (t, $\underline{C}$H$_2$CHOTMP), 50.6/52.6 (t, $\underline{C}$H$_2$CH=), 59.3/59.52 (s, CNO), 60.5/60.59 (s, CNO), 65.8/65.9 (d, $\underline{C}$HOTMS), 78.79/78.84 (d, $\underline{C}$HOTMP), 117.9/118.08 (t, CH=$\underline{C}$H$_2$), 133.2/134.0 (d, $\underline{C}$H=CH$_2$), 172.6/172.7 (s, C=O).

More polar diastereomer: ^{1}H NMR (400 MHz, CDCl$_3$): δ 0.08/0.09 (s, 9H, Si(CH$_3$)$_3$), 0.97/1.00 (s, 3H, NCCH$_3$), 1.05 (s, 3H, NCCH$_3$), 1.10/1.11 (s, 3H, NCCH$_3$), 1.13/1.14 (d, *J* = 6.2 Hz, 3H, C$\underline{H}_3$CHOTMS), 1.22 (s, 3H, NCCH$_3$), 1.24-1.31 (m, 1H, piperidine-H4), 1.35-1.47 (m, 4H, piperidine-H3, H5), 1.48-1.58 (m, 1H, piperidine-H4), 1.93-2.00/2.01-2.06 (m, 2H, C$\underline{H}_2$CHOTMP), 2.85/3.14 (s, 3H, NCH$_3$), 3.79/3.82-3.87 (sext, *J* = 6.3 Hz/m, 1H, C$\underline{H}$OTMS), 3.84/3.89 (dd, *J* = 17.0, 5.2 Hz/*J* = 14.5, 6.2 Hz, 1H, C$\underline{H}_2$CH=), 4.02/4.70 (dd, *J* = 14.5, 6.5 Hz/*J* = 17.0, 5.4 Hz, 1H, C$\underline{H}_2$CH=), 4.55-4.61 (m, 1H, C$\underline{H}$OTMP), 5.13-5.23 (m, 2H, CH=C$\underline{H}_2$), 5.74/5.88 (ddt, *J* = 16.6, 10.1, 6.5 Hz/*J* = 17.1, 10.5, 5.4 Hz, 1H, C$\underline{H}$=CH$_2$); ^{13}C NMR (101 MHz, CDCl$_3$): δ 0.3/0.6 (q, Si(CH$_3$)$_3$), 17.22 (t, piperidine-C4), 20.18/20.3 (q, NC$\underline{C}$H$_3$), 20.56/20.63 (q, NC$\underline{C}$H$_3$), 23.6/23.9 (q, $\underline{C}$H$_3$CHOTMS), 32.63/33.2 (q, NC$\underline{C}$H$_3$), 33.4/35.0 (q, NCH$_3$), 33.8/34.7 (q, NC$\underline{C}$H$_3$), 40.5/40.57 (t, piperidine-C3), 40.61/40.7 (t, piperidine-C5), 42.2/42.5 (t, $\underline{C}$H$_2$CHOTMP), 50.7/52.5 (t, $\underline{C}$H$_2$CH=), 59.4/59.53 (s, CNO), 60.56/60.8 (s, CNO), 65.50/65.54 (d, $\underline{C}$HOTMS), 78.3/80.7 (d, $\underline{C}$HOTMP), 117.3/118.11 (t, CH=$\underline{C}$H$_2$), 133.1/133.6 (d, $\underline{C}$H=CH$_2$), 172.4/173.0 (s, C=O).

***N*-Benzyl-*N*-(2-methylallyl)-2-((2,2,6,6-tetramethylpiperidin-1-yl)oxy)-4-((trimethylsilyl)oxy)pentanamide (9f):**

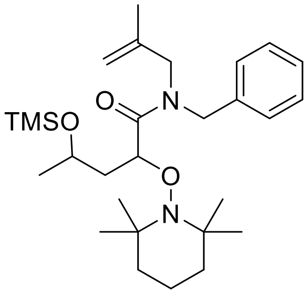

Prepared according to the general procedure, yield 170 mg (51%) as an inseparable 1:1 mixture of diastereomers. Ratio of rotamers is 1.9:1 for each diastereomer.

[R_f (hexanes/EtOAc 5:1) = 0.56]; IR (film); ν [cm^{-1}]: 2969 (m), 2930 (m), 1655 (s), 1495 (m), 1444 (m), 1376 (w), 1362 (w), 1250 (s), 1205 (w), 1182 (w), 1100 (m), 1080 (w), 1049 (w), 1013 (w), 992 (w), 958 (w), 895 (w), 840 (s), 748 (m), 702 (m); MS (+ESI) m/z, (%): 999 (30,

[2M+Na$^+$]), 511 (100, [M+Na$^+$]), 489 (40, [M+H$^+$]); HRMS (+ESI) m/z [$C_{28}H_{48}N_2O_3SiNa^+$]: calcd. 511.3326; found 511.3325.

First diastereomer: ^{1}H NMR (400 MHz, $CDCl_3$): δ 0.09 (s, 9H, $Si(CH_3)_3$), 1.05 (s, 3H, $NCCH_3$), 1.13 (s, 6H, $NCCH_3$), 1.16 (s, 3H, $NCCH_3$), 1.22 (d, *J* = 6.1 Hz, 3H, $C\underline{H}_3CHOTMS$), 1.26-1.34 (m, 1H, piperidine-H4), 1.36-1.51 (m, 4H, piperidine-H3, H5), 1.52-1.62 (m, 1H, piperidine-H4), 1.75/1.76 (s, 3H, CH_3C=), 2.01-2.15 (m, 1H, $C\underline{H}_2CHOTMP$), 2.22/2.30 (ddd, *J* = 13.6, 8.1, 4.0 Hz/*J* = 12.2, 9.7, 3.5 Hz, 1H, $C\underline{H}_2CHOTMP$), 3.68/3.84 (d, *J* = 17.6 Hz/*J* = 18.3 Hz, 1H, NCH_2C=), 3.94-4.03 (m, 1H, $C\underline{H}OTMS$), 4.20/4.40 (d, *J* = 14.1 Hz/*J* = 14.3 Hz, 1H, $C\underline{H}_2Ph$), 4.42/4.68 (d, *J* = 18.3 Hz/*J* = 17.6 Hz, 1H, NCH_2C=), 4.64/4.91 (dd, *J* = 9.1, 4.0 Hz/*J* = 6.9, 3.5 Hz, 1H, $C\underline{H}OTMP$), 4.77/4.89 (d, *J* = 14.1 Hz/*J* = 14.3 Hz, 1H, $C\underline{H}_2Ph$), 4.79/4.82 (d, *J* = 1.2 Hz, 1H, C=CH_2), 4.93/4.99 (d, *J* = 1.2 Hz, 1H, C=CH_2), 7.28-7.45 (m, 5H, Ar$\underline{H}$); ^{13}C NMR (101 MHz, $CDCl_3$): δ 0.01/0.03 (q, $Si(CH_3)_3$), 16.64/16.66 (t, piperidine-C4), 19.7/19.9 (q, $\underline{C}H_3C$=, $NC\underline{C}H_3$), 23.4/23.6 (q, $\underline{C}H_3CHOTMS$), 32.7 (q, $NC\underline{C}H_3$), 33.1 (q, $NC\underline{C}H_3$), 39.9 (t, piperidine-C3, C5), 40.2/40.8 (t, $\underline{C}H_2CHOTMP$), 47.9/48.9 (t, $\underline{C}H_2Ph$), 49.0/51.0 (t, $N\underline{C}H_2C$=), 59.5 (s, 2C, CNO), 64.9/65.1 (d, $\underline{C}HOTMS$), 77.8/78.0 (d, $\underline{C}HOTMP$), 111.2/112.4 (t, C=$\underline{C}H_2$), 126.8/126.93 (d, CH_{Ar}), 127.00/127.7 (d, CH_{Ar}), 128.1/128.7 (d, CH_{Ar}), 136.4/136.5 (s, C_{Ar}), 139.5/139.9 (s, $\underline{C}$=CH_2), 171.1/172.7 (s, C=O).

Second diastereomer: ^{1}H NMR (400 MHz, $CDCl_3$): δ 0.11/0.13 (s, 9H, $Si(CH_3)_3$), 1.08 (s, 3H, $NCCH_3$), 1.10 (s, 3H, $NCCH_3$), 1.12 (s, 6H, $NCCH_3$), 1.18 (d, *J* = 6.1 Hz, 3H, $C\underline{H}_3CHOTMS$), 1.26-1.34 (m, 1H, piperidine-H4), 1.36-1.51 (m, 4H, piperidine-H3, H5), 1.52-1.62 (m, 1H, piperidine-H4), 1.73 (s, 3H, CH_3C=), 1.98 (dt, *J* = 13.6, 6.7 Hz, 1H, $C\underline{H}_2CHOTMP$), 2.01-2.15 (m, 1H, $C\underline{H}_2CHOTMP$), 3.79/3.89 (d, *J* = 15.5 Hz/*J* = 14.9 Hz, 1H, NCH_2C=) 3.82-3.85 (m, 1H, $C\underline{H}OTMS$), 4.02/4.09 (d, *J* = 14.8 Hz/*J* = 15.5 Hz, 1H, NCH_2C=), 4.58/4.82 (t, *J* = 6.7 Hz, 1H, $C\underline{H}OTMP$), 4.63/4.75 (d, *J* = 17.0 Hz/*J* = 14.8 Hz, 1H, $C\underline{H}_2Ph$), 4.87/4.98 (d, *J* = 14.8 Hz/*J* = 17.0 Hz, 1H, $C\underline{H}_2Ph$), 4.89/4.92 (d, *J* = 1.4 Hz, 1H, C=CH_2), 4.91/5.00 (d, *J* = 1.4 Hz, 1H, C=CH_2), 7.28-7.45 (m, 5H, Ar$\underline{H}$); ^{13}C NMR (101 MHz, $CDCl_3$): δ 0.1/0.2 (q, $Si(CH_3)_3$), 16.56/16.73 (t, piperidine-C4), 20.0/20.2 (q, $\underline{C}H_3C$=, $NC\underline{C}H_3$), 24.1/24.2 (q, $\underline{C}H_3CHOTMS$), 32.8 (q, $NC\underline{C}H_3$), 33.1 (q, $NC\underline{C}H_3$), 39.96 (t, piperidine-C3), 40.03 (t, piperidine-C5), 41.5/42.1 (t, $\underline{C}H_2CHOTMP$), 48.5/49.2 (t, $\underline{C}H_2Ph$), 49.9/51.2 (t, $N\underline{C}H_2C$=), 59.0/59.2 (s, CNO), 59.8/60.0 (s, CNO), 65.3/65.6 (d, $\underline{C}HOTMS$), 78.1/78.4 (d, $\underline{C}HOTMP$), 111.3/112.9 (t, C=$\underline{C}H_2$), 126.85/126.97 (d, CH_{Ar}), 127.2/127.8 (d, CH_{Ar}), 128.1/129.3 (d, CH_{Ar}), 136.56/136.63 (s, C_{Ar}), 139.7/140.2 (s, $\underline{C}$=CH_2), 171.8/171.9 (s, C=O).

***N*-Benzyl-*N*-(3-methylbut-2-en-1-yl)-2-((2,2,6,6-tetramethylpiperidin-1-yl)oxy)-4-((trimethylsilyl)oxy)pentanamide (9g):**

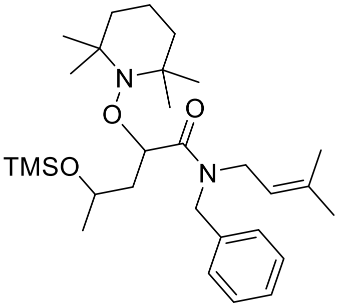

Prepared according to the general procedure, yield 271 mg (53%) as an inseparable 1:1 mixture of diastereomers. Ratio of rotamers is 1.2:1 for each diastereomer.

[R_f (hexanes/EtOAc 5:1) = 0.67]; IR (film); ν [cm^{-1}]: 2967 (m), 2929 (m), 1650 (s), 1495 (m), 1448 (m), 1376 (w), 1362 (w), 1250 (s), 1205 (w), 1133 (w), 1098 (m), 1079 (w), 1063 (w), 1020 (w), 990 (w), 973 (w), 957 (w), 892 (w), 840 (s), 748 (m), 701 (m); MS (+ESI) m/z, (%): 1027 (10, [$2M+Na^+$]), 525 (25, [$M+Na^+$]), 503 (100, [$M+H^+$]); HRMS (+ESI) m/z [$C_{29}H_{51}N_2O_3Si^+$]: calcd. 503.3664; found 503.3660.

First diastereomer: ^{1}H NMR (400 MHz, $CDCl_3$): δ 0.03 (s, 9H, $Si(CH_3)_3$), 1.06 (s, 3H, $NCCH_3$), 1.07 (s, 3H, $NCCH_3$), 1.08 (s, 3H, $NCCH_3$), 1.10 (s, 3H, $NCCH_3$), 1.15/1.20 (d, *J* = 6.1 Hz, 3H, C$\underline{H}_3$CHOTMS), 1.24-1.35 (m, 1H, piperidine-H4), 1.36-1.45 (m, 4H, piperidine-H3, H5), 1.46-1.53 (m, 1H, piperidine-H4), 1.59/1.61 (s, 3H, CH=C(C$\underline{H}_3$)$_2$), 1.73/1.74 (s, 3H, CH=C(C$\underline{H}_3$)$_2$), 2.03-2.13 (m, 2H, C$\underline{H}_2$CHOTMP), 3.85-3.90 (m, 1H, C$\underline{H}$OTMS), 3.91/4.00 (dd, *J* = 15.9, 6.1 Hz, 1H, C$\underline{H}_2$CH=), 4.07/4.25 (dd, *J* = 15.9, 6.8 Hz, 1H, C$\underline{H}_2$CH=), 4.27/4.34 (d, *J* = 14.4 Hz/*J* = 16.9 Hz, 1H, C$\underline{H}_2$Ph), 4.63/4.74 (d, *J* = 14.4 Hz/*J* = 16.9 Hz, 1H, C$\underline{H}_2$Ph), 4.70-4.81 (m, 1H, C$\underline{H}$OTMP), 5.15/5.31 (t, *J* = 6.8 Hz, 1H, C$\underline{H}$=C(CH_3)$_2$), 7.19-7.36 (m, 5H, Ar$\underline{H}$); ^{13}C NMR (101 MHz, $CDCl_3$): δ 0.3/0.5 (q, $Si(CH_3)_3$), 17.1 (t, piperidine-C4), 17.8/18.06 (q, CH=C($\underline{C}H_3$)$_2$), 20.2 (q, NC$\underline{C}H_3$), 20.4 (q, NC$\underline{C}H_3$), 23.7/23.9 (q, $\underline{C}H_3$CHOTMS), 25.7/25.8 (q, CH=C($\underline{C}H_3$)$_2$), 33.1 (q, NC$\underline{C}H_3$), 33.7 (q, NC$\underline{C}H_3$), 40.37/40.44 (t, piperidine-C3, C5), 41.9/42.1 (t, $\underline{C}H_2$CHOTMP), 42.9/44.5 (t, $\underline{C}H_2$CH=), 47.8/49.9 (t, $\underline{C}H_2$Ph), 59.4 (s, 2C, CNO), 65.3/65.4 (d, $\underline{C}$HOTMS), 77.3/77.8 (d, $\underline{C}$HOTMP), 119.1/121.0 (d, $\underline{C}$H=C(CH_3)$_2$), 127.0/127.1 (d, CH_{Ar}), 128.2 (d, CH_{Ar}), 128.6/128.8 (d, CH_{Ar}), 135.3/136.1 (s, CH=$\underline{C}$(CH_3)$_2$), 137.4/137.6 (s, C_{Ar}), 172.2/172.4 (s, C=O).

Second diastereomer: ^{1}H NMR (400 MHz, $CDCl_3$): δ 0.06/0.09 (s, 9H, $Si(CH_3)_3$), 0.99 (s, 3H, $NCCH_3$), 1.02 (d, *J* = 6.1 Hz, 3H, C$\underline{H}_3$CHOTMS), 1.04 (s, 3H, $NCCH_3$), 1.08 (s, 3H, $NCCH_3$), 1.10 (s, 3H, $NCCH_3$), 1.24-1.35 (m, 1H, piperidine-H4), 1.36-1.45 (m, 4H, piperidine-H3, H5),

1.46-1.53 (m, 1H, piperidine-H4), 1.50/1.54 (s, 3H, CH=C(C$\underline{H}_3$)$_2$), 1.67 (s, 3H, CH=C(C$\underline{H}_3$)$_2$), 1.94-2.02 (m, 2H, C$\underline{H}_2$CHOTMP), 3.70-3.79 (m, 1H, C$\underline{H}$OTMS), 3.80-4.02 (m, 2H, C$\underline{H}_2$CH=), 4.34/5.27 (d, *J* = 14.2 Hz/*J* = 16.9 Hz, 1H, C$\underline{H}_2$Ph), 4.61-4.69 (m, 1H, C$\underline{H}$OTMP), 4.70/4.78 (d, *J* = 16.9 Hz/*J* = 14.2 Hz, 1H, C$\underline{H}_2$Ph), 5.18/5.40 (t, *J* = 6.8 Hz, 1H, C$\underline{H}$=C(CH$_3$)$_2$), 7.19-7.36 (m, 5H, Ar$\underline{H}$); ^{13}C NMR (101 MHz, CDCl$_3$): δ 0.6/0.7 (q, Si(CH$_3$)$_3$), 17.2 (t, piperidine-C4), 17.8/18.12 (q, CH=C($\underline{C}$H$_3$)$_2$), 20.2 (q, NC$\underline{C}$H$_3$), 20.6 (q, NC$\underline{C}$H$_3$), 24.4/24.8 (q, $\underline{C}$H$_3$CHOTMS), 25.7/25.8 (q, CH=C($\underline{C}$H$_3$)$_2$), 32.9 (q, NC$\underline{C}$H$_3$), 33.6 (q, NC$\underline{C}$H$_3$), 40.5/40.6 (t, piperidine-C3, C5), 42.4/42.7 (t, $\underline{C}$H$_2$CHOTMP), 43.3/44.3 (t, $\underline{C}$H$_2$CH=), 48.3/49.8 (t, $\underline{C}$H$_2$Ph), 59.49/59.53 (s, CNO), 60.3/60.5 (s, CNO), 65.7/65.9 (d, $\underline{C}$HOTMS), 78.8/79.1 (d, $\underline{C}$HOTMP), 119.3/121.1 (d, $\underline{C}$H=C(CH$_3$)$_2$), 127.2/127.3 (d, CH$_{Ar}$), 128.3 (d, CH$_{Ar}$), 128.6/129.2 (d, CH$_{Ar}$), 135.4/136.1 (s, CH=$\underline{C}$(CH$_3$)$_2$), 137.5/137.7 (s, C$_{Ar}$), 172.6/173.1 (s, C=O).

(4*S*)-*N*,*N*-Diallyl-5-(benzyloxy)-2-((2,2,6,6-tetramethylpiperidin-1-yl)oxy)-4-((trimethylsilyl)oxy)pentanamide (9h):

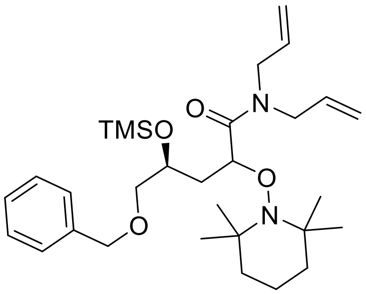

Prepared according to the general procedure, yield 300 mg (65%) as an inseparable 1.1:1 mixture of diastereomers.

[R$_f$ (hexanes/EtOAc 5:1) = 0.62]; IR (film); ν [cm^{-1}]: 3084 (w), 2953 (w), 2862 (w), 1654 (s), 1454 (w), 1413 (m), 1378 (w), 1362 (w), 1251 (w), 1249 (w), 1211 (w), 1132 (m), 1094 (m), 991 (w), 922 (w), 876 (m), 841 (s), 751 (w), 713 (m), 698 (w), 612 (w); MS (+ESI) m/z, (%): 1083 (60, [2M+Na$^+$]), 553 (100, [M+Na$^+$]), 531 (10, [M+H$^+$]); HRMS (+ESI) m/z [C$_{30}$H$_{50}$N$_2$O$_4$SiNa$^+$]: calcd. 553.3613; found 553.3610.

First diastereomer: ^{1}H NMR (400 MHz, CDCl$_3$): δ 0.08 (s, 9H, Si(CH$_3$)$_3$), 1.00 (s, 3H, NCCH$_3$), 1.06 (s, 6H, NCCH$_3$), 1.18 (s, 3H, NCCH$_3$), 1.25-1.33 (m, 1H, piperidine-H4), 1.39-1.47 (m, 4H, piperidine-H3, H5), 1.48-1.56 (m, 1H, piperidine-H4), 2.03-2.11 (m, 2H, C$\underline{H}_2$CHOTMP), 3.30-3.43 (m, 2H, C$\underline{H}_2$OBn), 3.84-3.89 (m, 1H, C$\underline{H}_2$CH=), 3.92-3.96 (m, 1H, C$\underline{H}$OTMS), 4.07 (dd, *J* = 16.5, 5.6 Hz, 1H, C$\underline{H}_2$CH=), 4.31 (dd, *J* = 16.4, 6.1 Hz, 1H, C$\underline{H}_2$CH=), 4.49 (s, 2H, C$\underline{H}_2$Ph),

4.56 (dd, *J* = 16.1, 5.7 Hz, 1H, C$\underline{H}_2$CH=), 4.67-4.71 (m, 1H, C$\underline{H}$OTMP), 5.10-5.20 (m, 4H, CH=C$\underline{H}_2$), 5.82-5.92 (m, 2H, C$\underline{H}$=CH$_2$), 7.24-7.36 (m, 5H, Ar$\underline{H}$); ^{13}C NMR (101 MHz, CDCl$_3$): δ 0.8 (q, Si(CH$_3$)$_3$), 17.3 (t, piperidine-C4), 20.3 (q, NC$\underline{C}$H$_3$), 20.5 (q, NC$\underline{C}$H$_3$), 33.1 (q, NC$\underline{C}$H$_3$), 33.8 (q, NC$\underline{C}$H$_3$), 36.9 (t, $\underline{C}$H$_2$CHOTMP), 40.6 (t, piperidine-C3, C5), 49.18 (t, $\underline{C}$H$_2$CH=), 49.19 (t, $\underline{C}$H$_2$CH=), 59.5 (s, CNO), 60.8 (s, CNO), 68.9 (d, $\underline{C}$HOTMS), 73.4 (t, $\underline{C}$H$_2$Ph), 75.3 (t, $\underline{C}$H$_2$OBn), 79.5 (d, $\underline{C}$HOTMP), 117.6 (t, CH=$\underline{C}$H$_2$), 117.8 (t, CH=$\underline{C}$H$_2$), 127.6 (d, CH$_{Ar}$), 127.83 (d, CH$_{Ar}$), 128.4 (d, CH$_{Ar}$), 134.0 (d, $\underline{C}$H=CH$_2$), 134.3 (d, $\underline{C}$H=CH$_2$), 138.4 (s, C$_{Ar}$), 171.9 (s, C=O).

Second diastereomer: ^{1}H NMR (400 MHz, CDCl$_3$): δ 0.09 (s, 9H, Si(CH$_3$)$_3$), 1.04 (s, 3H, NCCH$_3$), 1.09 (s, 3H, NCCH$_3$), 1.12 (s, 3H, NCCH$_3$), 1.21 (s, 3H, NCCH$_3$), 1.25-1.33 (m, 1H, piperidine-H4), 1.39-1.47 (m, 4H, piperidine-H3, H5), 1.48-1.56 (m, 1H, piperidine-H4), 1.93-2.02 (m, 1H, C$\underline{H}_2$CHOTMP), 2.11-2.18 (m, 1H, C$\underline{H}_2$CHOTMP), 3.30-3.43 (m, 2H, C$\underline{H}_2$OBn), 3.75-3.80 (m, 2H, C$\underline{H}_2$CH=, C$\underline{H}$OTMS), 3.90-4.02 (m, 3H, C$\underline{H}_2$CH=), 4.46 (d, *J* = 12.2 Hz, 1H, C$\underline{H}_2$Ph), 4.51 (d, *J* = 12.2 Hz, 1H, C$\underline{H}_2$Ph), 4.65-4.69 (m, 1H, C$\underline{H}$OTMP), 5.10-5.20 (m, 4H, CH=C$\underline{H}_2$), 5.70-5.81 (m, 2H, C$\underline{H}$=CH$_2$), 7.24-7.36 (m, 5H, Ar$\underline{H}$); ^{13}C NMR (101 MHz, CDCl$_3$): δ 0.7 (q, Si(CH$_3$)$_3$), 17.2 (t, piperidine-C4), 20.3 (q, NC$\underline{C}$H$_3$), 20.5 (q, NC$\underline{C}$H$_3$), 33.1 (q, NC$\underline{C}$H$_3$), 33.8 (q, NC$\underline{C}$H$_3$), 37.9 (t, $\underline{C}$H$_2$CHOTMP), 40.7 (t, piperidine-C3, C5), 47.8 (t, $\underline{C}$H$_2$CH=), 48.3 (t, $\underline{C}$H$_2$CH=), 59.7 (s, CNO), 60.5 (s, CNO), 69.0 (d, $\underline{C}$HOTMS), 73.3 (t, $\underline{C}$H$_2$Ph), 75.1 (t, $\underline{C}$H$_2$OBn), 77.4 (d, $\underline{C}$HOTMP), 118.1 (t, CH=$\underline{C}$H$_2$), 118.3 (t, CH=$\underline{C}$H$_2$), 127.6 (d, CH$_{Ar}$), 127.84 (d, CH$_{Ar}$), 128.4 (d, CH$_{Ar}$), 133.3 (d, 2C, $\underline{C}$H=CH$_2$), 138.5 (s, C$_{Ar}$), 172.7 (s, C=O).

(2*R*,4*S*)- and (2*S*,4*R*)- and (2*S*,4*S*)- and (2*R*,4*R*)-*N*-Allyl-*N*-((*S*)-1-phenylethyl)-2-((2,2,6,6-tetramethylpiperidin-1-yl)oxy)-4-((trimethylsilyl)oxy)pentanamide (9i):

Prepared according to the general procedure from epoxides (*S*)-**7b** and (*R*)-**7b**, yield 312 mg (64%) as an inseparable 3:1 mixture of diastereomers and 307 mg (63%) as an inseparable 8:1 mixture of diastereomers. Ratio of rotamers is 1.5:1 for each diastereomer.

[R$_f$ (hexanes/EtOAc 5:1) = 0.55]; IR (film); ν [cm^{-1}]: 2970 (w), 2931 (w), 1649 (s), 1453 (w), 1416 (w), 1376 (w), 1362 (w), 1250 (m), 1205 (w), 1179 (w), 1132 (w), 1116 (w), 1099 (w), 1061 (w), 1014 (w), 992 (w), 956 (w), 917 (w), 893 (w), 839 (s), 749 (w), 700 (m), 630 (w); MS (+ESI) m/z, (%): 999 (30, [2M+Na$^+$]), 511 (100, [M+Na$^+$]), 489 (40, [M+H$^+$]); HRMS (+ESI) m/z [$C_{28}H_{48}N_2O_3SiNa^+$]: calcd. 511.3326; found 511.3323.

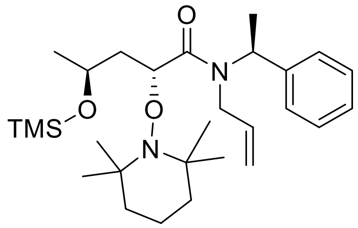

(2*R*,4*S*)-Diastereomer *anti*-**9i**: ^{1}H NMR (400 MHz, $CDCl_3$): δ 0.00/0.10 (s, 9H, $Si(CH_3)_3$), 1.10 (s, 3H, $NCCH_3$), 1.13 (s, 3H, $NCCH_3$), 1.16/1.22 (d, *J* = 6.0 Hz, 3H, $C\underline{H}_3CHOTMS$), 1.17 (s, 6H, $NCCH_3$), 1.19-1.37 (m, 1H, piperidine-H4), 1.38-1.54 (m, 4H, piperidine-H3, H5), 1.49/1.71 (d, *J* = 7.1 Hz, 3H, $PhCHC\underline{H}_3$), 1.51-1.59 (m, 1H, piperidine-H4), 1.92-2.13 (m, 2H, $C\underline{H}_2CHOTMP$), 3.60/3.87 (dd, *J* = 17.6, 5.8 Hz/*J* = 17.0, 5.2 Hz, 1H, $C\underline{H}_2CH=$), 3.70-3.85/3.90-3.98 (m, 1H, $C\underline{H}OTMS$), 4.03/4.64 (dd, *J* = 17.0, 6.7 Hz/*J* = 17.6, 4.6 Hz, 1H, $C\underline{H}_2CH=$), 4.70/4.86 (dd, *J* = 9.8, 3.9 Hz/t, *J* = 6.5 Hz, 1H, $C\underline{H}OTMP$), 4.87-5.13 (m, 2H, $CH=C\underline{H}_2$), 5.76-5.95 (m, 1H, $C\underline{H}=CH_2$), 5.87/5.98 (q, *J* = 7.1 Hz, 1H, $PhC\underline{H}CH_3$), 7.21-7.51 (m, 5H, $Ar\underline{H}$); ^{13}C NMR (101 MHz, $CDCl_3$): δ 0.5/0.8 (q, $Si(CH_3)_3$), 17.1/17.21 (t, piperidine-C4), 17.4/17.6 (q, $PhCH\underline{C}H_3$), 20.2 (q, 2C, $NC\underline{C}H_3$), 23.93/24.2 (q, $\underline{C}H_3CHOTMS$), 33.4 (q, 2C, $NC\underline{C}H_3$), 40.52 (t, piperidine-C3), 40.7 (t, piperidine-C5), 42.6/43.1 (t, $\underline{C}H_2CHOTMP$), 46.10/46.11 (t, $\underline{C}H_2CH=$), 51.8/52.4 (d, $Ph\underline{C}HCH_3$), 59.3/59.4 (s, CNO), 60.5/60.6 (s, CNO), 65.4/65.9 (d, $\underline{C}HOTMS$), 76.4/78.52 (d, $\underline{C}HOTMP$), 116.0/116.38 (t, $CH=\underline{C}H_2$), 126.8/127.39 (d, CH_{Ar}), 128.1/128.34 (d, CH_{Ar}), 128.2/128.59 (d, CH_{Ar}), 136.6/136.9 (d, $\underline{C}H=CH_2$), 140.5/141.1 (s, C_{Ar}), 173.2/173.3 (s, C=O).

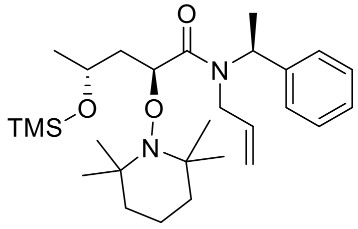

(2*S*,4*R*)-Diastereomer *anti*-**9i**: ^{1}H NMR (400 MHz, $CDCl_3$): δ 0.10/0.11 (s, 9H, $Si(CH_3)_3$), 1.07 (s, 3H, $NCCH_3$), 1.10 (s, 3H, $NCCH_3$), 1.13 (s, 3H, $NCCH_3$), 1.19 (s, 3H, $NCCH_3$), 1.22 (d, *J* = 6.2 Hz, 3H, $C\underline{H}_3CHOTMS$), 1.25-1.31 (m, 1H, piperidine-H4), 1.33-1.46 (m, 5H, piperidine-H3, H4, H5), 1.50/1.72 (d, *J* = 7.1 Hz, 3H, $PhCHC\underline{H}_3$), 1.95-2.05/2.07-2.12 (m, 2H, $C\underline{H}_2CHOTMP$), 3.68/3.83-3.92 (dd, *J* = 17.5, 6.1 Hz/m, 1H, $C\underline{H}_2CH=$), 3.79-3.91 (m, 1H, $C\underline{H}OTMS$), 4.39-4.48 (m, 1H, $C\underline{H}_2CH=$), 4.59/4.66 (dd, *J* = 7.6, 5.9 Hz/*J* = 8.3, 5.0 Hz, 1H, $C\underline{H}OTMP$), 4.80-4.95 (m, 1H, $CH=C\underline{H}_2$), 4.96-5.07 (m, 1H, $CH=C\underline{H}_2$), 5.46/5.62-5.74 (dddd, *J* = 16.9, 11.1, 6.4, 4.8 Hz/m,

1H, $C\underline{H}=CH_2$), 5.89/5.97 (q, J = 6.7 Hz/J = 7.0 Hz, 1H, $PhC\underline{H}CH_3$), 7.22-7.40 (m, 5H, $Ar\underline{H}$); ^{13}C NMR (101 MHz, $CDCl_3$): δ 0.5/0.86 (q, $Si(CH_3)_3$), 16.9/17.4 (q, $PhCH\underline{C}H_3$), 17.19/17.31 (t, piperidine-C4), 20.2 (q, $NC\underline{C}H_3$), 20.41 (q, $NC\underline{C}H_3$), 24.2/24.8 (q, $\underline{C}H_3CHOTMS$), 33.4 (q, $NC\underline{C}H_3$), 33.6 (q, $NC\underline{C}H_3$), 40.3/40.4 (t, piperidine-C3), 40.5/40.6 (t, piperidine-C5), 42.3/43.2 (t, $\underline{C}H_2CHOTMP$), 45.7/46.0 (t, $\underline{C}H_2CH=$), 52.0/52.1 (d, $Ph\underline{C}HCH_3$), 59.3 (s, CNO), 60.3 (s, CNO), 65.2/66.16 (d, $\underline{C}HOTMS$), 77.7/78.50 (d, $\underline{C}HOTMP$), 116.08/116.13 (t, $CH=\underline{C}H_2$), 127.0/127.44 (d, CH_{Ar}), 128.2/128.33 (d, CH_{Ar}), 128.35/128.5 (d, CH_{Ar}), 136.0/136.9 (d, $\underline{C}H=CH_2$), 140.5/140.9 (s, C_{Ar}), 172.7/173.5 (s, C=O).

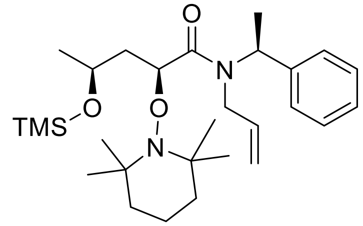

(2*S*,4*S*)-Diastereomer *syn*-**9i**: ^1H NMR (400 MHz, $CDCl_3$): δ 0.05/0.11 (s, 9H, $Si(CH_3)_3$), 1.04 (s, 3H, $NCCH_3$), 1.05 (s, 3H, $NCCH_3$), 1.16 (s, 3H, $NCCH_3$), 1.19 (s, 3H, $NCCH_3$), 1.20-1.37 (m, 1H, piperidine-H4), 1.22/1.24 (d, J = 6.2 Hz, 3H, $C\underline{H}_3CHOTMS$), 1.38-1.54 (m, 4H, piperidine-H3, H5), 1.47/1.63 (d, J = 7.1 Hz, 3H, $PhCHC\underline{H}_3$), 1.51-1.59 (m, 1H, piperidine-H4), 1.83-2.27 (m, 2H, $C\underline{H}_2CHOTMP$), 3.86 (dd, J = 13.0, 4.6 Hz, 1H, $C\underline{H}_2CH=$), 3.88-3.96 (m, 1H, $C\underline{H}OTMS$), 4.61 (dd, J = 13.0, 4.6 Hz, 1H, $C\underline{H}_2CH=$), 4.64/4.93 (t, J = 6.6 Hz, 1H, $C\underline{H}OTMP$), 4.81-5.13 (m, 2H, $CH=C\underline{H}_2$), 5.83-5.93 (m, 2H, $C\underline{H}=CH_2$, $PhC\underline{H}CH_3$), 7.22-7.40 (m, 5H, $Ar\underline{H}$); ^{13}C NMR (101 MHz, $CDCl_3$): δ 0.6/0.91 (q, $Si(CH_3)_3$), 17.24/17.29 (t, piperidine-C4), 19.3/19.5 (q, $PhCH\underline{C}H_3$), 20.42 (q, $NC\underline{C}H_3$), 20.5 (q, $NC\underline{C}H_3$), 23.91/24.8 (q, $\underline{C}H_3CHOTMS$), 33.2 (q, $NC\underline{C}H_3$), 33.6 (q, $NC\underline{C}H_3$), 40.46 (t, piperidine-C3), 40.8 (t, piperidine-C5), 41.0/42.4 (t, $\underline{C}H_2CHOTMP$), 45.9/46.0 (t, $\underline{C}H_2CH=$), 54.8/55.2 (d, $Ph\underline{C}HCH_3$), 59.5 (s, CNO), 60.7 (s, CNO), 65.5/66.22 (d, $\underline{C}HOTMS$), 77.5/79.2 (d, $\underline{C}HOTMP$), 116.12/116.43 (t, $CH=\underline{C}H_2$), 127.42/127.7 (d, CH_{Ar}), 128.0/128.32 (d, CH_{Ar}), 128.4/128.63 (d, CH_{Ar}), 135.0/135.2 (d, $\underline{C}H=CH_2$), 140.8/141.8 (s, C_{Ar}), 171.0/172.6 (s, C=O).

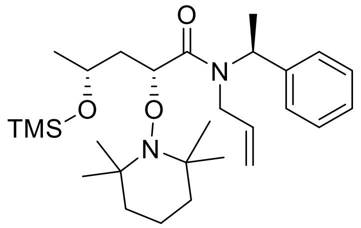

(2*R*,4*R*)-Diastereomer *syn*-**9i**: ^{1}H NMR (400 MHz, $CDCl_3$): δ 0.02/0.15 (s, 9H, $Si(CH_3)_3$), 1.05 (s, 3H, $NCCH_3$), 1.06 (s, 3H, $NCCH_3$), 1.18 (s, 3H, $NCCH_3$), 1.21 (s, 3H, $NCCH_3$), 1.22-1.34 (m, 1H, piperidine-H4), 1.25 (d, *J* = 6.1 Hz, 3H, $C\underline{H}_3CHOTMS$), 1.38-1.54 (m, 4H, piperidine-H3, H5), 1.55-1.59 (m, 1H, piperidine-H4), 1.61 (d, *J* = 7.0 Hz, 3H, $PhCHC\underline{H}_3$), 1.87 (dt, *J* = 13.8, 6.7 Hz, 1H, $C\underline{H}_2CHOTMP$), 2.11-2.23 (m, 1H, $C\underline{H}_2CHOTMP$), 3.37/3.46 (dd, *J* = 15.0, 6.7 Hz/ *J* = 15.3, 6.4 Hz, 1H, $C\underline{H}_2CH=$), 3.77-3.85 (m, 1H, $C\underline{H}OTMS$), 4.02-4.13 (m, 1H, $C\underline{H}_2CH=$), 4.83-4.90 (m, 2H, $C\underline{H}OTMP$, $CH=C\underline{H}_2$), 4.93-4.98 (m, 1H, $CH=C\underline{H}_2$), 5.63-5.81 (m, 2H, $PhC\underline{H}CH_3$, $C\underline{H}=CH_2$), 7.22-7.40 (m, 5H, Ar$\underline{H}$); ^{13}C NMR (101 MHz, $CDCl_3$): δ 0.7/1.5 (q, $Si(CH_3)_3$), 17.1 (t, piperidine-C4), 19.0/19.4 (q, $PhCH\underline{C}H_3$), 20.3 (q, $NC\underline{C}H_3$), 20.6 (q, $NC\underline{C}H_3$), 24.4/24.7 (q, $\underline{C}H_3CHOTMS$), 33.1 (q, $NC\underline{C}H_3$), 33.4 (q, $NC\underline{C}H_3$), 40.96 (t, piperidine-C3), 40.97 (t, piperidine-C5), 41.4/42.7 (t, $\underline{C}H_2CHOTMP$), 45.8/46.1 (t, $\underline{C}H_2CH=$), 55.0 (d, $Ph\underline{C}HCH_3$), 59.5 (s, CNO), 60.8 (s, CNO), 65.8/66.4 (d, $\underline{C}HOTMS$), 78.2/79.3 (d, $\underline{C}HOTMP$), 115.9/116.5 (t, $CH=\underline{C}H_2$), 126.7 (d, CH_{Ar}), 127.1/128.2 (d, CH_{Ar}), 128.6/128.8 (d, CH_{Ar}), 134.9/136.3 (d, $\underline{C}H=CH_2$), 140.5/141.4 (s, C_{Ar}), 171.1/171.9 (s, C=O).

(2*R*,4*S*)- and (2*S*,4*S*)-*N*-Allyl-*N*-((*S*)-1-(naphthalen-2-yl)ethyl)-2-((2,2,6,6-tetramethyl-piperidin-1-yl)oxy)-4-((trimethylsilyl)oxy)pentanamide (9j):

Prepared according to the general procedure, yield 334 mg (62%) as an inseparable 3:1 mixture of diastereomers. Ratio of rotamers is 1.2:1 for each diastereomer. The silyl group in **9j** was deprotected (see p. S35) and hydroxy amide **S13** was transformed to hydrochloride **S13·HCl** (see p. S105). The major diastereomer of **S13·HCl** crystallized from MTBE with a few drops of $CHCl_3$ and its configuration was determined by X-ray crystallography.

[R_f (hexanes/EtOAc 5:1) = 0.60]; IR (film); ν [cm^{-1}]: 2970 (m), 2931 (m), 1647 (s), 1457 (m), 1438 (m), 1417 (m), 1376 (m), 1362 (m), 1249 (s), 1183 (m), 1131 (m), 1098 (w), 1060 (w), 1018 (w), 992 (w), 976 (w), 956 (w), 917 (w), 894 (w), 840 (s), 822 (w), 749 (m), 699 (w); MS (+ESI) m/z, (%): 561 (50, [M+Na$^+$]), 539 (100, [M+H$^+$]); HRMS (+ESI) m/z [$C_{32}H_{51}N_2O_3Si^+$]: calcd. 539.3664; found 539.3661.

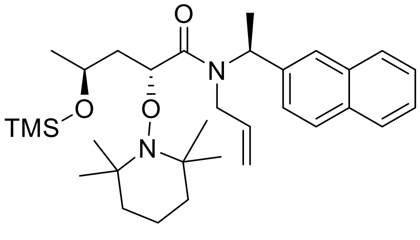

(2*R*,4*S*)

Major diastereomer *anti*-**9j**: ^{1}H NMR (400 MHz, $CDCl_3$): δ –0.03/0.09 (s, 9H, $Si(CH_3)_3$), 0.94 (s, 3H, $NCCH_3$), 1.11 (s, 3H, $NCCH_3$), 1.14 (s, 3H, $NCCH_3$), 1.17 (s, 3H, $NCCH_3$), 1.21/1.29 (d, *J* = 6.2 Hz, 3H, $C\underline{H}_3$CHOTMS), 1.28-1.40 (m, 1H, piperidine-H4), 1.41-1.55 (m, 4H, piperidine-H3, H5), 1.57-1.60 (m, 1H, piperidine-H4), 1.61 (d, *J* = 7.1 Hz, 3H, $C\underline{H}_3$CHAr), 1.96-2.18 (m, 2H, $C\underline{H}_2$CHOTMP), 3.61/3.73 (dd, *J* = 15.3, 6.0 Hz/*J* = 17.6, 6.4 Hz, 1H, $C\underline{H}_2$CH=), 3.78-3.86/3.91-4.02 (m, 1H, $C\underline{H}$OTMS), 4.05/4.45 (dd, *J* = 17.6, 5.7 Hz/*J* = 15.3, 5.4 Hz, 1H, $C\underline{H}_2$CH=), 4.60-4.68/4.73 (m/dd, *J* = 9.8, 3.9 Hz, 1H, $C\underline{H}$OTMP), 4.87-5.17 (m, 2H, $CH=C\underline{H}_2$), 5.71-6.02 (m, 1H, $C\underline{H}=CH_2$), 6.07/6.15 (q, *J* = 7.2 Hz, 1H, $ArC\underline{H}CH_3$), 7.41-7.58 (m, 2H, Ar$\underline{H}$), 7.70-7.94 (m, 5H, Ar$\underline{H}$); ^{13}C NMR (101 MHz, $CDCl_3$): δ 0.6/0.8 (q, $Si(CH_3)_3$), 17.1/17.26 (t, piperidine-C4), 17.5/17.6 (q, $\underline{C}H_3$CHAr), 20.2 (q, $NC\underline{C}H_3$), 20.44 (q, $NC\underline{C}H_3$), 24.0/24.2 (q, $\underline{C}H_3$CHOTMS), 33.5 (q, $NC\underline{C}H_3$), 33.6 (q, $NC\underline{C}H_3$), 40.5 (t, piperidine-C3), 40.7 (t, piperidine-C5), 42.7/43.2 (t, $\underline{C}H_2$CHOTMP), 46.19/46.23 (t, $\underline{C}H_2$CH=), 51.9/52.4 (d, $Ar\underline{C}HCH_3$), 59.4/59.5 (s, CNO), 60.62 (s, CNO), 65.4/65.9 (d, $\underline{C}$HOTMS), 77.6/78.6 (d, $\underline{C}$HOTMP), 116.2/116.5 (t, $CH=\underline{C}H_2$), 125.4 (d, CH_{Ar}), 126.0 (d, CH_{Ar}), 126.3 (d, CH_{Ar}), 126.82 (d, CH_{Ar}), 127.34 (d, CH_{Ar}), 127.6 (d, CH_{Ar}), 127.8 (d, CH_{Ar}), 132.3 (s, C_{Ar}), 133.2/133.3 (s, C_{Ar}), 136.6/137.0 (d, $\underline{C}H=CH_2$), 138.2/138.7 (s, C_{Ar}), 173.4/173.5 (s, C=O).

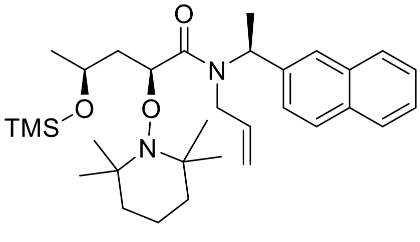

(2*S*,4*S*)

Minor diastereomer *syn*-**9j**: ^{1}H NMR (400 MHz, $CDCl_3$): δ 0.00/0.13 (s, 9H, $Si(CH_3)_3$), 1.00 (s, 3H, $NCCH_3$), 1.08 (s, 3H, $NCCH_3$), 1.14 (s, 6H, $NCCH_3$), 1.25/1.31 (d, *J* = 6.0 Hz, 3H, $C\underline{H}_3$CHOTMS), 1.28-1.40 (m, 1H, piperidine-H4), 1.41-1.55 (m, 4H, piperidine-H3, H5), 1.56-1.62 (m, 1H, piperidine-H4), 1.85/1.87 (d, *J* = 6.4 Hz, 3H, $C\underline{H}_3$CHAr), 1.99-2.21 (m, 2H,

C<u>H</u>$_2$CHOTMP), 3.40/3.64 (dd, *J* = 15.6, 5.4 Hz/*J* = 17.3, 4.9 Hz, 1H, C<u>H</u>$_2$CH=), 3.48-3.56/4.05-4.12 (m, 1H, C<u>H</u>$_2$CH=), 3.92-4.00 (m, 1H, C<u>H</u>OTMS), 4.64-4.86 (m, 1H, C<u>H</u>OTMP), 4.87-5.17 (m, 2H, CH=C<u>H</u>$_2$), 5.45-5.56 (m, 1H, C<u>H</u>=CH$_2$), 6.13-6.26 (m, 1H, ArC<u>H</u>CH$_3$), 7.41-7.58 (m, 2H, Ar<u>H</u>), 7.70-7.94 (m, 5H, Ar<u>H</u>); ^{13}C NMR (101 MHz, CDCl$_3$): δ 0.9/1.5 (q, Si(CH$_3$)$_3$), 17.2/17.29 (t, piperidine-C4), 19.3/19.5 (q, <u>C</u>H$_3$CHAr), 20.38 (q, NC<u>C</u>H$_3$), 20.6 (q, NC<u>C</u>H$_3$), 23.9/24.4 (q, <u>C</u>H$_3$CHOTMS), 33.3 (q, NC<u>C</u>H$_3$), 33.7 (q, NC<u>C</u>H$_3$), 40.4 (t, piperidine-C3), 41.0 (t, piperidine-C5), 41.5/42.5 (t, <u>C</u>H$_2$CHOTMP), 46.06/46.12 (t, <u>C</u>H$_2$CH=), 54.9/55.4 (d, Ar<u>C</u>HCH$_3$), 59.7 (s, CNO), 60.63 (s, CNO), 65.3/65.6 (d, <u>C</u>HOTMS), 76.5/79.3 (d, <u>C</u>HOTMP), 116.6/116.8 (t, CH=<u>C</u>H$_2$), 124.6 (d, CH$_{Ar}$), 125.5 (d, CH$_{Ar}$), 126.1 (d, CH$_{Ar}$), 126.7 (d, CH$_{Ar}$), 126.80 (d, CH$_{Ar}$), 127.29 (d, CH$_{Ar}$), 127.9 (d, CH$_{Ar}$), 132.93 (s, C$_{Ar}$), 133.4 (s, C$_{Ar}$), 135.1/136.0 (d, <u>C</u>H=CH$_2$), 138.0/138.4 (s, C$_{Ar}$), 172.8/175.0 (s, C=O).

(2*R*,4*S*)- and (2*S*,4*S*)-*N*-Allyl-4-hydroxy-*N*-((*S*)-1-(naphthalen-2-yl)ethyl)-2-((2,2,6,6-tetra-methylpiperidin-1-yl)oxy)pentanamide (S13):

In a similar manner as described in [11] amide **9j** (619 mg, 1.15 mmol) was dissolved in dry THF (5 mL). The reaction mixture was cooled to 0 °C in an ice/water bath, tetrabutylammonium fluoride (1 M solution in THF, 1.4 mL, 1.4 mmol) was added and the mixture was stirred at 0 °C for 30 min. The reaction was quenched by saturated NH_4Cl solution and diluted with water (5 mL) and diethyl ether (5 mL), the organic layer was separated and the aqueous was extracted with diethyl ether (2 × 5 mL). The combined organic layers were dried over $MgSO_4$ and filtered. The crude mixture was purified by column chromatography (gradient, hexanes/EtOAc 10:1 to 1:1) to give 493 mg (92%) **S13** as an inseparable 3:1 mixture of diastereomers.

[R_f (hexanes/EtOAc 2:1) = 0.36]; IR (film); ν [cm^{-1}]: 3411 (br), 2970 (m), 2928 (s), 2873 (w), 1629 (s), 1455 (w), 1417 (w), 1377 (w), 1362 (w), 1321 (w), 1298 (w), 1258 (w), 1242 (w), 1131 (m), 1081 (w), 990 (w), 920 (w), 858 (w), 820 (w), 749 (w); MS (+ESI) m/z, (%): 955 (10, [2M+Na$^+$], 489 (100, [M+Na$^+$]); HRMS (+ESI) m/z [$C_{29}H_{42}N_2O_3Na^+$]: calcd. 489.3088; found 489.3083.

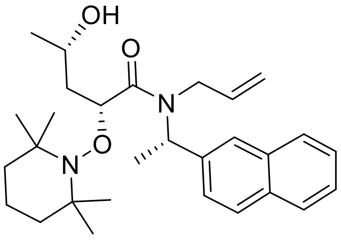

(2*R*,4*S*)

Major diastereomer: ^{1}H NMR (400 MHz, $CDCl_3$): δ 1.15 (s, 6H, $NCCH_3$), 1.16 (s, 3H, $NCCH_3$), 1.18 (s, 3H, $NCCH_3$), 1.24 (d, *J* = 6.2 Hz, 3H, $C\underline{H}_3CHOH$), 1.31-1.40 (m, 1H, piperidine-H4), 1.41-1.54 (m, 4H, piperidine-H3, H5), 1.55-1.59 (m, 1H, piperidine-H4), 1.60/1.61 (d, *J* = 7.1 Hz, 3H, $ArCHC\underline{H}_3$), 2.08-2.18 (m, 3H, OH, $C\underline{H}_2CHOTMP$), 3.85 (dd, *J* = 17.4, 4.6 Hz, 1H, $C\underline{H}_2CH=$), 4.07-4.22 (m, 2H, $C\underline{H}OH$, $C\underline{H}_2CH=$), 4.70/4.76 (dd, *J* = 7.2, 4.5 Hz/*J* = 8.6, 3.2 Hz, 1H, $C\underline{H}OTMP$), 4.85-4.95/5.11-5.17 (m, 2H, $CH=C\underline{H}_2$), 5.47/5.76-5.88 (dddd, *J* = 17.2, 10.6, 7.0, 4.5 Hz/m, 1H, $C\underline{H}=CH_2$), 6.17/6.18 (q, *J* = 7.1 Hz/*J* = 6.8 Hz, 1H, $ArC\underline{H}CH_3$), 7.35-7.53 (m, 3H, $Ar\underline{H}$), 7.62-7.86 (m, 4H, $Ar\underline{H}$); ^{13}C NMR (101 MHz, $CDCl_3$): δ 16.0/17.37 (q, $ArCH\underline{C}H_3$), 17.1/17.2 (t, piperidine-C4), 20.3/20.4 (q, $NC\underline{C}H_3$), 20.5/20.6 (q, $NC\underline{C}H_3$), 24.39/24.42 (q, $\underline{C}H_3CHOH$), 33.5 (q, 2C, $NC\underline{C}H_3$), 40.50 (t, piperidine-C3), 40.53 (t, piperidine-C5), 41.0/41.6 (t, $\underline{C}H_2CHOTMP$), 46.0/46.1 (t, $\underline{C}H_2CH=$), 51.9/52.1 (d, $Ar\underline{C}HCH_3$), 59.7 (s, CNO), 60.6 (s, CNO), 63.7/65.3 (d, CHOH), 79.3/80.1 (d, $\underline{C}HOTMP$), 116.6/116.8 (t, $CH=\underline{C}H_2$), 126.2 (d, CH_{Ar}), 126.34 (d, CH_{Ar}), 126.7 (d, CH_{Ar}), 127.10 (d, CH_{Ar}), 127.73 (d, CH_{Ar}), 128.12 (d, CH_{Ar}), 128.3 (d, CH_{Ar}), 132.92/132.94 (s, C_{Ar}), 133.26/133.28 (s, C_{Ar}), 136.0/136.4 (d, $\underline{C}H=CH_2$), 138.0/138.1 (s, C_{Ar}), 173.7/175.0 (s, C=O).

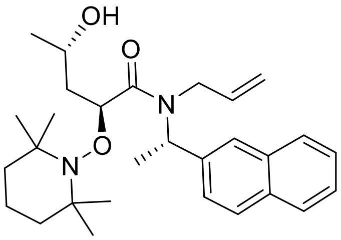

(2*S*,4*S*)

Minor diastereomer: ^{1}H NMR (400 MHz, $CDCl_3$): δ 1.08 (s, 3H, $NCCH_3$), 1.13 (s, 3H, $NCCH_3$), 1.16 (s, 3H, $NCCH_3$), 1.19 (s, 3H, $NCCH_3$), 1.25 (d, *J* = 6.1 Hz, 3H, $C\underline{H}_3CHOH$), 1.31-1.40 (m, 1H, piperidine-H4), 1.41-1.54 (m, 4H, piperidine-H3, H5), 1.55-1.59 (m, 1H, piperidine-H4), 1.79/1.83 (d, *J* = 6.8 Hz, 3H, $ArCHC\underline{H}_3$), 1.96-2.06 (m, 3H, OH, $C\underline{H}_2CHOTMP$), 3.62 (dd, *J* = 17.9, 5.0 Hz, 1H, $C\underline{H}_2CH=$), 3.97-4.06 (m, 1H, $C\underline{H}OH$), 4.40 (dd, *J* = 17.9, 5.2 Hz, 1H,

$C\underline{H}_2CH$=), 4.86-4.95 (m, 1H, $C\underline{H}OTMP$), 4.96-5.06/5.07-5.11 (m, 2H, CH=$C\underline{H}_2$), 5.67-5.75/5.76-5.88 (m, 1H, $C\underline{H}$=CH_2), 6.14-6.23 (m, 1H, $ArC\underline{H}CH_3$), 7.35-7.53 (m, 3H, $Ar\underline{H}$), 7.62-7.86 (m, 4H, $Ar\underline{H}$); ^{13}C NMR (101 MHz, $CDCl_3$): δ 17.3/17.44 (t, piperidine-C4), 19.4/19.5 (q, $ArCH\underline{C}H_3$), 20.3/20.4 (q, $NC\underline{C}H_3$), 20.5/20.6 (q, $NC\underline{C}H_3$), 24.0/24.1 (q, $\underline{C}H_3CHOH$), 33.6 (q, $NC\underline{C}H_3$), 33.7 (q, $NC\underline{C}H_3$), 40.0/41.3 (t, $\underline{C}H_2CHOTMP$), 40.4 (t, piperidine-C3), 40.6 (t, piperidine-C5), 46.2/46.3 (t, $\underline{C}H_2CH$=), 55.5/55.6 (d, $Ar\underline{C}HCH_3$), 59.8 (s, CNO), 60.7 (s, CNO), 64.6/65.7 (d, CHOH), 81.0/81.8 (d, $\underline{C}HOTMP$), 116.7/116.9 (t, CH=$\underline{C}H_2$), 125.2 (d, CH_{Ar}), 126.1 (d, CH_{Ar}), 126.29 (d, CH_{Ar}), 127.06 (d, CH_{Ar}), 127.68 (d, CH_{Ar}), 128.07 (d, CH_{Ar}), 128.7 (d, CH_{Ar}), 132.97/133.00 (s, C_{Ar}), 133.58/133.61 (s, C_{Ar}), 134.6/134.7 (d, $\underline{C}H$=CH_2), 138.2/138.3 (s, C_{Ar}), 174.3/174.6 (s, C=O).

(2*R*,4*S*) and (2*S*,4*S*)-*N*-(3-Methylbut-2-en-1-yl)-*N*-((*S*)-1-phenylethyl)-2-((2,2,6,6-tetra-methylpiperidin-1-yl)oxy)-4-((trimethylsilyl)oxy)pentanamide (9k):

Prepared according to the general procedure, yield 315 mg (61%) as an inseparable 3:1 mixture of diastereomers. Ratio of rotamers is 1.2:1 for each diastereomer.

[R_f (hexanes/EtOAc 5:1) = 0.60]; IR (film); ν [cm^{-1}]: 2968 (m), 2930 (m), 1648 (s), 1495 (m), 1439 (m), 1376 (w), 1363 (w), 1309 (w), 1250 (s), 1208 (w), 1167 (m), 1133 (m), 1098 (w), 1064 (w), 1017 (w), 993 (w), 957 (w), 896 (w), 840 (s), 784 (w), 750 (w), 700 (m), 600 (w); MS (+ESI) m/z, (%): 539 (100, [$M+Na^+$]), 517 (90, [$M+H^+$]); HRMS (+ESI) m/z [$C_{30}H_{52}N_2O_3SiNa^+$]: calcd. 539.3639; found 539.3638.

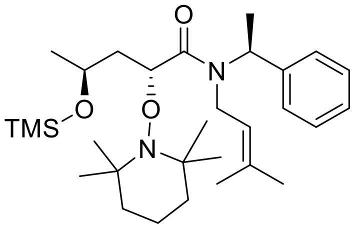

(2*R*,4*S*)

Major diastereomer *anti*-**9k**: ^1H NMR (400 MHz, $CDCl_3$): δ –0.03/0.11 (s, 9H, $Si(CH_3)_3$), 0.96/1.15 (d, *J* = 6.1 Hz, 3H, $C\underline{H}_3CHOTMS$), 1.04 (s, 3H, $NCCH_3$), 1.10 (s, 3H, $NCCH_3$), 1.14 (s, 6H, $NCCH_3$), 1.25-1.36 (m, 1H, piperidine-H4), 1.37-1.51 (m, 4H, piperidine-H3, H5), 1.48/1.49 (d, *J* = 7.1 Hz, 3H, $PhCHC\underline{H}_3$), 1.50 (s, 3H, CH=$C(C\underline{H}_3)_2$), 1.52-1.57 (m, 1H, piperidine-H4), 1.53/1.67 (s, 3H, CH=$C(C\underline{H}_3)_2$), 1.91-2.15 (m, 2H, $C\underline{H}_2CHOTMP$), 3.61 (dd, *J* = 16.1, 4.0 Hz, 1H, $C\underline{H}_2CH$=), 3.72-3.79/3.80-3.82 (m, 1H, $C\underline{H}OTMS$), 3.83-3.90 (m, 1H, $C\underline{H}_2CH$=), 4.56/4.71 (dd, *J* = 8.6, 5.1 Hz/*J* = 9.8, 3.6 Hz, 1H, $C\underline{H}OTMP$), 5.10-5.20/5.48-5.55

(m, 1H, $C\underline{H}=C(CH_3)_2$), 5.87/6.02 (q, *J* = 7.0 Hz, 1H, $PhC\underline{H}CH_3$), 7.20-7.41 (m, 5H, $Ar\underline{H}$); ^{13}C NMR (101 MHz, $CDCl_3$): δ 0.4/0.8 (q, $Si(CH_3)_3$), 16.1/17.0 (q, $PhCH\underline{C}H_3$), 17.1/17.24 (t, piperidine-C4), 18.1/18.3 (q, $CH=C(\underline{C}H_3)_2$), 20.1 (q, $NC\underline{C}H_3$), 20.4 (q, $NC\underline{C}H_3$), 23.9/25.6 (q, $\underline{C}H_3CHOTMS$), 25.47/25.53 (q, $CH=C(\underline{C}H_3)_2$), 33.3 (q, $NC\underline{C}H_3$), 33.4 (q, $NC\underline{C}H_3$), 40.46/40.70 (t, piperidine-C3), 40.50 (t, piperidine-C5), 41.6/41.8 (t, $\underline{C}H_2CH=$), 42.7/43.1 (t, $\underline{C}H_2CHOTMP$), 51.3/52.0 (d, $Ph\underline{C}HCH_3$), 59.3 (s, CNO), 60.49 (s, CNO), 65.3/65.9 (d, $\underline{C}HOTMS$), 78.0/78.6 (d, $\underline{C}HOTMP$), 124.0/124.3 (d, $\underline{C}H=C(CH_3)_2$), 126.7/127.3 (d, CH_{Ar}), 127.7/128.1 (d, CH_{Ar}), 128.4/128.5 (d, CH_{Ar}), 132.0/132.6 (s, $CH=\underline{C}(CH_3)_2$), 140.6/141.5 (s, C_{Ar}), 173.0/173.2 (s, C=O).

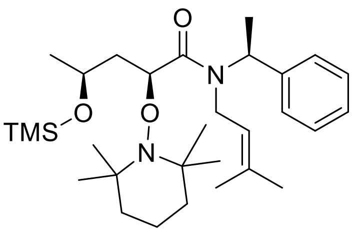

(2*S*,4*S*)

Minor diastereomer *syn*-**9k**: ^1H NMR (400 MHz, $CDCl_3$): δ 0.04/0.16 (s, 9H, $Si(CH_3)_3$), 0.89 (s, 3H, $NCCH_3$), 1.04 (s, 3H, $NCCH_3$), 1.08 (s, 3H, $NCCH_3$), 1.12 (s, 3H, $NCCH_3$), 1.22/1.23 (d, *J* = 6.1 Hz, 3H, $C\underline{H}_3CHOTMS$), 1.25-1.36 (m, 1H, piperidine-H4), 1.37-1.51 (m, 4H, piperidine-H3, H5), 1.44/1.52 (d, *J* = 7.2 Hz, 3H, $PhCHC\underline{H}_3$), 1.45/1.61 (s, 3H, $CH=C(C\underline{H}_3)_2$), 1.53-1.57 (m, 1H, piperidine-H4), 1.65 (s, 3H, $CH=C(C\underline{H}_3)_2$), 1.85-2.30 (m, 2H, $C\underline{H}_2CHOTMP$), 3.96-4.31 (m, 2H, $C\underline{H}_2CH=$), 4.67-4.73 (m, 1H, $C\underline{H}OTMP$), 5.51 (dd, *J* = 6.7, 5.2 Hz, 1H, $C\underline{H}=C(CH_3)_2$), 5.87 (q, *J* = 7.3 Hz, 1H, $PhC\underline{H}CH_3$), 7.20-7.41 (m, 5H, $Ar\underline{H}$); ^{13}C NMR (101 MHz, $CDCl_3$): δ 0.5/1.4 (q, $Si(CH_3)_3$), 15.3/15.9 (q, $PhCH\underline{C}H_3$), 17.18/17.3 (t, piperidine-C4), 17.6/17.7 (q, $CH=C(\underline{C}H_3)_2$), 20.2 (q, $NC\underline{C}H_3$), 20.5 (q, $NC\underline{C}H_3$), 24.3/24.9 (q, $\underline{C}H_3CHOTMS$), 24.8/25.7 (q, $CH=C(\underline{C}H_3)_2$), 33.1 (q, $NC\underline{C}H_3$), 33.5 (q, $NC\underline{C}H_3$), 40.4 (t, piperidine-C3), 40.72 (t, piperidine-C5), 40.9/41.3 (t, $\underline{C}H_2CH=$), 41.49/41.53 (t, $\underline{C}H_2CHOTMP$), 54.5/54.9 (d, $Ph\underline{C}HCH_3$), 59.2 (s, CNO), 60.53 (s, CNO), 65.5/66.2 (d, $\underline{C}HOTMS$), 76.6/79.5 (d, $\underline{C}HOTMP$), 122.3/123.2 (d, $\underline{C}H=C(CH_3)_2$), 127.2 (d, CH_{Ar}), 127.4 (d, CH_{Ar}), 128.6 (d, CH_{Ar}), 132.5/133.2 (s, $CH=\underline{C}(CH_3)_2$), 140.7/142.1 (s, C_{Ar}), 172.6/174.8 (s, C=O).

(*R)- and (*S**)-*N*,*N*-Diallyl-2-((2,2,6,6-tetramethylpiperidin-1-yl)oxy)-2-((1*R**,2*S**)-2-((trimethylsilyl)oxy)cyclohexyl)acetamide (9l):**

Prepared according to the general procedure, yield 292 mg (63%) as an inseparable 7:1 mixture of diastereomers.

[R_f (hexanes/EtOAc 10:1) = 0.63]; IR (film); ν [cm^{-1}]: 3006 (w), 2930 (m), 2857 (w), 1654 (s), 1447 (w), 1414 (w), 1378 (w), 1362 (w), 1250 (m), 1222 (w), 1202 (w), 1183 (w), 1133 (m), 1089 (w), 1058 (w), 989 (w), 941 (w), 920 (w), 881 (s), 839 (s), 792 (w), 709 (w), 689 (w); MS (+ESI) m/z, (%): 487 (15, [$M+Na^+$]), 465 (100, [$M+H^+$]); HRMS (+ESI) m/z [$C_{26}H_{49}N_2O_3Si^+$]: calcd. 465.3507; found 465.3508.

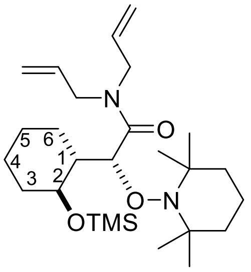

(*R**)

Major diastereomer: ^{1}H NMR (400 MHz, $CDCl_3$): δ 0.09 (s, 9H, $Si(CH_3)_3$), 1.04 (s, 3H, $NCCH_3$), 1.06-1.32 (m, 4H, H3, H4, H5, piperidine-H4), 1.09 (s, 3H, $NCCH_3$), 1.16 (s, 3H, $NCCH_3$), 1.19 (s, 3H, $NCCH_3$), 1.36-1.46 (m, 4H, piperidine-H3, H5), 1.47-1.51 (m, 2H, H1, H6), 1.52-1.57 (m, 1H, piperidine-H4), 1.60-1.74 (m, 2H, H4, H5), 1.95-2.04 (m, 1H, H3), 2.51-2.59 (m, 1H, H6), 3.73-3.80 (m, 2H, H2, $C\underline{H}_2CH=$), 3.86 (dd, *J* = 16.9, 5.9 Hz, 1H, $C\underline{H}_2CH=$), 4.01 (dd, *J* = 14.7, 6.1 Hz, 1H, $C\underline{H}_2CH=$), 4.53 (dd, *J* = 16.9, 5.2 Hz, 1H, $C\underline{H}_2CH=$), 5.06 (d, *J* = 3.3 Hz, 1H, $C\underline{H}OTMP$), 5.10-5.13 (m, 1H, $CH=C\underline{H}_2$), 5.14-5.16 (m, 2H, $CH=C\underline{H}_2$), 5.17-5.20 (m, 1H, $CH=C\underline{H}_2$), 5.72-5.83 (m, 1H, $C\underline{H}=CH_2$), 5.90 (dddd, *J* = 17.5, 9.9, 5.9, 5.2 Hz, 1H, $C\underline{H}=CH_2$); ^{13}C NMR (101 MHz, $CDCl_3$): δ 1.2 (q, $Si(CH_3)_3$), 17.1 (t, piperidine-C4), 20.4 (q, $NC\underline{C}H_3$), 20.6 (q, $NC\underline{C}H_3$), 24.3 (t, C6), 25.0 (t, C4), 25.8 (t, C5), 33.4 (q, $NC\underline{C}H_3$), 33.9 (q, $NC\underline{C}H_3$), 36.8 (t, C3), 40.9 (t, piperidine-C3, C5), 47.8 (t, $\underline{C}H_2CH=$), 49.1 (t, $\underline{C}H_2CH=$), 51.2 (d, C1), 59.7 (s, CNO), 60.9 (s, CNO), 72.8 (d, C2), 74.8 (d, $\underline{C}HOTMP$), 117.3 (t, $CH=\underline{C}H_2$), 117.7 (t, $CH=\underline{C}H_2$), 133.6 (d, $\underline{C}H=CH_2$), 134.1 (d, $\underline{C}H=CH_2$), 173.8 (s, C=O).

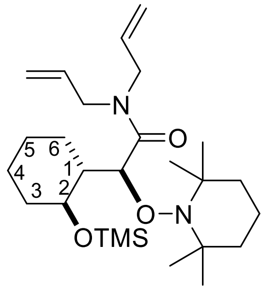

(*S**)

Minor diastereomer (detectable resonances): ^{1}H NMR (400 MHz, $CDCl_3$): δ 3.43 (td, *J* = 9.6, 4.2 Hz, 1H, C<u>H</u>OTMS), 3.53 (dd, *J* = 14.8, 7.1 Hz, 1H, C<u>H</u>$_2$CH=), 4.31 (dd, *J* = 14.8, 5.4 Hz, 1H, C<u>H</u>$_2$CH=), 4.40 (dd, *J* = 16.3, 4.3 Hz, 1H, C<u>H</u>$_2$CH=), 5.02 (d, *J* = 4.2 Hz, 1H, C<u>H</u>OTMP), 6.08-6.20 (m, 1H, C<u>H</u>=CH_2); ^{13}C NMR (101 MHz, $CDCl_3$): δ 1.4 (q, $Si(CH_3)_3$), 17.2 (t, piperidine-C4), 20.4 (q, NC<u>C</u>H_3), 20.6 (q, NC<u>C</u>H_3), 24.6 (t, C6), 24.7 (t, C4), 25.6 (t, C5), 33.4 (q, NC<u>C</u>H_3), 33.9 (q, NC<u>C</u>H_3), 36.4 (t, C3), 40.6 (t, piperidine-C3, C5), 47.0 (t, <u>C</u>H_2CH=), 49.7 (d, C1), 50.1 (t, <u>C</u>H_2CH=), 59.7 (s, CNO), 60.9 (s, CNO), 72.2 (d, C2), 78.2 (d, <u>C</u>HOTMP), 117.4 (t, CH=<u>C</u>H_2), 118.0 (t, CH=<u>C</u>H_2), 133.7 (d, <u>C</u>H=CH_2), 134.9 (d, <u>C</u>H=CH_2), 171.1 (s, C=O).

***N*,*N*-Diallyl-2-((1*R**,2*S**)-2-((trimethylsilyl)oxy)cyclohexyl)acetamide (S14):**

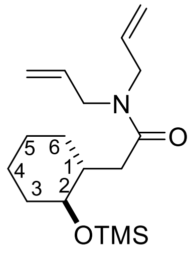

LiCl (252 mg, 6 mmol) was added to a round-bottomed flask containing a stirring bar, which was sealed with a septum, and dried under vacuum by a heat gun. Then, dry THF (8 mL) and amide **8a** (211 mg, 1.0 mmol) were added under argon. The mixture was cooled to 0 °C in an ice/water bath, *sec*-butyllithium (1.4 M solution in cyclohexane, 0.8 mL, 1.1 mmol) was added dropwise by syringe and the mixture was stirred at 0 °C for 15 min. The epoxide **7f** (102.9 mg, 1.05 mmol) was added at once by syringe and the reaction mixture was allowed to warm to room temperature and stirred at this temperature for 24 h. The reaction was quenched by saturated NH_4Cl solution and diluted with water (10 mL) and diethyl ether (10 mL). The organic layer was separated and the aqueous phase was extracted with diethyl ether (3 × 10 mL). The combined organic layers were dried over $MgSO_4$ and filtered. The filtrate was evaporated and the crude

product was purified by flash chromatography (gradient, hexanes/EtOAc 10:1 to 2:1) to give 247 mg (80%) pure amide **S14** as a single diastereomer.

[R_f (hexanes/EtOAc 10:1) = 0.41]; IR (film); ν [cm^{-1}]: 2929 (w), 2857 (w), 1642 (m), 1449 (w), 1411 (w), 1249 (w), 1083 (m), 992 (w), 949 (w), 919 (w), 887 (m), 838 (s), 750 (w), 690 (w); MS (+ESI) m/z, (%): 641 (30, [$2M+Na^+$]), 332 (100, [$M+Na^+$]); HRMS (+ESI) m/z [$C_{17}H_{31}NO_2SiNa^+$]: calcd. 332.2016; found 332.2014; ^{1}H NMR (400 MHz, $CDCl_3$): δ 0.09 (s, 9H, $Si(CH_3)_3$), 0.89-1.04 (m, 1H, H5), 1.13-1.37 (m, 3H, H3, H4, H6), 1.56-1.63 (m, 1H, H6), 1.66-1.77 (m, 2H, H1, H4), 1.81-1.86 (m, 1H, H3), 1.87 (dd, *J* = 14.3, 10.5 Hz, 1H, CH_2CO), 1.92-1.99 (m, 1H, H5), 2.85 (dd, *J* = 14.3, 2.7 Hz, 1H, CH_2CO), 3.21 (td, *J* = 10.1, 4.3 Hz, 1H, H2), 3.77-3.87 (m, 2H, $C\underline{H}_2CH=$), 3.98 (dd, *J* = 17.5, 4.8 Hz, 1H, $C\underline{H}_2CH=$), 4.11 (dd, *J* = 15.0, 5.8 Hz, 1H, $C\underline{H}_2CH=$), 5.10 (dd, *J* = 15.5, 1.6 Hz, 1H, $CH=C\underline{H}_2$), 5.13 (dd, *J* = 10.3, 1.6 Hz, 1H, $CH=C\underline{H}_2$), 5.14 (dd, *J* = 15.4, 1.3 Hz, 1H, $CH=C\underline{H}_2$), 5.19 (dd, *J* = 10.4, 1.3 Hz, 1H, $CH=C\underline{H}_2$), 5.69-5.81 (m, 2H, $C\underline{H}=CH_2$); ^{13}C NMR (101 MHz, $CDCl_3$): δ 0.6 (q, $Si(CH_3)_3$), 25.2 (t, C4), 25.6 (t, C6), 31.1 (t, C5), 36.2 (t, C3), 36.7 (t, $\underline{C}H_2CO$), 43.2 (d, C1), 47.8 (t, $\underline{C}H_2CH=$), 49.3 (t, $\underline{C}H_2CH=$), 75.2 (d, C2), 116.5 (t, $CH=\underline{C}H_2$), 117.0 (t, $CH=\underline{C}H_2$), 133.3 (d, $\underline{C}H=CH_2$), 133.8 (d, $\underline{C}H=CH_2$), 172.9 (s, C=O).

_N_-Benzyl-_N_-(cyclopent-1-en-1-ylmethyl)-2-((2,2,6,6-tetramethylpiperidin-1-yl)oxy)-4-((trimethylsilyl)oxy)pentanamide (9m):

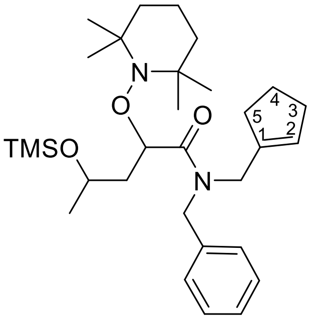

Prepared according to the general procedure, yield 312 mg (61%) as an inseparable 1.2:1 mixture of diastereomers. Ratio of rotamers is 1.4:1 for each diastereomer.

[R_f (hexanes/EtOAc 5:1) = 0.63]; IR (film); ν [cm^{-1}]: 2930 (m), 2871 (m), 2847 (m), 1652 (s), 1448 (m), 1376 (w), 1362 (w), 1250 (s), 1205 (w), 1133 (w), 1079 (m), 1021 (w), 990 (w), 973 (w), 957 (w), 892 (w), 841 (s), 747 (m), 702 (m), 601 (w); MS (+ESI) m/z, (%): 1051 (20, [$2M+Na^+$]), 537 (100, [$M+Na^+$]), 515 (15, [$M+H^+$]); HRMS (+ESI) m/z [$C_{30}H_{50}N_2O_3SiNa^+$]: calcd. 537.3483; found 537.3483.

First diastereomer: ^{1}H NMR (400 MHz, $CDCl_3$): δ 0.11 (s, 9H, $Si(CH_3)_3$), 0.89/1.13 (s, 3H, $NCCH_3$), 1.07/1.15 (s, 6H, $NCCH_3$), 1.12 (d, J = 6.0 Hz, 3H, CH_3), 1.23 (s, 3H, $NCCH_3$), 1.29-1.35 (m, 1H, piperidine-H4), 1.37-1.54 (m, 4H, piperidine-H3, H5), 1.55-1.68 (m, 1H, piperidine-H4), 1.84-2.01 (m, 2H, H4), 2.02-2.17 (m, 2H, C$\underline{H}_2$CHOTMP), 2.18-2.32 (m, 2H, H5), 2.33-2.44 (m, 2H, H3), 3.85 (d, J = 17.6 Hz, 1H, NCH_2C=), 3.93-4.04 (m, 1H, C$\underline{H}$OTMS), 4.35/4.48 (d, J = 13.9 Hz/J = 14.0 Hz, 1H, C$\underline{H}_2$Ph), 4.72 (d, J = 17.6 Hz, 1H, NCH_2C=), 4.80/4.89 (d, J = 14.0 Hz/J = 13.9 Hz, 1H, C$\underline{H}_2$Ph), 5.50-5.61 (m, 1H, H2), 7.27-7.44 (m, 5H, Ar$\underline{H}$); ^{13}C NMR (101 MHz, $CDCl_3$): δ 0.17 (q, $Si(CH_3)_3$), 16.5/16.6 (t, piperidine-C4), 19.5/19.85 (q, NC$\underline{C}H_3$), 19.7/19.93 (q, NC$\underline{C}H_3$), 22.78/22.85 (t, C4), 23.2/23.3 (q, CH_3), 31.6 (t, C3), 32.5 (q, NC$\underline{C}H_3$), 32.6 (q, NC$\underline{C}H_3$), 32.9/33.2 (t, C5), 39.7 (t, piperidine-C3), 39.8 (t, piperidine-C5), 41.7/41.9 (t, $\underline{C}H_2$CHOTMP), 43.8/46.5 (t, N$\underline{C}H_2$C=), 48.4/48.9 (t, $\underline{C}H_2$Ph), 59.4 (s, CNO), 59.8 (s, CNO), 64.7/64.8 (d, $\underline{C}$HOTMS), 78.7/79.5 (d, $\underline{C}$HOTMP), 126.5/126.6 (d, CH_{Ar}), 127.5/127.88 (d, CH_{Ar}), 129.0 (d, CH_{Ar}), 136.5/136.58 (s, C_{Ar}), 139.36/139.39 (s, C1), 171.1/172.2 (s, C=O).

Second diastereomer: ^{1}H NMR (400 MHz, $CDCl_3$): δ 0.15 (s, 9H, $Si(CH_3)_3$), 0.97/1.15 (s, 3H, $NCCH_3$), 0.99/1.20 (s, 3H, $NCCH_3$), 1.23 (s, 6H, $NCCH_3$), 1.22 (d, J = 6.1 Hz, 3H, CH_3), 1.29-1.35 (m, 1H, piperidine-H4), 1.37-1.54 (m, 4H, piperidine-H3, H5), 1.55-1.68 (m, 1H, piperidine-H4), 1.84-2.01 (m, 2H, H4), 2.03-2.17 (m, 2H, C$\underline{H}_2$CHOTMP), 2.18-2.32 (m, 2H, H5), 2.33-2.44 (m, 2H, H3), 3.80-3.92 (m, 1H, C$\underline{H}$OTMS), 3.98 (d, J = 17.5 Hz, 1H, NCH_2C=), 4.44 (d, J = 17.5 Hz, 1H, NCH_2C=), 4.48/4.60 (d, J = 14.0 Hz/J = 16.6 Hz, 1H, C$\underline{H}_2$Ph), 4.70/5.10 (d, J = 14.0 Hz/J = 16.6 Hz, 1H, C$\underline{H}_2$Ph), 5.50-5.61 (m, 1H, H2), 7.27-7.44 (m, 5H, Ar$\underline{H}$); ^{13}C NMR (101 MHz, $CDCl_3$): δ 0.14 (q, $Si(CH_3)_3$), 16.40/16.42 (t, piperidine-C4), 19.5/19.85 (q, NC$\underline{C}H_3$), 19.7/19.93 (q, NC$\underline{C}H_3$), 22.80/22.88 (t, C4), 23.9/24.0 (q, CH_3), 31.6 (t, C3), 32.5 (q, NC$\underline{C}H_3$), 32.6 (q, NC$\underline{C}H_3$), 33.0/33.5 (t, C5), 39.7 (t, piperidine-C3), 39.8 (t, piperidine-C5), 40.5/40.9 (t, $\underline{C}H_2$CHOTMP), 44.5/46.2 (t, N$\underline{C}H_2$C=), 47.8/49.3 (t, $\underline{C}H_2$Ph), 59.3 (s, CNO), 59.8 (s, CNO), 65.2/65.3 (d, $\underline{C}$HOTMS), 78.1/79.2 (d, $\underline{C}$HOTMP), 126.7/126.8 (d, CH_{Ar}), 127.6/127.90 (d, CH_{Ar}), 128.5 (d, CH_{Ar}), 136.63/136.7 (s, C_{Ar}), 139.0/139.1 (s, C1), 171.5/171.8 (s, C=O).

N-Benzyl-N-(cyclohex-1-en-1-ylmethyl)-2-((2,2,6,6-tetramethylpiperidin-1-yl)oxy)-4-((trimethylsilyl)oxy)pentanamide (9n):

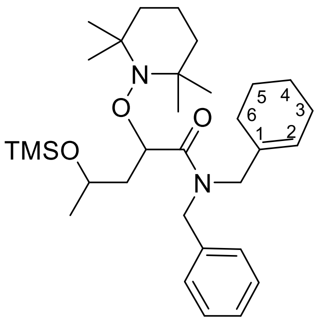

Prepared according to the general procedure, yield 327 mg (62%) as an inseparable 1.1:1 mixture of diastereomers. Ratio of rotamers is 1.5:1 for each diastereomer.

[R_f (hexanes/EtOAc 5:1) = 0.74]; IR (film); ν [cm^{-1}]: 2929 (m), 2873 (m), 1654 (s), 1445 (m), 1376 (w), 1362 (w), 1250 (m), 1205 (w), 1133 (w), 1100 (w), 1081 (w), 1013 (w), 957 (w), 894 (w), 840 (s), 750 (w), 701 (w), 613 (w); MS (+ESI) m/z, (%): 529 (100, [$M+H^+$]); HRMS (+ESI) m/z [$C_{31}H_{53}N_2O_3Si^+$]: calcd. 529.3820; found 529.3819.

First diastereomer: ^{1}H NMR (400 MHz, $CDCl_3$): δ 0.060/0.064 (s, 9H, $Si(CH_3)_3$), 0.85 (s, 3H, $NCCH_3$), 1.09 (s, 6H, $NCCH_3$), 1.11 (s, 3H, $NCCH_3$), 1.19 (d, J = 6.1 Hz, 3H, CH_3), 1.24-1.30 (m, 1H, piperidine-H4), 1.33-1.49 (m, 4H, piperidine-H3, H5), 1.50-1.54 (m, 1H, piperidine-H4), 1.55-1.65 (m, 4H, H4, H5), 1.82-1.94 (m, 2H, H6), 1.96-2.07 (m, 3H, H3, $C\underline{H}_2$CHOTMP), 2.08-2.15 (m, 1H, $C\underline{H}_2$CHOTMP), 3.63/3.67 (d, J = 16.8 Hz/J = 14.5 Hz, 1H, $C\underline{H}_2$Ph), 3.78-3.85 (m, 1H, C$\underline{H}$OTMS), 4.05/4.52 (d, J = 14.5 Hz/J = 16.8 Hz, 1H, $C\underline{H}_2$Ph), 4.27/4.54 (d, J = 14.1 Hz/J = 16.6 Hz, 1H, NCH_2C=), 4.65/4.84 (dd, J = 9.0, 4.1 Hz/J = 10.0, 3.4 Hz, 1H, C$\underline{H}$OTMP), 4.71/5.05 (d, J = 14.1 Hz/J = 16.6 Hz, 1H, NCH_2C=), 5.43-5.47/5.49 (m/dd, J = 3.4, 1.8 Hz, 1H, H2), 7.20-7.41 (m, 5H, Ar$\underline{H}$); ^{13}C NMR (101 MHz, $CDCl_3$): δ 0.57 (q, $Si(CH_3)_3$), 17.23 (t, piperidine-C4), 20.3/20.6 (q, $NC\underline{C}H_3$), 20.5/20.7 (q, $NC\underline{C}H_3$), 22.4/22.49 (t, C5), 22.50/22.7 (t, C4), 24.7/24.8 (q, CH_3), 25.0/25.2 (t, C3), 26.7/26.82 (t, C6), 33.3/33.4 (q, $NC\underline{C}H_3$), 33.65/33.73 (q, $NC\underline{C}H_3$), 40.5/40.6 (t, piperidine-C3, C5), 42.3/42.6 (t, $\underline{C}H_2$CHOTMP), 49.0/49.4 (t, $N\underline{C}H_2C$=), 50.3/52.4 (t, $\underline{C}H_2$Ph), 59.5/60.1 (s, 2C, CNO), 65.9/66.1 (d, $\underline{C}$HOTMS), 78.8/79.0 (d, $\underline{C}$HOTMP), 123.6/125.3 (d, C2), 127.20/128.2 (d, CH_{Ar}), 127.4/127.7 (d, CH_{Ar}), 128.6/129.7 (d, CH_{Ar}), 132.77/133.1 (s, C1), 137.3/137.45 (s, C_{Ar}), 171.9/173.1 (s, C=O).

Second diastereomer: ^{1}H NMR (400 MHz, $CDCl_3$): δ 0.07/0.10 (s, 9H, $Si(CH_3)_3$), 0.97 (s, 3H, $NCCH_3$), 1.03 (s, 3H, $NCCH_3$), 1.06/1.16 (d, J = 6.1 Hz, 3H, CH_3), 1.10 (s, 3H, $NCCH_3$), 1.19 (s, 3H, $NCCH_3$), 1.24-1.30 (m, 1H, piperidine-H4), 1.33-1.49 (m, 4H, piperidine-H3, H5), 1.50-

1.54 (m, 1H, piperidine-H4), 1.55-1.65 (m, 4H, H4, H5), 1.82-1.94 (m, 2H, H6), 1.95-2.09 (m, 3H, H3, C$\underline{H}_2$CHOTMP), 2.18-2.28 (m, 1H, C$\underline{H}_2$CHOTMP), 3.79 (s, 2H, C$\underline{H}_2$Ph)/3.82 (d, J = 16.8 Hz, 1H, C$\underline{H}_2$Ph)/4.16 (d, J = 16.8 Hz, 1H, C$\underline{H}_2$Ph), 3.89-3.98 (m, 1H, C$\underline{H}$OTMS), 4.49 (d, J = 14.4 Hz, 1H, NCH_2C=), 4.57 (d, J = 14.4 Hz, 1H, NCH_2C=), 4.58-4.61/4.72-4.76 (m, 1H, C$\underline{H}$OTMP), 5.43-5.47/5.51-5.55 (m, 1H, H2), 7.20-7.41 (m, 5H, Ar$\underline{H}$); ^{13}C NMR (101 MHz, $CDCl_3$): δ 0.61 (q, $Si(CH_3)_3$), 17.16/17.3 (t, piperidine-C4), 20.3/20.6 (q, N$C\underline{C}H_3$), 20.5/20.7 (q, N$C\underline{C}H_3$), 22.3/22.48 (t, C5), 22.53/22.6 (t, C4), 23.97/24.03 (q, CH_3), 25.0/25.3 (t, C3), 26.79/27.1 (t, C6), 33.3/33.4 (q, N$C\underline{C}H_3$), 33.65/33.73 (q, N$C\underline{C}H_3$), 40.5/40.6 (t, piperidine-C3, C5), 41.0/41.6 (t, $\underline{C}H_2$CHOTMP), 48.4/49.8 (t, N$\underline{C}H_2$C=), 51.3/52.1 (t, $\underline{C}H_2$Ph), 60.0/60.5 (s, 2C, CNO), 65.5/65.6 (d, $\underline{C}$HOTMS), 77.7/77.9 (d, $\underline{C}$HOTMP), 123.9/125.9 (d, C2), 127.24/128.3 (d, CH_{Ar}), 127.3/127.5 (d, CH_{Ar}), 128.6/129.2 (d, CH_{Ar}), 132.80/133.5 (s, C1), 137.51/137.6 (s, C_{Ar}), 172.5/172.7 (s, C=O).

***N*-Benzyl-*N*-(cyclopent-2-en-1-yl)-2-((2,2,6,6-tetramethylpiperidin-1-yl)oxy)-4-((trimethylsilyl)oxy)pentanamide (9o):**

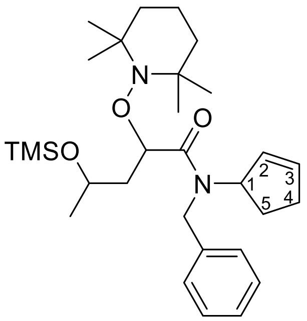

Prepared according to the general procedure, yield 340 mg (68%) as an inseparable 2:2:1:1 mixture of diastereomers. Ratio of rotamers is 1.3:1 for each diastereomer.

[R_f (hexanes/EtOAc 5:1) = 0.64]; IR (film); ν [cm^{-1}]: 2966 (w), 2930 (m), 2871 (w), 1651 (s), 1453 (w), 1439 (w), 1376 (w), 1362 (w), 1250 (m), 1133 (m), 1098 (w), 1078 (w), 1060 (w), 1015 (w), 990 (w), 957 (w), 892 (w), 840 (s), 748 (w), 726 (m), 699 (m), 605 (w); MS (+ESI) m/z, (%): 523 (65, [$M+Na^+$]), 501 (100, [$M+H^+$]); HRMS (+ESI) m/z [$C_{29}H_{49}N_2O_3Si^+$]: calcd. 501.3507; found 501.3506.

Major diastereomers: ^{1}H NMR (400 MHz, $CDCl_3$): δ –0.04/0.06 (s, 9H, $Si(CH_3)_3$), 0.08 (s, 9H, $Si(CH_3)_3$), 0.90/1.16 (d, J = 6.1 Hz, 3H, CH_3), 0.95/1.17 (d, J = 6.0 Hz, 3H, CH_3), 1.07 (s, 6H, $NCCH_3$), 1.10 (s, 6H, $NCCH_3$), 1.11 (s, 6H, $NCCH_3$), 1.12 (s, 6H, $NCCH_3$), 1.26-1.35 (m, 2H, piperidine-H4), 1.37-1.49 (m, 8H, piperidine-H3, H5), 1.50-1.70 (m, 4H, H5, piperidine-H4),

2.02-2.15 (m, 3H, C$\underline{H}_2$CHOTMP), 2.16-2.26 (m, 4H, H4, C$\underline{H}_2$CHOTMP), 2.27-2.50 (m, 3H, H4, H5), 3.76 (ddq, *J* = 9.2, 6.1, 3.0 Hz, 1H, C$\underline{H}$OTMS), 3.81-3.87 (m, 1H, C$\underline{H}$OTMS), 4.19 (d, *J* = 14.9 Hz, 1H, C$\underline{H}_2$Ph), 4.27 (d, *J* = 16.9 Hz, 1H, C$\underline{H}_2$Ph), 4.36-4.51 (m, 1H, C$\underline{H}$OTMP), 4.55 (d, *J* = 14.9 Hz, 1H, C$\underline{H}_2$Ph), 4.87 (dd, *J* = 10.5, 5.7 Hz, 1H, C$\underline{H}$OTMP), 5.09 (d, *J* = 16.9 Hz, 1H, C$\underline{H}_2$Ph), 5.43-5.48 (m, 1H, H2), 5.54-5.57 (m, 1H, H1), 5.58-5.63 (m, 2H, H1, H2), 5.77-5.83 (m, 1H, H3), 5.84-5.87 (m, 1H, H3), 7.14-7.34 (m, 10H, Ar$\underline{H}$); ^{13}C NMR (101 MHz, $CDCl_3$): δ 0.36/0.40 (q, $Si(CH_3)_3$), 0.61/0.64 (q, $Si(CH_3)_3$), 17.2 (t, 2C, piperidine-C4), 20.2 (q, 2C, NC$\underline{C}H_3$), 20.3 (q, 2C, NC$\underline{C}H_3$), 23.6/24.0 (q, CH_3), 23.9 (q, CH_3), 27.4/27.8 (t, C5), 28.3/28.8 (t, C5), 31.40 (t, C4), 31.42/31.47 (t, C4), 33.27 (q, 2C, NC$\underline{C}H_3$), 33.32 (q, NC$\underline{C}H_3$), 33.5 (q, NC$\underline{C}H_3$), 40.31/40.4 (t, piperidine-C3, C5), 40.34/40.5 (t, piperidine-C3, C5), 41.0 (t, $\underline{C}H_2$CHOTMP), 41.6/42.6 (t, $\underline{C}H_2$CHOTMP), 45.57/45.62 (t, $\underline{C}H_2$Ph), 45.7/45.8 (t, $\underline{C}H_2$Ph), 59.4 (s, CNO), 59.5 (s, CNO), 60.0 (s, CNO), 60.5 (s, CNO), 61.1/61.6 (d, C1), 61.2/61.8 (d, C1), 65.08/65.11 (d, $\underline{C}$HOTMS), 65.47/65.54 (d, $\underline{C}$HOTMS), 77.0 (d, $\underline{C}$HOTMP), 77.9/78.2 (d, $\underline{C}$HOTMP), 126.1 (d, CH_{Ar}), 126.2 (d, CH_{Ar}), 126.9 (d, CH_{Ar}), 127.6 (d, CH_{Ar}), 128.3 (d, CH_{Ar}), 128.6 (d, CH_{Ar}), 130.17/130.5 (d, C3), 130.24/130.6 (d, C3), 134.7/134.8 (d, C2), 134.9 (d, C2), 139.2/139.3 (s, C_{Ar}), 139.38/139.39 (s, C_{Ar}), 171.6/172.1 (s, C=O), 172.5/172.6 (s, C=O).

Minor diastereomers: ^{1}H NMR (400 MHz, $CDCl_3$): δ 0.05/0.06 (s, 9H, $Si(CH_3)_3$), 0.07/0.10 (s, 9H, $Si(CH_3)_3$), 0.96/1.21 (d, *J* = 6.1 Hz, 3H, CH_3), 1.03/1.22 (d, *J* = 6.1 Hz, 3H, CH_3), 1.08 (s, 6H, $NCCH_3$), 1.09 (s, 6H, $NCCH_3$), 1.12 (s, 12H, $NCCH_3$), 1.26-1.35 (m, 2H, piperidine-H4), 1.37-1.49 (m, 8H, piperidine-H3, H5), 1.50-1.70 (m, 4H, H5, piperidine-H4), 1.72-1.91 (m, 4H, H5, C$\underline{H}_2$CHOTMP), 1.94-2.02 (m, 4H, H4, C$\underline{H}_2$CHOTMP), 2.26-2.51 (m, 2H, H4), 3.79-4.00 (m, 2H, C$\underline{H}$OTMS), 4.28 (d, *J* = 15.2 Hz, 1H, C$\underline{H}_2$Ph), 4.35 (d, *J* = 16.7 Hz, 1H, C$\underline{H}_2$Ph), 4.36-4.51 (m, 1H, C$\underline{H}$OTMP), 4.81 (dd, *J* = 7.8, 6.1 Hz, 1H, C$\underline{H}$OTMP), 5.10 (d, *J* = 15.2 Hz, 1H, C$\underline{H}_2$Ph), 5.20 (d, *J* = 16.7 Hz, 1H, C$\underline{H}_2$Ph), 5.49-5.53 (m, 1H, H2), 5.64-5.71 (m, 2H, H1, H2), 5.72-5.78 (m, 1H, H1), 5.89-6.01 (m, 2H, H3), 7.14-7.34 (m, 10H, Ar$\underline{H}$); ^{13}C NMR (101 MHz, $CDCl_3$): δ 0.5 (q, $Si(CH_3)_3$), 0.7 (q, $Si(CH_3)_3$), 17.3 (t, 2C, piperidine-C4), 20.1 (q, 2C, NC$\underline{C}H_3$), 20.7 (q, 2C, NC$\underline{C}H_3$), 24.4/24.5 (q, CH_3), 24.88/24.92 (q, CH_3), 28.4/29.2 (t, C5), 28.9/29.4 (t, C5), 31.53/31.61 (t, C4), 31.63 (t, C4), 33.58 (q, NC$\underline{C}H_3$), 33.64 (q, NC$\underline{C}H_3$), 33.9 (q, NC$\underline{C}H_3$), 34.1 (q, NC$\underline{C}H_3$), 40.6/40.7 (t, piperidine-C3, C5), 40.76/40.79 (t, piperidine-C3, C5), 42.7/43.4 (t, $\underline{C}H_2$CHOTMP), 42.8/43.5 (t, $\underline{C}H_2$CHOTMP), 46.6/46.68 (t, $\underline{C}H_2$Ph), 46.70 (t, $\underline{C}H_2$Ph), 59.7 (s, CNO), 59.8 (s, CNO), 60.6 (s, CNO), 60.8 (s, CNO), 63.6 (d, C1), 63.7/63.8 (d, C1), 65.6/66.0 (d, $\underline{C}$HOTMS), 66.1 (d, $\underline{C}$HOTMS), 78.6/79.2 (d, $\underline{C}$HOTMP), 78.7/80.4 (d,

$\underline{C}$HOTMP), 126.3 (d, CH_{Ar}), 126.5 (d, CH_{Ar}), 126.8 (d, CH_{Ar}), 127.7 (d, CH_{Ar}), 128.1 (d, CH_{Ar}), 128.5 (d, CH_{Ar}), 130.7/131.0 (d, C3), 131.1/131.2 (d, C3), 134.96/135.00 (d, C2), 135.1/135.4 (d, C2), 139.6/139.69 (s, C_{Ar}), 139.70/139.8 (s, C_{Ar}), 173.7/173.8 (s, C=O), 174.4/174.5 (s, C=O).

***N*-Benzyl-*N*-((*R*)-cyclohex-2-en-1-yl)-2-((2,2,6,6-tetramethylpiperidin-1-yl)oxy)-4-((trimethylsilyl)oxy)pentanamide (9p):**

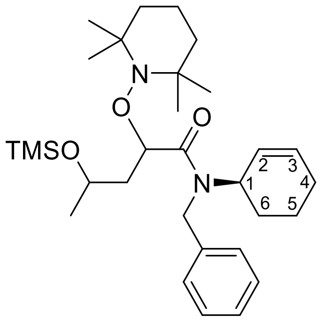

Prepared according to the general procedure, yield 323 mg (63%) as an inseparable 2:2:1:1 mixture of diastereomers. Ratio of rotamers is 1.5:1 for each diastereomer.

[R_f (hexanes/EtOAc 5:1) = 0.54]; IR (film); ν [cm^{-1}]: 3005 (m), 2930 (s), 2872 (m), 1647 (s), 1452 (m), 1376 (w), 1362 (w), 1250 (s), 1099 (w), 1078 (m), 1057 (w), 1016 (w), 989 (w), 957 (w), 897 (w), 840 (s), 729 (m), 698 (m), 615 (w); MS (+ESI) m/z, (%): 1051 (30, [$2M+Na^+$], 537 (100, [$M+Na^+$]), 515 (90, [$M+H^+$]); HRMS (+ESI) m/z [$C_{30}H_{50}N_2O_3SiNa^+$]: calcd. 537.3480; found 537.3479.

Major diastereomers: ^1H NMR (400 MHz, $CDCl_3$): δ –0.05/–0.03 (s, 9H, $Si(CH_3)_3$), 0.06/0.07 (s, 9H, $Si(CH_3)_3$), 0.89 (d, *J* = 6.1 Hz, 3H, CH_3), 0.96/1.16 (d, *J* = 6.1 Hz, 3H, CH_3), 1.07 (s, 3H, $NCCH_3$), 1.08 (s, 6H, $NCCH_3$), 1.09 (s, 6H, $NCCH_3$), 1.10 (s, 9H, $NCCH_3$), 1.24-1.34 (m, 2H, piperidine-H4), 1.36-1.48 (m, 10H, H4, piperidine-H3, H5), 1.49-1.65 (m, 6H, H4, H6, piperidine-H4), 1.68-1.83 (m, 2H, H6), 1.84-1.90 (m, 2H, $C\underline{H}_2$CHOTMP), 1.91-2.04 (m, 6H, H5, $C\underline{H}_2$CHOTMP), 3.70-3.79 (m, 2H, $C\underline{H}$OTMS), 4.16 (d, *J* = 15.0 Hz, 1H, $C\underline{H}_2$Ph), 4.25 (d, *J* = 15.4 Hz, 1H, $C\underline{H}_2$Ph), 4.40-4.55 (m, 2H, $C\underline{H}$OTMP), 4.45 (d, *J* = 15.4 Hz, 1H, $C\underline{H}_2$Ph), 4.78 (d, *J* = 15.0 Hz, 1H, $C\underline{H}_2$Ph), 5.04-5.17 (m, 2H, H1), 5.37-5.55 (m, 1H, H2), 5.60-5.68 (m, 1H, H2), 5.69-5.81 (m, 1H, H3), 5.82-5.96 (m, 1H, H3), 7.15-7.37 (m, 10H, Ar$\underline{H}$); ^{13}C NMR (101 MHz, $CDCl_3$): δ 0.4/0.5 (q, $Si(CH_3)_3$), 0.6/0.8 (q, $Si(CH_3)_3$), 17.19 (t, piperidine-C4), 17.23 (t, piperidine-C4), 20.2 (q, 2C, $NC\underline{C}H_3$), 20.25 (q, $NC\underline{C}H_3$), 20.29 (q, $NC\underline{C}H_3$), 21.47/21.53 (t, C6), 21.6/21.8 (t, C6), 23.8/23.9 (q, CH_3), 24.09/24.12 (q, CH_3), 24.66 (t, 2C, C5), 26.7/27.73 (t, C4), 27.0/27.68 (t, C4), 33.28 (q, $NC\underline{C}H_3$), 33.32 (q, $NC\underline{C}H_3$), 33.4 (q, 2C, $NC\underline{C}H_3$), 40.4 (t,

piperidine-C3), 40.5 (t, piperidine-C3), 40.6 (t, 2C, piperidine-C5), 41.0 (t, $\underline{C}H_2$CHOTMP), 42.7/42.8 (t, $\underline{C}H_2$CHOTMP), 46.2/46.33 (t, $\underline{C}H_2$Ph), 46.27 (t, $\underline{C}H_2$Ph), 51.9/52.0 (d, C1), 52.5/52.6 (d, C1), 59.4 (s, CNO), 60.0 (s, CNO), 60.5 (s, CNO), 60.7 (s, CNO), 65.1/65.2 (d, $\underline{C}$HOTMS), 65.60/65.71 (d, $\underline{C}$HOTMS), 77.7/78.1 (d, $\underline{C}$HOTMP), 78.2/78.6 (d, $\underline{C}$HOTMP), 126.19/126.23 (d, CH_{Ar}), 126.48/126.53 (d, CH_{Ar}), 127.6/127.7 (d, CH_{Ar}), 128.0/128.08 (d, CH_{Ar}), 128.12 (d, 2C, CH_{Ar}), 128.45/128.54 (d, C2), 128.51/128.53 (d, C2), 131.3/131.36 (d, C3), 131.41/131.44 (d, C3), 139.4 (s, C_{Ar}), 139.5 (s, C_{Ar}), 173.8 (s, C=O), 174.4 (s, C=O).

Minor diastereomers: ^{1}H NMR (400 MHz, $CDCl_3$): δ 0.076/0.077 (s, 9H, $Si(CH_3)_3$), 0.09/0.16 (s, 9H, $Si(CH_3)_3$), 1.02/1.17 (d, J = 6.2 Hz, 3H, CH_3), 1.03/1.21 (d, J = 6.2 Hz, 3H, CH_3), 1.12 (s, 6H, $NCCH_3$), 1.13 (s, 6H, $NCCH_3$), 1.14 (s, 6H, $NCCH_3$), 1.15 (s, 6H, $NCCH_3$), 1.24-1.34 (m, 2H, piperidine-H4), 1.36-1.48 (m, 8H, piperidine-H3, H5), 1.49-1.65 (m, 6H, H4, H6, piperidine-H4), 1.83-1.90 (m, 5H, H4, H6, $C\underline{H}_2$CHOTMP), 1.91-2.04 (m, 7H, H4, H5, $C\underline{H}_2$CHOTMP), 3.87-4.03 (m, 2H, C$\underline{H}$OTMS), 4.33 (d, J = 16.4 Hz, 1H, $C\underline{H}_2$Ph), 4.39 (d, J = 17.5 Hz, 1H, $C\underline{H}_2$Ph), 4.56 (d, J = 17.5 Hz, 1H, $C\underline{H}_2$Ph), 4.73-4.85 (m, 2H, C$\underline{H}$OTMP), 4.82 (d, J = 16.4 Hz, 1H, $C\underline{H}_2$Ph), 5.18-5.35 (m, 2H, H1), 5.37-5.55 (m, 2H, H2), 5.81-5.96 (m, 2H, H3), 7.15-7.37 (m, 10H, Ar$\underline{H}$); ^{13}C NMR (101 MHz, $CDCl_3$): δ 0.7 (q, $Si(CH_3)_3$), 1.4 (q, $Si(CH_3)_3$), 17.24 (t, piperidine-C4), 17.3 (t, piperidine-C4), 20.4 (q, $NC\underline{C}H_3$), 20.45 (q, $NC\underline{C}H_3$), 20.49 (q, 2C, $NC\underline{C}H_3$), 21.87/22.0 (t, C6), 21.90 (t, C6), 24.45/24.53 (q, CH_3), 24.6/24.73 (t, C5), 24.8 (t, C5), 25.0 (q, CH_3), 29.40/29.6 (t, C4), 29.42/29.7 (t, C4), 33.2 (q, $NC\underline{C}H_3$), 33.6 (q, $NC\underline{C}H_3$), 33.7 (q, 2C, $NC\underline{C}H_3$), 40.4 (t, piperidine-C3), 40.5 (t, piperidine-C3), 40.7 (t, piperidine-C5), 40.8 (t, piperidine-C5), 42.9/43.4 (t, $\underline{C}H_2$CHOTMP), 43.5/43.9 (t, $\underline{C}H_2$CHOTMP), 47.1/47.3 (t, $\underline{C}H_2$Ph), 47.4/47.5 (t, $\underline{C}H_2$Ph), 54.62 (d, C1), 54.63/54.8 (d, C1), 59.4 (s, CNO), 60.0 (s, CNO), 60.8 (s, CNO), 60.9 (s, CNO), 65.63/65.69 (d, $\underline{C}$HOTMS), 66.1/66.2 (d, $\underline{C}$HOTMS), 78.8 (d, $\underline{C}$HOTMP), 78.9/79.5 (d, $\underline{C}$HOTMP), 125.9/126.0 (d, CH_{Ar}), 126.3/126.4 (d, CH_{Ar}), 126.8/126.9 (d, CH_{Ar}), 127.0 (d, CH_{Ar}), 127.80/127.84 (d, CH_{Ar}), 128.2 (d, CH_{Ar}), 128.6/128.8 (d, C2), 128.7/128.9 (d, C2), 131.60/131.63 (d, C3), 132.1/132.5 (d, C3), 139.77 (s, C_{Ar}), 139.84 (s, C_{Ar}), 174.5 (s, C=O), 174.8 (s, C=O).

General procedure for the thermal radical cyclization of compounds 9 and subsequent deprotection:

The α-(aminoxy)amide **9** (0.65 mmol) was heated in *t*-BuOH (6 mL) in a microwave reactor at 150 °C for 1 h. The reaction mixture was diluted with diethyl ether (5 mL), transferred into a round-bottomed Schlenk flask and evaporated. The crude residue was dissolved in dry THF (5 mL), the reaction mixture was cooled to 0 °C in an ice/water bath, tetrabutylammonium fluoride (1 M solution in THF, 0.96 mL, 0.96 mmol) was added and the mixture was stirred at this temperature for 30 min. The reaction was quenched by saturated NH_4Cl solution and diluted with water (5 mL) and diethyl ether (5 mL), the organic layer was separated and the aqueous phase was extracted with diethyl ether (2 × 5 mL). The combined organic layers were dried over $MgSO_4$, filtered and evaporated. The crude mixture was purified by column chromatography (gradient, hexanes/EtOAc 5:1 to 1:1) to give pure lactams **12** as diastereomeric mixtures. Their configuration was deducted from the base-mediated *cis*–*trans* isomerization (vide infra) and from NOE measurement of the derived alcohol oxidation products (see pp. S79–S97).

Equilibration of lactams 12:

A solution of KO*t*-Bu (1 M in THF, 0.25 mL, 0.25 mmol) was added to a stirred solution of hydroxy lactam **12** (0.50 mmol) in *t*-BuOH (3 mL) at room temperature and the reaction mixture was stirred for 24 h. The reaction was quenched by saturated NH_4Cl solution and diluted with water (3 mL) and diethyl ether (5 mL). The organic layer was separated and the aqueous phase was extracted with diethyl ether (3 × 5 mL). The combined organic layers were dried over $MgSO_4$ and filtered. The filtrate was evaporated and the diastereomeric ratio was determined by ^{1}H NMR spectroscopy. The crude mixture was purified by flash chromatography (gradient, hexanes/EtOAc 10:1 to 1:1) to give lactam *trans*-**12**.

(3*R,4*R**)- and (3*S**,4*R**)-1-Allyl-3-(2-hydroxy-2-methylpropyl)-4-(((2,2,6,6-tetramethyl-piperidin-1-yl)oxy)methyl)pyrrolidin-2-one (12a):**

Prepared according to the general procedure, yield 270 mg (91%) as an inseparable 2(*trans*):1(*cis*) mixture of diastereomers. After equilibration the diastereomeric ratio for *trans*-**12a** increased to 17(*trans*):1(*cis*).

[R_f (hexanes/EtOAc 1:1) = 0.29]; IR (film); ν [cm^{-1}]: 3327 (br), 2970 (m), 2930 (m), 2872 (w), 1667 (s), 1469 (w), 1450 (w), 1418 (w), 1375 (w), 1360 (w), 1263 (w), 1155 (w), 1133 (w), 1046

(w), 993 (w), 923 (w), 787 (w), 719 (w), 636 (w); MS (+ESI) m/z, (%): 389 (25, [M+Na$^+$]), 367 (100, [M+H$^+$]); HRMS (+ESI) m/z [$C_{21}H_{39}N_2O_3^+$]: calcd. 367.2955; found 367.2954.

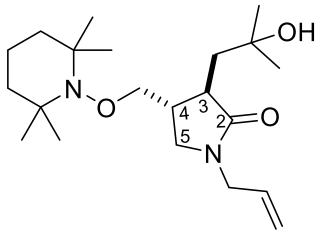

(3*R**,4*R**)

Major diastereomer: ^{1}H NMR (400 MHz, $CDCl_3$): δ 1.09 (s, 6H, $NCCH_3$), 1.14 (s, 6H, $NCCH_3$), 1.26 (s, 3H, CH_3), 1.27 (s, 3H, CH_3), 1.30-1.37 (m, 1H, piperidine-H4), 1.41-1.48 (m, 4H, piperidine-H3, H5), 1.49-1.56 (m, 1H, piperidine-H4), 1.74 (dd, *J* = 14.3, 3.7 Hz, 1H, CH_2C), 1.85 (dd, *J* = 14.3, 9.2 Hz, 1H, CH_2C), 2.19-2.30 (m, 1H, H4), 2.73 (ddd, *J* = 10.1, 9.2, 3.7 Hz, 1H, H3), 3.22 (t, *J* = 9.2 Hz, 1H, H5), 3.42 (t, *J* = 9.2 Hz, 1H, H5), 3.79-3.87 (m, 3H, C$\underline{H}_2$OTMP, C$\underline{H}_2$CH=), 3.95 (dd, *J* = 15.1, 5.9 Hz, 1H, C$\underline{H}_2$CH=), 5.11-5.25 (m, 2H, CH=C$\underline{H}_2$), 5.66-5.74 (m, 1H, C$\underline{H}$=CH_2), 5.76 (s, 1H, OH); ^{13}C NMR (101 MHz, $CDCl_3$): δ 17.1 (t, piperidine-C4), 20.31 (q, 2C, NC$\underline{C}H_3$), 28.5 (q, CH_3), 31.9 (q, CH_3), 33.3 (q, 2C, NC$\underline{C}H_3$), 39.2 (d, C4), 39.8 (t, piperidine-C3, C5), 42.1 (d, C3), 44.6 (t, $\underline{C}H_2$C), 45.7 (t, $\underline{C}H_2$CH=), 48.9 (t, C5), 60.1 (s, 2C, CNO), 69.06 (s, $\underline{C}$OH), 76.8 (t, $\underline{C}H_2$OTMP), 118.4 (t, CH=$\underline{C}H_2$), 132.0 (d, $\underline{C}$H=CH_2), 177.4 (s, C2).

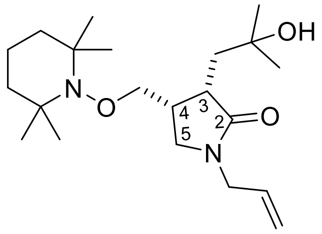

(3*S**,4*R**)

Minor diastereomer: ^{1}H NMR (400 MHz, $CDCl_3$, detectable signals): δ 1.11 (s, 6H, $NCCH_3$), 1.29 (s, 6H, CH_3), 1.61 (dd, *J* = 14.5, 4.2 Hz, 1H, CH_2C), 1.79-1.86 (m, 1H, CH_2C), 2.61-2.69 (m, 1H, H4), 2.85-2.96 (m, 1H, H3), 3.35 (dd, *J* = 10.3, 2.4 Hz, 1H, H5), 3.39-3.45 (m, 1H, H5), 3.66 (t, *J* = 9.2 Hz, 1H, C$\underline{H}_2$OTMP), 3.76 (dd, *J* = 9.0, 5.7 Hz, 1H, C$\underline{H}_2$OTMP); ^{13}C NMR (101 MHz, $CDCl_3$): δ 17.2 (t, piperidine-C4), 20.26 (q, NC$\underline{C}H_3$), 20.4 (q, NC$\underline{C}H_3$), 28.4 (q, CH_3), 31.8 (q, CH_3), 33.1 (q, NC$\underline{C}H_3$), 33.4 (q, NC$\underline{C}H_3$), 36.3 (d, C4), 39.0 (t, $\underline{C}H_2$C), 39.8 (t,

piperidine-C3, C5), 41.3 (d, C3), 45.8 (t, CH_2CH=), 48.8 (t, C5), 60.1 (s, 2C, CNO), 69.11 (s, COH), 75.0 (t, CH_2OTMP), 118.8 (t, CH=CH_2), 132.2 (d, CH=CH_2), 177.1 (s, C2).

(3*R,4*R**)- and (3*S**,4*R**)-1-Allyl-3-(2-hydroxypropyl)-4-(((2,2,6,6-tetramethylpiperidin-1-yl)oxy)methyl)pyrrolidin-2-one (12b):**

Prepared according to the general procedure, yield 200 mg (87%) as an inseparable 2.5:2.5(*trans*):1:1(*cis*) mixture of diastereomers. After equilibration the diastereomeric ratio for *trans*-**12b** increased to 12:12(*trans*):1:1(*cis*).

[R_f (hexanes/EtOAc 1:1) = 0.51]; IR (film); ν [cm^{-1}]: 3356 (br), 2975 (w), 2928 (m), 2870 (w), 1666 (s), 1492 (w), 1470 (m), 1453 (w), 1416 (w), 1374 (w), 1359 (w), 1262 (m), 1244 (w), 1133 (w), 1047 (w), 1029 (w), 993 (w), 924 (w), 788 (w), 720 (w), 654 (w); MS (+ESI) m/z, (%): 727 (40, [2M+Na^+], 375 (100, [M+Na^+]), 353 (60, [M+H^+]); HRMS (+ESI) m/z [$C_{20}H_{36}N_2O_3Na^+$]: calcd. 375.2618; found 375.2620.

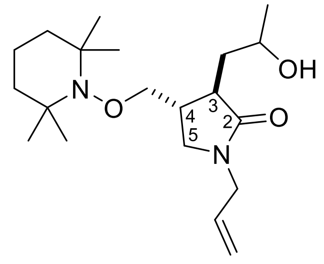

(3*R**,4*R**)

Major diastereomers: ^{1}H NMR (400 MHz, $CDCl_3$): δ 1.07 (s, 9H, $NCCH_3$), 1.08 (s, 6H, $NCCH_3$), 1.11 (s, 6H, $NCCH_3$), 1.13 (s, 3H, $NCCH_3$), 1.20 (d, *J* = 6.2 Hz, 3H, CH_3), 1.22 (d, *J* = 6.4 Hz, 3H, CH_3), 1.27-1.35 (m, 2H, piperidine-H4), 1.39-1.46 (m, 8H, piperidine-H3, H5), 1.47-1.59 (m, 2H, piperidine-H4), 1.60-1.69 (m, 1H, CH_2CHOH), 1.70-1.81 (m, 2H, CH_2CHOH), 1.90 (ddd, *J* = 14.3, 8.5, 3.4 Hz, 1H, CH_2CHOH), 2.17-2.28 (m, 1H, H4), 2.29-2.40 (m, 1H, H4), 2.56 (td, *J* = 9.8, 3.2 Hz, 1H, H3), 2.63 (td, *J* = 8.5, 4.9 Hz, 1H, H3), 3.12-3.20 (m, 2H, H5), 3.41 (dd, *J* = 11.7, 8.4 Hz, 1H, H5), 3.42 (dd, *J* = 10.9, 7.5 Hz, 1H, H5), 3.78-3.86 (m, 6H, CH_2OTMP, CH_2CH=), 3.89-3.97 (m, 3H, CHOH, CH_2CH=), 4.03-4.13 (m, 1H, CHOH), 4.31 (br. s, 2H, OH), 5.14-5.23 (m, 4H, CH=CH_2), 5.70 (ddt, *J* = 16.5, 10.4, 6.1 Hz, 2H, CH=CH_2); ^{13}C NMR (101 MHz, $CDCl_3$): δ 17.1 (t, 2C, piperidine-C4), 20.3 (q, 4C, $NCCH_3$), 23.1 (q, CH_3), 24.3 (q, CH_3), 33.3 (q, 4C, $NCCH_3$), 38.0 (d, C4), 38.9 (d, C4), 39.3 (t, CH_2CHOH), 39.7 (t, piperidine-C3, C5), 39.8 (t, piperidine-C3, C5), 40.8 (t, CH_2CHOH), 42.2 (d, C3), 45.56 (t, CH_2CH=), 45.7 (t, CH_2CH=), 45.83 (d, C3), 48.7 (t, C5), 48.81 (t, C5), 60.11 (s, 2C, CNO),

60.13 (s, 2C, CNO), 66.0 (d, CHOH), 68.2 (d, CHOH), 76.8 (t, $\underline{C}H_2$OTMP), 77.4 (t, $\underline{C}H_2$OTMP), 118.3 (t, CH=$\underline{C}H_2$), 118.4 (t, CH=$\underline{C}H_2$), 131.9 (d, $\underline{C}$H=CH_2), 132.14 (d, $\underline{C}$H=CH_2), 176.8 (s, C2), 177.4 (s, C2).

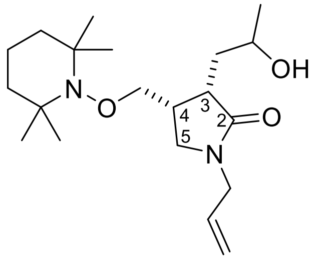

(3*S**,4*R**)

Minor diastereomers: ^{1}H NMR (400 MHz, $CDCl_3$, detectable signals): δ 1.59-1.66 (m, 2H, C$\underline{H}_2$CHOH), 1.79-1.87 (m, 2H, C$\underline{H}_2$CHOH), 2.64-2.73 (m, 2H, H4), 2.74-2.82 (m, 1H, H3), 2.83-2.89 (m, 1H, H3), 3.30 (dd, *J* = 10.0, 3.9 Hz, 1H, H5), 3.36 (dd, *J* = 10.1, 2.7 Hz, 1H, H5), 3.39-3.43 (m, 1H, H5), 3.63-3.70 (m, 3H, $\underline{C}H_2$OTMP), 3.74 (dd, *J* = 9.2, 5.4 Hz, 1H, $\underline{C}H_2$OTMP), 4.03-4.14 (m, 2H, C$\underline{H}$OH), 4.66 (br. s, 1H, OH), 5.41 (br. s, 1H, OH); ^{13}C NMR (101 MHz, $CDCl_3$): δ 17.1 (t, 2C, piperidine-C4), 20.2 (q, 2C, NC$\underline{C}H_3$), 20.3 (q, 2C, NC$\underline{C}H_3$), 23.2 (q, CH_3), 24.5 (q, CH_3), 33.1 (q, NC$\underline{C}H_3$), 33.16 (q, NC$\underline{C}H_3$), 33.22 (q, NC$\underline{C}H_3$), 33.5 (q, NC$\underline{C}H_3$), 34.3 (t, $\underline{C}H_2$CHOH), 35.8 (t, $\underline{C}H_2$CHOH), 35.9 (d, 2C, C4), 39.7 (t, piperidine-C3, C5), 39.8 (t, piperidine-C3, C5), 41.2 (d, C3), 44.8 (d, C3), 45.61 (t, $\underline{C}H_2$CH=), 45.81 (t, $\underline{C}H_2$CH=), 48.80 (t, C5), 48.9 (t, C5), 59.98 (s, 2C, CNO), 60.04 (s, 2C, CNO), 66.0 (d, CHOH), 68.3 (d, CHOH), 74.6 (t, $\underline{C}H_2$OTMP), 75.3 (t, $\underline{C}H_2$OTMP), 118.5 (t, CH=$\underline{C}H_2$), 118.8 (t, CH=$\underline{C}H_2$), 132.11 (d, $\underline{C}$H=CH_2), 132.4 (d, $\underline{C}$H=CH_2), 176.4 (s, C2), 177.0 (s, C2).

(3*R,4*R**)- and (3*S**,4*R**)-1-Allyl-3-(2-hydroxyhexyl)-4-(((2,2,6,6-tetramethylpiperidin-1-yl)oxy)methyl)pyrrolidin-2-one (12c):**

Prepared according to the general procedure, yield 220 mg (86%) as an inseparable 2:2(*trans*):1:1(*cis*) mixture of diastereomers. After equilibration the diastereomeric ratio for *trans*-**12c** increased to 16:16(*trans*):1:1(*cis*).

[R_f (hexanes/EtOAc 1:1) = 0.56]; IR (film); ν [cm^{-1}]: 3366 (br), 2930 (s), 2871 (m), 1670 (s), 1490 (w), 1448 (m), 1417 (w), 1374 (w), 1359 (w), 1262 (m), 1208 (w), 1185 (w), 1113 (w), 1046 (w), 994 (w), 972 (w), 956 (w), 925 (w), 790 (w), 717 (w), 620 (w), 605 (w); MS (+ESI)

m/z, (%): 811 (60, [2M+Na$^+$], 417 (100, [M+Na$^+$]), 395 (90, [M+H$^+$]); HRMS (+ESI) m/z [$C_{23}H_{42}N_2O_3Na^+$]: calcd. 417.3088; found 417.3084.

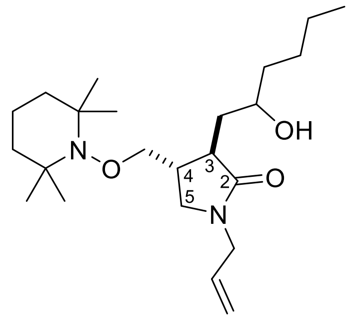

(3*R**,4*R**)

Major diastereomers: ^{1}H NMR (400 MHz, $CDCl_3$): δ 0.89 (t, *J* = 7.1 Hz, 6H, CH_3), 1.09 (s, 12H, $NCCH_3$), 1.13 (s, 3H, $NCCH_3$), 1.14 (s, 9H, $NCCH_3$), 1.28-1.37 (m, 8H, $CH_3C\underline{H}_2C\underline{H}_2$, piperidine-H4), 1.39-1.47 (m, 12H, $CH_3CH_2C\underline{H}_2C\underline{H}_2$, piperidine-H3, H5), 1.49-1.60 (m, 4H, $CH_3CH_2CH_2C\underline{H}_2$, piperidine-H4), 1.61-1.72 (m, 2H, $CHC\underline{H}_2CH$), 1.73-1.84 (m, 1H, $CHC\underline{H}_2CH$), 1.89 (ddd, *J* = 14.5, 8.7, 3.4 Hz, 1H, $CHC\underline{H}_2CH$), 2.19-2.29 (m, 1H, H4), 2.30-2.43 (m, 1H, H4), 2.53-2.66 (m, 2H, H3), 3.11-3.23 (m, 2H, H5), 3.41 (dd, *J* = 12.9, 8.8 Hz, 1H, H5), 3.42 (dd, *J* = 11.6, 6.7 Hz, 1H, H5), 3.69-3.78 (m, 1H, $C\underline{H}OH$), 3.80-3.87 (m, 7H, $C\underline{H}_2CH$=, $C\underline{H}_2$OTMP, $C\underline{H}OH$), 3.95 (dd, *J* = 15.6, 6.1 Hz, 2H, $C\underline{H}_2CH$=), 4.05 (br. s, 1H, OH), 5.15-5.24 (m, 4H, CH=$C\underline{H}_2$), 5.60 (br. s, 1H, OH), 5.72 (ddt, *J* = 16.5, 10.3, 6.1 Hz, 2H, $C\underline{H}$=CH_2); ^{13}C NMR (101 MHz, $CDCl_3$): δ 14.20 (q, CH_3), 14.22 (q, CH_3), 17.1 (t, 2C, piperidine-C4), 20.34 (q, 4C, $NC\underline{C}H_3$), 22.90 (t, $CH_3\underline{C}H_2$), 22.94 (t, $CH_3\underline{C}H_2$), 28.1 (t, $CH_3CH_2\underline{C}H_2$), 28.4 (t, $CH_3CH_2\underline{C}H_2$), 33.21 (q, $NC\underline{C}H_3$), 33.29 (q, 2C, $NC\underline{C}H_3$), 33.31 (q, $NC\underline{C}H_3$), 37.0 (t, $CH_3CH_2CH_2\underline{C}H_2$), 37.8 (t, $CH\underline{C}H_2CH$), 38.06 (d, C4), 38.09 (t, $CH_3CH_2CH_2\underline{C}H_2$), 38.9 (t, $CH\underline{C}H_2CH$), 39.1 (d, C4), 39.7 (t, piperidine-C3, C5), 39.8 (t, piperidine-C3, C5), 42.4 (d, C3), 45.5 (t, $\underline{C}H_2CH$=), 45.62 (d, C3), 45.7 (t, $\underline{C}H_2CH$=), 48.7 (t, C5), 48.79 (t, C5), 60.1 (s, 4C, CNO), 69.9 (d, CHOH), 71.9 (d, CHOH), 76.8 (t, $\underline{C}H_2$OTMP), 77.6 (t, $\underline{C}H_2$OTMP), 118.2 (t, CH=$\underline{C}H_2$), 118.37 (t, CH=$\underline{C}H_2$), 132.0 (d, $\underline{C}H$=CH_2), 132.2 (d, $\underline{C}H$=CH_2), 176.86 (s, C2), 177.4 (s, C2).

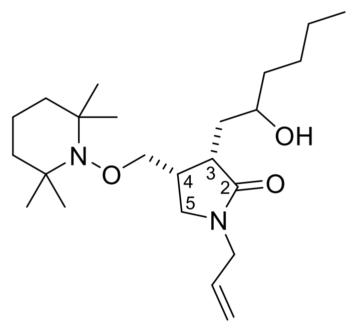

(3*S**,4*R**)

Minor diastereomers: ^{1}H NMR (400 MHz, $CDCl_3$, detectable signals): δ 2.62-2.71 (m, 2H, H4), 2.74 (td, *J* = 9.2, 4.1 Hz, 1H, H3), 2.83 (td, *J* = 9.3, 4.5 Hz, 1H, H3), 3.26 (dd, *J* = 10.0, 4.0 Hz, 1H, H5), 3.57-3.68 (m, 3H, C<u>H</u>$_2$OTMP), 3.69-3.74 (m, 1H, C<u>H</u>OH), 3.71 (dd, *J* = 9.3, 5.5 Hz, 1H, C<u>H</u>$_2$OTMP); ^{13}C NMR (101 MHz, $CDCl_3$): δ 14.20 (q, CH_3), 14.22 (q, CH_3), 17.1 (t, 2C, piperidine-C4), 20.2 (q, NC<u>C</u>H$_3$), 20.29 (q, NC<u>C</u>H$_3$), 20.34 (q, 2C, NC<u>C</u>H$_3$), 22.90 (t, CH$_3$<u>C</u>H$_2$), 22.94 (t, CH$_3$<u>C</u>H$_2$), 28.0 (t, CH$_3$CH$_2$<u>C</u>H$_2$), 28.3 (t, CH$_3$CH$_2$<u>C</u>H$_2$), 32.7 (t, CH$_3$CH$_2$CH$_2$<u>C</u>H$_2$), 33.1 (q, NC<u>C</u>H$_3$), 33.17 (q, NC<u>C</u>H$_3$), 33.22 (q, NC<u>C</u>H$_3$), 33.30 (q, NC<u>C</u>H$_3$), 34.0 (t, CH$_3$CH$_2$CH$_2$<u>C</u>H$_2$), 36.0 (d, 2C, C4), 37.1 (t, CH<u>C</u>H$_2$CH), 38.3 (t, CH<u>C</u>H$_2$CH), 39.7 (t, piperidine-C3, C5), 39.8 (t, piperidine-C3, C5), 41.1 (d, C3), 44.7 (d, C3), 45.59 (t, <u>C</u>H$_2$CH=), 45.8 (t, <u>C</u>H$_2$CH=), 48.76 (t, C5), 48.9 (t, C5), 59.99 (s, 2C, CNO), 60.04 (s, 2C, CNO), 69.9 (d, CHOH), 72.0 (d, CHOH), 74.8 (t, <u>C</u>H$_2$OTMP), 75.3 (t, <u>C</u>H$_2$OTMP), 118.42 (t, CH=<u>C</u>H$_2$), 118.7 (t, CH=<u>C</u>H$_2$), 132.2 (d, <u>C</u>H=CH$_2$), 132.4 (d, <u>C</u>H=CH$_2$), 176.90 (s, C2), 177.1 (s, C2).

(3*R,4*R**)- and (3*S**,4*R**)-1-Allyl-3-(2-hydroxy-2-phenylethyl)-4-(((2,2,6,6-tetramethyl-piperidin-1-yl)oxy)methyl)pyrrolidin-2-one (12d):**

Prepared according to the general procedure, yield 231 mg (86%) as an inseparable 3:3(*trans*):1:1(*cis*) mixture of diastereomers. After equilibration the diastereomeric ratio for *trans*-**12d** increased to 13:13(*trans*):1:1(*cis*).

[R_f (hexanes/EtOAc 1:1) = 0.49]; IR (film); ν [cm^{-1}]: 3276 (br), 2974 (w), 2930 (m), 2871 (w), 1667 (s), 1492 (w), 1451 (m), 1418 (w), 1374 (w), 1360 (w), 1262 (w), 1133 (w), 1047 (w), 993 (w), 926 (w), 757 (w), 701 (m), 669 (w), 627 (w); MS (+ESI) m/z, (%): 851 (20, [2M+Na$^+$], 437 (60, [M+Na$^+$]), 415 (100, [M+H$^+$]); HRMS (+ESI) m/z [$C_{25}H_{39}N_2O_3^+$]: calcd. 415.2955; found 415.2951.

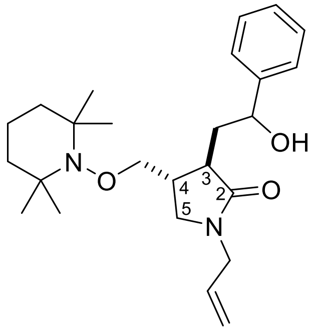

(3*R**,4*R**)

Major diastereomers: ^{1}H NMR (400 MHz, $CDCl_3$): δ 0.95 (s, 6H, $NCCH_3$), 1.03 (s, 3H, $NCCH_3$), 1.06 (s, 3H, $NCCH_3$), 1.10 (s, 3H, $NCCH_3$), 1.11 (s, 3H, $NCCH_3$), 1.15 (s, 3H, $NCCH_3$), 1.16 (s, 3H, $NCCH_3$), 1.24-1.36 (m, 2H, piperidine-H4), 1.37-1.48 (m, 8H, piperidine-H3, H5), 1.49-1.59 (m, 2H, piperidine-H4), 1.89-2.03 (m, 2H, CH_2CHOH), 2.10 (ddd, *J* = 14.5, 5.7, 3.7 Hz, 1H, CH_2CHOH), 2.15-2.33 (m, 3H, H4, CH_2CHOH), 2.39 (td, *J* = 9.5, 3.7 Hz, 1H, H3), 2.80 (td, *J* = 9.7, 3.7 Hz, 1H, H3), 3.15 (dd, *J* = 9.9, 8.2 Hz, 1H, H5), 3.25 (dd, *J* = 9.9, 8.8 Hz, 1H, H5), 3.39 (dd, *J* = 14.5, 3.5 Hz, 1H, H5), 3.44 (dd, *J* = 14.5, 3.9 Hz, 1H, H5), 3.60 (dd, *J* = 9.2, 6.2 Hz, 1H, CH_2OTMP), 3.66 (dd, *J* = 9.2, 5.3 Hz, 1H, CH_2OTMP), 3.82 (dd, *J* = 15.2, 6.2 Hz, 1H, CH_2CH=), 3.85 (d, *J* = 5.6 Hz, 2H, CH_2OTMP), 3.87 (dd, *J* = 14.9, 5.9 Hz, 1H, CH_2CH=), 3.92-4.05 (m, 2H, CH_2CH=), 4.88 (dd, *J* = 9.1, 3.7 Hz, 1H, CHOH), 5.09-5.11 (m, 1H, CHOH), 5.16-5.25 (m, 4H, $CH=CH_2$), 5.65-5.81 (m, 2H, $CH=CH_2$), 6.34 (br. s, 2H, OH), 7.18-7.25 (m, 2H, ArH), 7.29-7.35 (m, 6H, ArH), 7.36-7.43 (m, 2H, ArH); ^{13}C NMR (101 MHz, $CDCl_3$): δ 17.10 (t, piperidine-C4), 17.14 (t, piperidine-C4), 20.2 (q, $NCCH_3$), 20.3 (q, $NCCH_3$), 20.42 (q, 2C, $NCCH_3$), 33.18 (q, $NCCH_3$), 33.23 (q, $NCCH_3$), 33.3 (q, $NCCH_3$), 33.4 (q, $NCCH_3$), 38.7 (d, C4), 39.2 (d, C4), 39.5 (t, CH_2CHOH), 39.7 (t, 4C, piperidine-C3, C5), 41.8 (d, C3), 42.0 (t, CH_2CHOH), 45.67 (d, C3), 45.70 (t, CH_2CH=), 45.76 (t, CH_2CH=), 48.81 (t, C5), 49.1 (t, C5), 60.00 (s, 2C, CNO), 60.2 (s, 2C, CNO), 72.0 (d, CHOH), 74.7 (d, CHOH), 76.3 (t, CH_2OTMP), 76.7 (t, CH_2OTMP), 118.4 (t, $CH=CH_2$), 118.6 (t, $CH=CH_2$), 125.8 (d, CH_{Ar}), 125.9 (d, CH_{Ar}), 126.9 (d, CH_{Ar}), 127.2 (d, CH_{Ar}), 128.38 (d, CH_{Ar}), 128.43 (d, CH_{Ar}), 131.9 (d, $CH=CH_2$), 132.0 (d, $CH=CH_2$), 144.53 (s, C_{Ar}), 145.7 (s, C_{Ar}), 177.00 (s, C2), 177.4 (s, C2).

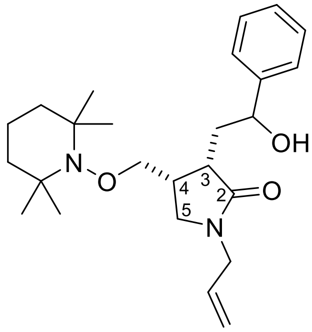

(3S*,4R*)

Minor diastereomers: ^{1}H NMR (400 MHz, $CDCl_3$, detectable signals): δ 1.01 (s, 3H, $NCCH_3$), 2.46 (dquint, J = 8.4, 4.7 Hz, 1H, H4), 2.55-2.62 (m, 1H, H3), 2.64-2.70 (m, 1H, H4), 2.91 (ddd, J = 9.9, 7.6, 3.3 Hz, 1H, H3), 3.73 (dd, J = 9.2, 5.3 Hz, 1H, C<u>H</u>$_2$OTMP), 4.78-4.83 (m, 1H, C<u>H</u>OH), 5.96 (br. s, 2H, OH); ^{13}C NMR (101 MHz, $CDCl_3$): δ 17.13 (t, 2C, piperidine-C4), 20.2 (q, NC<u>C</u>H$_3$), 20.3 (q, NC<u>C</u>H$_3$), 20.37 (q, NC<u>C</u>H$_3$), 20.44 (q, NC<u>C</u>H$_3$), 33.0 (q, NC<u>C</u>H$_3$), 33.08 (q, NC<u>C</u>H$_3$), 33.12 (q, NC<u>C</u>H$_3$), 33.5 (q, NC<u>C</u>H$_3$), 34.4 (t, <u>C</u>H$_2$CHOH), 35.9 (d, C4), 36.1 (d, C4), 36.9 (t, <u>C</u>H$_2$CHOH), 39.8 (t, 4C, piperidine-C3, C5), 41.0 (d, C3), 44.8 (d, C3), 45.81 (t, <u>C</u>H$_2$CH=), 45.9 (t, <u>C</u>H$_2$CH=), 48.76 (t, C5), 48.82 (t, C5), 59.9 (s, 2C, CNO), 60.03 (s, 2C, CNO), 71.8 (d, CHOH), 74.5 (t, <u>C</u>H$_2$OTMP), 74.78 (d, CHOH), 74.83 (t, <u>C</u>H$_2$OTMP), 118.8 (t, CH=<u>C</u>H$_2$), 118.9 (t, CH=<u>C</u>H$_2$), 125.9 (d, CH_{Ar}), 126.0 (d, CH_{Ar}), 127.0 (d, CH_{Ar}), 127.3 (d, CH_{Ar}), 128.38 (d, CH_{Ar}), 128.43 (d, CH_{Ar}), 132.1 (d, <u>C</u>H=CH$_2$), 132.2 (d, <u>C</u>H=CH$_2$), 144.51 (s, C_{Ar}), 145.5 (s, C_{Ar}), 176.97 (s, C2), 177.2 (s, C2).

(3*R*,4*R***)- and (3*S***,4*R***)-3-(2-Hydroxypropyl)-1-methyl-4-(((2,2,6,6-tetramethylpiperidin-1-yl)oxy)methyl)pyrrolidin-2-one (12e):**

Prepared according to the general procedure, yield 214 mg (82%) as an inseparable 2:2(*trans*):1:1(*cis*) mixture of diastereomers. After equilibration the diastereomeric ratio for *trans*-**12e** increased to 4:4(*trans*):1:1(*cis*).

[R_f (hexanes/EtOAc 1:1) = 0.25]; IR (film); ν [cm^{-1}]: 3351 (br), 2970 (w), 2928 (m), 2871 (w), 1669 (s), 1501 (w), 1468 (w), 1453 (w), 1404 (w), 1373 (w), 1359 (w), 1263 (w), 1245 (w), 1133 (w), 1096 (w), 1078 (w), 1046 (w), 994 (w), 793 (w), 721 (w), 636 (w); MS (+ESI) m/z, (%): 349 (100, [$M+Na^+$]), 327 (10, [$M+H^+$]); HRMS (+ESI) m/z [$C_{18}H_{34}N_2O_3Na^+$]: calcd. 349.2462; found 349.2460.

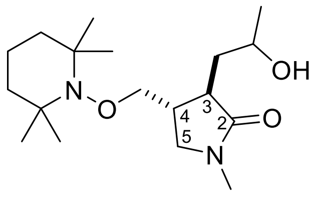

(3*R**,4*R**)

Major diastereomers: ^{1}H NMR (400 MHz, CDCl$_3$): δ 1.09 (s, 12H, NCCH$_3$), 1.14 (s, 12H, NCCH$_3$), 1.21 (d, *J* = 6.6 Hz, 3H, CHC$\underline{H}_3$), 1.23 (d, *J* = 6.2 Hz, 3H, CHC$\underline{H}_3$), 1.28-1.36 (m, 2H, piperidine-H4), 1.40-1.47 (m, 8H, piperidine-H3, H5), 1.50-1.58 (m, 2H, piperidine-H4), 1.59-1.67 (m, 1H, C$\underline{H}_2$CHOH), 1.68-1.80 (m, 2H, C$\underline{H}_2$CHOH), 1.90 (ddd, *J* = 14.5, 8.7, 3.6 Hz, 1H, C$\underline{H}_2$CHOH), 2.16-2.24 (m, 1H, H4), 2.28-2.42 (m, 1H, H4), 2.53 (td, *J* = 9.8, 3.2 Hz, 1H, H3), 2.62 (td, *J* = 8.7, 5.0 Hz, 1H, H3), 2.85 (s, 3H, NCH$_3$), 2.86 (s, 3H, NCH$_3$), 3.21 (dd, *J* = 9.1, 4.5 Hz, 1H, H5), 3.23 (dd, *J* = 13.2, 7.9 Hz, 1H, H5), 3.43 (dd, *J* = 9.1, 8.2 Hz, 1H, H5), 3.44 (dd, *J* = 13.2, 3.5 Hz, 1H, H5), 3.79-3.84 (m, 4H, C$\underline{H}_2$OTMP), 3.88-4.02 (m, 1H, C$\underline{H}$OH), 4.04-4.12 (m, 1H, C$\underline{H}$OH), 5.82 (br. s, 2H, OH); ^{13}C NMR (101 MHz, CDCl$_3$): δ 17.1 (t, 2C, piperidine-C4), 20.3 (q, 4C, NC$\underline{C}H_3$), 23.0 (q, CH$\underline{C}H_3$), 24.3 (q, CH$\underline{C}H_3$), 30.2 (q, NCH$_3$), 31.4 (q, NCH$_3$), 33.1 (q, NC$\underline{C}H_3$), 33.39 (q, NC$\underline{C}H_3$), 33.42 (q, NC$\underline{C}H_3$), 33.5 (q, NC$\underline{C}H_3$), 34.3 (t, $\underline{C}H_2$CHOH), 35.86 (t, $\underline{C}H_2$CHOH), 35.88 (d, C4), 35.93 (d, C4), 39.7 (t, 2C, piperidine-C3), 39.8 (t, 2C, piperidine-C5), 40.8 (d, C3), 44.6 (d, C3), 51.62 (t, C5), 51.7 (t, C5), 59.96 (s, CNO), 59.97 (s, CNO), 59.99 (s, CNO), 60.01 (s, CNO), 65.9 (d, CHOH), 68.3 (d, CHOH), 74.9 (t, $\underline{C}H_2$OTMP), 75.3 (t, $\underline{C}H_2$OTMP), 177.08 (s, C2), 177.6 (s, C2).

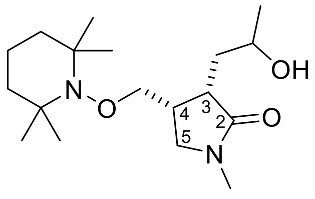

(3*S**,4*R**)

Minor diastereomers: ^{1}H NMR (400 MHz, CDCl$_3$, detectable signals): δ 1.11 (s, 12H, NCCH$_3$), 1.13 (s, 12H, NCCH$_3$), 1.80-1.85 (m, 1H, C$\underline{H}_2$CHOH), 2.65-2.70 (m, 2H, H4), 2.74 (td, *J* = 8.7, 4.6 Hz, 1H, H3), 2.78-2.86 (m, 1H, H3), 2.87 (s, 3H, NCH$_3$), 3.32 (dd, *J* = 9.9, 3.9 Hz, 1H, H5), 3.37 (dd, *J* = 10.1, 2.6 Hz, 1H, H5), 3.67 (dd, *J* = 9.2, 6.0 Hz, 1H, C$\underline{H}_2$OTMP), 3.74 (dd, *J* = 9.1, 5.3 Hz, 1H, C$\underline{H}_2$OTMP), 3.82-3.92 (m, 1H, C$\underline{H}$OH), 4.38 (br. s, 1H, OH), 5.54 (br. s, 1H, OH); ^{13}C NMR (101 MHz, CDCl$_3$): δ 17.1 (t, 2C, piperidine-C4), 20.3 (q, 4C, NC$\underline{C}H_3$), 23.1 (q, CH$\underline{C}H_3$), 24.4 (q, CH$\underline{C}H_3$), 30.0 (q, NCH$_3$), 30.1 (q, NCH$_3$), 33.2 (q, 2C, NC$\underline{C}H_3$), 33.3 (q, 2C,

$NCCH_3$), 38.1 (d, C4), 38.9 (d, C4), 39.4 (t, CH_2CHOH), 39.7 (t, 2C, piperidine-C3), 39.8 (t, 2C, piperidine-C5), 40.9 (t, CH_2CHOH), 41.9 (d, C3), 45.6 (d, C3), 51.5 (t, C5), 51.56 (t, C5), 60.1 (s, 2C, CNO), 60.2 (s, 2C, CNO), 66.0 (d, CHOH), 68.2 (d, CHOH), 76.9 (t, CH_2OTMP), 77.4 (t, CH_2OTMP), 177.12 (s, C2), 177.6 (s, C2).

(3*R,4*R**)- and (3*S**,4*R**)-1-Benzyl-3-(2-hydroxypropyl)-4-methyl-4-(((2,2,6,6-tetramethyl-piperidin-1-yl)oxy)methyl)pyrrolidin-2-one (12f):**

Prepared according to the general procedure, yield 178 mg (66%) as an inseparable 3:3(*trans*):1:1(*cis*) mixture of diastereomers. After equilibration the diastereomeric ratio for *trans*-**12f** did not change.

[R_f (hexanes/EtOAc 1:1) = 0.56]; IR (film); ν [cm^{-1}]: 3341 (br), 2969 (m), 2928 (m), 2872 (m), 1667 (s), 1490 (w), 1452 (m), 1373 (w), 1359 (w), 1322 (w), 1260 (w), 1208 (w), 1153 (w), 1132 (w), 1054 (w), 1030 (m), 994 (w), 954 (w), 925 (w), 726 (w), 701 (w), 646 (w), 620 (w); MS (+ESI) m/z, (%): 417 (100, [$M+H^+$]); HRMS (+ESI) m/z [$C_{25}H_{41}N_2O_3^+$]: calcd. 417.3112; found 417.3111.

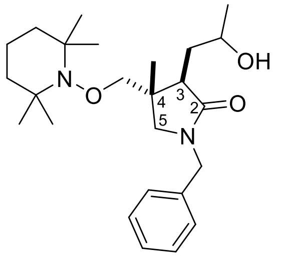

(3*R**,4*R**)

Major diastereomers: ^1H NMR (400 MHz, $CDCl_3$): δ 0.89 (s, 3H, CCH_3), 0.92 (s, 3H, CCH_3), 1.03 (s, 3H, $NCCH_3$), 1.04 (s, 3H, $NCCH_3$), 1.07 (s, 18H, $NCCH_3$), 1.23 (d, *J* = 6.5 Hz, 6H, $CHCH_3$), 1.25-1.33 (m, 2H, piperidine-H4), 1.34-1.44 (m, 8H, piperidine-H3, H5), 1.46-1.51 (m, 2H, piperidine-H4), 1.57-1.62 (m, 1H, CH_2CHOH), 1.63-1.68 (m, 2H, CH_2CHOH), 1.88 (ddd, *J* = 14.7, 11.4, 3.5 Hz, 1H, CH_2CHOH), 2.78 (d, *J* = 9.5 Hz, 1H, H5), 2.79-2.83 (m, 1H, H3), 2.81 (d, *J* = 9.7 Hz, 1H, H5), 2.92 (dd, *J* = 9.8, 6.5 Hz, 1H, H3), 3.39 (d, *J* = 9.5 Hz, 1H, H5), 3.43 (d, *J* = 9.7 Hz, 1H, H5), 3.57 (d, *J* = 9.0 Hz, 1H, CH_2OTMP), 3.58 (d, *J* = 9.1 Hz, 1H, CH_2OTMP), 3.61 (d, *J* = 9.0 Hz, 1H, CH_2OTMP), 3.62 (d, *J* = 9.1 Hz, 1H, CH_2OTMP), 3.89-3.98 (m, 1H, CHOH), 4.16-4.26 (m, 1H, CHOH), 4.23 (d, *J* = 14.7 Hz, 1H, CH_2Ph), 4.24 (d, *J* = 14.9 Hz, 1H, CH_2Ph), 4.66 (d, *J* = 14.7 Hz, 1H, CH_2Ph), 4.67 (d, *J* = 14.9 Hz, 1H, CH_2Ph), 6.13 (br. s, 2H, OH), 7.20-7.37 (m, 10H, ArH); ^{13}C NMR (101 MHz, $CDCl_3$): δ 17.04 (t, piperidine-C4), 17.07

(t, piperidine-C4), 18.9 (q, 2C, $C\underline{C}H_3$), 20.3 (q, $NC\underline{C}H_3$), 20.4 (q, $NC\underline{C}H_3$), 20.48 (q, $NC\underline{C}H_3$), 20.53 (q, $NC\underline{C}H_3$), 22.8 (q, $CH\underline{C}H_3$), 24.7 (q, $CH\underline{C}H_3$), 33.17 (q, $NC\underline{C}H_3$), 33.24 (q, $NC\underline{C}H_3$), 33.3 (q, $NC\underline{C}H_3$), 33.4 (q, $NC\underline{C}H_3$), 33.6 (t, $\underline{C}H_2$CHOH), 35.3 (t, $\underline{C}H_2$CHOH), 39.8 (t, piperidine-C3, C5), 39.9 (t, piperidine-C3, C5), 41.1 (s, C4), 41.4 (s, C4), 43.9 (d, C3), 46.98 (t, C5), 47.04 (t, C5), 49.2 (d, C3), 55.2 (t, $\underline{C}H_2$Ph), 55.3 (t, $\underline{C}H_2$Ph), 60.3 (s, 4C, CNO), 65.2 (d, CHOH), 68.76 (d, CHOH), 79.9 (t, $\underline{C}H_2$OTMP), 80.2 (t, $\underline{C}H_2$OTMP), 127.8 (d, CH_{Ar}), 127.9 (d, CH_{Ar}), 128.28 (d, CH_{Ar}), 128.29 (d, CH_{Ar}), 128.88 (d, CH_{Ar}), 128.89 (d, CH_{Ar}), 136.04 (s, C_{Ar}), 136.3 (s, C_{Ar}), 176.9 (s, C2), 177.4 (s, C2).

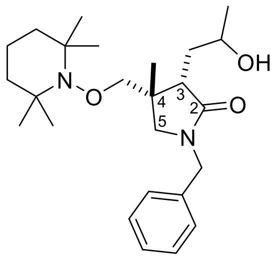

(3*S**,4*R**)

Minor diastereomers: ^{1}H NMR (400 MHz, $CDCl_3$, detectable signals): δ 0.86 (s, 3H, CCH_3), 0.98 (s, 3H, $NCCH_3$), 0.99 (s, 3H, $NCCH_3$), 1.13 (s, 3H, $NCCH_3$), 1.19 (d, J = 6.1 Hz, 3H, $CHC\underline{H}_3$), 1.21 (d, J = 6.2 Hz, 3H, $CHC\underline{H}_3$), 1.80 (ddd, J = 14.7, 11.0, 3.7 Hz, 1H, $C\underline{H}_2$CHOH), 2.33-2.44 (m, 1H, H3), 2.54 (dd, J = 11.0, 3.2 Hz, 1H, H3), 2.93 (d, J = 9.8 Hz, 1H, H5), 3.03 (d, J = 9.7 Hz, 1H, H5), 3.44 (d, J = 9.8 Hz, 1H, H5), 3.49 (d, J = 9.7 Hz, 1H, $C\underline{H}_2$OTMP), 3.51 (d, J = 9.7 Hz, 1H, H5), 3.57 (d, J = 9.9 Hz, 1H, $C\underline{H}_2$OTMP), 3.83-3.89 (m, 1H, $C\underline{H}$OH), 4.27 (d, J = 14.3 Hz, 1H, $C\underline{H}_2$Ph), 4.28 (d, J = 14.4 Hz, 1H, $C\underline{H}_2$Ph), 4.57 (d, J = 14.4 Hz, 1H, $C\underline{H}_2$Ph), 4.58 (d, J = 14.3 Hz, 1H, $C\underline{H}_2$Ph), 5.97 (br. s, 2H, OH); ^{13}C NMR (101 MHz, $CDCl_3$): δ 17.00 (t, piperidine-C4), 17.13 (t, piperidine-C4), 20.3 (q, $NC\underline{C}H_3$), 20.4 (q, $NC\underline{C}H_3$), 20.48 (q, $NC\underline{C}H_3$), 20.53 (q, $NC\underline{C}H_3$), 22.0 (q, $CH\underline{C}H_3$), 22.3 (q, $CH\underline{C}H_3$), 22.7 (q, $C\underline{C}H_3$), 22.9 (q, $C\underline{C}H_3$), 32.6 (t, $\underline{C}H_2$CHOH), 33.17 (q, $NC\underline{C}H_3$), 33.24 (q, $NC\underline{C}H_3$), 33.3 (q, $NC\underline{C}H_3$), 33.4 (q, $NC\underline{C}H_3$), 34.2 (t, $\underline{C}H_2$CHOH), 39.8 (t, piperidine-C3, C5), 39.9 (t, piperidine-C3, C5), 41.0 (s, C4), 41.2 (s, C4), 47.3 (t, C5), 47.4 (t, C5), 48.0 (d, C3), 48.7 (d, C3), 54.6 (t, $\underline{C}H_2$Ph), 54.7 (t, $\underline{C}H_2$Ph), 59.0 (s, 4C, CNO), 65.0 (d, CHOH), 68.84 (d, CHOH), 77.1 (t, $\underline{C}H_2$OTMP), 77.3 (t, $\underline{C}H_2$OTMP), 127.7 (d, CH_{Ar}), 128.0 (d, CH_{Ar}), 128.4 (d, CH_{Ar}), 128.7 (d, CH_{Ar}), 128.8 (d, CH_{Ar}), 129.0 (d, CH_{Ar}), 136.00 (s, C_{Ar}), 136.1 (s, C_{Ar}), 177.2 (s, C2), 177.3 (s, C2).

(3*R,4*S**)- and (3*S**,4*S**)-1-Benzyl-3-(2-hydroxypropyl)-4-(prop-1-en-2-yl)pyrrolidin-2-one (12g):**

Prepared according to the general procedure, yield 165 mg (93%) as an inseparable 2:2(*trans*):1:1(*cis*) mixture of diastereomers. After equilibration the diastereomeric ratio for *trans*-**12g** increased to 7:7(*trans*):1:0(*cis*).

[R_f (hexanes/EtOAc 1:1) = 0.43]; IR (film); ν [cm^{-1}]: 3367 (br), 3076 (w), 3031 (w), 2966 (w), 2926 (w), 1666 (s), 1495 (w), 1435 (m), 1376 (w), 1321 (w), 1255 (m), 1202 (w), 1138 (w), 1079 (w), 1057 (w), 1029 (w), 939 (w), 899 (w), 820 (w), 750 (w), 701 (m), 646 (w); MS (+ESI) m/z, (%): 569 (30, [$2M+Na^+$]), 296 (100, [$M+Na^+$]), 274 (30, [$M+H^+$]); HRMS (+ESI) m/z [$C_{17}H_{23}NO_2Na^+$]: calcd. 296.1621; found 296.1619.

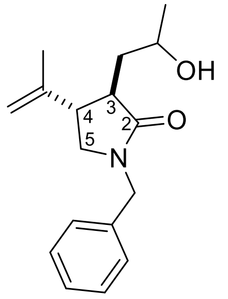

(3*R**,4*S**)

Major diastereomers: ^{1}H NMR (400 MHz, $CDCl_3$): δ 1.22 (d, *J* = 6.2 Hz, 6H, $CHC\underline{H}_3$), 1.62-1.72 (m, 3H, $C\underline{H}_2CHOH$), 1.68 (s, 6H, $=CCH_3$), 1.87 (ddd, *J* = 14.5, 9.1, 3.5 Hz, 1H, $C\underline{H}_2CHOH$), 2.57-2.72 (m, 3H, H3, H4), 2.77 (td, *J* = 9.7, 3.9 Hz, 1H, H3), 3.08 (dd, *J* = 9.8, 4.2 Hz, 1H, H5), 3.10 (dd, *J* = 9.5, 4.7 Hz, 1H, H5), 3.29 (dd, *J* = 9.5, 8.4 Hz, 1H, H5), 3.30 (dd, *J* = 9.8, 8.2 Hz, 1H, H5), 3.95 (sext, *J* = 6.0 Hz, 1H, C$\underline{H}$OH), 4.08-4.20 (m, 1H, C$\underline{H}$OH), 4.39 (d, *J* = 14.5 Hz, 1H, $C\underline{H}_2Ph$), 4.40 (d, *J* = 14.7 Hz, 1H, $C\underline{H}_2Ph$), 4.51 (d, *J* = 14.7 Hz, 1H, $C\underline{H}_2Ph$), 4.52 (d, *J* = 14.5 Hz, 1H, $C\underline{H}_2Ph$), 4.82 (d, *J* = 1.7 Hz, 1H, $C=CH_2$), 4.83 (d, *J* = 1.7 Hz, 1H, $C=CH_2$), 4.85 (d, *J* = 1.5 Hz, 1H, $C=CH_2$), 4.86 (d, *J* = 1.5 Hz, 1H, $C=CH_2$), 5.71 (br. s, 2H, OH), 7.20-7.25 (m, 4H, Ar$\underline{H}$), 7.28-7.36 (m, 6H, Ar$\underline{H}$); ^{13}C NMR (101 MHz, $CDCl_3$): δ 19.5 (q, $CH\underline{C}H_3$), 19.6 (q, $CH\underline{C}H_3$), 23.1 (q, $=C\underline{C}H_3$), 24.4 (q, $=C\underline{C}H_3$), 38.2 (t, $\underline{C}H_2CHOH$), 40.0 (t, $\underline{C}H_2CHOH$), 42.0 (d, C4), 45.9 (d, C4), 46.8 (d, C3), 46.94 (t, $\underline{C}H_2Ph$), 47.1 (t, $\underline{C}H_2Ph$), 47.6 (d, C3), 49.87 (t, C5), 49.94 (t, C5), 65.3 (d, CHOH), 68.0 (d, CHOH), 113.4 (t, $C=\underline{C}H_2$), 113.9 (t, $C=\underline{C}H_2$), 127.86 (d, CH_{Ar}), 127.94 (d, CH_{Ar}), 128.25 (d, CH_{Ar}), 128.28 (d, CH_{Ar}), 128.90 (d, CH_{Ar}), 128.94 (d, CH_{Ar}), 136.0 (s, C_{Ar}), 136.17 (s, C_{Ar}), 142.1 (s, $\underline{C}=CH_2$), 142.6 (s, $\underline{C}=CH_2$), 176.9 (s, C2), 177.3 (s, C2).

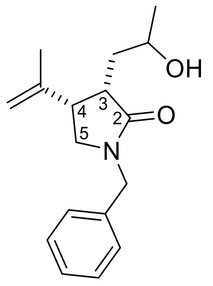

(3*S**,4*S**)

Minor diastereomers: ^{1}H NMR (400 MHz, $CDCl_3$): δ 1.20 (d, *J* = 6.4 Hz, 3H, $CHC\underline{H}_3$), 1.21 (d, *J* = 6.1 Hz, 3H, $CHC\underline{H}_3$), 1.38-1.46 (m, 1H, $C\underline{H}_2CHOH$), 1.49 (s, 3H, $=CCH_3$), 1.52-1.56 (m, 2H, $C\underline{H}_2CHOH$), 1.57 (s, 3H, $=CCH_3$), 1.60-1.68 (m, 1H, $C\underline{H}_2CHOH$), 2.76-2.83 (m, 1H, H4), 2.86-2.93 (m, 1H, H4), 2.94-3.05 (m, 2H, H3), 3.13 (dd, *J* = 10.5, 3.2 Hz, 1H, H5), 3.20 (dd, *J* = 10.0, 5.7 Hz, 1H, H5), 3.32 (dd, *J* = 10.5, 5.7 Hz, 1H, H5), 3.41 (dd, *J* = 10.0, 6.8 Hz, 1H, H5), 3.89-4.00 (m, 1H, $C\underline{H}OH$), 4.03-4.10 (m, 1H, $C\underline{H}OH$), 4.45 (s, 2H, $C\underline{H}_2Ph$), 4.46 (s, 2H, $C\underline{H}_2Ph$), 4.65 (d, *J* = 1.7 Hz, 1H, $C=CH_2$), 4.68 (d, *J* = 1.7 Hz, 1H, $C=CH_2$), 4.79 (d, *J* = 1.5 Hz, 1H, $C=CH_2$), 4.80-4.83 (m, 1H, $C=CH_2$), 5.21 (br. s, 2H, OH); ^{13}C NMR (101 MHz, $CDCl_3$): δ 20.1 (q, $CH\underline{C}H_3$), 21.1 (q, $CH\underline{C}H_3$), 22.9 (q, $=C\underline{C}H_3$), 24.4 (q, $=C\underline{C}H_3$), 34.3 (t, $\underline{C}H_2CHOH$), 35.9 (t, $\underline{C}H_2CHOH$), 41.0 (d, C4), 43.7 (d, C3), 43.8 (d, C3), 45.0 (d, C4), 46.92 (t, $\underline{C}H_2Ph$), 47.2 (t, $\underline{C}H_2Ph$), 48.9 (t, C5), 49.7 (t, C5), 65.5 (d, CHOH), 68.3 (d, CHOH), 113.4 (t, $C=\underline{C}H_2$), 114.5 (t, $C=\underline{C}H_2$), 127.89 (d, CH_{Ar}), 128.0 (d, CH_{Ar}), 128.5 (d, CH_{Ar}), 128.7 (d, CH_{Ar}), 128.87 (d, 2C, CH_{Ar}), 135.9 (s, C_{Ar}), 136.20 (s, C_{Ar}), 142.8 (s, $\underline{C}=CH_2$), 143.1 (s, $\underline{C}=CH_2$), 176.8 (s, C2), 177.5 (s, C2).

(3*R*,4*R*)- and (3*S*,4*S*)- and (3*R*,4*S*)- and (3*S*,4*R*)-1-Allyl-3-((*S*)-3-(benzyloxy)-2-hydroxypropyl)-4-(((2,2,6,6-tetramethylpiperidin-1-yl)oxy)methyl)pyrrolidin-2-one (12h):

Prepared according to the general procedure, yield 214 mg (72%) as an inseparable 4:4(*trans*):1:1(*cis*) mixture of diastereomers. After equilibration the diastereomeric ratio for *trans*-**12h** increased to 17:17(*trans*):1:1(*cis*).

[R_f (hexanes/EtOAc 1:1) = 0.61]; IR (film); ν [cm^{-1}]: 3295 (br), 2974 (w), 2929 (m), 2869 (w), 1667 (s), 1493 (w), 1452 (m), 1418 (w), 1374 (w), 1359 (w), 1262 (m), 1208 (w), 1185 (w), 1132 (m), 1101 (m), 1046 (w), 1029 (w), 993 (w), 971 (w), 956 (w), 925 (w), 736 (w), 698 (w), 606 (w); MS (+ESI) m/z, (%): 939 (50, [2M+Na$^+$], 481 (100, [M+Na$^+$]), 459 (65, [M+H$^+$]); HRMS (+ESI) m/z [$C_{27}H_{42}N_2O_4Na^+$]: calcd. 481.3037; found 481.3033.

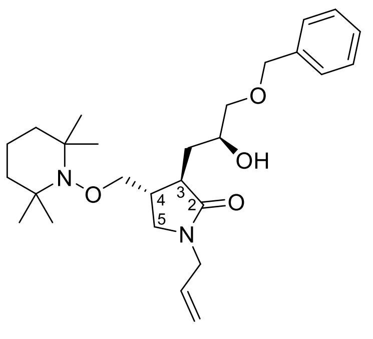

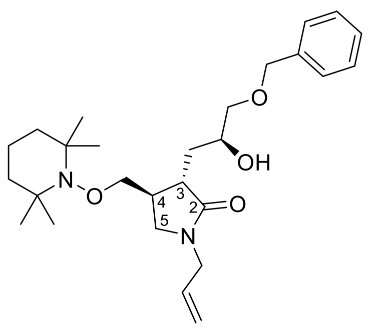

(3*R*,4*R*) (3*S*,4*S*)

Major diastereomers: ^{1}H NMR (400 MHz, $CDCl_3$): δ 1.07 (s, 12H, $NCCH_3$), 1.12 (s, 12H, $NCCH_3$), 1.28-1.36 (m, 2H, piperidine-H4), 1.39-1.47 (m, 8H, piperidine-H3, H5), 1.48-1.61 (m, 2H, piperidine-H4), 1.67 (dt, *J* = 14.5, 10.0 Hz, 1H, C$\underline{H}_2$CHOH), 1.87 (t, *J* = 6.1 Hz, 2H, C$\underline{H}_2$CHOH), 1.99 (ddd, *J* = 14.5, 3.4, 2.1 Hz, 1H, C$\underline{H}_2$CHOH), 2.21-2.31 (m, 1H, H4), 2.32-2.41 (m, 1H, H4), 2.52-2.62 (m, 2H, H3), 3.17 (dd, *J* = 10.5, 7.5 Hz, 1H, H5), 3.20 (dd, *J* = 17.9, 9.3 Hz, 1H, H5), 3.38-3.44 (m, 3H, CHC$\underline{H}_2$O, H5), 3.47 (dd, *J* = 9.3, 3.3 Hz, 1H, CHC$\underline{H}_2$O), 3.50 (dd, *J* = 9.3, 4.2 Hz, 1H, CHC$\underline{H}_2$O), 3.55 (dd, *J* = 9.5, 5.3 Hz, 1H, CHC$\underline{H}_2$O), 3.78 (dd, *J* = 9.0, 7.1 Hz, 1H, C$\underline{H}_2$OTMP), 3.81-3.88 (m, 5H, C$\underline{H}_2$OTMP, $\underline{C}H_2$CH=), 3.89-3.99 (m, 3H, $\underline{C}H_2$CH=, C$\underline{H}$OH), 4.11 (quint, *J* = 5.5 Hz, 1H, C$\underline{H}$OH), 4.54 (d, *J* = 12.0 Hz, 1H, C$\underline{H}_2$Ph), 4.55 (d, *J* = 12.0 Hz, 1H, C$\underline{H}_2$Ph), 4.56 (d, *J* = 12.4 Hz, 1H, C$\underline{H}_2$Ph), 4.58 (d, *J* = 12.4 Hz, 1H, C$\underline{H}_2$Ph), 5.15-5.24 (m, 4H, CH=C$\underline{H}_2$), 5.65 (br. s, 2H, OH), 5.73 (ddt, *J* = 16.4, 10.4, 6.2 Hz, 2H, C$\underline{H}$=CH_2), 7.26-7.37 (m, 10H, Ar$\underline{H}$); ^{13}C NMR (101 MHz, $CDCl_3$): δ 17.14 (t, piperidine-C4), 17.15 (t, piperidine-C4), 20.3 (q, 4C, NC$\underline{C}H_3$), 33.3 (q, 2C, NC$\underline{C}H_3$), 33.4 (q, 2C, NC$\underline{C}H_3$), 34.4 (t, $\underline{C}H_2$CHOH), 35.6 (t, $\underline{C}H_2$CHOH), 38.1 (d, C4), 39.0 (d, C4), 39.7 (t, piperidine-C3, C5), 39.8 (t, piperidine-C3, C5), 42.1 (d, C3), 45.1 (d, C3), 45.5 (t, $\underline{C}H_2$CH=), 45.7 (t, $\underline{C}H_2$CH=), 48.9 (t, C5), 49.0 (t, C5), 60.04 (s, 2C, CNO), 60.1 (s, 2C, CNO), 68.6 (d, CHOH), 70.7 (d, CHOH), 73.45 (t, $\underline{C}H_2$Ph), 73.47 (t, $\underline{C}H_2$Ph), 73.9 (t, CH$\underline{C}H_2$O), 74.73 (t, CH$\underline{C}H_2$O), 76.7 (t, $\underline{C}H_2$OTMP), 77.6 (t, $\underline{C}H_2$OTMP), 118.2 (t, CH=$\underline{C}H_2$), 118.4 (t, CH=$\underline{C}H_2$), 127.6 (d, CH_{Ar}), 127.72 (d, CH_{Ar}), 127.76 (d, CH_{Ar}), 127.84 (d, CH_{Ar}), 128.4 (d, CH_{Ar}), 128.52 (d, CH_{Ar}), 132.0 (d, $\underline{C}$H=CH_2), 132.23 (d, $\underline{C}$H=CH_2), 138.4 (s, C_{Ar}), 138.6 (s, C_{Ar}), 176.6 (s, C2), 177.2 (s, C2).

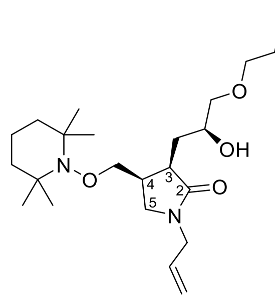

(3*R*,4*S*)

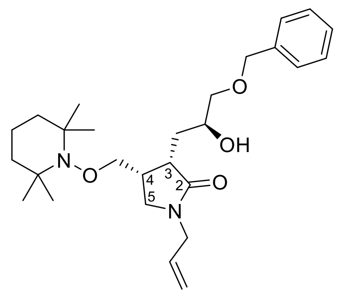

(3*S*,4*R*)

Minor diastereomers: ^{1}H NMR (400 MHz, $CDCl_3$, detectable signals): δ 1.78 (ddd, *J* = 12.8, 9.3, 3.4 Hz, 1H, C$\underline{H}_2$CHOH), 2.63-2.71 (m, 2H, H4), 2.80 (dt, *J* = 8.9, 4.6 Hz, 1H, H3), 2.87 (dd, *J* = 9.7, 2.1 Hz, 1H, H5), 2.97 (dd, *J* = 18.3, 4.7 Hz, 1H, H5), 3.10 (dd, *J* = 18.3, 4.8 Hz, 1H, H5), 3.11 (dd, *J* = 9.7, 4.4 Hz, 1H, H5), 3.31-3.40 (m, 1H, CHC$\underline{H}_2$O), 3.64 (dd, *J* = 9.5, 6.1 Hz, 1H, C$\underline{H}_2$OTMP), 3.66 (dd, *J* = 9.5, 6.2 Hz, 1H, C$\underline{H}_2$OTMP), 4.09-4.16 (m, 2H, C$\underline{H}_2$OTMP), 4.56 (d, *J* = 11.8 Hz, 1H, C$\underline{H}_2$Ph), 4.62 (d, *J* = 11.8 Hz, 1H, C$\underline{H}_2$Ph), 5.42 (br. s, 2H, OH); ^{13}C NMR (101 MHz, $CDCl_3$): δ 17.2 (t, 2C, piperidine-C4), 20.20 (q, NC$\underline{C}H_3$), 20.22 (q, NC$\underline{C}H_3$), 20.39 (q, NC$\underline{C}H_3$), 20.41 (q, NC$\underline{C}H_3$), 29.2 (t, $\underline{C}H_2$CHOH), 30.9 (t, $\underline{C}H_2$CHOH), 33.08 (q, NC$\underline{C}H_3$), 33.11 (q, NC$\underline{C}H_3$), 33.4 (q, NC$\underline{C}H_3$), 33.5 (q, NC$\underline{C}H_3$), 35.8 (d, C4), 35.9 (d, C4), 39.7 (t, piperidine-C3, C5), 39.8 (t, piperidine-C3, C5), 41.1 (d, C3), 44.3 (d, C3), 45.6 (t, $\underline{C}H_2$CH=), 45.9 (t, $\underline{C}H_2$CH=), 48.6 (t, C5), 48.8 (t, C5), 59.98 (s, CNO), 60.01 (s, CNO), 60.19 (s, CNO), 60.21 (s, CNO), 68.8 (d, CHOH), 70.8 (d, CHOH), 73.4 (t, $\underline{C}H_2$Ph), 73.6 (t, $\underline{C}H_2$Ph), 73.8 (t, CH$\underline{C}H_2$O), 74.69 (t, CH$\underline{C}H_2$O), 75.0 (t, $\underline{C}H_2$OTMP), 75.2 (t, $\underline{C}H_2$OTMP), 118.5 (t, CH=$\underline{C}H_2$), 118.8 (t, CH=$\underline{C}H_2$), 127.74 (d, CH_{Ar}), 127.81 (d, CH_{Ar}), 127.89 (d, CH_{Ar}), 127.94 (d, CH_{Ar}), 128.54 (d, CH_{Ar}), 128.7 (d, CH_{Ar}), 132.16 (d, $\underline{C}H$=CH_2), 132.4 (d, $\underline{C}H$=CH_2), 138.3 (s, C_{Ar}), 138.5 (s, C_{Ar}), 176.9 (s, C2), 177.0 (s, C2).

(3*R*,4*R*)- and (3*S*,4*S*)- and (3*R*,4*S*)- and (3*S*,4*R*)-3-((*S*)-2-Hydroxypropyl)-1-((*S*)-1-phenylethyl)-4-(((2,2,6,6-tetramethylpiperidin-1-yl)oxy)methyl)pyrrolidin-2-one (12i):

Prepared according to the general procedure, yield 228 mg (82%) as an inseparable 2:2(*trans*):1:1(*cis*) mixture of diastereomers. After equilibration the diastereomeric ratio for *trans*-**12i** increased to 1:1(*trans*):0:0(*cis*).

[R_f (hexanes/EtOAc 1:1) = 0.42]; IR (film); ν [cm^{-1}]: 3352 (br), 2973 (m), 2931 (m), 2873 (w), 1652 (s), 1493 (w), 1452 (m), 1430 (m), 1374 (w), 1359 (w), 1263 (w), 1245 (w), 1209 (w), 1186 (w), 1133 (w), 1048 (w), 994 (w), 956 (w), 786 (w), 746 (w), 699 (m), 637 (w); MS (+ESI) m/z, (%): 439 (100, [$M+Na^+$]), 417 (30, [$M+H^+$]); HRMS (+ESI) m/z [$C_{25}H_{40}N_2O_3Na^+$]: calcd. 439.2931; found 439.2931.

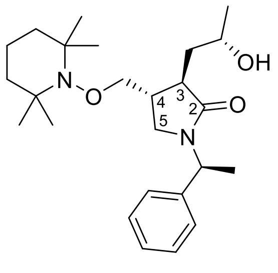

(3*R*,4*R*)

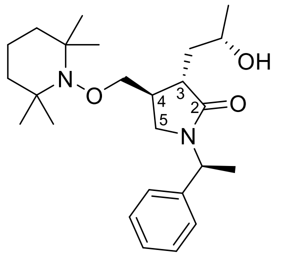

(3*S*,4*S*)

Major diastereomers: ^{1}H NMR (400 MHz, $CDCl_3$): δ 0.98 (s, 9H, $NCCH_3$), 1.04 (s, 3H, $NCCH_3$), 1.05 (s, 6H, $NCCH_3$), 1.09 (s, 3H, $NCCH_3$), 1.11 (s, 3H, $NCCH_3$), 1.21 (d, *J* = 6.5 Hz, 3H, $CHOHCH_3$), 1.22 (d, *J* = 6.2 Hz, 3H, $CHOHCH_3$), 1.28-1.35 (m, 2H, piperidine-H4), 1.37-1.46 (m, 8H, piperidine-H3, H5), 1.47-1.50 (m, 2H, piperidine-H4), 1.51 (d, *J* = 7.3 Hz, 3H, $PhCHCH_3$), 1.52 (d, *J* = 7.0 Hz, 3H, $PhCHCH_3$), 1.62-1.82 (m, 3H, CH_2CHOH), 1.91 (ddd, *J* = 14.3, 8.8, 3.5 Hz, 1H, CH_2CHOH), 2.06-2.13 (m, 1H, H4), 2.24-2.38 (m, 1H, H4), 2.58-2.65 (m, 2H, H3), 2.75 (dd, *J* = 9.8, 7.4 Hz, 1H, H5), 3.04 (dd, *J* = 10.1, 5.3 Hz, 1H, H5), 3.13 (dd, *J* = 9.8, 2.3 Hz, 1H, H5), 3.38 (dd, *J* = 10.1, 8.6 Hz, 1H, H5), 3.65 (d, *J* = 6.4 Hz, 2H, CH_2OTMP), 3.78 (d, *J* = 6.2 Hz, 2H, CH_2OTMP), 3.93-4.02 (m, 1H, CHOH), 4.10 (ddq, *J* = 9.9, 6.5, 3.3 Hz, 1H, CHOH), 5.45 (q, *J* = 7.2 Hz, 2H, $PhCHCH_3$), 5.86 (br. s, 2H, OH), 7.23-7.37 (m, 10H, ArH); ^{13}C NMR (101 MHz, $CDCl_3$): δ 16.1 (q, $PhCHCH_3$), 16.4 (q, $PhCHCH_3$), 17.10 (t, piperidine-C4), 17.12 (t, piperidine-C4), 20.26 (q, 2C, $NCCH_3$), 20.31 (q, 2C, $NCCH_3$), 23.1 (q, $CHOHCH_3$), 24.4 (q, $CHOHCH_3$), 33.2 (q, 4C, $NCCH_3$), 37.8 (d, C4), 38.9 (d, C4), 39.4 (t, CH_2CHOH), 39.70 (t, piperidine-C3, C5), 39.8 (t, piperidine-C3, C5), 40.9 (t, CH_2CHOH), 42.7 (d, C3), 44.16 (t, C5), 44.22 (t, C5), 46.3 (d, C3), 49.4 (d, $PhCHCH_3$), 49.5 (d, $PhCHCH_3$), 60.0 (s, 2C, CNO), 60.1 (s, 2C, CNO), 66.1 (d, CHOH), 68.2 (d, CHOH), 76.8 (t, CH_2OTMP), 77.3 (t,

CH$_2$OTMP), 126.9 (d, CH$_{Ar}$), 127.1 (d, CH$_{Ar}$), 127.57 (d, CH$_{Ar}$), 127.62 (d, CH$_{Ar}$), 128.60 (d, CH$_{Ar}$), 128.64 (d, CH$_{Ar}$), 139.6 (s, C$_{Ar}$), 139.7 (s, C$_{Ar}$), 176.6 (s, C2), 177.0 (s, C2).

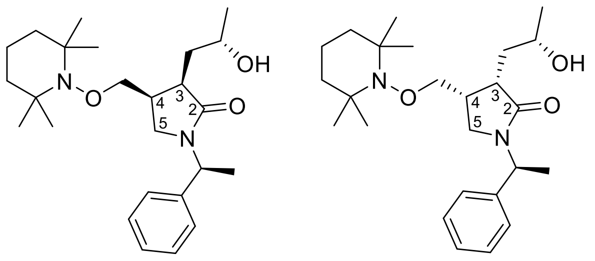

(3*R*,4*S*) (3*S*,4*R*)

Minor diastereomers: ^{1}H NMR (400 MHz, CDCl$_3$, detectable signals): δ 2.80-2.84 (m, 1H, H3), 2.97 (dd, *J* = 10.0, 2.1 Hz, 1H, H5), 2.99 (dd, *J* = 9.4, 3.2 Hz, 1H, H5), 3.27 (dd, *J* = 10.0, 3.5 Hz, 1H, H5), 3.55 (dd, *J* = 9.4, 5.1 Hz, 1H, H5), 3.64 (dd, *J* = 6.2, 3.0 Hz, 1H, CH$_2$OTMP), 3.88-3.95 (m, 1H, CHOH), 5.08 (br. s, 2H, OH); ^{13}C NMR (101 MHz, CDCl$_3$): δ 16.2 (q, PhCHCH$_3$), 16.5 (q, PhCHCH$_3$), 17.2 (t, 2C, piperidine-C4), 20.1 (q, 2C, NCCH$_3$), 20.4 (q, 2C, NCCH$_3$), 23.2 (q, CHOHCH$_3$), 24.3 (q, CHOHCH$_3$), 33.2 (q, 3C, NCCH$_3$), 33.3 (q, NCCH$_3$), 34.2 (t, CH$_2$CHOH), 35.71 (d, C4), 35.74 (d, C4), 35.8 (t, CH$_2$CHOH), 39.71 (t, piperidine-C3, C5), 39.74 (t, piperidine-C3, C5), 41.5 (d, C3), 42.4 (t, C3), 48.3 (t, C5), 48.6 (t, C5), 49.1 (d, PhCHCH$_3$), 49.2 (d, PhCHCH$_3$), 59.8 (s, 2C, CNO), 59.96 (s, 2C, CNO), 66.0 (d, CHOH), 68.3 (d, CHOH), 74.3 (t, CH$_2$OTMP), 75.0 (t, CH$_2$OTMP), 127.2 (d, CH$_{Ar}$), 127.4 (d, CH$_{Ar}$), 127.58 (d, CH$_{Ar}$), 127.63 (d, CH$_{Ar}$), 128.57 (d, CH$_{Ar}$), 128.7 (d, CH$_{Ar}$), 140.1 (s, C$_{Ar}$), 140.4 (s, C$_{Ar}$), 176.0 (s, C2), 176.3 (s, C2).

(3*R*,4*R*)- and (3*S*,4*S*)- and (3*R*,4*S*)- and (3*S*,4*R*)-3-((*S*)-2-Hydroxypropyl)-1-((*S*)-1-(naphthalen-2-yl)ethyl)-4-(((2,2,6,6-tetramethylpiperidin-1-yl)oxy)methyl)pyrrolidin-2-one (12j):

Prepared according to the general procedure, yield 233 mg (77%) as an inseparable 2:2(*trans*):1:1(*cis*) mixture of diastereomers. After equilibration the diastereomeric ratio for *trans*-**12j** increased to 4:4(*trans*):1:1(*cis*).

[R$_f$ (hexanes/EtOAc 1:1) = 0.51]; IR (film); ν [cm^{-1}]: 3374 (br), 2972 (m), 2931 (m), 2873 (w), 1661 (s), 1508 (w), 1489 (w), 1456 (m), 1430 (m), 1374 (w), 1360 (w), 1263 (w), 1132 (w), 1046 (w), 989 (w), 903 (w), 821 (w), 752 (w), 693 (w); MS (+ESI) m/z, (%): 489 (20, [M+Na$^+$]), 467 (100, [M+H$^+$]); HRMS (+ESI) m/z [$C_{29}H_{43}N_2O_3^+$]: calcd. 467.3268; found 467.3260.

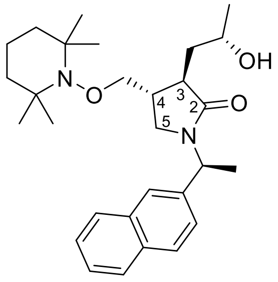

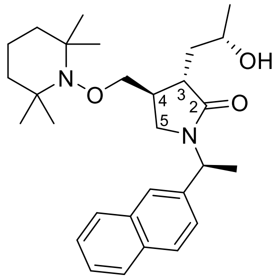

(3*R*,4*R*) (3*S*,4*S*)

Major diastereomers: ^{1}H NMR (400 MHz, CDCl$_3$): δ 1.06 (s, 12H, NCCH$_3$), 1.12 (s, 12H, NCCH$_3$), 1.26 (d, *J* = 6.4 Hz, 3H, CHOHCH$_3$), 1.27 (d, *J* = 6.4 Hz, 3H, CHOHCH$_3$), 1.33-1.42 (m, 2H, piperidine-H4), 1.43-1.49 (m, 8H, piperidine-H3, H5), 1.50-1.59 (m, 2H, piperidine-H4), 1.65 (d, *J* = 6.8 Hz, 3H, ArCHCH$_3$), 1.66 (d, *J* = 7.1 Hz, 3H, ArCHCH$_3$), 1.68-1.88 (m, 4H, CH$_2$CHOH), 2.02-2.13 (m, 1H, H4), 2.29-2.36 (m, 1H, H4), 2.56 (td, *J* = 9.5, 3.5 Hz, 1H, H3), 2.64 (td, *J* = 9.6, 4.3 Hz, 1H, H3), 2.74 (dd, *J* = 9.2, 5.3 Hz, 1H, H5), 2.76 (dd, *J* = 8.9, 3.1 Hz, 1H, H5), 3.06 (dd, *J* = 8.9, 5.3 Hz, 1H, H5), 3.40 (dd, *J* = 9.2, 4.9 Hz, 1H, H5), 3.61 (d, *J* = 5.9 Hz, 2H, CH$_2$OTMP), 3.78 (d, *J* = 6.1 Hz, 2H, CH$_2$OTMP), 3.96-4.04 (m, 1H, CHOH), 4.07-4.18 (m, 1H, CHOH), 5.64 (q, *J* = 7.1 Hz, 2H, ArCHCH$_3$), 5.88 (br. s, 2H, OH), 7.35-7.44 (m, 2H, ArH), 7.45-7.53 (m, 4H, ArH), 7.71-7.78 (m, 2H, ArH), 7.79-7.88 (m, 6H, ArH); ^{13}C NMR (101 MHz, CDCl$_3$): δ 16.2 (q, ArCHCH$_3$), 16.3 (q, ArCHCH$_3$), 17.1 (t, 2C, piperidine-C4), 20.2 (q, 2C, NCCH$_3$), 20.3 (q, 2C, NCCH$_3$), 23.1 (q, CHOHCH$_3$), 24.3 (q, CHOHCH$_3$), 33.04 (q, 2C, NCCH$_3$), 33.3 (q, 2C, NCCH$_3$), 37.7 (d, C4), 38.7 (d, C4), 39.4 (t, CH$_2$CHOH), 39.6 (t, piperidine-C3, C5), 39.7 (t, piperidine-C3, C5), 40.9 (t, CH$_2$CHOH), 42.6 (d, C3), 44.1 (t, C5), 44.3 (t, C5), 46.3 (d, C3), 49.3 (d, ArCHCH$_3$), 49.7 (d, ArCHCH$_3$), 59.9 (s, 2C, CNO), 60.0 (s, 2C, CNO), 66.1 (d, CHOH), 68.2 (d, CHOH), 76.9 (t, CH$_2$OTMP), 77.5 (t, CH$_2$OTMP), 125.32 (d, CH$_{Ar}$), 125.41 (d, CH$_{Ar}$), 125.6 (d, CH$_{Ar}$), 125.8 (d, CH$_{Ar}$), 126.2 (d, CH$_{Ar}$), 126.28 (d, CH$_{Ar}$), 126.33 (d, CH$_{Ar}$), 126.4 (d, CH$_{Ar}$), 127.6 (d, CH$_{Ar}$), 127.68 (d, CH$_{Ar}$), 127.71 (d, CH$_{Ar}$), 128.12 (d, CH$_{Ar}$), 128.62 (d, CH$_{Ar}$), 128.73 (d, CH$_{Ar}$), 132.87 (s, C$_{Ar}$), 132.92 (s, C$_{Ar}$), 133.26 (s, C$_{Ar}$), 133.32 (s, C$_{Ar}$), 137.12 (s, C$_{Ar}$), 137.3 (s, C$_{Ar}$), 176.6 (s, C2), 177.2 (s, C2).

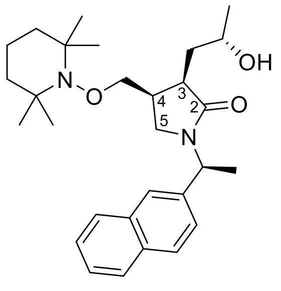

(3*R*,4*S*)

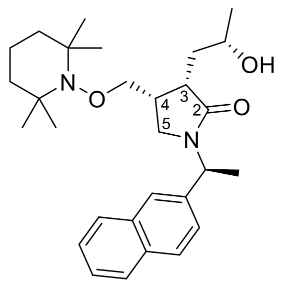

(3*S*,4*R*)

Minor diastereomers: ^{1}H NMR (400 MHz, CDCl$_3$, detectable signals): δ 2.84 (td, *J* = 8.1, 5.3 Hz, 1H, H3), 2.96 (dd, *J* = 10.3, 6.7 Hz, 1H, H5), 2.98 (dd, *J* = 10.1, 6.6 Hz, 1H, H5), 3.23-3.38 (m, 2H, C$\underline{H}_2$OTMP), 4.32-4.43 (m, 2H, C$\underline{H}$OH), 5.05 (br. s, 1H, OH), 5.08 (br. s, 1H, OH); ^{13}C NMR (101 MHz, CDCl$_3$): δ 15.9 (q, ArCH$\underline{C}$H$_3$), 16.4 (q, ArCH$\underline{C}$H$_3$), 17.0 (t, piperidine-C4), 17.12 (t, piperidine-C4), 20.1 (q, 2C, NC$\underline{C}$H$_3$), 20.4 (q, 2C, NC$\underline{C}$H$_3$), 23.2 (q, CHOH$\underline{C}$H$_3$), 24.5 (q, CHOH$\underline{C}$H$_3$), 29.4 (d, C4), 30.4 (d, C4), 33.03 (q, 2C, NC$\underline{C}$H$_3$), 33.4 (q, 2C, NC$\underline{C}$H$_3$), 34.1 (t, $\underline{C}$H$_2$CHOH), 34.2 (t, $\underline{C}$H$_2$CHOH), 39.46 (t, piperidine-C3, C5), 39.48 (t, piperidine-C3, C5), 41.4 (d, C3), 41.5 (d, C3), 44.2 (t, C5), 44.3 (t, C5), 49.3 (d, Ar$\underline{C}$HCH$_3$), 49.6 (d, Ar$\underline{C}$HCH$_3$), 59.6 (s, 2C, CNO), 59.7 (s, 2C, CNO), 66.0 (d, CHOH), 68.3 (d, CHOH), 74.1 (t, $\underline{C}$H$_2$OTMP), 75.0 (t, $\underline{C}$H$_2$OTMP), 125.31 (d, CH$_{Ar}$), 125.43 (d, CH$_{Ar}$), 125.7 (d, CH$_{Ar}$), 126.0 (d, CH$_{Ar}$), 126.1 (d, CH$_{Ar}$), 126.31 (d, CH$_{Ar}$), 126.5 (d, CH$_{Ar}$), 127.2 (d, CH$_{Ar}$), 128.14 (d, CH$_{Ar}$), 128.2 (d, CH$_{Ar}$), 128.56 (d, CH$_{Ar}$), 128.64 (d, CH$_{Ar}$), 128.74 (d, CH$_{Ar}$), 128.8 (d, CH$_{Ar}$), 132.88 (s, C$_{Ar}$), 133.0 (s, C$_{Ar}$), 133.25 (s, C$_{Ar}$), 133.32 (s, C$_{Ar}$), 137.10 (s, C$_{Ar}$), 137.3 (s, C$_{Ar}$), 176.8 (s, C2), 177.1 (s, C2).

(3*R*,4*S*)- and (3*S*,4*R*)- and (3*R*,4*R*)- and (3*S*,4*S*)-3-((*S*)-2-Hydroxypropyl)-1-((*S*)-1-phenylethyl)-4-(prop-1-en-2-yl)pyrrolidin-2-one (12k):

Prepared according to the general procedure, yield 170 mg (92%) **12k** as a partly separable 2:2(*trans*):1:1(*cis*) mixture of diastereomers. After equilibration the diastereomeric ratio for *trans*-**12k** increased to 1:1(*trans*):0:0(*cis*). The minor more polar diastereomer of **12k** crystallized from hexane with a few drops of DCM and its configuration was determined by X-ray crystallography.

[R$_f$ (hexanes/EtOAc 1:1) = 0.36]; IR (film); ν [cm^{-1}]: 3354 (br), 3031 (w), 2969 (w), 2930 (w), 1656 (s), 1494 (w), 1429 (m), 1377 (w), 1342 (w), 1253 (m), 1187 (w), 1136 (w), 1077 (w), 1053 (w), 1028 (w), 939 (w), 897 (w), 786 (w), 753 (w), 700 (m), 637 (w); MS (+ESI) m/z, (%):

597 (10, [2M+Na$^+$]), 310 (100, [M+Na$^+$]), 288 (15, [M+H$^+$]); HRMS (+ESI) m/z [$C_{18}H_{25}NO_2Na^+$]: calcd. 310.1778; found 310.1779.

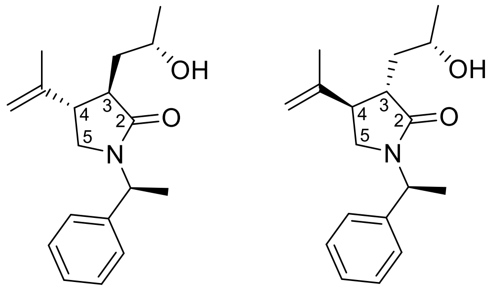

(3*R*,4*S*) (3*S*,4*R*)

Major diastereomers: ^{1}H NMR (400 MHz, $CDCl_3$): δ 1.21 (d, *J* = 6.4 Hz, 3H, $CHOHC\underline{H}_3$), 1.22 (d, *J* = 6.2 Hz, 3H, $CHOHC\underline{H}_3$), 1.51-1.56 (m, 2H, $C\underline{H}_2CHOH$), 1.52 (d, *J* = 7.1 Hz, 3H, $PhCHC\underline{H}_3$), 1.54 (d, *J* = 7.2 Hz, 3H, $PhCHC\underline{H}_3$), 1.60 (s, 3H, $=CCH_3$), 1.61-1.69 (m, 1H, $C\underline{H}_2CHOH$), 1.70 (s, 3H, $=CCH_3$), 1.86 (ddd, *J* = 14.5, 9.0, 3.7 Hz, 1H, $C\underline{H}_2CHOH$), 2.42-2.54 (m, 1H, H4), 2.59-2.70 (m, 1H, H3), 2.71-2.79 (m, 3H, H3, H4, H5), 3.06 (dd, *J* = 9.7, 8.3 Hz, 1H, H5), 3.13 (dd, *J* = 9.7, 4.0 Hz, 1H, H5), 3.37 (dd, *J* = 9.5, 4.0 Hz, 1H, H5), 3.91-3.99 (m, 1H, $C\underline{H}OH$), 4.08-4.17 (m, 1H, $C\underline{H}OH$), 4.79 (d, *J* = 1.7 Hz, 1H, $C=CH_2$), 4.81 (d, *J* = 1.5 Hz, 1H, $C=CH_2$), 4.83 (d, *J* = 1.7 Hz, 1H, $C=CH_2$), 4.88 (d, *J* = 1.5 Hz, 1H, $C=CH_2$), 5.47 (q, *J* = 6.9 Hz, 1H, $PhC\underline{H}CH_3$), 5.49 (q, *J* = 7.0 Hz, 1H, $PhC\underline{H}CH_3$), 5.64 (br. s, 2H, OH), 7.25-7.37 (m, 10H, $Ar\underline{H}$); ^{13}C NMR (101 MHz, $CDCl_3$): δ 16.2 (q, $PhCH\underline{C}H_3$), 16.4 (q, $PhCH\underline{C}H_3$), 19.5 (q, $=C\underline{C}H_3$), 19.6 (q, $=C\underline{C}H_3$), 23.1 (q, $CHOH\underline{C}H_3$), 24.4 (q, $CHOH\underline{C}H_3$), 38.2 (t, $\underline{C}H_2CHOH$), 40.0 (t, $\underline{C}H_2CHOH$), 42.4 (d, C4), 45.7 (t, C5), 45.8 (t, C5), 46.3 (d, C3), 46.9 (d, C3), 47.7 (d, C4), 49.5 (d, $Ph\underline{C}HCH_3$), 49.6 (d, $Ph\underline{C}HCH_3$), 65.3 (d, CHOH), 68.1 (d, CHOH), 113.4 (t, $C=\underline{C}H_2$), 114.0 (t, $C=\underline{C}H_2$), 127.1 (d, CH_{Ar}), 127.3 (d, CH_{Ar}), 127.83 (d, 2C, CH_{Ar}), 128.78 (d, CH_{Ar}), 128.82 (d, CH_{Ar}), 139.72 (s, C_{Ar}), 139.9 (s, C_{Ar}), 142.1 (s, $\underline{C}=CH_2$), 142.7 (s, $\underline{C}=CH_2$), 176.5 (s, C2), 176.82 (s, C2).

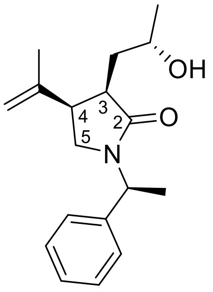

(3*R*,4*R*)

Less polar minor diastereomer: ^{1}H NMR (400 MHz, CDCl$_3$,): δ 1.22 (d, *J* = 6.1 Hz, 3H, CHOHC$\underline{H}_3$), 1.39-1.46 (m, 1H, C$\underline{H}_2$CHOH), 1.55 (d, *J* = 7.1 Hz, 3H, PhCHC$\underline{H}_3$), 1.61-1.69 (m, 1H, C$\underline{H}_2$CHOH), 1.70 (s, 3H, =CCH$_3$), 2.84-2.94 (m, 2H, H3, H4), 3.03 (dd, *J* = 10.0, 6.7 Hz, 1H, H5), 3.27 (dd, *J* = 10.0, 6.2 Hz, 1H, H5), 4.01-4.12 (m, 1H, C$\underline{H}$OH), 4.69 (d, *J* = 1.6 Hz, 1H, C=CH$_2$), 4.87 (d, *J* = 1.6 Hz, 1H, C=CH$_2$), 5.53 (q, *J* = 7.1 Hz, 1H, PhC$\underline{H}$CH$_3$), 5.82 (br. s, 1H, OH), 7.27-7.37 (m, 5H, Ar$\underline{H}$); ^{13}C NMR (101 MHz, CDCl$_3$): δ 16.2 (q, PhCH$\underline{C}$H$_3$), 19.8 (q, =C$\underline{C}$H$_3$), 21.2 (q, =C$\underline{C}$H$_3$), 22.9 (q, CHOH$\underline{C}$H$_3$), 34.3 (t, $\underline{C}$H$_2$CHOH), 41.3 (d, C3), 43.5 (d, C4), 44.6 (t, C5), 49.4 (d, Ph$\underline{C}$HCH$_3$), 65.6 (d, CHOH), 113.3 (t, C=$\underline{C}$H$_2$), 127.2 (d, CH$_{Ar}$), 127.78 (d, CH$_{Ar}$), 128.81 (d, CH$_{Ar}$), 139.66 (s, C$_{Ar}$), 142.8 (s, $\underline{C}$=CH$_2$), 176.84 (s, C2).

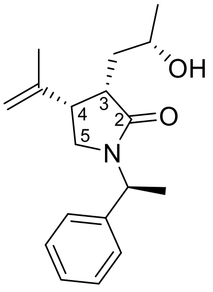

(3*S*,4*S*)

More polar minor diastereomer: ^{1}H NMR (400 MHz, CDCl$_3$): δ 1.20 (d, *J* = 6.1 Hz, 3H, CHOHC$\underline{H}_3$), 1.22 (dd, *J* = 1.5, 0.9 Hz, 3H, =CCH$_3$), 1.48-1.51 (m, 2H, C$\underline{H}_2$CHOH), 1.53 (d, *J* = 7.2 Hz, 3H, PhCHC$\underline{H}_3$), 2.80 (td, *J* = 8.6, 5.2 Hz, 1H, H3), 2.89 (dd, *J* = 10.1, 3.1 Hz, 1H, H5), 2.95-3.00 (m, 1H, H4), 3.48 (dd, *J* = 10.1, 6.9 Hz, 1H, H5), 3.87-3.97 (m, 1H, C$\underline{H}$OH), 4.59 (d, *J* = 1.7 Hz, 1H, C=CH$_2$), 4.68 (d, *J* = 1.7 Hz, 1H, C=CH$_2$), 5.51 (q, *J* = 7.1 Hz, 1H, PhC$\underline{H}$CH$_3$), 5.74 (br. s, 1H, OH), 7.27-7.37 (m, 5H, Ar$\underline{H}$); ^{13}C NMR (101 MHz, CDCl$_3$): δ 15.8 (q, PhCH$\underline{C}$H$_3$), 19.7 (q, =C$\underline{C}$H$_3$), 24.4 (q, CHOH$\underline{C}$H$_3$), 35.8 (t, $\underline{C}$H$_2$CHOH), 43.6 (d, C4), 45.3 (t, C5), 45.5 (d, C3), 49.8 (d, Ph$\underline{C}$HCH$_3$), 68.4 (d, CHOH), 114.5 (t, C=$\underline{C}$H$_2$), 127.84 (d, CH$_{Ar}$), 128.1 (d, CH$_{Ar}$), 128.7 (d, CH$_{Ar}$), 140.0 (s, C$_{Ar}$), 143.4 (s, $\underline{C}$=CH$_2$), 176.9 (s, C2).

(3*R,4*S**)- and (3*S**,4*R**)- and (3*R**,4*R**)-(1-Allyl-3-((1*S**,2*R**)-2-hydroxycyclohexyl)-4-(((2,2,6,6-tetramethylpiperidin-1-yl)oxy)methyl)pyrrolidin-2-one (12l):**

Prepared according to the general procedure, yield 214 mg (84%) as an inseparable 10:8.5(*trans*):1(*cis*) mixture of diastereomers.

[R_f (hexanes/EtOAc 1:1) = 0.52]; IR (film); ν [cm^{-1}]: 3424 (br), 2974 (w), 2928 (s), 2856 (w), 1671 (s), 1489 (w), 1448 (m), 1417 (w), 1374 (w), 1359 (w), 1264 (m), 1245 (w), 1186 (w), 1132 (w), 1049 (m), 993 (w), 956 (w), 924 (w), 791 (w), 732 (w), 712 (w), 656 (w), 635 (w), 610 (w); MS (+ESI) m/z, (%): 807 (60, [$2M+Na^+$]), 415 (90, [$M+Na^+$]), 393 (100, [$M+H^+$]); HRMS (+ESI) m/z [$C_{23}H_{41}N_2O_3^+$]: calcd. 393.3112; found 393.3108.

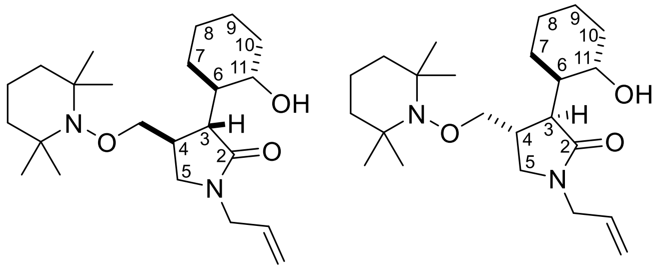

(3*R**,4*S**) (3*S**,4*R**)

Major diastereomers **12lA,B**: ^{1}H NMR (400 MHz, $CDCl_3$): δ 0.92-1.05 (m, 1H, H10), 1.09 (s, 6H, $NCCH_3$), 1.10 (s, 6H, $NCCH_3$), 1.15 (s, 6H, $NCCH_3$), 1.16 (s, 6H, $NCCH_3$), 1.20-1.28 (m, 4H, H7, H9, H10), 1.29-1.37 (m, 3H, H8, piperidine-H4), 1.40-1.50 (m, 8H, piperidine-H3, H5), 1.51-1.63 (m, 3H, H7, piperidine-H4), 1.64-1.71 (m, 5H, H7, H8), 1.72-1.80 (m, 2H, H9), 1.81-1.92 (m, 2H, H6), 1.96-2.09 (m, 2H, H10), 2.45-2.54 (m, 3H, H3, H4), 2.82 (t, *J* = 4.6 Hz, 1H, H3), 2.98 (dd, *J* = 10.2, 3.7 Hz, 1H, H5), 3.14 (dd, *J* = 9.8, 4.6 Hz, 1H, H5), 3.35-3.39 (m, 1H, H11), 3.40 (dd, *J* = 10.2, 8.7 Hz, 1H, H5), 3.42 (dd, *J* = 9.8, 6.3 Hz, 1H, H5), 3.59-3.72 (m, 1H, H11), 3.73-3.81 (m, 4H, C$\underline{H}_2$OTMP), 3.82-3.90 (m, 3H, C$\underline{H}_2$CH=), 3.95 (dd, *J* = 15.1, 6.1 Hz, 1H, C$\underline{H}_2$CH=), 5.14-5.26 (m, 6H, OH, CH=C$\underline{H}_2$), 5.64-5.78 (m, 2H, C$\underline{H}$=CH_2); ^{13}C NMR (101 MHz, $CDCl_3$): δ 17.19 (q, piperidine-C4), 17.20 (q, piperidine-C4), 20.2 (q, NC$\underline{C}H_3$), 20.3 (q, NC$\underline{C}H_3$), 20.5 (q, NC$\underline{C}H_3$), 20.7 (q, NC$\underline{C}H_3$), 25.3 (t, C9), 25.7 (t, C9), 25.9 (t, C8), 26.0 (t, C8), 26.8 (t, C7), 29.2 (t, C7), 31.6 (d, C4), 33.2 (q, 2C, NC$\underline{C}H_3$), 33.3 (q, 2C, NC$\underline{C}H_3$), 33.4 (d, C4), 35.2 (t, C10), 36.5 (t, C10), 39.8 (t, piperidine-C3, C5), 39.9 (t, piperidine-C3), 40.0 (t, piperidine-C5), 45.5 (t, 2C, $\underline{C}H_2$CH=), 46.7 (d, 2C, C3, C6), 47.0 (d, C6), 47.1 (d, C3), 49.0 (t, C5), 49.2 (t, C5), 60.1 (s, 2C, CNO), 60.20 (s, 2C, CNO), 72.16 (d, C11), 72.18 (d, C11), 79.0 (t, $\underline{C}H_2$OTMP), 79.8 (t, $\underline{C}H_2$OTMP), 117.9 (t, CH=$\underline{C}H_2$), 118.29 (t, CH=$\underline{C}H_2$), 132.5 (d, $\underline{C}$H=CH_2), 132.6 (d, $\underline{C}$H=CH_2), 176.3 (s, C2), 176.9 (s, C2).

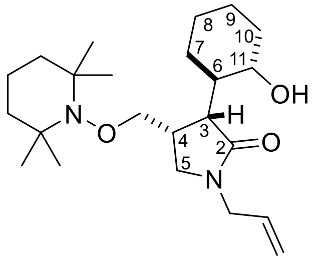

(3*R**,4*R**)

Minor *cis*-diastereomer: ^{1}H NMR (400 MHz, $CDCl_3$, detectable signals): δ 3.15-3.24 (m, 1H, H5), 3.60-3.70 (m, 1H, C<u>H</u>OH); ^{13}C NMR (101 MHz, $CDCl_3$, detectable signals): δ 25.0 (t, C9), 27.9 (t, C8), 28.0 (t, C7), 35.1 (d, C4), 36.2 (t, C10), 45.3 (t, <u>C</u>H_2CH=), 50.8 (t, C5), 60.0 (s, CNO), 60.16 (s, CNO), 71.1 (d, C11), 75.4 (t, <u>C</u>H_2OTMP), 118.26 (t, CH=<u>C</u>H_2), 132.7 (d, <u>C</u>H=CH_2), 176.1 (s, C2).

(4*S,5*S**,6*R**)- and (4*S**,5*S**,6*S**)- and (4*R**,5*S**,6*R**)- and (4*R**,5*S**,6*S**)-2-Benzyl-4-(2-hydroxypropyl)-6-((2,2,6,6-tetramethylpiperidin-1-yl)oxy)-2-azaspiro[4.4]nonan-3-one (12m):**

Prepared according to the general procedure, yield 198 mg (69%) as an inseparable 9:9:5:5(*trans*):2:2:1:1(*cis*) mixture of diastereomers. After equilibration the diastereomeric ratio for *equil*-**12m** increased to 11:11:6:6(*trans*):3:3:1:1(*cis*).

[R_f (hexanes/EtOAc 2:1) = 0.24]; IR (film); ν [cm^{-1}]: 3336 (br), 2966 (m), 2930 (m), 2874 (w), 1668 (s), 1493 (w), 1453 (m), 1375 (w), 1360 (w), 1319 (w), 1259 (w), 1208 (w), 1180 (w), 1133 (w), 1081 (w), 1030 (w), 975 (w), 955 (w), 736 (w), 701 (m), 606 (w); MS (+ESI) m/z, (%): 907 (35, [$2M+Na^+$]), 465 (100, [$M+Na^+$]), 443 (15, [$M+H^+$]); HRMS (+ESI) m/z [$C_{26}H_{41}N_2O_3Na^+$]: calcd. 465.3088; found 465.3088.

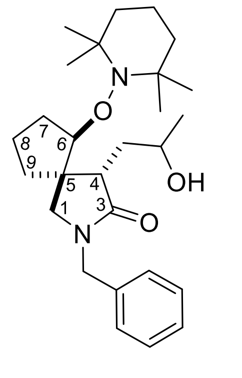

(4*S**,5*S**,6*R**)

12mA

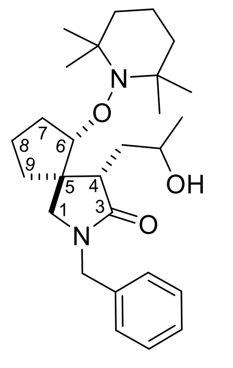

(4*S**,5*S**,6*S**)

12mB

Major diastereomers **12mA**,**B**: ^{1}H NMR (400 MHz, $CDCl_3$): δ 1.00 (s, 6H, $NCCH_3$), 1.05 (s, 6H, $NCCH_3$), 1.07 (s, 9H, $NCCH_3$), 1.11 (s, 9H, $NCCH_3$), 1.12 (s, 9H, $NCCH_3$), 1.15 (s, 9H, $NCCH_3$), 1.22-1.34 (m, 4H, piperidine-H4), 1.24 (d, *J* = 6.2 Hz, 6H, CH_3), 1.25 (d, *J* = 6.2 Hz, 3H, CH_3), 1.26 (d, *J* = 6.4 Hz, 3H, CH_3), 1.36-1.48 (m, 16H, piperidine-H3, H5), 1.49-1.65 (m, 20H, H7, H8, H9, CH_2CHOH, piperidine-H4), 1.66-1.75 (m, 8H, H7, H8, H9, CH_2CHOH), 1.76-1.87 (m, 2H, CH_2CHOH), 1.89-1.97 (m, 1H, H7), 1.98-2.06 (m, 1H, H7), 2.75 (d, *J* = 9.4 Hz, 1H, H1), 2.82 (d, *J* = 9.4 Hz, 1H, H1), 2.92-3.05 (m, 4H, H4), 2.96 (d, *J* = 10.0 Hz, 1H, H1), 2.99 (d, *J* = 10.1 Hz, 1H, H1), 3.60 (d, *J* = 9.4 Hz, 1H, H1), 3.67 (d, *J* = 9.4 Hz, 1H, H1), 3.68 (d, *J* = 10.0 Hz, 1H, H1), 3.72 (d, *J* = 10.1 Hz, 1H, H1), 3.90-4.08 (m, 4H, H6, CHOH), 4.14-4.24 (m, 4H, H6, CHOH), 4.17 (d, *J* = 14.3 Hz, 1H, CH_2Ph), 4.18 (d, *J* = 14.5 Hz, 1H, CH_2Ph), 4.20 (d, *J* = 14.4 Hz, 1H, CH_2Ph), 4.21 (d, *J* = 13.0 Hz, 1H, CH_2Ph), 4.72 (d, *J* = 13.0 Hz, 1H, CH_2Ph), 4.73 (d, *J* = 14.4 Hz, 1H, CH_2Ph), 4.76 (d, *J* = 14.3 Hz, 1H, CH_2Ph), 4.97 (d, *J* = 14.5 Hz, 1H, CH_2Ph), 5.72 (br. s, 1H, OH), 6.31 (br. s, 3H, OH), 7.19-7.26 (m, 8H, ArH), 7.27-7.37 (m, 12H, ArH); ^{13}C NMR (101 MHz, $CDCl_3$): δ 17.2 (t, 3C, piperidine-C4), 17.3 (t, piperidine-C4), 18.1 (t, C8), 18.8 (t, C8), 19.4 (t, C8), 19.97 (t, C8), 20.57 (q, $NCCH_3$), 20.59 (q, $NCCH_3$), 20.61 (q, $NCCH_3$), 20.63 (q, $NCCH_3$), 20.64 (q, $NCCH_3$), 20.68 (q, $NCCH_3$), 20.71 (q, $NCCH_3$), 20.8 (q, $NCCH_3$), 22.8 (q, 2C, CH_3), 24.6 (q, CH_3), 24.7 (q, CH_3), 28.1 (t, C7), 28.7 (t, C7), 28.9 (t, C7), 29.5 (t, C7), 34.0 (t, 2C, CH_2CHOH), 34.17 (q, $NCCH_3$), 34.24 (q, $NCCH_3$), 34.5 (q, $NCCH_3$), 34.6 (q, $NCCH_3$), 34.7 (q, $NCCH_3$), 34.8 (q, $NCCH_3$), 34.87 (q, $NCCH_3$), 34.94 (q, $NCCH_3$), 35.1 (t, CH_2CHOH), 35.3 (t, CH_2CHOH), 35.7 (t, C9), 36.2 (t, C9), 37.0 (t, 2C, C9), 40.49 (t, piperidine-C3, C5), 40.53 (t, piperidine-C3, C5), 40.6 (t, piperidine-C3, C5), 40.7 (t, piperidine-C3, C5), 42.8 (d, C4), 46.8 (d, C4), 46.88 (d, C4), 47.0 (t, 2C, CH_2Ph), 47.1 (t, 2C, CH_2Ph), 50.7 (d, C4), 51.20 (t, C1), 51.5 (t, C1), 53.3 (t, C1), 53.7 (t, C1), 56.5 (s, 2C, C5), 57.1

(s, 2C, C5), 59.2 (s, CNO), 59.28 (s, CNO), 59.31 (s, CNO), 59.4 (s, CNO), 61.10 (s, CNO), 61.12 (s, CNO), 61.16 (s, CNO), 61.19 (s, CNO), 64.9 (d, CHOH), 65.2 (d, CHOH), 68.6 (d, CHOH), 68.7 (d, CHOH), 82.7 (d, C6), 83.8 (d, C6), 84.1 (d, C6), 84.6 (d, C6), 127.74 (d, CH_{Ar}), 127.81 (d, CH_{Ar}), 128.11 (d, 2C, CH_{Ar}), 128.14 (d, CH_{Ar}), 128.17 (d, 2C, CH_{Ar}), 128.3 (d, CH_{Ar}), 128.86 (d, 4C, CH_{Ar}), 136.1 (s, C_{Ar}), 136.3 (s, C_{Ar}), 136.4 (s, C_{Ar}), 136.5 (s, C_{Ar}), 176.8 (s, C3), 177.2 (s, C3), 177.7 (s, C3), 177.8 (s, C3).

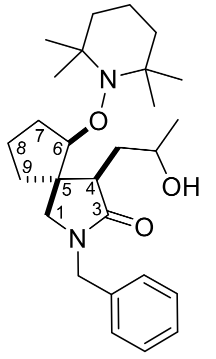

(4*R**,5*S**,6*R**)

12mC

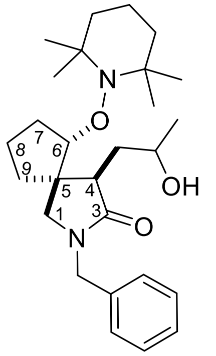

(4*R**,5*S**,6*S**)

12mD

Minor diastereomers **12mC,D**: ^{1}H NMR (400 MHz, $CDCl_3$, detectable signals): δ 2.43 (d, *J* = 11.3 Hz, 1H, H1), 2.44 (d, *J* = 11.3 Hz, 1H, H1), 2.50 (d, *J* = 11.9 Hz, 1H, H1), 2.51 (d, *J* = 11.9 Hz, 1H, H1), 2.66 (d, *J* = 11.9 Hz, 1H, H1), 2.68 (d, *J* = 11.9 Hz, 1H, H1), 2.81 (d, *J* = 13.7 Hz, 1H, H1), 2.84 (d, *J* = 13.7 Hz, 1H, H1), 3.10-3.18 (m, 3H, H4), 3.32 (dd, *J* = 9.4, 6.2 Hz, 1H, H4), 4.06-4.16 (m, 1H, $C\underline{H}OH$), 4.29 (d, *J* = 14.4 Hz, 1H, $C\underline{H}_2Ph$), 4.33 (d, *J* = 14.4 Hz, 1H, $C\underline{H}_2Ph$), 4.35 (d, *J* = 14.7 Hz, 1H, $C\underline{H}_2Ph$), 4.36 (d, *J* = 14.4 Hz, 1H, $C\underline{H}_2Ph$), 4.55 (d, *J* = 14.4 Hz, 1H, $C\underline{H}_2Ph$), 4.57 (d, *J* = 14.4 Hz, 1H, $C\underline{H}_2Ph$), 4.62 (d, *J* = 14.4 Hz, 1H, $C\underline{H}_2Ph$), 4.97 (d, *J* = 14.7 Hz, 1H, $C\underline{H}_2Ph$), 5.16 (br. s, 1H, OH), 6.27 (br. s, 3H, OH); ^{13}C NMR (101 MHz, $CDCl_3$): δ 17.2 (t, 3C, piperidine-C4), 17.3 (t, piperidine-C4), 18.8 (t, C8), 20.00 (t, C8), 20.2 (t, C8), 20.5 (t, C8), 20.57 (q, $NC\underline{C}H_3$), 20.59 (q, $NC\underline{C}H_3$), 20.61 (q, $NC\underline{C}H_3$), 20.63 (q, $NC\underline{C}H_3$), 20.64 (q, $NC\underline{C}H_3$), 20.68 (q, $NC\underline{C}H_3$), 20.71 (q, $NC\underline{C}H_3$), 20.8 (q, $NC\underline{C}H_3$), 22.7 (q, 2C, CH_3), 22.9 (q, CH_3), 24.3 (q, CH_3), 28.3 (t, C7), 28.5 (t, C7), 29.4 (t, C7), 30.4 (t, C7), 34.0 (t, $\underline{C}H_2CHOH$), 34.17 (q, $NC\underline{C}H_3$), 34.24 (q, $NC\underline{C}H_3$), 34.5 (q, $NC\underline{C}H_3$), 34.6 (q, $NC\underline{C}H_3$), 34.7 (q, $NC\underline{C}H_3$), 34.8 (q, $NC\underline{C}H_3$), 34.87 (q, $NC\underline{C}H_3$), 34.94 (q, $NC\underline{C}H_3$), 35.1 (t, $\underline{C}H_2CHOH$), 35.2 (t, $\underline{C}H_2CHOH$), 35.9 (t, $\underline{C}H_2CHOH$), 36.5 (t, C9), 37.0 (t, 2C, C9), 37.9 (t, C9), 40.77 (t, piperidine-C3, C5), 40.83 (t, piperidine-C3, C5), 41.0 (t, piperidine-C3, C5), 41.1 (t, piperidine-C3, C5),

41.6 (d, C4), 42.8 (d, C4), 46.7 (d, C4), 46.93 (t, 2C, C̲H_2Ph), 47.3 (t, 2C, C̲H_2Ph), 51.0 (t, C1), 51.1 (t, C1), 51.17 (t, C1), 51.38 (t, C1), 51.43 (d, C4), 54.7 (s, 2C, C5), 55.7 (s, 2C, C5), 59.2 (s, CNO), 59.28 (s, CNO), 59.31 (s, CNO), 59.4 (s, CNO), 61.10 (s, CNO), 61.12 (s, CNO), 61.16 (s, CNO), 61.19 (s, CNO), 65.4 (d, CHOH), 65.5 (d, CHOH), 68.8 (d, CHOH), 69.0 (d, CHOH), 87.2 (d, C6), 88.0 (d, C6), 88.7 (d, C6), 89.5 (d, C6), 127.73 (d, CH_{Ar}), 127.78 (d, CH_{Ar}), 127.9 (d, CH_{Ar}), 128.14 (d, CH_{Ar}), 128.24 (d, CH_{Ar}), 128.3 (d, CH_{Ar}), 128.36 (d, CH_{Ar}), 128.43 (d, CH_{Ar}), 128.86 (d, 3C, CH_{Ar}), 128.92 (d, CH_{Ar}), 136.1 (s, C_{Ar}), 136.3 (s, C_{Ar}), 136.4 (s, C_{Ar}), 136.5 (s, C_{Ar}), 177.2 (s, C3), 177.58 (s, C3), 177.63 (s, C3), 177.8 (s, C3).

(4*S,5*S**,6*R**)- and (4*S**,5*S**,6*S**)- and (4*R**,5*S**,6*R**)-2-Benzyl-4-(2-hydroxypropyl)-6-((2,2,6,6-tetramethylpiperidin-1-yl)oxy)-2-azaspiro[4.5]decan-3-one (12n):**

Prepared according to the general procedure, yield 196 mg (66%) as an inseparable 4:4:1:1(*trans*):2:2(*cis*) mixture of diastereomers. After equilibration the diastereomeric ratio for *equil*-**12n** increased to 5:5:1:1(*trans*):1:1(*cis*).

[R_f (hexanes/EtOAc 2:1) = 0.35]; IR (film); ν [cm^{-1}]: 3320 (br), 2928 (m), 2857 (w), 1664 (s), 1493 (w), 1450 (m), 1375 (w), 1360 (w), 1256 (w), 1133 (w), 1081 (w), 1039 (w), 991 (w), 913 (w), 878 (w), 842 (w), 790 (w), 730 (m), 700 (m), 645 (w); MS (+ESI) m/z, (%): 457 (100, $[M+H^+]$); HRMS (+ESI) m/z [$C_{28}H_{45}N_2O_3^+$]: calcd. 457.3425; found 457.3420.

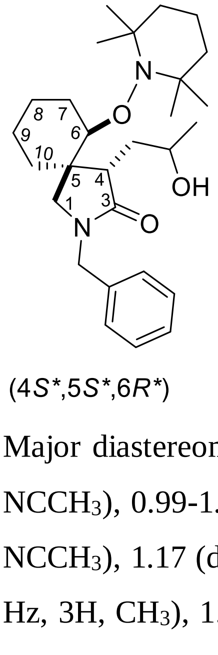

(4*S**,5*S**,6*R**)

Major diastereomers **12nA**: ^{1}H NMR (400 MHz, $CDCl_3$): δ 0.96 (s, 6H, $NCCH_3$), 0.98 (s, 6H, $NCCH_3$), 0.99-1.05 (m, 4H, H7, H8), 1.08 (s, 6H, $NCCH_3$), 1.12-1.22 (m, 2H, H9), 1.15 (s, 6H, $NCCH_3$), 1.17 (d, *J* = 6.4 Hz, 3H, CH_3), 1.23-1.33 (m, 4H, H10, piperidine-H4), 1.25 (d, *J* = 6.2 Hz, 3H, CH_3), 1.34-1.49 (m, 12H, H9, H10, piperidine-H3, H5), 1.50-1.61 (m, 6H, CH̲$_2$CHOH, piperidine-H4), 1.62-1.75 (m, 2H, H8), 2.29-2.40 (m, 3H, H4, H7), 2.44-2.48 (m, 1H, H4), 2.98 (d, *J* = 9.9 Hz, 1H, H1), 3.02 (d, *J* = 10.2 Hz, 1H, H1), 3.48 (d, *J* = 9.9 Hz, 1H, H1), 3.51 (d, *J* =

10.2 Hz, 1H, H1), 3.65 (dd, J = 11.6, 3.7 Hz, 1H, H6), 3.67-3.71 (m, 1H, H6), 3.72 (d, J = 14.9 Hz, 1H, $C\underline{H}_2Ph$), 3.96 (tq, J = 6.2, 3.1 Hz, 1H, $C\underline{H}OH$), 4.23 (tq, J = 6.2, 2.9 Hz, 1H, $C\underline{H}OH$), 4.36 (d, J = 14.8 Hz, 1H, $C\underline{H}_2Ph$), 4.59 (d, J = 14.8 Hz, 1H, $C\underline{H}_2Ph$), 5.24 (d, J = 14.9 Hz, 1H, $C\underline{H}_2Ph$), 6.44 (br. s, 1H, OH), 6.50 (br. s, 1H, OH), 7.20-7.25 (m, 4H, $Ar\underline{H}$), 7.27-7.36 (m, 6H, $Ar\underline{H}$); ^{13}C NMR (101 MHz, $CDCl_3$): δ 17.3 (t, piperidine-C4), 17.37 (t, piperidine-C4), 20.7 (q, 2C, $NC\underline{C}H_3$), 21.3 (q, 2C, $NC\underline{C}H_3$), 21.7 (t, C9), 22.0 (t, C9), 22.98 (q, CH_3), 24.6 (t, 2C, C8), 24.8 (q, CH_3), 28.0 (t, C7), 28.2 (t, C7), 29.3 (t, C10), 30.2 (t, C10), 33.0 (t, $\underline{C}H_2CHOH$), 33.9 (q, 2C, $NC\underline{C}H_3$), 34.1 (q, 2C, $NC\underline{C}H_3$), 34.5 (t, $\underline{C}H_2CHOH$), 40.56 (t, piperidine-C3, C5), 40.60 (t, piperidine-C3, C5), 42.2 (d, 2C, C4), 45.8 (s, C5), 46.3 (s, C5), 46.98 (t, $\underline{C}H_2Ph$), 47.01 (t, $\underline{C}H_2Ph$), 48.2 (t, C1), 48.6 (t, C1), 59.19 (s, CNO), 59.23 (s, CNO), 60.9 (s, CNO), 61.2 (s, CNO), 65.3 (d, CHOH), 68.8 (d, CHOH), 79.0 (d, C6), 80.0 (d, C6), 127.7 (d, CH_{Ar}), 128.11 (d, 2C, CH_{Ar}), 128.86 (d, 2C, CH_{Ar}), 128.87 (d, CH_{Ar}), 136.2 (s, C_{Ar}), 136.36 (s, C_{Ar}), 177.21 (s, C3), 177.7 (s, C3).

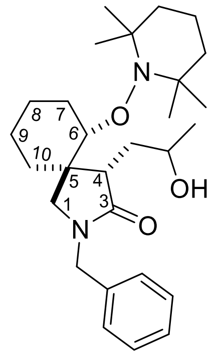

(4*S**,5*S**,6*S**)

Diastereomers **12nB**: ^1H NMR (400 MHz, $CDCl_3$, detectable signals): δ 2.18 (dd, J = 14.0, 9.7 Hz, 1H, H4), 2.49-2.57 (m, 2H, H7), 2.80 (d, J = 12.6 Hz, 1H, H1), 2.93 (d, J = 9.8 Hz, 1H, H1), 3.25 (d, J = 9.8 Hz, 1H, H1), 3.29 (d, J = 9.8 Hz, 1H, H1), 4.08-4.15 (m, 1H, $C\underline{H}OH$), 5.10 (d, J = 14.7 Hz, 1H, $C\underline{H}_2Ph$), 5.20 (d, J = 14.3 Hz, 1H, $C\underline{H}_2Ph$), 5.63 (br. s, 1H, OH), 6.44 (br. s, 1H, OH); ^{13}C NMR (101 MHz, $CDCl_3$): δ 17.37 (t, piperidine-C4), 17.43 (t, piperidine-C4), 20.7 (q, $NC\underline{C}H_3$), 20.9 (q, $NC\underline{C}H_3$), 21.1 (q, $NC\underline{C}H_3$), 21.3 (q, $NC\underline{C}H_3$), 22.4 (q, CH_3), 22.8 (t, C9), 23.6 (t, C9), 23.9 (t, C8), 24.3 (t, C8), 24.7 (q, CH_3), 26.79 (t, C7), 26.83 (t, C7), 32.5 (t, C10), 32.7 (t, C10), 33.9 (q, $NC\underline{C}H_3$), 34.1 (q, $NC\underline{C}H_3$), 34.5 (q, $NC\underline{C}H_3$), 34.8 (q, $NC\underline{C}H_3$), 35.6 (t, $\underline{C}H_2CHOH$), 37.0 (t, $\underline{C}H_2CHOH$), 41.0 (d, C4), 41.36 (t, piperidine-C3, C5), 41.43 (t, piperidine-C3, C5), 45.8 (s, C5), 46.0 (s, C5), 46.8 (t, $\underline{C}H_2Ph$), 46.9 (t, $\underline{C}H_2Ph$), 46.99 (d, C4), 58.3 (t, C1),

58.5 (t, C1), 59.19 (s, CNO), 59.23 (s, CNO), 60.9 (s, CNO), 61.1 (s, CNO), 65.0 (d, CHOH), 68.7 (d, CHOH), 87.6 (d, C6), 88.7 (d, C6), 127.6 (d, CH_{Ar}), 127.8 (d, CH_{Ar}), 128.2 (d, CH_{Ar}), 128.3 (d, CH_{Ar}), 128.4 (d, CH_{Ar}), 128.90 (d, CH_{Ar}), 136.3 (s, C_{Ar}), 136.5 (s, C_{Ar}), 177.23 (s, C3), 177.33 (s, C3).

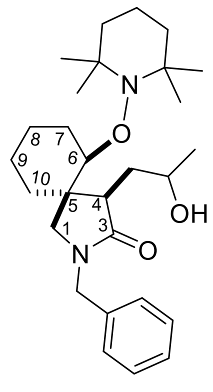

(4*R**,5*S**,6*R**)

Diastereomers **12nC**: ^{1}H NMR (400 MHz, $CDCl_3$): δ 0.96 (s, 6H, $NCCH_3$), 0.98 (s, 6H, $NCCH_3$), 0.99-1.05 (m, 2H, H8), 1.08 (s, 6H, $NCCH_3$), 1.12-1.22 (m, 2H, H7), 1.15 (s, 6H, $NCCH_3$), 1.23-1.33 (m, 4H, H10, piperidine-H4), 1.29 (d, *J* = 6.5 Hz, 6H, CH_3), 1.34-1.49 (m, 12H, H7, H10, piperidine-H3, H5), 1.50-1.61 (m, 5H, $C\underline{H}_2CHOH$, piperidine-H4), 1.62-1.75 (m, 4H, H8, H9), 1.81 (ddd, *J* = 15.0, 11.9, 3.2 Hz, 1H, $C\underline{H}_2CHOH$), 2.29-2.40 (m, 2H, H9), 2.44-2.48 (m, 2H, H4), 2.96 (d, *J* = 9.7 Hz, 1H, H1), 3.01 (d, *J* = 10.3 Hz, 1H, H1), 3.54 (d, *J* = 10.3 Hz, 1H, H1), 3.55 (d, *J* = 14.5 Hz, 1H, $C\underline{H}_2Ph$), 3.61 (d, *J* = 9.7 Hz, 1H, H1), 3.62-3.80 (m, 2H, H6), 3.85-3.94 (m, 2H, $C\underline{H}OH$), 4.30 (d, *J* = 14.8 Hz, 1H, $C\underline{H}_2Ph$), 4.64 (d, *J* = 14.8 Hz, 1H, $C\underline{H}_2Ph$), 5.25 (d, *J* = 14.5 Hz, 1H, $C\underline{H}_2Ph$), 6.50 (br. s, 2H, OH), 7.20-7.25 (m, 4H, $Ar\underline{H}$), 7.27-7.36 (m, 6H, $Ar\underline{H}$); ^{13}C NMR (101 MHz, $CDCl_3$): δ 17.3 (t, piperidine-C4), 17.37 (t, piperidine-C4), 20.7 (q, 2C, $NC\underline{C}H_3$), 20.9 (q, $NC\underline{C}H_3$), 21.1 (q, $NC\underline{C}H_3$), 23.2 (t, 2C, C9), 24.7 (q, CH_3), 24.8 (t, 2C, C8), 24.9 (q, CH_3), 26.3 (t, C7), 26.4 (t, C7), 32.4 (t, C10), 33.3 (t, C10), 34.5 (q, 2C, $NC\underline{C}H_3$), 34.8 (q, 2C, $NC\underline{C}H_3$), 37.37 (t, $\underline{C}H_2CHOH$), 37.40 (t, $\underline{C}H_2CHOH$), 40.5 (t, piperidine-C3), 40.7 (t, piperidine-C5), 40.8 (t, piperidine-C3, C5), 46.2 (s, C5), 46.3 (s, C5), 47.5 (t, $\underline{C}H_2Ph$), 47.6 (t, $\underline{C}H_2Ph$), 47.8 (d, C4), 52.98 (t, C1), 52.99 (t, C1), 53.6 (d, C4), 59.3 (s, 2C, CNO), 61.1 (s, 2C, CNO), 65.2 (d, CHOH), 69.1 (d, CHOH), 82.8 (d, C6), 83.0 (d, C6), 127.8 (d, CH_{Ar}), 128.07 (d, CH_{Ar}), 128.5 (d, CH_{Ar}), 128.8 (d, CH_{Ar}), 128.86 (d, 2C, CH_{Ar}), 136.40 (s, C_{Ar}), 136.6 (s, C_{Ar}), 177.5 (s, C3), 177.8 (s, C3).

(3*R,3a*R**,4*R**,6a*R**)- and (3*S**,3a*R**,4*R**,6a*R**)-1-Benzyl-3-(2-hydroxypropyl)-4-((2,2,6,6-tetramethylpiperidin-1-yl)oxy)hexahydrocyclopenta[*b*]pyrrol-2(1*H*)-one (12o):**

Prepared according to the general procedure, yield 228 mg (82%) as an inseparable 4:4(*trans*):1:1(*cis*) mixture of diastereomers.

[R_f (hexanes/EtOAc 1:1) = 0.31]; IR (film); ν [cm^{-1}]: 3386 (br), 2966 (w), 2930 (m), 2871 (w), 1659 (s), 1449 (m), 1434 (m), 1374 (w), 1359 (m), 1299 (w), 1254 (m), 1182 (w), 1133 (m), 1080 (w), 1030 (w), 961 (m), 732 (m), 701 (m), 645 (w), 618 (w); MS (+ESI) m/z, (%): 451 (25, [$M+Na^+$]), 429 (100, [$M+H^+$]); HRMS (+ESI) m/z [$C_{26}H_{41}N_2O_3^+$]: calcd. 429.3112; found 429.3106.

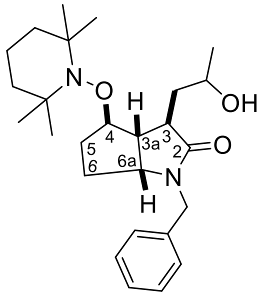

(3*R**,3a*R**,4*R**,6a*R**)

Major diastereomers **12oA**: ^1H NMR (400 MHz, $CDCl_3$): δ 1.04 (s, 6H, $NCCH_3$), 1.08 (s, 12H, $NCCH_3$), 1.14 (s, 6H, $NCCH_3$), 1.24 (d, *J* = 6.2 Hz, 3H, CH_3), 1.26 (d, *J* = 6.1 Hz, 3H, CH_3), 1.29-1.36 (m, 2H, piperidine-H4), 1.41-1.48 (m, 8H, piperidine-H3, H5), 1.49-1.56 (m, 2H, piperidine-H4), 1.59-1.74 (m, 5H, H5, H6, $C\underline{H}_2CHOH$), 1.75-1.81 (m, 4H, H6, $C\underline{H}_2CHOH$), 1.82-2.09 (m, 3H, H5, $C\underline{H}_2CHOH$), 2.29-2.42 (m, 2H, H3), 2.56 (ddd, *J* = 8.7, 4.6, 1.7 Hz, 1H, H3a), 2.80-2.91 (m, 1H, H3a), 3.84-3.90 (m, 2H, H6a), 3.95 (d, *J* = 14.8 Hz, 2H, $C\underline{H}_2Ph$), 3.96-4.05 (m, 1H, $C\underline{H}OH$), 4.06-4.12 (m, 1H, $C\underline{H}OH$), 4.15 (q, *J* = 2.3 Hz, 1H, H4), 4.19 (dt, *J* = 4.6, 2.4 Hz, 1H, H4), 4.92 (d, *J* = 14.9 Hz, 2H, $C\underline{H}_2Ph$), 5.31 (br. s, 2H, OH), 7.19-7.25 (m, 4H, $Ar\underline{H}$), 7.27-7.37 (m, 6H, $Ar\underline{H}$); ^{13}C NMR (101 MHz, $CDCl_3$): δ 17.2 (t, piperidine-C4), 17.27 (t, piperidine-C4), 20.5 (q, 2C, $NC\underline{C}H_3$), 20.7 (q, 2C, $NC\underline{C}H_3$), 23.51 (q, CH_3), 24.3 (q, CH_3), 28.20 (t, C6), 28.24 (t, C6), 29.68 (t, C5), 29.73 (t, C5), 34.6 (q, 2C, $NC\underline{C}H_3$), 35.0 (q, 2C, $NC\underline{C}H_3$), 40.2 (t, piperidine-C3, C5), 40.3 (t, piperidine-C3, C5), 41.5 (t, $\underline{C}H_2CHOH$), 43.0 (t, $\underline{C}H_2CHOH$), 45.2 (d, C3), 45.3 (t, $\underline{C}H_2Ph$), 45.6 (t, $\underline{C}H_2Ph$), 47.1 (d, C3a), 47.3 (d, C3), 49.0 (d, C3a), 60.3 (s, 4C, CNO), 60.7 (d, C6a), 60.79 (d, C6a), 66.7 (d, CHOH), 67.9 (d, CHOH), 90.78 (d, C4), 90.82 (d, C4), 127.72 (d, CH_{Ar}), 127.8 (d, CH_{Ar}), 128.2 (d, CH_{Ar}), 128.3 (d, CH_{Ar}), 128.86 (d, CH_{Ar}), 128.91 (d, CH_{Ar}), 136.2 (s, C_{Ar}), 136.5 (s, C_{Ar}), 176.6 (s, C2), 177.25 (s, C2).

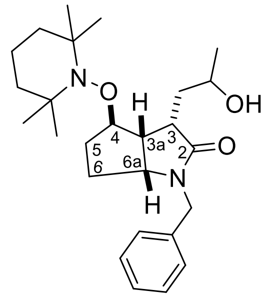

(3S*,3aR*,4R*,6aR*)

Minor diastereomers **12oB**: ^{1}H NMR (400 MHz, $CDCl_3$, detectable signals): δ 2.80 (td, J = 7.9, 4.5 Hz, 1H, H3a), 2.96 (ddd, J = 10.1, 7.9, 5.7 Hz, 1H, H3a), 3.93 (d, J = 14.8 Hz, 1H, C$\underline{H}_2$Ph), 3.94 (d, J = 14.6 Hz, 1H, C$\underline{H}_2$Ph), 4.30 (dt, J = 9.0, 5.7 Hz, 1H, H4), 4.31 (dt, J = 9.6, 4.5 Hz, 1H, H4), 4.91 (d, J = 14.8 Hz, 1H, C$\underline{H}_2$Ph), 4.95 (d, J = 14.6 Hz, 1H, C$\underline{H}_2$Ph), 5.81 (br. s, 1H, OH); ^{13}C NMR (101 MHz, $CDCl_3$): δ 17.31 (t, 2C, piperidine-C4), 20.5 (q, 2C, NC$\underline{C}H_3$), 20.7 (q, 2C, NC$\underline{C}H_3$), 23.49 (q, CH_3), 24.4 (q, CH_3), 27.7 (t, C6), 27.8 (t, C6), 29.8 (t, C5), 30.0 (t, C5), 34.6 (q, 2C, NC$\underline{C}H_3$), 35.0 (q, 2C, NC$\underline{C}H_3$), 36.1 (t, $\underline{C}H_2$CHOH), 37.8 (t, $\underline{C}H_2$CHOH), 40.2 (t, piperidine-C3, C5), 40.3 (t, piperidine-C3, C5), 40.6 (d, C3), 43.8 (d, C3), 44.8 (t, $\underline{C}H_2$Ph), 45.0 (t, $\underline{C}H_2$Ph), 46.5 (d, C3a), 46.6 (d, C3a), 60.3 (s, 4C, CNO), 60.81 (d, C6a), 61.0 (d, C6a), 66.8 (d, CHOH), 68.3 (d, CHOH), 85.0 (d, C4), 85.1 (d, C4), 127.74 (d, CH_{Ar}), 127.8 (d, CH_{Ar}), 128.39 (d, CH_{Ar}), 128.41 (d, CH_{Ar}), 128.8 (d, CH_{Ar}), 128.86 (d, CH_{Ar}), 136.1 (s, C_{Ar}), 136.4 (s, C_{Ar}), 177.32 (s, C2), 177.6 (s, C2).

(3*R*,3a*R*,4*R*,7a*R*)-1-Benzyl-3-(2-hydroxypropyl)-4-((2,2,6,6-tetramethylpiperidin-1-yl)oxy)octahydro-2*H*-indol-2-one (12pA):

Prepared according to the general procedure, yield 233 mg (81%) as an inseparable 1:1 mixture of *trans*-diastereomers.

[R_f (hexanes/EtOAc 1:1) = 0.35]; IR (film); ν [cm^{-1}]: 3406 (br), 2965 (m), 2931 (s), 2869 (m), 1666 (s), 1496 (w), 1450 (m), 1375 (m), 1360 (m), 1311 (w), 1298 (w), 1258 (m), 1241 (w), 1207 (w), 1182 (w), 1132 (m), 1078 (w), 1045 (w), 1030 (w), 958 (w), 702 (m), 637 (w), 609 (w); MS (+ESI) m/z, (%): 465 (25, [M+Na$^+$]), 443 (100, [M+H$^+$]); HRMS (+ESI) m/z [$C_{27}H_{43}N_2O_3^+$]: calcd. 443.3268; found 443.3268.

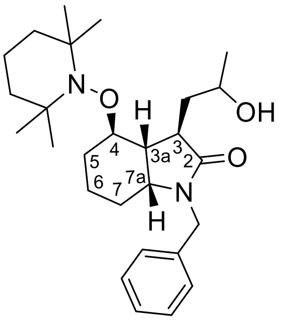

^{1}H NMR (400 MHz, $CDCl_3$): δ 1.07 (s, 12H, $NCCH_3$), 1.10 (s, 12H, $NCCH_3$), 1.25 (d, *J* = 6.4 Hz, 6H, CH_3), 1.27-1.37 (m, 4H, H6, H7, piperidine-H4), 1.40-1.47 (m, 8H, piperidine-H3, H5), 1.48-1.60 (m, 6H, H5, H6, H7, piperidine-H4), 1.61-1.70 (m, 2H, H6, H7), 1.71-1.75 (m, 5H, H5, $C\underline{H}_2CHOH$), 1.76-1.84 (m, 2H, H5, H7), 1.86-1.92 (m, 1H, $C\underline{H}_2CHOH$), 2.21-2.33 (m, 2H, H3a), 2.58 (td, *J* = 9.0, 5.1 Hz, 1H, H3), 2.73 (q, *J* = 6.9 Hz, 1H, H3), 3.53 (q, *J* = 7.1 Hz, 1H, H7a), 3.58 (q, *J* = 6.4 Hz, 1H, H7a), 3.81-3.86 (m, 1H, H4), 3.87-3.90 (m, 1H, H4), 3.93-3.99 (m, 1H, $C\underline{H}OH$), 4.00 (d, *J* = 15.2 Hz, 1H, $C\underline{H}_2Ph$), 4.03 (d, *J* = 15.0 Hz, 1H, $C\underline{H}_2Ph$), 4.13-4.20 (m, 1H, $C\underline{H}OH$), 4.88 (d, *J* = 15.0 Hz, 1H, $C\underline{H}_2Ph$), 4.91 (d, *J* = 15.2 Hz, 1H, $C\underline{H}_2Ph$), 5.27 (br. s, 1H, OH), 5.68 (br. s, 1H, OH), 7.18-7.24 (m, 4H, $Ar\underline{H}$), 7.27-7.36 (m, 6H, $Ar\underline{H}$); ^{13}C NMR (101 MHz, $CDCl_3$): δ 17.26 (t, piperidine-C4), 17.31 (t, piperidine-C4), 17.6 (t, C6), 18.0 (t, C6), 20.3 (q, $NC\underline{C}H_3$), 20.4 (q, $NC\underline{C}H_3$), 20.5 (q, $NC\underline{C}H_3$), 20.6 (q, $NC\underline{C}H_3$), 23.0 (q, CH_3), 24.3 (q, CH_3), 27.02 (t, C5), 27.04 (t, C5), 27.4 (t, C7), 27.8 (t, C7), 34.59 (q, $NC\underline{C}H_3$), 34.64 (q, $NC\underline{C}H_3$), 34.7 (q, $NC\underline{C}H_3$), 34.8 (q, $NC\underline{C}H_3$), 37.5 (t, $\underline{C}H_2CHOH$), 39.1 (t, $\underline{C}H_2CHOH$), 40.5 (t, piperidine-C3, C5), 40.6 (t, piperidine-C3, C5), 42.5 (d, C3), 43.5 (d, C3a), 43.9 (d, C3a), 44.4 (t, $\underline{C}H_2Ph$), 44.7 (t, $\underline{C}H_2Ph$), 44.8 (d, C3), 54.8 (d, C7a), 55.0 (d, C7a), 59.5 (s, CNO), 59.8 (s, CNO), 60.1 (s, CNO), 60.2 (s, CNO), 66.5 (d, CHOH), 68.0 (d, CHOH), 78.8 (d, C4), 79.5 (d, C4), 127.65 (d, CH_{Ar}), 127.73 (d, CH_{Ar}), 127.9 (d, CH_{Ar}), 128.0 (d, CH_{Ar}), 128.82 (d, CH_{Ar}), 128.84 (d, CH_{Ar}), 136.6 (s, C_{Ar}), 136.7 (s, C_{Ar}), 177.7 (s, C2), 177.9 (s, C2).

General procedure for the Dess–Martin oxidation of hydroxy lactams 12:

A solution of hydroxy lactam **12** or *trans*-**12** (0.7 mmol) in dichloromethane (4 mL) was added to a stirred solution of Dess–Martin periodinane (386 mg, 0.9 mmol) and *t*-BuOH (0.1 mL, 1.1 mmol) in dichloromethane (4 mL) at room temperature. After 30 min, saturated Na_2CO_3 solution (2 mL) and saturated $Na_2S_2O_3$ solution (2 mL) were added. After 5 min of vigorous stirring, the mixture was diluted with dichloromethane (10 mL), the organic layer was separated, washed with brine, dried over $MgSO_4$, and filtered. The filtrate was evaporated and the crude mixture was purified by column chromatography (gradient, hexanes/EtOAc 10:1 to 1:1) to give keto lactam **13**.

(3*R,4*R**)- and (3*S**,4*R**)-1-Allyl-3-(2-oxopropyl)-4-(((2,2,6,6-tetramethylpiperidin-1-yl)oxy)methyl)pyrrolidin-2-one (13b):**

Prepared according to the general procedure from lactam *trans*-**12b**, yield 216 mg (88%) as a single diastereomer, and from lactam **12b**, yield 211 mg (86%) as a separable 2.5:1 mixture of diastereomers.

[R_f (hexanes/EtOAc 1:1) = 0.41]; IR (film); ν [cm^{-1}]: 2974 (w), 2930 (m), 2871 (w), 1709 (m), 1692 (s), 1490 (w), 1417 (w), 1374 (w), 1359 (w), 1286 (w), 1263 (w), 1244 (w), 1209 (w), 1185 (w), 1163 (w), 1133 (w), 1046 (w), 1016 (w), 993 (w), 956 (w), 925 (w), 707 (w), 614 (w); MS (+ESI) m/z, (%): 723 (40, [$2M+Na^+$]), 373 (100, [$M+Na^+$]), 351 (30, [$M+H^+$]); HRMS (+ESI) m/z [$C_{20}H_{34}N_2O_3Na^+$]: calcd. 373.2462; found 373.2465.

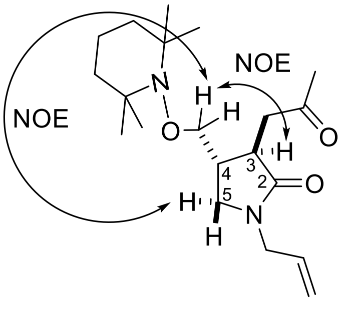

(3*R**,4*R**)

Major *trans*-diastereomer: ^1H NMR (400 MHz, $CDCl_3$): δ 1.06 (s, 6H, $NCCH_3$), 1.12 (s, 6H, $NCCH_3$), 1.24-1.35 (m, 1H, piperidine-H4), 1.39-1.46 (m, 4H, piperidine-H3, H5), 1.47-1.60 (m, 1H, piperidine-H4), 2.18 (s, 3H, CH_3), 2.36 (ttd, *J* = 8.6, 7.2, 6.4 Hz, 1H, H4), 2.66-2.73 (m, 1H, H3), 2.74-2.78 (m, 1H, CH_2CO), 2.88-2.94 (m, 1H, CH_2CO), 3.11 (dd, *J* = 9.8, 7.2 Hz, 1H, H5), 3.42 (dd, *J* = 9.8, 8.6 Hz, 1H, H5), 3.79 (dd, *J* = 8.9, 6.3 Hz, 1H, C<u>H</u>$_2$OTMP), 3.81 (dd, *J* = 8.9,

6.2 Hz, 1H, C$\underline{H}_2$OTMP), 3.82 (dd, J = 15.5, 6.1 Hz, 1H, C$\underline{H}_2$CH=), 3.95 (dd, J = 15.5, 5.9 Hz, 1H, C$\underline{H}_2$CH=), 5.19 (dd, J = 10.1, 1.3 Hz, 1H, CH=C$\underline{H}_2$), 5.22 (dd, J = 17.2, 1.3 Hz, 1H, CH=C$\underline{H}_2$), 5.73 (ddt, J = 17.2, 10.1, 6.0 Hz, 1H, C$\underline{H}$=CH_2); ^{13}C NMR (101 MHz, $CDCl_3$): δ 17.2 (t, piperidine-C4), 20.23 (q, NC$\underline{C}H_3$), 20.27 (q, NC$\underline{C}H_3$), 30.4 (q, CH_3), 33.26 (q, 2C, NC$\underline{C}H_3$), 37.4 (d, C4), 37.9 (t, piperidine-C3, C5), 41.5 (d, C3), 43.7 (t, $\underline{C}H_2$CO), 45.5 (t, $\underline{C}H_2$CH=), 48.32 (t, C5), 60.03 (s, 2C, CNO), 78.1 (t, $\underline{C}H_2$OTMP), 118.0 (t, CH=$\underline{C}H_2$), 132.4 (d, $\underline{C}$H=CH_2), 175.0 (s, C2), 206.8 (s, C=O).

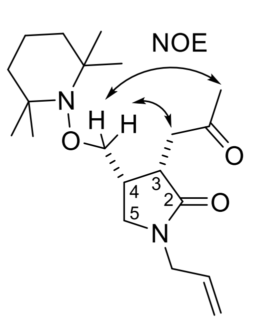

(3*S**,4*R**)

Minor *cis*-diastereomer: ^{1}H NMR (400 MHz, $CDCl_3$): δ 1.05 (s, 6H, $NCCH_3$), 1.08 (s, 6H, $NCCH_3$), 1.22-1.34 (m, 1H, piperidine-H4), 1.39-1.46 (m, 4H, piperidine-H3, H5), 1.47-1.60 (m, 1H, piperidine-H4), 2.18 (s, 3H, CH_3), 2.55 (dd, J = 18.5, 9.8 Hz, 1H, CH_2CO), 2.76-2.86 (m, 1H, H4), 2.99 (dd, J = 18.5, 4.3 Hz, 1H, CH_2CO), 3.07 (ddd, J = 9.8, 8.1, 4.3 Hz, 1H, H3), 3.28 (dd, J = 10.1, 1.7 Hz, 1H, H5), 3.39 (dd, J = 10.1, 6.5 Hz, 1H, H5), 3.53 (dd, J = 9.1, 5.1 Hz, 1H, C$\underline{H}_2$OTMP), 3.57 (dd, J = 9.1, 4.2 Hz, 1H, C$\underline{H}_2$OTMP), 3.81 (dd, J = 15.0, 6.1 Hz, 1H, C$\underline{H}_2$CH=), 3.94 (dd, J = 15.0, 6.0 Hz, 1H, C$\underline{H}_2$CH=), 5.19 (dd, J = 9.7, 1.4 Hz, 1H, CH=C$\underline{H}_2$), 5.20 (dd, J = 17.5, 1.4 Hz, 1H, CH=C$\underline{H}_2$), 5.70 (ddt, J = 17.5, 9.7, 6.0 Hz, 1H, C$\underline{H}$=CH_2); ^{13}C NMR (101 MHz, $CDCl_3$): δ 17.1 (t, piperidine-C4), 20.21 (q, NC$\underline{C}H_3$), 20.33 (q, NC$\underline{C}H_3$), 30.2 (q, CH_3), 33.0 (q, NC$\underline{C}H_3$), 33.34 (q, NC$\underline{C}H_3$), 34.2 (d, C4), 39.7 (t, piperidine-C3), 39.8 (t, piperidine-C5), 40.1 (d, C3), 40.2 (t, $\underline{C}H_2$CO), 45.6 (t, $\underline{C}H_2$CH=), 48.31 (t, C5), 59.98 (s, CNO), 60.01 (s, CNO), 75.3 (t, $\underline{C}H_2$OTMP), 118.5 (t, CH=$\underline{C}H_2$), 132.5 (d, $\underline{C}$H=CH_2), 174.6 (s, C2), 206.9 (s, C=O).

(3*R,4*R**)-1-Allyl-3-(2-oxohexyl)-4-(((2,2,6,6-tetramethylpiperidin-1-yl)oxy)methyl)pyrrolidin-2-one (13c):**

Prepared according to the general procedure, yield 238 mg (87%) as a single diastereomer.

[R_f (hexanes/EtOAc 2:1) = 0.40]; IR (film); ν [cm^{-1}]: 2973 (w), 2930 (m), 2872 (w), 1708 (m), 1685 (m), 1693 (s), 1489 (w), 1440 (w), 1416 (w), 1374 (w), 1359 (w), 1263 (w), 1208 (w), 1185 (w), 1132 (w), 1046 (w), 993 (w), 957 (w), 708 (w), 615 (w); MS (+ESI) m/z, (%): 807 (20, [$2M+Na^+$]), 415 (65, [$M+Na^+$]), 393 (100, [$M+H^+$]); HRMS (+ESI) m/z [$C_{23}H_{41}N_2O_3^+$]: calcd. 393.3112; found 393.3108.

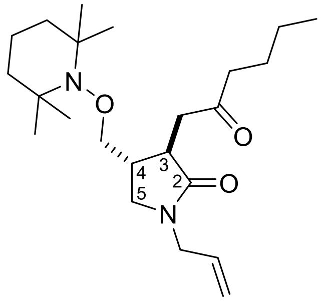

^{1}H NMR (400 MHz, $CDCl_3$): δ 0.89 (t, *J* = 7.3 Hz, 3H, CH_3), 1.06 (s, 6H, $NCCH_3$), 1.12 (s, 6H, $NCCH_3$), 1.25-1.29 (m, 1H, piperidine-H4), 1.30 (sext, *J* = 7.3 Hz, 2H, $CH_3C\underline{H}_2$), 1.39-1.45 (m, 4H, piperidine-H3, H5), 1.46-1.50 (m, 1H, piperidine-H4), 1.56 (quint, *J* = 7.4 Hz, 2H, $CH_3CH_2C\underline{H}_2$), 2.29-2.42 (m, 1H, H4), 2.43 (t, *J* = 7.4 Hz, 1H, $CH_3CH_2CH_2C\underline{H}_2$), 2.44 (t, *J* = 7.6 Hz, 1H, $CH_3CH_2CH_2C\underline{H}_2$), 2.68-2.71 (m, 1H, H3), 2.73 (dd, *J* = 15.0, 6.2 Hz, 1H, $CHC\underline{H}_2CO$), 2.89 (dd, *J* = 15.0, 2.7 Hz, 1H, $CHC\underline{H}_2CO$), 3.11 (dd, *J* = 9.8, 7.2 Hz, 1H, H5), 3.42 (dd, *J* = 9.8, 8.5 Hz, 1H, H5), 3.78 (dd, *J* = 9.1, 6.2 Hz, 1H, $C\underline{H}_2$OTMP), 3.81 (dd, *J* = 9.1, 5.3 Hz, 1H, $C\underline{H}_2$OTMP), 3.83 (dd, *J* = 15.3, 6.1 Hz, 1H, $C\underline{H}_2CH=$), 3.94 (dd, *J* = 15.3, 5.9 Hz, 1H, $C\underline{H}_2CH=$), 5.16-5.20 (m, 1H, $CH=C\underline{H}_2$), 5.21-5.26 (m, 1H, $CH=C\underline{H}_2$), 5.73 (ddt, *J* = 16.1, 10.1, 6.0 Hz, 1H, $C\underline{H}=CH_2$); ^{13}C NMR (101 MHz, $CDCl_3$): δ 14.0 (q, CH_3), 17.2 (t, piperidine-C4), 20.2 (q, $NC\underline{C}H_3$), 20.3 (q, $NC\underline{C}H_3$), 22.5 (t, $CH_3\underline{C}H_2$), 26.0 (t, $CH_3CH_2\underline{C}H_2$), 33.26 (q, $NC\underline{C}H_3$), 33.29 (q, $NC\underline{C}H_3$), 37.5 (d, C4), 39.7 (t, piperidine-C3, C5), 41.5 (d, C3), 42.8 (t, $CH\underline{C}H_2CO$), 43.0 (t, $CH_3CH_2CH_2\underline{C}H_2$), 45.5 (t, $\underline{C}H_2CH=$), 48.4 (t, C5), 60.0 (s, 2C, CNO), 78.2 (t, $\underline{C}H_2$OTMP), 118.0 (t, $CH=\underline{C}H_2$), 132.6 (d, $\underline{C}H=CH_2$), 175.1 (s, C2), 209.2 (s, C=O).

(3*R,4*R**)-1-Allyl-3-(2-oxo-2-phenylethyl)-4-(((2,2,6,6-tetramethylpiperidin-1-yl)oxy)methyl)pyrrolidin-2-one (13d):**

Prepared according to the general procedure, yield 231 mg (80%) as a single diastereomer.

[R_f (hexanes/EtOAc 2:1) = 0.46]; IR (film); ν [cm^{-1}]: 2974 (w), 2929 (w), 2871 (w), 1683 (s), 1598 (w), 1490 (w), 1448 (w), 1416 (w), 1380 (w), 1373 (w), 1360 (w), 1289 (w), 1263 (w), 1221 (w), 1183 (w), 1133 (w), 1046 (w), 995 (w), 925 (w), 755 (w), 691 (w); MS (+ESI) m/z, (%): 847 (20, [$2M+Na^+$]), 435 (65, [$M+Na^+$]), 413 (100, [$M+H^+$]); HRMS (+ESI) m/z [$C_{25}H_{37}N_2O_3^+$]: calcd. 413.2799; found 413.2795.

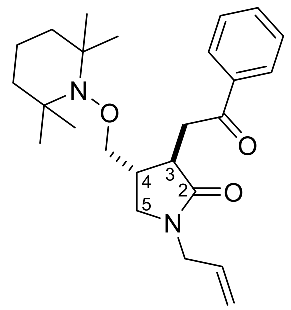

^{1}H NMR (400 MHz, $CDCl_3$): δ 1.02 (s, 6H, $NCCH_3$), 1.10 (s, 3H, $NCCH_3$), 1.11 (s, 3H, $NCCH_3$), 1.26-1.33 (m, 1H, piperidine-H4), 1.37-1.45 (m, 4H, piperidine-H3, H5), 1.46-1.57 (m, 1H, piperidine-H4), 2.40-2.51 (m, 1H, H4), 2.89 (td, *J* = 7.6, 4.0 Hz, 1H, H3), 3.19 (dd, *J* = 9.8, 6.6 Hz, 1H, H5), 3.24 (dd, *J* = 17.5, 7.6 Hz, 1H, CH_2CO), 3.49 (dd, *J* = 9.8, 6.7 Hz, 1H, H5), 3.57 (dd, *J* = 17.5, 4.0 Hz, 1H, CH_2CO), 3.82 (dd, *J* = 8.9, 6.6 Hz, 1H, $C\underline{H}_2OTMP$), 3.85 (dd, *J* = 8.9, 6.6 Hz, 1H, $C\underline{H}_2OTMP$), 3.89 (dd, *J* = 15.2, 6.0 Hz, 1H, $C\underline{H}_2CH=$), 3.97 (dd, *J* = 15.2, 6.0 Hz, 1H, $C\underline{H}_2CH=$), 5.19-5.22 (m, 1H, $CH=C\underline{H}_2$), 5.24-5.28 (m, 1H, $CH=C\underline{H}_2$), 5.76 (ddt, *J* = 16.1, 10.1, 6.0 Hz, 1H, $C\underline{H}=CH_2$), 7.42-7.47 (m, 2H, $Ar\underline{H}$), 7.52-7.59 (m, 1H, $Ar\underline{H}$), 7.94-8.00 (m, 2H, $Ar\underline{H}$); ^{13}C NMR (101 MHz, $CDCl_3$): δ 17.2 (t, piperidine-C4), 20.2 (q, 2C, $NC\underline{C}H_3$), 33.2 (q, $NC\underline{C}H_3$), 33.3 (q, $NC\underline{C}H_3$), 37.5 (d, C4), 39.3 (t, $\underline{C}H_2CO$), 39.7 (t, piperidine-C3, C5), 41.6 (d, C3), 45.6 (t, $\underline{C}H_2CH=$), 48.5 (t, C5), 60.0 (s, 2C, CNO), 78.2 (t, $\underline{C}H_2OTMP$), 118.0 (t, $CH=\underline{C}H_2$), 128.2 (d, CH_{Ar}), 128.7 (d, CH_{Ar}), 132.5 (d, $\underline{C}H=CH_2$), 133.2 (d, CH_{Ar}), 136.9 (s, C_{Ar}), 175.1 (s, C2), 198.2 (s, C=O).

(3*R,4*R**)- and (3*S**,4*R**)-1-Benzyl-4-methyl-3-(2-oxopropyl)-4-(((2,2,6,6-tetramethyl-piperidin-1-yl)oxy)methyl)pyrrolidin-2-one (13f):**

Prepared according to the general procedure, yield 261 mg (90%) as a partly separable 3:1 mixture of diastereomers.

[R_f (hexanes/EtOAc 1:1) = 0.42]; IR (film); ν [cm^{-1}]: 2971 (w), 2928 (m), 2873 (w), 1717 (m), 1690 (s), 1488 (w), 1430 (m), 1373 (m), 1359 (m), 1323 (w), 1295 (w), 1261 (w), 1244 (w), 1165 (w), 1133 (w), 1060 (w), 994 (w), 971 (w), 957 (w), 925 (w), 750 (w), 701 (m), 642 (w); MS (+ESI) m/z, (%): 415 (100, [$M+H^+$]); HRMS (+ESI) m/z [$C_{25}H_{39}N_2O_3^+$]: calcd. 415.2955; found 415.2954.

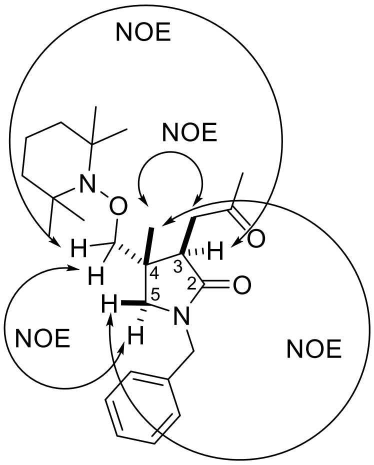

(3*R**,4*R**)

Major *trans*-diastereomer: ^{1}H NMR (400 MHz, $CDCl_3$): δ 0.96 (s, 3H, CCH_3), 1.03 (s, 3H, $NCCH_3$), 1.08 (s, 6H, $NCCH_3$), 1.10 (s, 3H, $NCCH_3$), 1.25-1.33 (m, 1H, piperidine-H4), 1.37-1.45 (m, 4H, piperidine-H3, H5), 1.46-1.58 (m, 1H, piperidine-H4), 2.27 (s, 3H, $COCH_3$), 2.49 (dd, *J* = 17.0, 5.2 Hz, 1H, CH_2CO), 2.80 (d, *J* = 9.7 Hz, 1H, H5), 2.83 (dd, *J* = 17.0, 7.9 Hz, 1H, CH_2CO), 3.20 (dd, *J* = 7.9, 5.2 Hz, 1H, H3), 3.30 (d, *J* = 9.7 Hz, 1H, H5), 3.64 (d, *J* = 8.8 Hz, 1H, $C\underline{H}_2OTMP$), 3.67 (d, *J* = 8.8 Hz, 1H, $C\underline{H}_2OTMP$), 4.25 (d, *J* = 14.8 Hz, 1H, $C\underline{H}_2Ph$), 4.57 (d, *J* = 14.8 Hz, 1H, $C\underline{H}_2Ph$), 7.21-7.36 (m, 5H, Ar$\underline{H}$); ^{13}C NMR (101 MHz, $CDCl_3$): δ 17.1 (t, piperidine-C4), 19.2 (q, $C\underline{C}H_3$), 20.3 (q, $NC\underline{C}H_3$), 20.4 (q, $NC\underline{C}H_3$), 30.7 (q, $CO\underline{C}H_3$), 33.2 (q, $NC\underline{C}H_3$), 33.3 (q, $NC\underline{C}H_3$), 39.8 (t, piperidine-C3), 39.88 (t, $\underline{C}H_2CO$, piperidine-C5), 40.5 (s, C4), 45.1 (d, C3), 46.9 (t, $\underline{C}H_2Ph$), 55.2 (t, C5), 60.2 (s, 2C, CNO), 81.7 (t, $\underline{C}H_2OTMP$), 127.7 (d, CH_{Ar}), 128.3 (d, CH_{Ar}), 128.8 (d, CH_{Ar}), 136.5 (s, C_{Ar}), 175.0 (s, C2), 206.8 (s, C=O).

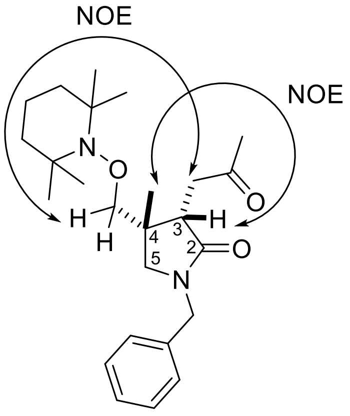

(3*S**,4*R**)

Minor *cis*-diastereomer: ^{1}H NMR (400 MHz, $CDCl_3$): δ 0.87 (s, 3H, $NCCH_3$), 0.99 (s, 3H, $NCCH_3$), 1.06 (s, 3H, $NCCH_3$), 1.11 (s, 3H, $NCCH_3$), 1.23 (s, 3H, CCH_3), 1.25-1.33 (m, 1H, piperidine-H4), 1.37-1.45 (m, 4H, piperidine-H3, H5), 1.46-1.58 (m, 1H, piperidine-H4), 2.26 (s, 3H, $COCH_3$), 2.39 (dd, J = 17.1, 6.3 Hz, 1H, CH_2CO), 2.86 (dd, J = 17.1, 6.7 Hz, 1H, CH_2CO), 2.91 (d, J = 10.5 Hz, 1H, H5), 2.96 (t, J = 6.5 Hz, 1H, H3), 3.406 (d, J = 10.5 Hz, 1H, H5), 3.415 (d, J = 8.4 Hz, 1H, $C\underline{H}_2OTMP$), 3.54 (d, J = 8.4 Hz, 1H, $C\underline{H}_2OTMP$), 4.25 (d, J = 14.8 Hz, 1H, $C\underline{H}_2Ph$), 4.57 (d, J = 14.8 Hz, 1H, $C\underline{H}_2Ph$), 7.21-7.36 (m, 5H, Ar$\underline{H}$); ^{13}C NMR (101 MHz, $CDCl_3$): δ 17.0 (t, piperidine-C4), 20.3 (q, $NC\underline{C}H_3$), 20.4 (q, $NC\underline{C}H_3$), 22.6 (q, $C\underline{C}H_3$), 30.8 (q, $CO\underline{C}H_3$), 33.1 (q, $NC\underline{C}H_3$), 33.2 (q, $NC\underline{C}H_3$), 38.7 (t, $\underline{C}H_2CO$), 39.88 (t, piperidine-C3), 39.92 (t, piperidine-C5), 40.6 (s, C4), 47.2 (t, $\underline{C}H_2Ph$), 47.9 (d, C3), 54.2 (t, C5), 60.2 (s, 2C, CNO), 78.0 (t, $\underline{C}H_2OTMP$), 127.8 (d, CH_{Ar}), 128.7 (d, CH_{Ar}), 128.9 (d, CH_{Ar}), 136.4 (s, C_{Ar}), 176.9 (s, C2), 207.0 (s, C=O).

(3*R*,4*R*)- and (3*S*,4*S*)-3-(2-Oxopropyl)-1-((*S*)-1-phenylethyl)-4-(((2,2,6,6-tetramethyl-piperidin-1-yl)oxy)methyl)pyrrolidin-2-one (13i):

Prepared according to the general procedure, yield 223 mg (77%) as a partly separable 1:1 mixture of diastereomers.

[R_f (hexanes/EtOAc 1:1) = 0.24]; IR (film); ν [cm^{-1}]: 2973 (w), 2930 (w), 2872 (w), 1717 (m), 1682 (s), 1488 (w), 1450 (w), 1430 (m), 1373 (m), 1358 (m), 1261 (w), 1244 (w), 1185 (w), 1162 (w), 1133 (w), 1047 (w), 1029 (w), 993 (w), 972 (w), 956 (w), 924 (w), 786 (w), 746 (w), 700 (m), 632 (w); MS (+ESI) m/z, (%): 437 (65, [M+Na^+]), 415 (100, [M+H^+]); HRMS (+ESI) m/z [$C_{25}H_{39}N_2O_3^+$]: calcd. 415.2955; found 415.2953.

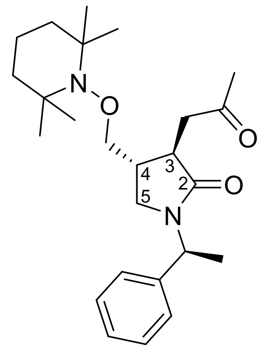 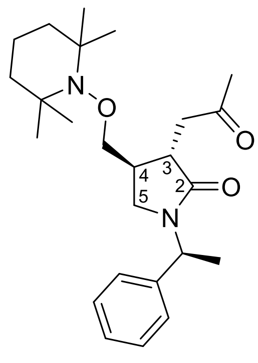

(3*R*,4*R*) (3*S*,4*S*)

Less polar diastereomer: ^{1}H NMR (400 MHz, $CDCl_3$): δ 1.05 (s, 6H, $NCCH_3$), 1.11 (s, 6H, $NCCH_3$), 1.28-1.35 (m, 1H, piperidine-H4), 1.38-1.47 (m, 4H, piperidine-H3, H5), 1.48-1.52 (m, 1H, piperidine-H4), 1.53 (d, *J* = 7.3 Hz, 3H, $PhCHC\underline{H}_3$), 2.20 (s, 3H, $COCH_3$), 2.22-2.34 (m, 1H, H4), 2.72-2.81 (m, 2H, H3, CH_2CO), 2.94 (dd, *J* = 19.2, 6.6 Hz, 1H, CH_2CO), 3.04 (dd, *J* = 9.4, 4.2 Hz, 1H, H5), 3.08 (dd, *J* = 9.4, 5.1 Hz, 1H, H5), 3.78 (d, *J* = 6.4 Hz, 2H, $C\underline{H}_2OTMP$), 5.48 (q, *J* = 7.3 Hz, 1H, $PhC\underline{H}CH_3$), 7.26-7.40 (m, 5H, $Ar\underline{H}$); ^{13}C NMR (101 MHz, $CDCl_3$): δ 16.3 (q, $PhCH\underline{C}H_3$), 17.2 (t, piperidine-C4), 20.25 (q, $NC\underline{C}H_3$), 20.34 (q, $NC\underline{C}H_3$), 30.4 (q, $CO\underline{C}H_3$), 33.3 (q, 2C, $NC\underline{C}H_3$), 37.2 (d, C4), 39.74 (t, piperidine-C3, C5), 42.02 (d, C3), 43.5 (t, $\underline{C}H_2CO$), 43.7 (t, C5), 49.1 (d, $Ph\underline{C}HCH_3$), 60.0 (s, 2C, CNO), 78.1 (t, $\underline{C}H_2OTMP$), 127.1 (d, CH_{Ar}), 127.51 (d, CH_{Ar}), 128.67 (d, CH_{Ar}), 140.3 (s, C_{Ar}), 174.7 (s, C2), 206.6 (s, C=O).

More polar diastereomer: ^{1}H NMR (400 MHz, $CDCl_3$): δ 0.97 (s, 9H, $NCCH_3$), 1.05 (s, 3H, $NCCH_3$), 1.23-1.33 (m, 1H, piperidine-H4), 1.35-1.43 (m, 4H, piperidine-H3, H5), 1.44-1.52 (m, 1H, piperidine-H4), 1.54 (d, *J* = 7.2 Hz, 3H, $PhCHC\underline{H}_3$), 2.19 (s, 3H, $COCH_3$), 2.23-2.38 (m, 1H, H4), 2.67 (ddd, *J* = 7.8, 6.4, 4.8 Hz, 1H, H3), 2.72 (dd, *J* = 9.8, 6.5 Hz, 1H, H5), 2.78 (dd, *J* = 17.6, 6.4 Hz, 1H, CH_2CO), 2.93 (dd, *J* = 17.6, 4.8 Hz, 1H, CH_2CO), 3.38 (dd, *J* = 9.8, 8.4 Hz, 1H, H5), 3.65 (dd, *J* = 9.0, 6.7 Hz, 1H, $C\underline{H}_2OTMP$), 3.68 (dd, *J* = 9.0, 6.5 Hz, 1H, $C\underline{H}_2OTMP$), 5.46 (q, *J* = 7.2 Hz, 1H, $PhC\underline{H}CH_3$), 7.21-7.37 (m, 5H, $Ar\underline{H}$); ^{13}C NMR (101 MHz, $CDCl_3$): δ 16.1 (q, $PhCH\underline{C}H_3$), 17.1 (t, piperidine-C4), 20.1 (q, $NC\underline{C}H_3$), 20.2 (q, $NC\underline{C}H_3$), 30.3 (q, $CO\underline{C}H_3$), 33.1 (q, 2C, $NC\underline{C}H_3$), 37.1 (d, C4), 39.68 (t, piperidine-C3, C5), 42.00 (d, C3), 43.8 (t, $\underline{C}H_2CO$), 43.9 (t, C5), 49.4 (d, $Ph\underline{C}HCH_3$), 59.9 (s, 2C, CNO), 78.0 (t, $\underline{C}H_2OTMP$), 127.2 (d, CH_{Ar}), 127.54 (d, CH_{Ar}), 128.65 (d, CH_{Ar}), 140.0 (s, C_{Ar}), 174.8 (s, C2), 206.8 (s, C=O).

(3*R*,4*R*)- and (3*S*,4*S*)- and (3*S*,4*R*)- and (3*R*,4*S*)-1-((*S*)-1-(Naphthalen-2-yl)ethyl)-3-(2-oxopropyl)-4-(((2,2,6,6-tetramethylpiperidin-1-yl)oxy)methyl)pyrrolidin-2-one (13j):

Prepared according to the general procedure, yield 255 mg (81%) as a partly separable 4:4:1:1 mixture of diastereomers. The minor diastereomer (3*S*,4*R*)-**13j** crystallized from hexane with a few drops of DCM and its configuration was determined by X-ray crystallography.

[R_f (hexanes/EtOAc 2:1) = 0.36]; IR (film); ν [cm^{-1}]: 2973 (w), 2930 (w), 2872 (w), 1716 (m), 1681 (s), 1486 (w), 1469 (w), 1427 (m), 1373 (m), 1359 (w), 1261 (w), 1245 (w), 1163 (w), 1131 (w), 1047 (w), 993 (w), 972 (w), 956 (w), 923 (w), 858 (w), 821 (w), 751 (m), 731 (m), 673 (w), 645 (w); MS (+ESI) m/z, (%): 951 (5, [$2M+Na^+$]), 487 (45, [$M+Na^+$]), 465 (100, [$M+H^+$]); HRMS (+ESI) m/z [$C_{29}H_{41}N_2O_3^+$]: calcd. 465.3112; found 465.3112.

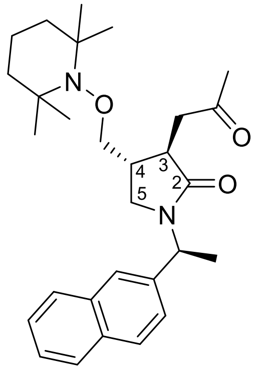

(3*R*,4*R*)

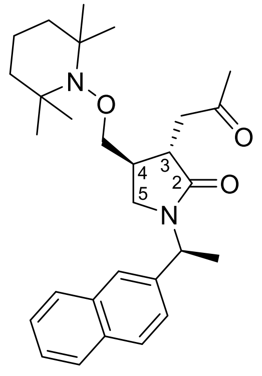

(3*S*,4*S*)

Major *trans*-diastereomers: ^1H NMR (400 MHz, $CDCl_3$): δ 0.88 (s, 6H, $NCCH_3$), 0.92 (s, 6H, $NCCH_3$), 1.03 (s, 3H, $NCCH_3$), 1.04 (s, 3H, $NCCH_3$), 1.10 (s, 3H, $NCCH_3$), 1.11 (s, 3H, $NCCH_3$), 1.22-1.29 (m, 2H, piperidine-H4), 1.30-1.39 (m, 8H, piperidine-H3, H5), 1.40-1.44 (m, 2H, piperidine-H4), 1.64 (d, *J* = 7.1 Hz, 3H, $ArCHC\underline{H}_3$), 1.67 (d, *J* = 7.1 Hz, 3H, $ArCHC\underline{H}_3$), 2.21 (s, 6H, $COCH_3$), 2.23-2.37 (m, 2H, H4), 2.68-2.75 (m, 1H, H3), 2.72 (dd, *J* = 9.8, 6.7 Hz, 1H, H5), 2.76-2.80 (m, 1H, H3), 2.78 (dd, *J* = 12.8, 6.1 Hz, 1H, $C\underline{H}_2CO$), 2.82 (dd, *J* = 17.5, 6.4 Hz, 1H, $C\underline{H}_2CO$), 2.95 (dd, *J* = 17.5, 4.9 Hz, 1H, $C\underline{H}_2CO$), 2.96 (dd, *J* = 12.8, 5.2 Hz, 1H, $C\underline{H}_2CO$), 3.03 (dd, *J* = 9.7, 8.4 Hz, 1H, H5), 3.09 (dd, *J* = 9.7, 7.6 Hz, 1H, H5), 3.42 (dd, *J* = 9.8, 8.4 Hz, 1H, H5), 3.62 (dd, *J* = 9.0, 6.7 Hz, 1H, $C\underline{H}_2OTMP$), 3.66 (dd, *J* = 9.0, 5.6 Hz, 1H, $C\underline{H}_2OTMP$), 3.77 (d, *J* = 6.4 Hz, 2H, $C\underline{H}_2OTMP$), 5.64 (q, *J* = 7.1 Hz, 2H, $ArC\underline{H}CH_3$), 7.38 (dd, *J* = 8.6, 1.8 Hz, 2H, $Ar\underline{H}$), 7.43-7.52 (m, 4H, $Ar\underline{H}$), 7.71-7.88 (m, 8H, $Ar\underline{H}$); ^{13}C NMR (101 MHz, $CDCl_3$): δ 16.0 (q, $ArCH\underline{C}H_3$), 16.1 (q, $ArCH\underline{C}H_3$), 17.1 (t, piperidine-C4), 17.2 (t, piperidine-C4), 20.08 (q, $NC\underline{C}H_3$), 20.13 (q, $NC\underline{C}H_3$), 20.27 (q, $NC\underline{C}H_3$), 20.33 (q, $NC\underline{C}H_3$), 30.39 (q, $CO\underline{C}H_3$), 30.44 (q, $CO\underline{C}H_3$), 33.0 (q, 2C, $NC\underline{C}H_3$), 33.17 (q, $NC\underline{C}H_3$), 33.24 (q,

$NC\underline{C}H_3$), 37.1 (d, C4), 37.2 (d, C4), 39.66 (t, piperidine-C3, C5), 39.72 (t, piperidine-C3, C5), 42.07 (d, C3), 42.12 (d, C3), 43.4 (t, $\underline{C}H_2CO$), 43.7 (t, C5), 43.8 (t, $\underline{C}H_2CO$), 43.89 (t, C5), 49.1 (d, $Ar\underline{C}HCH_3$), 49.5 (d, $Ar\underline{C}HCH_3$), 59.9 (s, 2C, CNO), 60.02 (s, 2C, CNO), 78.0 (t, $\underline{C}H_2OTMP$), 78.1 (t, $\underline{C}H_2OTMP$), 125.4 (d, CH_{Ar}), 125.6 (d, CH_{Ar}), 125.9 (d, CH_{Ar}), 126.0 (d, CH_{Ar}), 126.11 (d, CH_{Ar}), 126.3 (d, 2C, CH_{Ar}), 127.69 (d, 2C, CH_{Ar}), 127.72 (d, CH_{Ar}), 128.11 (d, CH_{Ar}), 128.13 (d, CH_{Ar}), 128.51 (d, CH_{Ar}), 128.53 (d, CH_{Ar}), 132.87 (s, C_{Ar}), 132.92 (s, C_{Ar}), 133.3 (s, C_{Ar}), 133.38 (s, C_{Ar}), 137.61 (s, C_{Ar}), 137.9 (s, C_{Ar}), 174.85 (s, C2), 174.89 (s, C2), 206.6 (s, C=O), 206.8 (s, C=O).

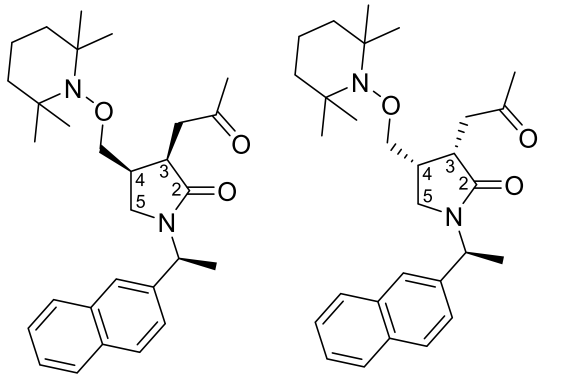

(3*R*,4*S*) (3*S*,4*R*)

Minor *cis*-diastereomers: ^1H NMR (400 MHz, $CDCl_3$): δ 0.91 (s, 3H, $NCCH_3$), 0.96 (s, 3H, $NCCH_3$), 1.04 (s, 6H, $NCCH_3$), 1.08 (s, 9H, $NCCH_3$), 1.12 (s, 3H, $NCCH_3$), 1.28-1.38 (m, 2H, piperidine-H4), 1.39-1.47 (m, 8H, piperidine-H3, H5), 1.48-1.54 (m, 2H, piperidine-H4), 1.65 (d, J = 7.1 Hz, 3H, $ArCHC\underline{H}_3$), 1.67 (d, J = 7.1 Hz, 3H, $ArCHC\underline{H}_3$), 2.19 (s, 3H, $COCH_3$), 2.21 (s, 3H, $COCH_3$), 2.50 (dd, J = 18.6, 10.1 Hz, 1H, $C\underline{H}_2CO$), 2.51-2.64 (m, 1H, H4), 2.68-2.79 (m, 2H, H3, H4), 2.96 (dd, J = 10.1, 6.4 Hz, 1H, H5), 3.00-3.10 (m, 4H, H5, $C\underline{H}_2CO$), 3.15 (ddd, J = 10.3, 4.9, 2.6 Hz, 1H, H3), 3.19 (dd, J = 8.7, 6.9 Hz, 1H, $C\underline{H}_2OTMP$), 3.30 (dd, J = 10.1, 2.7 Hz, 1H, H5), 3.31 (dd, J = 8.7, 5.5 Hz, 1H, $C\underline{H}_2OTMP$), 3.41 (dd, J = 9.8, 3.6 Hz, 1H, H5), 3.55 (dd, J = 8.9, 6.1 Hz, 1H, $C\underline{H}_2OTMP$), 3.60 (dd, J = 8.9, 5.7 Hz, 1H, $C\underline{H}_2OTMP$), 5.65 (q, J = 7.1 Hz, 2H, $ArC\underline{H}CH_3$), 7.36 (dd, J = 8.6, 2.0 Hz, 1H, $Ar\underline{H}$), 7.41 (dd, J = 8.4, 2.1 Hz, 1H, $Ar\underline{H}$), 7.45-7.50 (m, 4H, $Ar\underline{H}$), 7.71-7.88 (m, 8H, $Ar\underline{H}$); ^{13}C NMR (101 MHz, $CDCl_3$): δ 16.2 (q, $ArCH\underline{C}H_3$), 16.3 (q, $ArCH\underline{C}H_3$), 17.0 (t, piperidine-C4), 17.2 (t, piperidine-C4), 20.2 (q, 2C, $NC\underline{C}H_3$), 20.4 (q, 2C, $NC\underline{C}H_3$), 30.2 (q, $CO\underline{C}H_3$), 30.5 (q, $CO\underline{C}H_3$), 32.9 (q, 2C, $NC\underline{C}H_3$), 33.4 (q, 2C, $NC\underline{C}H_3$), 33.9 (d, C4), 34.0 (d, C4), 39.69 (t, piperidine-C3, C5), 39.8 (t, piperidine-C3, C5), 40.07 (t, $\underline{C}H_2CO$), 40.10 (t, $\underline{C}H_2CO$), 40.5 (d, C3), 40.6 (d, C3), 43.7 (t, C5), 43.92 (t, C5),

49.2 (d, Ar<u>C</u>HCH3), 49.4 (d, Ar<u>C</u>HCH3), 59.98 (s, 2C, CNO), 60.00 (s, 2C, CNO), 74.8 (t, <u>C</u>H2OTMP), 75.2 (t, <u>C</u>H2OTMP), 125.5 (d, CHAr), 125.7 (d, CHAr), 125.8 (d, 2C, CHAr), 126.11 (d, 2C, CHAr), 126.14 (d, CHAr), 126.2 (d, CHAr), 126.4 (d, CHAr), 127.65 (d, CHAr), 127.74 (d, CHAr), 128.11 (d, CHAr), 128.6 (d, CHAr), 128.7 (d, CHAr), 132.85 (s, CAr), 132.91 (s, CAr), 133.36 (s, CAr), 133.37 (s, CAr), 137.5 (s, CAr), 137.60 (s, CAr), 174.1 (s, C2), 174.5 (s, C2), 206.88 (s, C=O), 206.93 (s, C=O).

(3*R,4*S**)- and (3*S**,4*R**)- and (3*R**,4*R**)-1-Allyl-3-((*R**)-2-oxocyclohexyl)-4-(((2,2,6,6-tetramethylpiperidin-1-yl)oxy)methyl)pyrrolidin-2-one (13l):**

Prepared according to the general procedure, yield 229 mg (81%) as a partly separable 10:8.5(*trans*):1(*cis*) mixture of diastereomers.

[R*f* (hexanes/EtOAc 1:1) = 0.44]; IR (film); ν [cm^{-1}]: 2973 (w), 2931 (m), 2868 (w), 1722 (m), 1688 (s), 1488 (w), 1447 (w), 1373 (w), 1359 (w), 1307 (w), 1269 (w), 1132 (w), 1047 (w), 995 (w), 924 (w), 756 (w), 691 (w); MS (+ESI) m/z, (%): 803 (40, [2M+Na$^+$]), 413 (85, [M+Na$^+$]), 391 (100, [M+H$^+$]); HRMS (+ESI) m/z [$C_{23}H_{39}N_2O_3^+$]: calcd. 391.2955; found 391.2953.

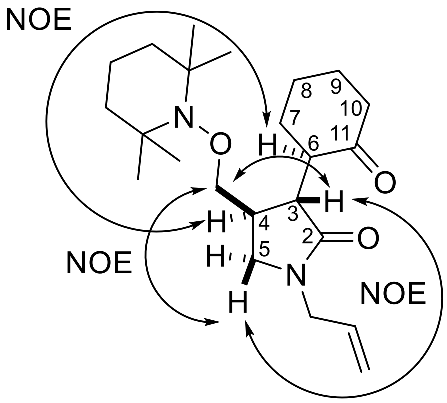

(3*R**,4*S**)

Less polar *trans*-diastereomer **13lA**: ^{1}H NMR (400 MHz, CDCl3): δ 1.07 (s, 6H, NCCH3), 1.15 (s, 6H, NCCH3), 1.27-1.36 (m, 1H, piperidine-H4), 1.39-1.52 (m, 4H, piperidine-H3, H5), 1.53-1.61 (m, 1H, piperidine-H4), 1.62-1.75 (m, 2H, H8, H9), 1.83-2.00 (m, 3H, H7, H8), 2.01-2.11 (m, 1H, H9), 2.24-2.46 (m, 3H, H4, H10), 2.64 (dd, *J* = 6.8, 2.4 Hz, 1H, H3), 2.86-2.95 (m, 1H, H6), 3.19 (dd, *J* = 9.8, 5.4 Hz, 1H, H5), 3.35-3.42 (m, 1H, H5), 3.79-3.91 (m, 3H, C<u>H</u>2CH=, C<u>H</u>2OTMP), 3.96 (dd, *J* = 15.2, 5.9 Hz, 1H, C<u>H</u>2CH=), 5.16-5.29 (m, 2H, CH=C<u>H</u>2), 5.68-5.81 (m, 1H, C<u>H</u>=CH2); ^{13}C NMR (101 MHz, CDCl3): δ 17.21 (t, piperidine-C4), 20.24 (q, NC<u>C</u>H3), 20.3 (q, NC<u>C</u>H3), 25.42 (t, C8), 27.27 (t, C9), 30.3 (t, C7), 33.27 (q, NC<u>C</u>H3), 33.40 (q, NC<u>C</u>H3),

35.0 (d, C4), 39.81 (t, piperidine-C3, C5), 42.2 (t, C10), 45.4 (d, C3), 45.5 (t, $\underline{C}H_2CH=$), 49.0 (t, C5), 51.8 (d, C6), 60.06 (s, 2C, CNO), 78.6 (t, $\underline{C}H_2$OTMP), 118.0 (t, CH=$\underline{C}H_2$), 132.6 (d, $\underline{C}H=CH_2$), 175.2 (s, C2), 210.8 (s, C11).

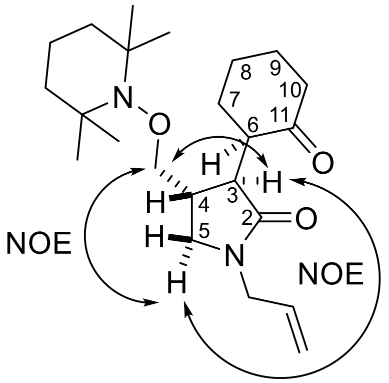

(3*S**,4*R**)

More polar *trans*-diastereomer **13lB**: ^{1}H NMR (400 MHz, $CDCl_3$): δ 1.07 (s, 6H, $NCCH_3$), 1.15 (s, 6H, $NCCH_3$), 1.27-1.36 (m, 1H, piperidine-H4), 1.40-1.47 (m, 4H, piperidine-H3, H5), 1.51-1.64 (m, 1H, piperidine-H4), 1.65-1.76 (m, 3H, H7, H8), 1.87-1.99 (m, 1H, H8), 2.01-2.13 (m, 2H, H9), 2.26-2.43 (m, 4H, H3, H4, H10), 3.01-3.09 (m, 1H, H6), 3.11 (dd, J = 9.5, 3.9 Hz, 1H, H5), 3.63 (t, J = 9.4 Hz, 1H, H5), 3.69 (dd, J = 9.1, 3.8 Hz, 1H, $C\underline{H}_2$OTMP), 3.74 (dd, J = 9.1, 4.1 Hz, 1H, $C\underline{H}_2$OTMP), 3.85 (dd, J = 15.3, 6.1 Hz, 1H, $C\underline{H}_2CH=$), 3.95 (dd, J = 15.3, 5.9 Hz, 1H, $C\underline{H}_2CH=$), 5.19 (dd, J = 10.1, 1.5 Hz, 1H, CH=$C\underline{H}_2$), 5.26 (dd, J = 17.2, 1.5 Hz, 1H, CH=$C\underline{H}_2$), 5.77 (ddt, J = 17.2, 10.1, 6.0 Hz, 1H, $C\underline{H}=CH_2$); ^{13}C NMR (101 MHz, $CDCl_3$): δ 17.17 (t, piperidine-C4), 20.43 (q, 2C, $NC\underline{C}H_3$), 25.43 (t, C8), 27.30 (t, C9), 32.1 (t, C7), 33.34 (q, $NC\underline{C}H_3$), 33.41 (d, C4), 39.80 (t, piperidine-C3, C5), 42.3 (t, C10), 45.6 (t, $\underline{C}H_2CH=$), 46.6 (d, C3), 49.3 (t, C5), 51.6 (d, C6), 60.14 (s, 2C, CNO), 79.6 (t, $\underline{C}H_2$OTMP), 117.7 (t, CH=$\underline{C}H_2$), 132.71 (d, $\underline{C}H=CH_2$), 174.8 (s, C2), 211.2 (s, C11).

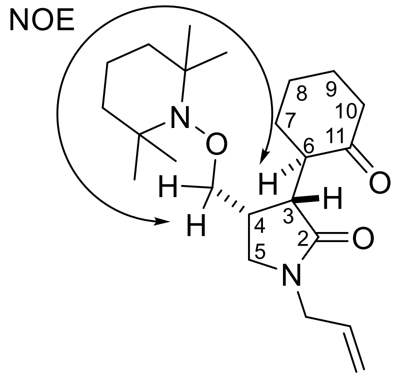

(3*R**,4*R**)

cis-Diastereomer: ^{1}H NMR (400 MHz, $CDCl_3$): δ 1.04 (s, 3H, $NCCH_3$), 1.05 (s, 6H, $NCCH_3$), 1.08 (s, 3H, $NCCH_3$), 1.27-1.33 (m, 1H, piperidine-H4), 1.37-1.45 (m, 5H, H7, piperidine-H3, H5), 1.48-1.57 (m, 1H, piperidine-H4), 1.60-1.72 (m, 2H, H8, H9), 1.84-1.90 (m, 1H, H8), 2.06-2.14 (m, 1H, H9), 2.31-2.38 (m, 2H, H10), 2.67 (ddd, *J* = 12.2, 9.1, 5.1 Hz, 1H, H6), 2.75 (dd, *J* = 9.0, 7.9 Hz, 1H, H3), 2.95-3.02 (m, 1H, H4), 3.09-3.19 (m, 1H, H7), 3.29-3.35 (m, 2H, H5), 3.44 (dd, *J* = 8.6, 4.5 Hz, 1H, $C\underline{H}_2OTMP$), 3.55 (dd, *J* = 10.4, 8.6 Hz, 1H, $C\underline{H}_2OTMP$), 3.78 (dd, *J* = 15.0, 6.3 Hz, 1H, $C\underline{H}_2CH=$), 3.94 (dd, *J* = 15.0, 6.0 Hz, 1H, $C\underline{H}_2CH=$), 5.18 (dd, *J* = 10.1, 1.9 Hz, 1H, $CH=C\underline{H}_2$), 5.21 (dd, *J* = 16.6, 1.9 Hz, 1H, $CH=C\underline{H}_2$), 5.71 (ddt, *J* = 16.6, 10.1, 6.2 Hz, 1H, $C\underline{H}=CH_2$); ^{13}C NMR (101 MHz, $CDCl_3$): δ 17.1 (t, piperidine-C4), 20.16 (q, $NC\underline{C}H_3$), 20.38 (q, $NC\underline{C}H_3$), 25.5 (t, C8), 29.0 (t, C9), 32.8 (q, $NC\underline{C}H_3$), 33.2 (t, C7), 33.43 (q, $NC\underline{C}H_3$), 34.8 (d, C4), 39.6 (t, piperidine-C3), 39.7 (t, piperidine-C5), 42.7 (t, C10), 43.4 (d, C3), 45.5 (t, $\underline{C}H_2CH=$), 48.0 (t, C5), 49.5 (d, C6), 59.9 (s, CNO), 60.0 (s, CNO), 75.1 (t, $\underline{C}H_2OTMP$), 118.4 (t, $CH=\underline{C}H_2$), 132.66 (d, $\underline{C}H=CH_2$), 174.6 (s, C2), 212.0 (s, C11).

(4*S,5*S**,6*R**)- and (4*S**,5*S**,6*S**)- and (4*R**,5*S**,6*R**)- and (4*R**,5*S**,6*S**)-2-Benzyl-4-(2-oxopropyl)-6-((2,2,6,6-tetramethylpiperidin-1-yl)oxy)-2-azaspiro[4.4]nonan-3-one (13m):**
Prepared according to the general procedure, yield 262 mg (81%) as a partly separable 11:6(*trans*):3:1(*cis*) mixture of diastereomers.
[R_f (hexanes/EtOAc 1:1) = 0.25]; IR (film); ν [cm^{-1}]: 2927 (w), 2871 (m), 1720 (m), 1689 (s), 1487 (w), 1429 (m), 1359 (m), 1284 (w), 1259 (w), 1238 (w), 1163 (w), 1133 (w), 1080 (w), 1029 (w), 975 (w), 910 (w), 731 (m), 701 (m), 645 (w); MS (+ESI) m/z, (%): 903 (10, [$2M+Na^+$]), 463 (30, [$M+Na^+$]), 441 (100, [$M+H^+$]); HRMS (+ESI) m/z [$C_{27}H_{41}N_2O_3^+$]: calcd. 441.3112; found 441.3111.

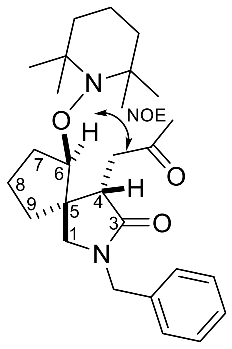

(4*S**,5*S**,6*R**)

Major diastereomer **13mA**: ^{1}H NMR (400 MHz, $CDCl_3$): δ 1.09 (s, 6H, $NCCH_3$), 1.15 (s, 6H, $NCCH_3$), 1.24-1.33 (m, 1H, piperidine-H4), 1.35-1.47 (m, 7H, H9, H8, piperidine-H3, H5), 1.48-1.60 (m, 2H, H8, piperidine-H4), 1.61-1.70 (m, 1H, H7), 1.88-1.98 (m, 1H, H7), 2.27 (s, 3H, $COCH_3$), 2.39 (dd, *J* = 17.1, 5.1 Hz, 1H, CH_2CO), 2.75 (d, *J* = 9.3 Hz, 1H, H1), 2.82 (dd, *J* = 17.1, 7.9 Hz, 1H, CH_2CO), 3.39 (dd, *J* = 7.9, 5.1 Hz, 1H, H4), 3.64 (d, *J* = 9.3 Hz, 1H, H1), 4.01-4.15 (m, 1H, H6), 4.23 (d, *J* = 14.8 Hz, 1H, $C\underline{H}_2Ph$), 4.67 (d, *J* = 14.8 Hz, 1H, $C\underline{H}_2Ph$), 7.21-7.35 (m, 5H, $Ar\underline{H}$); ^{13}C NMR (101 MHz, $CDCl_3$): δ 17.31 (t, piperidine-C4), 18.8 (t, C8), 20.6 (q, $NC\underline{C}H_3$), 20.8 (q, $NC\underline{C}H_3$), 29.1 (t, C7), 30.0 (t, C9), 30.7 (q, $CO\underline{C}H_3$), 34.3 (q, $NC\underline{C}H_3$), 34.80 (q, $NC\underline{C}H_3$), 40.1 (t, $\underline{C}H_2CO$), 40.5 (t, piperidine-C3, C5), 43.4 (d, C4), 46.9 (t, $\underline{C}H_2Ph$), 50.2 (s, C5), 51.4 (t, C1), 59.27 (s, CNO), 61.2 (s, CNO), 84.67 (d, C6), 127.59 (d, CH_{Ar}), 128.2 (d, CH_{Ar}), 128.76 (d, CH_{Ar}), 136.61 (s, C_{Ar}), 175.4 (s, C3), 206.7 (s, C=O).

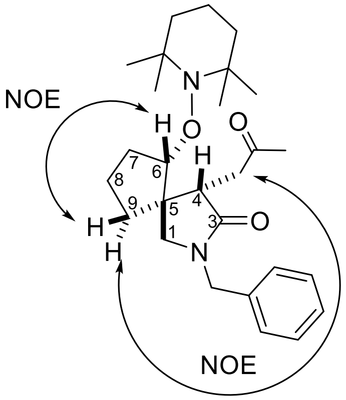

(4*S**,5*S**,6*S**)

Diastereomer **13mB**: ^{1}H NMR (400 MHz, $CDCl_3$): δ 0.97 (s, 6H, $NCCH_3$), 1.05 (s, 3H, $NCCH_3$), 1.16 (s, 3H, $NCCH_3$), 1.24-1.33 (m, 1H, piperidine-H4), 1.35-1.47 (m, 4H, piperidine-H3, H5), 1.48-1.60 (m, 3H, H8, piperidine-H4), 1.61-1.70 (m, 1H, H9), 1.76-1.86 (m, 2H, H9, H7), 2.06 (ddt, *J* = 13.6, 9.5, 6.5 Hz, 1H, H7), 2.24 (s, 3H, $COCH_3$), 2.67 (dd, *J* = 18.4, 8.9 Hz, 1H,

CH2CO), 2.88-2.99 (m, 2H, CH2CO, H4), 2.94 (d, *J* = 9.9 Hz, 1H, H1), 3.68 (d, *J* = 9.9 Hz, 1H, H1), 4.01-4.15 (m, 1H, H6), 4.04 (d, *J* = 14.7 Hz, 1H, CH2Ph), 4.91 (d, *J* = 14.7 Hz, 1H, CH2Ph), 7.21-7.35 (m, 5H, ArH); ^{13}C NMR (101 MHz, CDCl3): δ 17.2 (t, piperidine-C4), 19.6 (t, C8), 20.6 (q, NCCH3), 20.8 (q, NCCH3), 28.3 (t, C7), 30.59 (q, COCH3), 34.5 (q, NCCH3), 34.7 (q, NCCH3), 36.6 (t, C9), 40.78 (t, piperidine-C3), 40.92 (t, piperidine-C5), 41.1 (t, CH2CO), 46.2 (d, C4), 47.1 (t, CH2Ph), 50.7 (s, C5), 53.1 (t, C1), 59.4 (s, CNO), 60.9 (s, CNO), 84.72 (d, C6), 127.61 (d, CHAr), 128.3 (d, CHAr), 128.79 (d, CHAr), 136.64 (s, CAr), 175.3 (s, C3), 207.1 (s, C=O).

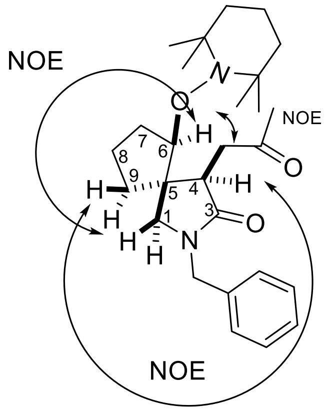

(4*R**,5*S**,6*R**)

Diastereomer **13mC**: ^{1}H NMR (400 MHz, CDCl3): δ 1.11 (s, 12H, NCCH3), 1.23-1.34 (m, 1H, piperidine-H4), 1.35-1.50 (m, 5H, H7, piperidine-H3, H5), 1.51-1.67 (m, 3H, H9, H7, piperidine-H4), 1.71-1.84 (m, 1H, H8), 1.85-1.90 (m, 1H, H9), 2.02-2.12 (m, 1H, H8), 2.27 (s, 3H, COCH3), 2.94 (dd, *J* = 7.4, 5.3 Hz, 1H, H4), 2.99 (d, *J* = 9.6 Hz, 1H, H1), 3.10 (dd, *J* = 17.4, 5.3 Hz, 1H, CH2CO), 3.14 (d, *J* = 9.6 Hz, 1H, H1), 3.23 (dd, *J* = 17.4, 7.4 Hz, 1H, CH2CO), 4.09 (d, *J* = 14.5 Hz, 1H, CH2Ph), 4.16 (t, *J* = 6.2 Hz, 1H, H6), 4.80 (d, *J* = 14.5 Hz, 1H, CH2Ph), 7.19-7.36 (m, 5H, ArH); ^{13}C NMR (101 MHz, CDCl3): δ 17.26 (t, piperidine-C4), 20.0 (t, C8), 21.1 (q, 2C, NCCH3), 30.56 (q, COCH3), 30.59 (t, C7), 34.21 (q, NCCH3), 34.4 (q, NCCH3), 35.2 (t, C9), 40.7 (t, piperidine-C3), 40.89 (t, piperidine-C5), 41.86 (t, CH2CO), 46.6 (d, C4), 47.0 (t, CH2Ph), 51.0 (s, C5), 57.1 (t, C1), 59.34 (s, CNO), 61.14 (s, CNO), 90.1 (d, C6), 127.70 (d, CHAr), 128.4 (d, CHAr), 128.81 (d, CHAr), 136.53 (s, CAr), 175.5 (s, C3), 207.9 (s, C=O).

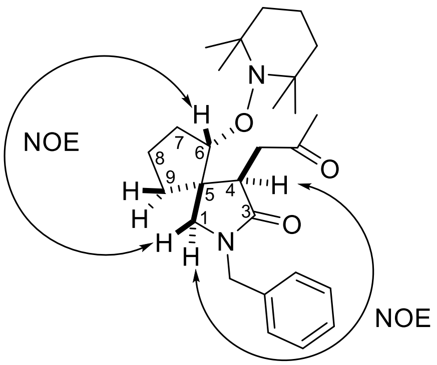

(4*R**,5*S**,6*S**)

Diastereomer **13mD**: ^{1}H NMR (400 MHz, $CDCl_3$): δ 1.00 (s, 3H, $NCCH_3$), 1.04 (s, 3H, $NCCH_3$), 1.07 (s, 3H, $NCCH_3$), 1.11 (s, 3H, $NCCH_3$), 1.22-1.33 (m, 1H, piperidine-H4), 1.35-1.58 (m, 7H, H7, H8, H9, piperidine-H3, H5), 1.60-1.71 (m, 3H, H7, H9, piperidine-H4), 2.17-2.25 (m, 1H, H8), 2.27 (s, 3H, $COCH_3$), 2.78 (dd, *J* = 15.0, 2.4 Hz, 1H, CH_2CO), 2.82 (d, *J* = 9.5 Hz, 1H, H1), 2.95 (d, *J* = 9.5 Hz, 1H, H1), 3.58-3.63 (m, 2H, H4, CH_2CO), 4.10-4.15 (m, 1H, H6), 4.36 (d, *J* = 14.7 Hz, 1H, $C\underline{H}_2Ph$), 4.49 (d, *J* = 14.7 Hz, 1H, $C\underline{H}_2Ph$), 7.19-7.36 (m, 5H, Ar$\underline{H}$); ^{13}C NMR (101 MHz, $CDCl_3$): δ 17.31 (t, piperidine-C4), 20.3 (t, C8), 20.7 (q, $NC\underline{C}H_3$), 21.2 (q, $NC\underline{C}H_3$), 28.4 (t, C7), 30.5 (q, $CO\underline{C}H_3$), 33.9 (q, $NC\underline{C}H_3$), 34.76 (q, $NC\underline{C}H_3$), 35.3 (t, C9), 40.6 (t, piperidine-C3), 40.77 (t, piperidine-C5), 41.91 (t, $\underline{C}H_2CO$), 42.4 (d, C4), 46.8 (t, $\underline{C}H_2Ph$), 51.5 (s, C5), 56.2 (t, C1), 59.34 (s, CNO), 61.13 (s, CNO), 89.7 (d, C6), 127.68 (d, CH_{Ar}), 128.3 (d, CH_{Ar}), 128.80 (d, CH_{Ar}), 136.51 (s, C_{Ar}), 175.9 (s, C3), 206.9 (s, C=O).

(4*S,5*S**,6*R**)- and (4*R**,5*S**,6*R**)- and (4*S**,5*S**,6*S**)-2-Benzyl-4-(2-oxopropyl)-6-((2,2,6,6-tetramethylpiperidin-1-yl)oxy)-2-azaspiro[4.5]decan-3-one (13n):**

Prepared according to the general procedure, yield 273 mg (88%) as a partly separable 5(*trans*):1(*cis*):1(*trans*) mixture of diastereomers. The major diastereomer **13nA** was transformed to the hydrochloride **13nA**·HCl (see p. S105), which crystallized from hexane with a few drops of DCM and its configuration was determined by X-ray crystallography.

[R_f (hexanes/EtOAc 1:1) = 0.38]; IR (film); ν [cm^{-1}]: 2927 (m), 2855 (w), 1719 (m), 1694 (s), 1486 (w), 1448 (m), 1432 (w), 1375 (m), 1359 (w), 1311 (w), 1270 (w), 1250 (w), 1166 (w), 1134 (w), 1080 (w), 1041 (w), 959 (w), 937 (w), 747 (w), 712 (m), 700 (w), 644 (w); MS (+ESI) m/z, (%): 931 (10, [$2M+Na^+$]), 477 (80, [$M+Na^+$]), 455 (100, [$M+H^+$]); HRMS (+ESI) m/z [$C_{28}H_{43}N_2O_3^+$]: calcd. 455.3268; found 455.3267.

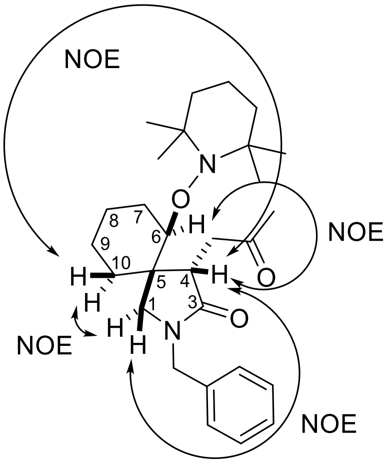

(4*S**,5*S**,6*R**)

Major diastereomer **13nA**: ^{1}H NMR (400 MHz, $CDCl_3$): δ 0.96 (s, 6H, $NCCH_3$), 1.00-1.13 (m, 4H, H7, H8, H9, H10), 1.17 (s, 3H, $NCCH_3$), 1.23 (s, 3H, $NCCH_3$), 1.24-1.32 (m, 1H, piperidine-H4), 1.33-1.50 (m, 6H, H9, H10, piperidine-H3, H5), 1.51-1.61 (m, 1H, piperidine-H4), 1.62-1.72 (m, 1H, H8), 2.29 (s, 3H, $COCH_3$), 2.31-2.38 (m, 1H, H7), 2.35 (dd, *J* = 16.6, 5.4 Hz, 1H, CH_2CO), 2.80 (dd, *J* = 16.6, 8.6 Hz, 1H, CH_2CO), 2.89 (d, *J* = 9.7 Hz, 1H, H1), 3.50 (d, *J* = 9.7 Hz, 1H, H1), 3.75 (dd, *J* = 10.9, 3.6 Hz, 1H, H6), 3.94 (dd, *J* = 8.6, 5.4 Hz, 1H, H4), 4.31 (d, *J* = 14.8 Hz, 1H, $C\underline{H}_2Ph$), 4.60 (d, *J* = 14.8 Hz, 1H, $C\underline{H}_2Ph$), 7.21-7.28 (m, 3H, Ar$\underline{H}$), 7.29-7.36 (m, 2H, Ar$\underline{H}$); ^{13}C NMR (101 MHz, $CDCl_3$): δ 17.4 (t, piperidine-C4), 20.73 (q, $NC\underline{C}H_3$), 21.0 (q, $NC\underline{C}H_3$), 22.0 (t, C9), 24.5 (t, C8), 28.2 (t, C7), 30.9 (q, $CO\underline{C}H_3$), 31.10 (t, C10), 34.5 (q, $NC\underline{C}H_3$), 34.7 (q, $NC\underline{C}H_3$), 39.6 (t, $\underline{C}H_2CO$), 40.60 (t, piperidine-C3), 41.0 (t, piperidine-C5), 43.3 (d, C4), 44.6 (s, C5), 46.86 (t, $\underline{C}H_2Ph$), 48.7 (t, C1), 59.25 (s, CNO), 61.2 (s, CNO), 80.7 (d, C6), 127.57 (d, CH_{Ar}), 128.2 (d, CH_{Ar}), 128.77 (d, CH_{Ar}), 136.6 (s, C_{Ar}), 175.5 (s, C3), 207.2 (s, C=O).

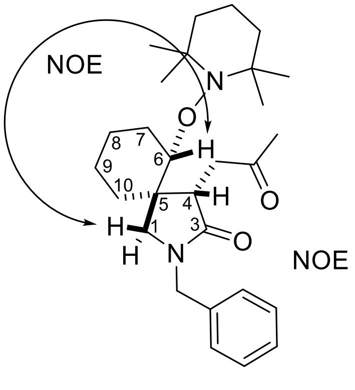

(4*S**,5*S**,6*S**)

Diastereomer **13nB**: ^{1}H NMR (400 MHz, $CDCl_3$): δ 0.96 (s, 6H, $NCCH_3$), 1.02-1.16 (m, 2H, H9, H10), 1.17 (s, 3H, $NCCH_3$), 1.23 (s, 3H, $NCCH_3$), 1.24-1.32 (m, 1H, piperidine-H4), 1.33-1.50 (m, 7H, H7, H8, H9, piperidine-H3, H5), 1.51-1.61 (m, 1H, piperidine-H4), 1.62-1.72 (m, 1H, H10), 1.73-1.79 (m, 1H, H8), 2.24-2.31 (m, 1H, H7), 2.26 (s, 3H, $COCH_3$), 2.76 (dd, *J* = 13.5, 7.7 Hz, 1H, CH_2CO), 2.86 (d, *J* = 9.4 Hz, 1H, H1), 3.36-3.38 (m, 2H, H4, CH_2CO), 3.39 (d, *J* = 9.4 Hz, 1H, H1), 3.69-3.73 (m, 1H, H6), 4.09 (d, *J* = 14.3 Hz, 1H, $C\underline{H}_2Ph$), 4.86 (d, *J* = 14.3 Hz, 1H, $C\underline{H}_2Ph$), 7.21-7.28 (m, 3H, Ar$\underline{H}$), 7.29-7.36 (m, 2H, Ar$\underline{H}$); ^{13}C NMR (101 MHz, $CDCl_3$): δ 17.4 (t, piperidine-C4), 20.73 (q, $NC\underline{C}H_3$), 20.94 (q, $NC\underline{C}H_3$), 23.5 (t, C9), 24.4 (t, C8), 27.0 (t, C7), 30.5 (q, $CO\underline{C}H_3$), 33.3 (t, C10), 34.5 (q, $NC\underline{C}H_3$), 34.7 (q, $NC\underline{C}H_3$), 40.60 (t, piperidine-C3), 41.0 (t, piperidine-C5), 42.0 (d, C4), 42.2 (t, $\underline{C}H_2CO$), 43.4 (s, C5), 46.93 (t, $\underline{C}H_2Ph$), 58.0 (t, C1), 59.25 (s, CNO), 61.2 (s, CNO), 86.7 (t, C6), 127.57 (d, CH_{Ar}), 128.5 (d, CH_{Ar}), 128.7 (d, CH_{Ar}), 136.6 (s, C_{Ar}), 175.5 (s, C3), 207.2 (s, C=O).

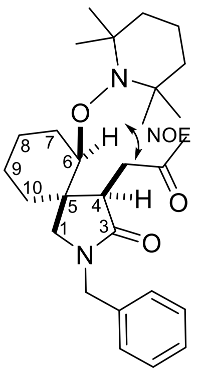

(4*R**,5*S**,6*R**)

Diastereomer **13nC**: ^{1}H NMR (400 MHz, $CDCl_3$): δ 0.95-1.07 (m, 2H, H7, H8), 0.99 (s, 3H, $NCCH_3$), 1.09 (s, 3H, $NCCH_3$), 1.20 (s, 3H, $NCCH_3$), 1.22 (s, 3H, $NCCH_3$), 1.24-1.33 (m, 1H, piperidine-H4), 1.34-1.51 (m, 7H, H7, H9, H10, piperidine-H3, H5), 1.52-1.62 (m, 2H, H10,

piperidine-H4), 1.67-1.75 (m, 1H, H8), 2.32 (s, 3H, $COCH_3$), 2.49-2.56 (m, 1H, H9), 2.62 (dd, *J* = 16.9, 5.9 Hz, 1H, CH_2CO), 2.92 (t, *J* = 6.4 Hz, 1H, H4), 2.97 (d, *J* = 10.3 Hz, 1H, H1), 3.33 (dd, *J* = 16.9, 7.0 Hz, 1H, CH_2CO), 3.53 (d, *J* = 10.3 Hz, 1H, H1), 3.70 (dd, *J* = 11.7, 3.8 Hz, 1H, H6), 3.71 (d, *J* = 14.4 Hz, 1H, $C\underline{H}_2Ph$), 5.23 (d, *J* = 14.4 Hz, 1H, $C\underline{H}_2Ph$), 7.20-7.24 (m, 2H, Ar$\underline{H}$), 7.26-7.36 (m, 3H, Ar$\underline{H}$); ^{13}C NMR (101 MHz, $CDCl_3$): δ 17.2 (t, piperidine-C4), 20.66 (q, $NC\underline{C}H_3$), 20.91 (q, $NC\underline{C}H_3$), 23.2 (t, C9), 24.8 (t, C8), 26.3 (t, C7), 31.11 (q, $CO\underline{C}H_3$), 34.0 (q, $NC\underline{C}H_3$), 34.2 (q, $NC\underline{C}H_3$), 38.0 (t, C10), 39.3 (t, $\underline{C}H_2CO$), 40.57 (t, piperidine-C3), 41.4 (t, piperidine-C5), 45.8 (s, C5), 47.4 (t, $\underline{C}H_2Ph$), 48.3 (d, C4), 52.3 (t, C1), 59.33 (s, CNO), 60.8 (s, CNO), 83.6 (d, C6), 127.59 (d, CH_{Ar}), 128.4 (d, CH_{Ar}), 128.80 (d, CH_{Ar}), 136.8 (s, C_{Ar}), 174.9 (s, C3), 208.3 (s, C=O).

(3*R,3a*R**,4*R**,6a*R**)- and (3*S**,3a*R**,4*R**,6a*R**)-1-Benzyl-3-(2-oxopropyl)-4-((2,2,6,6-tetramethylpiperidin-1-yl)oxy)hexahydrocyclopenta[*b*]pyrrol-2(1*H*)-one (13o):**

Prepared according to the general procedure, yield 265 mg (89%) as a partly separable 4:1 mixture of diastereomers.

[R_f (hexanes/EtOAc 1:1) = 0.29]; IR (film); ν [cm^{-1}]: 2971 (w), 2932 (m), 2867 (w), 1717 (m), 1684 (s), 1432 (m), 1359 (m), 1256 (w), 1182 (w), 1166 (w), 1133 (w), 1083 (w), 1029 (w), 962 (m), 740 (w), 700 (m), 646 (w); MS (+ESI) m/z, (%): 875 (20, $[2M+Na^+]$), 449 (10, $[M+Na^+]$), 427 (100, $[M+H^+]$); HRMS (+ESI) m/z [$C_{26}H_{39}N_2O_3^+$]: calcd. 427.2955; found 427.2956.

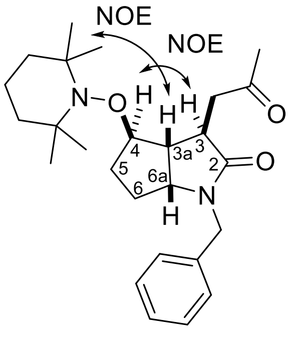

(3*R**,3a*R**,4*R**,6a*R**)

Major diastereomer **13oA**: ^{1}H NMR (400 MHz, $CDCl_3$): δ 1.02 (s, 6H, $NCCH_3$), 1.10 (s, 3H, $NCCH_3$), 1.12 (s, 3H, $NCCH_3$), 1.23-1.34 (m, 1H, piperidine-H4), 1.39-1.46 (m, 4H, piperidine-H3, H5), 1.47-1.54 (m, 1H, piperidine-H4), 1.55-1.61 (m, 1H, H5), 1.62-1.66 (m, 1H, H6), 1.68-1.75 (m, 1H, H6), 1.87-2.00 (m, 1H, H5), 2.19 (s, 3H, $COCH_3$), 2.46 (td, *J* = 6.6, 4.0 Hz, 1H, H3), 2.58-2.62 (m, 1H, H3a), 2.86 (dd, *J* = 17.7, 6.6 Hz, 1H, CH_2CO), 2.96 (dd, *J* = 17.7, 4.0 Hz, 1H, CH_2CO), 3.86-3.95 (m, 1H, H6a), 3.98 (d, *J* = 15.3 Hz, 1H, $C\underline{H}_2Ph$), 4.13-4.18 (m, 1H, H4),

4.93 (d, *J* = 15.3 Hz, 1H, C<u>H</u>$_2$Ph), 7.27-7.30 (m, 3H, Ar<u>H</u>), 7.32-7.36 (m, 2H, Ar<u>H</u>); ^{13}C NMR (101 MHz, CDCl$_3$): δ 17.27 (t, piperidine-C4), 20.3 (q, 2C, NC<u>C</u>H$_3$), 27.9 (t, C6), 29.6 (t, C5), 30.4 (q, CH$_3$), 34.6 (q, NC<u>C</u>H$_3$), 35.0 (q, NC<u>C</u>H$_3$), 40.31 (t, piperidine-C3, C5), 42.8 (d, C3), 45.0 (t, <u>C</u>H$_2$CO), 45.6 (t, <u>C</u>H$_2$Ph), 47.9 (d, C3a), 59.5 (s, CNO), 60.1 (s, CNO), 60.5 (d, C6a), 90.6 (d, C4), 127.6 (d, CH$_{Ar}$), 128.33 (d, CH$_{Ar}$), 128.7 (d, CH$_{Ar}$), 136.6 (s, C$_{Ar}$), 175.3 (s, C2), 206.4 (s, C=O).

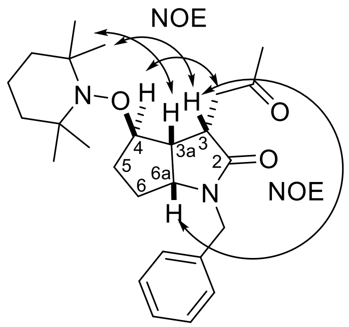

(3*S**,3a*R**,4*R**,6a*R**)

Minor diastereomer **13oB**: ^{1}H NMR (400 MHz, CDCl$_3$): δ 0.98 (s, 3H, NCCH$_3$), 1.05 (s, 3H, NCCH$_3$), 1.06 (s, 3H, NCCH$_3$), 1.09 (s, 3H, NCCH$_3$), 1.27-1.33 (m, 1H, piperidine-H4), 1.36-1.47 (m, 4H, piperidine-H3, H5), 1.48-1.56 (m, 1H, piperidine-H4), 1.63-1.71 (m, 2H, H5, H6), 1.73-1.82 (m, 1H, H6), 1.96-2.06 (m, 1H, H5), 2.24 (s, 3H, COCH$_3$), 2.78 (dd, *J* = 18.2, 8.8 Hz, 1H, CH$_2$CO), 2.84 (ddd, *J* = 10.1, 7.4, 4.6 Hz, 1H, H3a), 3.07 (dd, *J* = 18.2, 4.4 Hz, 1H, CH$_2$CO), 3.30 (ddd, *J* = 10.1, 8.8, 4.4 Hz, 1H, H3), 3.82-3.88 (m, 1H, H6a), 3.89 (d, *J* = 14.8 Hz, 1H, C<u>H</u>$_2$Ph), 4.08 (q, *J* = 5.0 Hz, 1H, H4), 4.97 (d, *J* = 14.8 Hz, 1H, C<u>H</u>$_2$Ph), 7.17-7.23 (m, 2H, Ar<u>H</u>), 7.24-7.35 (m, 3H, Ar<u>H</u>); ^{13}C NMR (101 MHz, CDCl$_3$): δ 17.30 (t, piperidine-C4), 20.4 (q, NC<u>C</u>H$_3$), 20.6 (q, NC<u>C</u>H$_3$), 27.1 (t, C6), 30.0 (t, C5), 30.3 (q, CH$_3$), 34.46 (q, NC<u>C</u>H$_3$), 34.52 (q, NC<u>C</u>H$_3$), 39.3 (d, C3), 40.26 (t, piperidine-C3), 40.33 (t, piperidine-C5), 41.4 (t, <u>C</u>H$_2$CO), 45.4 (d, C3a), 59.3 (s, CNO), 60.0 (d, C6a), 60.3 (s, CNO), 85.2 (d, C4), 127.7 (d, CH$_{Ar}$), 128.34 (d, CH$_{Ar}$), 128.8 (d, CH$_{Ar}$), 136.4 (s, C$_{Ar}$), 175.1 (s, C2), 206.6 (s, C=O).

(3*R,4*R**)-1-Allyl-4-(hydroxymethyl)-3-(2-hydroxypropyl)pyrrolidin-2-one (*trans*-14):**

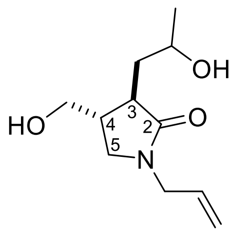

Lactam *trans*-**12b** (100 mg, 0.28 mmol), acetic acid (2 mL), water (1 mL), THF (1 mL), and zinc dust (740 mg, 11.4 mmol) were heated to 60 °C for 2 h. The reaction mixture was diluted with diethyl ether and filtered through a plug of silica gel. The solvent was evaporated, the crude product was dissolved in diethyl ether, washed with brine, dried over $MgSO_4$, and filtered. The filtrate was evaporated and the crude mixture was purified by column chromatography (gradient, hexanes/EtOAc 1:1 to pure EtOAc) to give 51 mg (82%) *trans*-**14** as an inseparable 1:1 mixture of diastereomers.

[R_f (EtOAc/MeOH 40:1) = 0.45]; IR (film); ν [cm^{-1}]: 3371 (br), 2966 (w), 2929 (w), 1659 (s), 1494 (w), 1453 (w), 1419 (w), 1375 (w), 1270 (w), 1135 (w), 1056 (w), 1029 (w), 994 (w), 937 (w), 686 (w), 621 (w); MS (+ESI) m/z, (%): 449 (10, [$2M+Na^+$]), 236 (100, [$M+Na^+$]); HRMS (+ESI) m/z [$C_{11}H_{19}NO_3Na^+$]: calcd. 236.1257; found 236.1255; ^{1}H NMR (400 MHz, $CDCl_3$): δ 1.22 (d, *J* = 6.2 Hz, 6H, CH_3), 1.54-1.63 (m, 1H, C$\underline{H}_2$CHOH), 1.64 (ddd, *J* = 14.3, 5.6, 2.7 Hz, 1H, C$\underline{H}_2$CHOH), 1.77 (ddd, *J* = 14.4, 9.7, 8.5 Hz, 1H, C$\underline{H}_2$CHOH), 1.96 (ddd, *J* = 14.3, 6.3, 3.2 Hz, 1H, C$\underline{H}_2$CHOH), 2.19-2.28 (m, 1H, H4), 2.29-2.39 (m, 1H, H4), 2.53-2.60 (m, 2H, H3), 3.08 (dd, *J* = 10.0, 7.0 Hz, 1H, H5), 3.16 (dd, *J* = 10.0, 7.5 Hz, 1H, H5), 3.32 (br. s, 4H, OH), 3.40 (dd, *J* = 10.0, 5.7 Hz, 1H, H5), 3.42 (dd, *J* = 10.0, 5.8 Hz, 1H, H5), 3.63-3.73 (m, 4H, C$\underline{H}_2$OH), 3.85-3.90 (m, 4H, C$\underline{H}_2$CH=), 3.93-4.00 (m, 1H, C$\underline{H}$OH), 4.06-4.14 (m, 1H, C$\underline{H}$OH), 5.14-5.22 (m, 4H, CH=C$\underline{H}_2$), 5.63-5.78 (m, 2H, C$\underline{H}$=CH_2); ^{13}C NMR (101 MHz, $CDCl_3$): δ 23.6 (q, CH_3), 24.1 (q, CH_3), 40.0 (t, $\underline{C}H_2$CHOH), 40.7 (t, $\underline{C}H_2$CHOH), 40.9 (d, C4), 41.0 (d, C4), 42.6 (d, C3), 44.1 (d, C3), 45.6 (t, $\underline{C}H_2$CH=), 45.7 (t, $\underline{C}H_2$CH=), 48.2 (t, C5), 48.5 (t, C5), 63.4 (t, $\underline{C}H_2$OH), 64.0 (t, $\underline{C}H_2$OH), 67.0 (d, CHOH), 67.2 (d, CHOH), 118.3 (t, CH=$\underline{C}H_2$), 118.5 (t, CH=$\underline{C}H_2$), 131.9 (d, $\underline{C}$H=CH_2), 132.1 (d, $\underline{C}$H=CH_2), 176.7 (s, C2), 177.1 (s, C2).

((3*R,4*R**)-1-Allyl-3-(((2,2,6,6-tetramethylpiperidin-1-yl)oxy)methyl)-4-(2-hydroxypropyl)pyrrolidine (*trans*-15):**

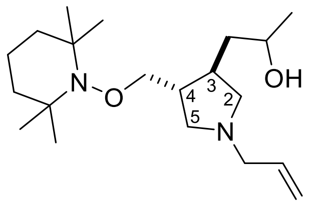

Lithium aluminum hydride solution (1 M in THF, 1.7 mL, 1.7 mmol) was added dropwise by syringe to dry THF (4.5 mL). A solution of lactam *trans*-**12b** (200 mg, 0.57 mmol) in dry THF (3 mL) was added dropwise by syringe at 0 °C and the reaction mixture was stirred for 1 h. The reaction was quenched by methanol (2 mL), diluted with ethyl acetate (10 mL), and filtered through a pad of silica gel, which was washed with a fresh portion of ethyl acetate. The filtrate was evaporated and the crude mixture was purified by flash chromatography (gradient, $CHCl_3$/MeOH 50:1 to 10:1) to give 190 mg (95%) *trans*-**15** as an inseparable 1:1 mixture of diastereomers.

[R_f(EtOAc) = 0.22]; IR (film); ν [cm^{-1}]: 3355 (br), 2967 (m), 2927 (s), 2871 (w), 1470 (w), 1452 (w), 1373 (m), 1359 (m), 1262 (w), 1244 (w), 1208 (w), 1184 (w), 1133 (s), 1046 (s), 994 (m), 955 (m), 923 (m), 874 (m), 788 (w), 715 (w), 645 (w), 605 (w); MS (+ESI) m/z, (%): 361 (10, [$M+Na^+$]), 339 (100, [$M+H^+$]); HRMS (+ESI) m/z [$C_{20}H_{39}N_2O_2^+$]: calcd. 339.3006; found 339.3006; ^{1}H NMR (400 MHz, $CDCl_3$): δ 1.08 (s, 12H, $NCCH_3$), 1.13 (s, 12H, $NCCH_3$), 1.18 (d, *J* = 6.4 Hz, 3H, CH_3), 1.20 (d, *J* = 6.4 Hz, 3H, CH_3), 1.26-1.34 (m, 2H, piperidine-H4), 1.40-1.48 (m, 8H, piperidine-H3, H5), 1.49-1.58 (m, 4H, C$\underline{H}_2$CHOH, piperidine-H4), 1.59-1.64 (m, 1H, C$\underline{H}_2$CHOH), 1.72 (ddd, *J* = 13.8, 6.0, 3.9 Hz, 1H, C$\underline{H}_2$CHOH), 2.15-2.27 (m, 2H, H3, H4), 2.28-2.37 (m, 2H, H3, H4), 2.47 (dd, *J* = 10.6, 7.3 Hz, 1H, H5), 2.59 (dd, *J* = 10.0, 8.6 Hz, 1H, H5), 2.78-2.90 (m, 3H, H2), 3.05-3.15 (m, 2H, H2, H5), 3.19 (dd, *J* = 10.0, 7.7 Hz, 1H, H5), 3.23-3.41 (m, 4H, C$\underline{H}_2$CH=), 3.70-3.84 (m, 4H, C$\underline{H}_2$OTMP), 3.85-3.89 (m, 1H, C$\underline{H}$OH), 3.90-3.96 (m, 1H, C$\underline{H}$OH), 5.22-5.35 (m, 4H, CH=C$\underline{H}_2$), 5.54 (br. s, 2H, OH), 5.89-6.08 (m, 2H, C$\underline{H}$=CH_2); ^{13}C NMR (101 MHz, $CDCl_3$): δ 17.2 (t, 2C, piperidine-C4), 20.4 (q, 3C, NC$\underline{C}H_3$), 20.5 (q, NC$\underline{C}H_3$), 23.9 (q, CH_3), 24.1 (q, CH_3), 33.2 (q, NC$\underline{C}H_3$), 33.3 (q, 3C, NC$\underline{C}H_3$), 38.2 (d, C4), 38.6 (d, C4), 39.79 (t, piperidine-C3, C5), 39.83 (t, piperidine-C3, C5), 42.0 (d, C3), 42.9 (d, C3), 43.1 (t, $\underline{C}H_2$CHOH), 43.3 (t, $\underline{C}H_2$CHOH), 55.9 (t, C5), 56.6 (t, C5), 58.4 (t, 2C, $\underline{C}H_2$CH=), 59.1 (t, C2), 59.3 (t, C2), 60.1 (s, 4C, CNO), 65.6 (d, CHOH), 66.7 (d, CHOH), 78.1

(t, C̲H2OTMP), 78.2 (t, C̲H2OTMP), 120.4 (t, CH=C̲H2), 121.1 (t, CH=C̲H2), 131.5 (d, C̲H=CH2), 132.2 (d, C̲H=CH2).

(3a*R,4*R**,6a*R**)-6-Allyl-2-methyl-4-(((2,2,6,6-tetramethylpiperidin-1-yl)oxy)methyl)hexa-hydro-2*H*-furo[2,3-*b*]pyrrole (16):**

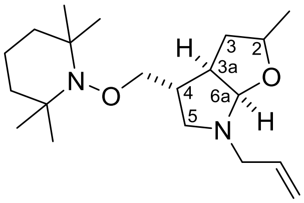

Sodium bis(2-methoxyethoxy)aluminum hydride (Red-Al, 70% solution in toluene, 246 mg, 0.85 mmol) was added dropwise by syringe to a stirred solution of lactam *trans*-**12b** (100 mg, 0.28 mmol) and *t*-BuOK (6.5 mg, 0.06 mmol) in dry THF (2 mL) at −10 °C and the reaction mixture was stirred at this temperature for 1 h. The reaction was quenched by saturated NH_4Cl solution and diluted with water (5 mL) and diethyl ether (5 mL). The organic layer was separated and the aqueous phase was extracted with diethyl ether (3 × 5 mL). The combined organic layers were dried over $MgSO_4$ and filtered. The filtrate was evaporated and the crude product was purified by flash chromatography (gradient, hexanes/EtOAc 50:1 to 20:1) to give 79 mg (83%) **16** as an inseparable 1:1 mixture of diastereomers.

[R_f (hexanes/EtOAc 10:1) = 0.44]; IR (film); ν [cm^{-1}]: 3004 (s), 2930 (s), 2871 (s), 1470 (w), 1448 (w), 1419 (w), 1373 (w), 1359 (w), 1335 (w), 1262 (w), 1245 (w), 1208 (w), 1171 (w), 1133 (w), 1083 (m), 1047 (w), 1013 (w), 994 (w), 957 (w), 918 (w), 859 (w), 710 (w); MS (+ESI) m/z, (%): 359 (15, [$M+Na^+$]), 337 (100, [$M+H^+$]); HRMS (+ESI) m/z [$C_{20}H_{37}N_2O_2^+$]: calcd. 337.2850; found 337.2846; ^{1}H NMR (400 MHz, $CDCl_3$): δ 1.08 (s, 12H, $NCCH_3$), 1.13 (s, 6H, $NCCH_3$), 1.14 (s, 6H, $NCCH_3$), 1.21-1.29 (m, 1H, H3), 1.24 (d, *J* = 6.0 Hz, 3H, CH_3), 1.27 (d, *J* = 6.1 Hz, 3H, CH_3), 1.30-1.35 (m, 2H, piperidine-H4), 1.39-1.46 (m, 8H, piperidine-H3, H5), 1.47-1.52 (m, 2H, piperidine-H4), 1.53 (ddd, *J* = 12.2, 10.8, 8.1 Hz, 1H, H3), 1.84 (dd, *J* = 12.3, 4.5 Hz, 1H, H3), 2.03-2.15 (m, 2H, H4), 2.22 (ddd, *J* = 12.2, 9.3, 5.8 Hz, 1H, H3), 2.35-2.50 (m, 3H, H3a, H5), 2.70 (dd, *J* = 9.0, 4.5 Hz, 1H, H5), 2.85-3.05 (m, 2H, H5), 3.21-3.53 (m, 4H, CH̲2CH=), 3.61-3.76 (m, 4H, CH̲2OTMP), 3.80-3.96 (m, 1H, H2), 4.10 (tq, *J* = 10.7, 5.9 Hz, 1H, H2), 4.82 (d, *J* = 4.4 Hz, 1H, H6a), 4.83 (d, *J* = 3.8 Hz, 1H, H6a), 5.04-5.25 (m, 4H, CH=CH̲2), 5.81-5.98 (m, 2H, CH̲=CH2); ^{13}C NMR (101 MHz, $CDCl_3$): δ 17.2 (t, 2C, piperidine-C4), 20.26 (q, 4C, NCC̲H3), 20.31 (q, CH_3), 20.8 (q, CH_3), 33.3 (q, 4C, NCC̲H3), 39.7 (t, 2C,

piperidine-C3), 39.8 (t, 2C, piperidine-C5), 40.5 (t, C3), 40.9 (t, C3), 43.1 (d, C4), 45.0 (d, C4), 46.3 (d, C3a), 46.7 (d, C3a), 52.9 (t, C5), 53.6 (t, <u>C</u>H2CH=), 54.4 (t, C5), 55.1 (t, <u>C</u>H2CH=), 59.92 (s, 2C, CNO), 59.94 (s, 2C, CNO), 73.7 (d, C2), 74.5 (d, C2), 78.5 (t, <u>C</u>H2OTMP), 78.9 (t, <u>C</u>H2OTMP), 98.9 (d, C6a), 99.1 (d, C6a), 116.4 (t, CH=<u>C</u>H2), 116.7 (t, CH=<u>C</u>H2), 136.0 (d, <u>C</u>H=CH2), 136.5 (d, <u>C</u>H=CH2).

(3*R,4*R**)-3-(2-Hydroxypropyl)-1-((*E*)-prop-1-en-1-yl)-4-(((2,2,6,6-tetramethylpiperidin-1-yl)oxy)methyl)pyrrolidin-2-one (S15):**

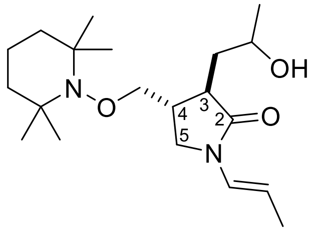

$ClRh(PPh_3)_3$ (52 mg, 0.06 mmol) was added to a solution of lactam *trans*-**12b** (250 mg, 0.7 mmol) in dry toluene (1.5 mL). The reaction mixture was heated at 120 °C for 14 h, cooled to room temperature, diluted with diethyl ether (5 mL), and filtered through a pad of silica gel, which was washed with a fresh portion of diethyl ether. The filtrate was evaporated and the crude product **S15** was used in the next step without further purification. Yield 195 mg (78%) as an inseparable 1:1 mixture of diastereomers.

[R_f (hexanes/EtOAc 1:1) = 0.46]; IR (film); ν [cm^{-1}]: 3373 (br), 2971 (m), 2929 (m), 1666 (s), 1454 (w), 1415 (m), 1375 (m), 1359 (w), 1332 (w), 1281 (w), 1244 (w), 1209 (w), 1185 (w), 1133 (w), 1047 (w), 953 (w), 790 (w), 724 (w), 695 (w), 623 (w); MS (+ESI) m/z, (%): 727 (25, [$2M+Na^+$]), 375 (100, [$M+Na^+$]), 353 (65, [$M+H^+$]); HRMS (+ESI) m/z [$C_{20}H_{36}N_2O_3Na^+$]: calcd. 375.2618; found 375.2615; ^1H NMR (400 MHz, $CDCl_3$): δ 1.10 (s, 12H, $NCCH_3$), 1.14 (s, 6H, $NCCH_3$), 1.15 (s, 6H, $NCCH_3$), 1.23 (d, *J* = 6.4 Hz, 3H, CHOHC<u>H</u>3), 1.24 (d, *J* = 6.0 Hz, 3H, CHOHC<u>H</u>3), 1.29-1.37 (m, 2H, piperidine-H4), 1.40-1.50 (m, 8H, piperidine-H3, H5), 1.51-1.61 (m, 2H, piperidine-H4), 1.65-1.84 (m, 3H, C<u>H</u>2CHOH), 1.73 (d, *J* = 7.3 Hz, 6H, C<u>H</u>3CH=), 1.91 (ddd, *J* = 14.7, 8.1, 3.4 Hz, 1H, C<u>H</u>2CHOH), 2.24-2.35 (m, 1H, H4), 2.37-2.48 (m, 1H, H4), 2.64 (dt, *J* = 9.4, 3.5 Hz, 1H, H3), 2.69 (dt, *J* = 10.2, 5.8 Hz, 1H, H3), 3.24 (dd, *J* = 10.0, 6.4 Hz, 1H, H5), 3.25 (dd, *J* = 10.2, 8.0 Hz, 1H, H5), 3.60 (dd, *J* = 10.2, 3.2 Hz, 1H, H5), 3.62 (dd, *J* = 10.0, 3.3 Hz, 1H, H5), 3.82 (dd, *J* = 17.0, 6.2 Hz, 2H, C<u>H</u>2OTMP), 3.88 (dd, *J* = 17.0, 5.7 Hz, 2H, C<u>H</u>2OTMP), 3.92-4.02 (m, 1H, C<u>H</u>OH), 4.06-4.14 (m, 1H, C<u>H</u>OH), 5.02 (dq, *J* = 14.2, 7.3 Hz,

2H, $CH_3C\underline{H}$=), 5.25 (br. s, 2H, OH), 6.85 (d, *J* = 14.2 Hz, 2H, $C\underline{H}$=$CHCH_3$); ^{13}C NMR (101 MHz, $CDCl_3$): δ 15.32 (q, $\underline{C}H_3CH$=), 15.34 (q, $\underline{C}H_3CH$=), 17.2 (t, 2C, piperidine-C4), 20.4 (q, 4C, $NC\underline{C}H_3$), 23.26 (q, $CHOH\underline{C}H_3$), 23.30 (q, $CHOH\underline{C}H_3$), 33.2 (q, $NC\underline{C}H_3$), 33.4 (q, 3C, $NC\underline{C}H_3$), 37.6 (d, C4), 38.5 (d, C4), 39.5 (t, $\underline{C}H_2CHOH$), 39.76 (t, piperidine-C3, C5), 39.82 (t, piperidine-C3, C5), 40.7 (t, $\underline{C}H_2CHOH$), 42.9 (d, C3), 45.9 (d, C3), 47.3 (t, C5), 47.4 (t, C5), 60.17 (s, 2C, CNO), 60.19 (s, 2C, CNO), 66.1 (d, CHOH), 68.0 (d, CHOH), 76.9 (t, $\underline{C}H_2OTMP$), 77.6 (t, $\underline{C}H_2OTMP$), 107.9 (d, $CH_3\underline{C}H$=), 108.4 (d, $CH_3\underline{C}H$=), 124.2 (d, $\underline{C}H$=$CHCH_3$), 124.4 (d, $\underline{C}H$=$CHCH_3$), 174.8 (s, C2), 175.3 (s, C2).

(3*R,4*R**)-3-(2-Hydroxypropyl)-4-(((2,2,6,6-tetramethylpiperidin-1-yl)oxy)methyl)pyrrolidin-2-one (*trans*-17):**

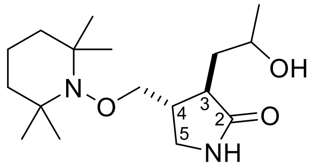

OsO_4 (2.5% solution in *t*-BuOH, 380 mg, 0.04 mmol) was added by syringe to a stirred solution of lactam **S15** (195 mg, 0.55 mmol) in THF (3 mL) at room temperature. After 5 min, a solution of $NaIO_4$ (179 mg, 0.88 mmol) in water (5 mL) was added by syringe and the reaction mixture was stirred at room temperature overnight. The reaction mixture was diluted with water (5 mL) and dichloromethane (10 mL). The organic layer was separated and the aqueous was extracted with dichloromethane (3 × 5 mL). The combined organic layers were dried over $MgSO_4$ and filtered. The filtrate was evaporated and the crude product was purified by flash chromatography (gradient, $CHCl_3$/MeOH 30:1 to 5:1) to give 130 mg (80%) *trans*-**17** as an inseparable 1:1 mixture of diastereomers.

[R_f(EtOAc) = 0.26]; IR (film); ν [cm^{-1}]: 3344 (br), 2972 (m), 2929 (s), 1669 (s), 1487 (w), 1436 (m), 1374 (m), 1360 (m), 1261 (m), 1208 (w), 1133 (m), 994 (m), 938 (w), 793 (w), 719 (w), 690 (w), 624 (w); MS (+ESI) m/z, (%): 335 (100, [$M+Na^+$]), 313 (35, [$M+H^+$]); HRMS (+ESI) m/z [$C_{17}H_3N_2O_3Na^+$]: calcd. 335.2305; found 335.2305; ^1H NMR (400 MHz, $CDCl_3$): δ 1.08 (s, 12H, $NCCH_3$), 1.14 (s, 12H, $NCCH_3$), 1.21 (d, *J* = 6.7 Hz, 6H, CH_3), 1.28-1.36 (m, 2H, piperidine-H4), 1.41-1.48 (m, 8H, piperidine-H3, H5), 1.49-1.59 (m, 2H, piperidine-H4), 1.60-1.74 (m, 2H, $C\underline{H}_2CHOH$), 1.75-1.83 (m, 1H, $C\underline{H}_2CHOH$), 1.84-1.94 (m, 1H, $C\underline{H}_2CHOH$), 2.19-2.35 (m, 2H, H4), 2.53-2.67 (m, 2H, H3), 3.35-3.42 (m, 2H, H5), 3.60-3.68 (m, 2H, H5), 3.78-3.87 (m, 4H, $C\underline{H}_2OTMP$), 3.92-4.00 (m, 2H, $C\underline{H}OH$), 4.82 (br. s, 1H, OH), 4.85 (br. s, 1H, OH), 5.34 (br. s,

1H, NH), 5.40 (br. s, 1H, NH); ^{13}C NMR (101 MHz, $CDCl_3$): δ 17.1 (t, 2C, piperidine-C4), 20.4 (br. q, 4C, $NC\underline{C}H_3$), 24.2 (q, $CHOH\underline{C}H_3$), 24.3 (q, $CHOH\underline{C}H_3$), 33.2 (q, 2C, $NC\underline{C}H_3$), 33.3 (q, 2C, $NC\underline{C}H_3$), 39.1 (d, C4), 39.4 (d, C4), 39.7 (t, piperidine-C3, C5), 39.8 (t, piperidine-C3, C5), 40.2 (t, $\underline{C}H_2CHOH$), 40.4 (t, $\underline{C}H_2CHOH$), 46.2 (d, C3), 46.5 (d, C3), 46.9 (t, C5), 47.3 (t, C5), 60.1 (s, 4C, CNO), 67.5 (d, CHOH), 68.1 (d, CHOH), 76.6 (t, $\underline{C}H_2OTMP$), 76.8 (t, $\underline{C}H_2OTMP$), 178.7 (s, C2), 179.4 (s, C2).

(3*R,3a*R**,6a*R**)- and (3*S**,3a*R**,6a*R**)-1-Benzyl-3-(2-hydroxypropyl)hexahydrocyclo-penta[*b*]pyrrole-2,4-dione (18):**

mCPBA (75 mg, 0.30 mmol) was added to a solution of lactam **12o** (100 mg, 0.23 mmol) in dry DCM (5 mL) at 0 °C under an argon atmosphere. The reaction mixture was stirred for 15 min and saturated Na_2SO_3 solution (5 mL) was added. The organic layer was separated and the aqueous phase was extracted with dichloromethane (3 × 5 mL). The combined organic layers were dried over $MgSO_4$ and filtered. The filtrate was evaporated and the crude product was purified by flash chromatography (gradient, hexane/EtOAc 3:1 to 1:4) to give 54 mg (81%) **18** as an inseparable 4:4:1:1 mixture of diastereomers.

[R_f(EtOAc) = 0.45]; IR (film); ν [cm^{-1}]: 3414 (br), 2965 (w), 2928 (w), 1741 (s), 1667 (s), 1440 (m), 1358 (w), 1344 (w), 1313 (w), 1264 (w), 1204 (w), 1182 (w), 1150 (w), 1132 (w), 1083 (w), 1056 (w), 1007 (w), 949 (w), 732 (w), 705 (m), 669 (w); MS (+ESI) m/z, (%): 310 (100, $[M+Na^+]$), 288 (40, $[M+H^+]$); HRMS (+ESI) m/z $[C_{17}H_{21}NO_3Na^+]$: calcd. 310.1414; found 310.1411.

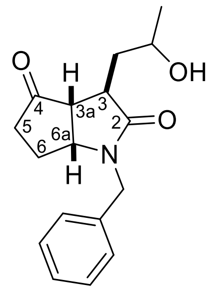

(3*R**,3a*R**,6a*R**)

Major diastereomers **18A**: ^{1}H NMR (400 MHz, $CDCl_3$): δ 1.25 (d, *J* = 6.2 Hz, 3H, CH_3), 1.27 (d, *J* = 6.2 Hz, 3H, CH_3), 1.60-1.74 (m, 2H, $C\underline{H}_2CHOH$), 1.76-1.88 (m, 4H, $C\underline{H}_2CHO\underline{H}$), 1.95-2.09 (m, 4H, H6), 2.17-2.33 (m, 4H, H5), 2.55 (dd, *J* = 7.3, 3.7 Hz, 1H, H3a), 2.73-2.85 (m, 3H, H3, H3a), 4.00-4.11 (m, 2H, $C\underline{H}OH$), 4.12-4.17 (m, 2H, H6a), 4.18 (d, *J* = 14.9 Hz, 1H, $C\underline{H}_2Ph$), 4.20 (d, *J* = 14.9 Hz, 1H, $C\underline{H}_2Ph$), 4.84 (d, *J* = 14.9 Hz, 1H, $C\underline{H}_2Ph$), 4.86 (d, *J* = 14.9 Hz, 1H, $C\underline{H}_2Ph$), 7.21-7.27 (m, 4H, $Ar\underline{H}$), 7.28-7.39 (m, 6H, $Ar\underline{H}$); ^{13}C NMR (101 MHz, $CDCl_3$): δ 23.6 (q, CH_3), 23.8 (q, CH_3), 25.3 (t, 2C, C6), 35.7 (t, C5), 35.8 (t, C5), 40.83 (t, $\underline{C}H_2CHOH$), 41.8 (t, $\underline{C}H_2CHOH$), 43.4 (d, C3), 44.2 (d, C3), 45.07 (t, $\underline{C}H_2Ph$), 45.11 (t, $\underline{C}H_2Ph$), 50.7 (d, C3a), 51.3 (d, C3a), 58.48 (d, C6a), 58.53 (d, C6a), 66.9 (d, CHOH), 67.1 (d, CHOH), 128.0 (d, CH_{Ar}), 128.1 (d, CH_{Ar}), 128.2 (d, CH_{Ar}), 128.27 (d, CH_{Ar}), 129.0 (d, CH_{Ar}), 129.1 (d, CH_{Ar}), 135.9 (s, C_{Ar}), 136.13 (s, C_{Ar}), 175.84 (s, C2), 176.58 (s, C2), 217.38 (s, C4), 218.28 (s, C4).

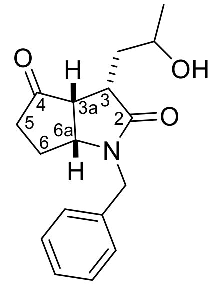

(3*S**,3a*R**,6a*R**)

Minor diastereomers **18B**: ^{1}H NMR (400 MHz, $CDCl_3$, detectable signals): δ 1.21 (d, *J* = 6.2 Hz, 3H, CH_3), 1.24 (d, *J* = 6.1 Hz, 3H, CH_3), 1.44-1.54 (m, 2H, $C\underline{H}_2CHOH$), 2.33-2.53 (m, 2H, H3), 2.89-2.97 (m, 1H, H3a), 3.54-3.62 (m, 1H, $C\underline{H}OH$), 3.89-3.99 (m, 1H, $C\underline{H}OH$), 4.85 (d, *J* = 14.7 Hz, 1H, $C\underline{H}_2Ph$), 4.95 (d, *J* = 15.0 Hz, 1H, $C\underline{H}_2Ph$); ^{13}C NMR (101 MHz, $CDCl_3$): δ 21.8 (q, CH_3), 22.3 (q, CH_3), 26.1 (t, C6), 27.8 (t, C6), 29.2 (t, C5), 29.5 (t, C5), 36.9 (d, C3), 39.0 (d, C3), 40.2 (t, 2C, $\underline{C}H_2CHOH$), 40.77 (d, C3a), 41.1 (d, C3a), 44.7 (t, $\underline{C}H_2Ph$), 44.8 (t, $\underline{C}H_2Ph$), 60.2 (d, C6a), 61.5 (d, C6a), 64.8 (d, CHOH), 66.4 (d, CHOH), 127.7 (d, CH_{Ar}), 127.8 (d, CH_{Ar}),

128.32 (d, CH_{Ar}), 128.4 (d, CH_{Ar}), 128.8 (d, CH_{Ar}), 128.9 (d, CH_{Ar}), 136.0 (s, C_{Ar}), 136.10 (s, C_{Ar}), 175.80 (s, C2), 176.56 (s, C2), 217.39 (s, C4), 218.27 (s, C4).

Preparation of the hydrochloride adduct of S13 and 13n

Compound **S13** or **13n** (0.5 mmol) was dissolved in a round-bottomed flask in diethyl ether (5 mL). The solution was cooled to 0 °C and HCl solution (0.5 mL, 1.0 mmol, 2 M in Et_2O) was added dropwise. The mixture was stirred for 10 min when a white precipitate formed. The solvent was removed carefully under reduced pressure to give a quantitative yield of the HCl salt as an off-white solid. The solid salt **S13·HCl** was crystallized from MTBE with a few drops of $CHCl_3$ and salt **13nA·HCl** was crystallized from hexane with a few drops of DCM to give crystals suitable for X-ray analysis. After X-ray analysis, the crystal of **S13·HCl** was redissolved in $CDCl_3$ and the ^{1}H NMR spectrum was recorded. The comparison of the ^{1}H NMR spectrum of the crystal and the ^{1}H NMR spectrum of the diastereomeric mixture of the salt **S13·HCl** showed that the measured crystal was a crystal of the major diastereomer (see section ^{1}H and ^{13}C NMR spectra, p. S202).

1-(((2*R*,4*S*)- and (2*R*,4*S*)-1-(Allyl((*S*)-1-(naphthalen-2-yl)ethyl)amino)-4-hydroxy-1-oxopentan-2-yl)oxy)-2,2,6,6-tetramethylpiperidin-1-ium chloride (S13·HCl):

IR (film); ν [cm^{-1}]: 3550-3250 (br), 2945 (w), 2926 (w), 2500-2000 (br), 1631 (m), 1507 (w), 1451 (w), 1418 (w), 1378 (w), 1362 (w), 1321 (w), 1242 (w), 1182 (w), 1128 (w), 1091 (w), 958 (m), 908 (w), 727 (s), 644 (w); MS (+ESI) m/z, (%): 467 (100, [M–HCl+H^+]); HRMS (+ESI) m/z [$C_{29}H_{43}N_2O_3^+$]: calcd. 467.3268; found 467.3269.

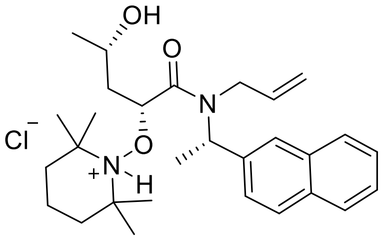

(2*R*,4*S*)

Major diastereomer: ^{1}H NMR (400 MHz, CDCl$_3$): δ 1.21/1.25 (d, *J* = 6.2 Hz, 3H, C$\underline{H}_3$CHOH), 1.46 (s, 6H, NCCH$_3$), 1.47 (s, 6H, NCCH$_3$), 1.58-1.77 (m, 6H, piperidine-H3, H4, H5), 1.64/1.68 (d, *J* = 7.0 Hz, 3H, ArCHC$\underline{H}_3$), 2.23-2.29 (m, 1H, C$\underline{H}_2$CHOTMP), 2.30-2.58 (m, 2H, OH, C$\underline{H}_2$CHOTMP), 3.66/3.81 (dd, *J* = 15.1, 6.7 Hz/*J* = 15.8, 3.2 Hz, 1H, C$\underline{H}_2$CH=), 4.16-4.22/4.25-4.30 (m, 1H, C$\underline{H}$OH), 4.31-4.40 (m, 1H, C$\underline{H}_2$CH=), 4.92-5.16 (m, 2H, CH=C$\underline{H}_2$), 5.24-5.51 (m, 1H, C$\underline{H}$=CH$_2$), 6.03/6.12 (q, *J* = 7.0/*J* = 7.2 Hz, 1H, ArC$\underline{H}$CH$_3$), 6.15-6.20/6.25-6.33 (m, 1H, C$\underline{H}$OTMP), 7.32-7.54 (m, 3H, Ar$\underline{H}$), 7.76-7.87 (m, 4H, Ar$\underline{H}$), 13.54/13.63 (br. s, 1H, NH); ^{13}C NMR (101 MHz, CDCl$_3$): δ 15.86/15.91 (t, piperidine-C4), 15.89/17.1 (q, ArCH$\underline{C}$H$_3$), 21.26 (q, NC$\underline{C}$H$_3$), 21.29 (q, NC$\underline{C}$H$_3$), 22.9/24.2 (q, $\underline{C}$H$_3$CHOH), 28.5 (q, NC$\underline{C}$H$_3$), 29.0 (q, NC$\underline{C}$H$_3$), 37.4 (t, piperidine-C3, C5), 42.1 (t, $\underline{C}$H$_2$CHOTMP), 46.5/46.59 (t, $\underline{C}$H$_2$CH=), 52.8/53.2 (d, Ar$\underline{C}$HCH$_3$), 59.7 (s, CNO), 60.2 (s, CNO), 62.3/63.5 (d, CHOH), 79.1/81.2 (d, $\underline{C}$HOTMP), 117.8/118.4 (t, CH=$\underline{C}$H$_2$), 124.7/125.3 (d, CH$_{Ar}$), 126.27/126.32 (d, CH$_{Ar}$), 126.4/126.47 (d, CH$_{Ar}$), 126.52/126.87 (d, CH$_{Ar}$), 127.7/127.8 (d, CH$_{Ar}$), 128.14/128.2 (d, CH$_{Ar}$), 128.4/128.6 (d, CH$_{Ar}$), 132.9/133.0 (s, C$_{Ar}$), 133.27/133.31 (s, C$_{Ar}$), 134.0/134.4 (d, $\underline{C}$H=CH$_2$), 137.5 (s, C$_{Ar}$), 173.6/175.1 (s, C=O).

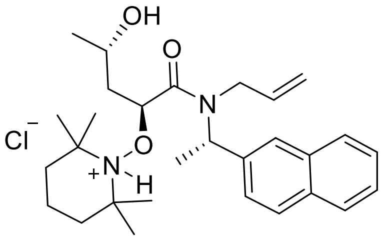

(2*S*,4*S*)

Minor diastereomer: ^{1}H NMR (400 MHz, CDCl$_3$): δ 1.00 (d, *J* = 6.3 Hz, 3H, C$\underline{H}_3$CHOH), 1.49 (s, 6H, NCCH$_3$), 1.51 (s, 3H, NCCH$_3$), 1.53 (s, 3H, NCCH$_3$), 1.58-1.77 (m, 7H, OH, piperidine-H3, H4, H5), 1.79/1.94 (d, *J* = 6.8 Hz, 3H, ArCHC$\underline{H}_3$), 1.84-2.00 (m, 2H, C$\underline{H}_2$CHOTMP), 2.30-2.58 (m, 1H, OH), 3.93-4.00 (m, 1H, C$\underline{H}_2$CH=), 4.08-4.15 (m, 1H, C$\underline{H}$OH), 4.62-4.71 (m, 1H,

$C\underline{H}_2CH=$), 4.92-5.16 (m, 2H, $CH=C\underline{H}_2$), 5.24-5.51 (m, 1H, $C\underline{H}=CH_2$), 5.66-5.85 (m, 1H, $ArC\underline{H}CH_3$), 6.60-6.65 (m, 1H, C$\underline{H}$OTMP), 7.32-7.54 (m, 3H, Ar$\underline{H}$), 7.76-7.87 (m, 4H, Ar$\underline{H}$), 13.77 (br. s, 1H, NH); ^{13}C NMR (101 MHz, $CDCl_3$): δ 16.0 (t, piperidine-C4), 20.5 (q, $ArCH\underline{C}H_3$), 21.1 (q, 2C, $NC\underline{C}H_3$), 22.6 (q, $\underline{C}H_3CHOH$), 29.2 (q, $NC\underline{C}H_3$), 29.3 (q, $NC\underline{C}H_3$), 37.9 (t, piperidine-C3, C5), 43.6 (t, $\underline{C}H_2$CHOTMP), 46.62 (t, $\underline{C}H_2CH=$), 55.7 (d, $Ar\underline{C}HCH_3$), 59.7 (s, CNO), 60.2 (s, CNO), 64.0 (d, CHOH), 81.3 (d, $\underline{C}$HOTMP), 119.8 (t, $CH=\underline{C}H_2$), 125.1 (d, CH_{Ar}), 126.2 (d, CH_{Ar}), 126.7 (d, CH_{Ar}), 126.90 (d, CH_{Ar}), 127.9 (d, CH_{Ar}), 128.07 (d, CH_{Ar}), 128.8 (d, CH_{Ar}), 132.7 (s, C_{Ar}), 133.4 (s, C_{Ar}), 136.9 (d, $\underline{C}H=CH_2$), 138.3 (s, C_{Ar}), 175.6 (s, C=O).

2. X-Ray crystallography

The crystallographic data for **12k**, **13j**, **13nA·HCl**, and **S13·HCl** were collected on a Bruker D8 VENTURE Kappa Duo diffractometer equipped with a PHOTON III detector, and an X-ray source IμS micro-focus sealed tube using CuKα (λ= 1.54178 Å) radiation at a temperature 120(2) K. The structures were solved by direct methods (XT)[12a] and refined by full matrix least squares based on F^2 (SHELXL2018).[12b] The hydrogen atoms on carbon were fixed into idealized positions (riding model) and assigned temperature factors either H_{iso}(H) = 1.2 U_{eq}(pivot atom) or H_{iso}(H) = 1.5 U_{eq}(pivot atom) for methyl groups. The hydrogen atoms of –N-H groups were found on the difference Fourier maps and refined under rigid body assumption with assigned temperature factors H_{iso}(H) = 1.2 U_{eq}(pivot atom). The absolute configuration for compounds **12k**, **13j**, and **S13·HCl** was confirmed by anomalous scattering of N and O and Cl atoms. The crystallographic data are summarized in Table S1.

Table S1: Crystallographic data, data collection, and structure refinement parameters for compounds **S13·HCl**, **12k**, **13j**, and **13nA·HCl**.

Compound	**S13·HCl**	**12k**	**13j**	**13nA·HCl**
CCDC entry	2040885	2040886	2040887	2040888
Formula	$C_{30}H_{44}Cl_4N_2O_3$	$C_{18}H_{25}NO_2$	$C_{29}H_{40}N_2O_3$	$C_{28}H_{43}ClN_2O_3$
M (g mol^{-1})	622.47	287.39	464.63	491.09
Crystal size (mm)	0.22 × 0.14 × 0.097	0.42 × 0.24 × 0.17	0.29 × 0.055 × 0.048	0.29 × 0.11 × 0.076
Crystal system	orthorhombic	monoclinic	monoclinic	monoclinic
Space group	$P2_12_12_1$	$P2_1$	$C2$	$P2_1/c$
a (Å)	8.4685(4)	10.9324(8)	21.9325(9)	11.4050(4)
b (Å)	12.8323(7)	5.7722(4)	6.0099(3)	21.9241(8)
c (Å)	30.4621(16)	13.1448(9)	22.1042(10)	11.7015(4)
α(°)	90	90	90	90
β (°)	90	93.122(2)	114.102(2)	113.5230(10)
γ (°)	90	90	90	90
V (Å^3)	3310.3(3)	828.26(10)	2659.6(2)	2682.75(16)
Z	4	2	4	4
D_{calc} *(mg m^{-3})*	1.249	1.152	1.160	1.216
μ(Mo Kα) (mm^{-1})	3.497	0.58	0.59	1.50
2θ(max) (°)	72.4	72.2	72.1	72.2
Number of reflections	34067	14442	14851	45024
Unique reflections / Rint[a]	6521/0.044	3177/0.021	5107/0.044	5275/0.034
$R1$ ($F^2 > 2\sigma(F)$)[b]	0.037	0.030	0.042	0.036
wR2[c]	0.094	0.076	0.114	0.097
GOF[d]	1.05	1.06	1.05	1.03
min/max Δρ(e Å^{-3})	–0.44/0.49	–0.12/0.23	–0.19/0.17	–0.23/0.34
Flack parameter	0.015(5)	0.06(5)	–0.1(2)	-

[a] $R_{int} = \Sigma|F_o^2 - F_o^2{}_{,mean}|/\Sigma F_0^2$, [b] $R(F)=\Sigma||F_o|-|F_c||/\Sigma|F_o$, [c] $wR(F^2)=[\Sigma(w(F_o^2-F_c^2)^2)/(\Sigma(F_o^2)^2)]^{1/2}$, weighting scheme: $w=[\sigma^2(Fo^2)+(w_1P)^2+w_2P)^2]^{-1}$, where $P=[\max(F_o^2,0)+2F_c^2]^{1/3}$, [d] $S=[\Sigma(w(F_o^2-F_c^2)^2)/N_{diffrs} - N_{params})]^{1/2}$.

3. References

1. L. J. Peterson, J. Luo, J. P. Wolfe, *Org. Lett.* **2017**, *19*, 2817-2820.
2. F. Xu, S. A. Shuler, D. A. Watson, *Angew. Chem. Int. Ed.* **2018**, *57*, 12081-12085.
3. S. Kerres, E. Plut, S. Malcherek, J. Rehbein, O. Reisera, *Adv. Synth. Catal.* **2019**, *361*, 1400-1407.
4. M. T. Flynn, R. Stott, V. L. Blair, P. C. Andrews, *Organometallics* **2016**, *35*, 2707-2714.
5. J. Balsells, A. Moyano, A. Riera, M. A. Pericas, *Org. Lett.* **1999**, *1*, 1981-1984.
6. D. Just, D. Hernandez-Guerra, S. Kritsch, R. Pohl, I. Císařová, P. G. Jones, R. Mackman, G. Bahador, U. Jahn, *Eur. J. Org. Chem.* **2018**, 5213-5221.
7. S. Krompiec, M. Pigulla, N. Kuznik, M. Krompiec, B. Marciniec, D. Chadyniak, J. Kasperczyk, *J. Mol. Catal. A: Chem.* **2005**, *225*, 91-101.
8. N. Ohmura, A. Nakamura, A. Hamasaki, M. Tokunaga, *Eur. J. Org. Chem.* **2008**, 5042-5045.
9. R. Tamura, L. S. Hegedus, *J. Am. Chem. Soc.* **1982**, *104*, 3727-3729.
10. A. L. Bartuschat, K. Wicht, M. R. Heinrich, *Angew. Chem. Int. Ed.* **2015**, *54*, 10294-10298.
11. M. K. Klychnikov, R. Pohl, I. Císařová, U. Jahn, *Eur. J. Org. Chem.* **2020**, 2854–2866
12. a) SHELXT: G. M. Sheldrick, *Acta Cryst*. **2015**, *A71*, 3-8; b) SHELXT: G. M. Sheldrick, *Acta Cryst*. **2015**, *C71*, 3-8.

4. ^{1}H and ^{13}C NMR spectra

(*S*)-*N*-Allyl-*N*-(1-(naphthalen-2-yl)ethyl)amine (S10)

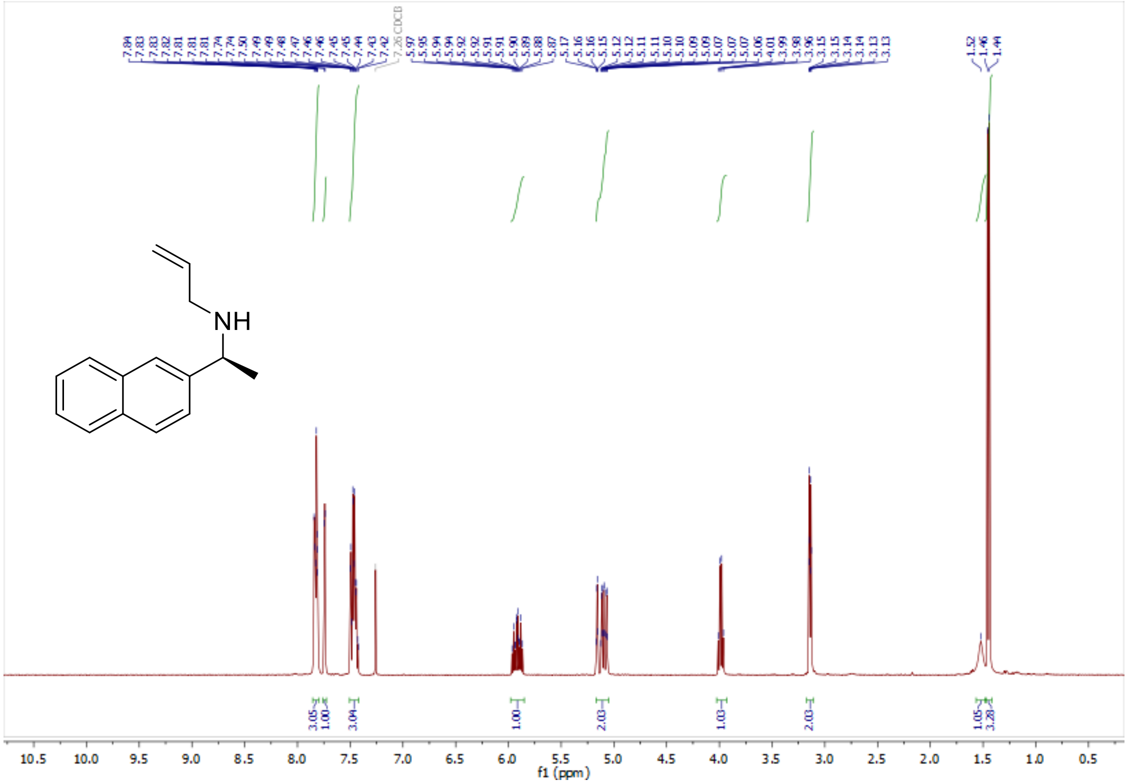

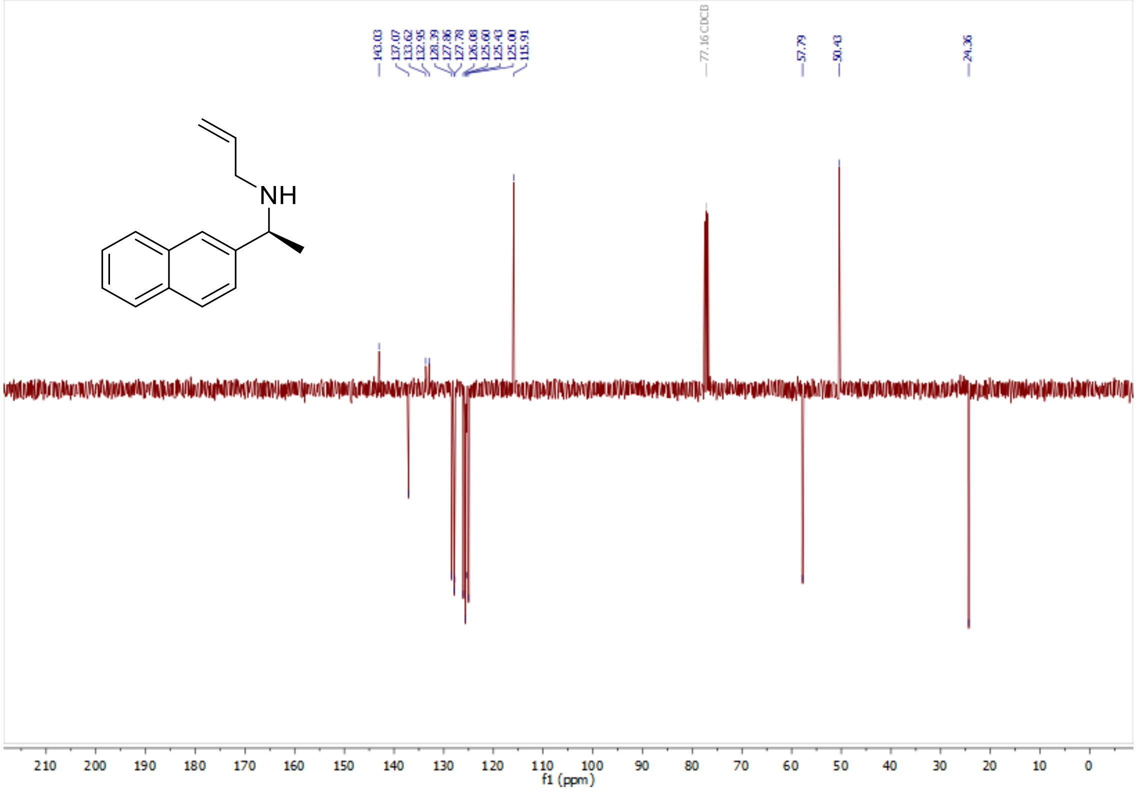

N-Benzyl-N-(cyclopent-1-en-1-ylmethyl)amine (S11)

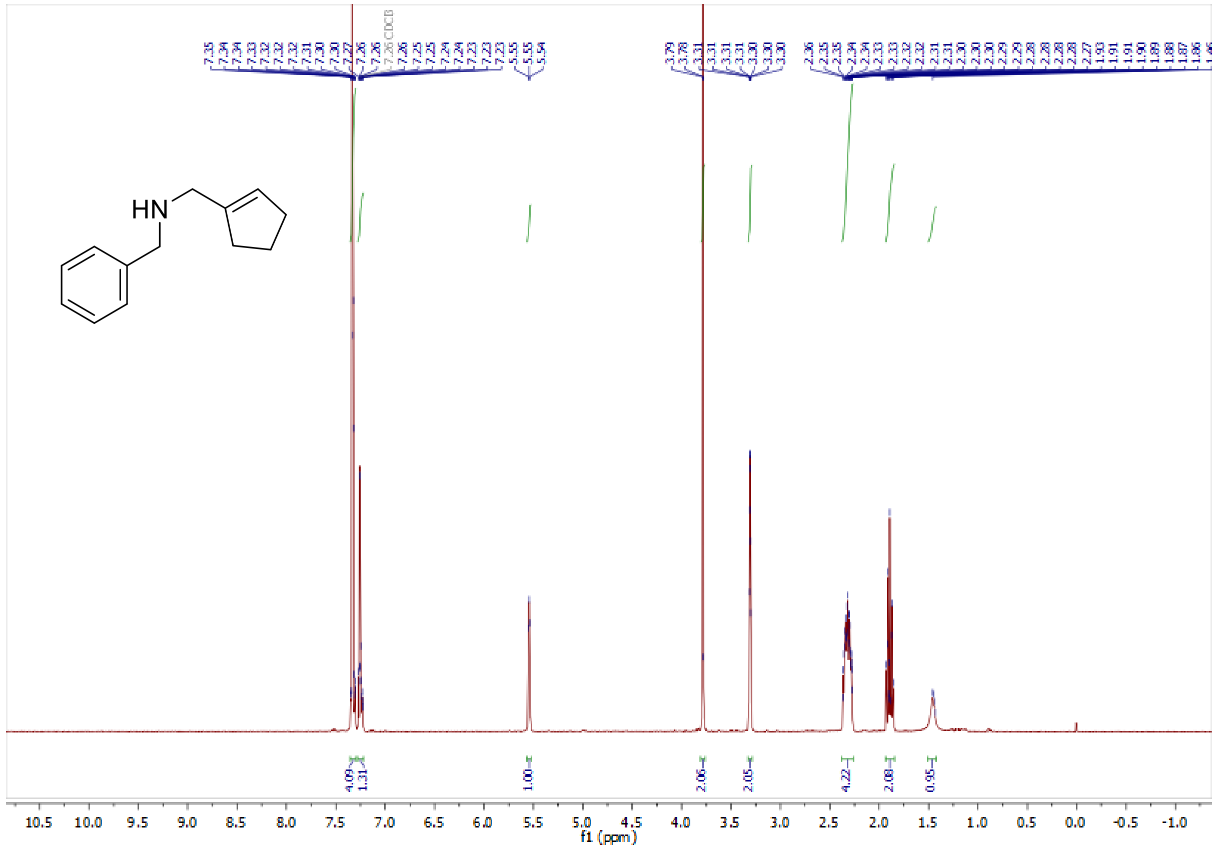

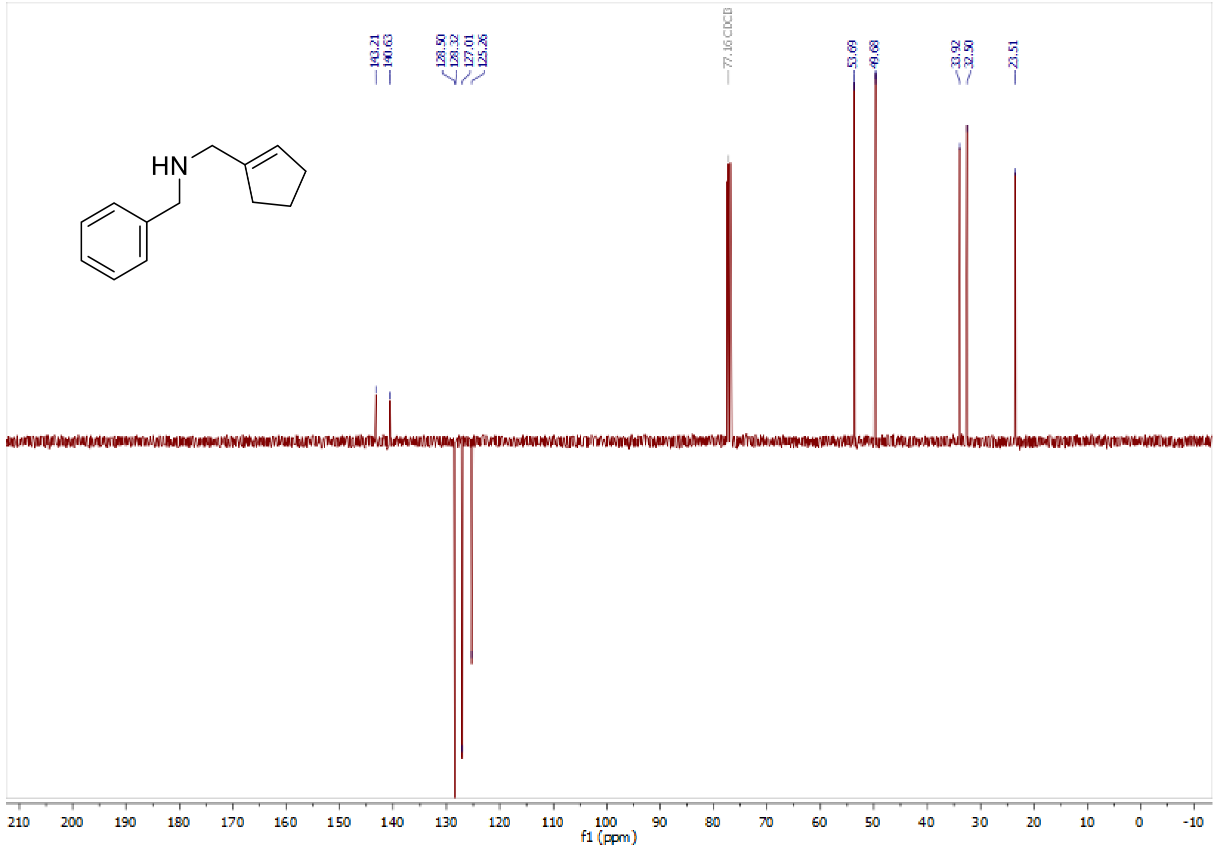

(*S*)-3-Methyl-*N*-(1-phenylethyl)but-2-en-1-amine (S12)

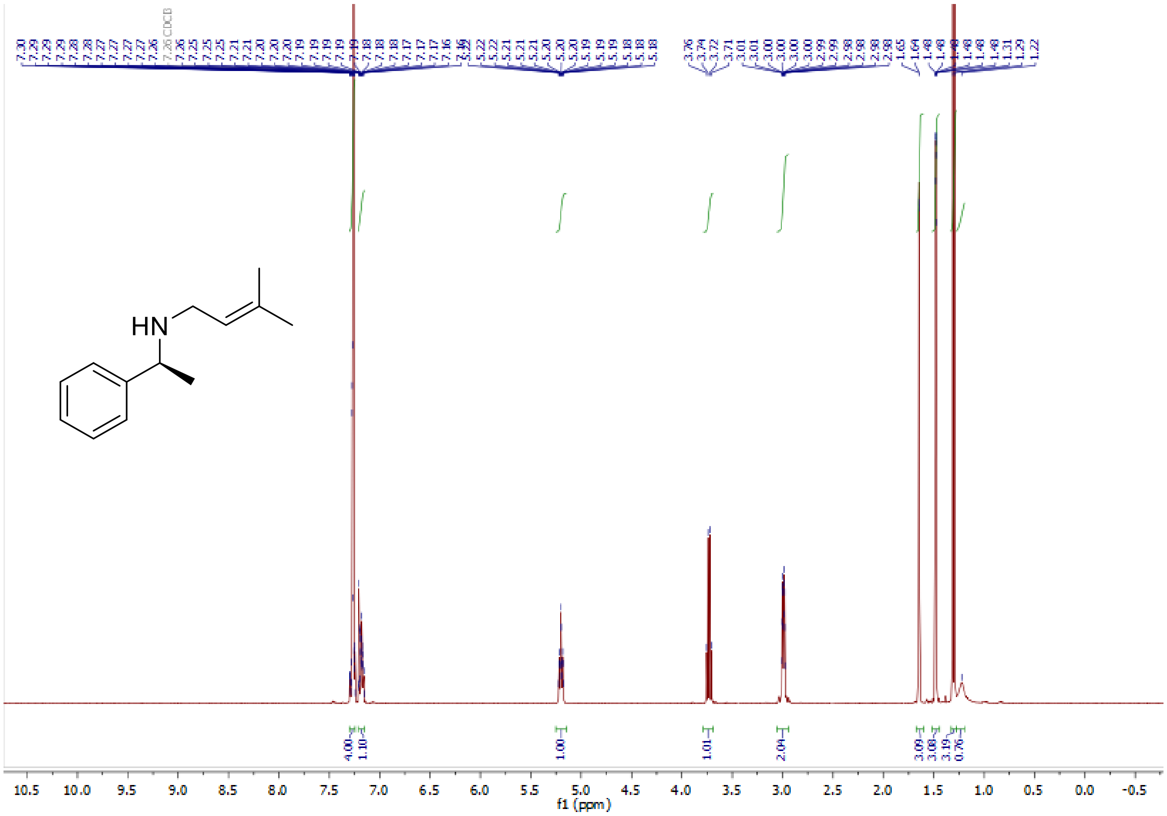

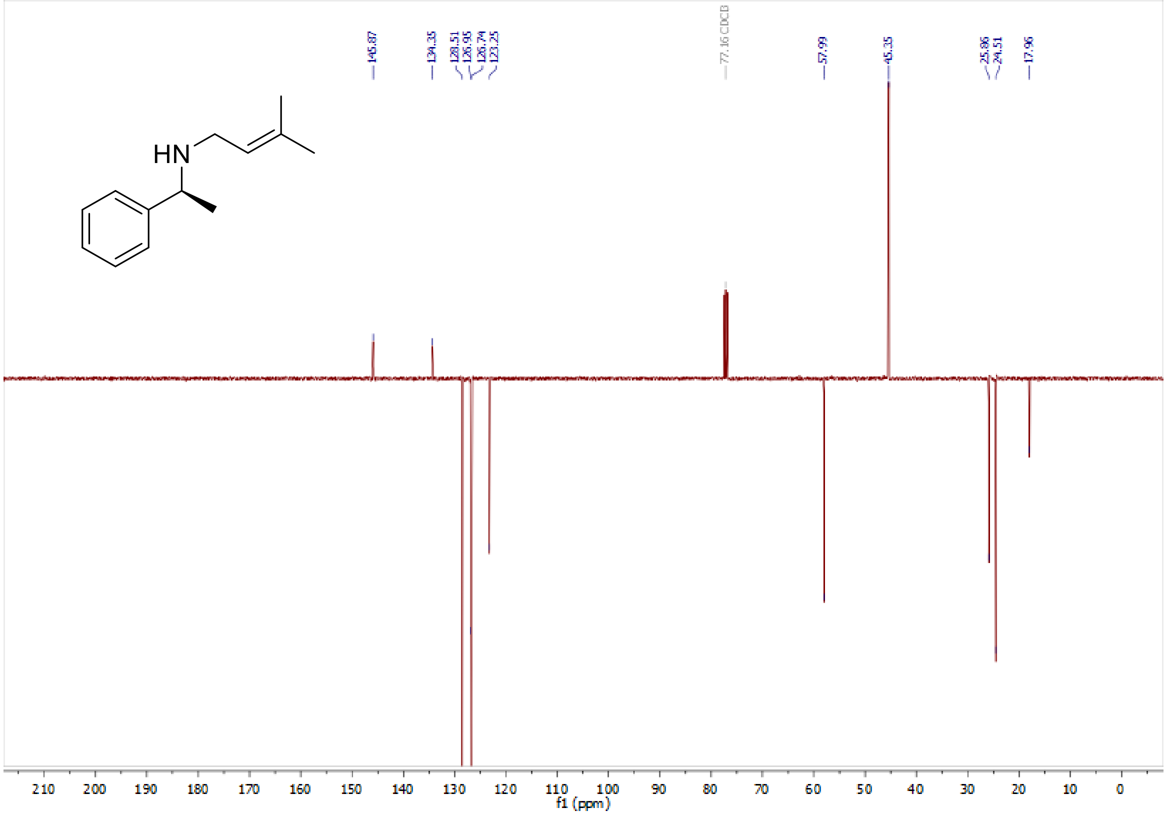

_N_-Benzyl-_N_-(2-methylallyl)acetamide (11c)

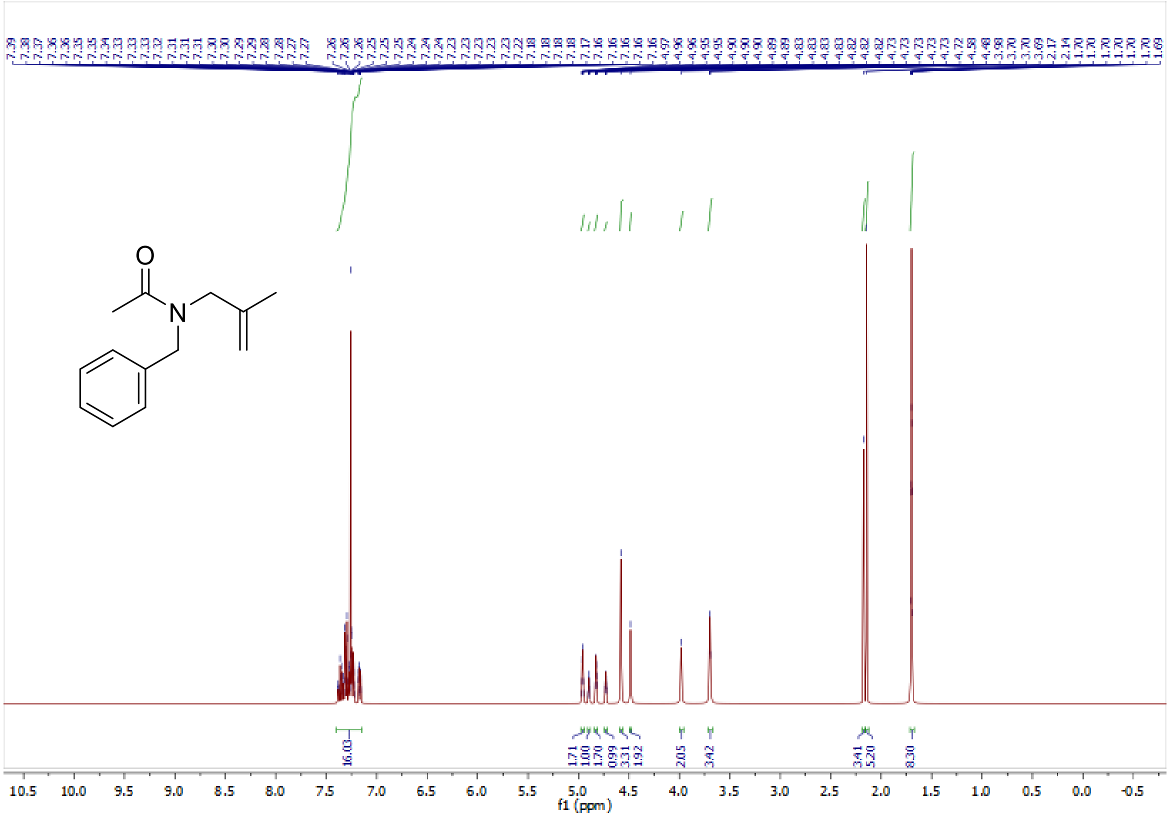

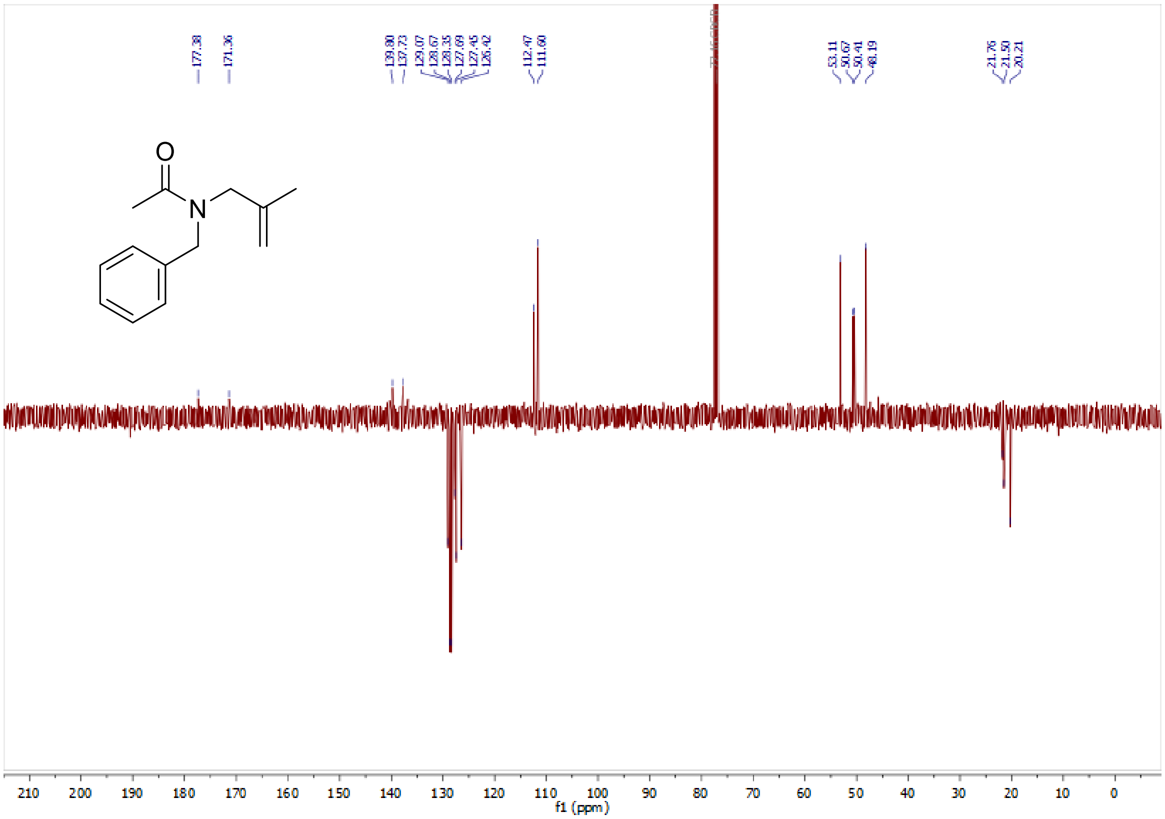

N-Benzyl-*N*-(3-methylbut-2-en-1-yl)acetamide (11d)

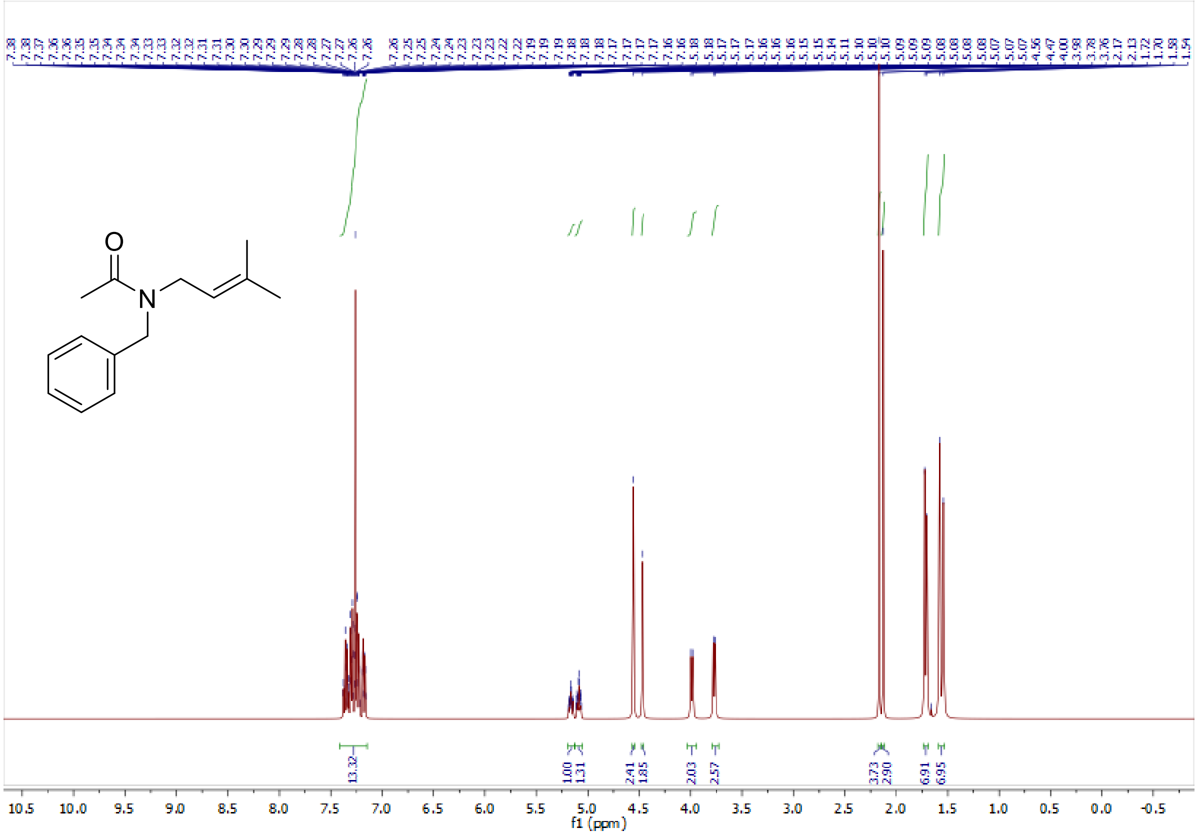

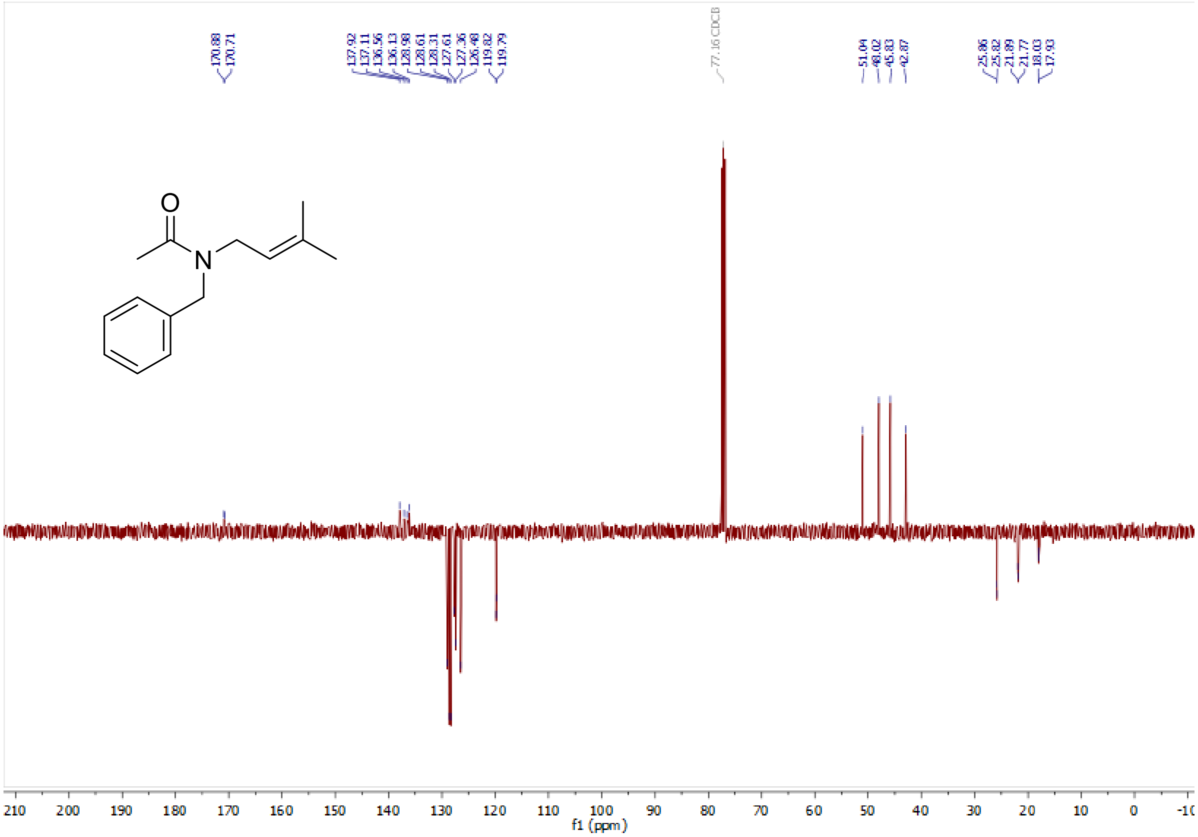

(*S*)-*N*-Allyl-*N*-(1-phenylethyl)acetamide (11e)

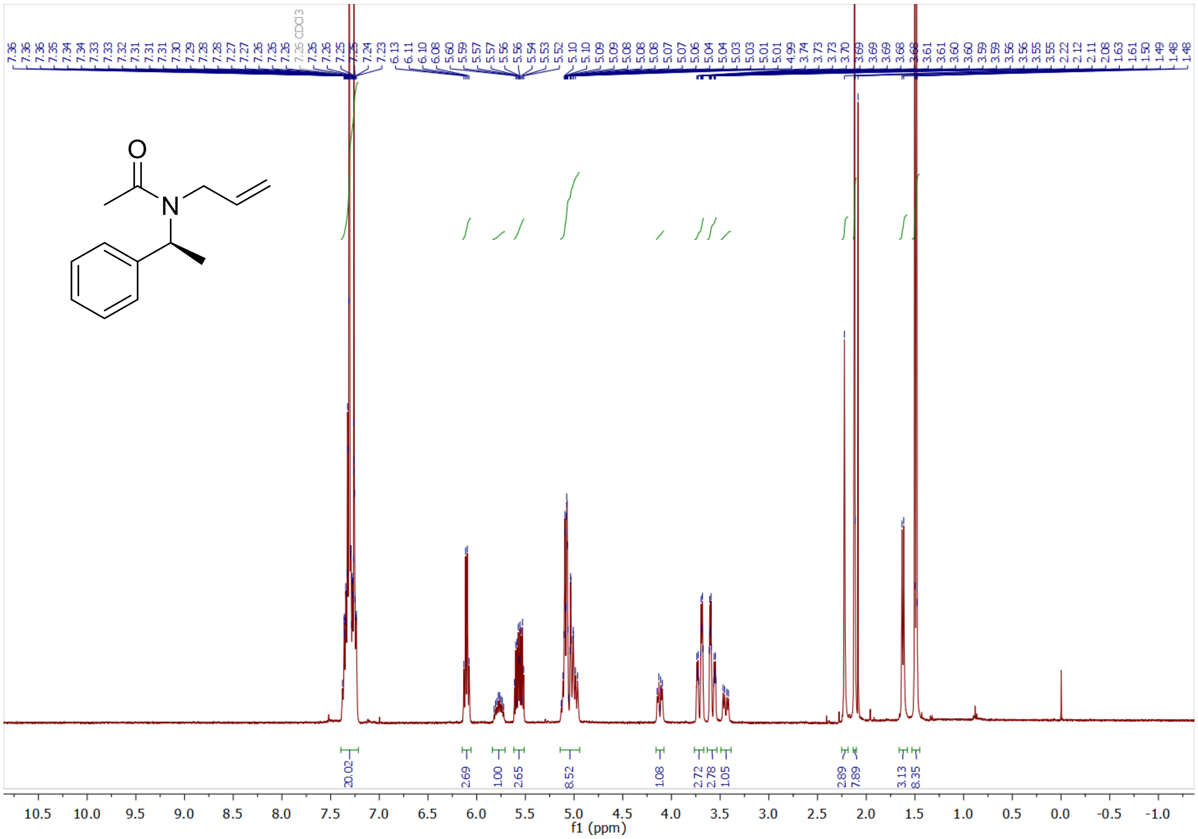

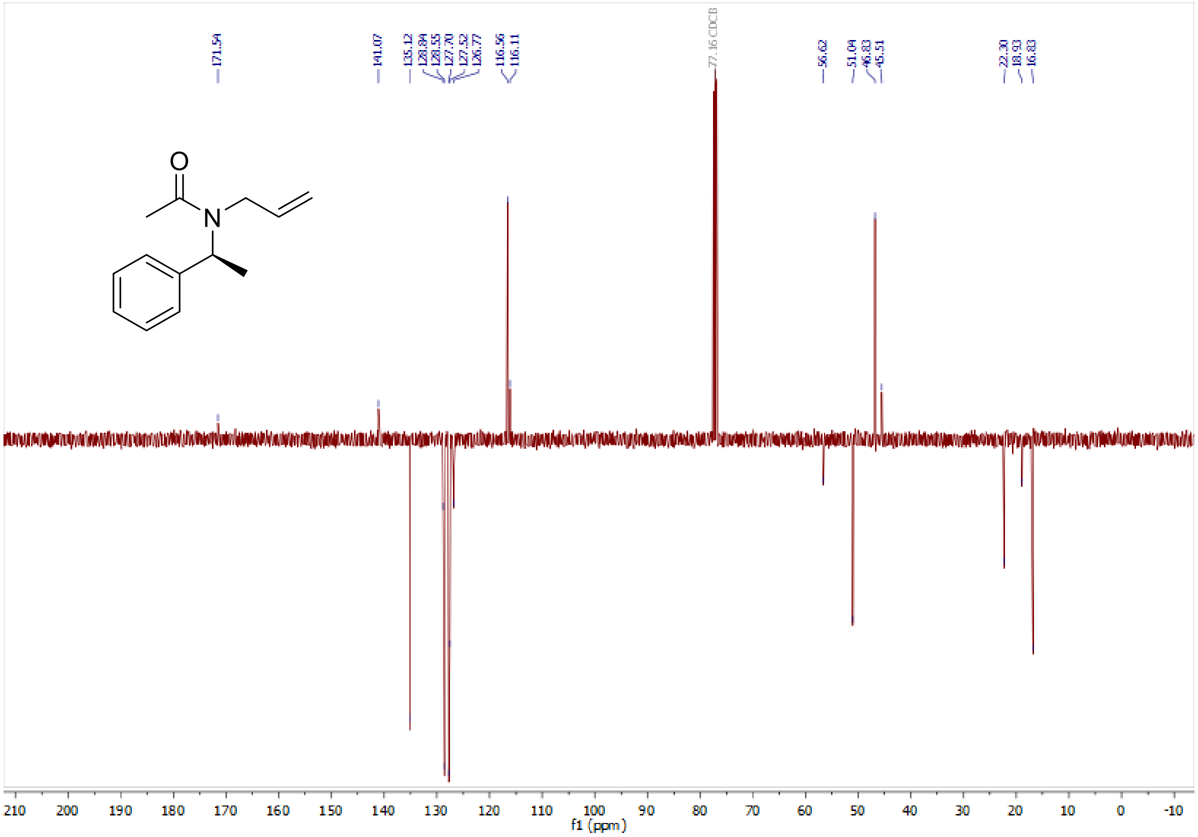

(*S*)-*N*-Allyl-*N*-(1-(naphthalen-2-yl)ethyl)acetamide (11f)

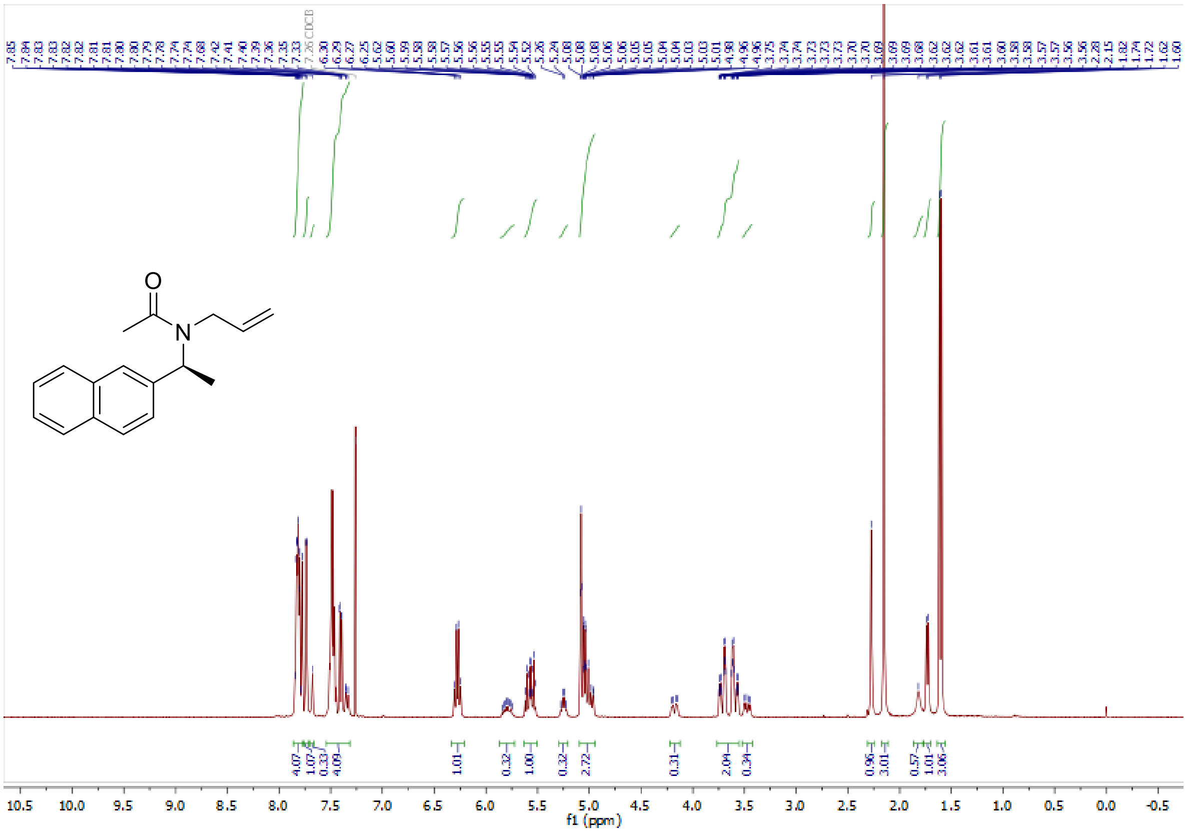

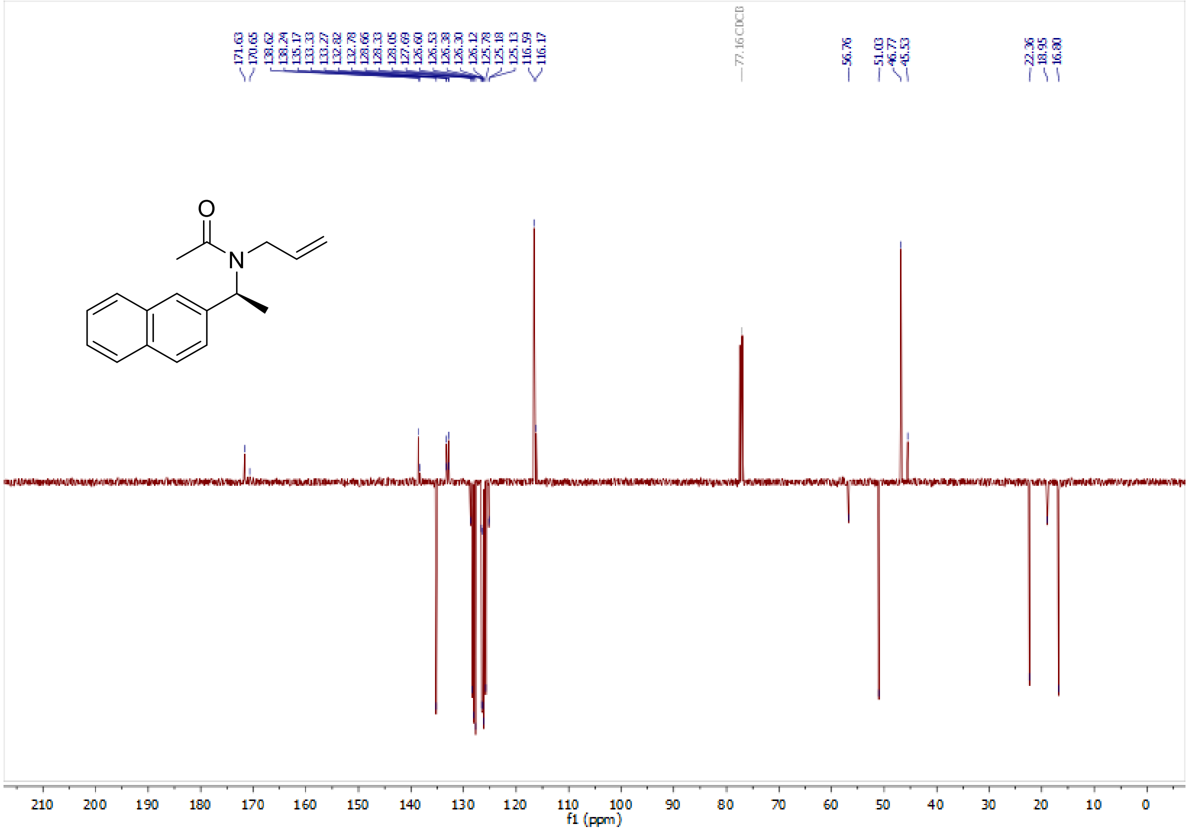

(*S*)-*N*-(3-Methylbut-2-en-1-yl)-*N*-(1-phenylethyl)acetamide (11g)

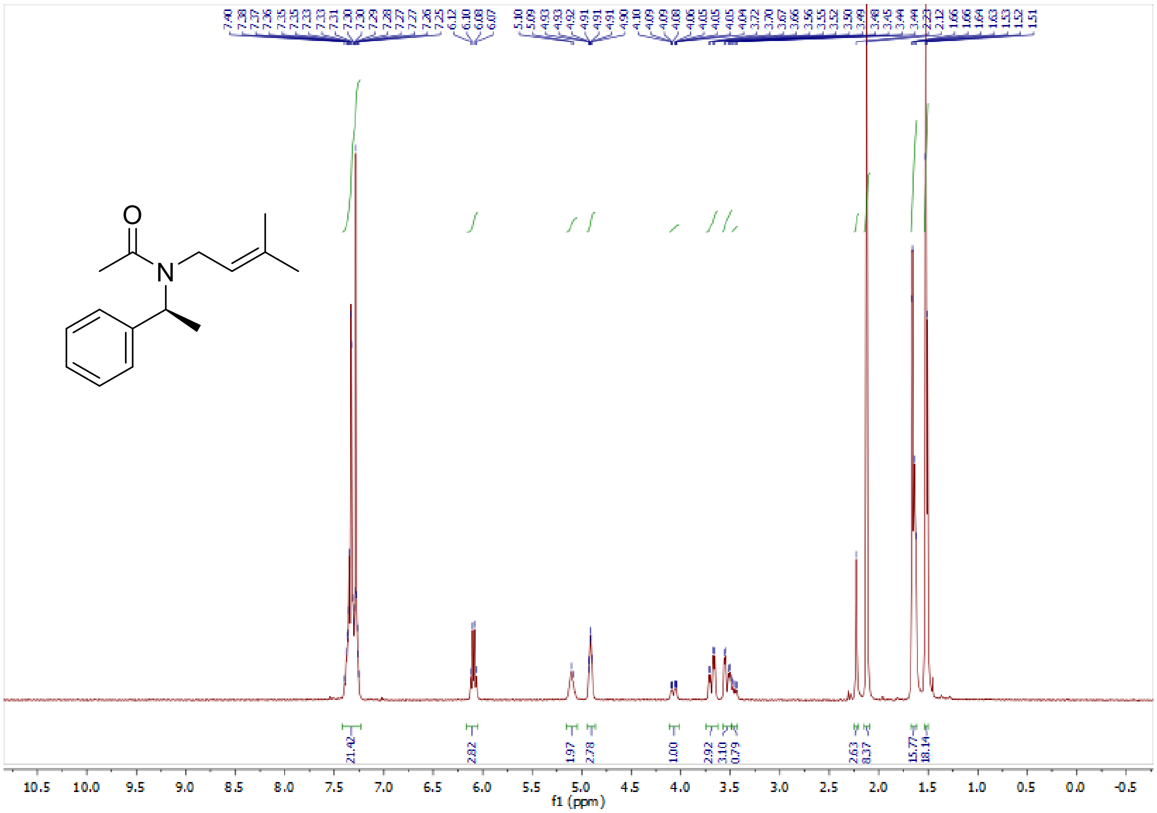

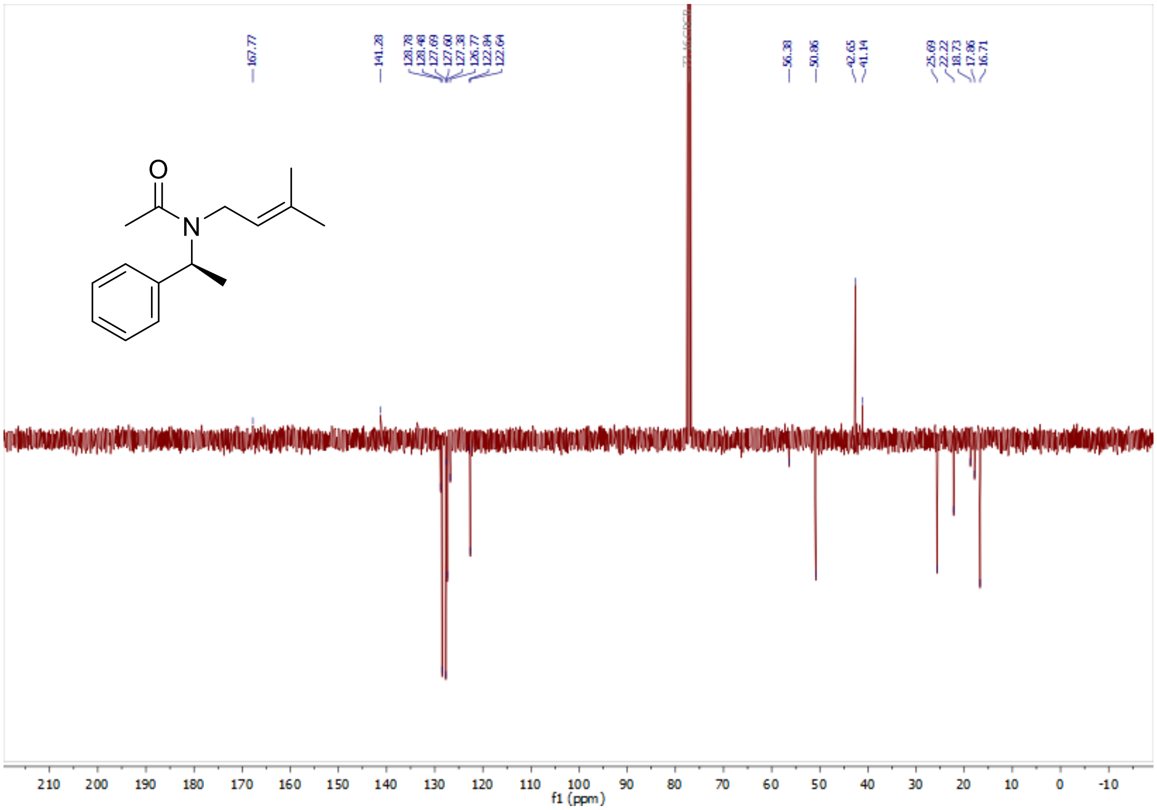

(*S*)-*N*-(2-Methylallyl)-*N*-(1-phenylethyl)acetamide (11h)

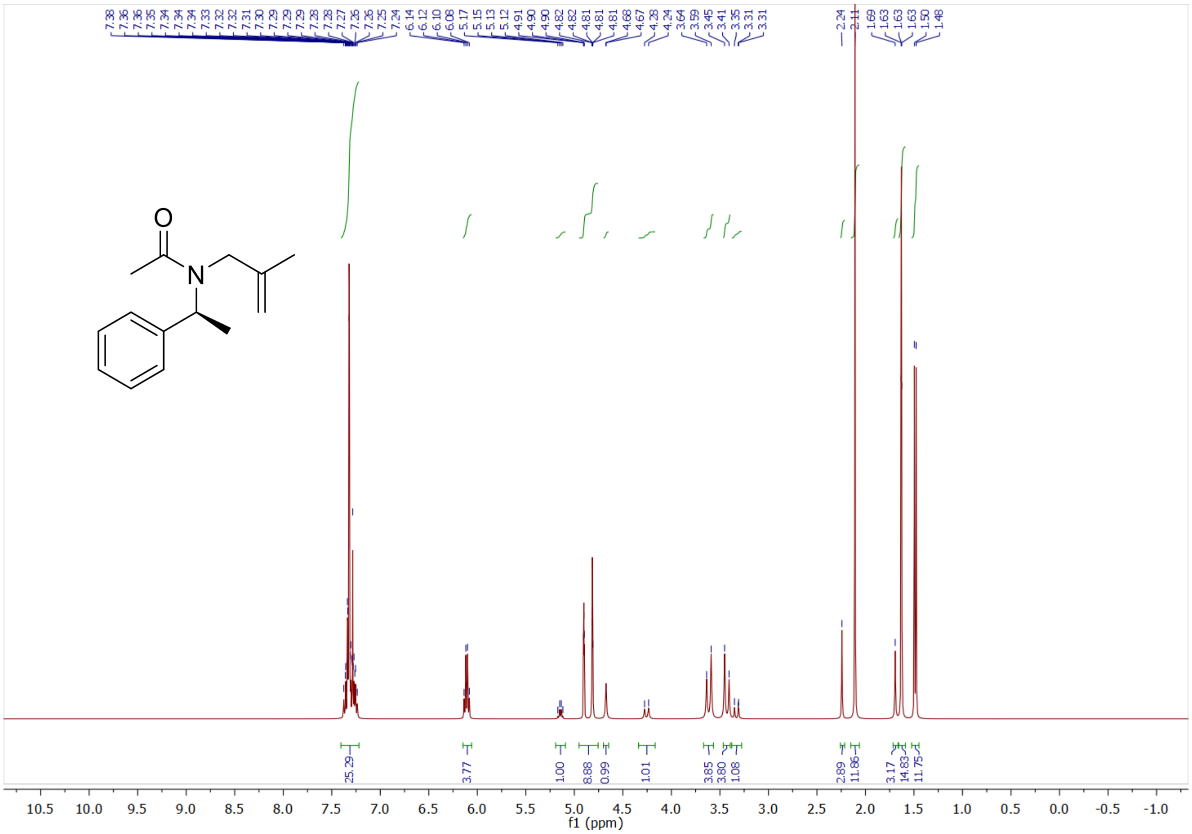

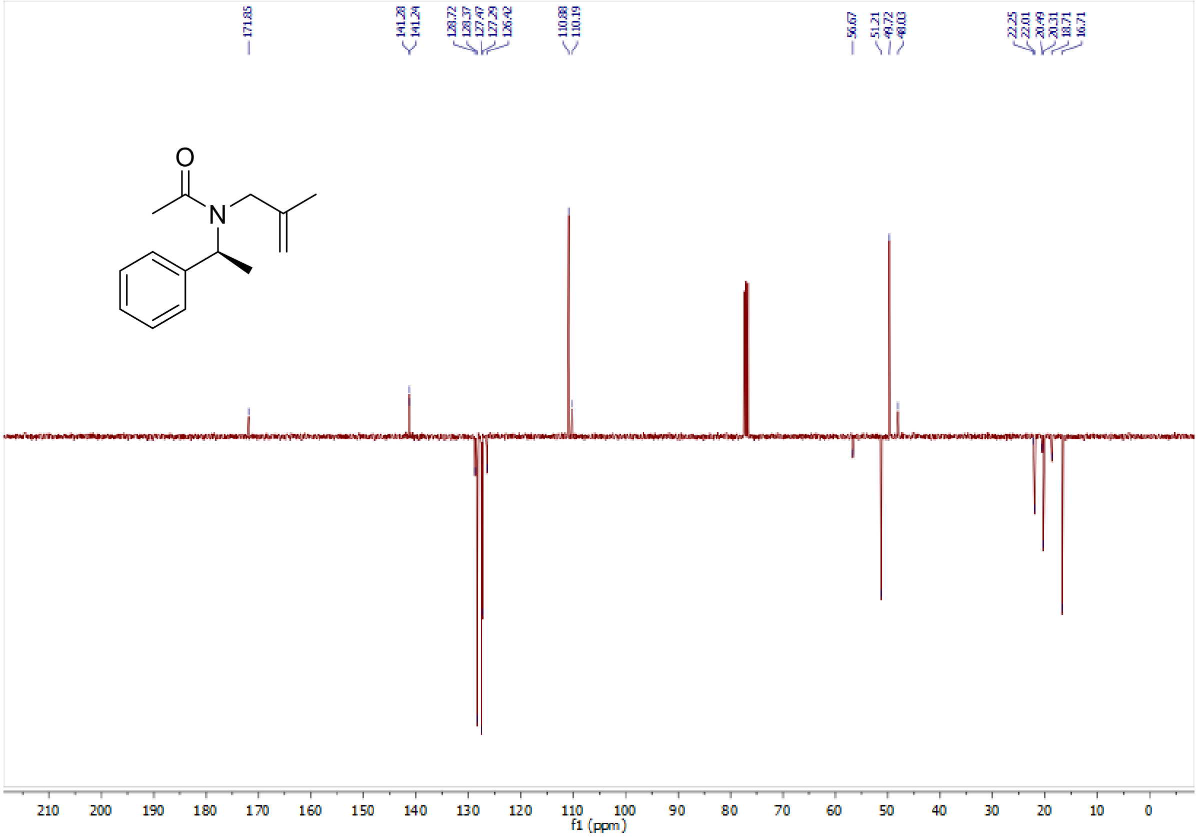

(*S*)-*N*-(Cyclopent-1-en-1-ylmethyl)-*N*-(1-phenylethyl)acetamide (11i)

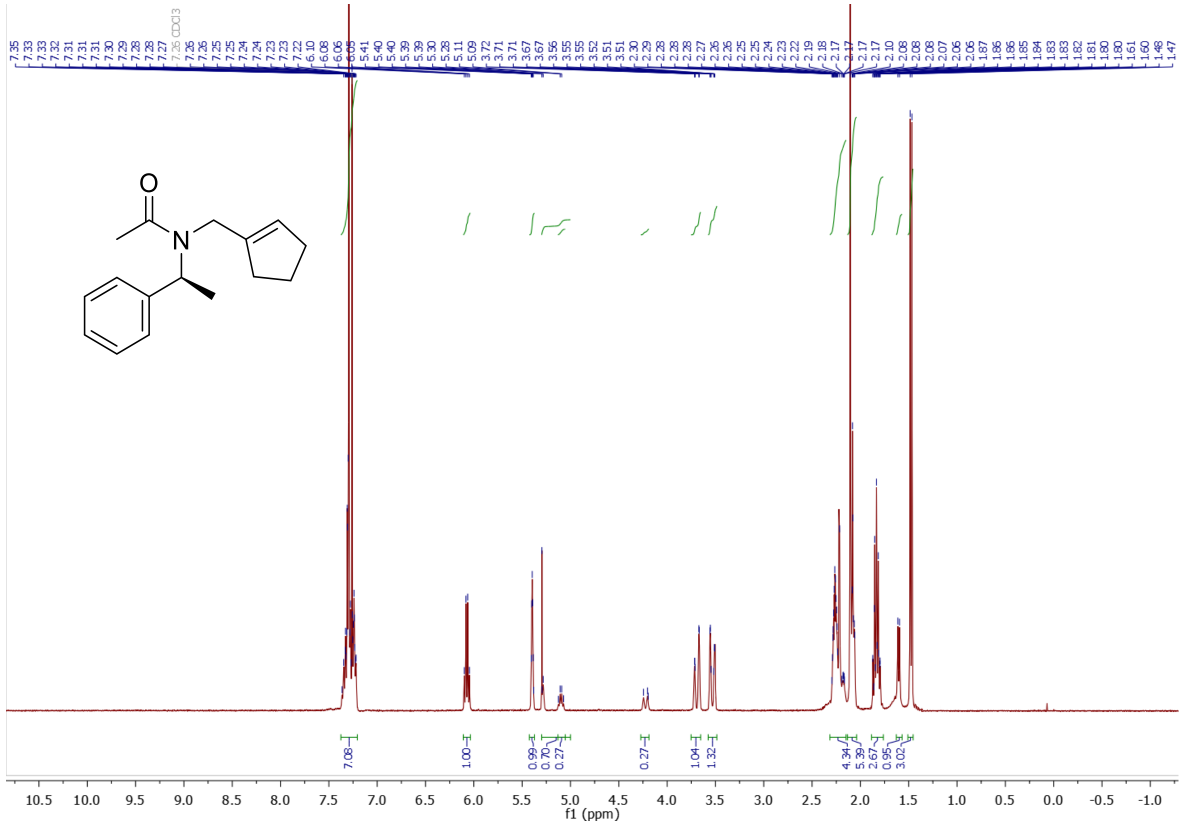

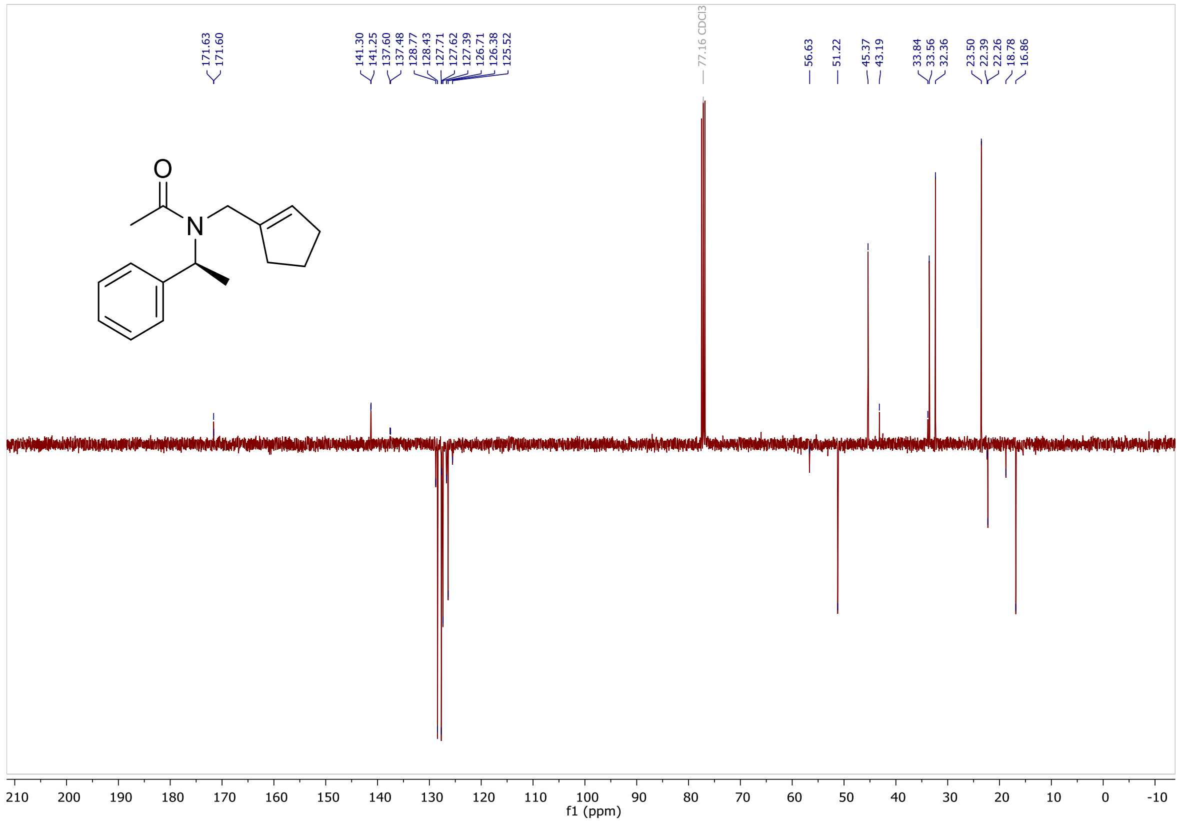

_N_-Benzyl-_N_-(cyclopent-1-en-1-ylmethyl)acetamide (11j)

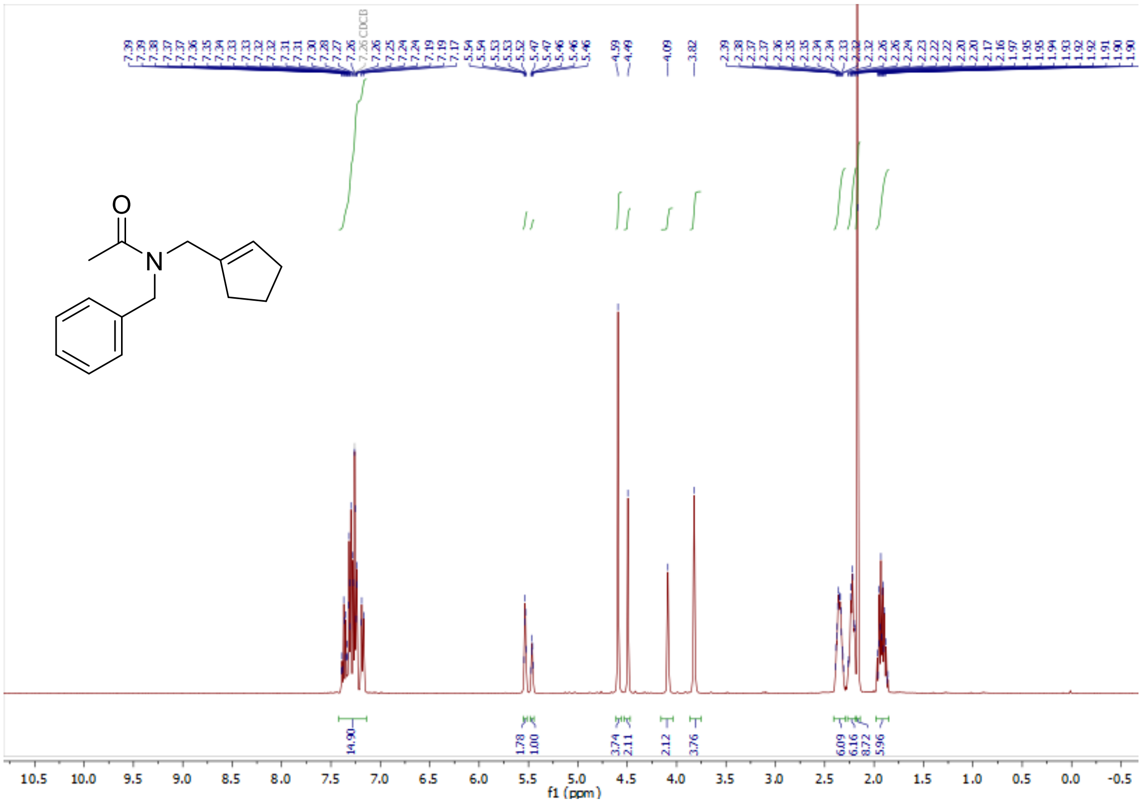

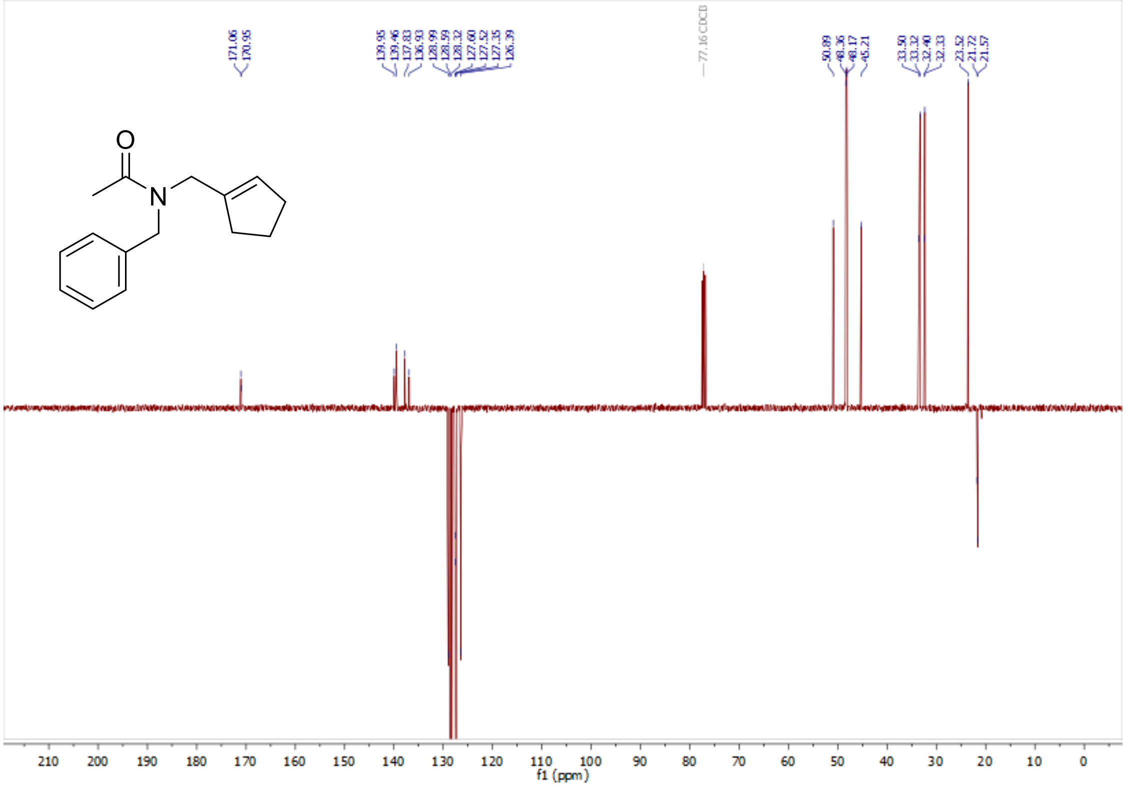

N-Benzyl-*N*-(cyclohex-1-en-1-ylmethyl)acetamide (11k)

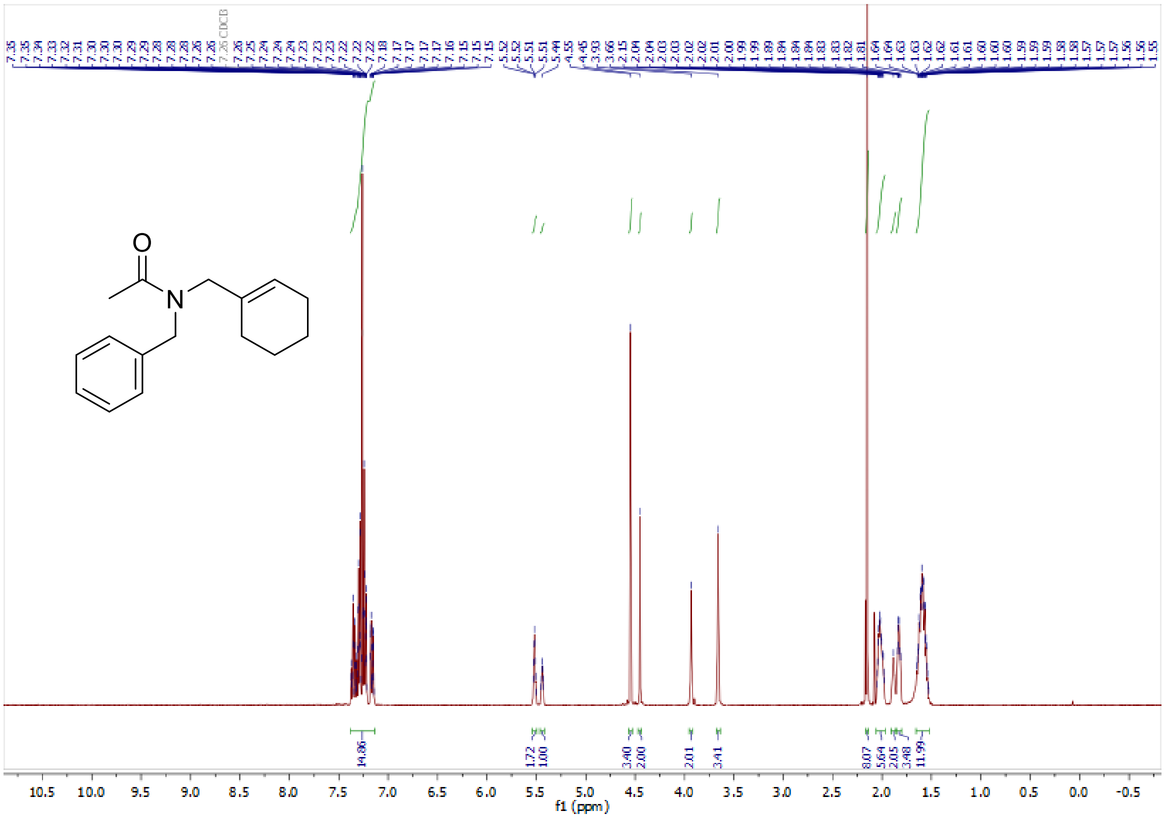

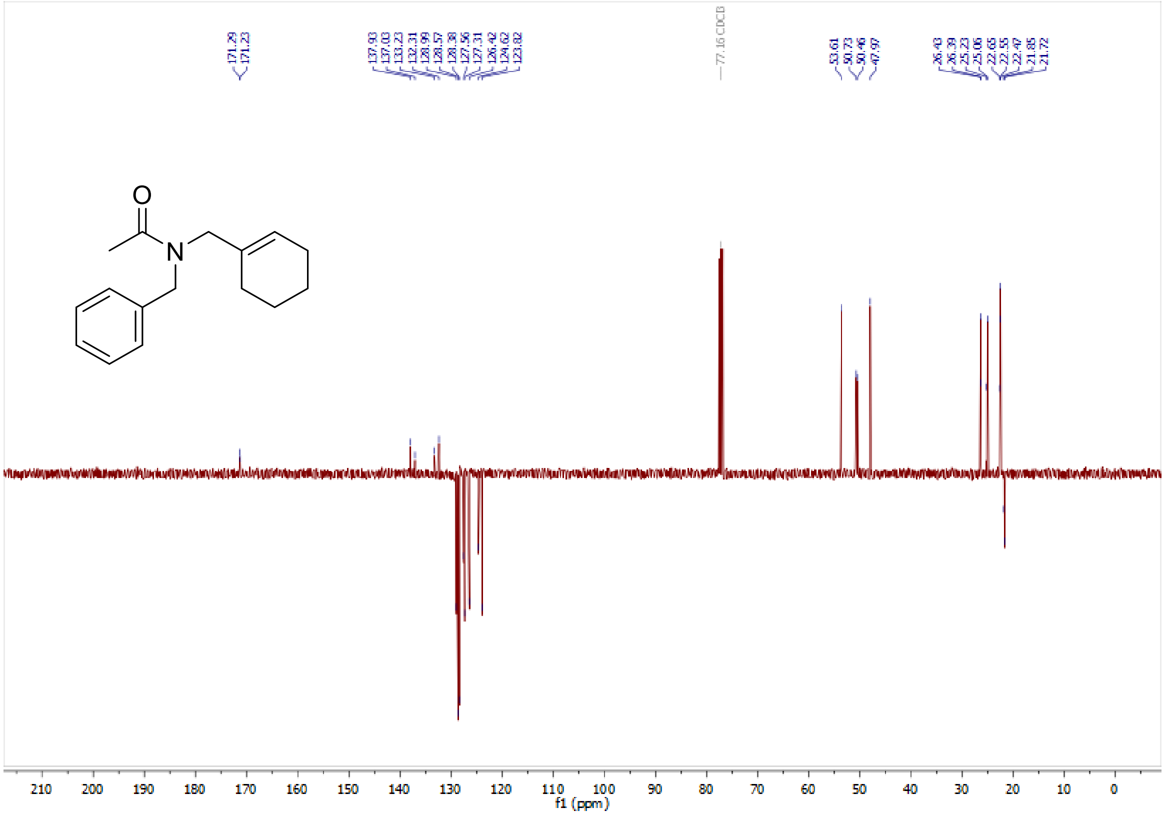

N-Benzyl-*N*-(cyclopent-2-en-1-yl)acetamide (11l)

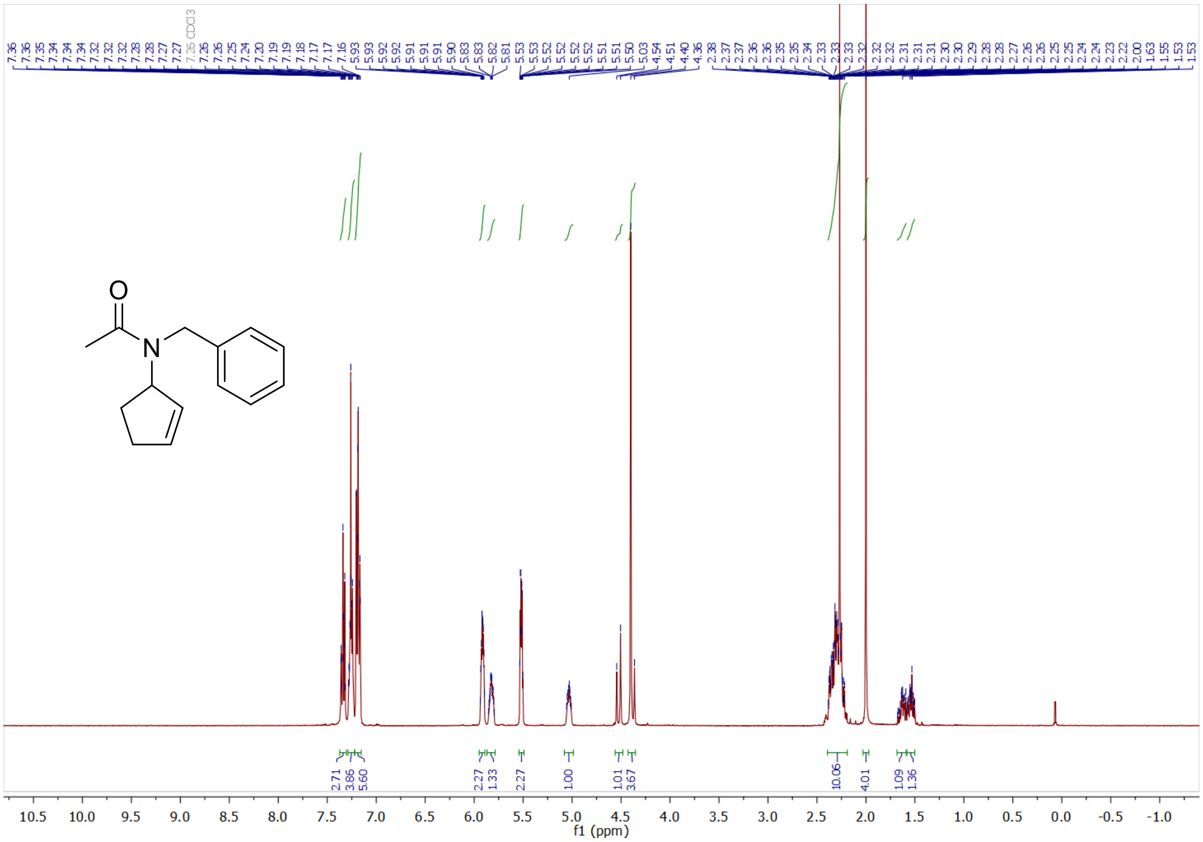

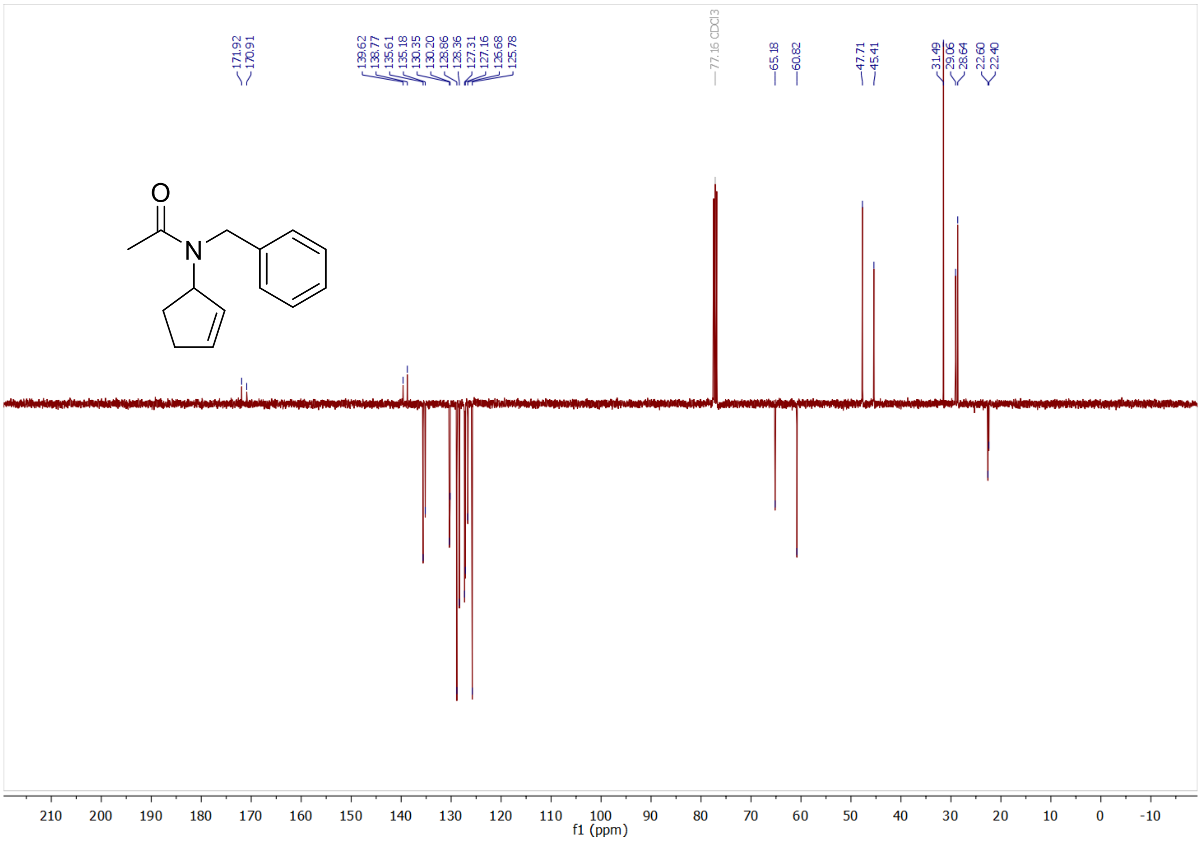

(*R*)-*N*-Benzyl-*N*-(cyclohex-2-en-1-yl)acetamide (11m)

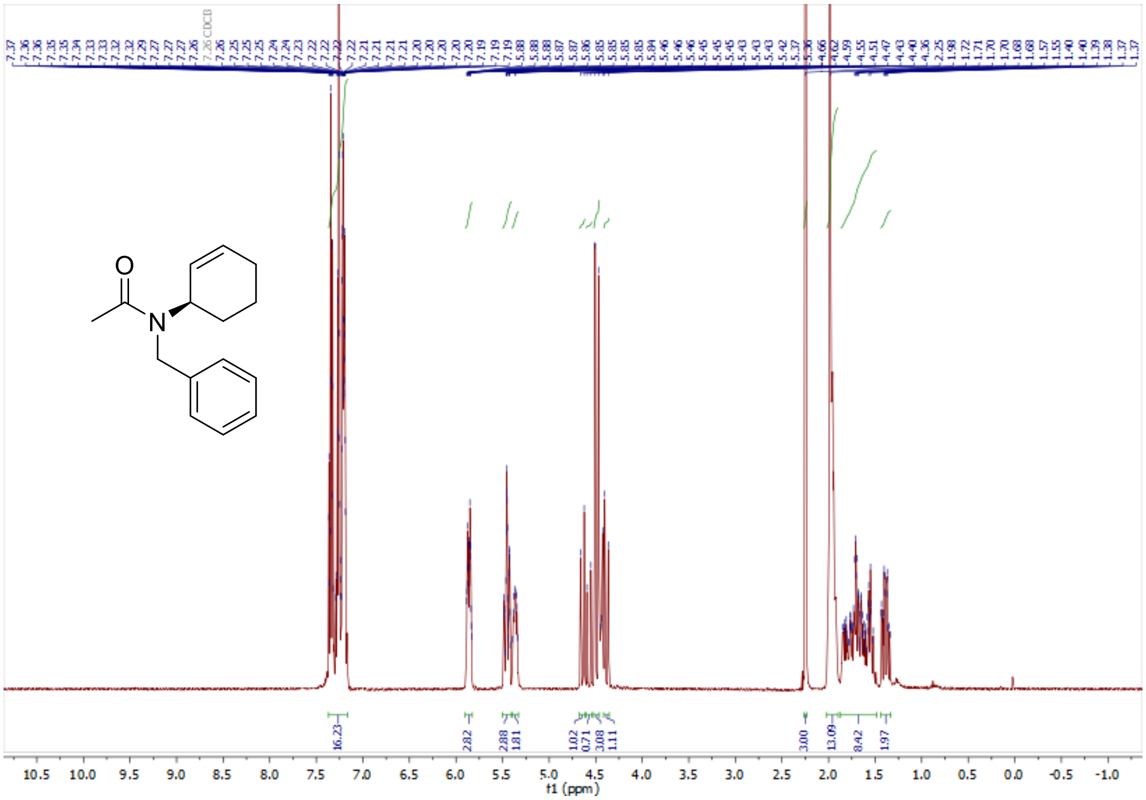

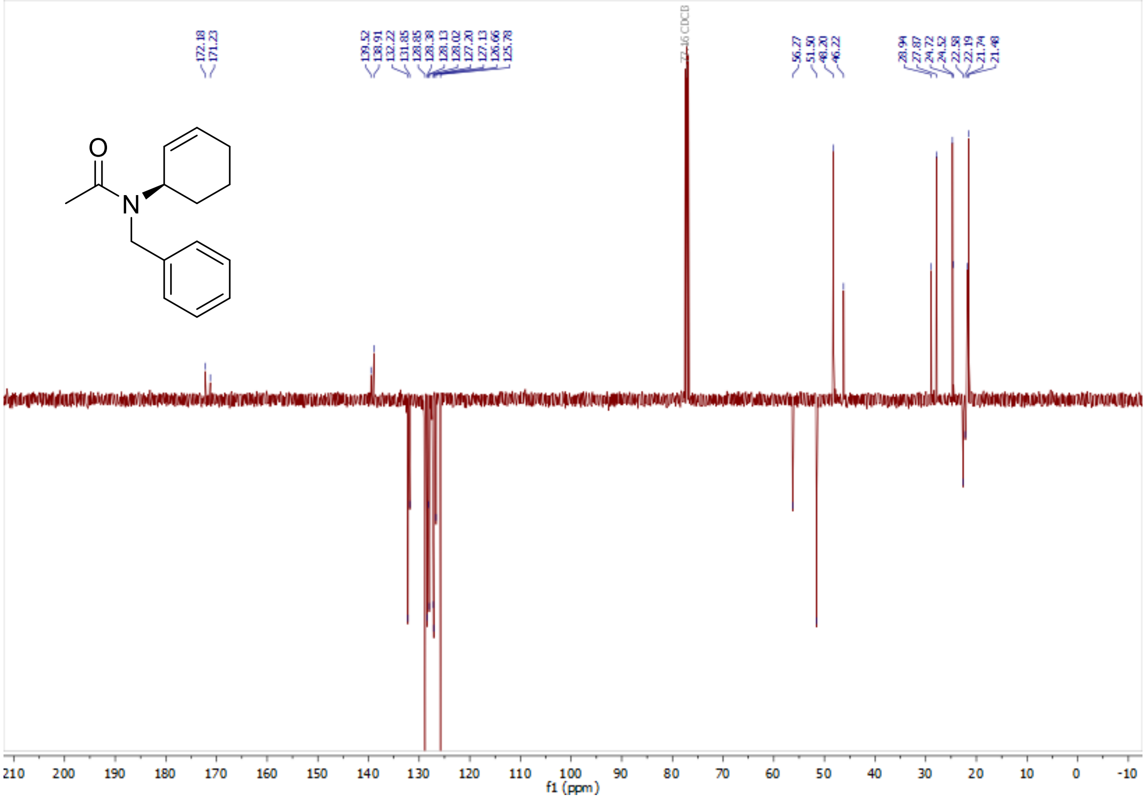

N,*N*-Diallyl-2-(trimethylsilyl)acetamide (8a)

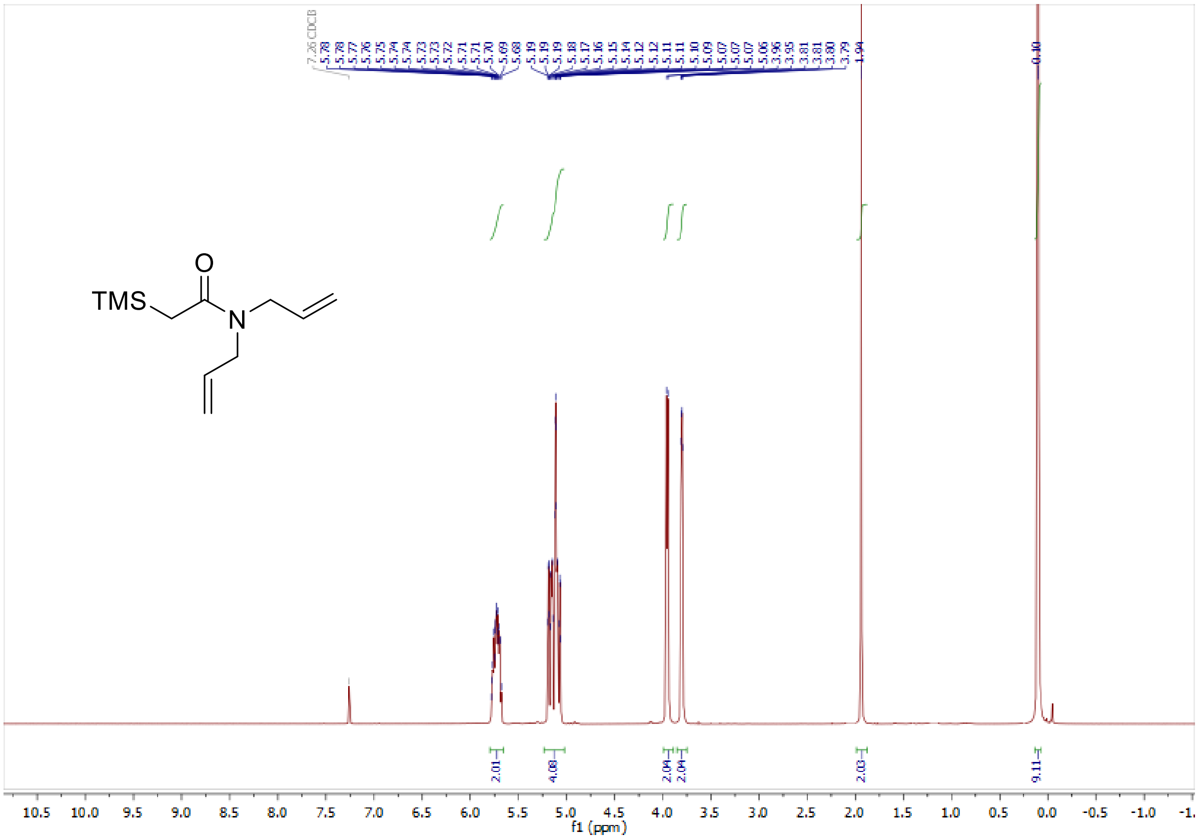

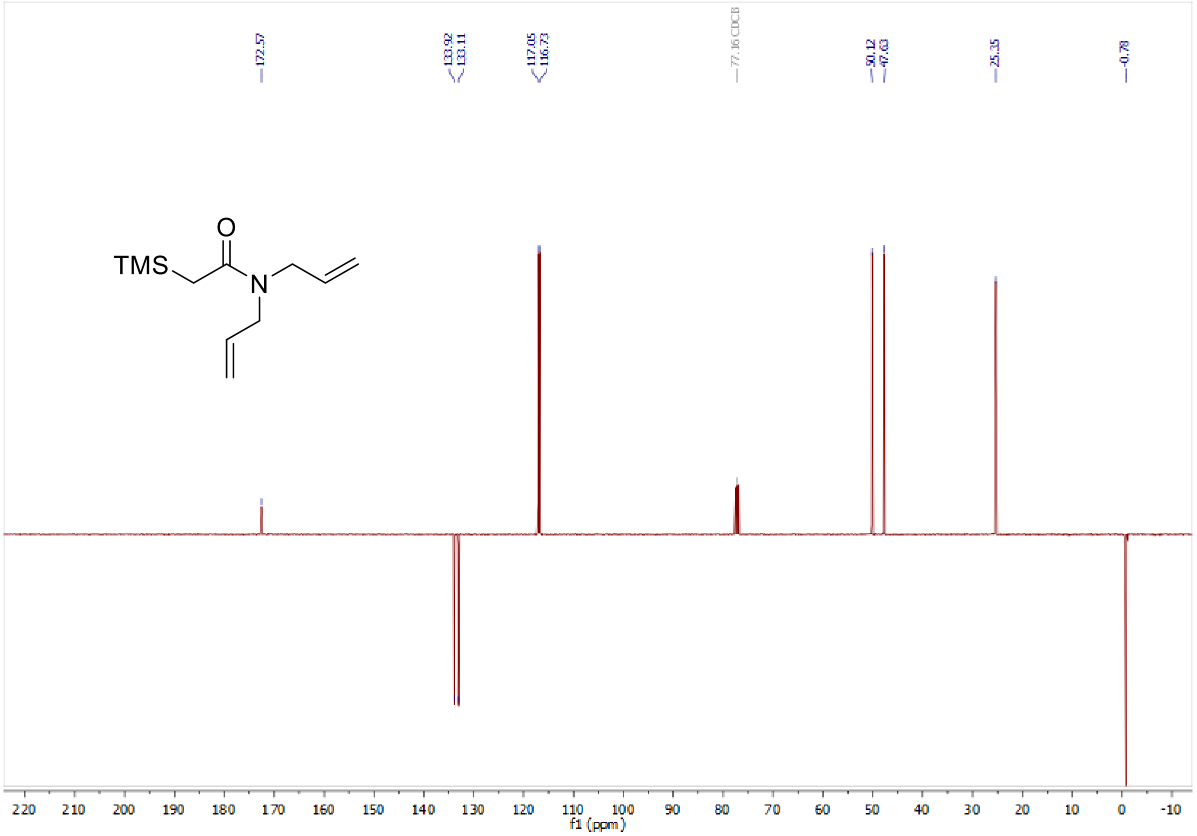

N-Allyl-*N*-methyl-2-(trimethylsilyl)acetamide (8b)

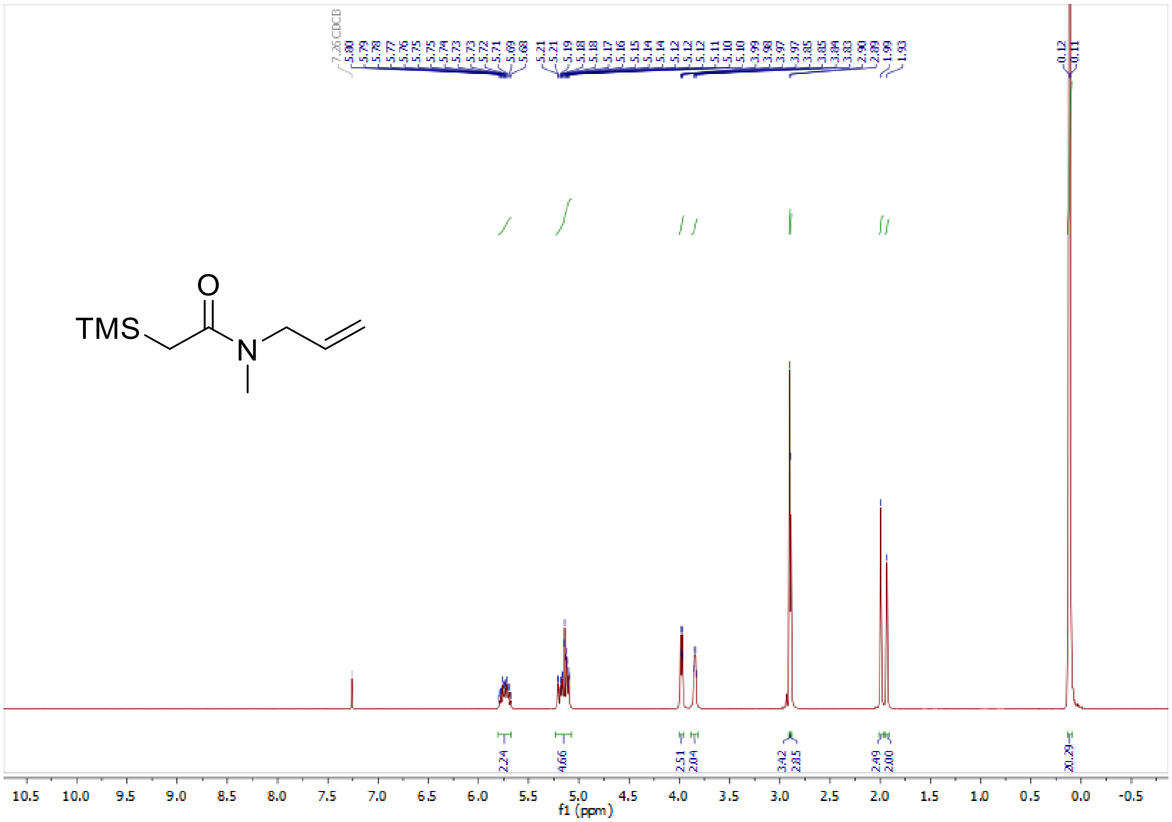

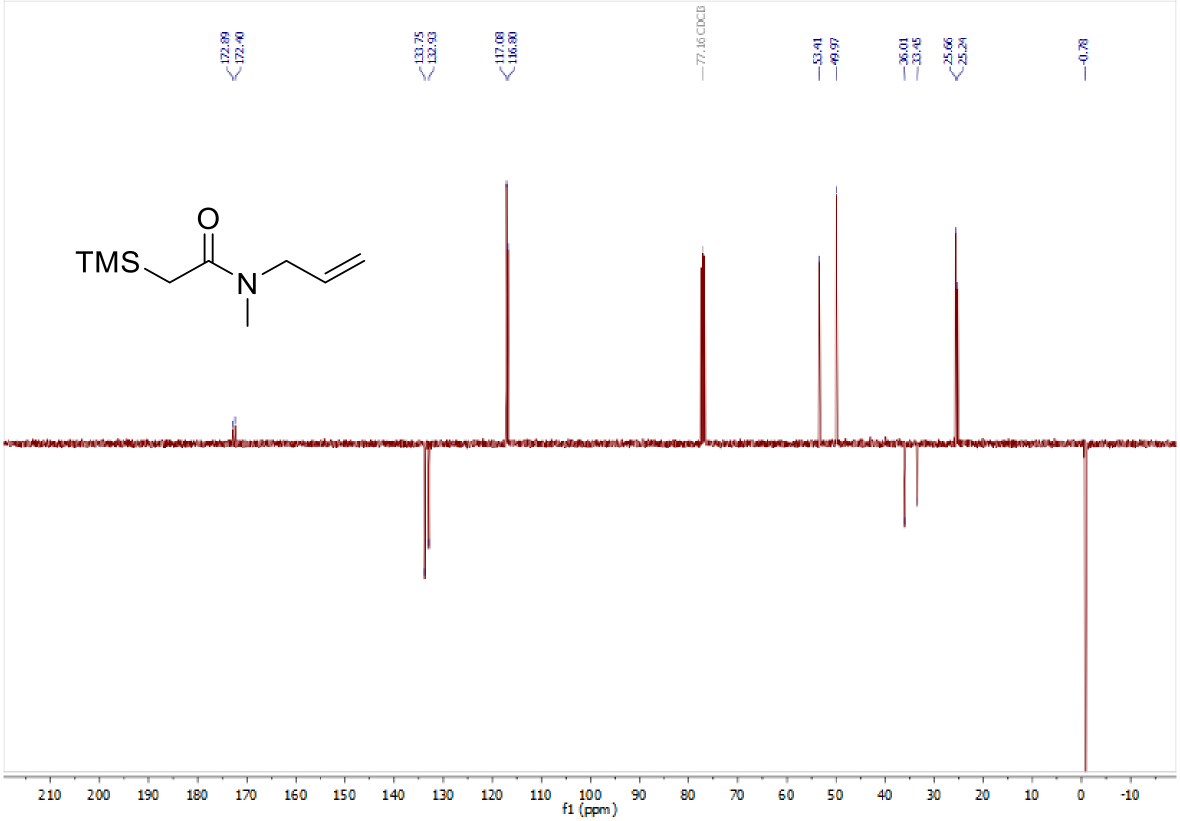

N-Benzyl-N-(2-methylallyl)-2-(trimethylsilyl)acetamide (8c)

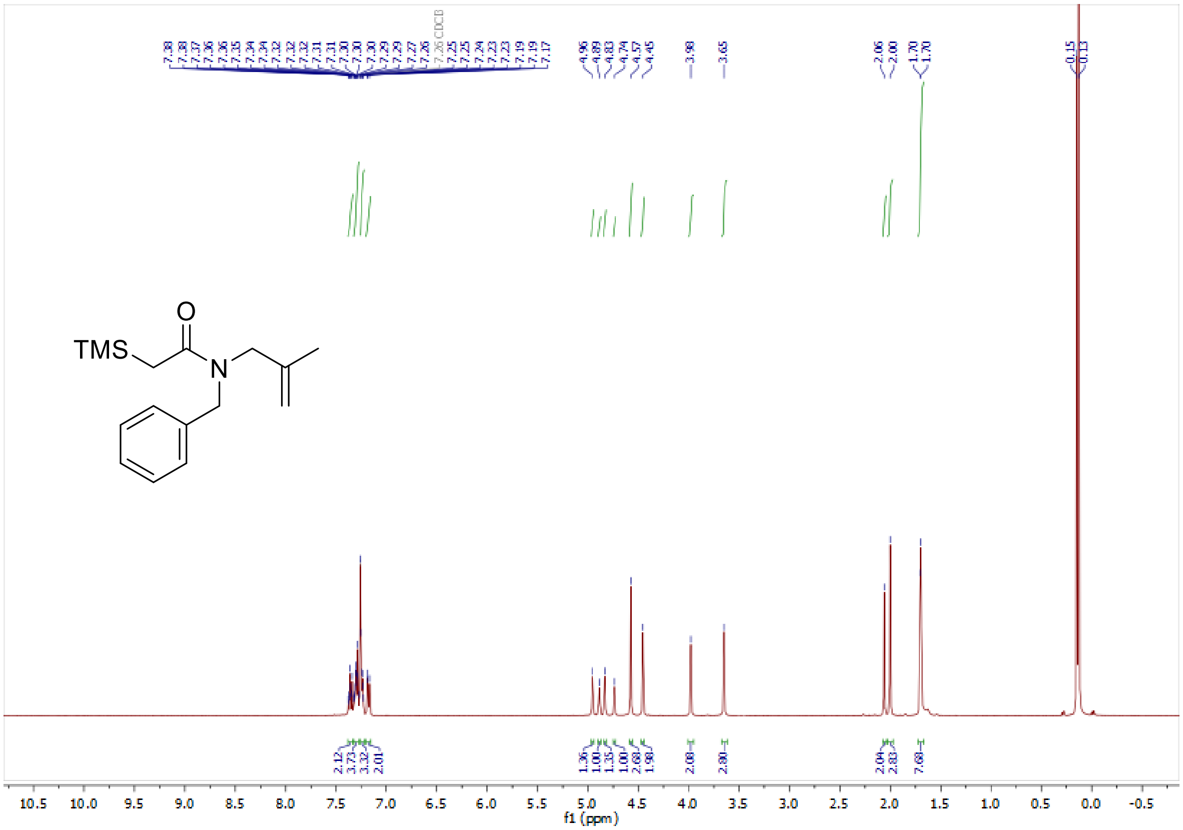

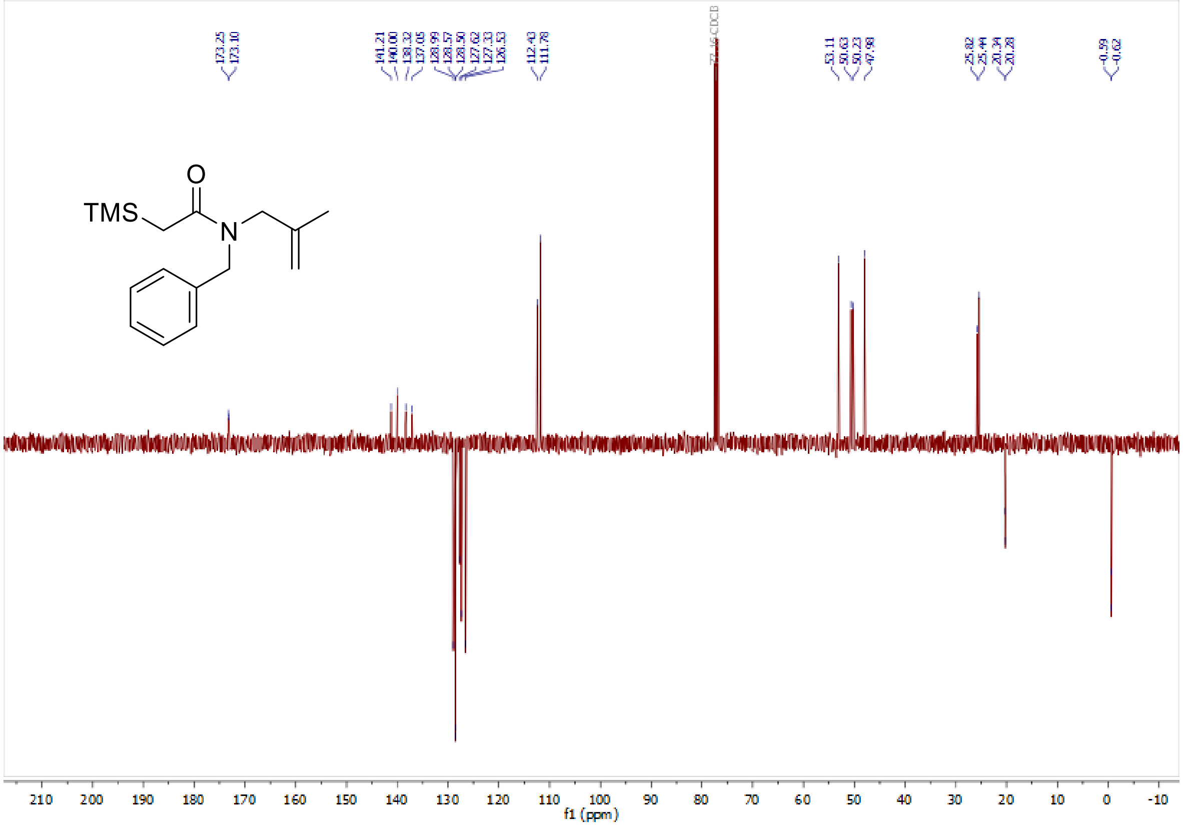

N-Benzyl-N-(3-methylbut-2-en-1-yl)-2-(trimethylsilyl)acetamide (8d)

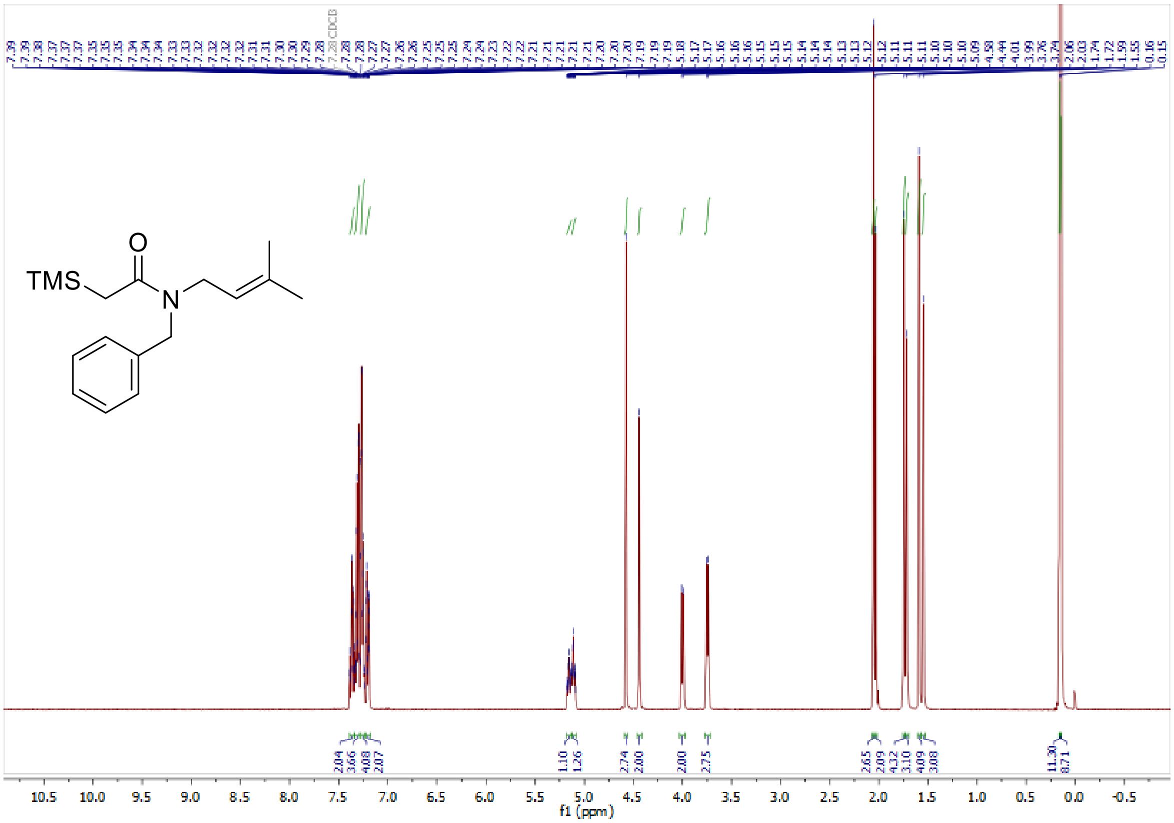

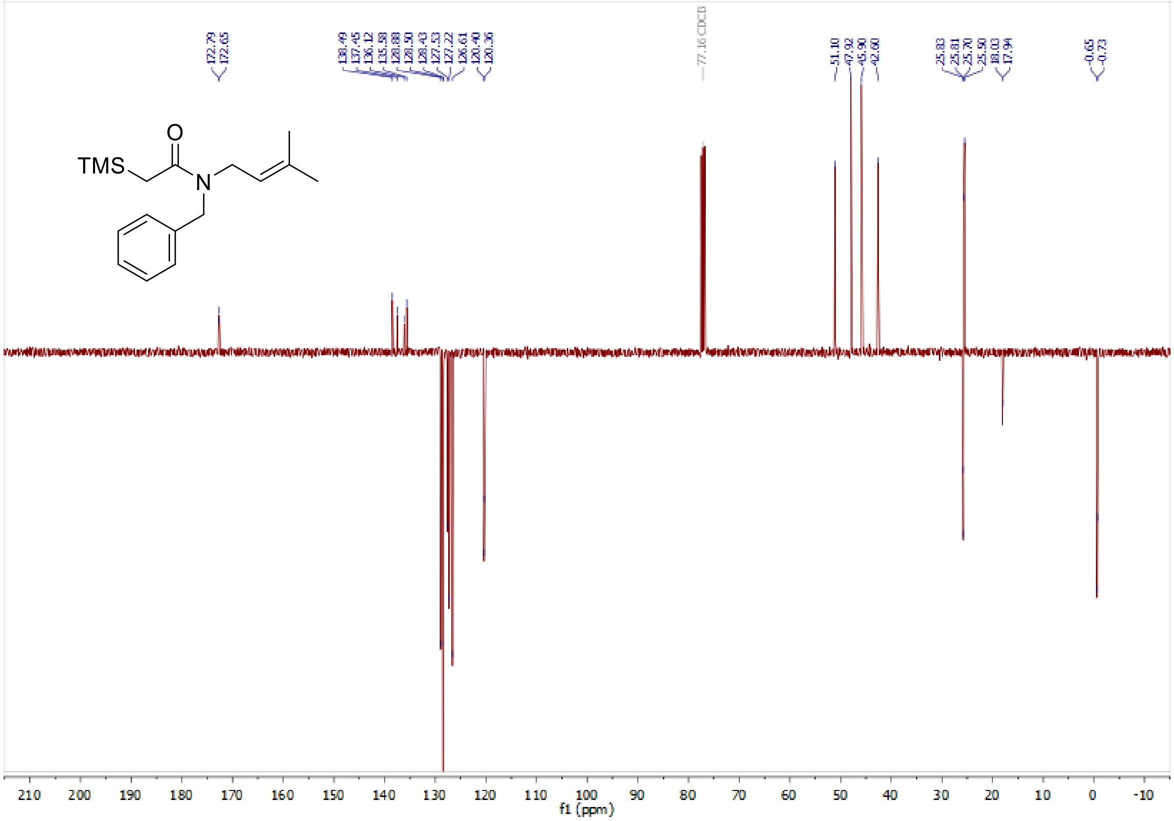

(*S*)-*N*-Allyl-*N*-(1-phenylethyl)-2-(trimethylsilyl)acetamide (8e)

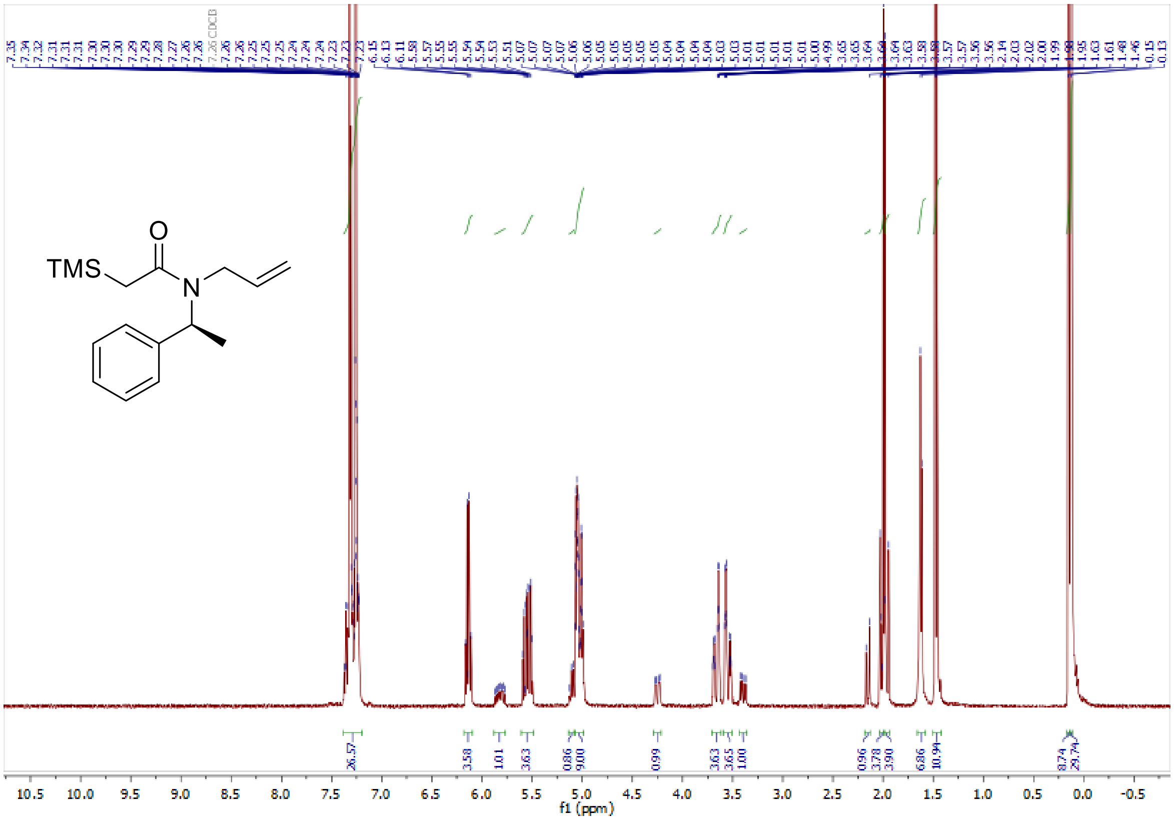

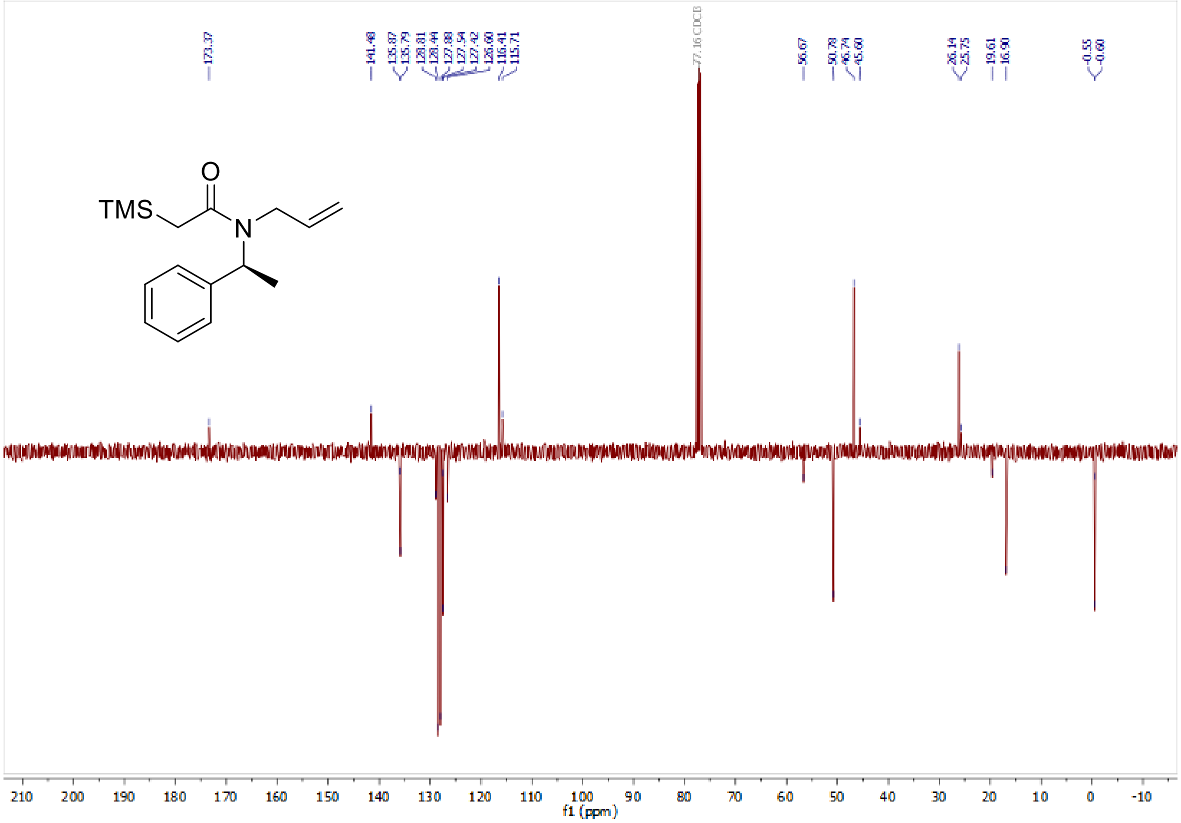

(*S*)-*N*-Allyl-*N*-(1-(naphthalen-2-yl)ethyl)-2-(trimethylsilyl)acetamide (8f)

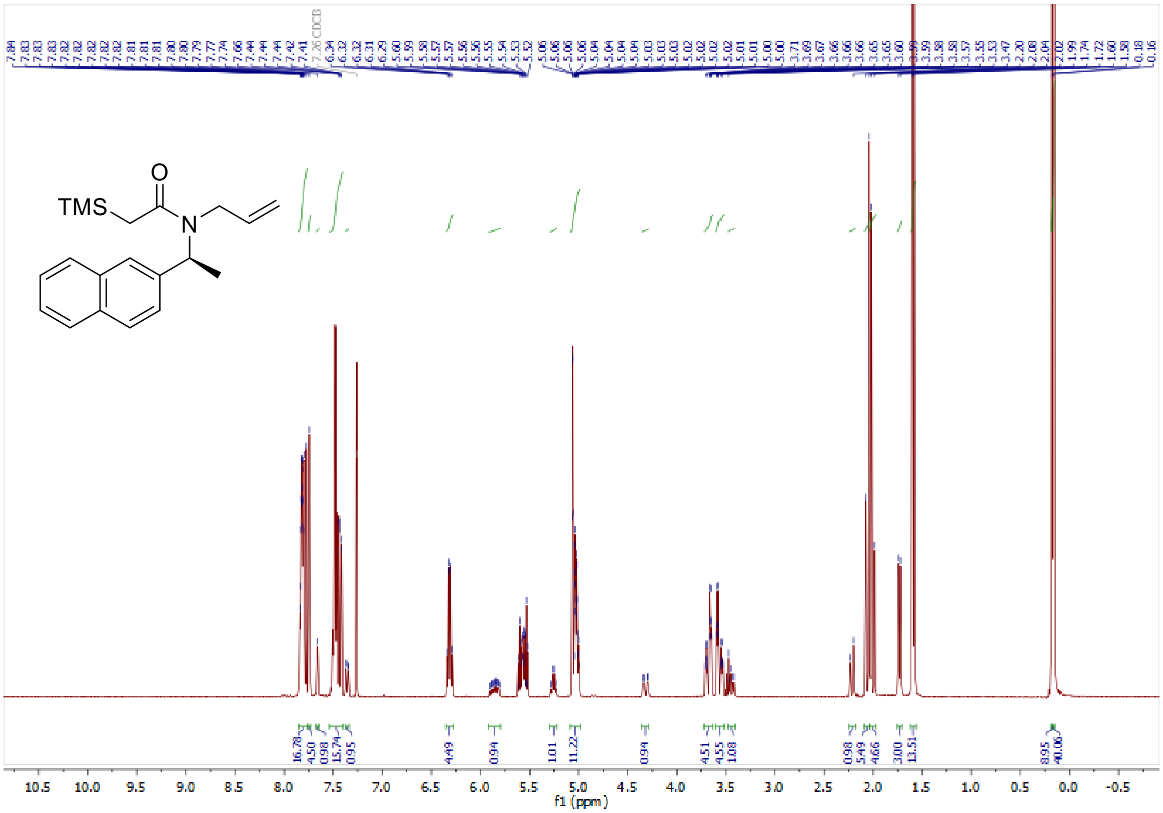

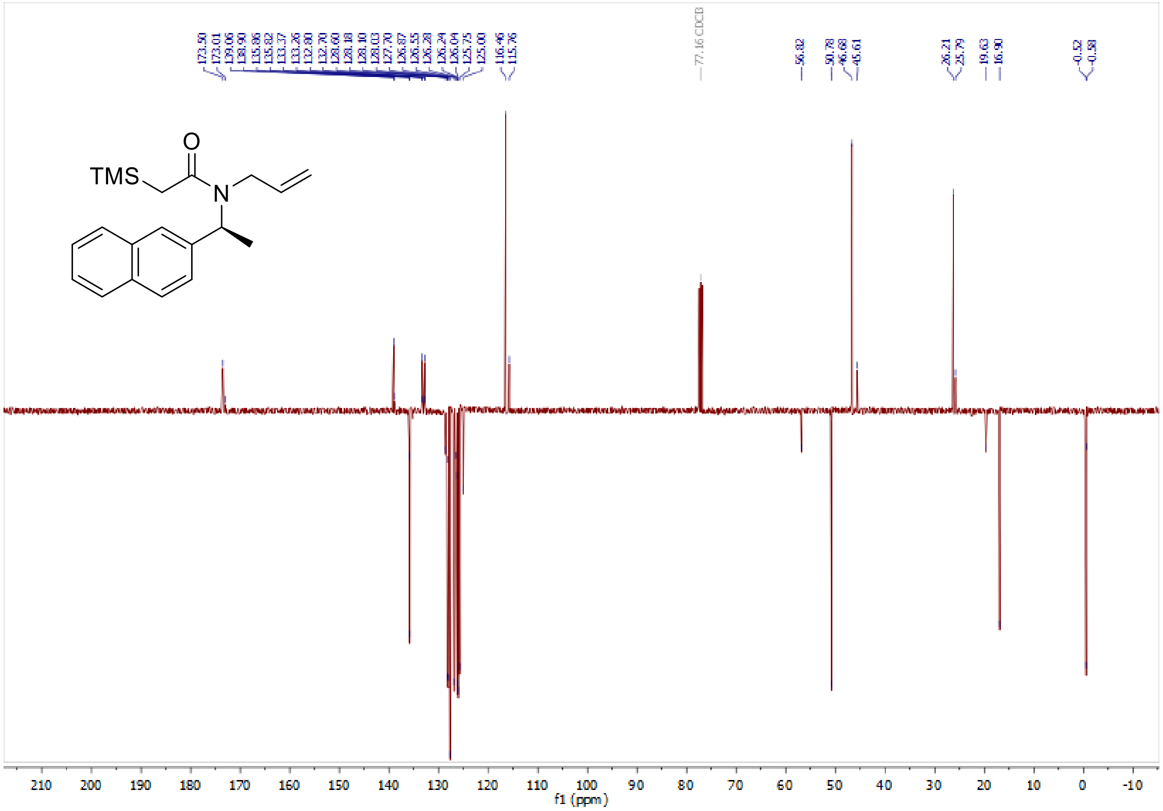

(*S*)-*N*-(3-Methylbut-2-en-1-yl)-*N*-(1-phenylethyl)-2-(trimethylsilyl)acetamide (8g)

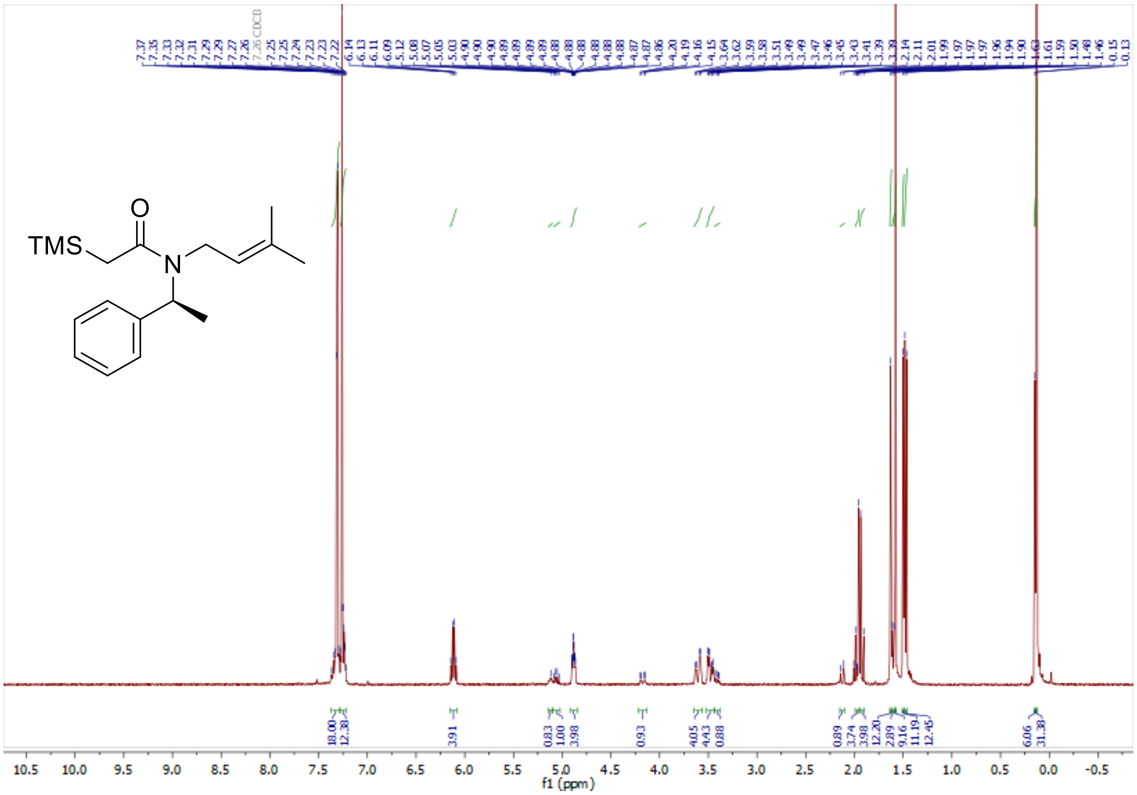

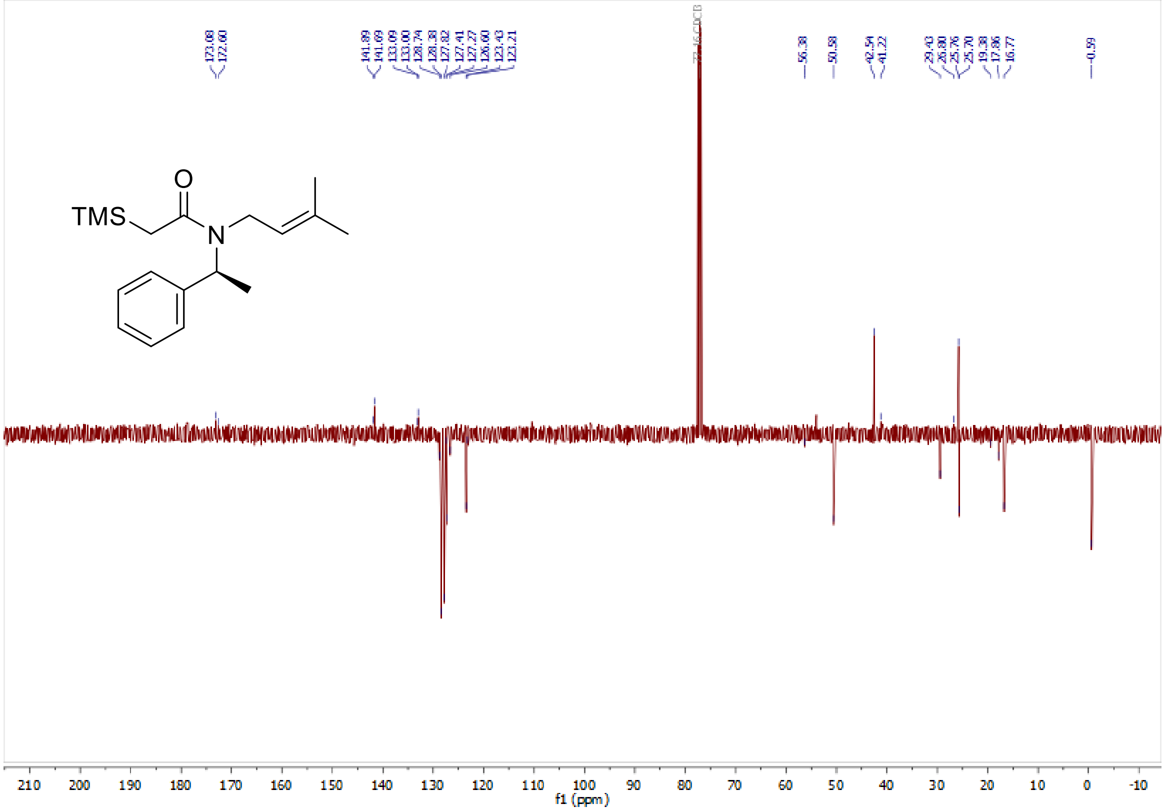

(*S*)-*N*-(2-Methylallyl)-*N*-(1-phenylethyl)-2-(trimethylsilyl)acetamide (8h)

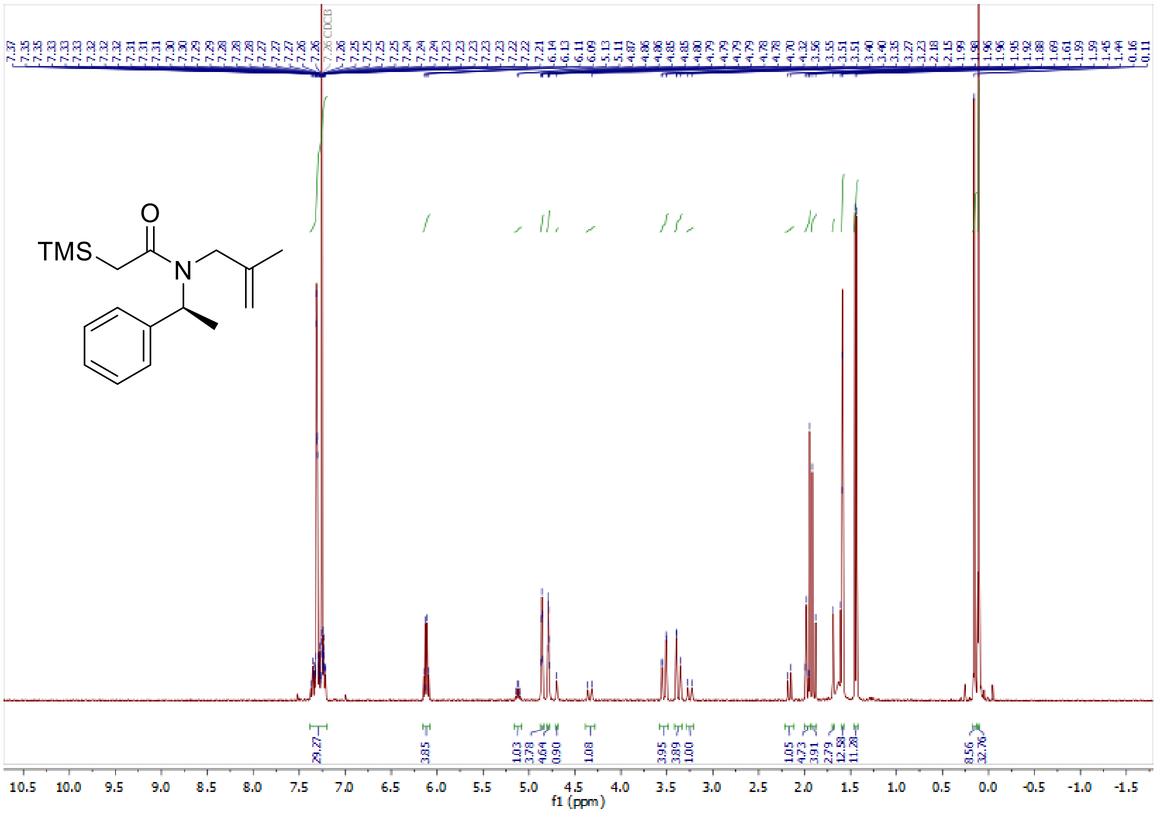

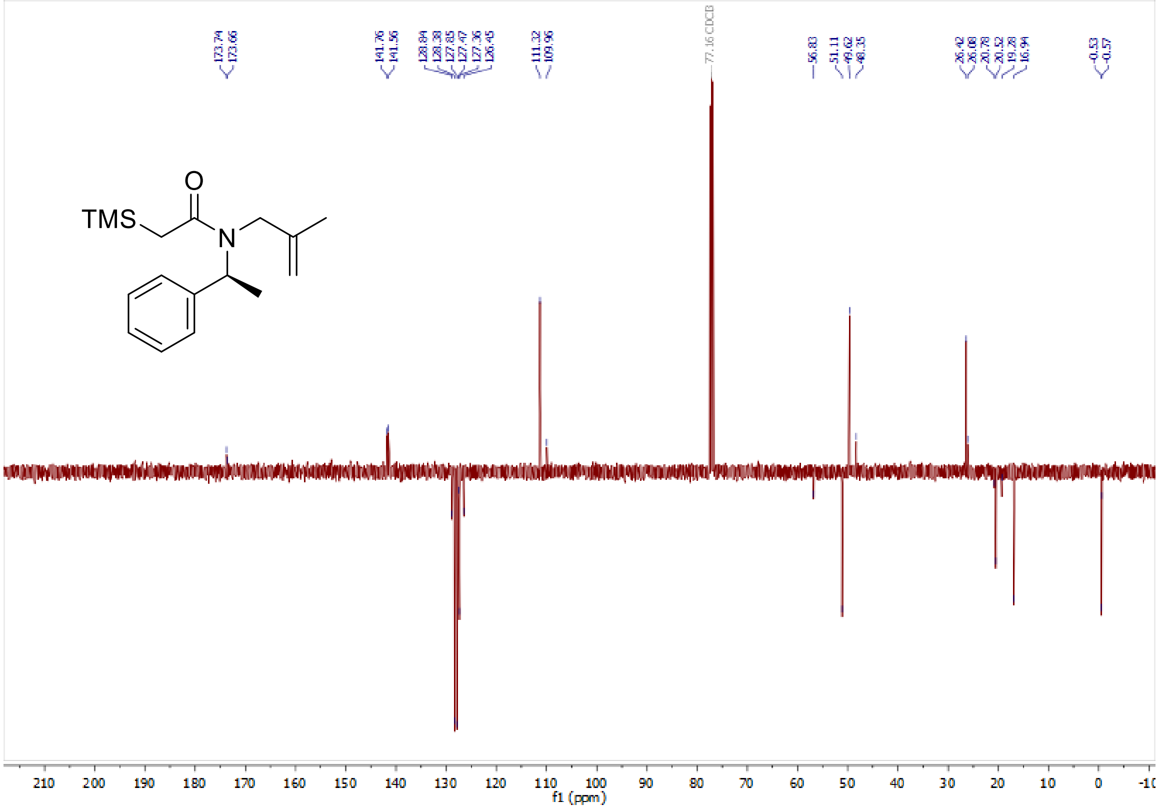

(*S*)-*N*-(Cyclopent-1-en-1-ylmethyl)-*N*-(1-phenylethyl)-2-(trimethylsilyl)acetamide (8i)

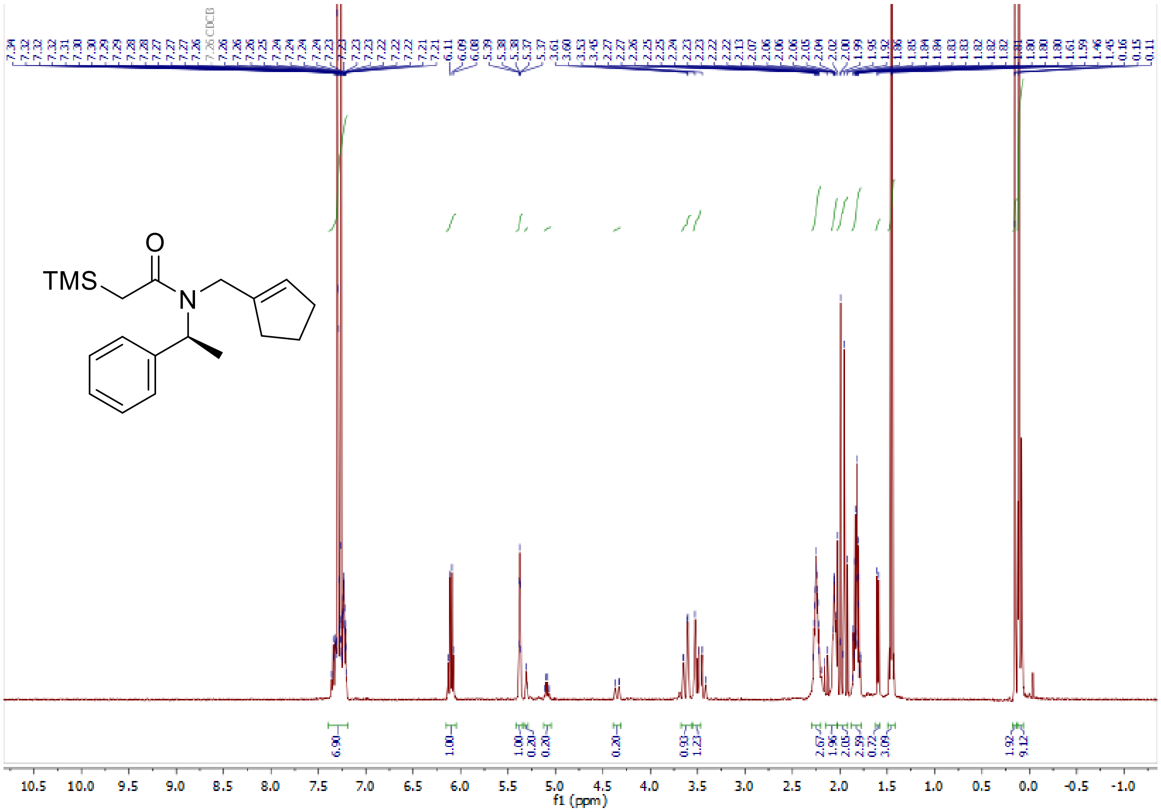

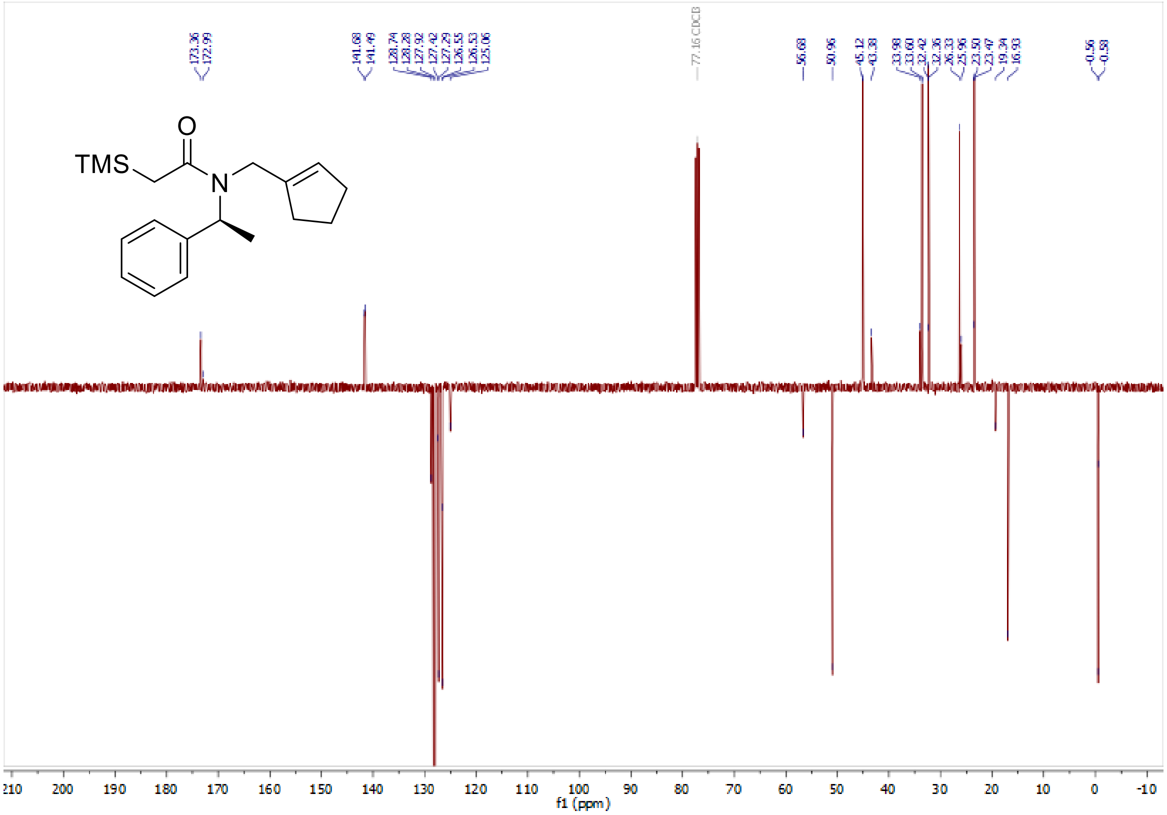

N-Benzyl-*N*-(cyclopent-1-en-1-ylmethyl)-2-(trimethylsilyl)acetamide (8j)

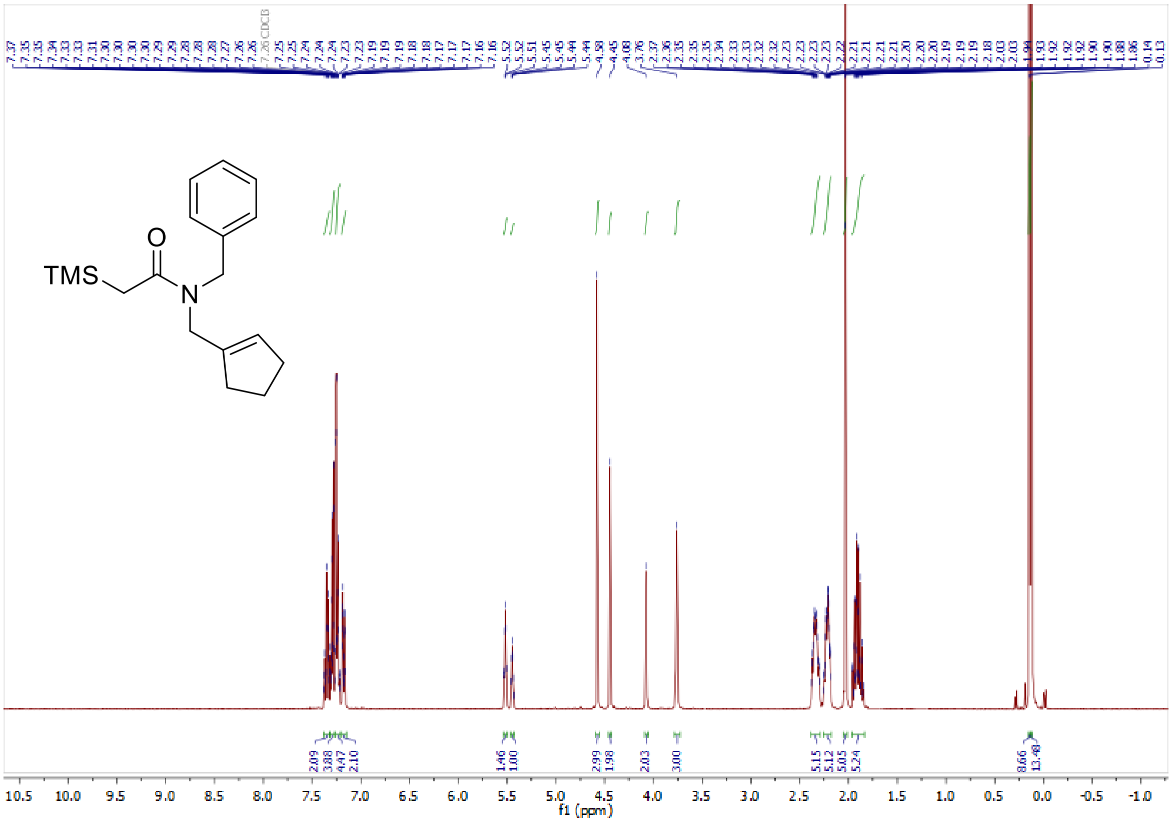

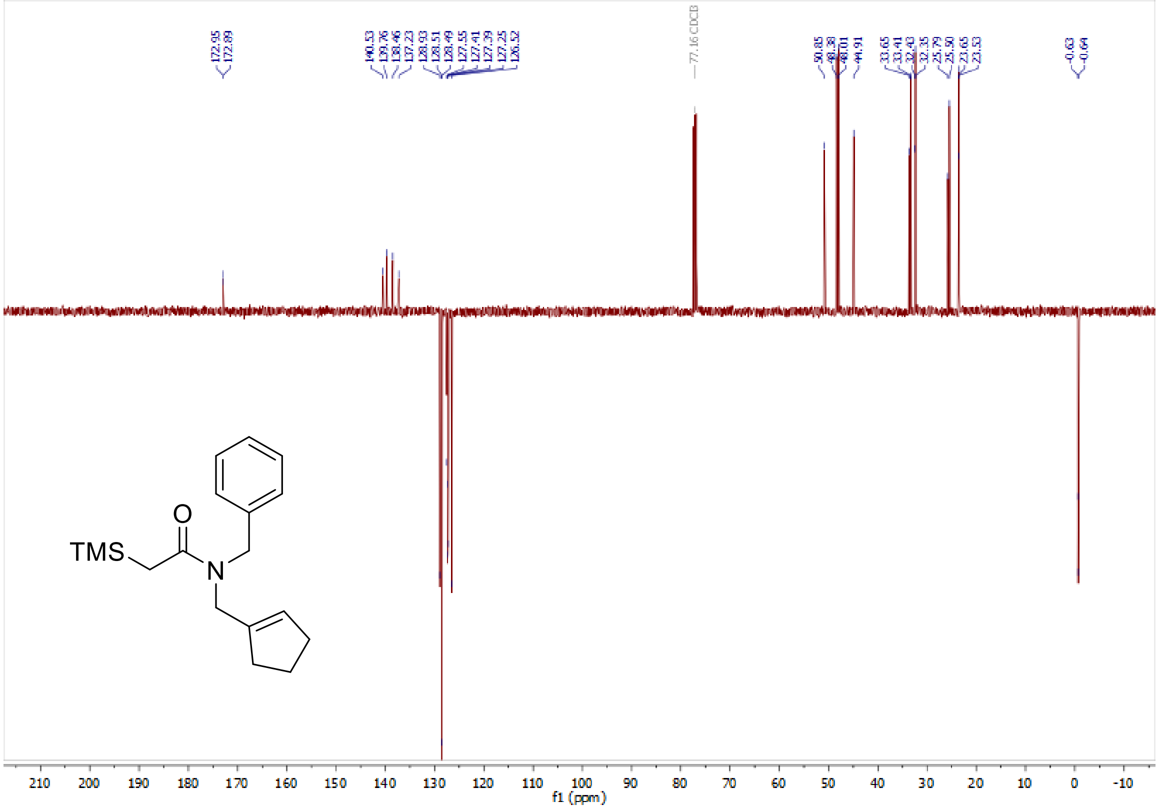

N-Benzyl-*N*-(cyclohex-1-en-1-ylmethyl)-2-(trimethylsilyl)acetamide (8k)

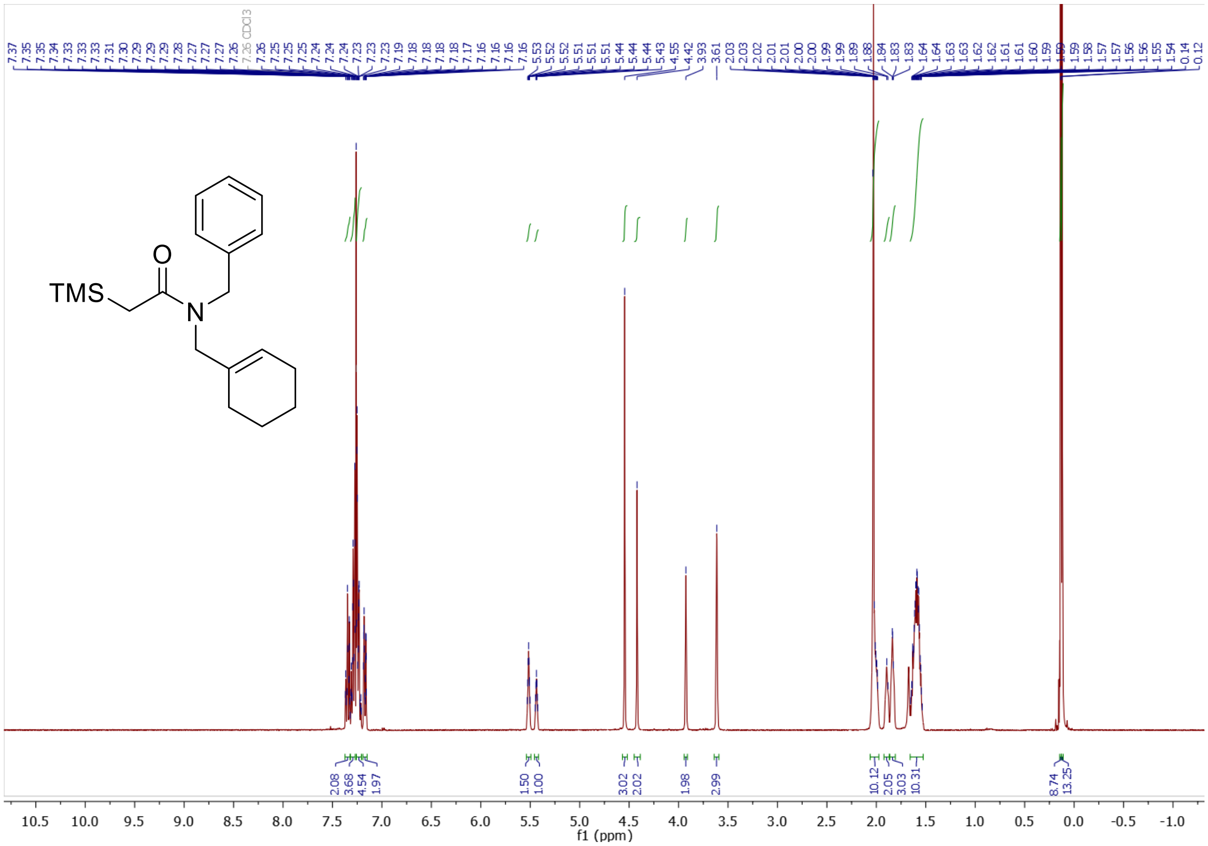

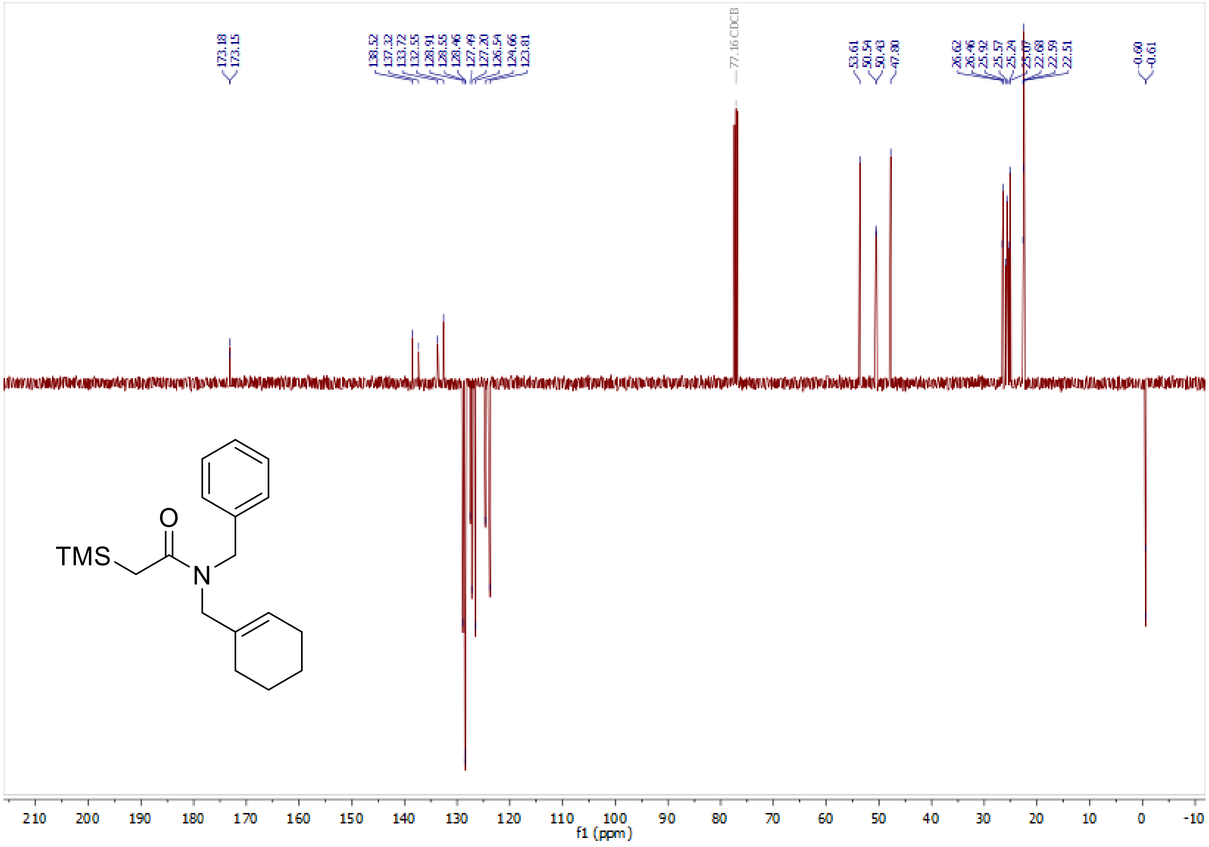

N-Benzyl-*N*-(cyclopent-2-en-1-yl)-2-(trimethylsilyl)acetamide (8l)

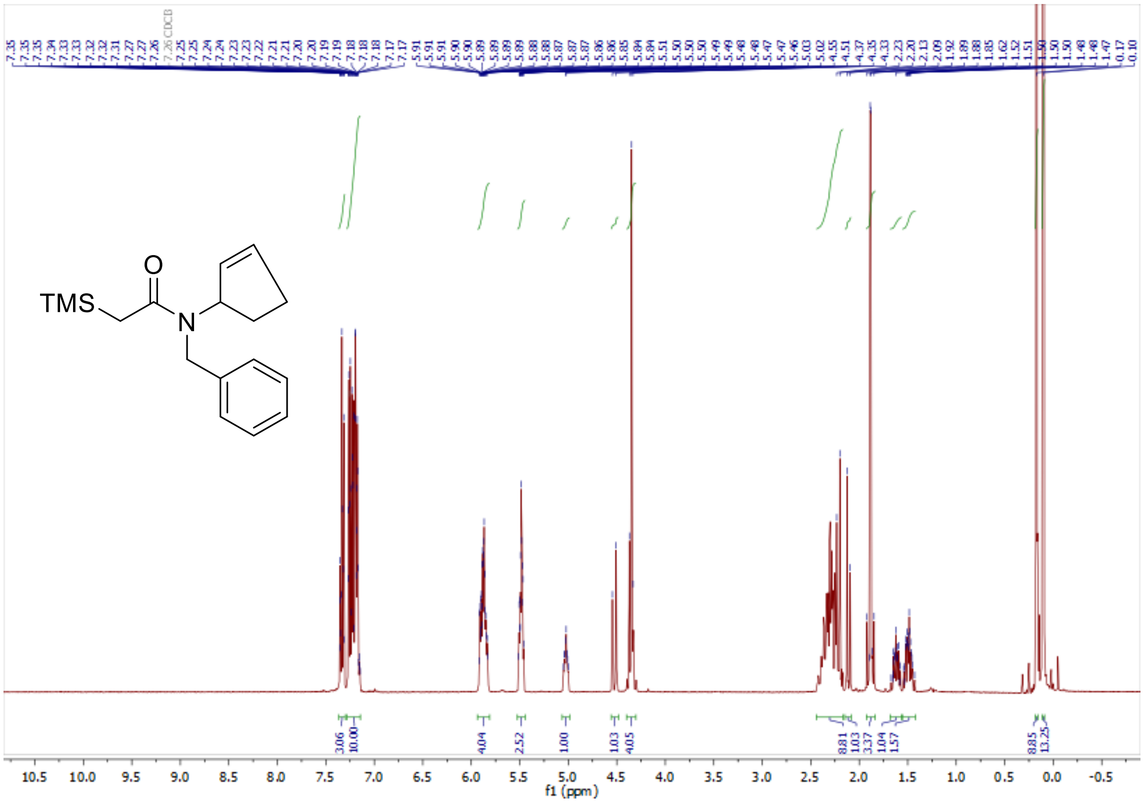

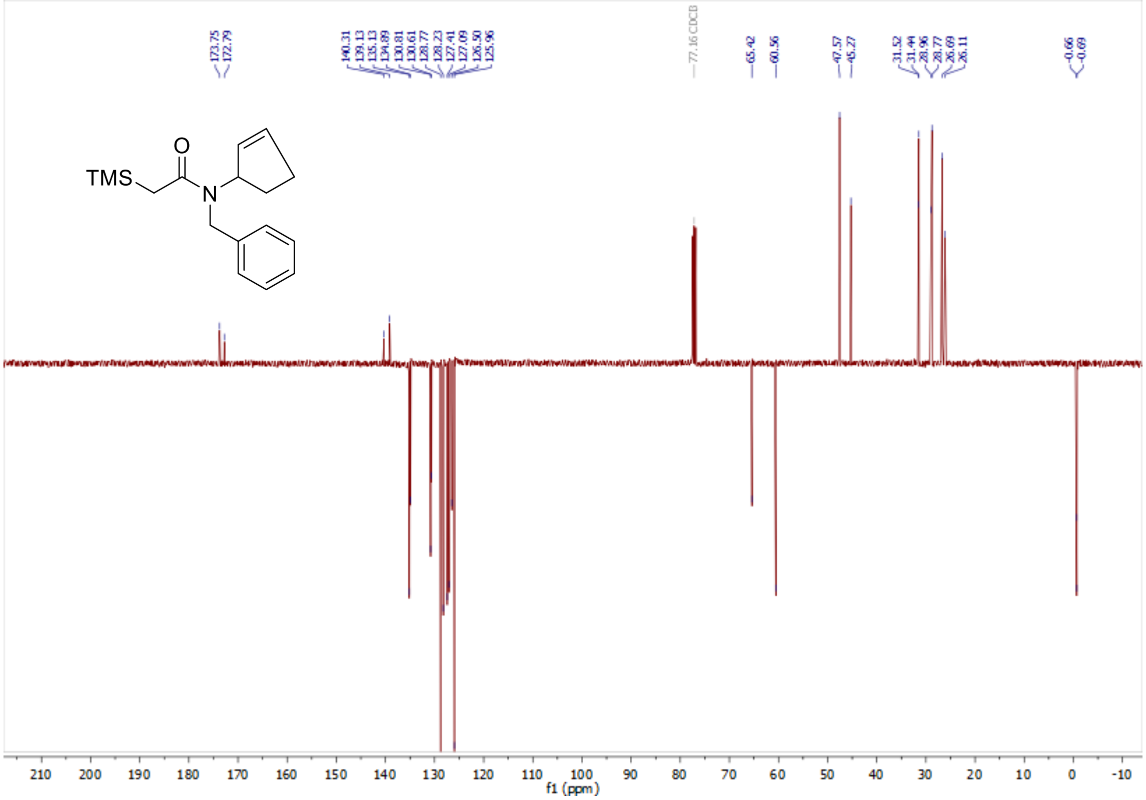

(*R*)-*N*-Benzyl-*N*-(cyclohex-2-en-1-yl)-2-(trimethylsilyl)acetamide (8m)

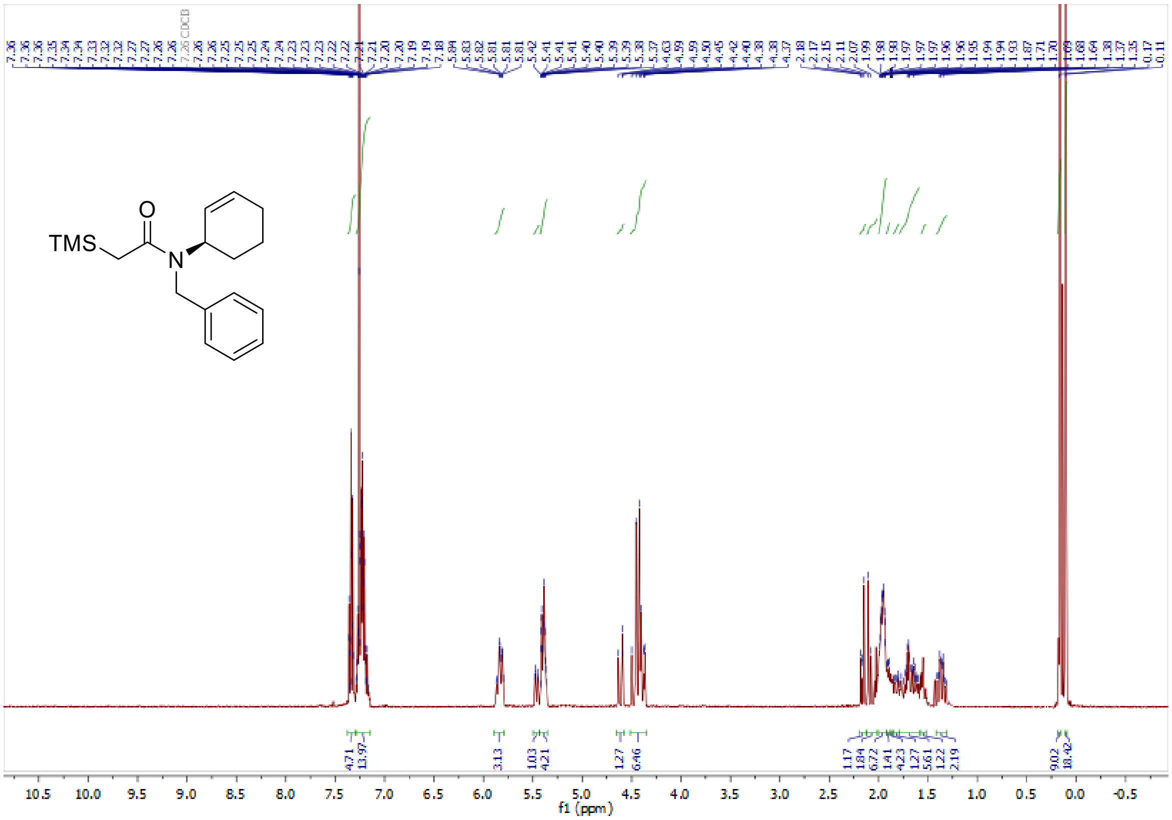

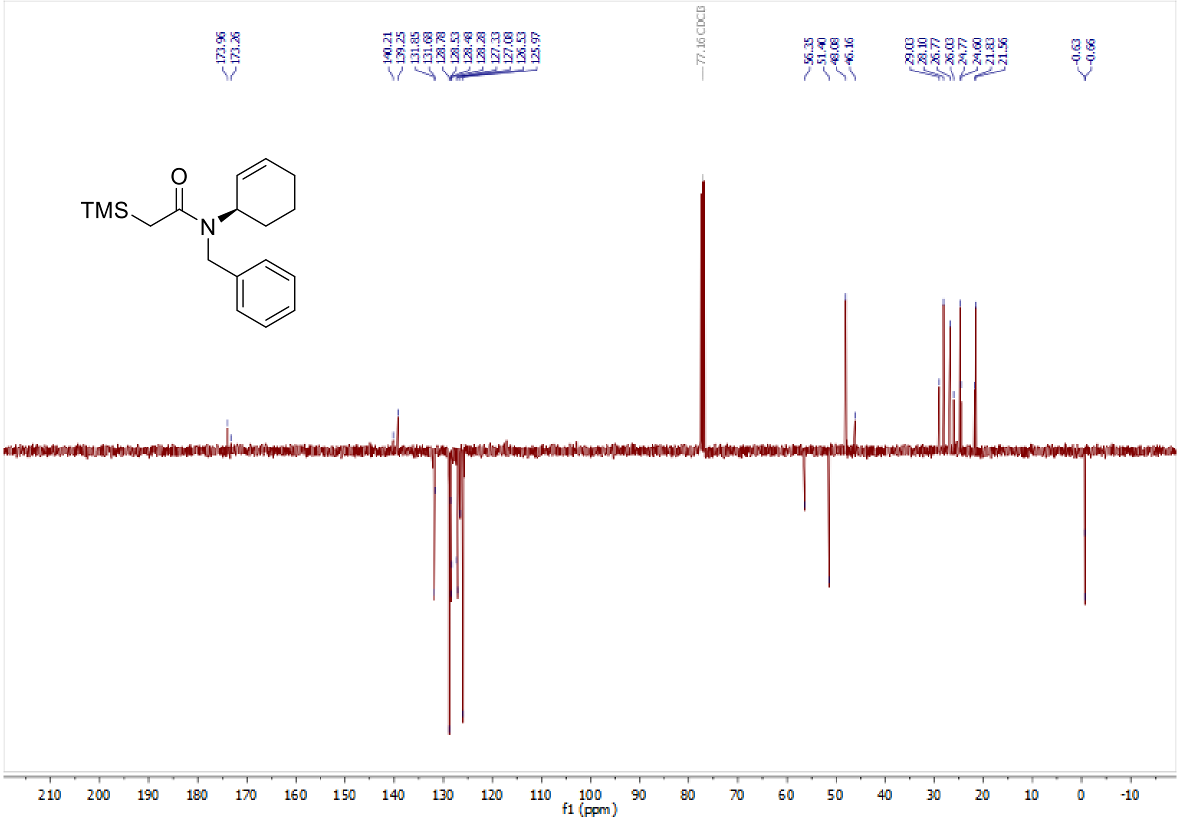

***N*,*N*-Diallyl-4-methyl-2-((2,2,6,6-tetramethylpiperidin-1-yl)oxy)-4-((trimethylsilyl)oxy)pentanamide (9a)**

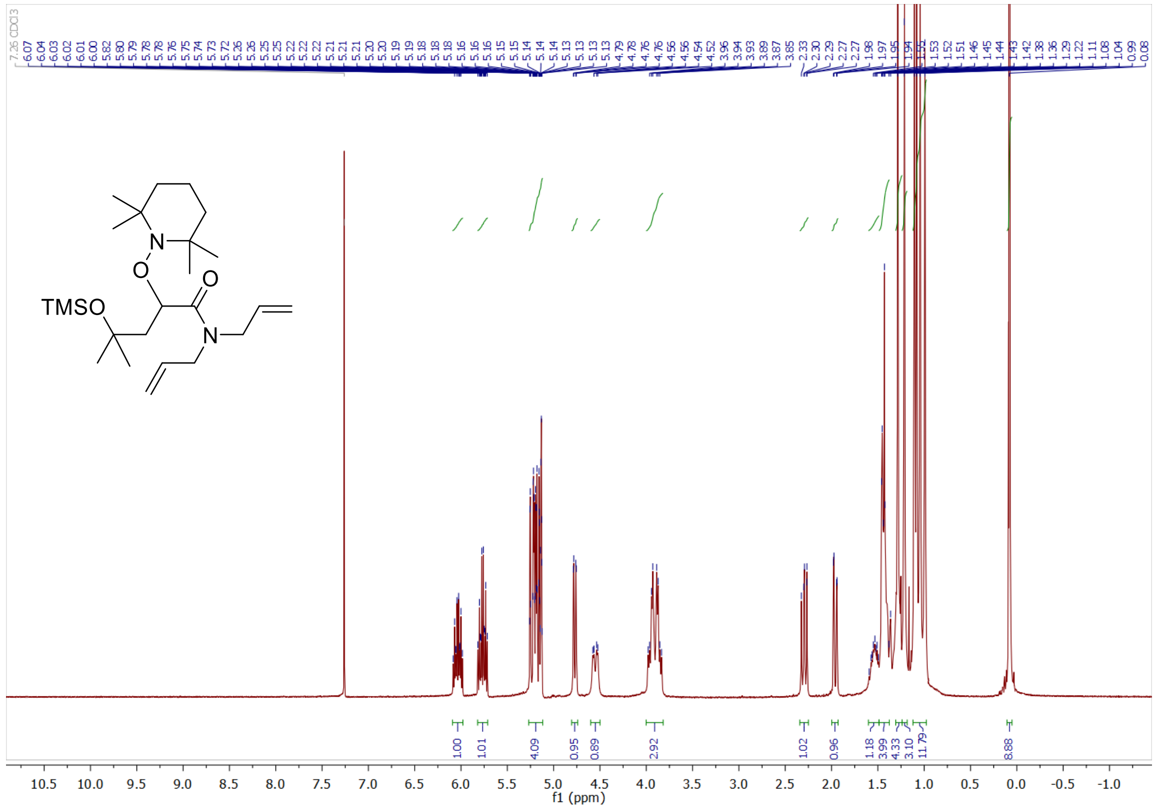

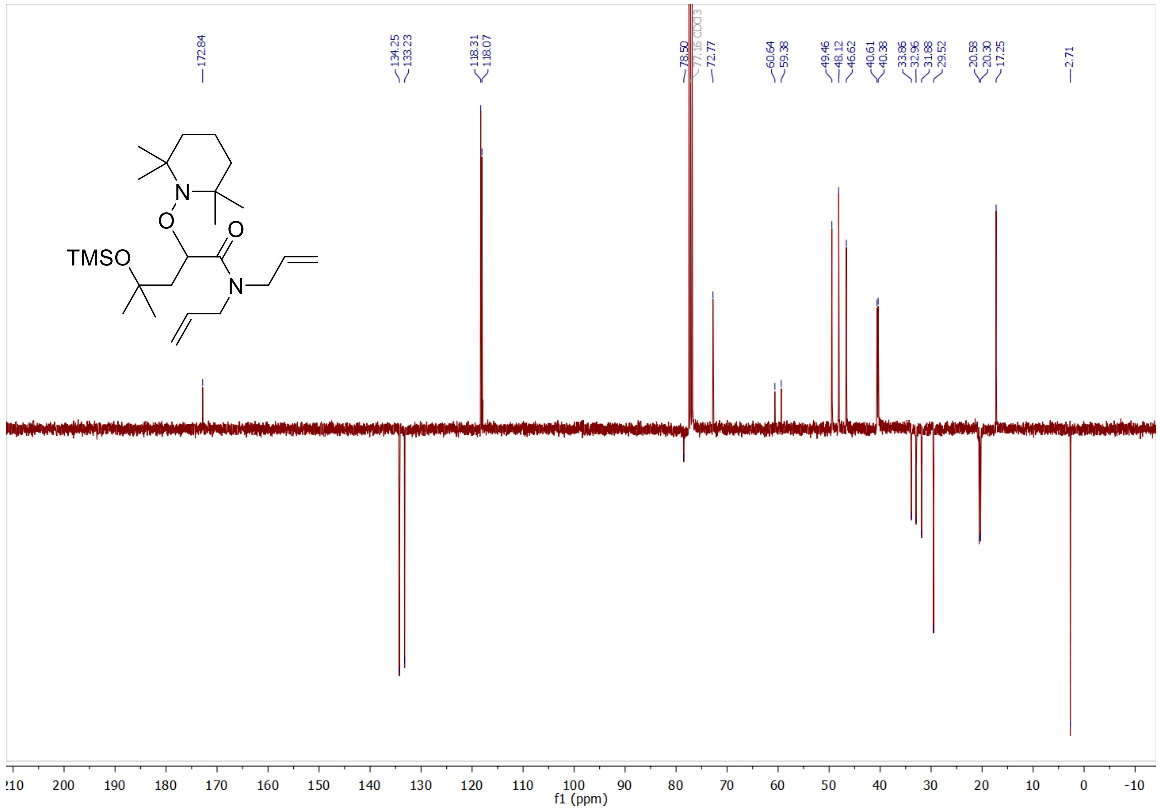

N,*N*-Diallyl-2-((2,2,6,6-tetramethylpiperidin-1-yl)oxy)-4-((trimethylsilyl)oxy)pentanamide (9b)

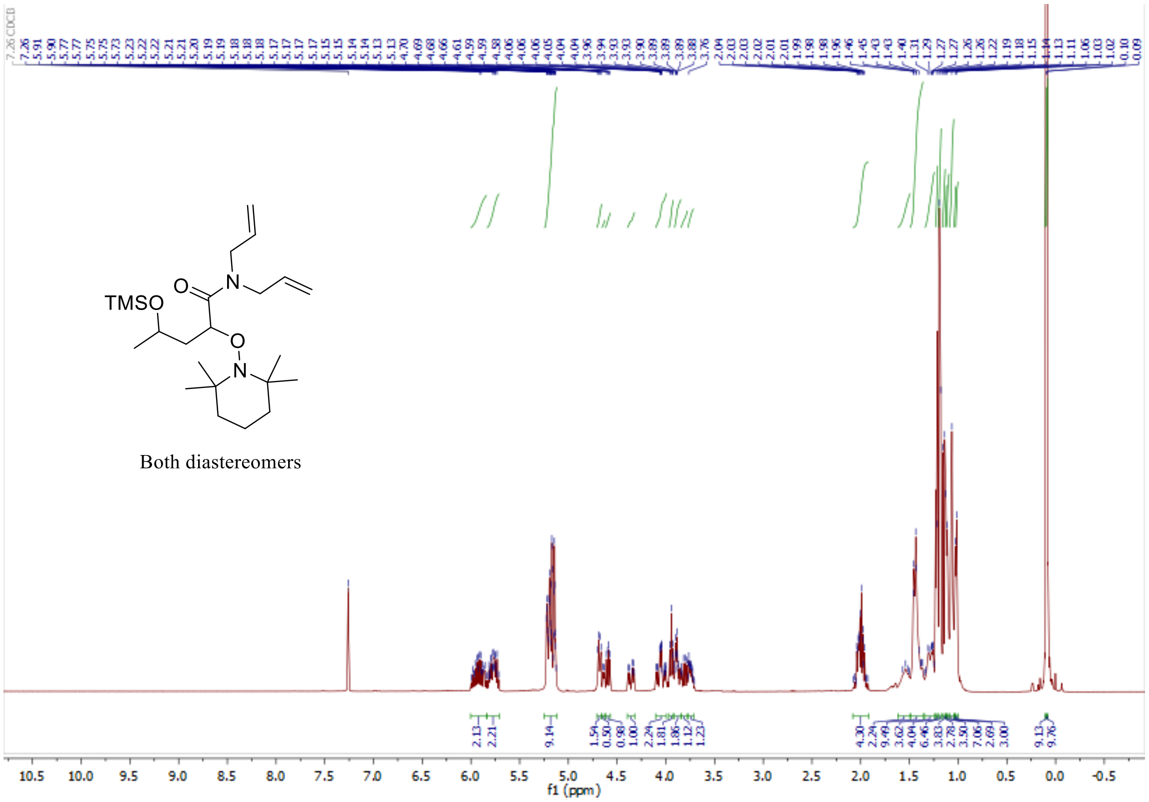

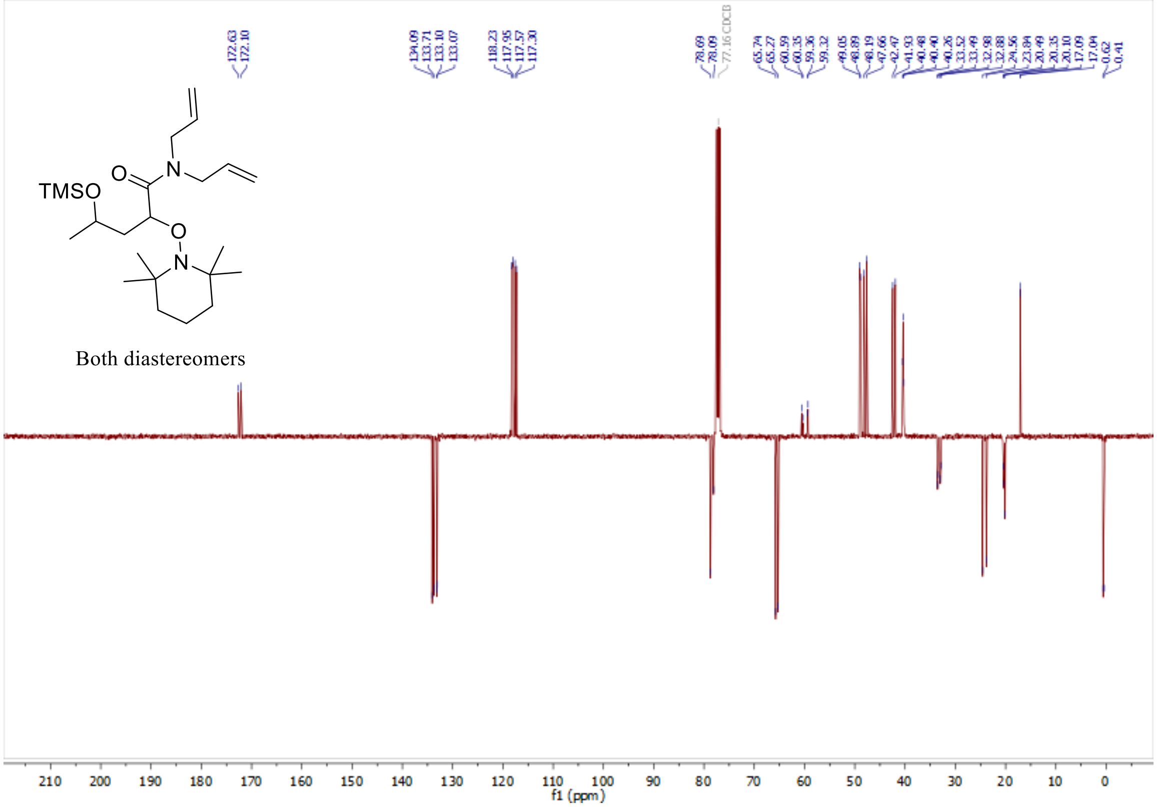

N,*N*-Diallyl-2-((2,2,6,6-tetramethylpiperidin-1-yl)oxy)-4-((trimethylsilyl)oxy)octanamide (9c)

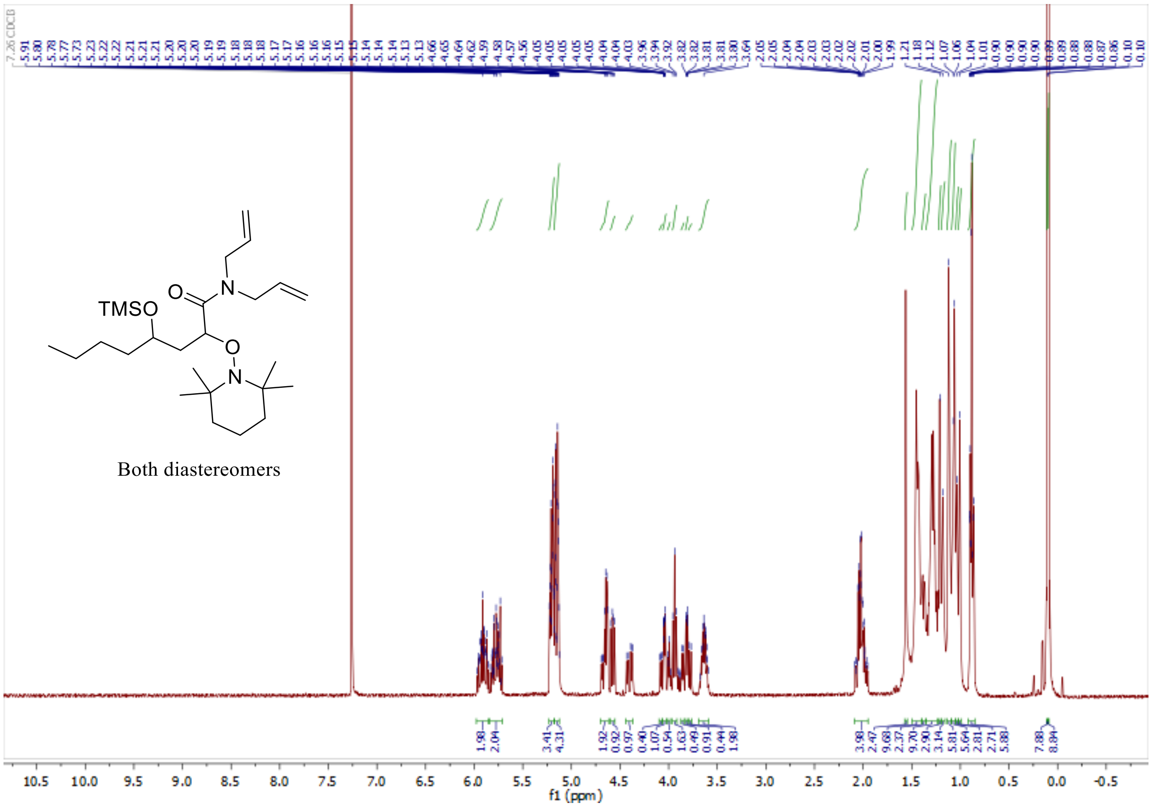

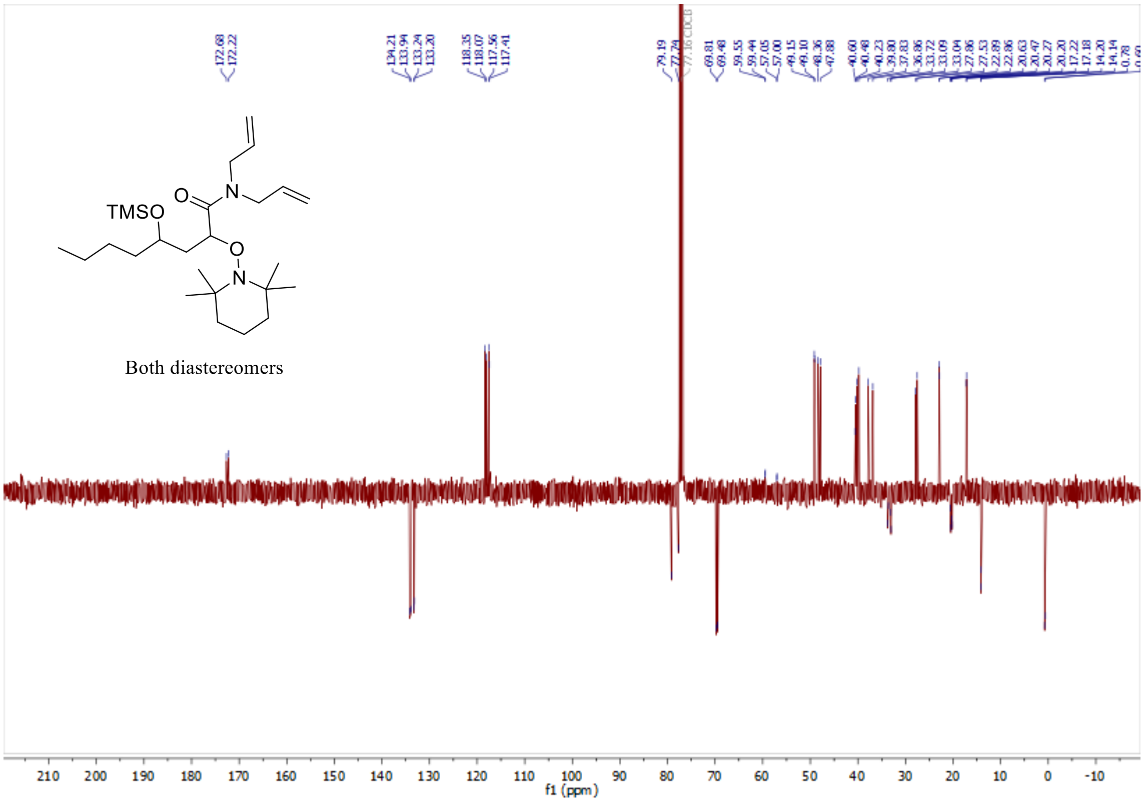

N,*N*-Diallyl-4-phenyl-2-((2,2,6,6-tetramethylpiperidin-1-yl)oxy)-4-((trimethylsilyl)oxy)butanamide (9d)

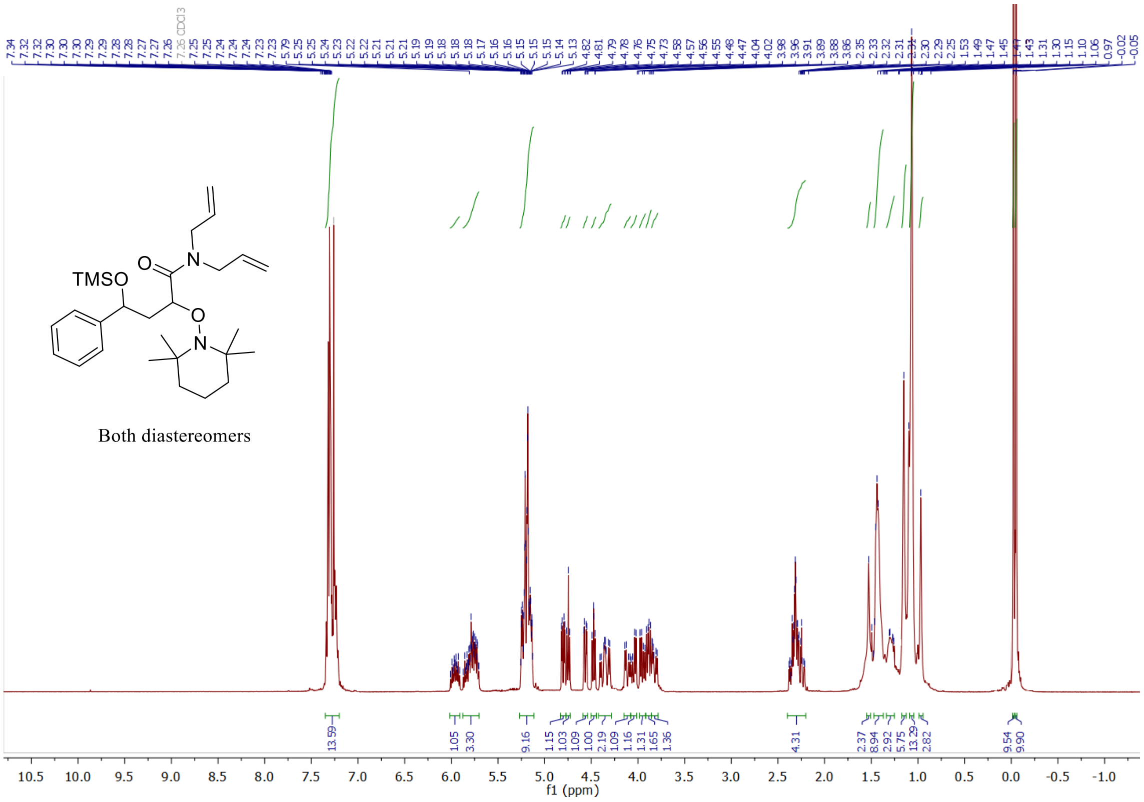

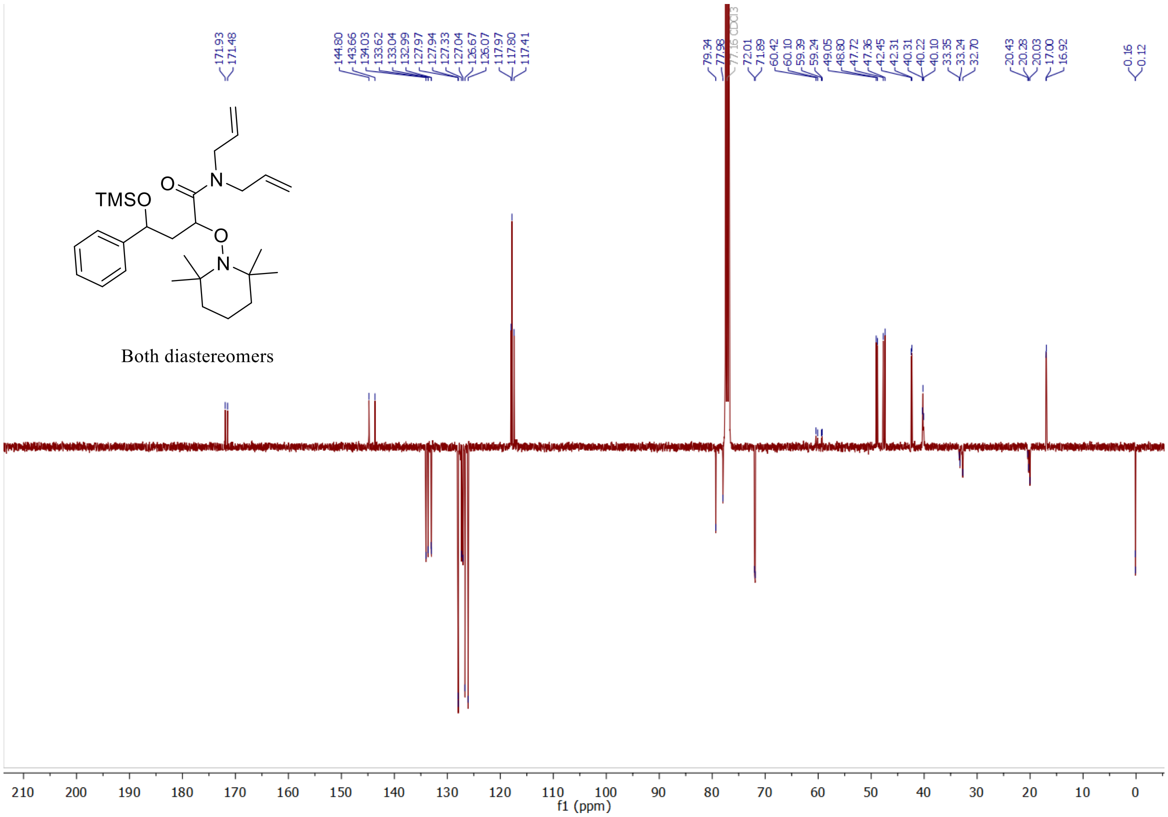

N-Allyl-N-methyl-2-((2,2,6,6-tetramethylpiperidin-1-yl)oxy)-4-((trimethylsilyl)oxy)pentanamide (9e)

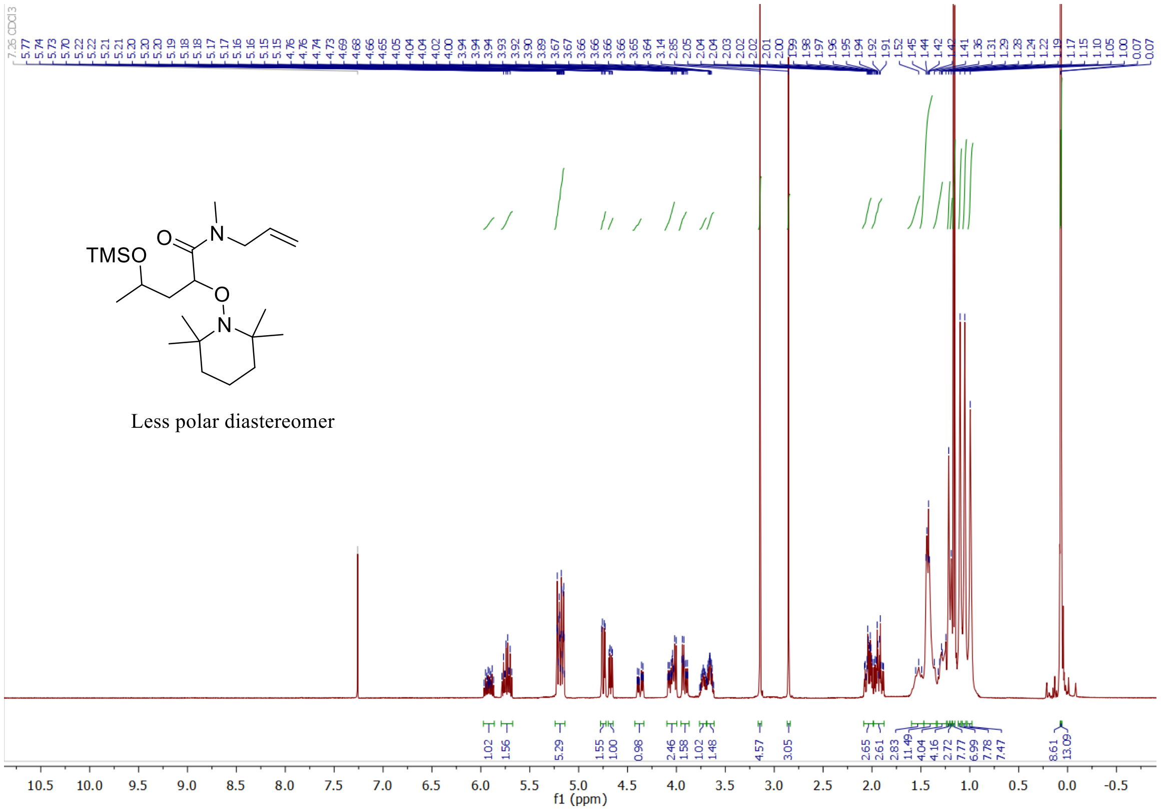

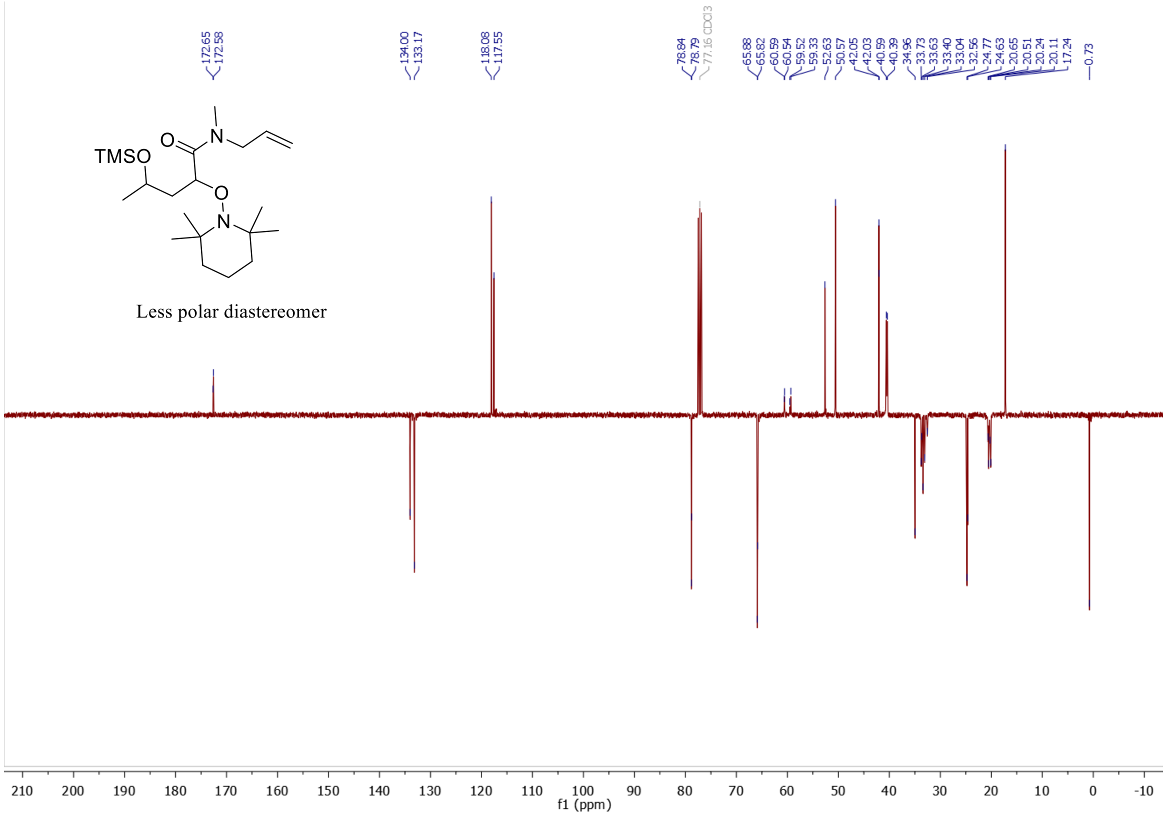

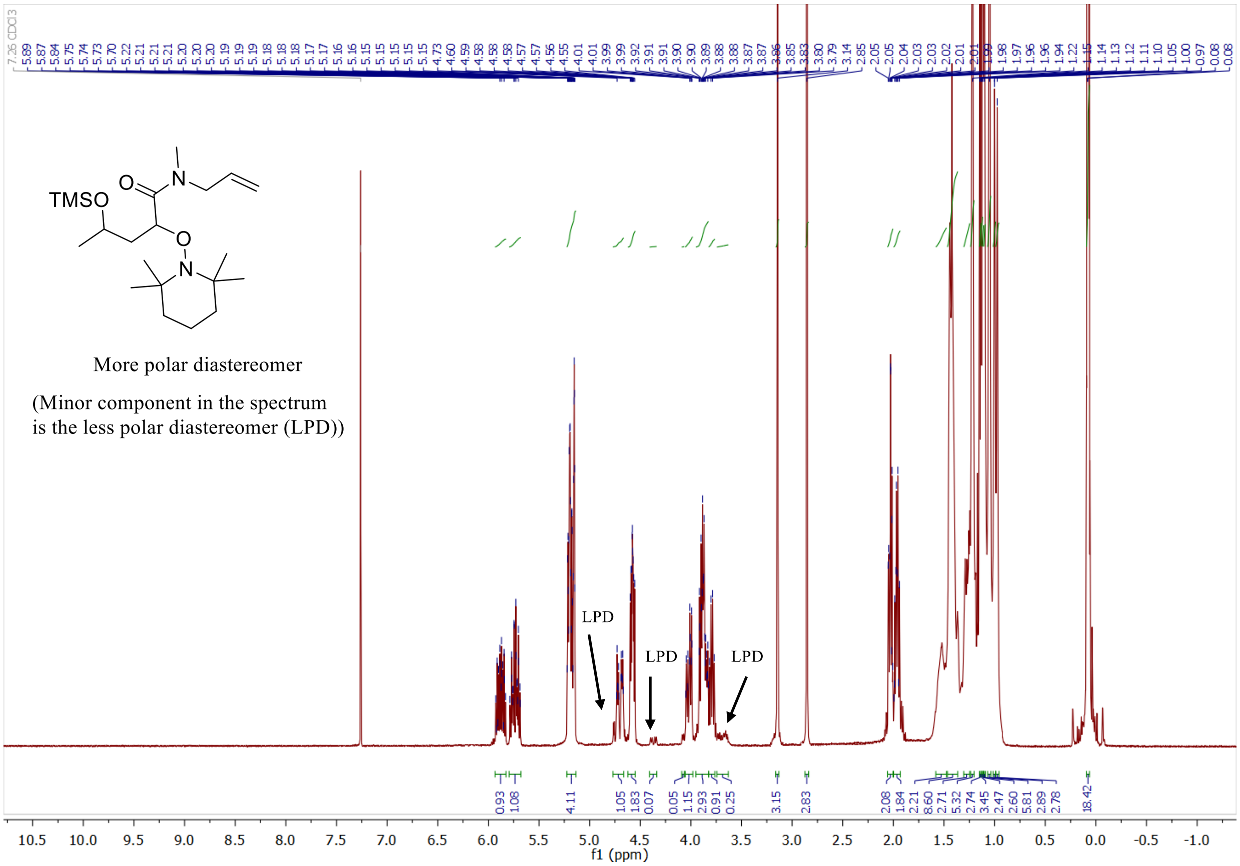
TMSO
O
N
O
N
More polar diastereomer
(Minor component in the spectrum
is the less polar diastereomer (LPD))
LPD
LPD
LPD
7.26 CDCl3
f1 (ppm)
10.5 10.0 9.5 9.0 8.5 8.0 7.5 7.0 6.5 6.0 5.5 5.0 4.5 4.0 3.5 3.0 2.5 2.0 1.5 1.0 0.5 0.0 -0.5 -1.0

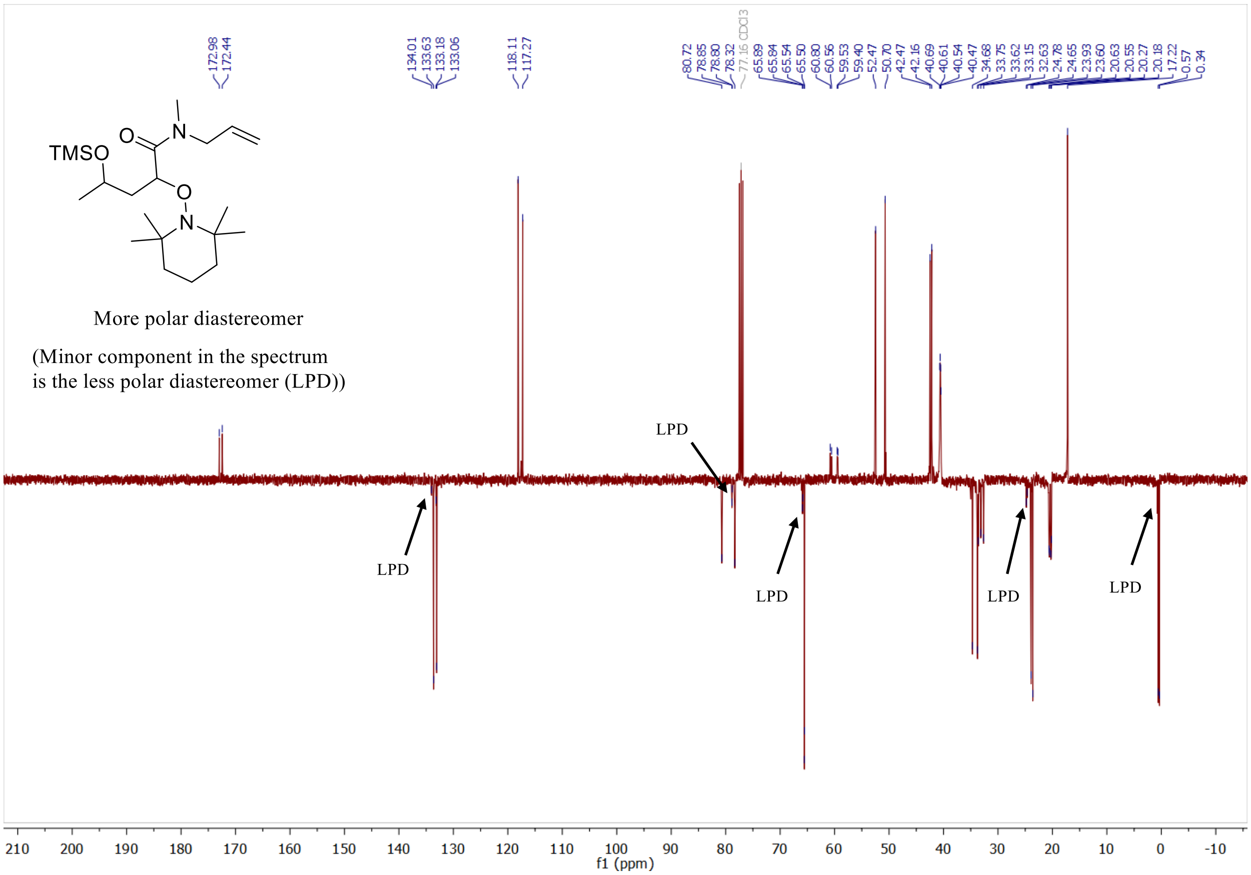
172.98
172.44
134.01
133.63
133.18
133.06
118.11
117.27
80.72
78.85
78.80
78.32
77.16 CDCl3
65.89
65.84
65.54
65.50
60.80
60.56
59.53
59.40
52.47
50.70
42.47
42.16
40.69
40.61
40.54
40.47
34.68
33.75
33.62
33.15
32.63
24.78
24.65
23.93
23.60
20.63
20.55
20.27
20.18
17.22
0.57
0.34
TMSO
O
N
O
N
More polar diastereomer
(Minor component in the spectrum
is the less polar diastereomer (LPD))
LPD
LPD
LPD
LPD
LPD
f1 (ppm)
210 200 190 180 170 160 150 140 130 120 110 100 90 80 70 60 50 40 30 20 10 0 -10

_N_-Benzyl-_N_-(2-methylallyl)-2-((2,2,6,6-tetramethylpiperidin-1-yl)oxy)-4-((trimethylsilyl)oxy)pentanamide (9f)

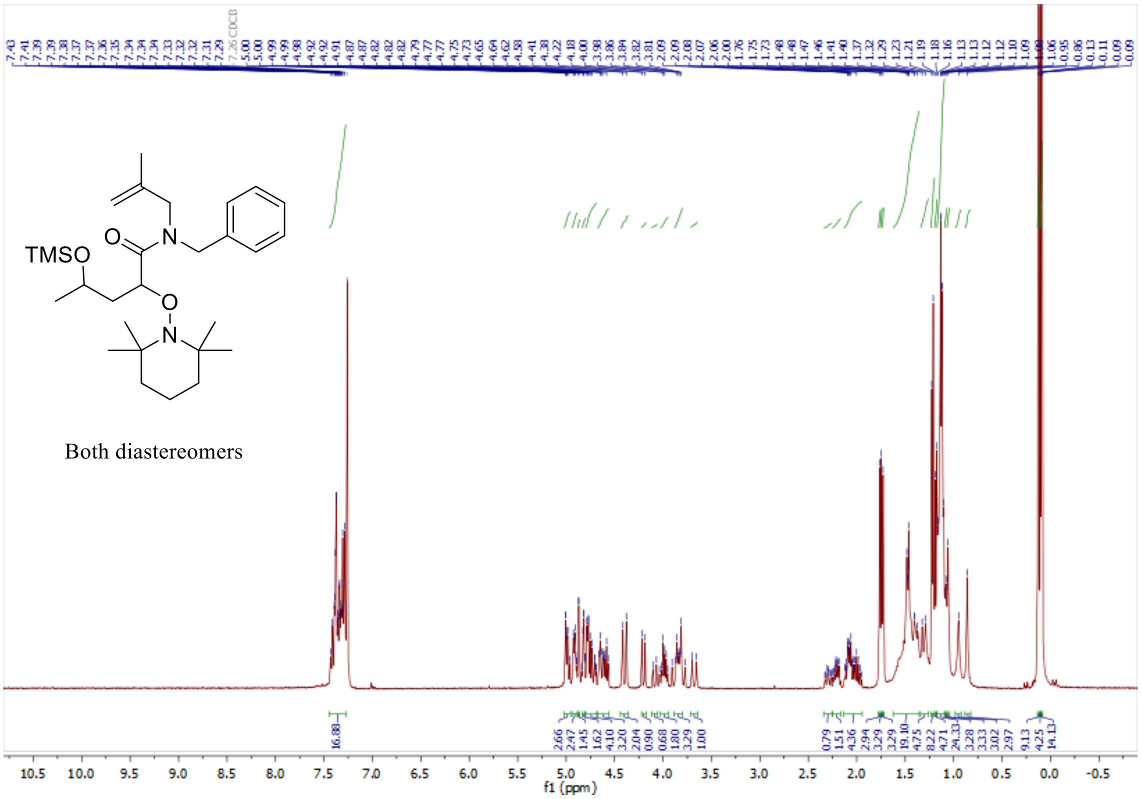

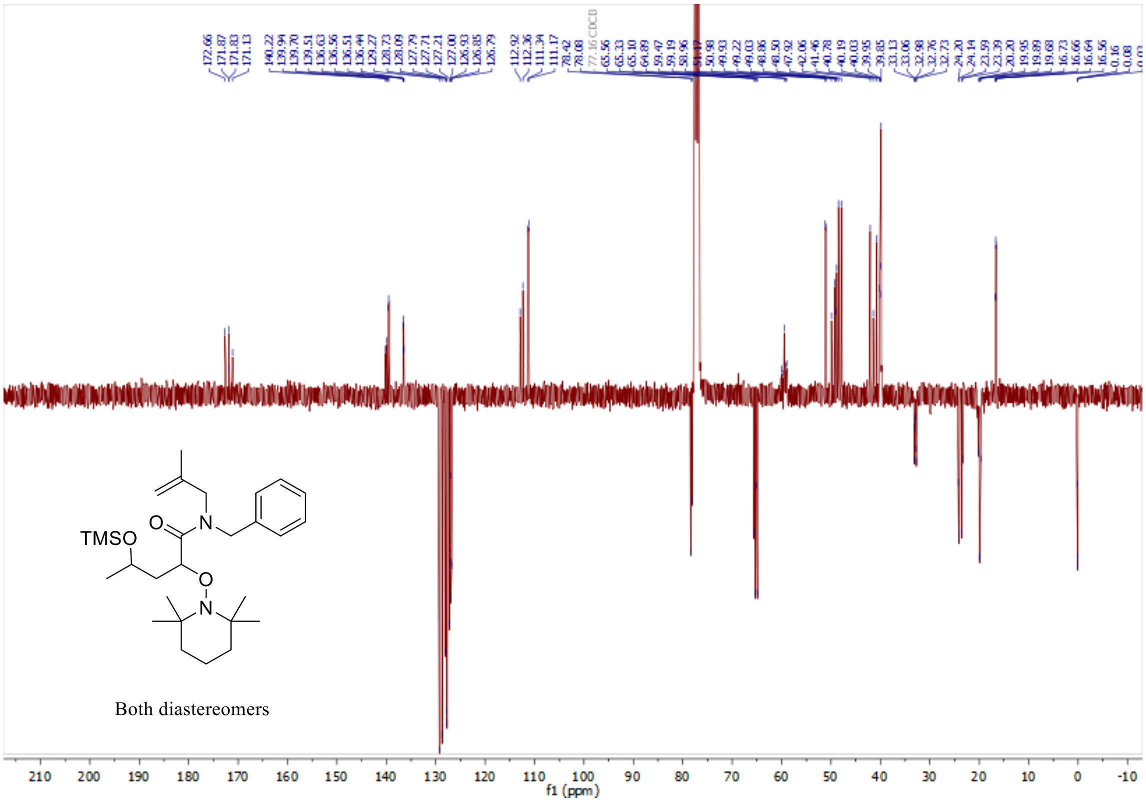

N-Benzyl-*N*-(3-methylbut-2-en-1-yl)-2-((2,2,6,6-tetramethylpiperidin-1-yl)oxy)-4-((trimethylsilyl)oxy)pentanamide (9g)

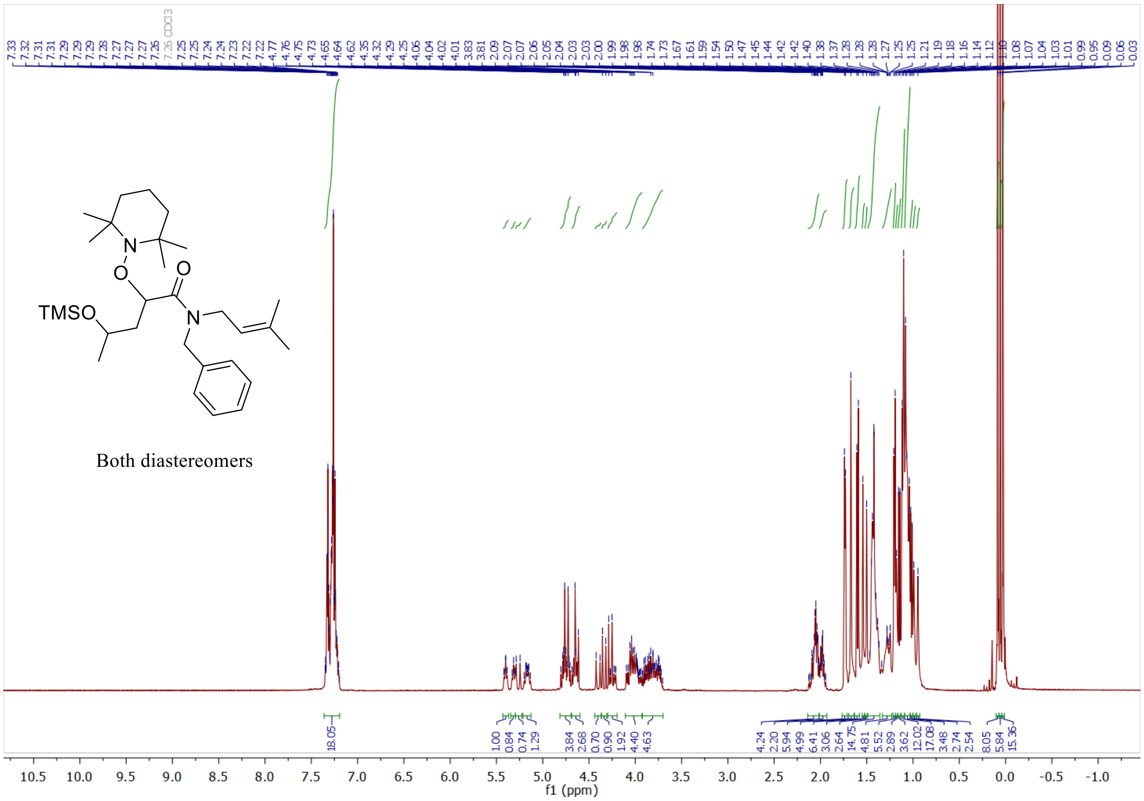

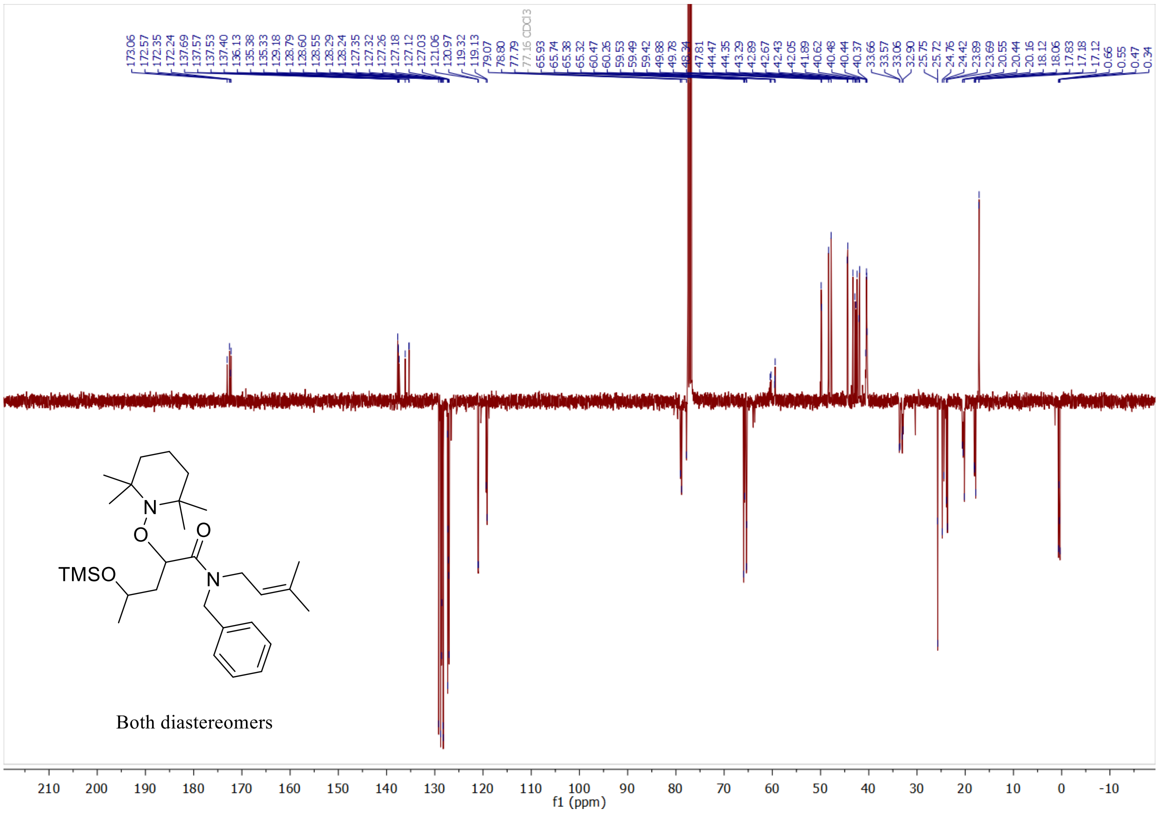

(4*S*)-*N*,*N*-Diallyl-5-(benzyloxy)-2-((2,2,6,6-tetramethylpiperidin-1-yl)oxy)-4-((trimethylsilyl)oxy)pentanamide (9h)

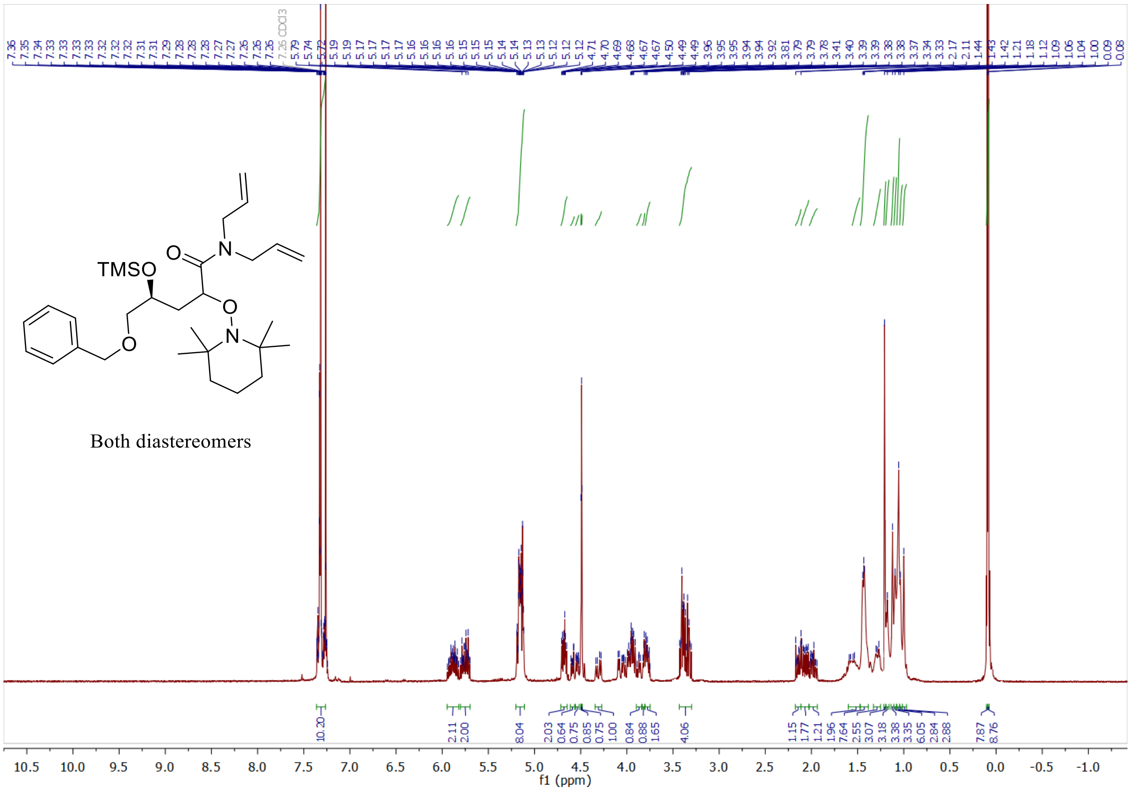

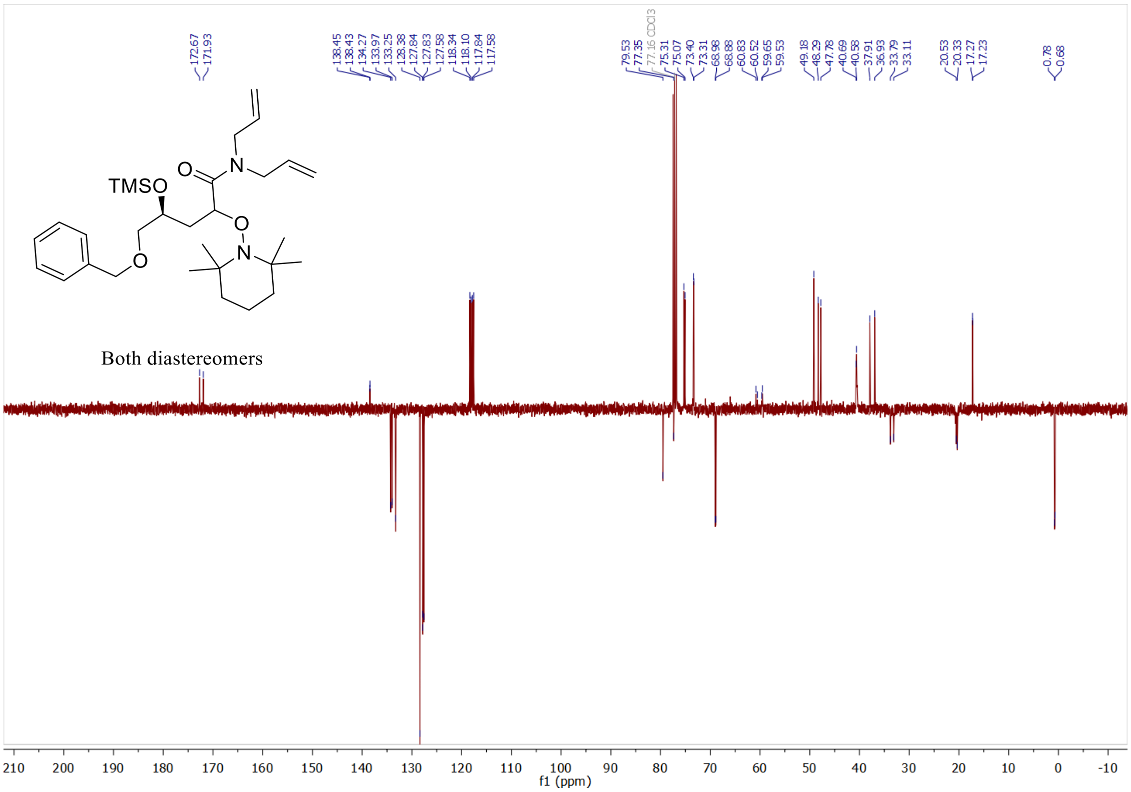

(2*R*,4*S*)- and (2*S*,4*R*)- and (2*S*,4*S*)- and (2*R*,4*R*)-*N*-Allyl-*N*-((*S*)-1-phenylethyl)-2-((2,2,6,6-tetramethylpiperidin-1-yl)oxy)-4-((trimethylsilyl)oxy)pentanamide (9i)

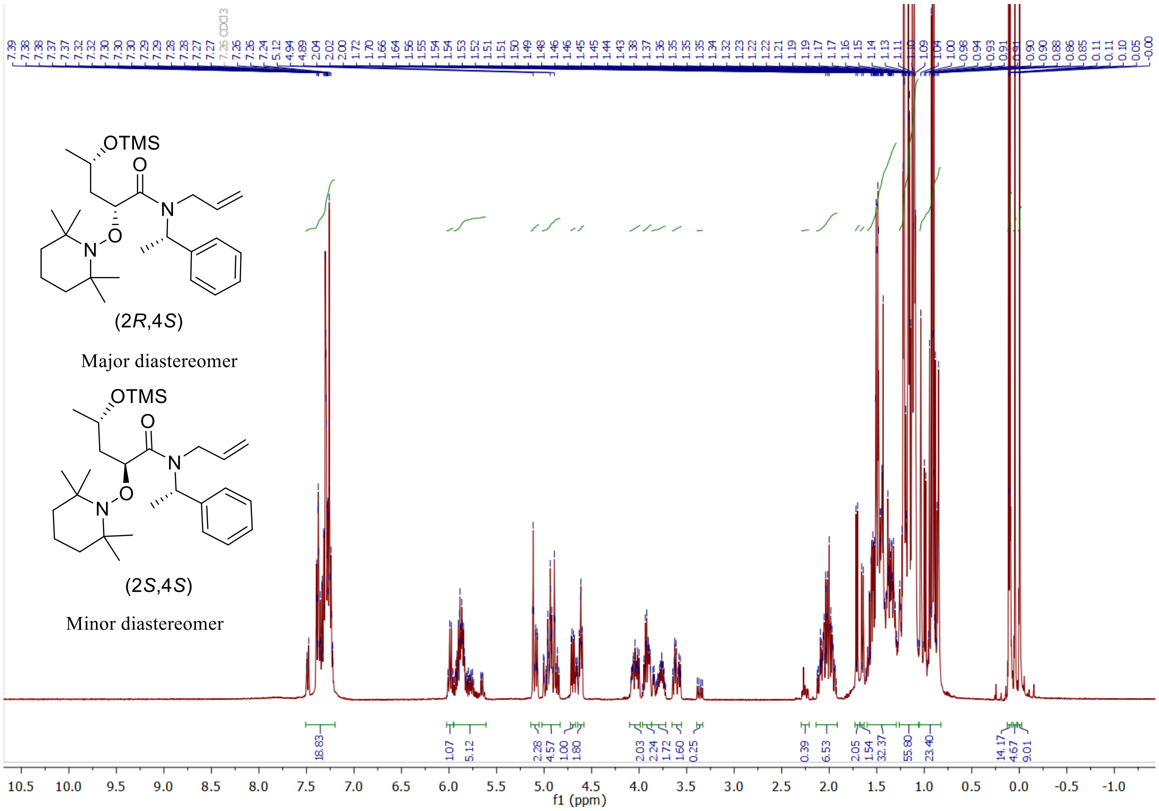

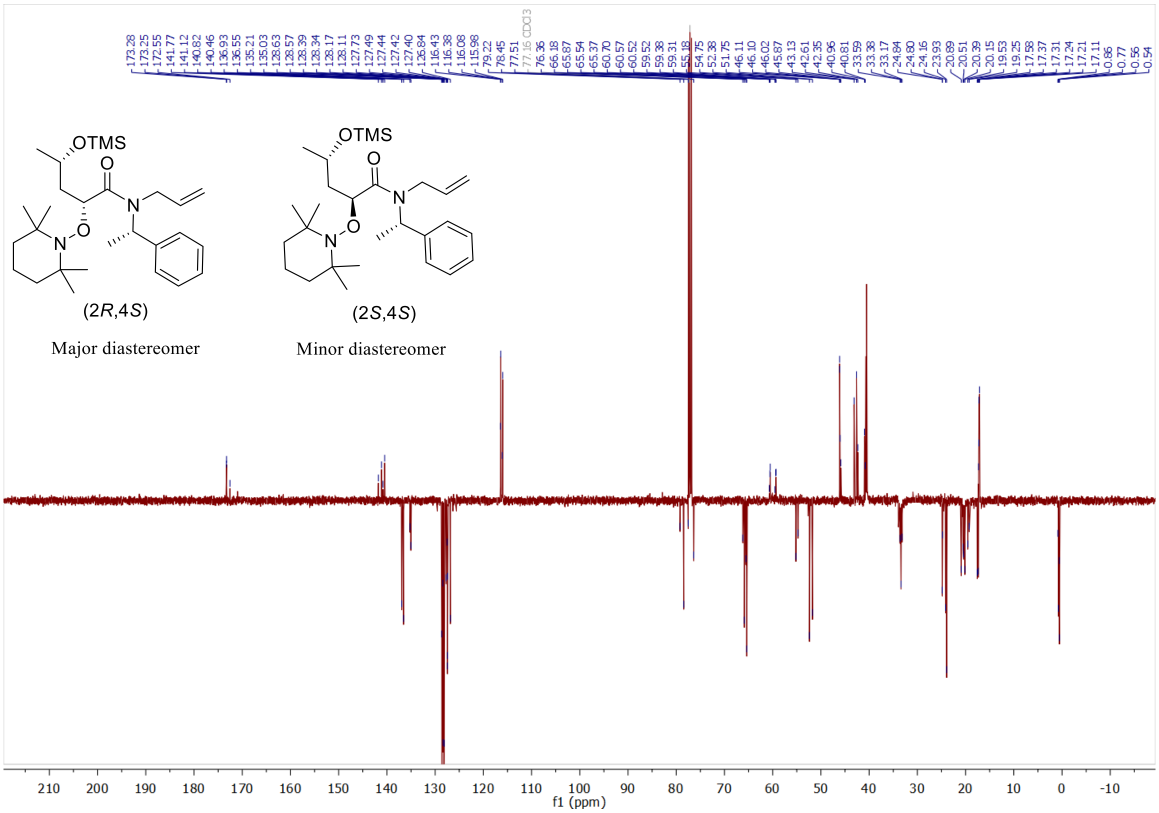

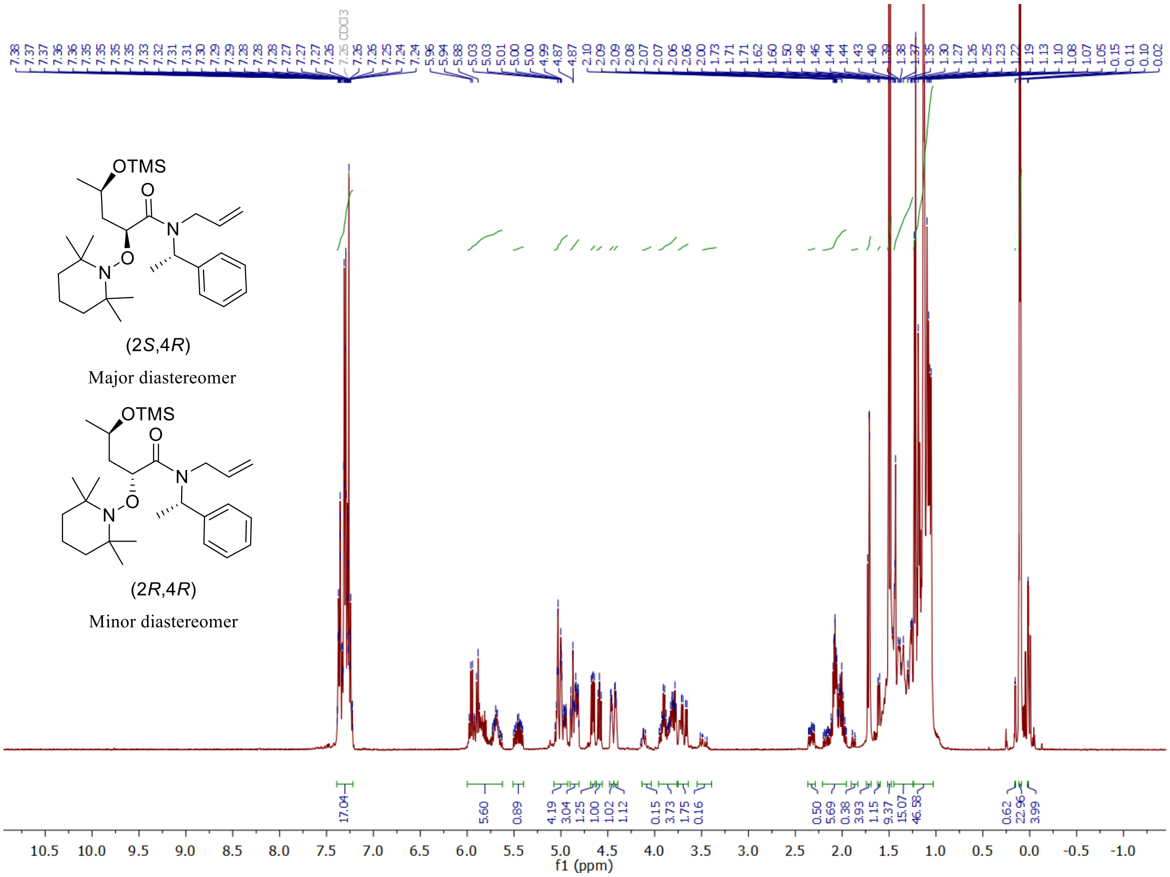
7.38 7.37 7.37 7.36 7.36 7.35 7.35 7.35 7.33 7.32 7.31 7.31 7.30 7.29 7.29 7.28 7.28 7.28 7.27 7.27 7.26 7.26 CDCl3 7.26 7.25 7.25 7.24 7.24 5.96 5.94 5.88 5.03 5.03 5.01 5.00 5.00 4.99 4.87 4.87 2.10 2.09 2.09 2.08 2.07 2.07 2.06 2.06 2.00 1.73 1.71 1.71 1.62 1.60 1.50 1.49 1.46 1.44 1.44 1.43 1.40 1.39 1.38 1.37 1.35 1.30 1.27 1.26 1.25 1.23 1.22 1.19 1.13 1.10 1.08 1.07 1.05 0.15 0.11 0.10 0.02
OTMS
O
N
O
N
(2S,4R)
Major diastereomer
OTMS
O
N
O
N
(2R,4R)
Minor diastereomer
17.04
5.60
0.89
4.19
3.04
1.25
1.00
1.02
1.12
0.15
3.73
1.75
0.16
0.50
5.69
0.38
3.93
1.15
9.37
15.07
46.58
0.62
22.96
3.99
10.5 10.0 9.5 9.0 8.5 8.0 7.5 7.0 6.5 6.0 5.5 5.0 4.5 4.0 3.5 3.0 2.5 2.0 1.5 1.0 0.5 0.0 -0.5 -1.0
f1 (ppm)

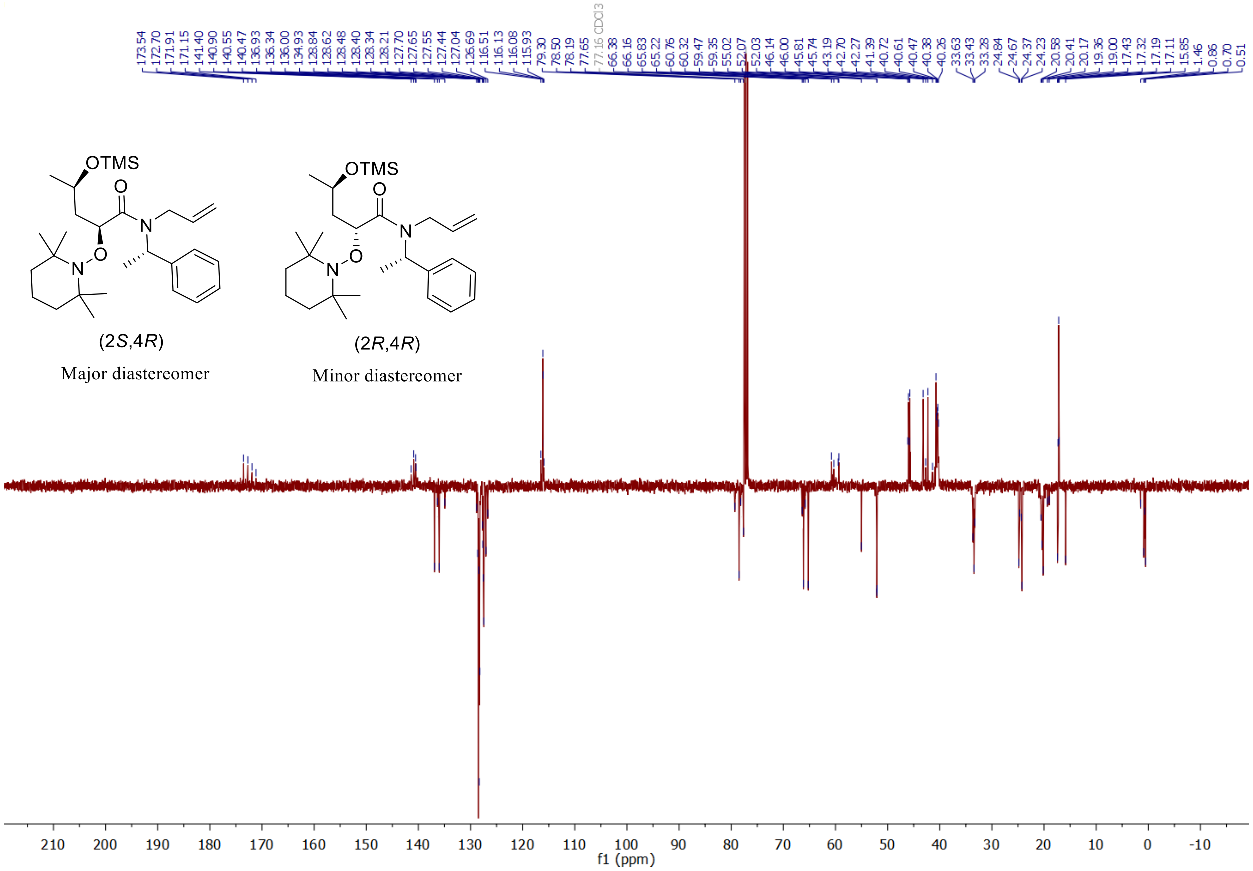
173.54 172.70 171.91 171.15 141.40 140.90 140.55 140.47 136.93 136.34 136.00 134.93 128.84 128.62 128.48 128.40 128.34 128.21 127.70 127.65 127.55 127.44 127.04 126.69 116.51 116.13 116.08 115.93 79.30 78.50 78.19 77.65 77.16 CDCl3 66.38 66.16 65.83 65.22 60.76 60.32 59.47 59.35 55.02 52.07 52.03 46.14 46.00 45.81 45.74 43.19 42.70 42.27 41.39 40.72 40.61 40.47 40.38 40.26 33.63 33.43 33.28 24.84 24.67 24.37 24.23 20.58 20.41 20.17 19.36 19.00 17.43 17.32 17.19 17.11 15.85 1.46 0.86 0.70 0.51
OTMS
O
N
O
N
(2S,4R)
Major diastereomer
OTMS
O
N
O
N
(2R,4R)
Minor diastereomer
210 200 190 180 170 160 150 140 130 120 110 100 90 80 70 60 50 40 30 20 10 0 -10
f1 (ppm)

(2*R*,4*S*)- and (2*S*,4*S*)-*N*-Allyl-*N*-((*S*)-1-(naphthalen-2-yl)ethyl)-2-((2,2,6,6-tetramethyl-piperidin-1-yl)oxy)-4-((trimethylsilyl)oxy)pentanamide (9j)

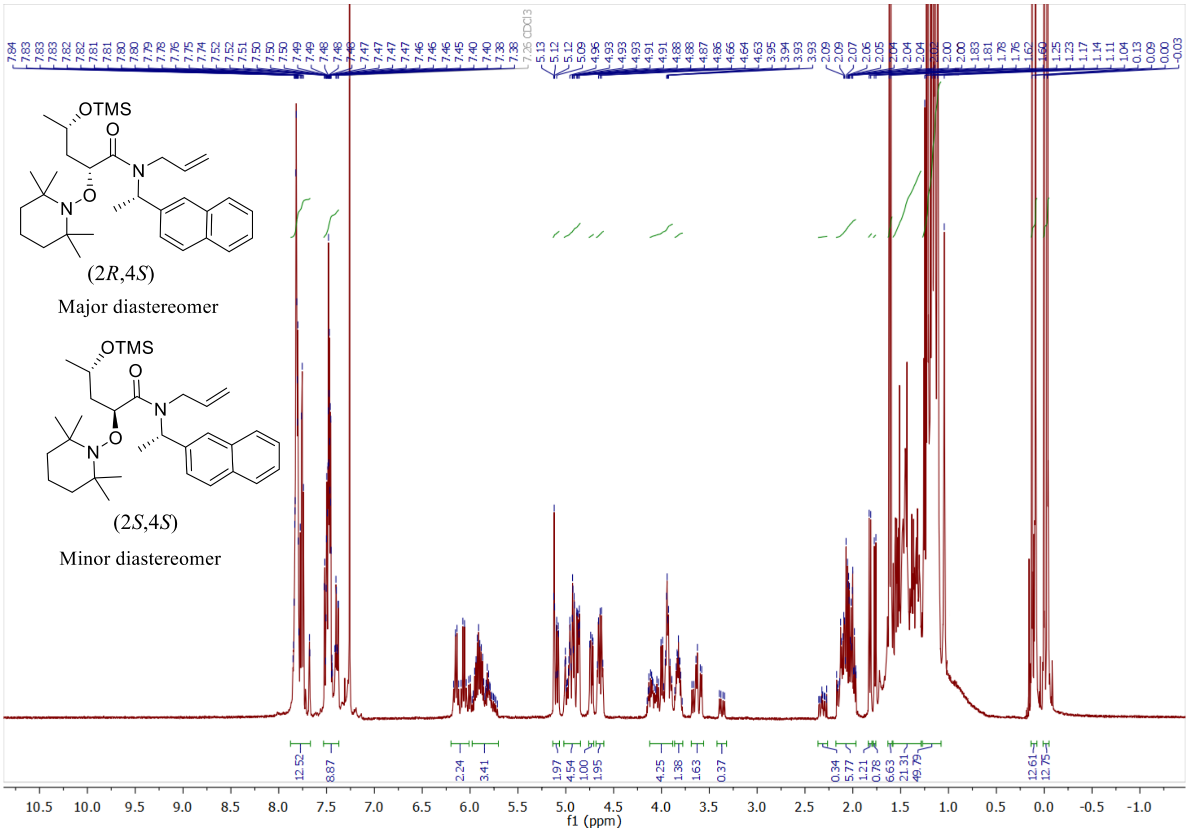

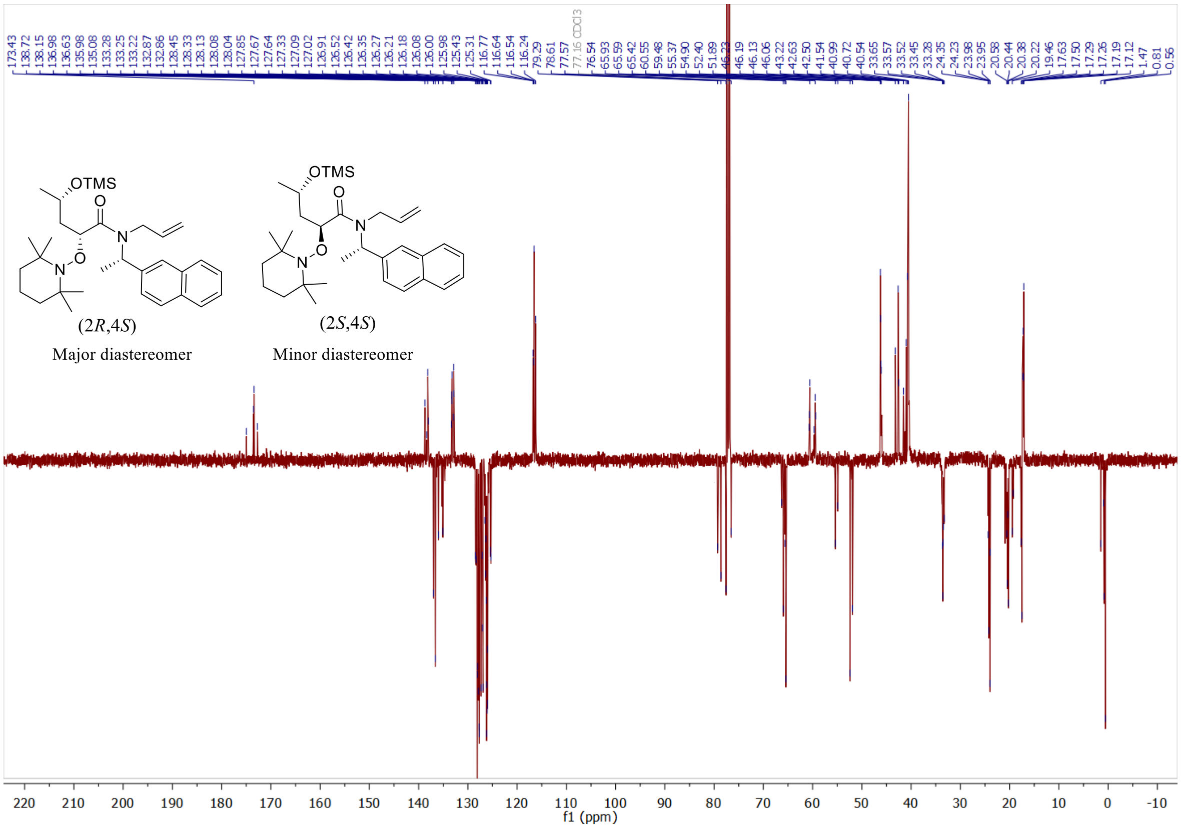

(2*R*,4*S*)- and (2*S*,4*S*)-*N*-Allyl-4-hydroxy-*N*-((*S*)-1-(naphthalen-2-yl)ethyl)-2-((2,2,6,6-tetramethylpiperidin-1-yl)oxy)pentanamide (S13)

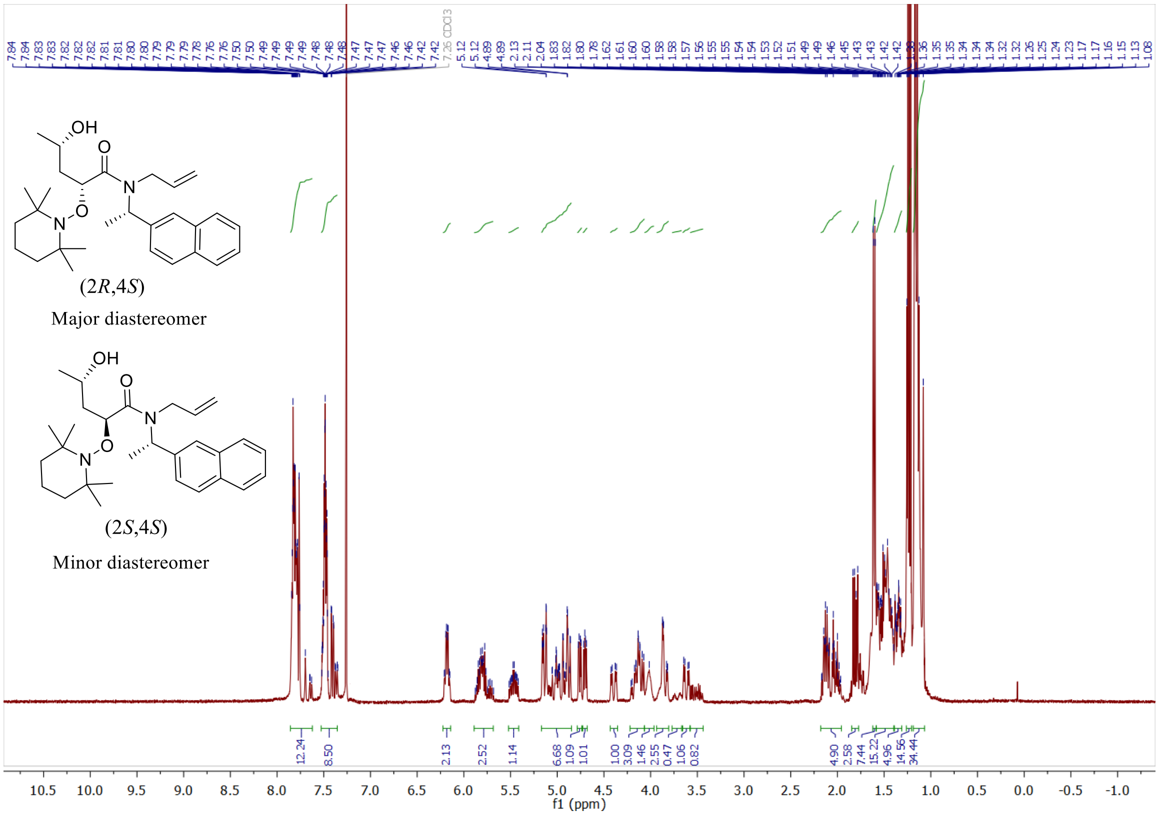

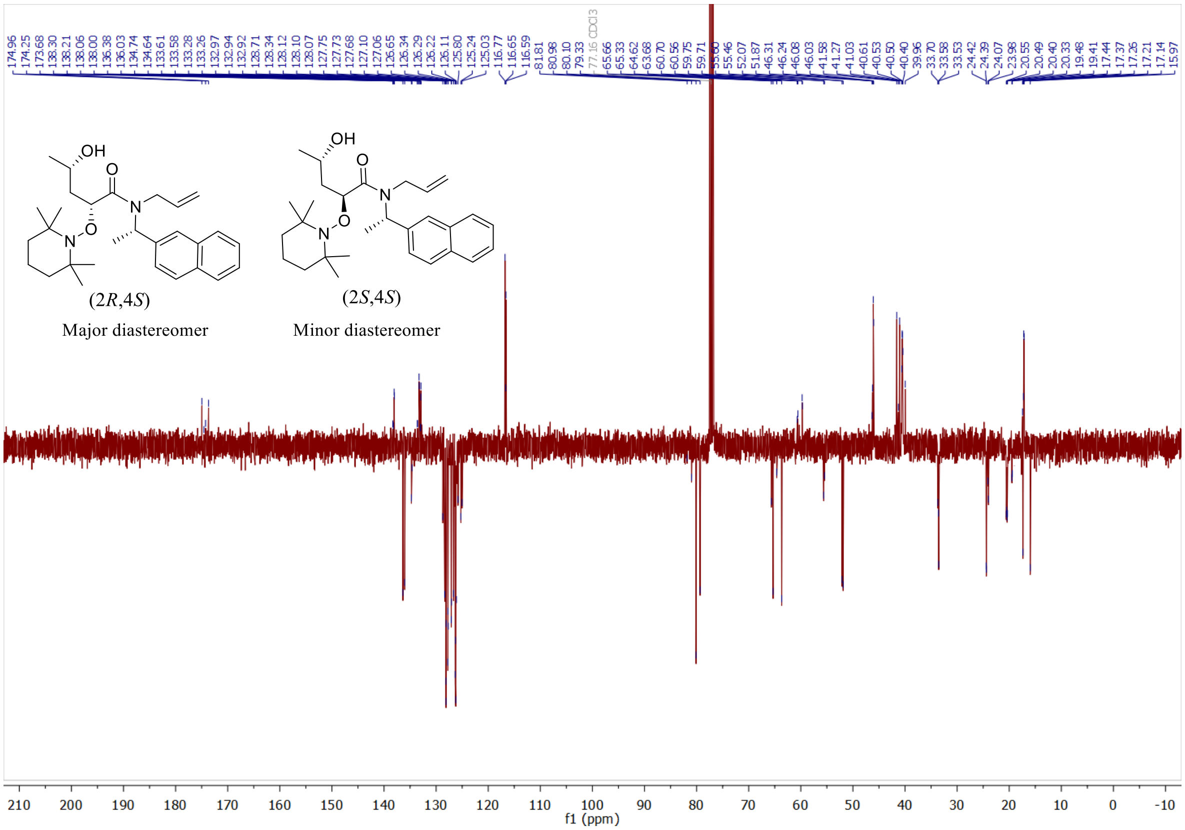

(2*R*,4*S*)- and (2*S*,4*S*)-*N*-(3-Methylbut-2-en-1-yl)-*N*-((*S*)-1-phenylethyl)-2-((2,2,6,6-tetramethylpiperidin-1-yl)oxy)-4-((trimethylsilyl)oxy)pentanamide (9k)

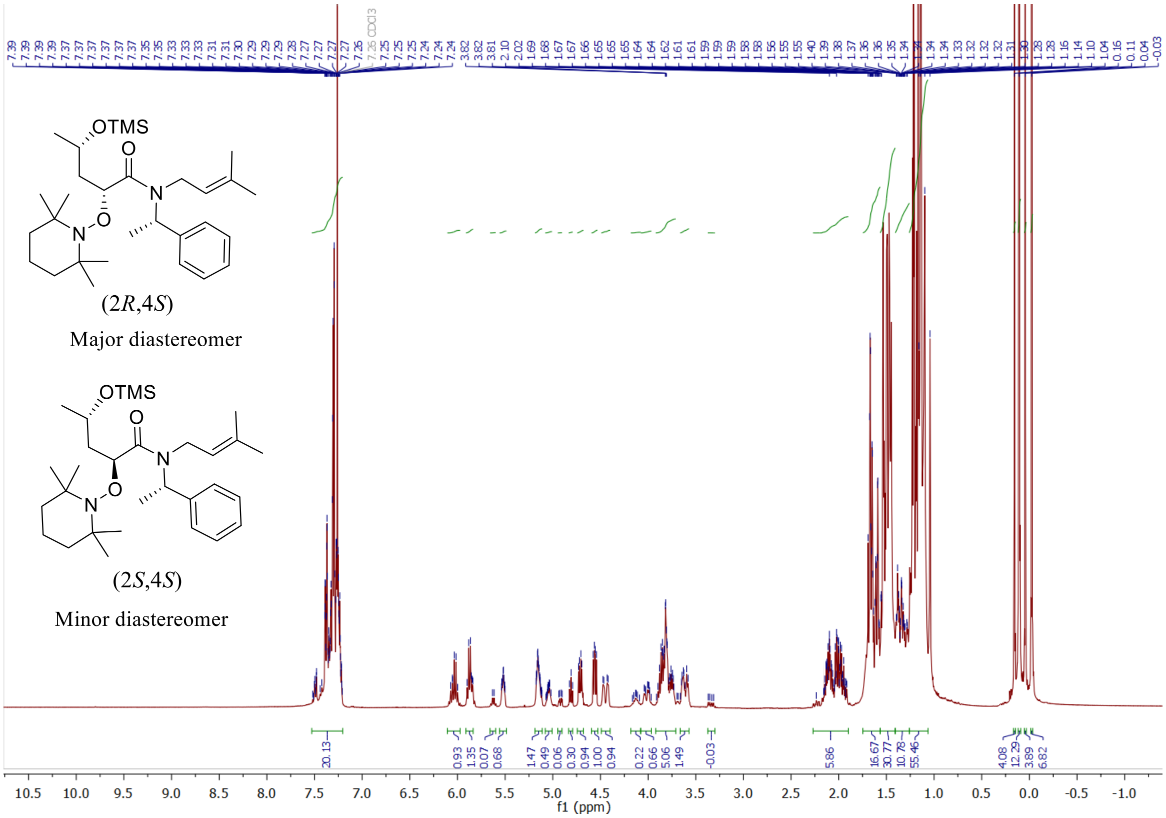

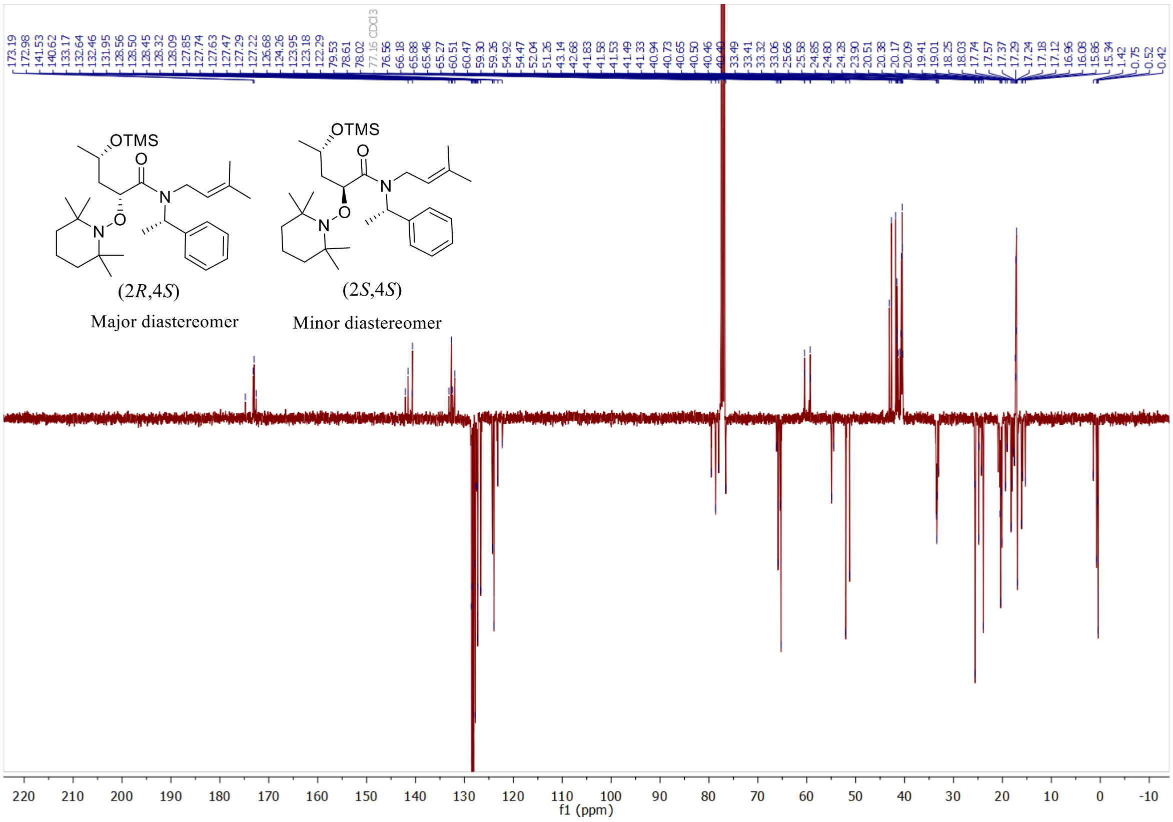

(*R)- and (*S**)-*N*,*N*-Diallyl-2-((2,2,6,6-tetramethylpiperidin-1-yl)oxy)-2-((1*R**,2*S**)-2-((trimethylsilyl)oxy)cyclohexyl)acetamide (9l)**

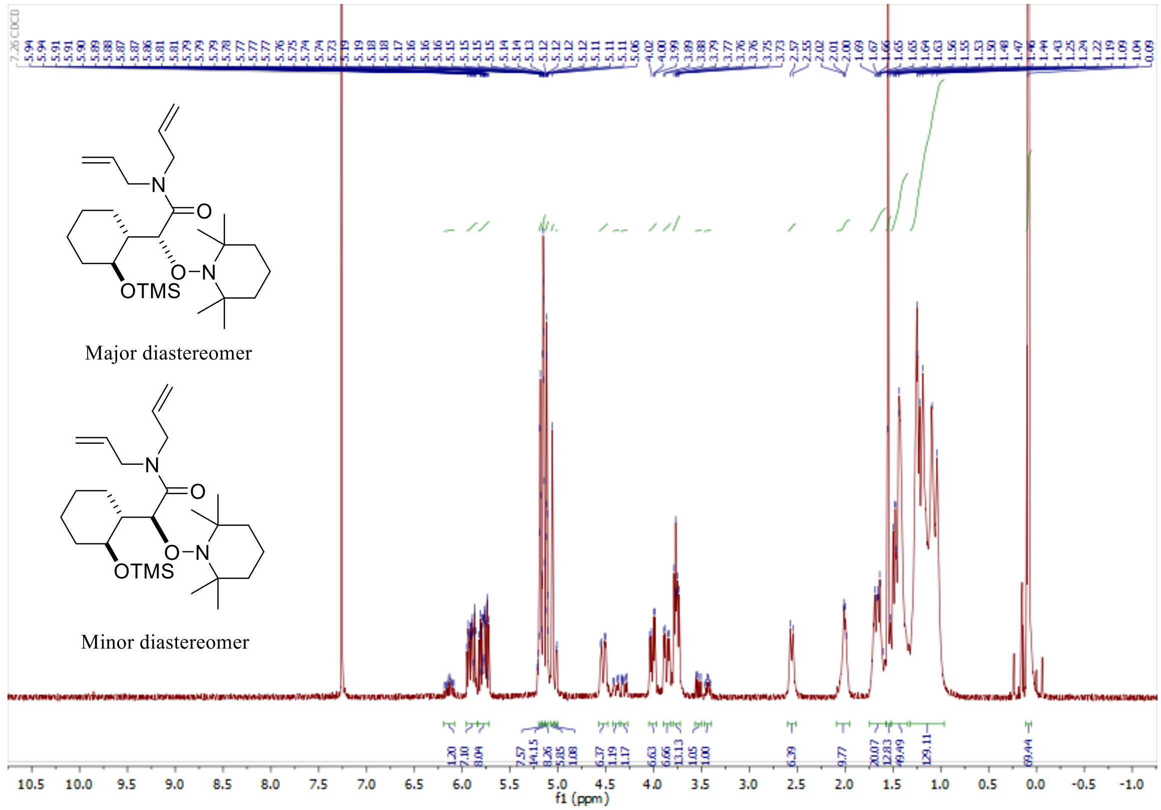

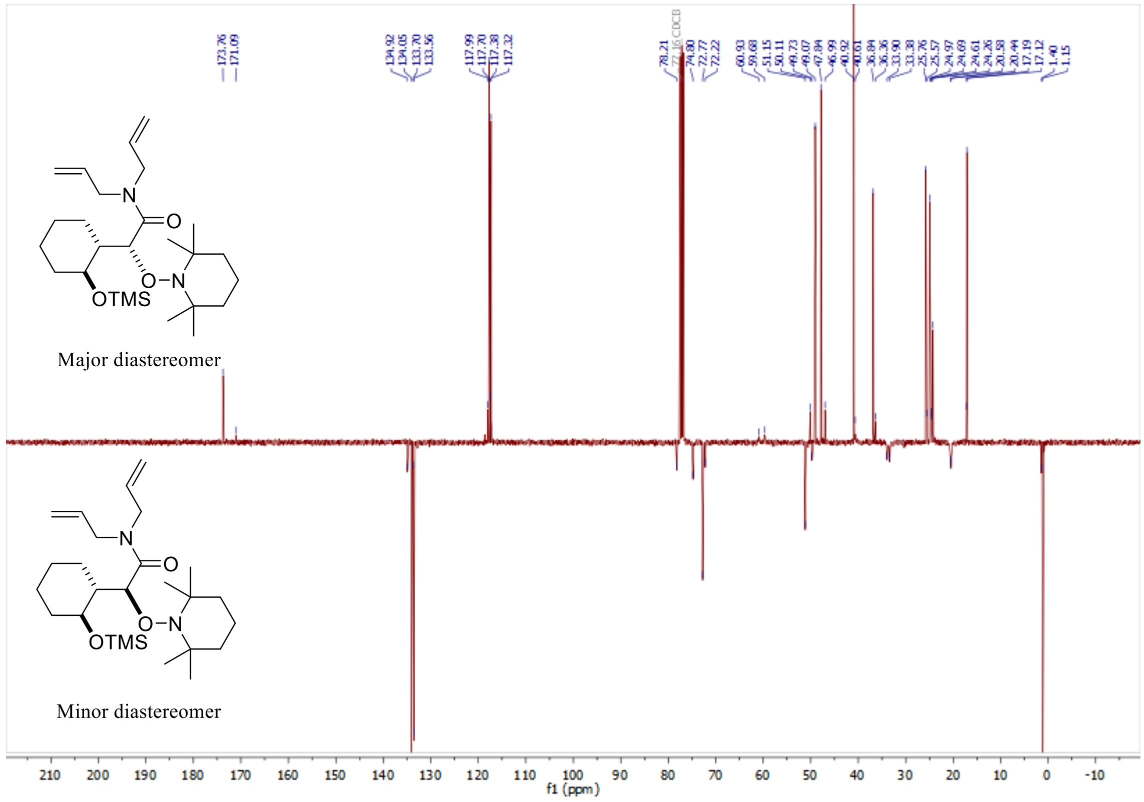

_N,N_-Diallyl-2-((1_R_*,2_S_*)-2-((trimethylsilyl)oxy)cyclohexyl)acetamide (S14)

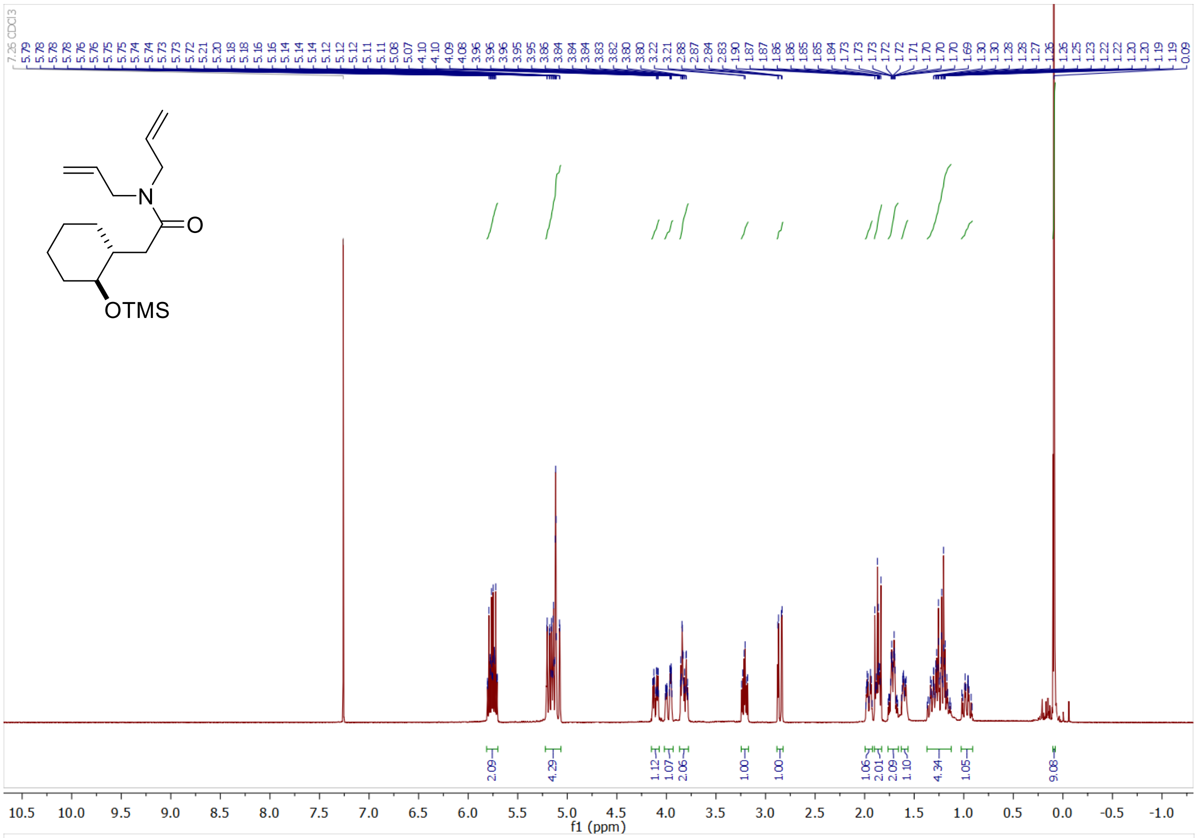

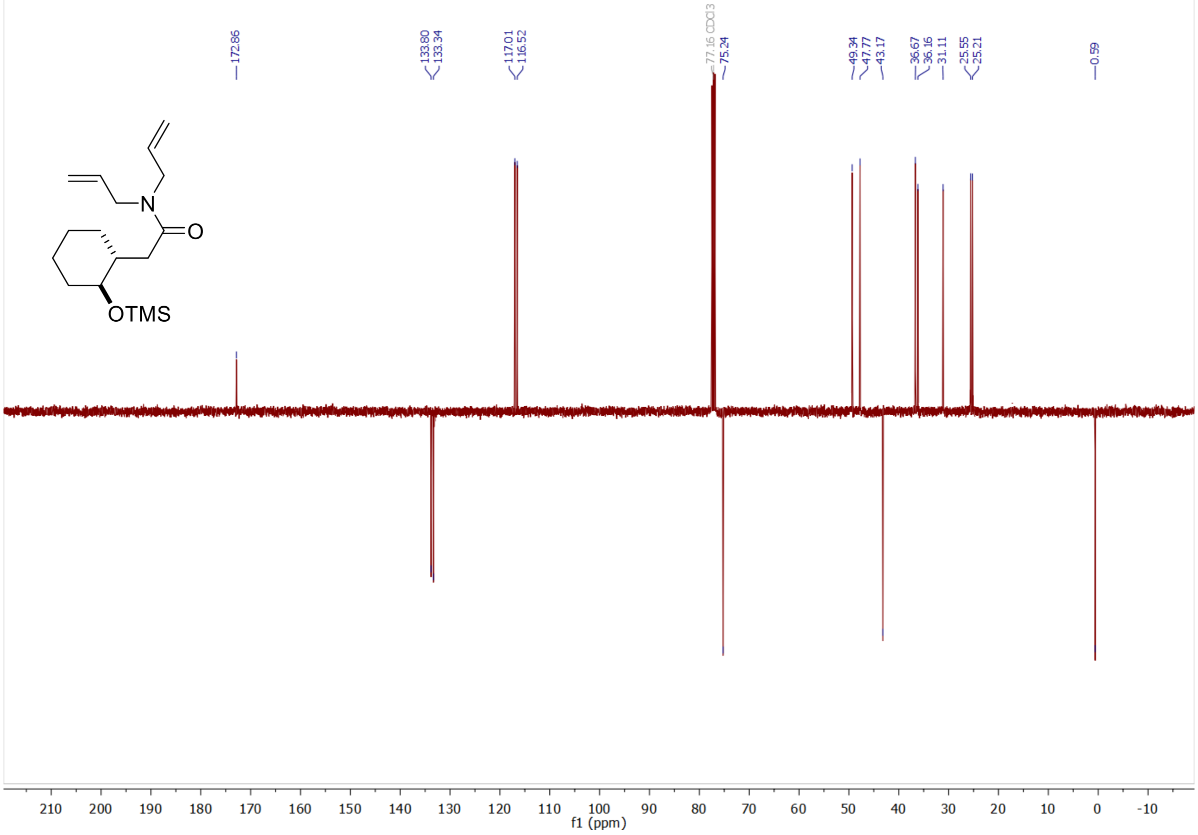

N-Benzyl-N-(cyclopent-1-en-1-ylmethyl)-2-((2,2,6,6-tetramethylpiperidin-1-yl)oxy)-4-((trimethylsilyl)oxy)pentanamide (9m)

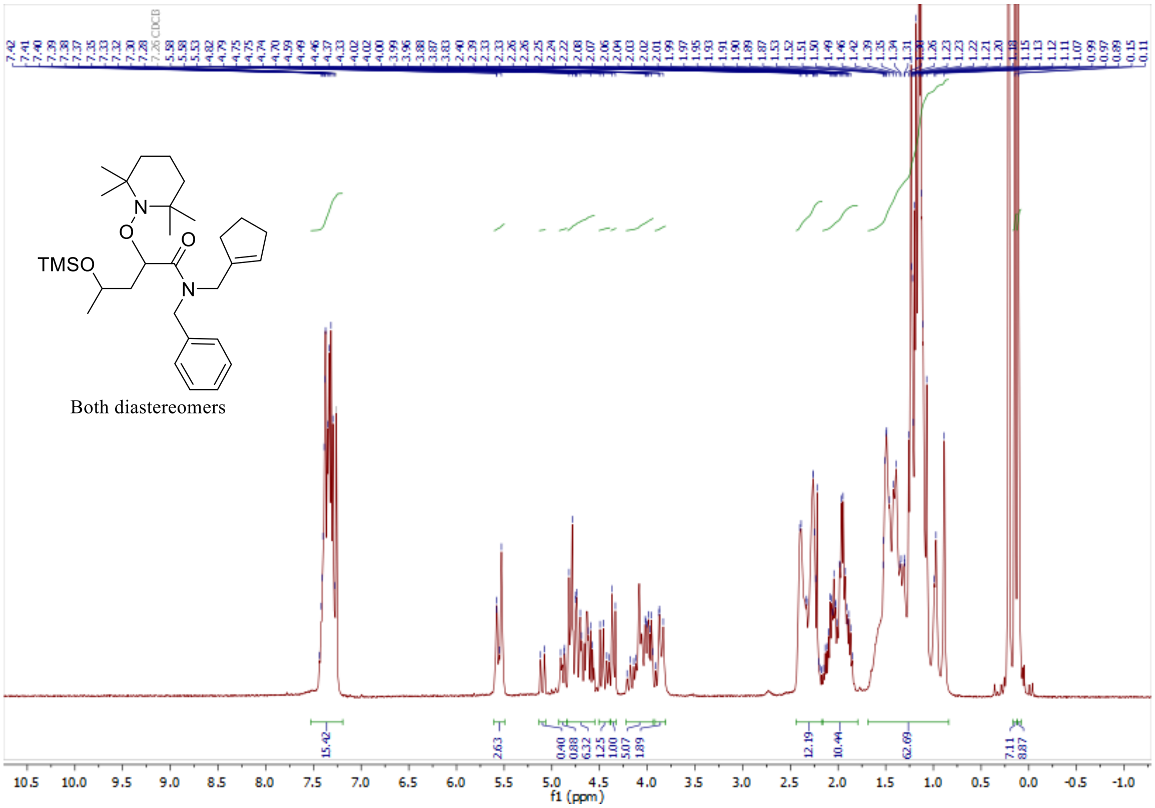

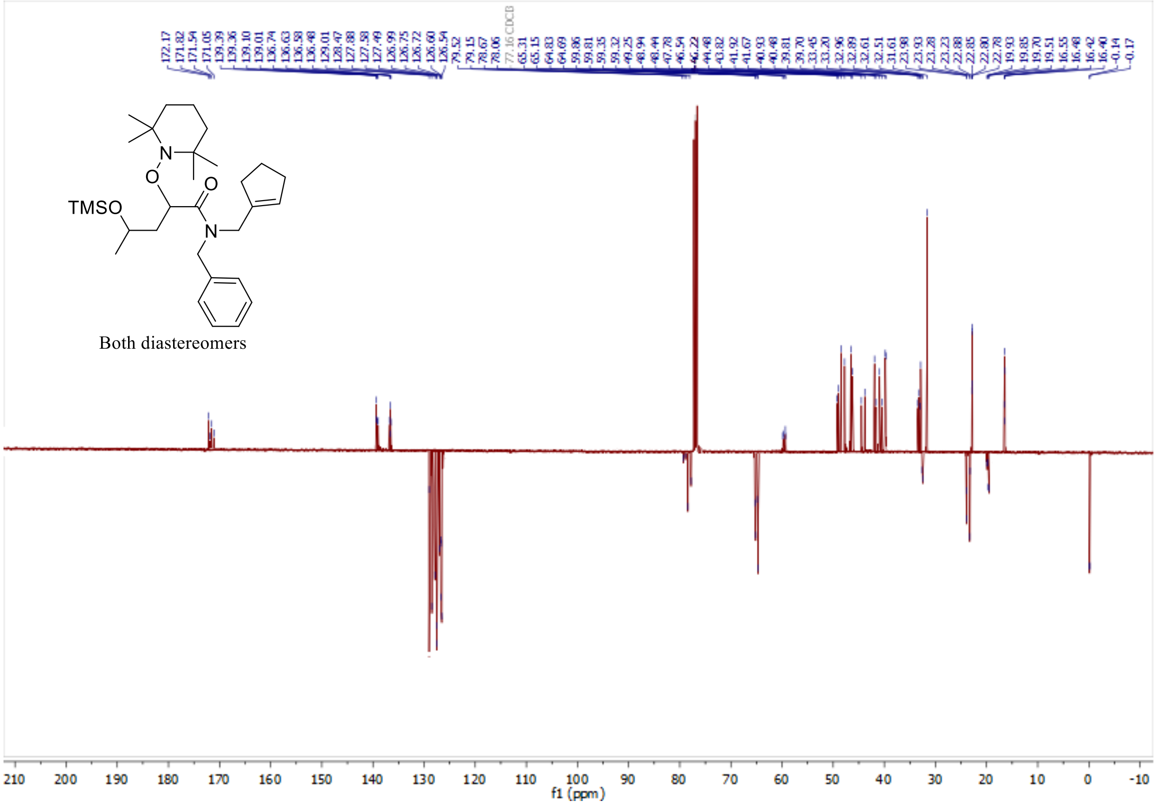

N-Benzyl-*N*-(cyclohex-1-en-1-ylmethyl)-2-((2,2,6,6-tetramethylpiperidin-1-yl)oxy)-4-((trimethylsilyl)oxy)pentanamide (9n)

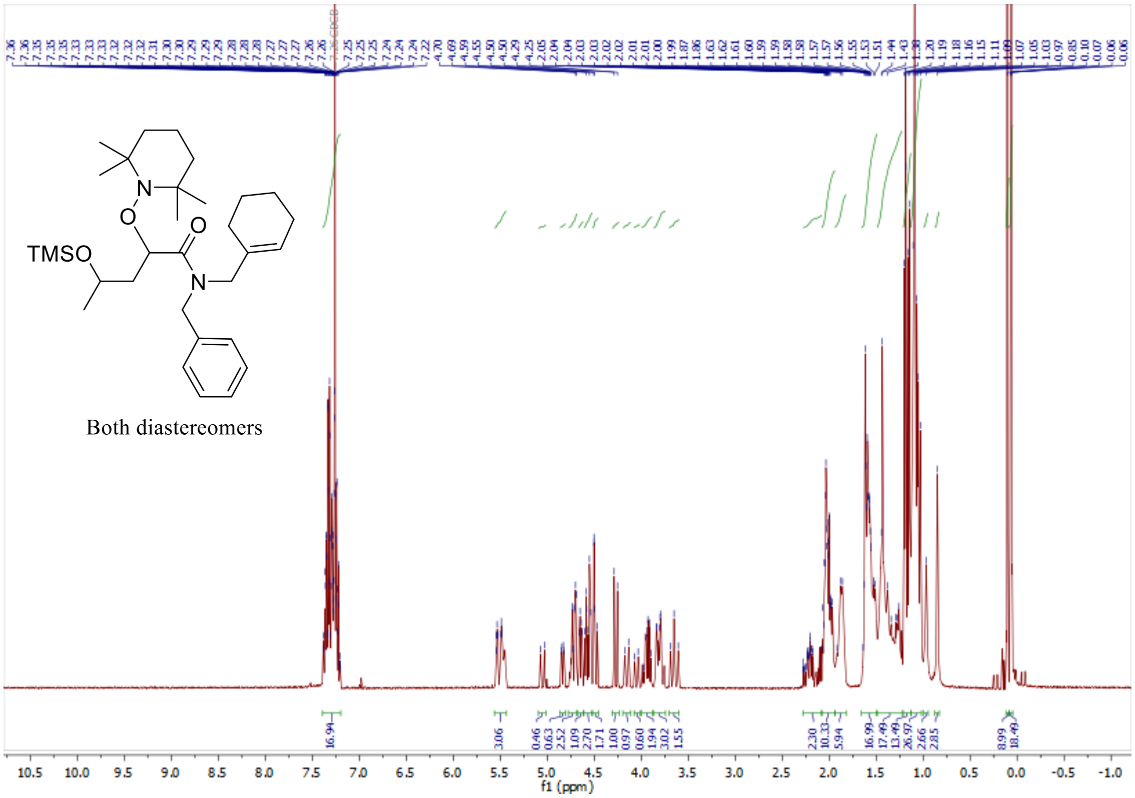

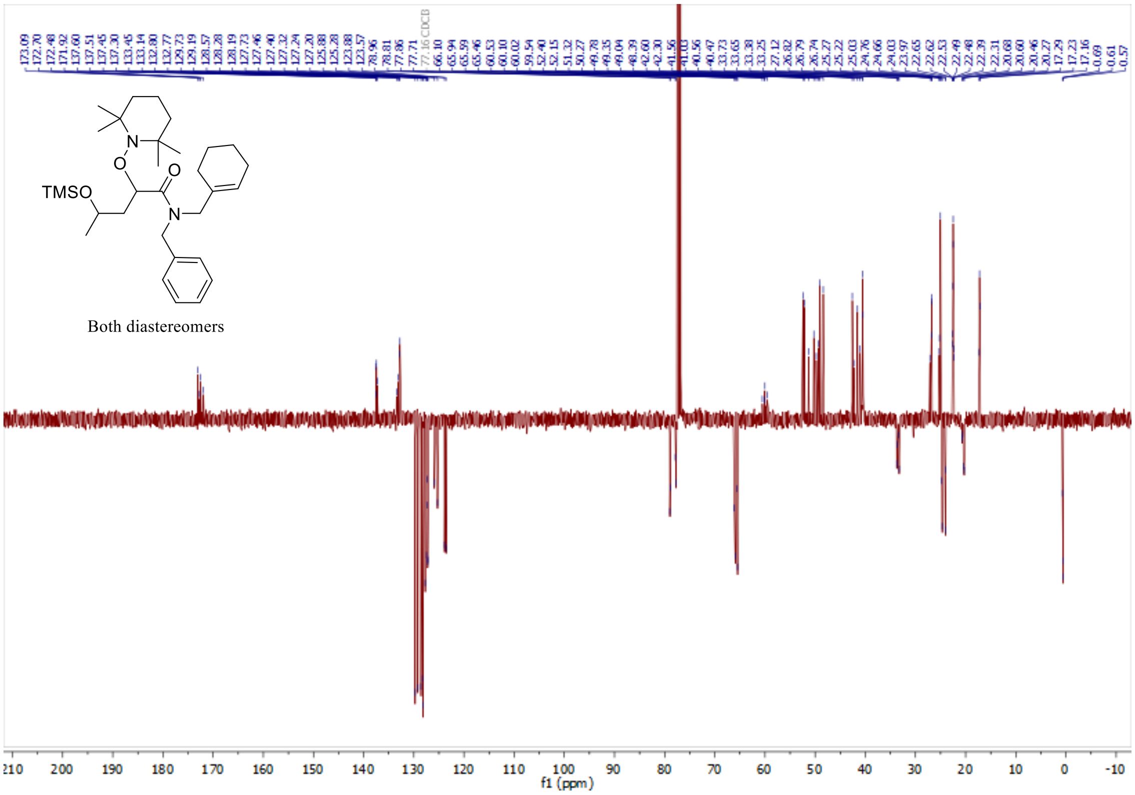

N-Benzyl-*N*-(cyclopent-2-en-1-yl)-2-((2,2,6,6-tetramethylpiperidin-1-yl)oxy)-4-((trimethylsilyl)oxy)pentanamide (9o)

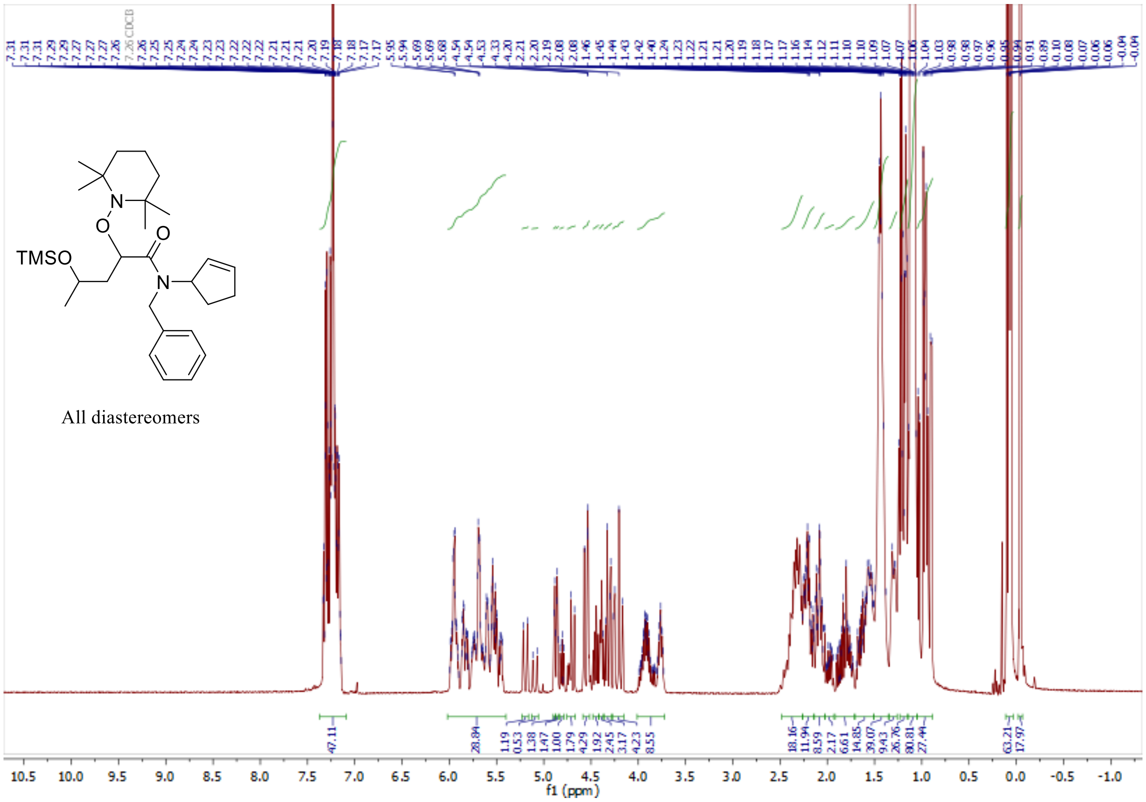

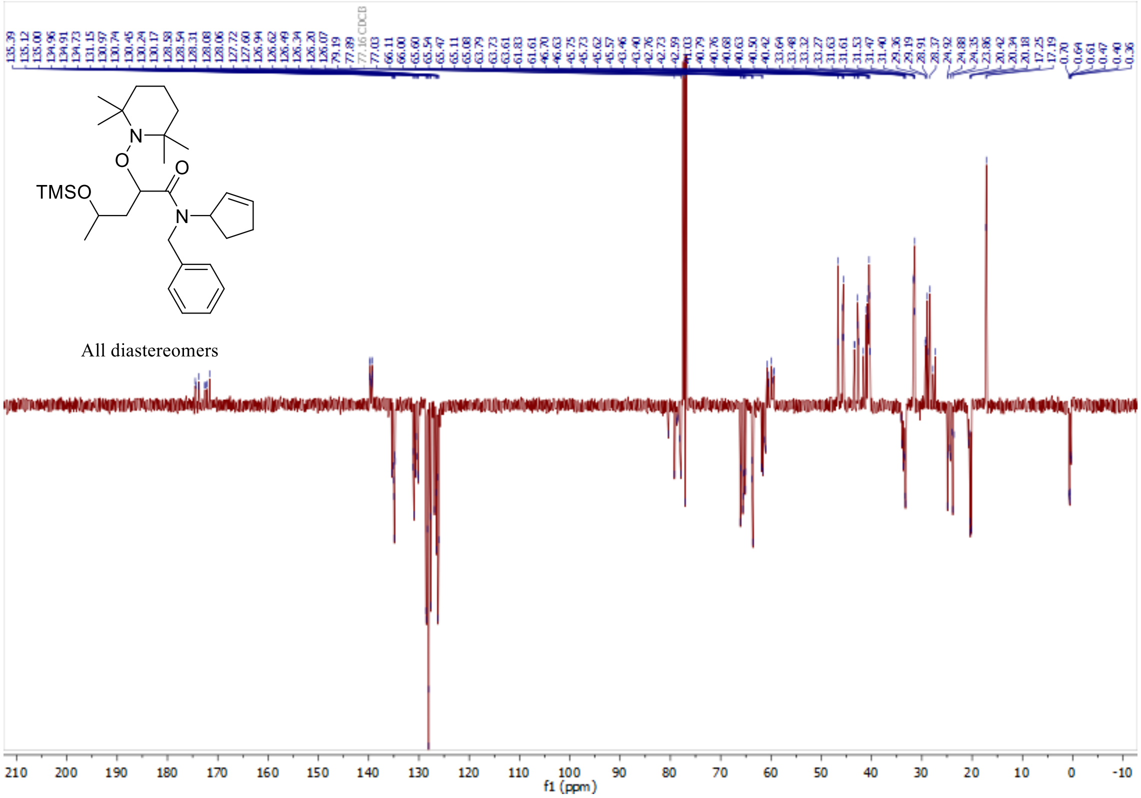

N-Benzyl-N-((R)-cyclohex-2-en-1-yl)-2-((2,2,6,6-tetramethylpiperidin-1-yl)oxy)-4-((trimethylsilyl)oxy)pentanamide (9p)

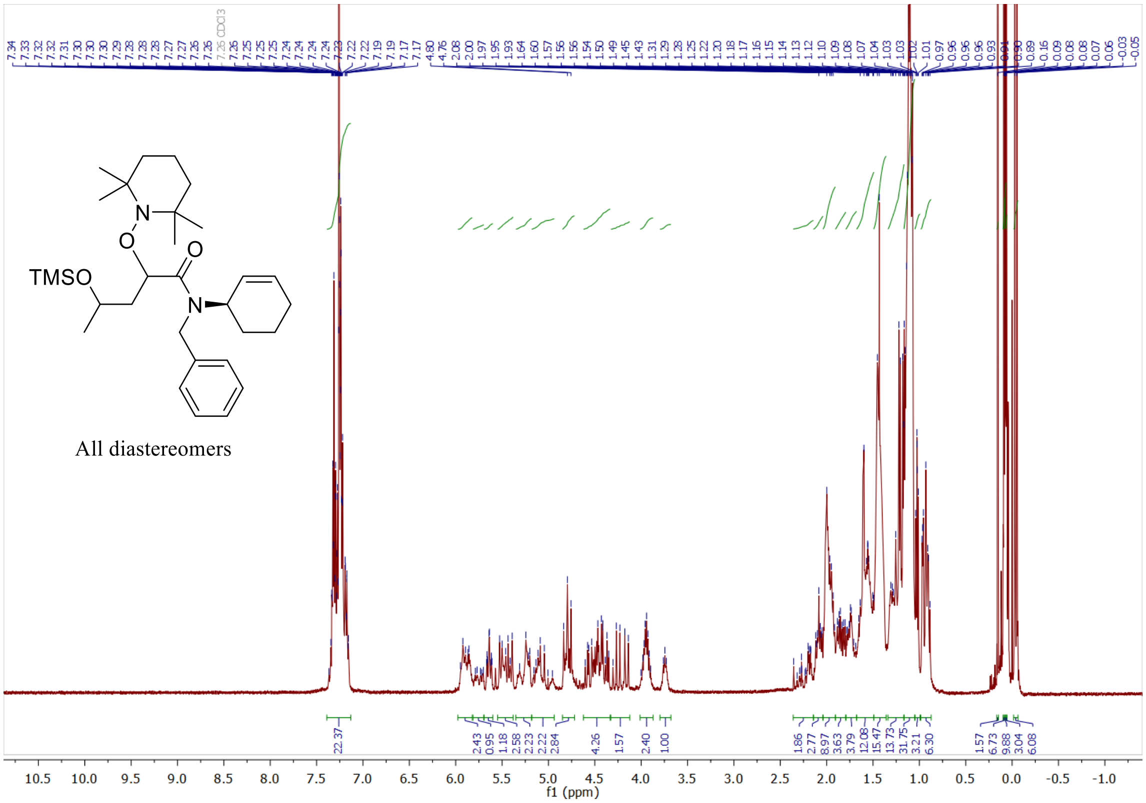

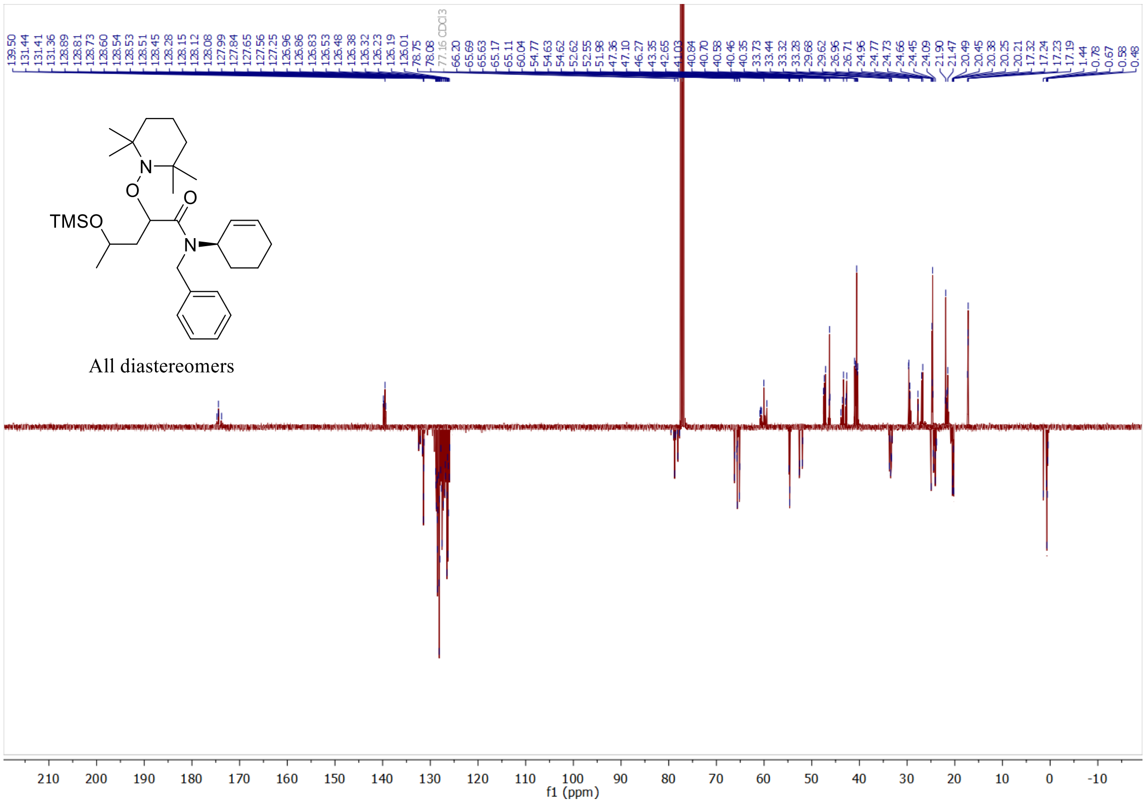

(3*R,4*R**)- and (3*S**,4*R**)-1-Allyl-3-(2-hydroxy-2-methylpropyl)-4-(((2,2,6,6-tetramethyl-piperidin-1-yl)oxy)methyl)pyrrolidin-2-one (12a).** Spectral data for compound *trans*-**12a** are identical, only signals of the major diastereomers are more intense.

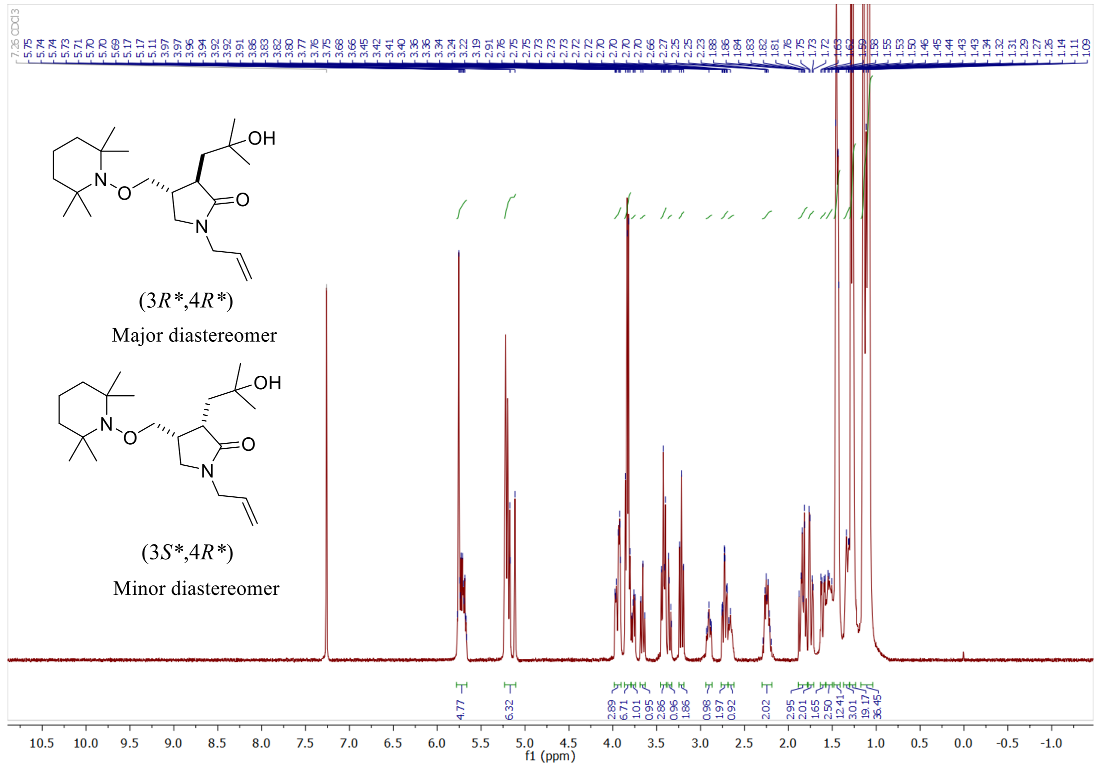

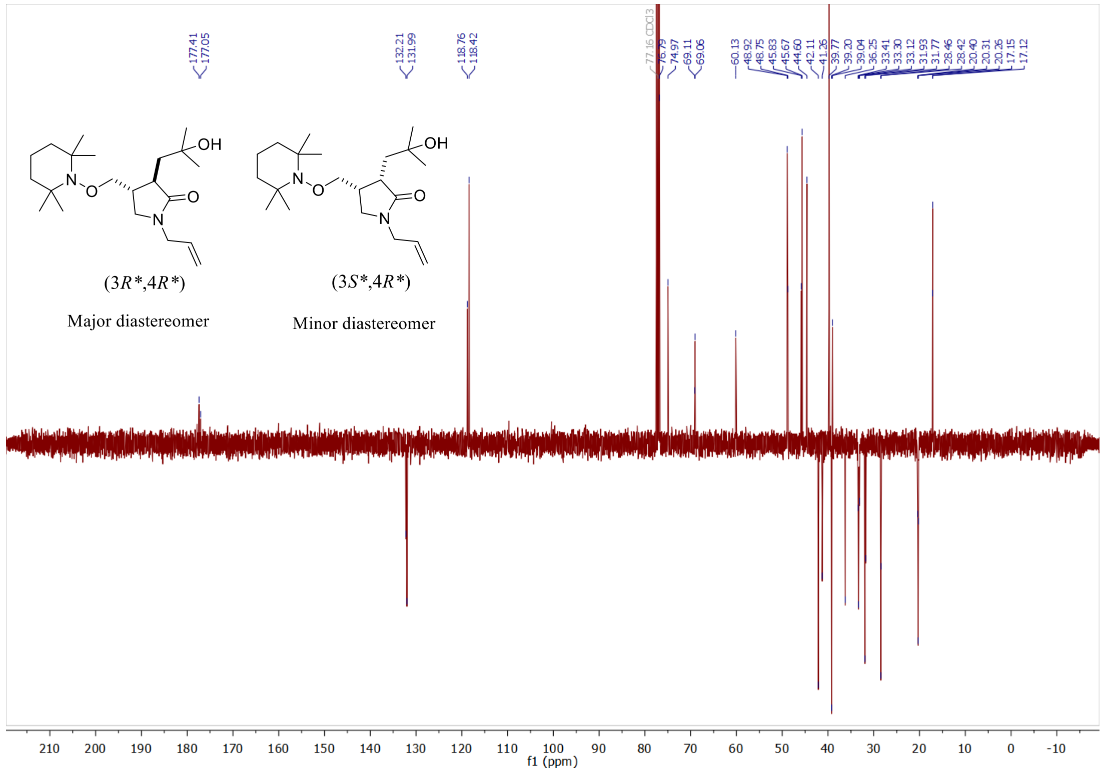

(3*R,4*R**)- and (3*S**,4*R**)-1-Allyl-3-(2-hydroxypropyl)-4-(((2,2,6,6-tetramethylpiperidin-1-yl)oxy)methyl)pyrrolidin-2-one (12b).** Spectral data for compound *trans*-**12b** are identical, only signals of the major diastereomers are more intense.

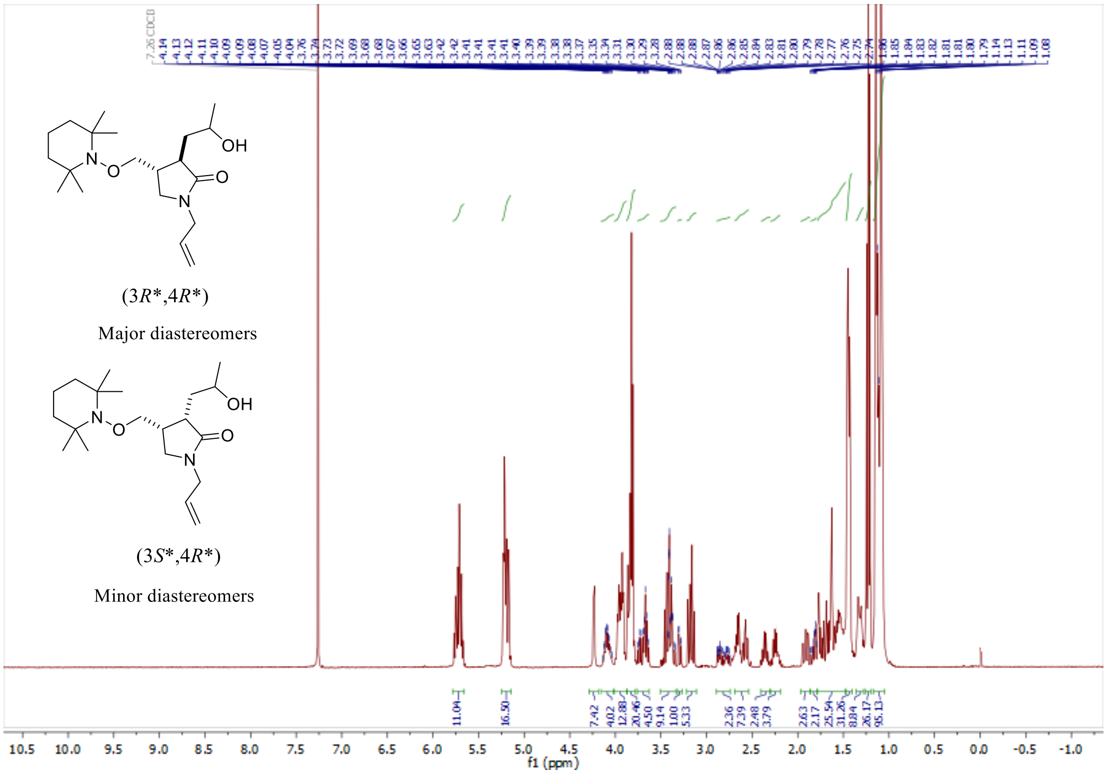

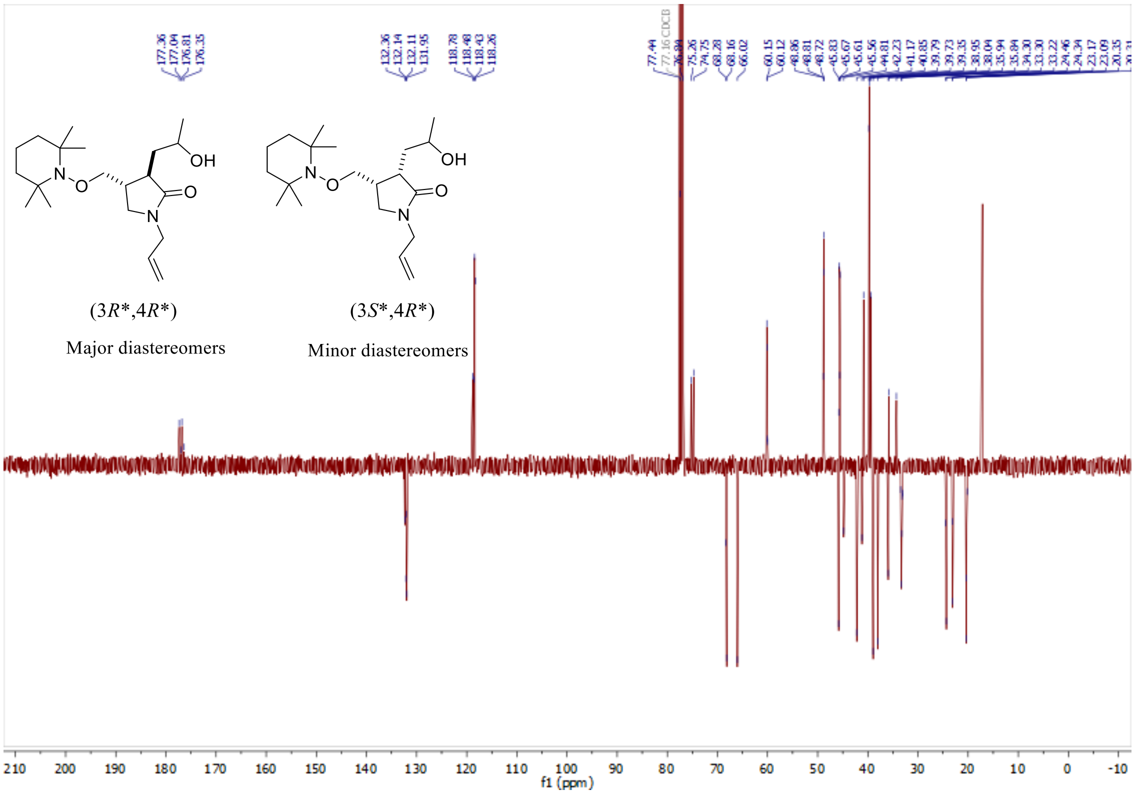

(3*R,4*R**)- and (3*S**,4*R**)-1-Allyl-3-(2-hydroxyhexyl)-4-(((2,2,6,6-tetramethylpiperidin-1-yl)oxy)methyl)pyrrolidin-2-one (12c).** Spectral data for compound *trans*-**12c** are identical, only signals of the major diastereomers are more intense.

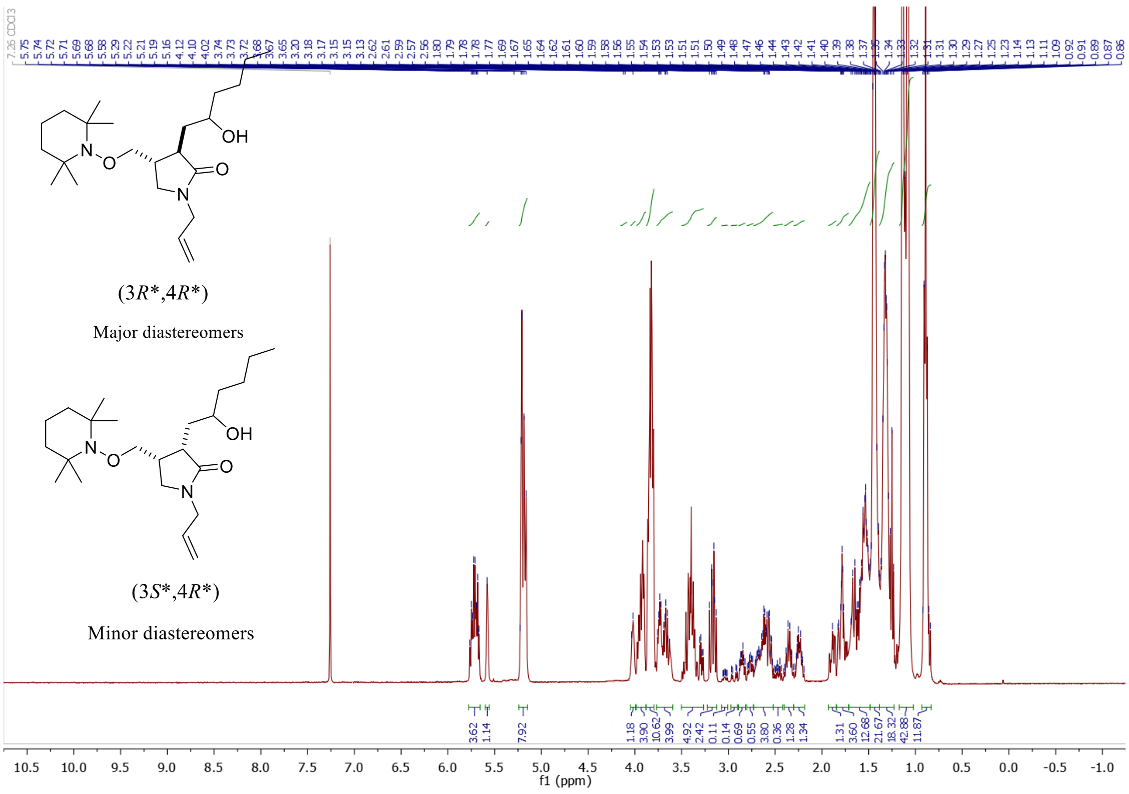

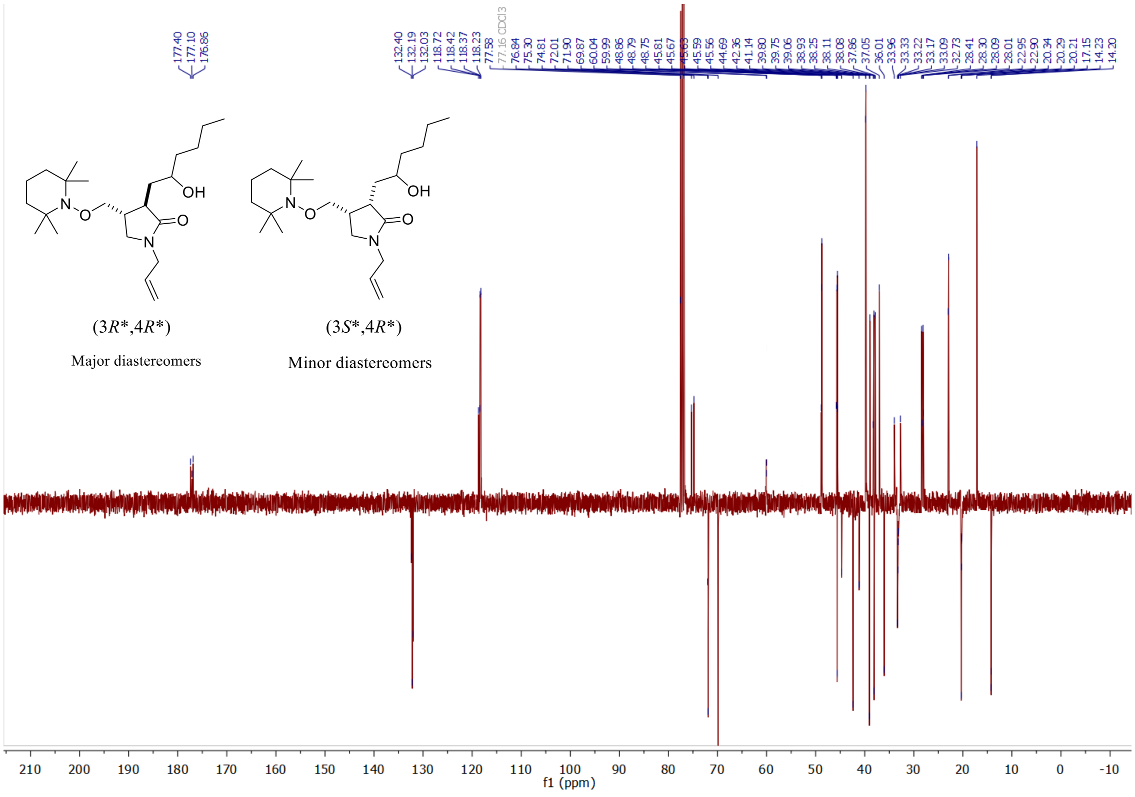

(3*R,4*R**)- and (3*S**,4*R**)-1-Allyl-3-(2-hydroxy-2-phenylethyl)-4-(((2,2,6,6-tetramethylpiperidin-1-yl)oxy)methyl)pyrrolidin-2-one (12d).** Spectral data for compound *trans*-**12d** are identical, only signals of the major diastereomers are more intense.

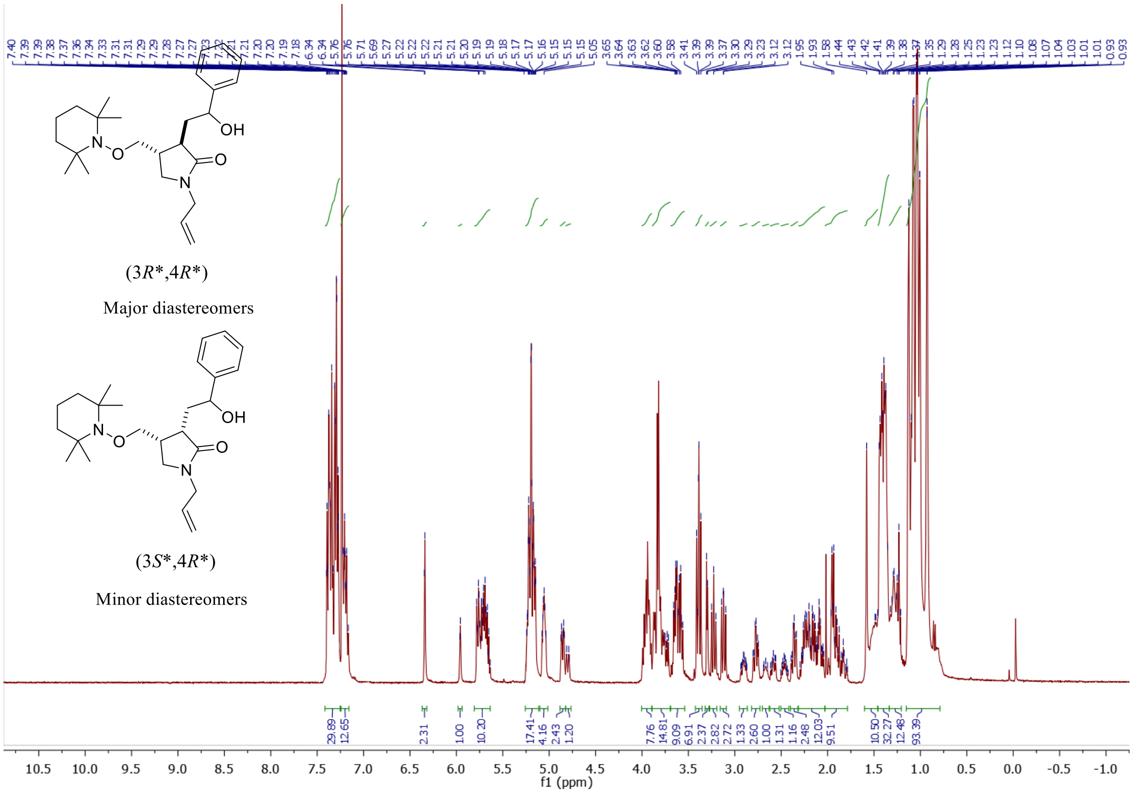

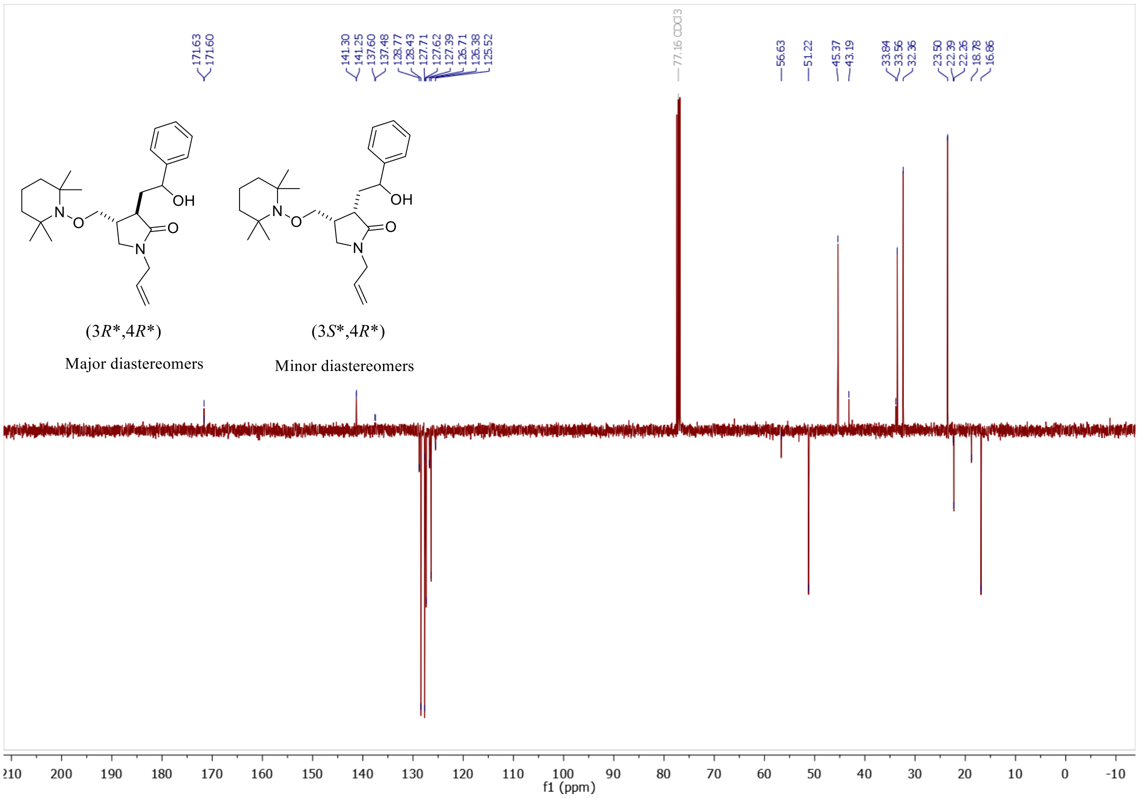

(3*R,4*R**)- and (3*S**,4*R**)-3-(2-Hydroxypropyl)-1-methyl-4-(((2,2,6,6-tetramethylpiperidin-1-yl)oxy)methyl)pyrrolidin-2-one (12e).** Spectral data for compound *trans*-**12e** are identical, only signals of the major diastereomers are more intense.

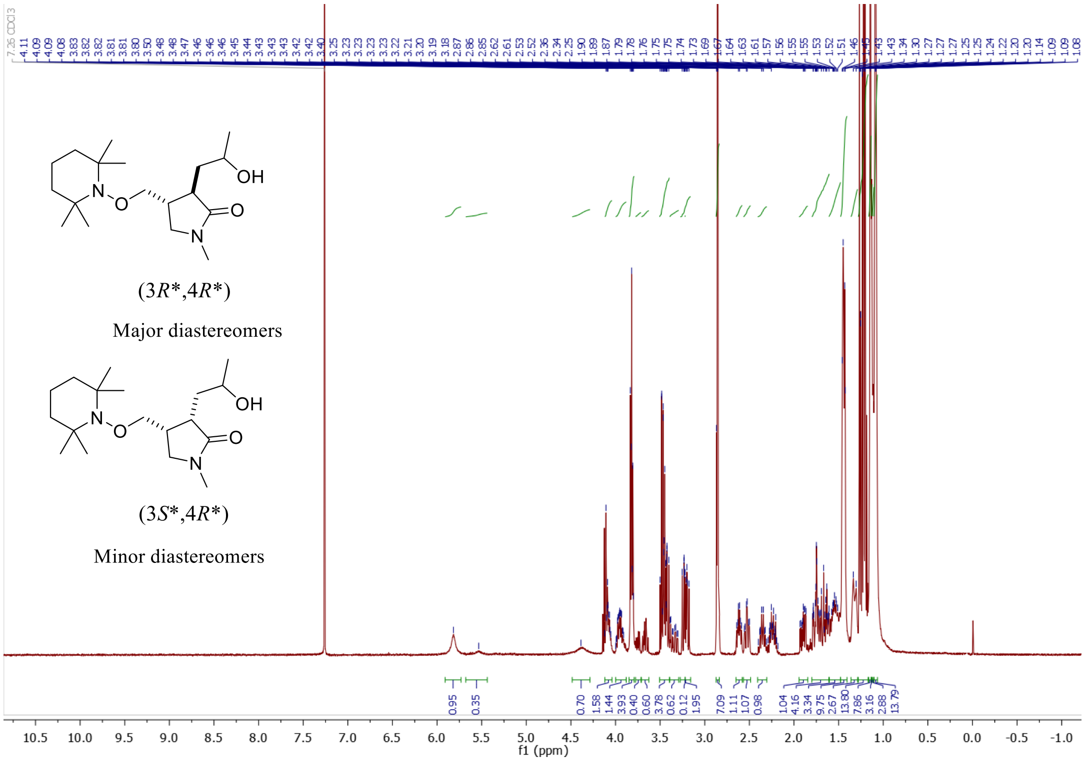

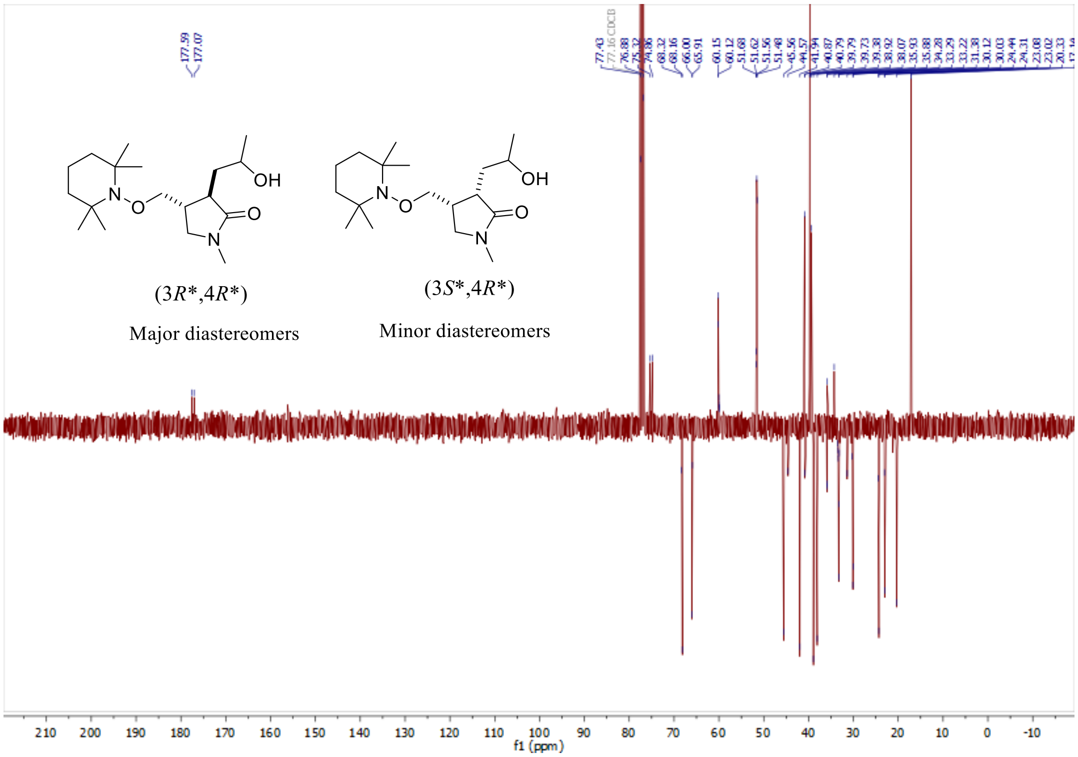

(3*R,4*R**)- and (3*S**,4*R**)-1-Benzyl-3-(2-hydroxypropyl)-4-methyl-4-(((2,2,6,6-tetramethyl-piperidin-1-yl)oxy)methyl)pyrrolidin-2-one (12f)**

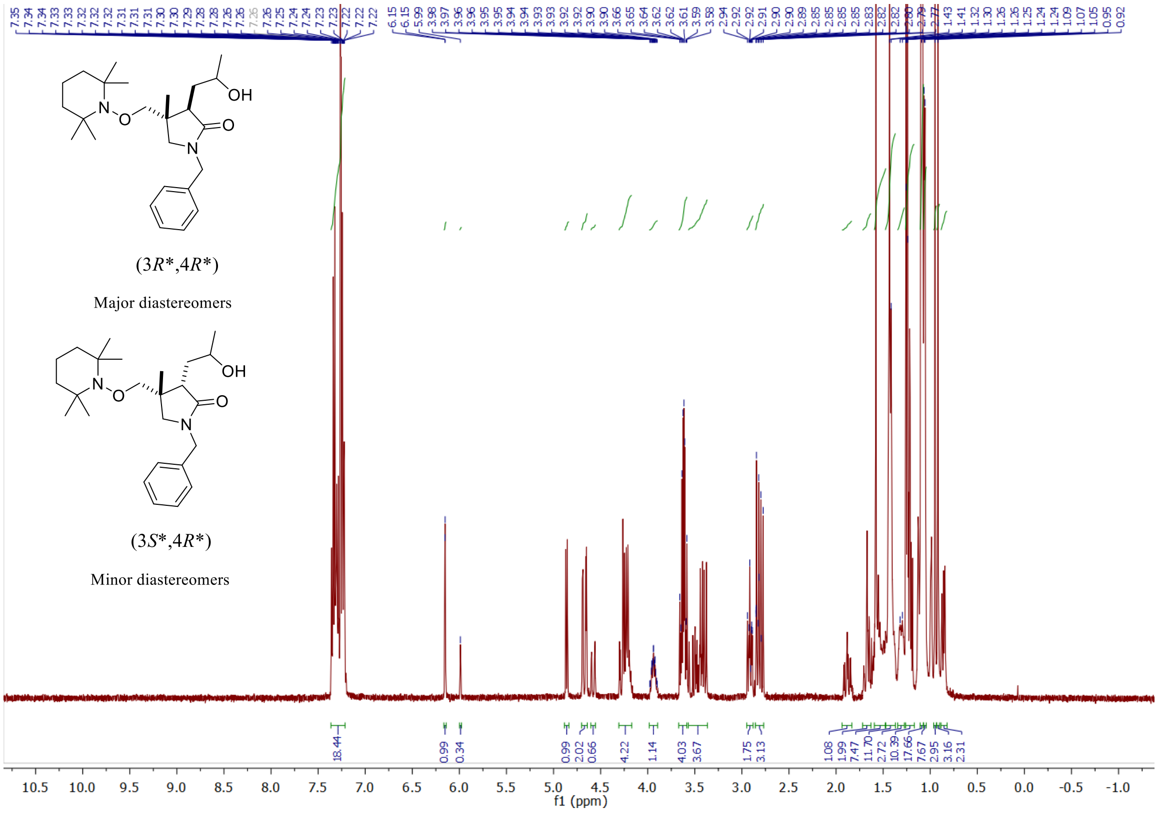

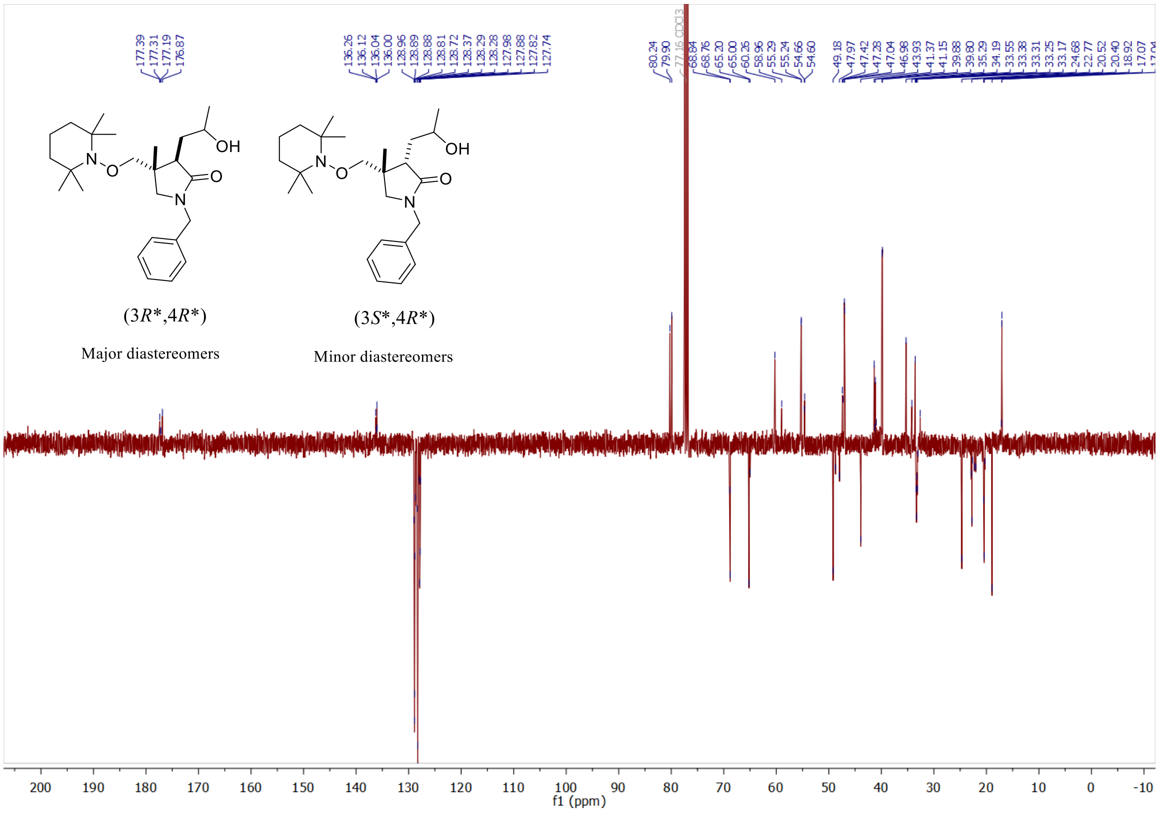

(3*R,4*S**)- and (3*S**,4*S**)-1-Benzyl-3-(2-hydroxypropyl)-4-(prop-1-en-2-yl)pyrrolidin-2-one (12g).** Spectral data for compound *trans*-**12g** are identical, only signals of the major diastereomers are more intense.

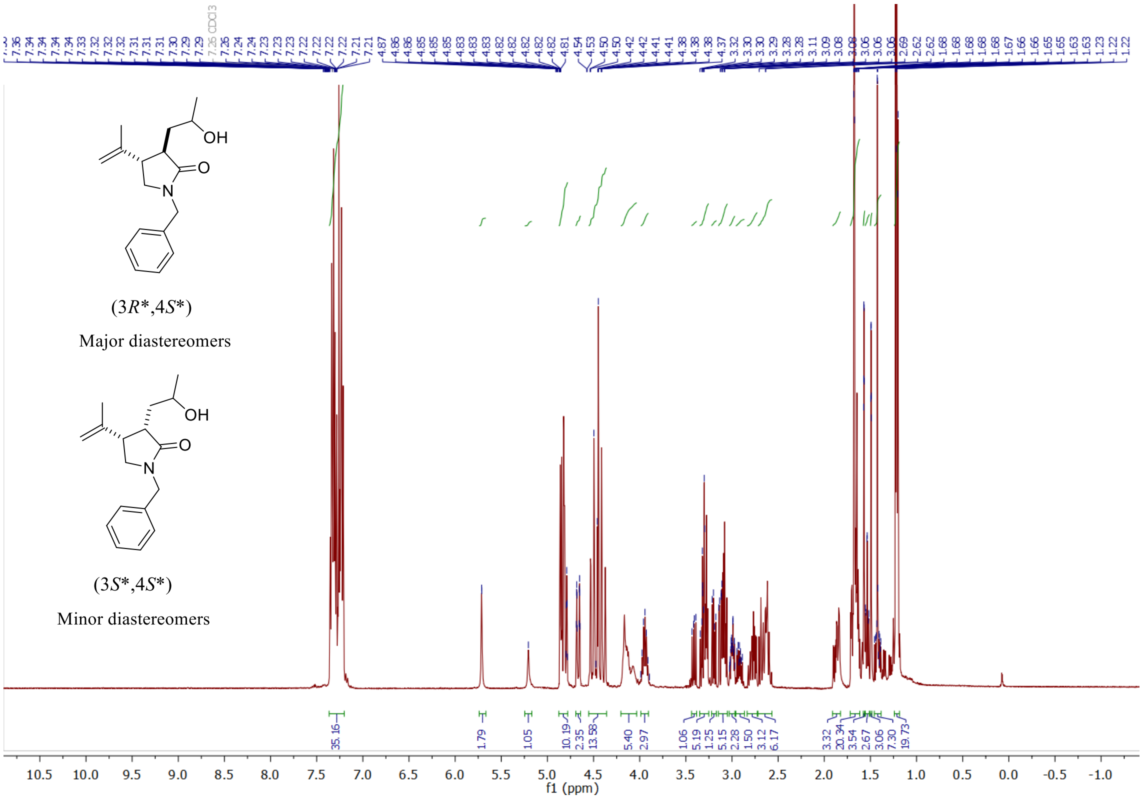

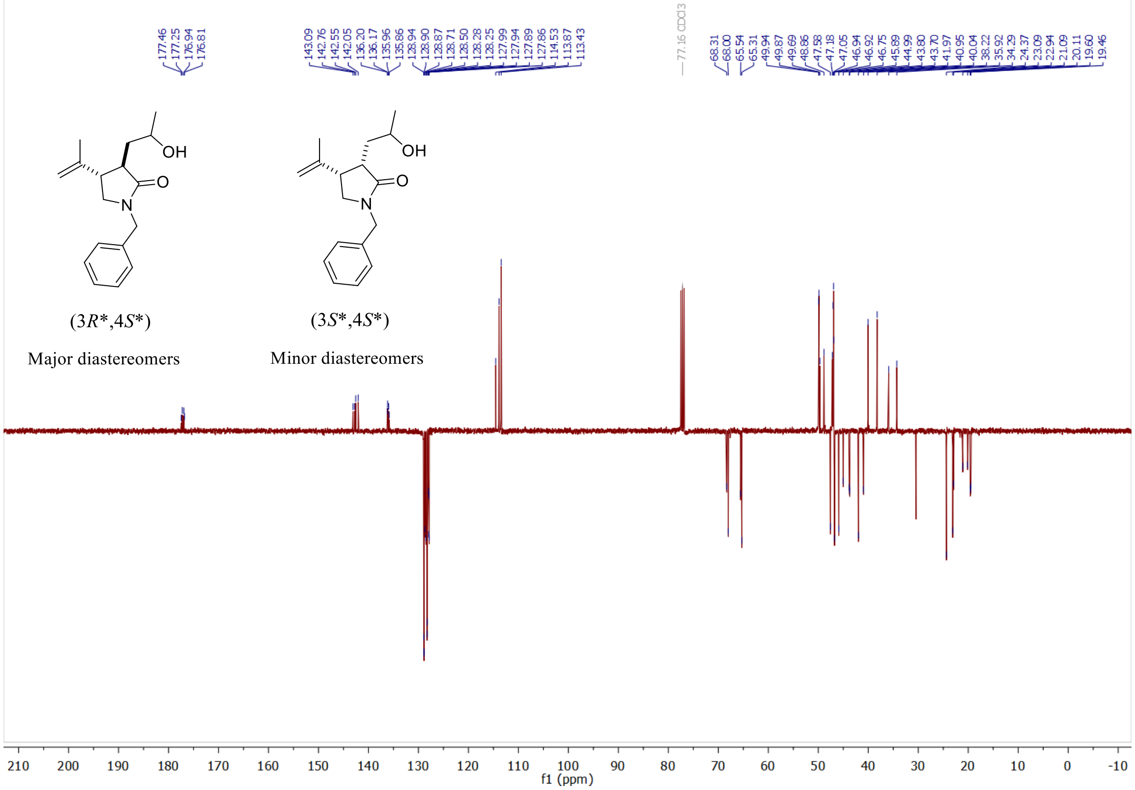

(3*R*,4*R*)- and (3*S*,4*S*)- and (3*R*,4*S*)- and (3*S*,4*R*)-1-Allyl-3-((*S*)-3-(benzyloxy)-2-hydroxypropyl)-4-(((2,2,6,6-tetramethylpiperidin-1-yl)oxy)methyl)pyrrolidin-2-one (12h). Spectral data for compound *trans*-**12h** are identical, only signals of the major diastereomers are more intense.

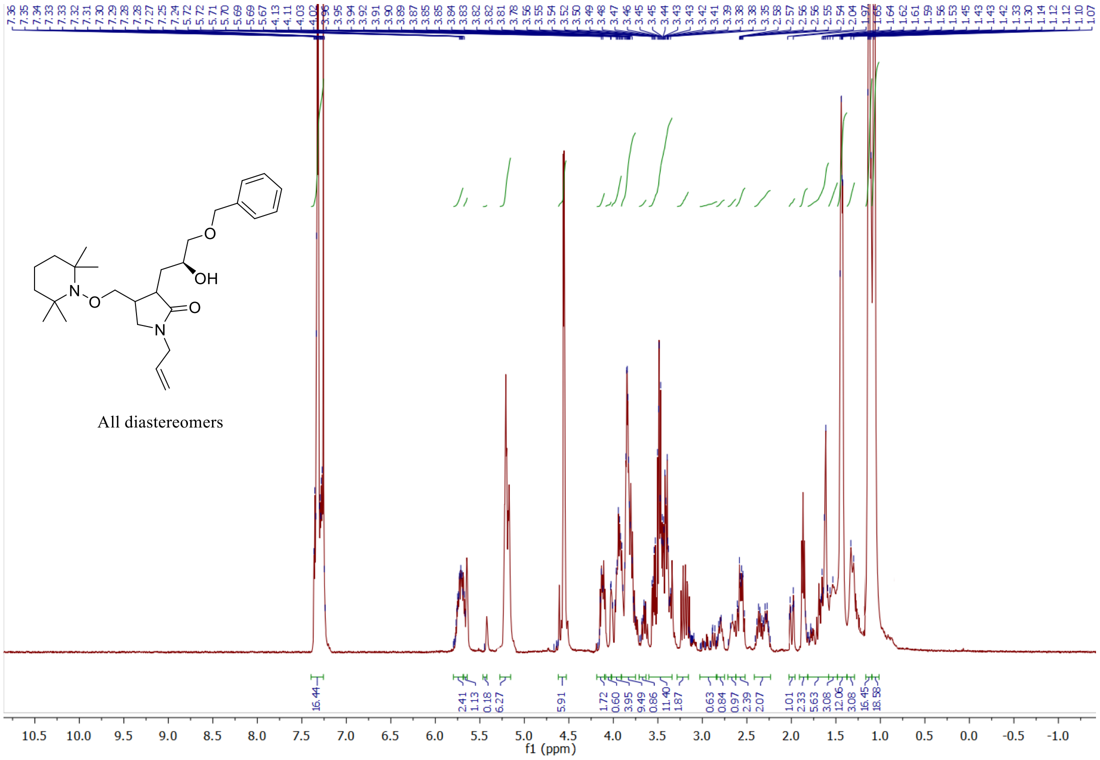

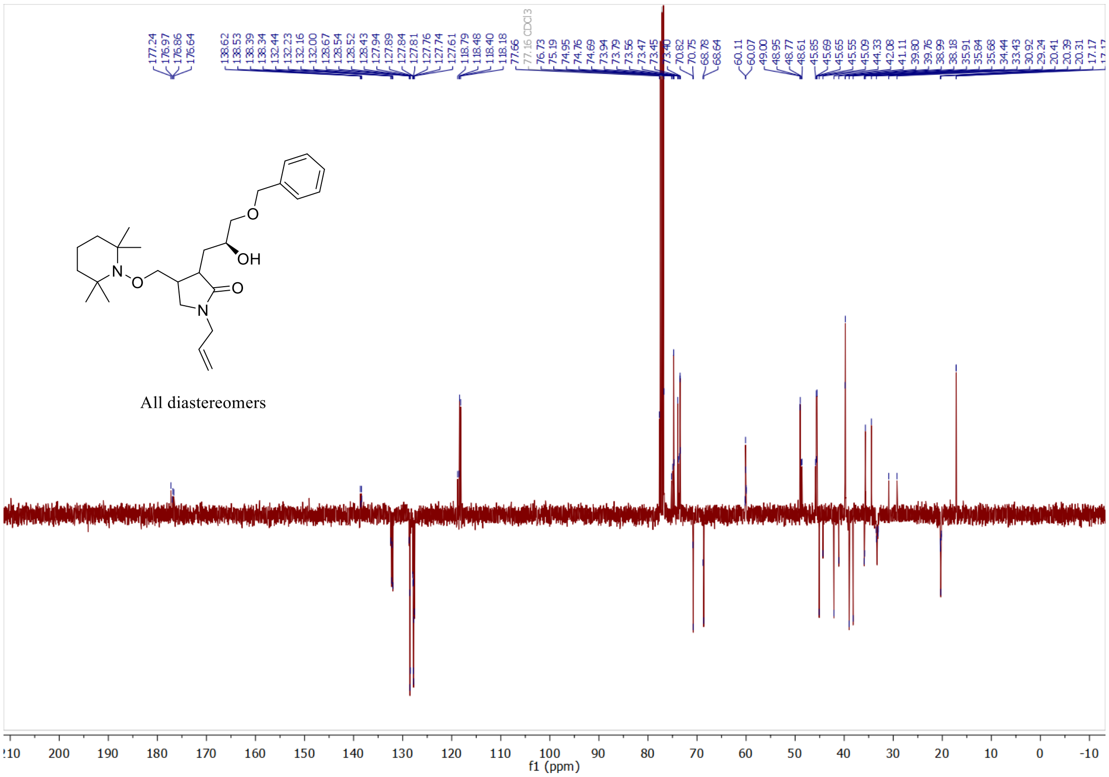

(3*R*,4*R*)- and (3*S*,4*S*)- and (3*R*,4*S*)- and (3*S*,4*R*)-3-((*S*)-2-Hydroxypropyl)-1-((*S*)-1-phenylethyl)-4-(((2,2,6,6-tetramethylpiperidin-1-yl)oxy)methyl)pyrrolidin-2-one (12i).

Spectral data for compound *trans*-**12i** are identical, only signals of the major diastereomers are more intense.

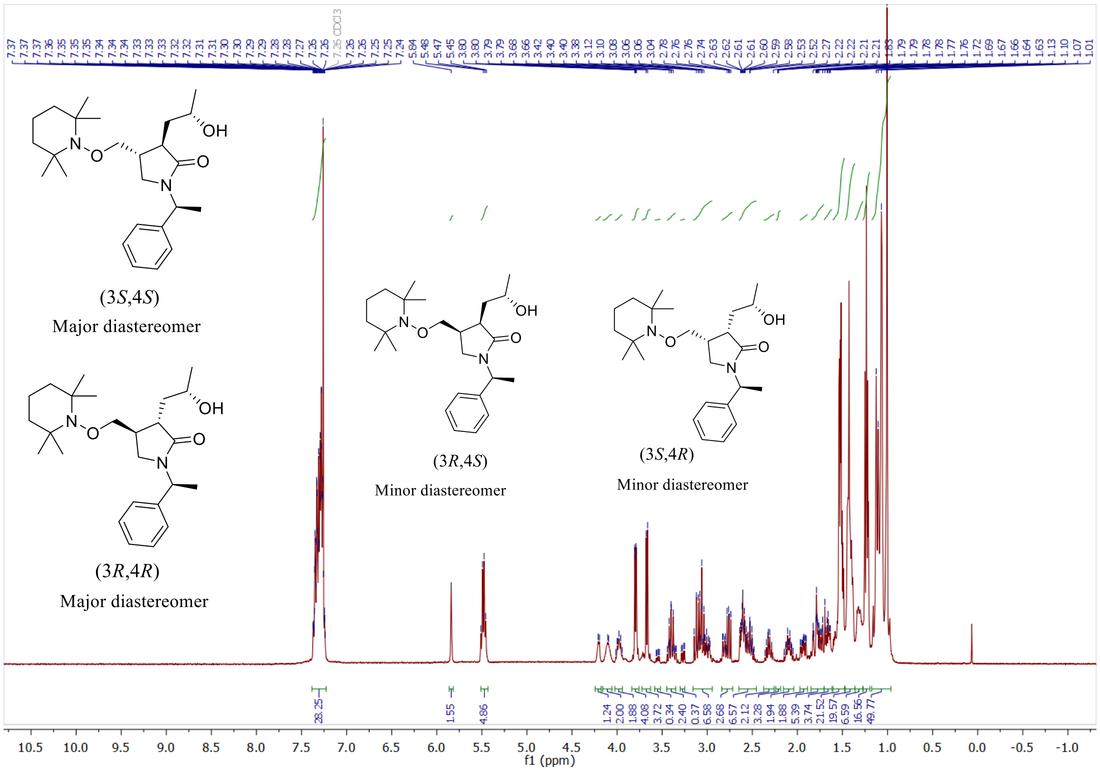

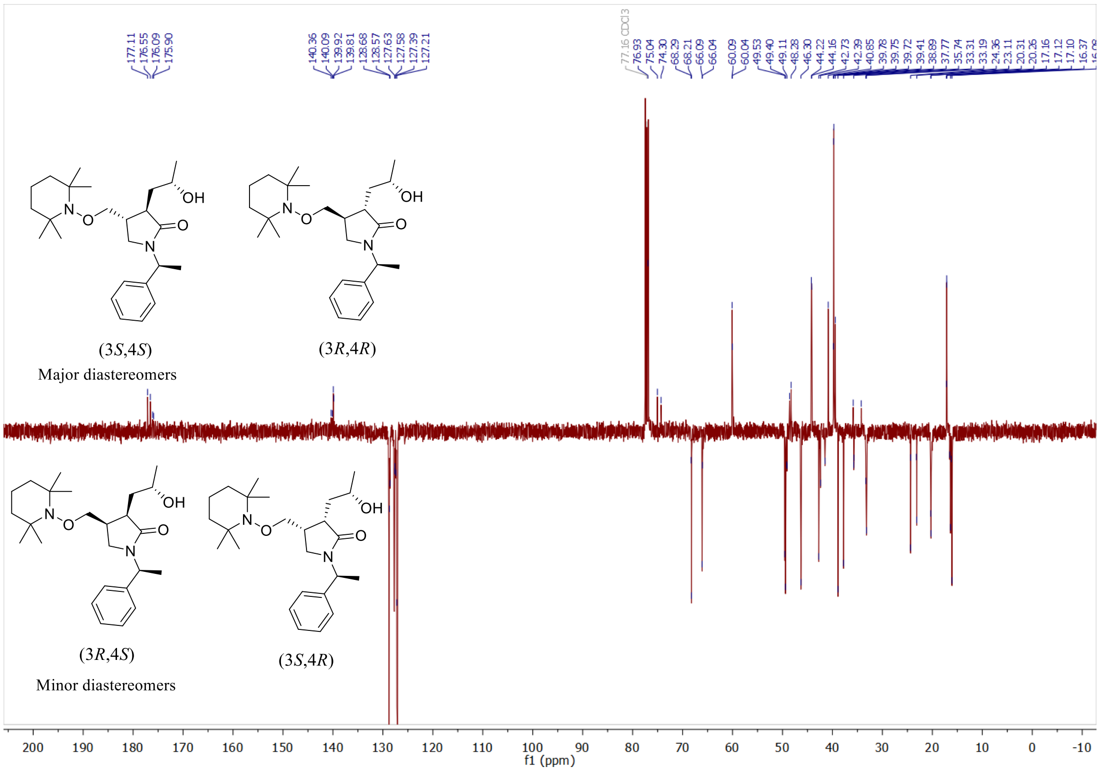

(3*R*,4*R*)- and (3*S*,4*S*)- and (3*R*,4*S*)- and (3*S*,4*R*)-3-((*S*)-2-Hydroxypropyl)-1-((*S*)-1-(naphthalen-2-yl)ethyl)-4-(((2,2,6,6-tetramethylpiperidin-1-yl)oxy)methyl)pyrrolidin-2-one (12j). Spectral data for compound *trans*-**12j** are identical, only signals of the major diastereomers are more intense.

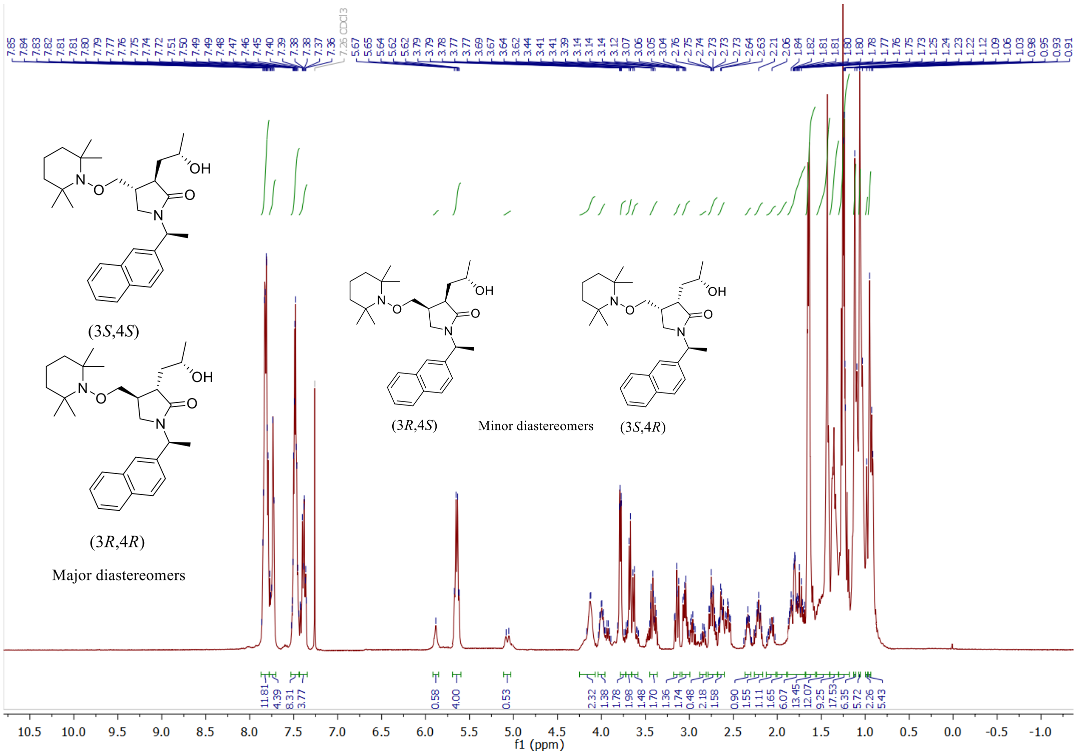

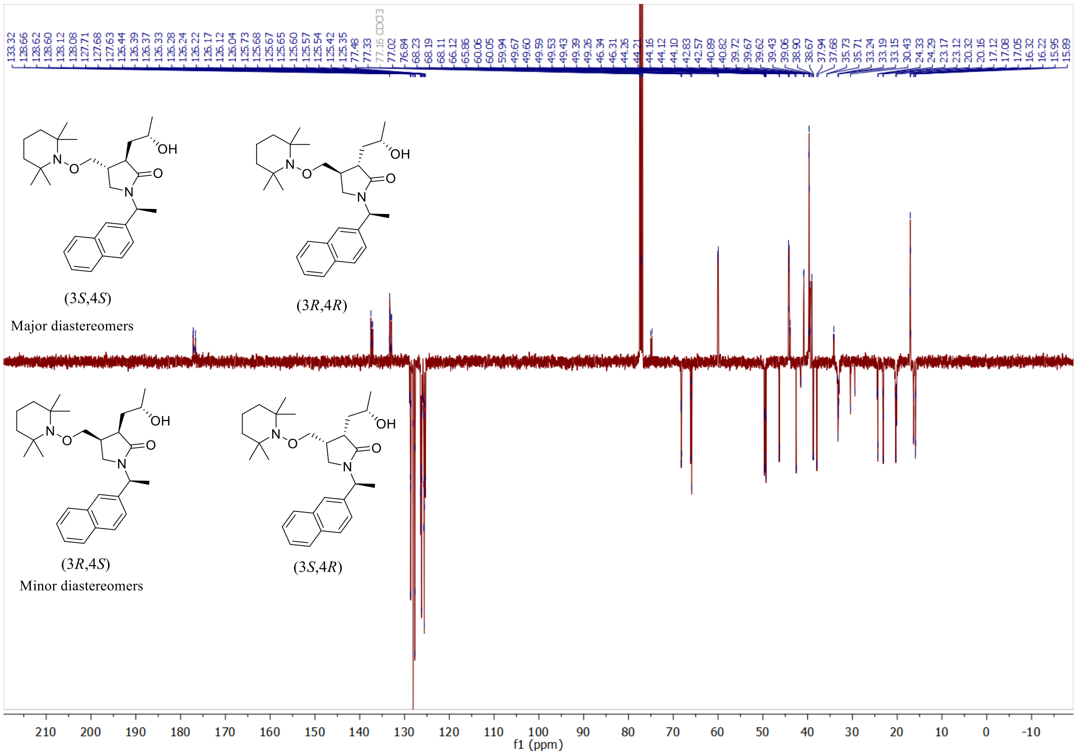

(3*R*,4*S*)- and (3*S*,4*R*)- and (3*R*,4*R*)- and (3*S*,4*S*)-3-((*S*)-2-Hydroxypropyl)-1-((*S*)-1-phenylethyl)-4-(prop-1-en-2-yl)pyrrolidin-2-one (12k). Spectral data for compound *trans*-**12k** are identical, only signals of the major diastereomers are more intense.

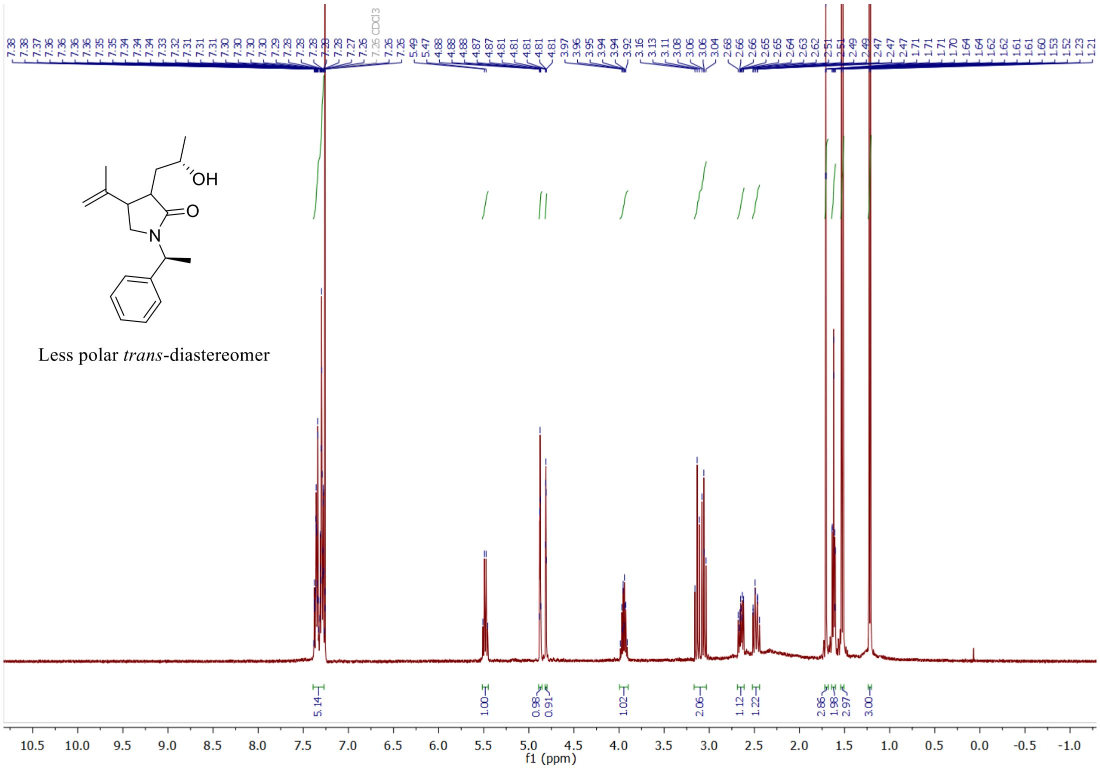

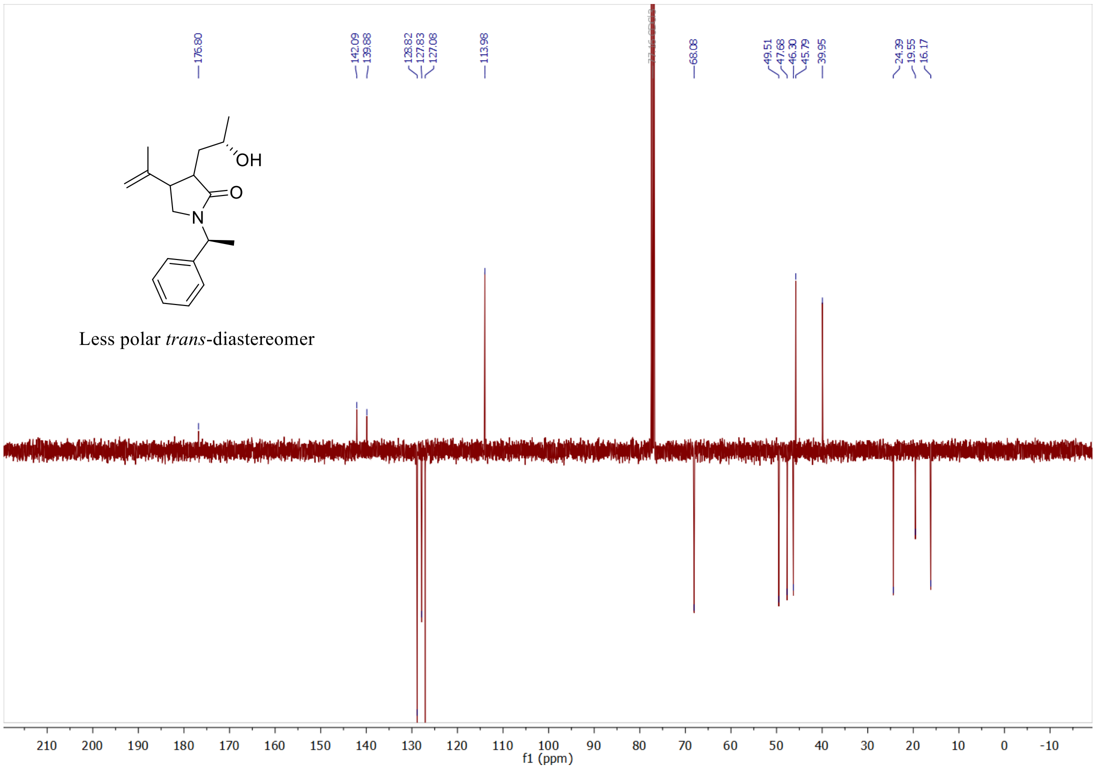

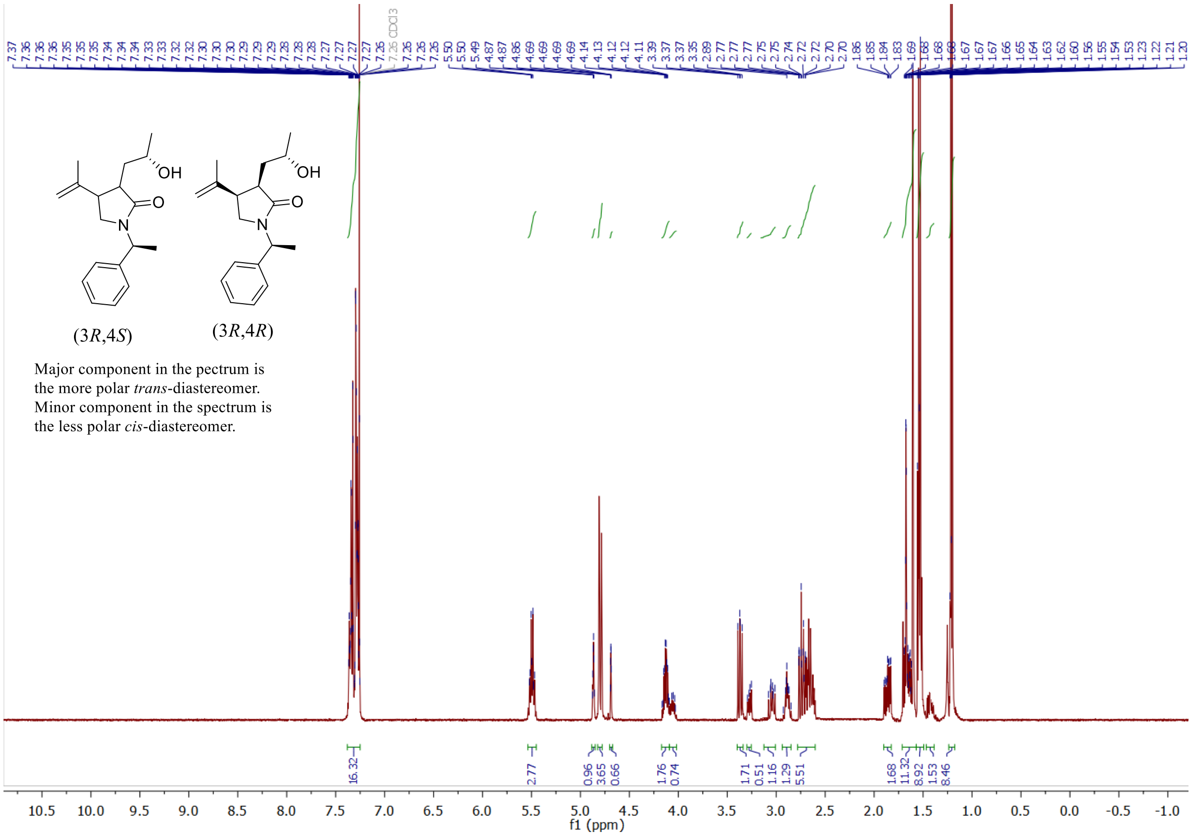
(3R,4S)
(3R,4R)
Major component in the pectrum is the more polar trans-diastereomer. Minor component in the spectrum is the less polar cis-diastereomer.
f1 (ppm)

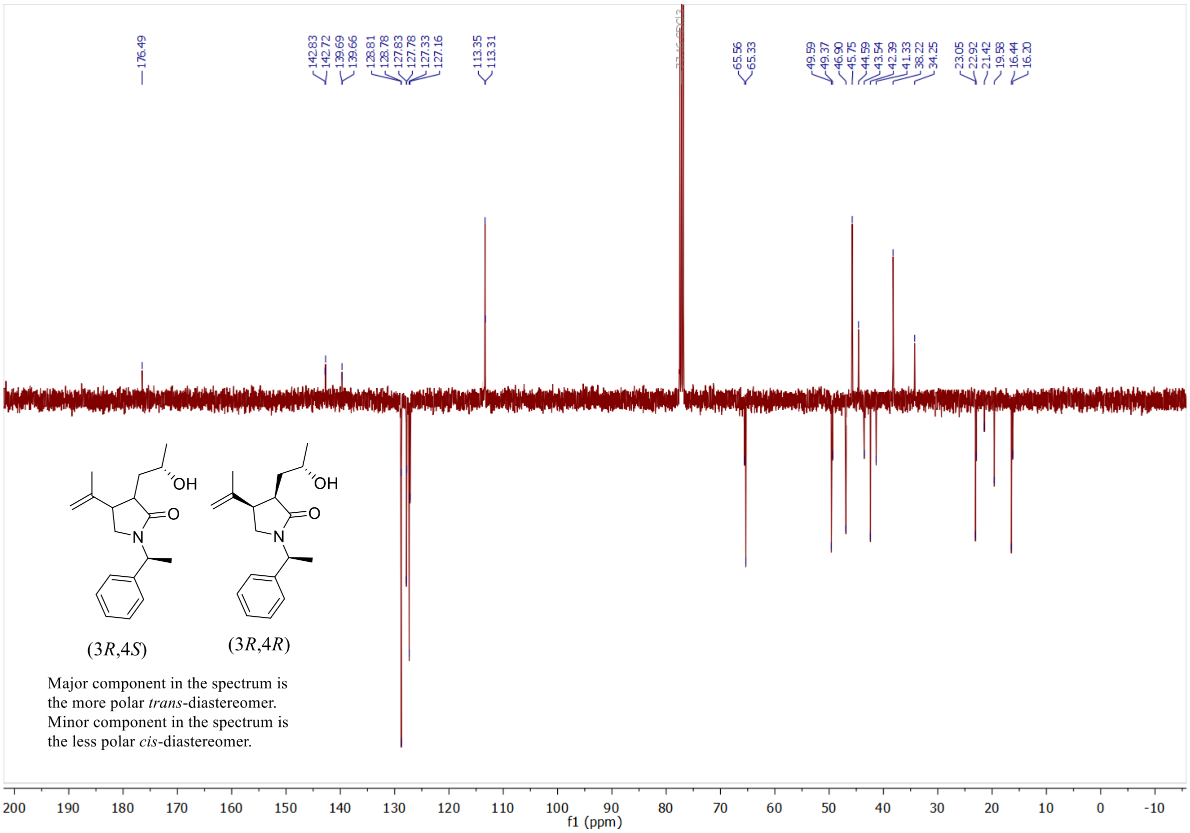
176.49
142.83
142.72
139.69
139.66
128.81
128.78
127.78
127.33
127.16
113.35
113.31
65.56
65.33
49.59
49.37
46.90
45.75
44.59
43.54
42.39
41.33
38.22
34.25
23.05
22.92
21.42
19.58
16.44
16.20
(3R,4S)
(3R,4R)
Major component in the spectrum is the more polar trans-diastereomer. Minor component in the spectrum is the less polar cis-diastereomer.
f1 (ppm)

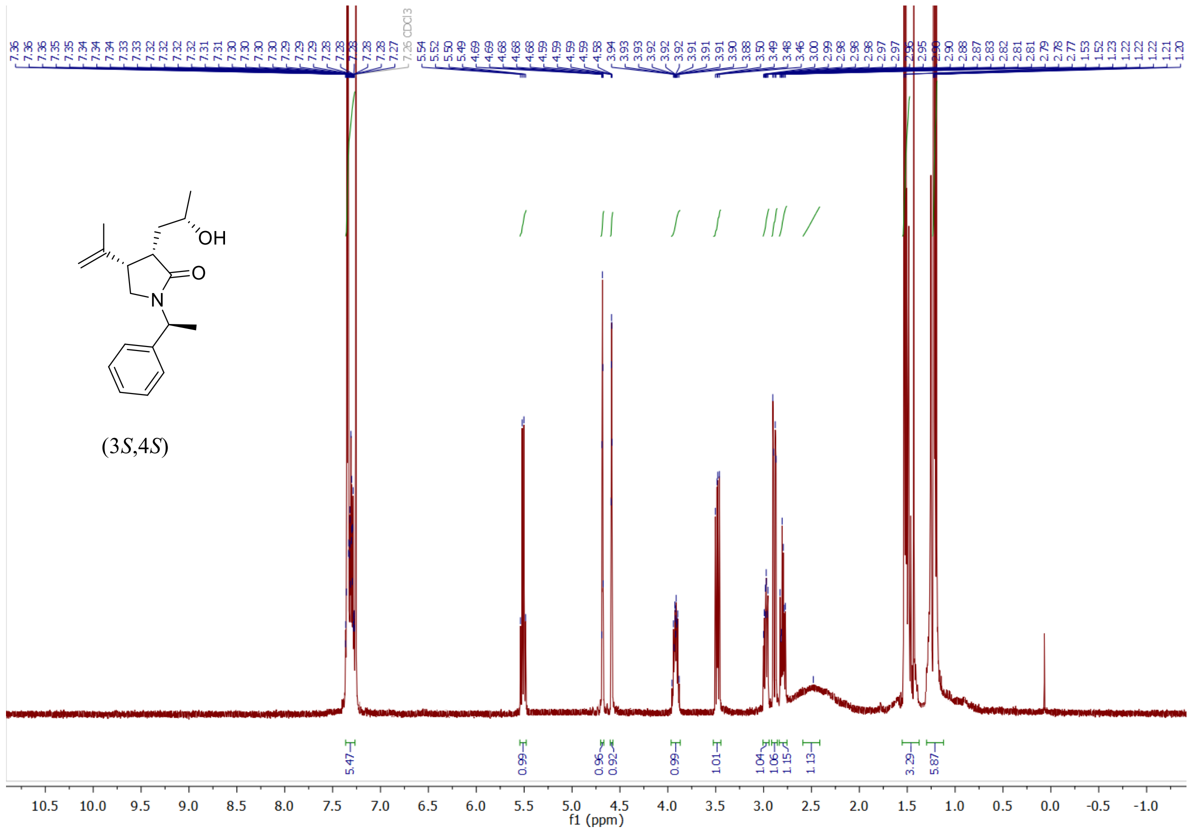

(3*S*,4*S*)

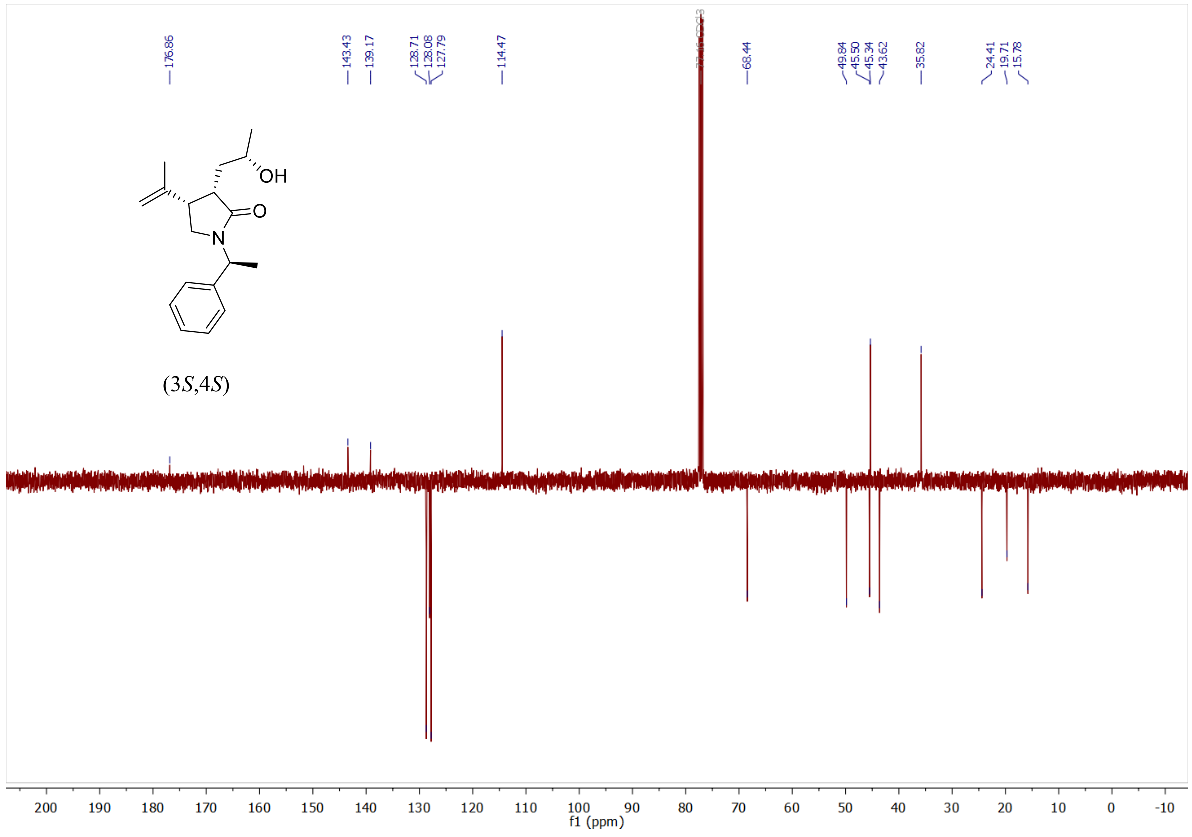

(3*S*,4*S*)

(3*R,4*S**)- and (3*S**,4*R**)- and (3*R**,4*R**)-(1-Allyl-3-((1*S**,2*R**)-2-hydroxycyclohexyl)-4-(((2,2,6,6-tetramethylpiperidin-1-yl)oxy)methyl)pyrrolidin-2-one (12l)**

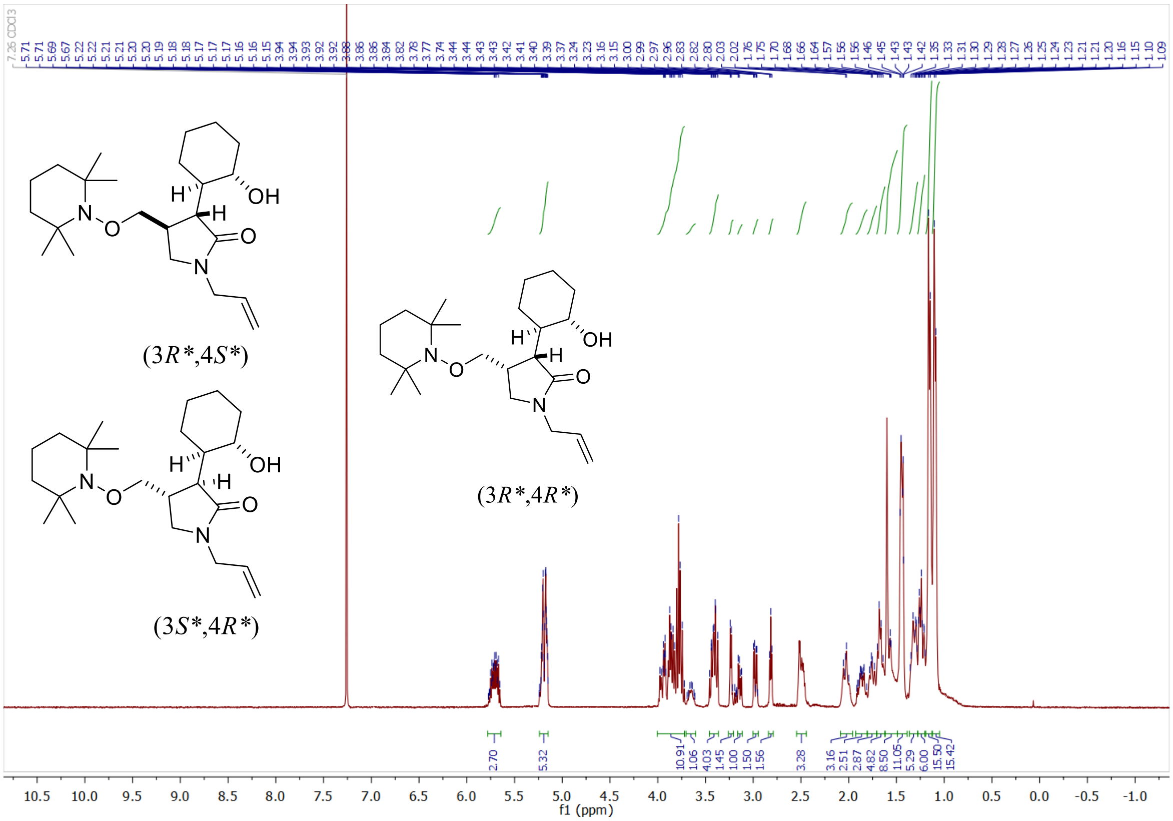

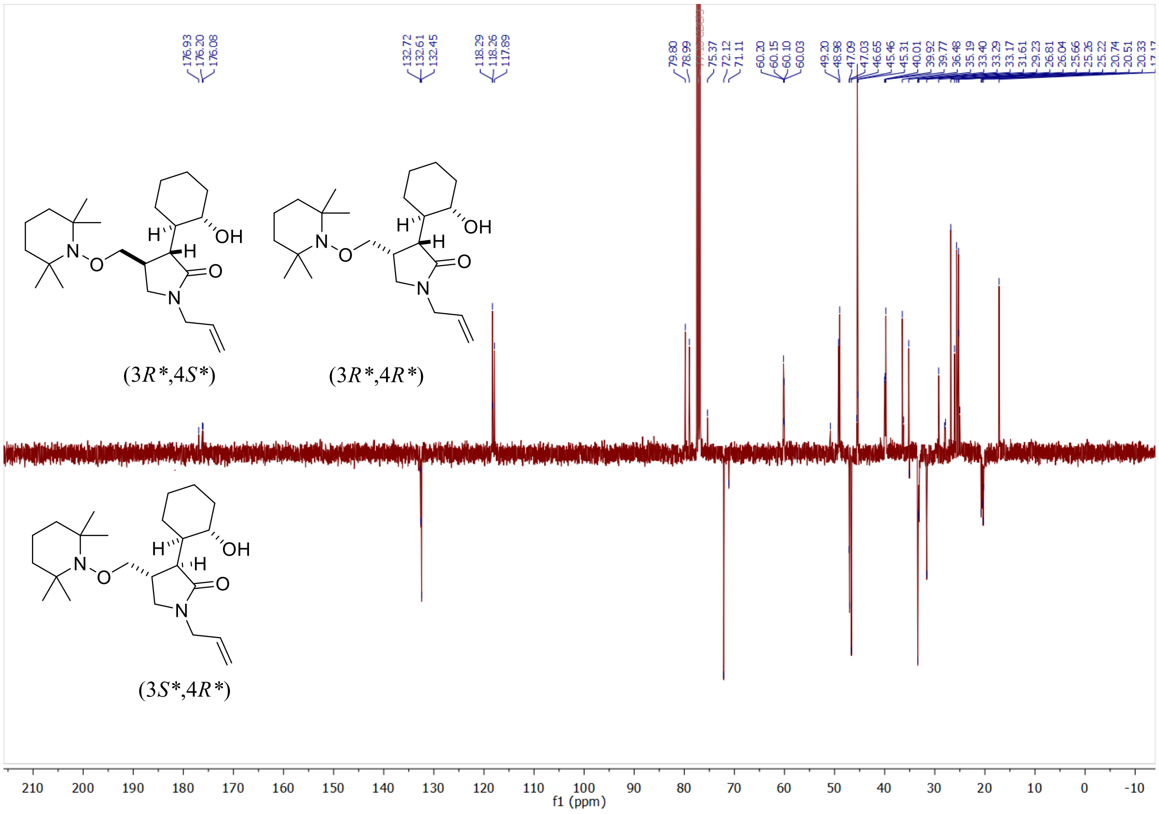

(4*S,5*S**,6*R**)- and (4*S**,5*S**,6*S**)- and (4*R**,5*S**,6*R**)- and (4*R**,5*S**,6*S**)-2-Benzyl-4-(2-hydroxypropyl)-6-((2,2,6,6-tetramethylpiperidin-1-yl)oxy)-2-azaspiro[4.4]nonan-3-one (12m).** Spectral data for compound *equil*-**12m** are identical, only signals of the major diastereomers are more intense.

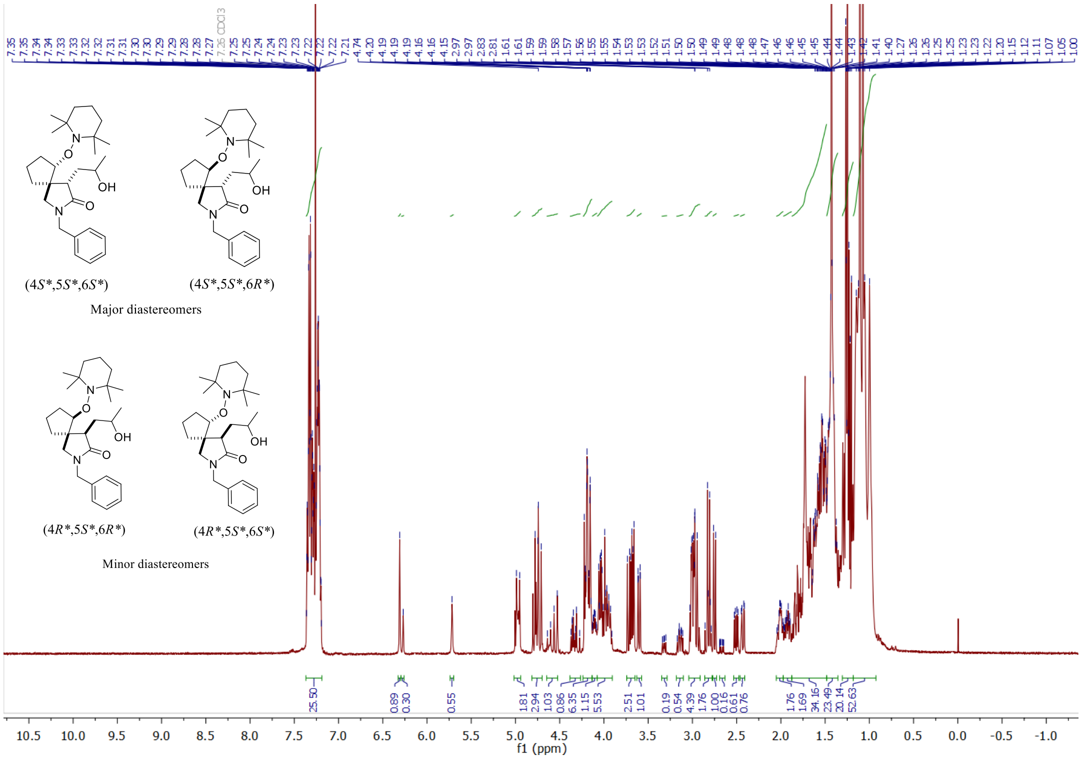

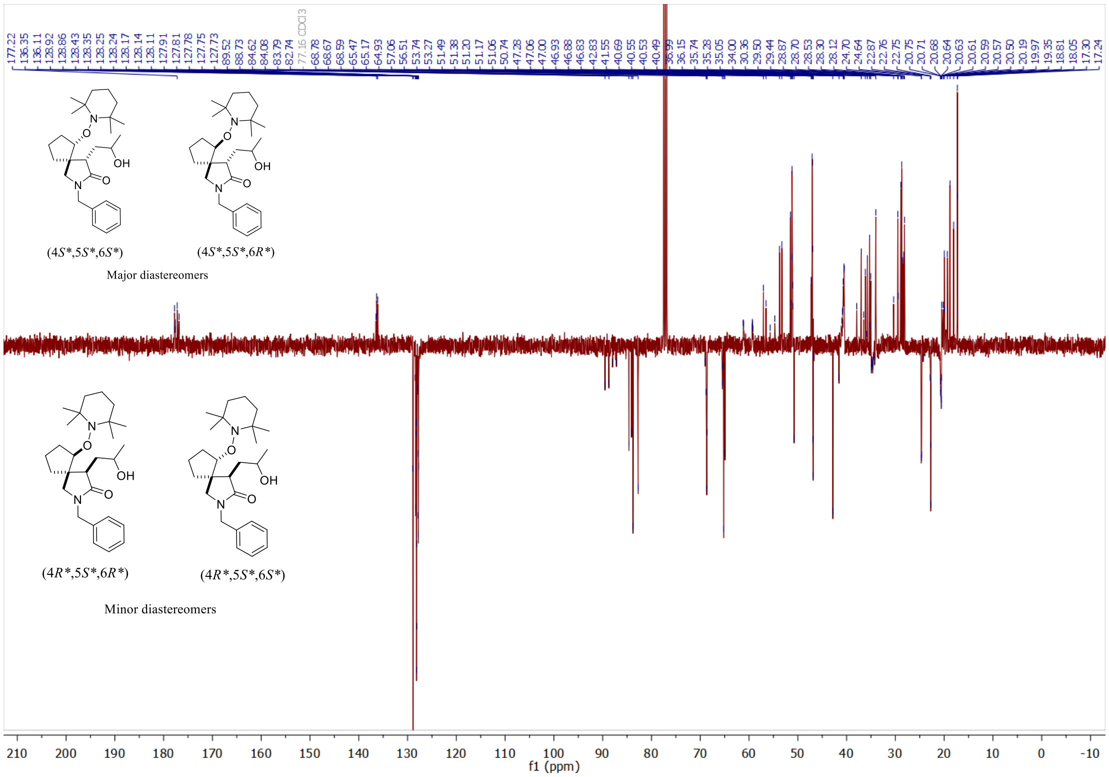

(4*S,5*S**,6*R**)- and (4*S**,5*S**,6*S**)- and (4*R**,5*S**,6*R**)-2-Benzyl-4-(2-hydroxypropyl)-6-((2,2,6,6-tetramethylpiperidin-1-yl)oxy)-2-azaspiro[4.5]decan-3-one (12n).** Spectral data for compound *equil*-**12n** are identical, only signals of the major diastereomers are more intense.

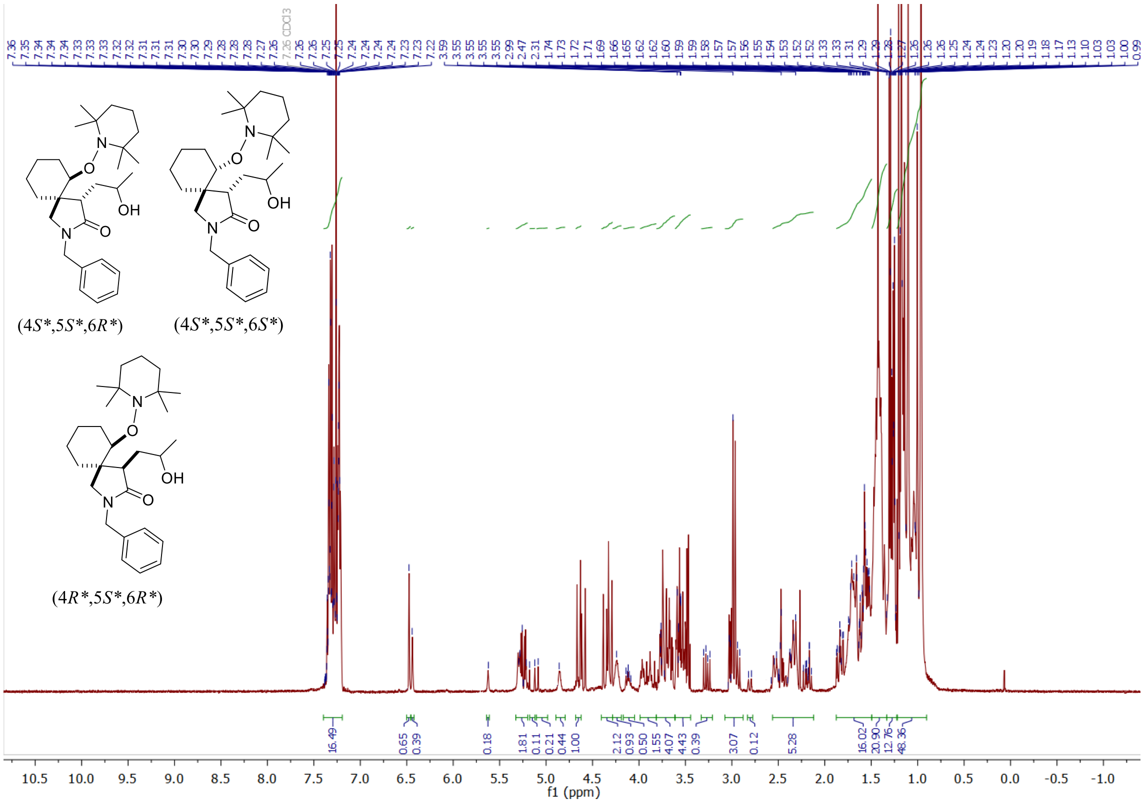

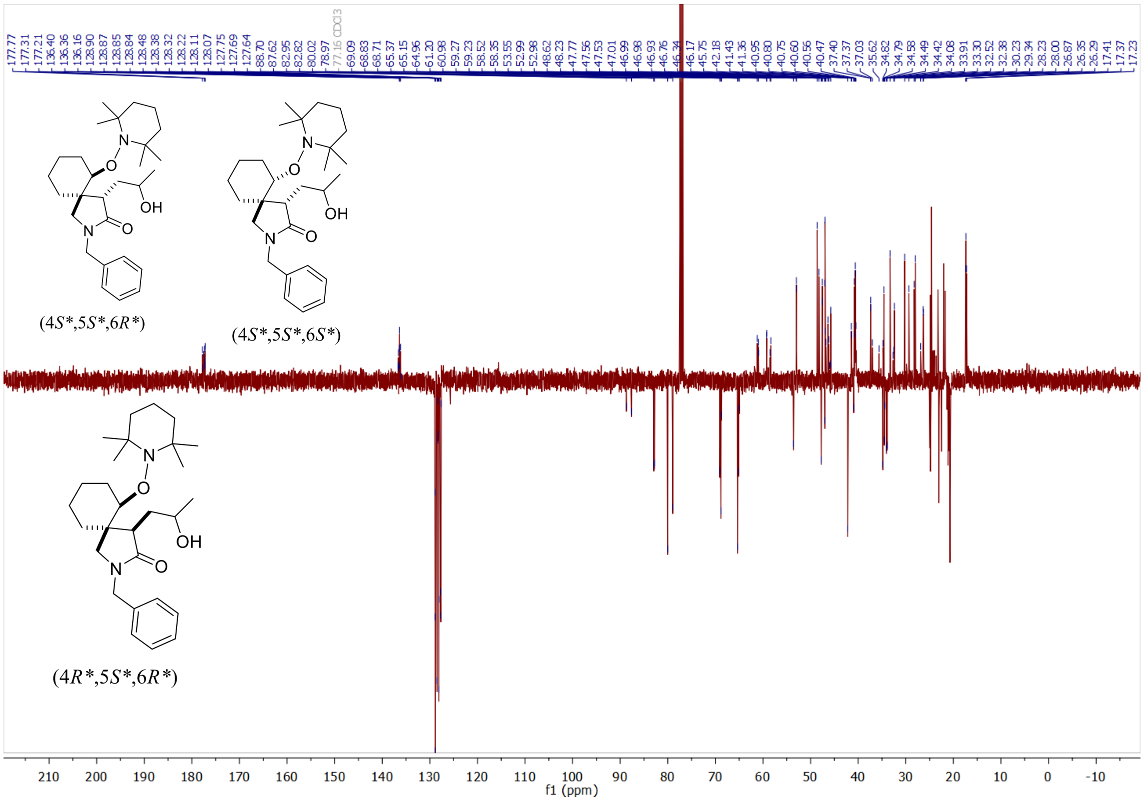

(3*R,3a*R**,4*R**,6a*R**)- and (3*S**,3a*R**,4*R**,6a*R**)-1-Benzyl-3-(2-hydroxypropyl)-4-((2,2,6,6-tetramethylpiperidin-1-yl)oxy)hexahydrocyclopenta[*b*]pyrrol-2(1*H*)-one (12o):**

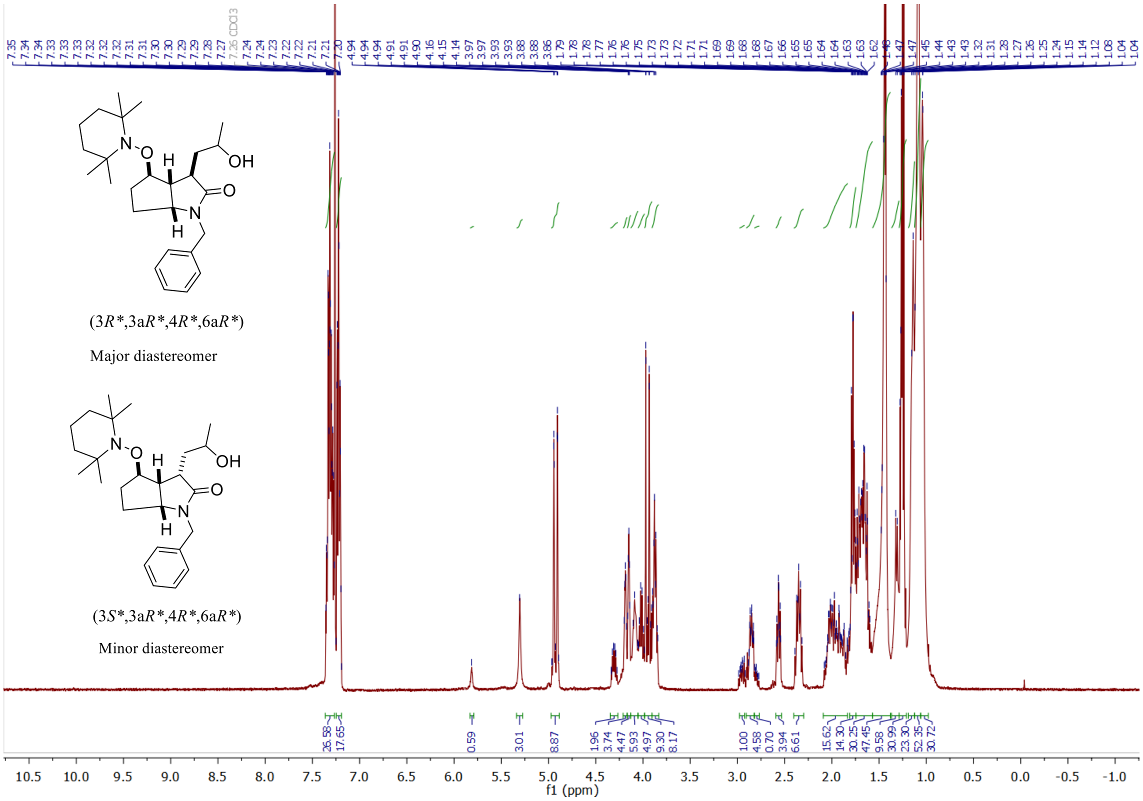

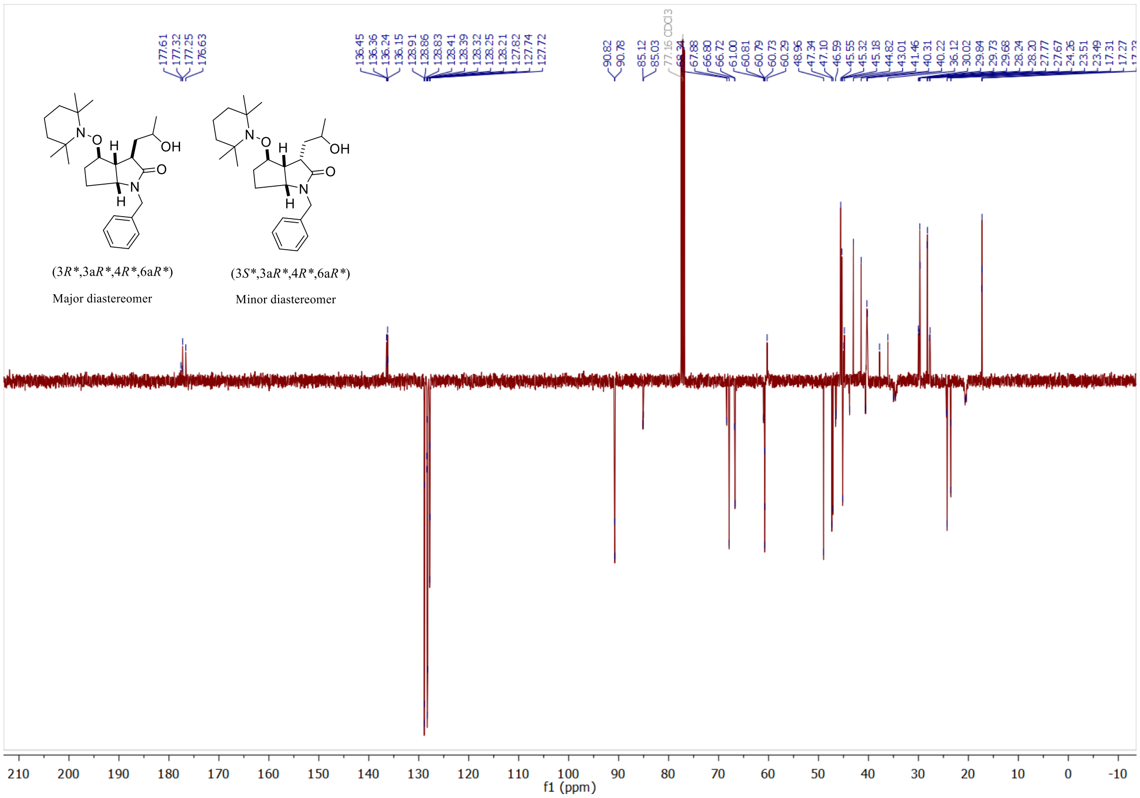

(3*R*,3a*R*,4*R*,7a*R*)-1-Benzyl-3-(2-hydroxypropyl)-4-((2,2,6,6-tetramethylpiperidin-1-yl)oxy)octahydro-2*H*-indol-2-one (12pA):

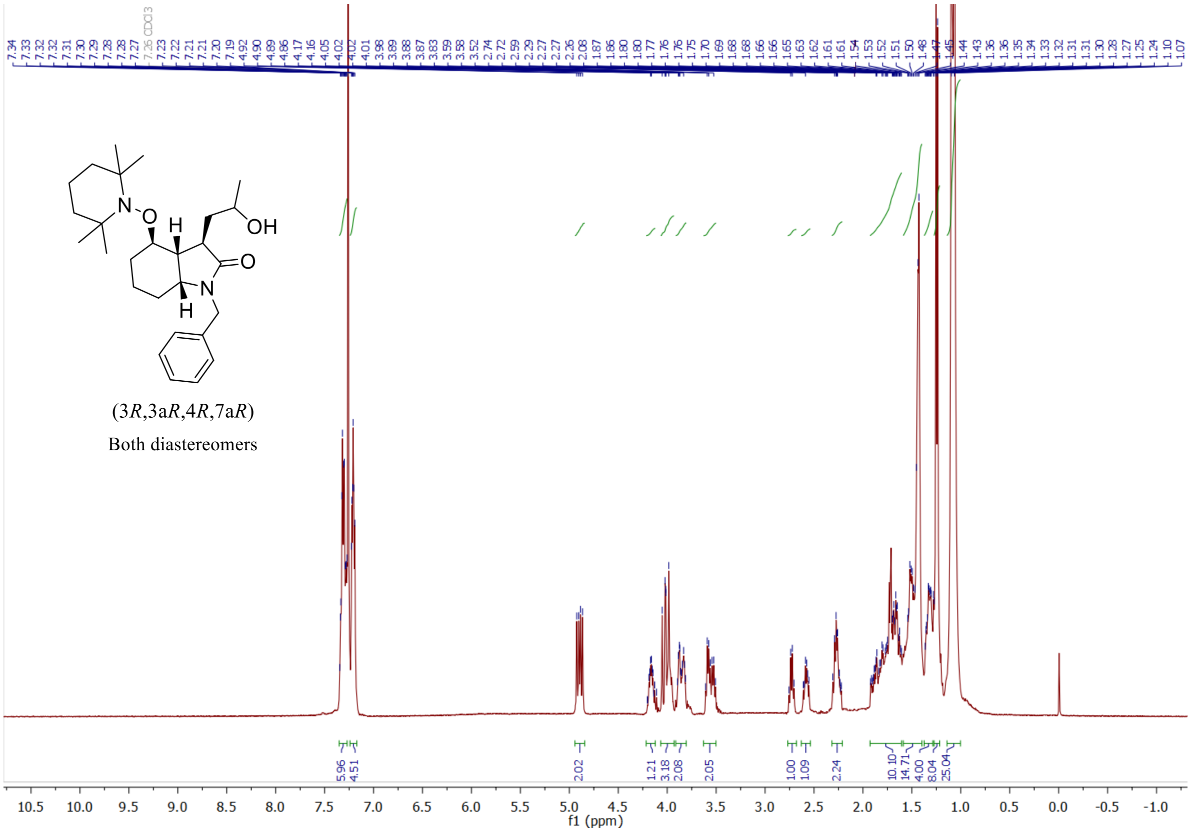

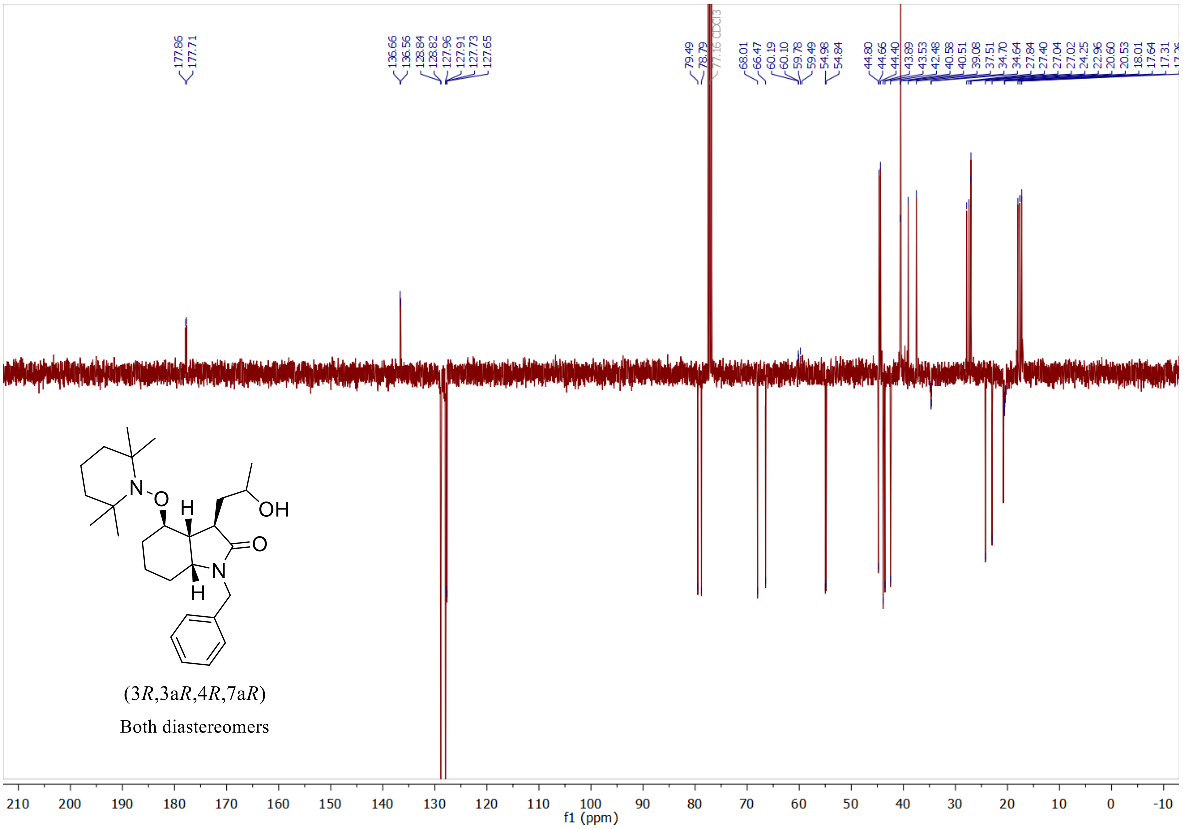

(3*R,4*R**)- and (3*S**,4*R**)-1-Allyl-3-(2-oxopropyl)-4-(((2,2,6,6-tetramethylpiperidin-1-yl)oxy)methyl)pyrrolidin-2-one (13b)**

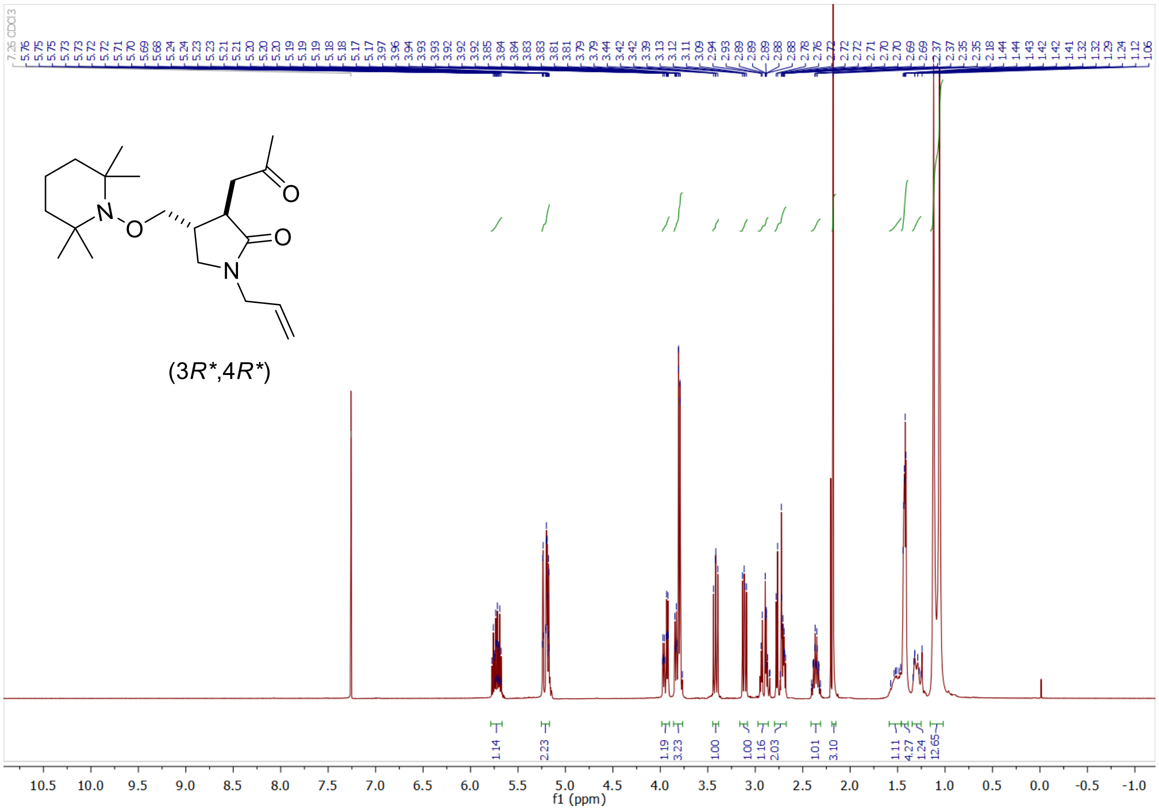

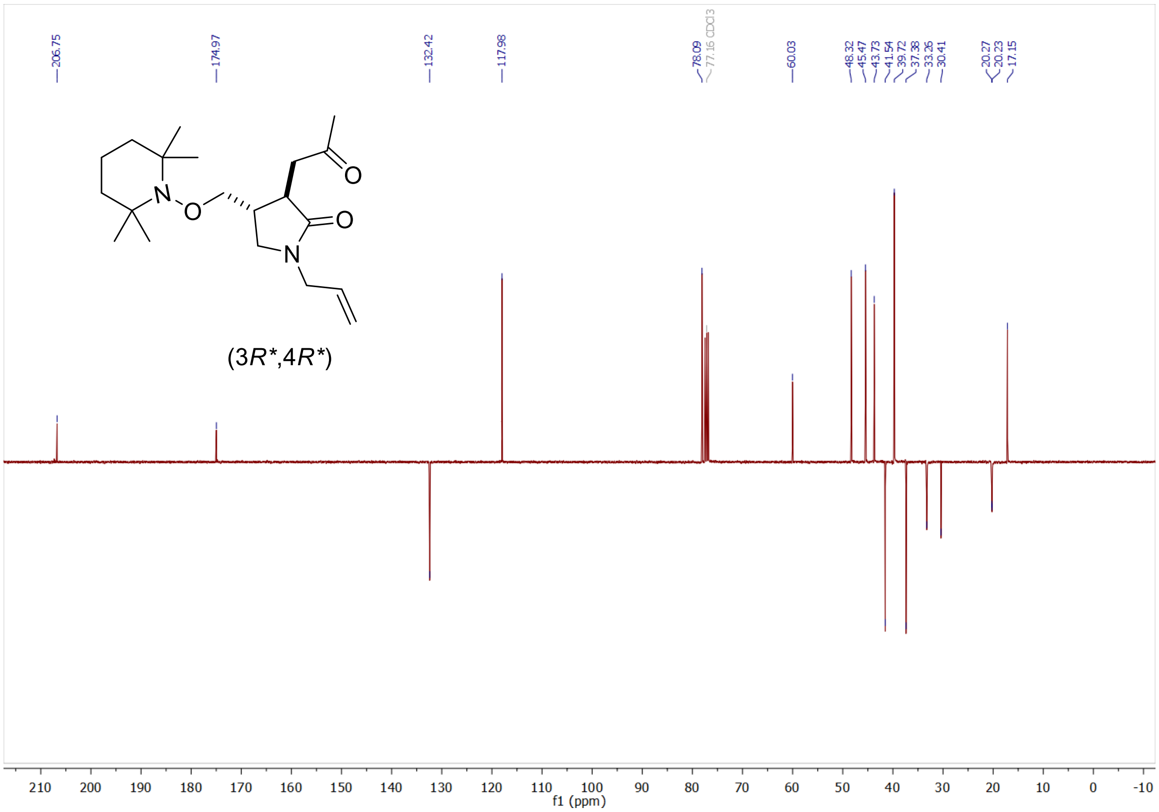

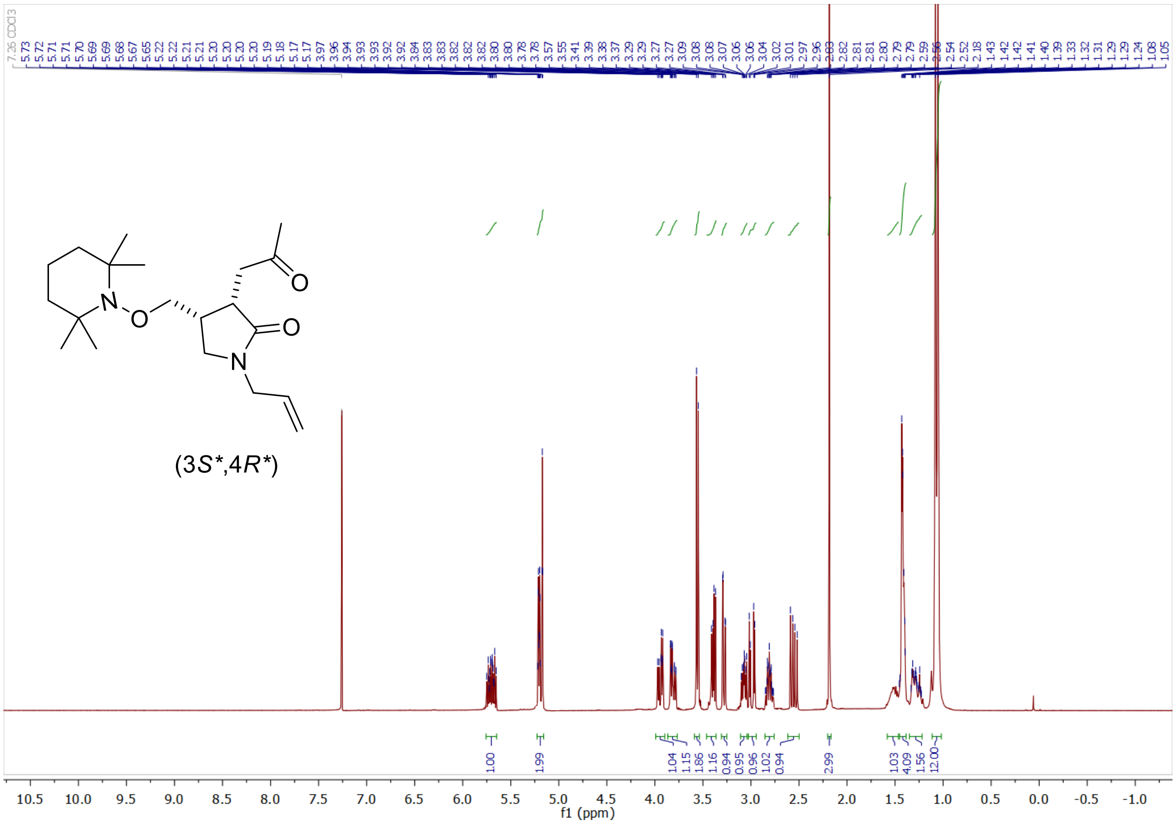

(3S*,4R*)
f1 (ppm)

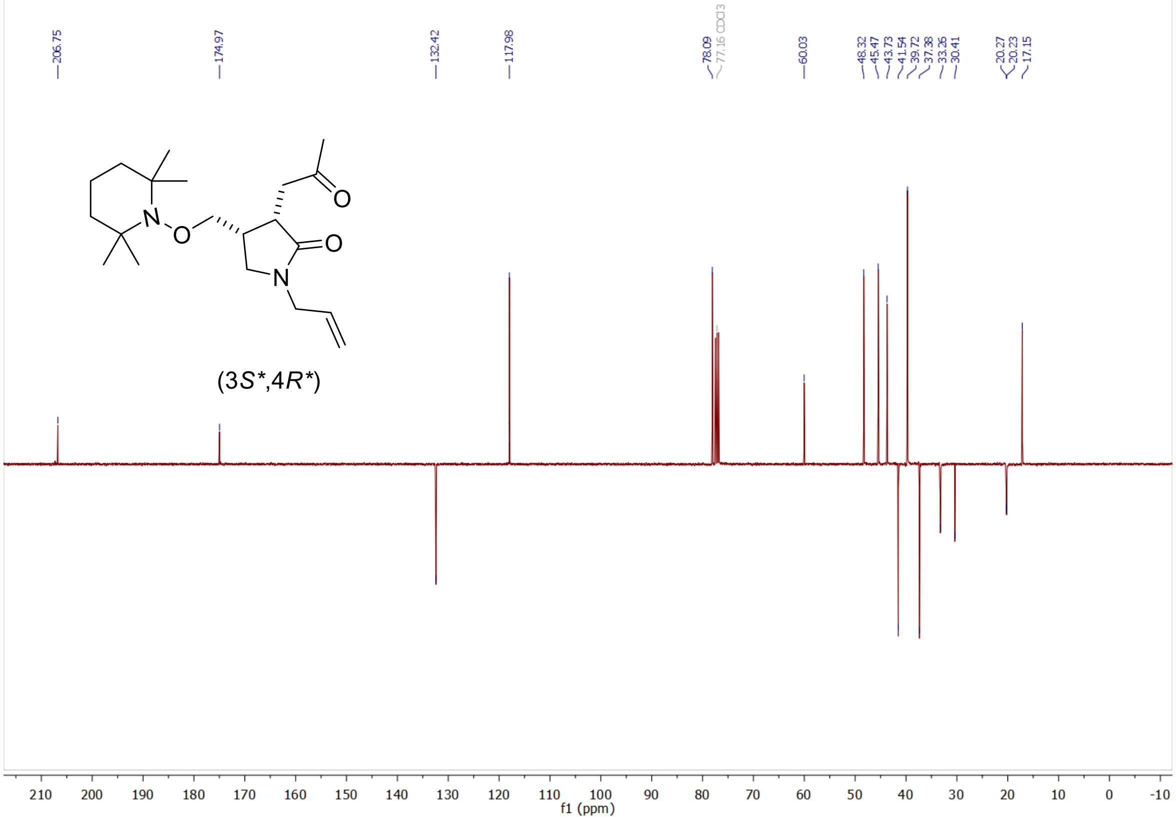

206.75
174.97
132.42
117.98
78.09
77.16 CDCl3
60.03
48.32
45.47
43.73
41.54
39.72
37.38
33.26
30.41
20.27
20.23
17.15
(3S*,4R*)
f1 (ppm)

(3*R,4*R**)-1-Allyl-3-(2-oxohexyl)-4-(((2,2,6,6-tetramethylpiperidin-1-yl)oxy)methyl)pyrrolidin-2-one (13c)**

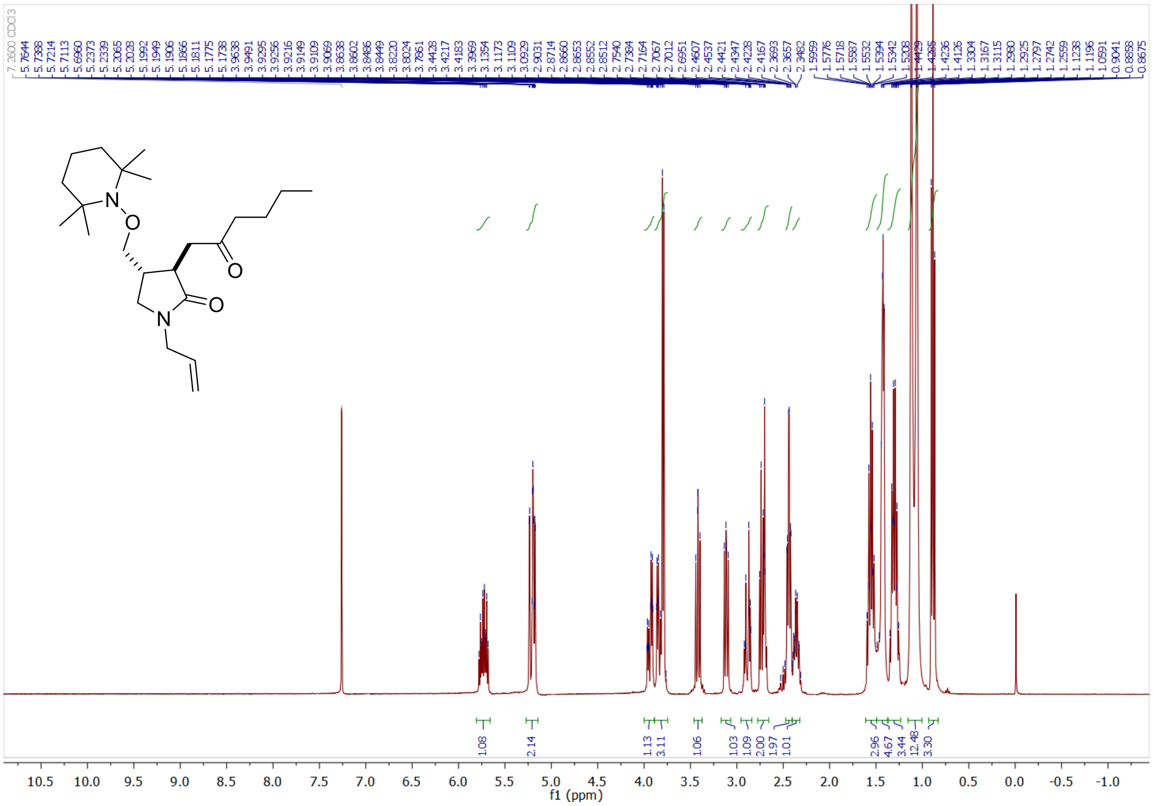

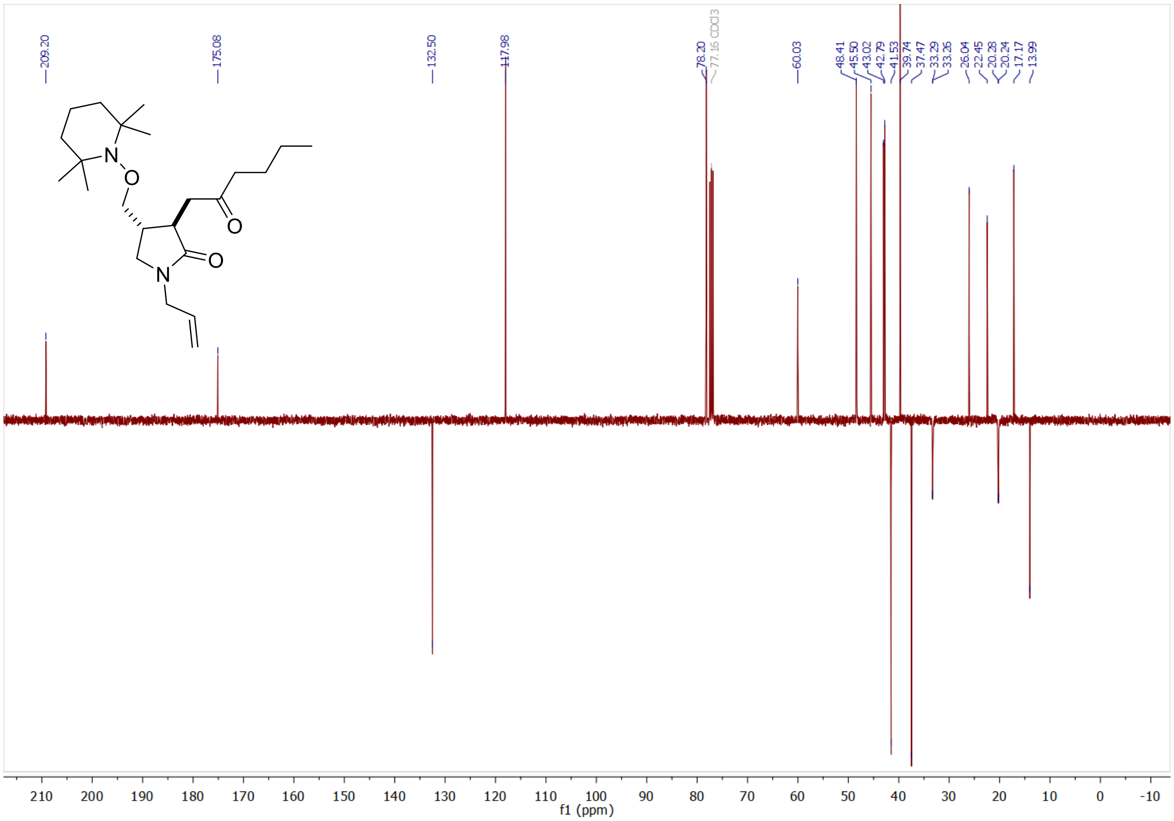

(3*R,4*R**)-1-Allyl-3-(2-oxo-2-phenylethyl)-4-(((2,2,6,6-tetramethylpiperidin-1-yl)oxy)methyl)pyrrolidin-2-one (13d)**

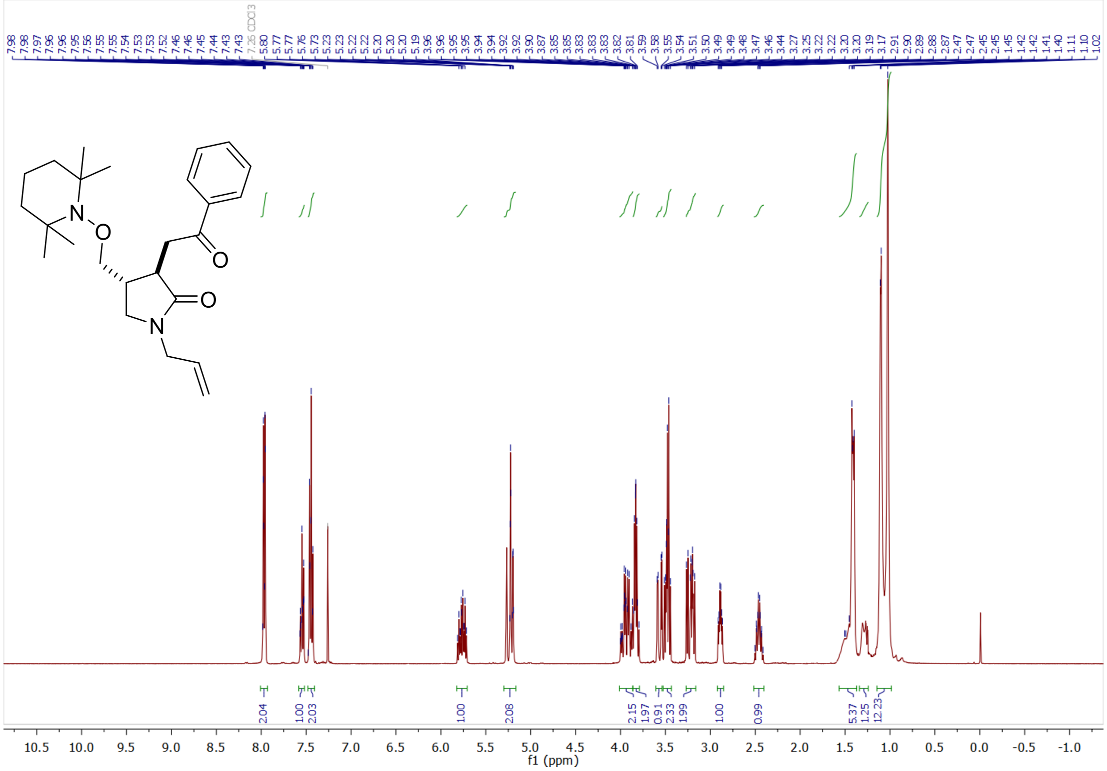

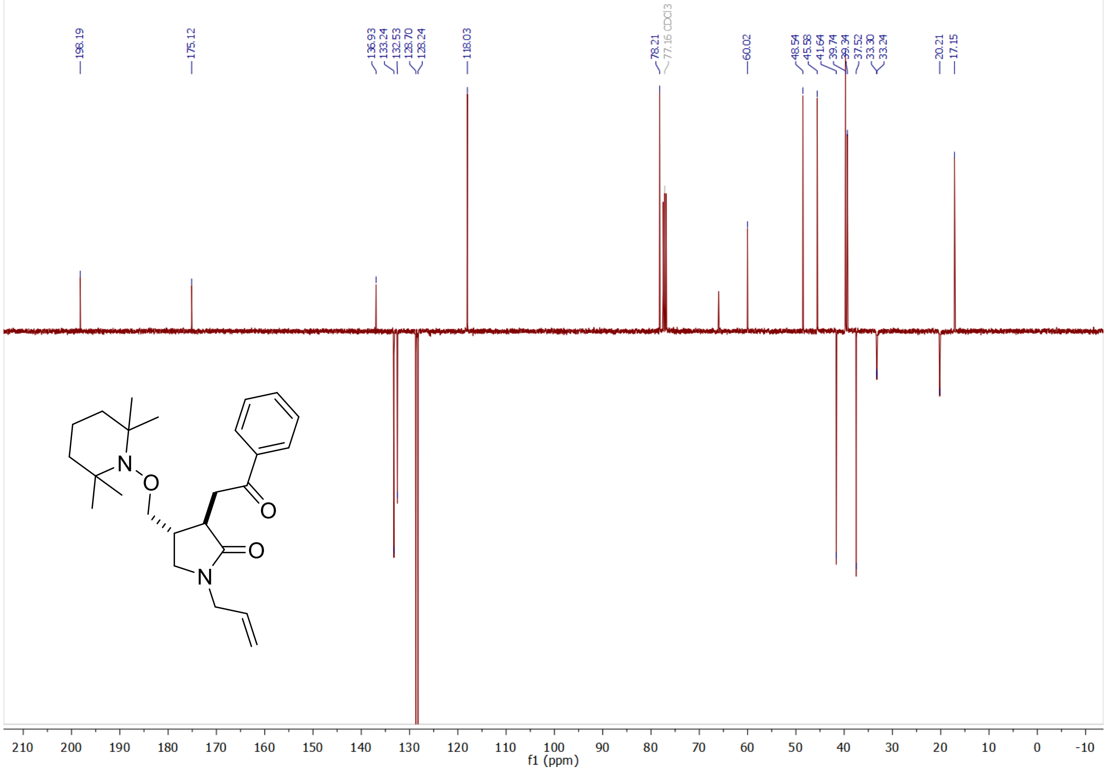

(3*R,4*R**)- and (3*S**,4*R**)-1-Benzyl-4-methyl-3-(2-oxopropyl)-4-(((2,2,6,6-tetramethyl-piperidin-1-yl)oxy)methyl)pyrrolidin-2-one (13f)**

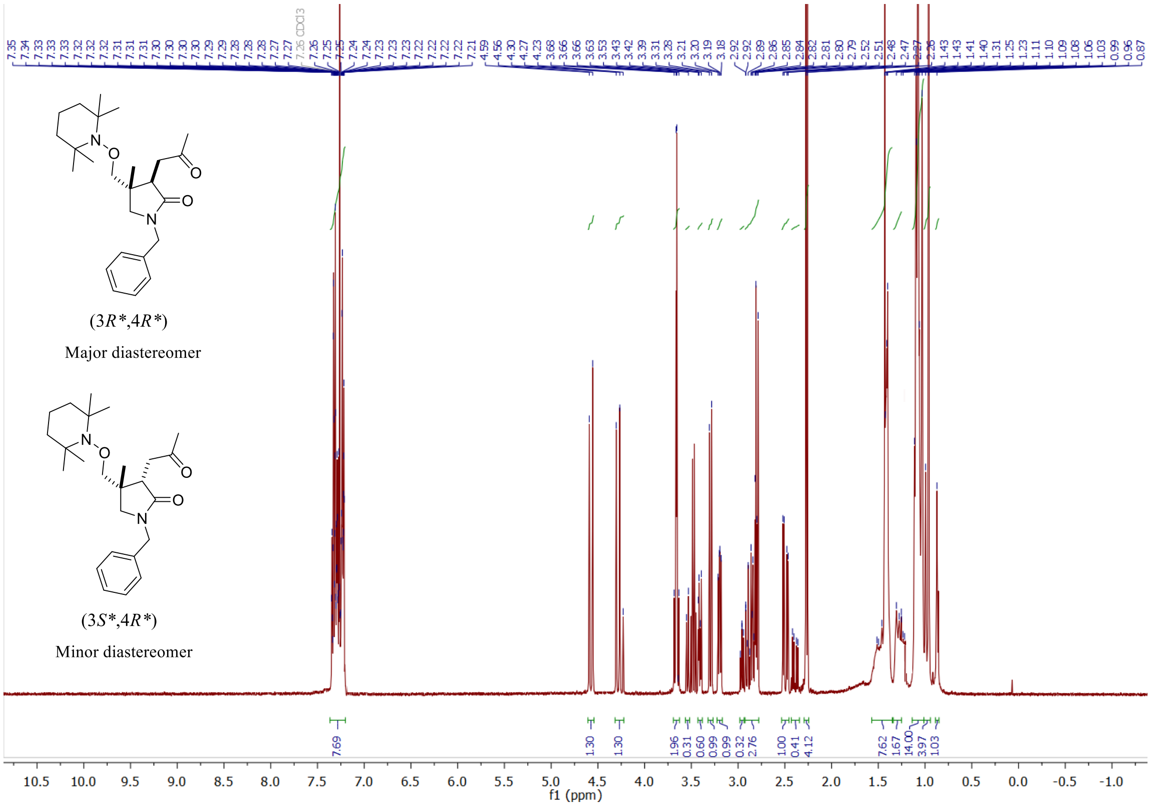

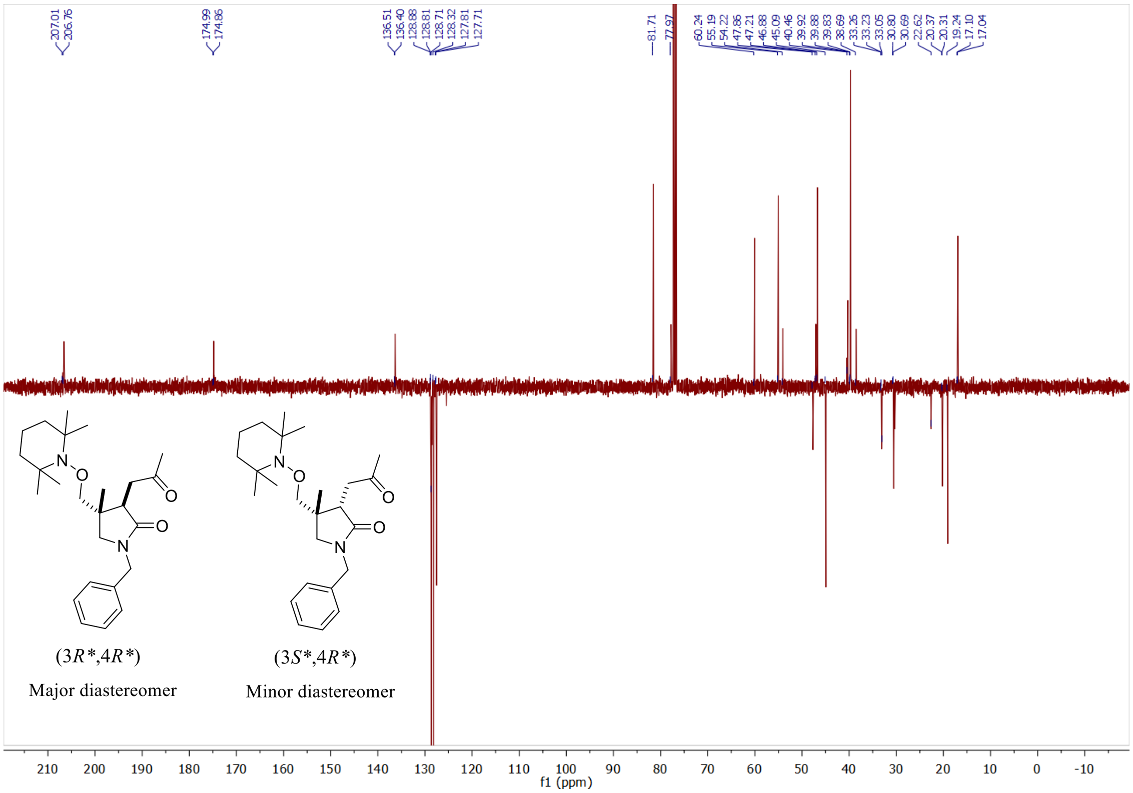

(3*R*,4*R*)- and (3*S*,4*S*)-3-(2-Oxopropyl)-1-((*S*)-1-phenylethyl)-4-(((2,2,6,6-tetramethyl-piperidin-1-yl)oxy)methyl)pyrrolidin-2-one (13i)

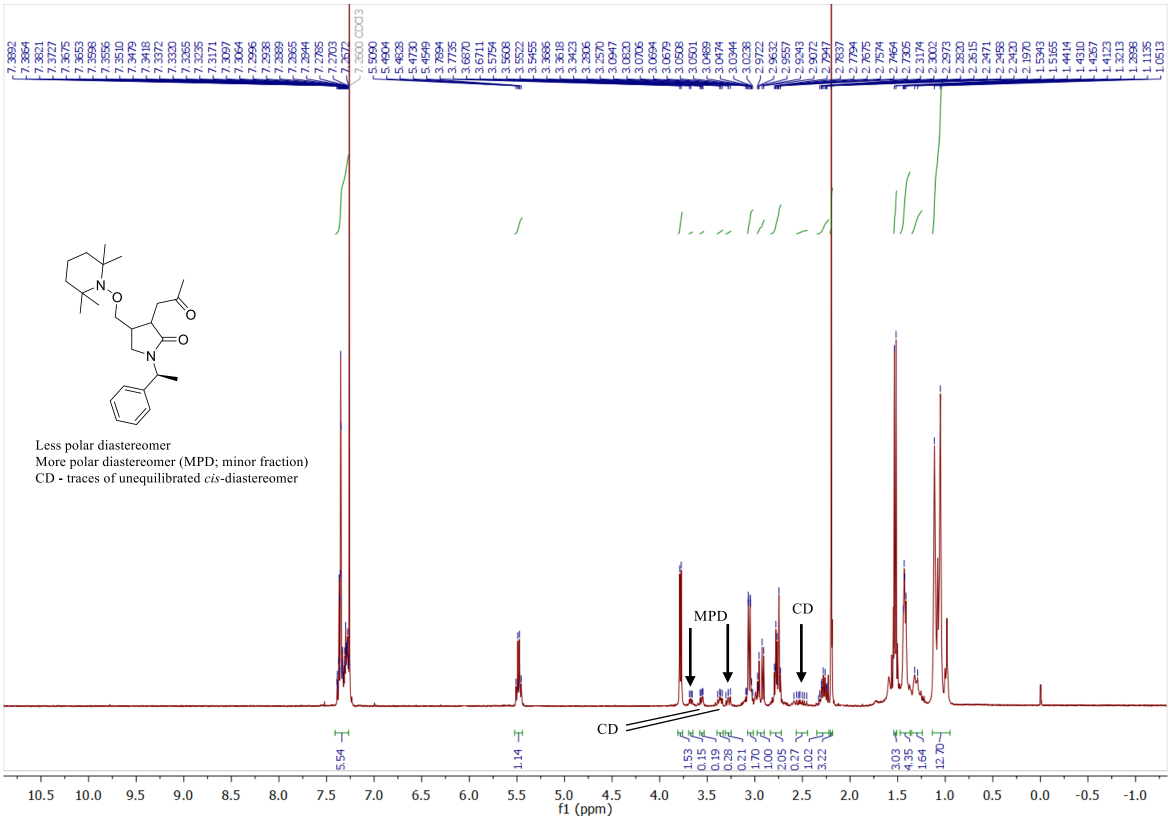

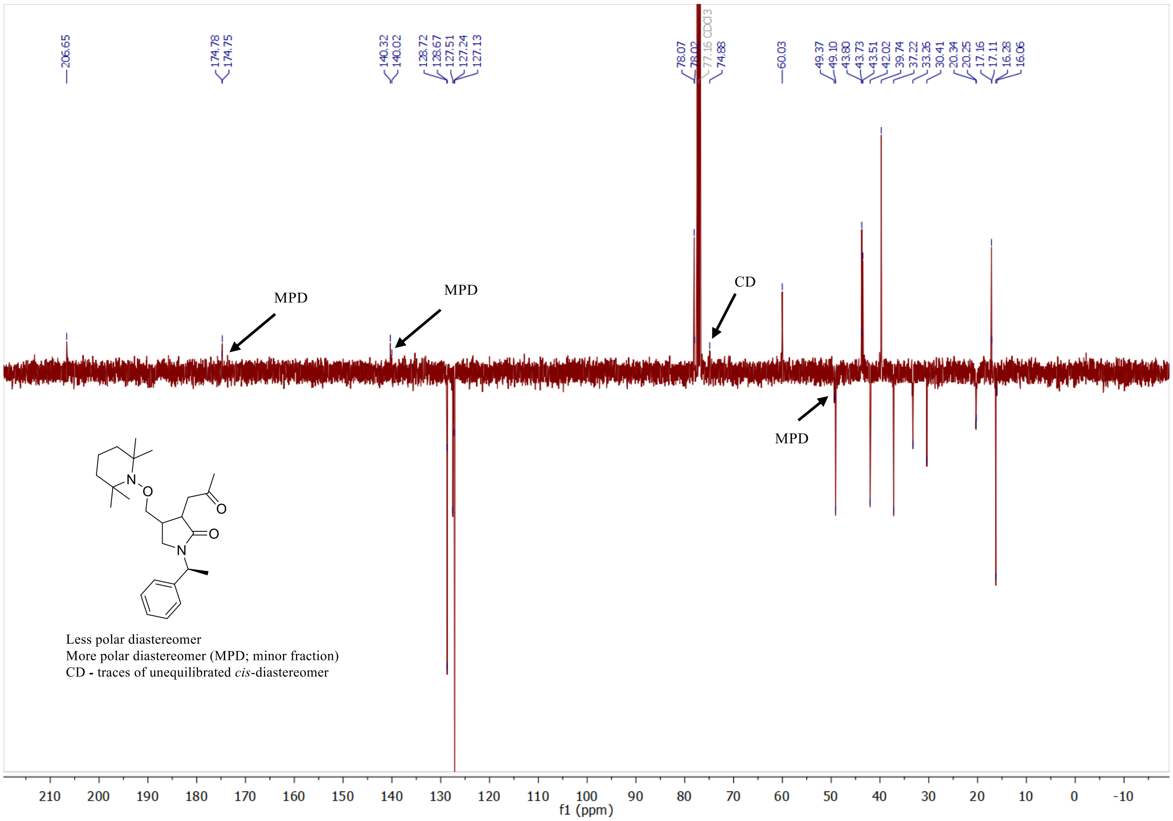

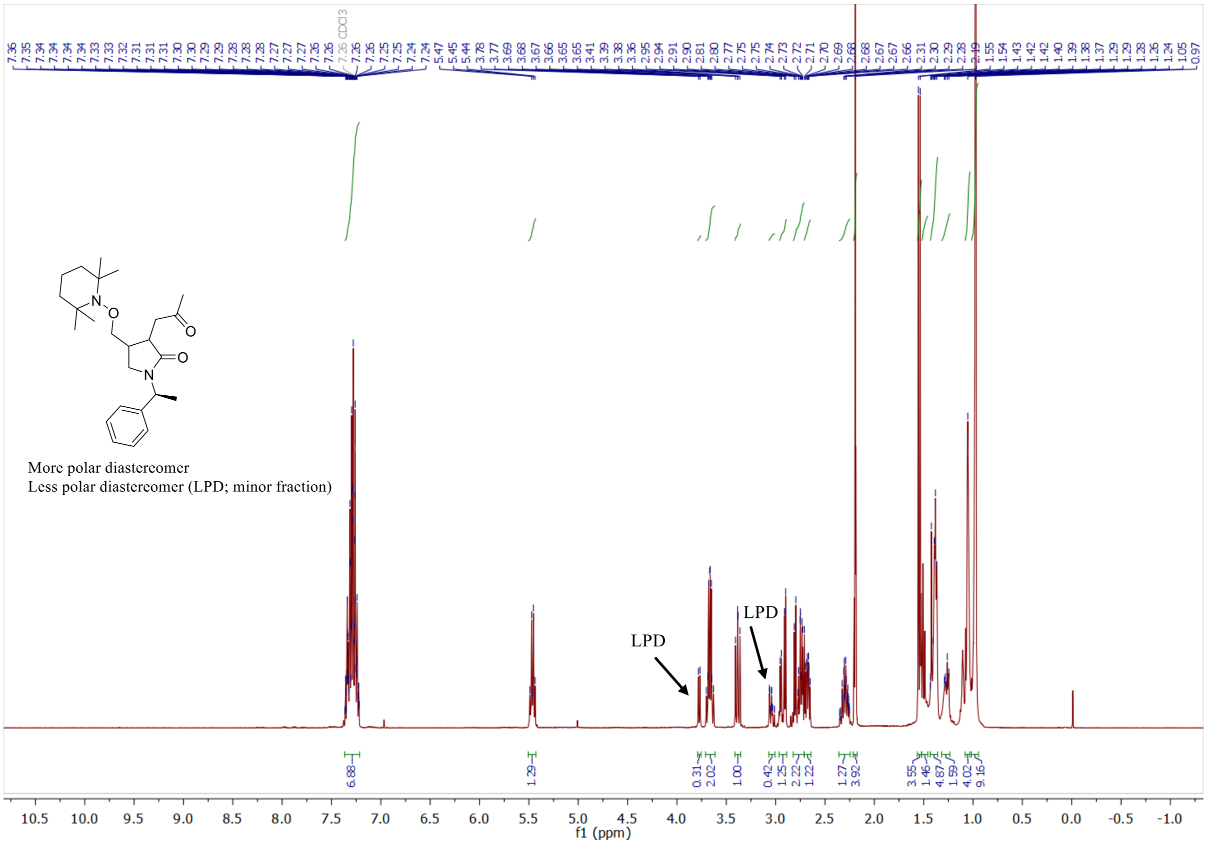
7.36 7.35 7.34 7.34 7.34 7.34 7.33 7.32 7.31 7.31 7.31 7.30 7.30 7.29 7.29 7.29 7.28 7.28 7.28 7.27 7.27 7.27 7.26 7.26 7.26 CDCl3 7.26 7.26 7.25 7.25 7.24 5.47 5.45 5.44 3.78 3.77 3.69 3.68 3.67 3.66 3.65 3.65 3.41 3.39 3.38 3.36 3.36 2.95 2.94 2.91 2.90 2.81 2.80 2.77 2.75 2.75 2.74 2.73 2.72 2.71 2.70 2.69 2.68 2.68 2.67 2.67 2.66 2.31 2.30 2.29 2.28 2.19 1.55 1.54 1.43 1.42 1.40 1.39 1.38 1.37 1.29 1.29 1.28 1.26 1.24 1.05 0.97
N
O
O
O
N
More polar diastereomer
Less polar diastereomer (LPD; minor fraction)
LPD
LPD
6.88 1.29 0.31 2.02 1.00 0.42 1.25 2.22 1.22 1.27 3.92 3.55 1.46 4.87 1.59 4.02 9.16
10.5 10.0 9.5 9.0 8.5 8.0 7.5 7.0 6.5 6.0 5.5 5.0 4.5 4.0 3.5 3.0 2.5 2.0 1.5 1.0 0.5 0.0 -0.5 -1.0
f1 (ppm)

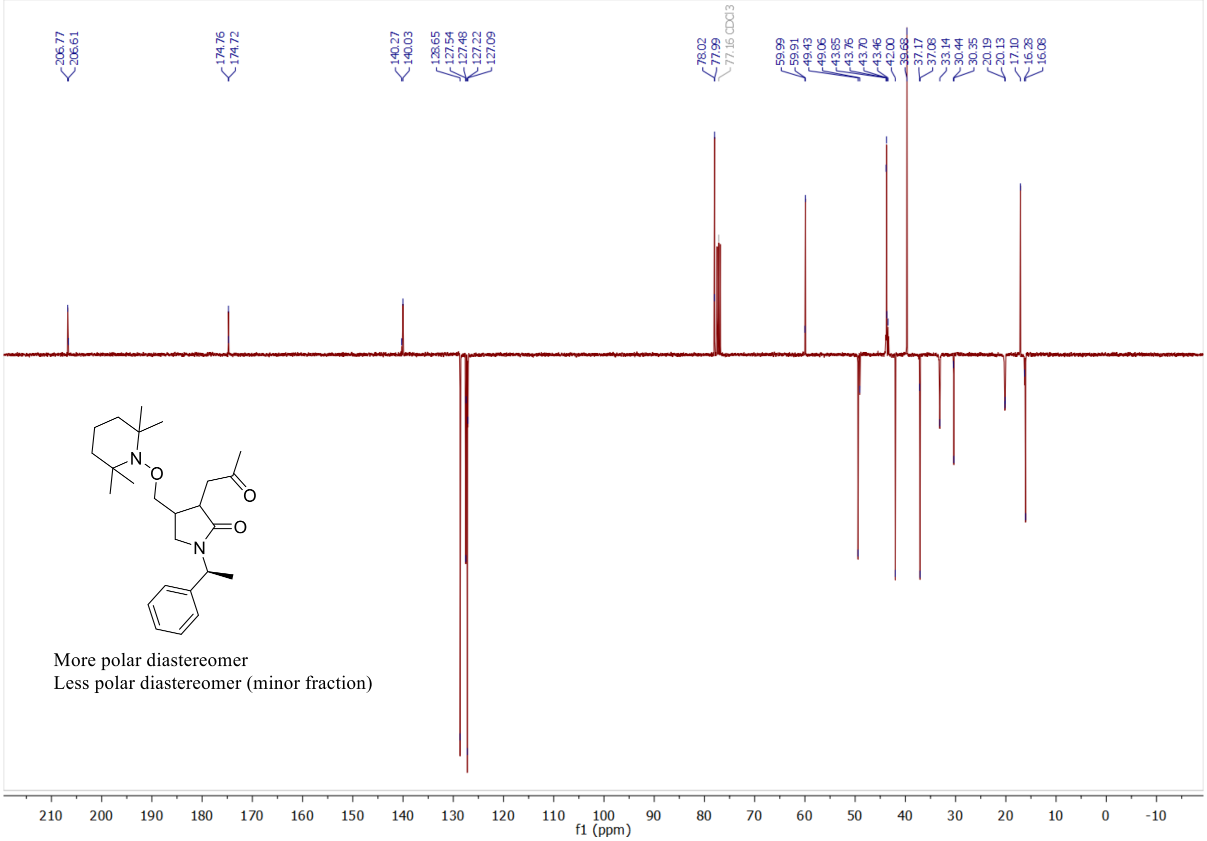
206.77 206.61 174.76 174.72 140.27 140.03 128.65 127.54 127.48 127.22 127.09 78.02 77.99 77.16 CDCl3 59.99 59.91 49.43 49.06 43.85 43.76 43.70 43.46 42.00 39.68 37.17 37.08 33.14 30.44 30.35 20.19 20.13 17.10 16.28 16.08
N
O
O
O
N
More polar diastereomer
Less polar diastereomer (minor fraction)
210 200 190 180 170 160 150 140 130 120 110 100 90 80 70 60 50 40 30 20 10 0 -10
f1 (ppm)

(3*R*,4*R*)- and (3*S*,4*S*)- and (3*S*,4*R*)- and (3*R*,4*S*)-1-((*S*)-1-(Naphthalen-2-yl)ethyl)-3-(2-oxopropyl)-4-(((2,2,6,6-tetramethylpiperidin-1-yl)oxy)methyl)pyrrolidin-2-one (13j)

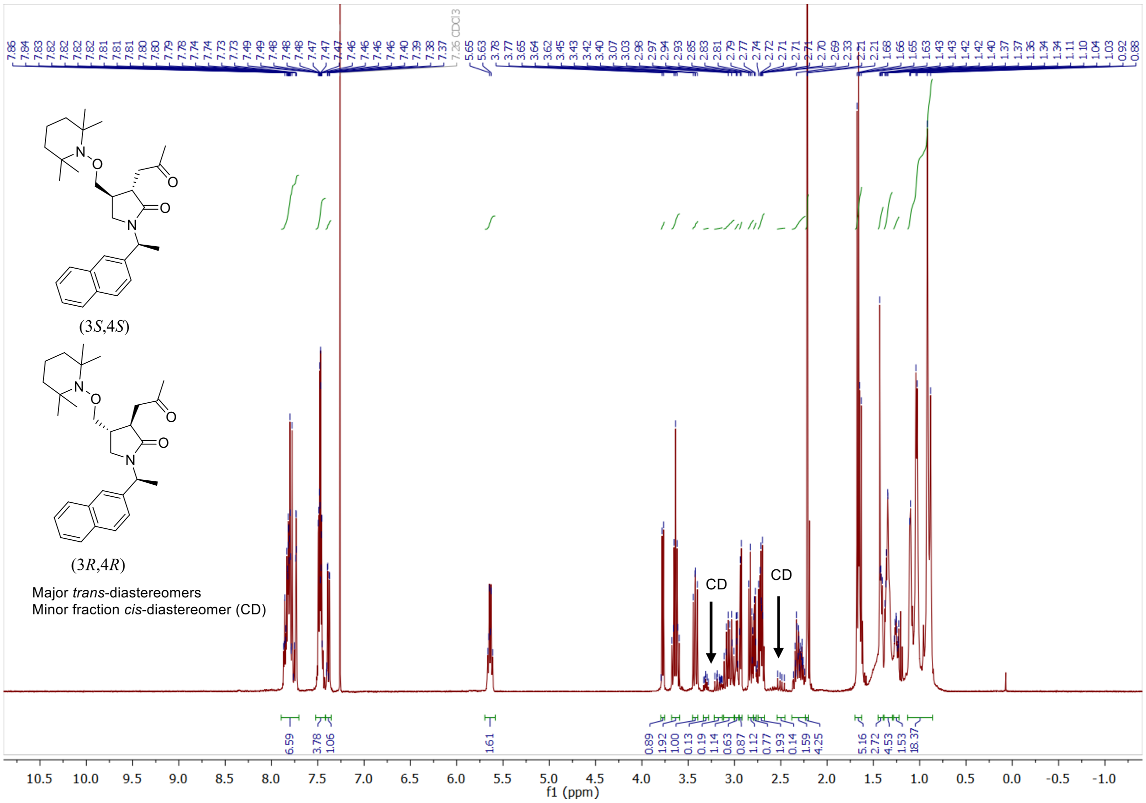

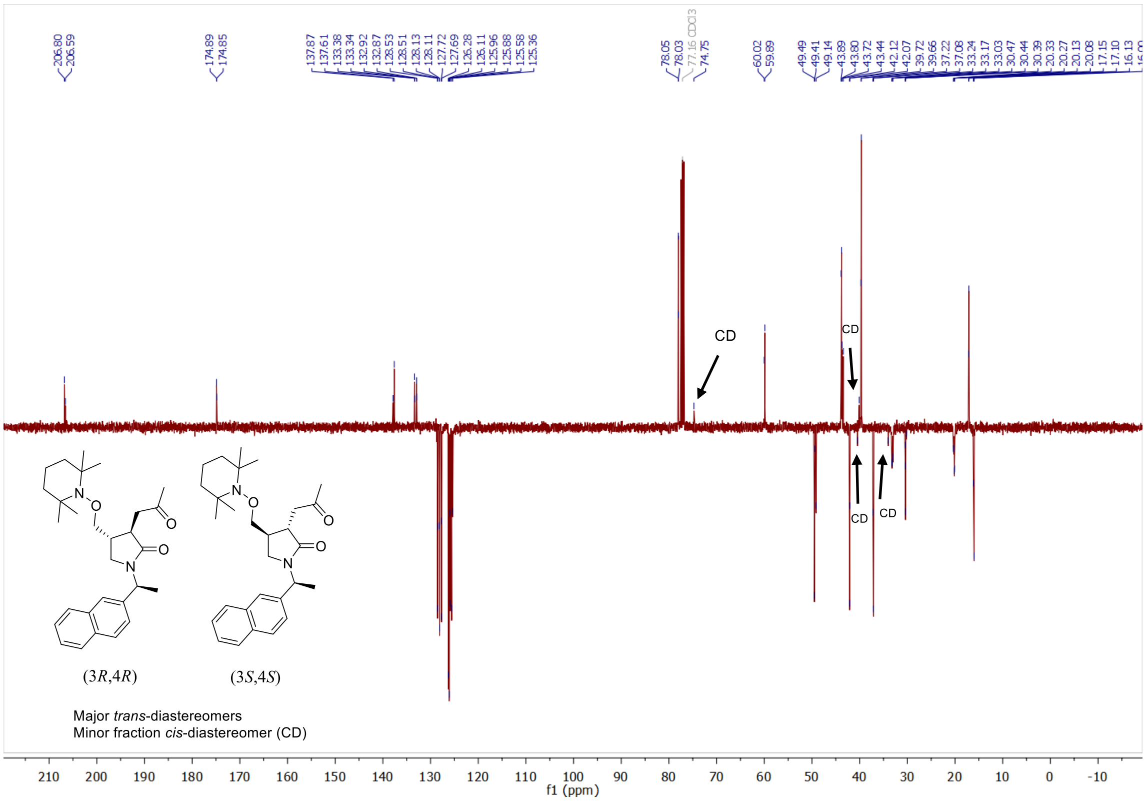

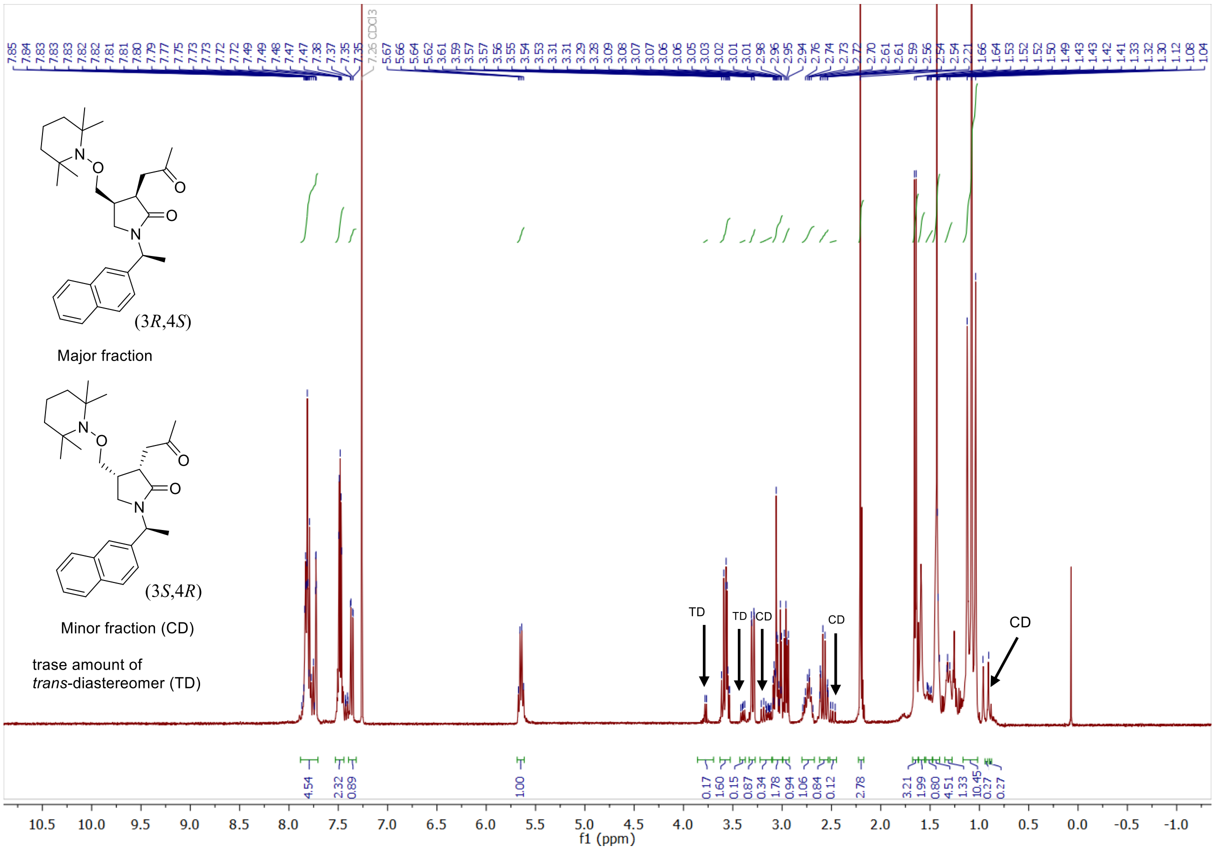
(3R,4S)
Major fraction
(3S,4R)
Minor fraction (CD)
trase amount of
trans-diastereomer (TD)
TD
TD
CD
CD
CD
f1 (ppm)

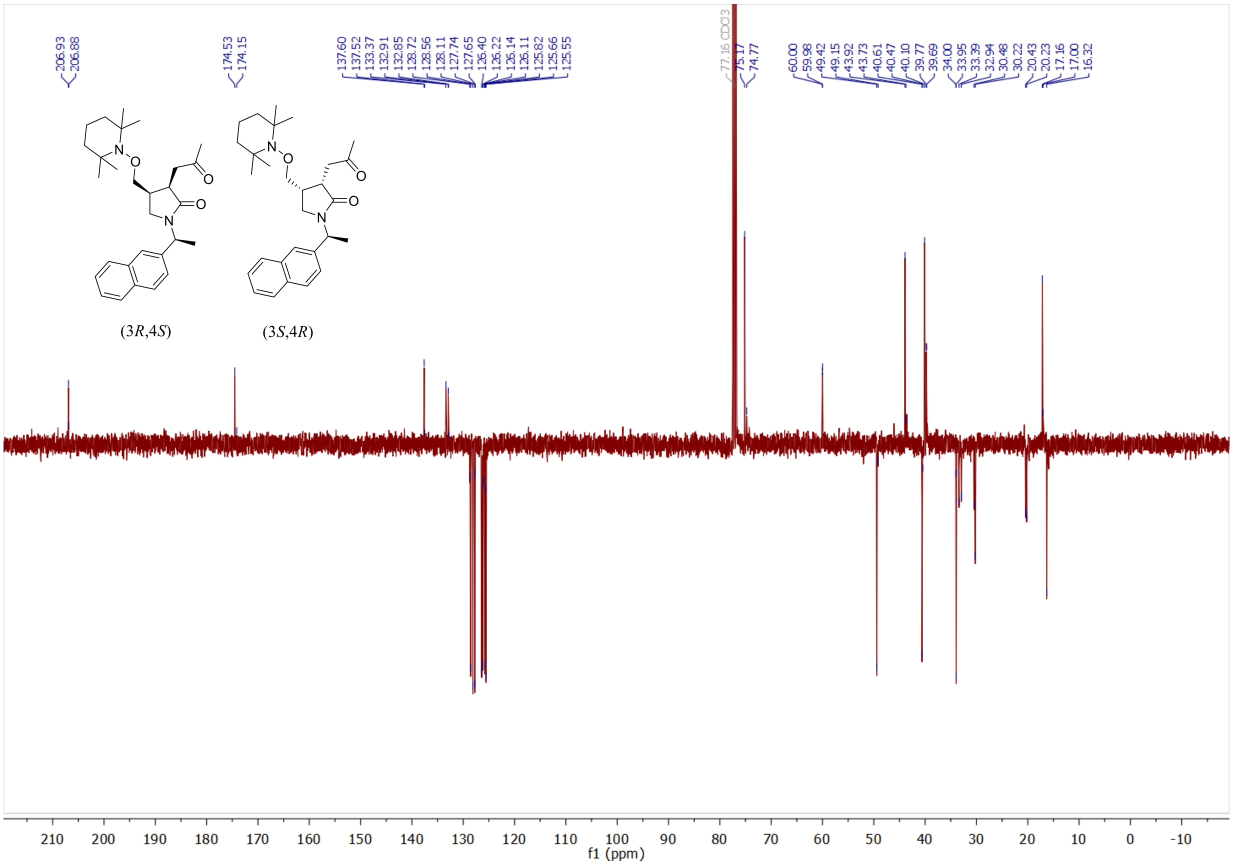
(3R,4S)
(3S,4R)
f1 (ppm)

(3*R,4*S**)- and (3*S**,4*R**)- and (3*R**,4*R**)-1-Allyl-3-((*R**)-2-oxocyclohexyl)-4-(((2,2,6,6-tetramethylpiperidin-1-yl)oxy)methyl)pyrrolidin-2-one (13l)**

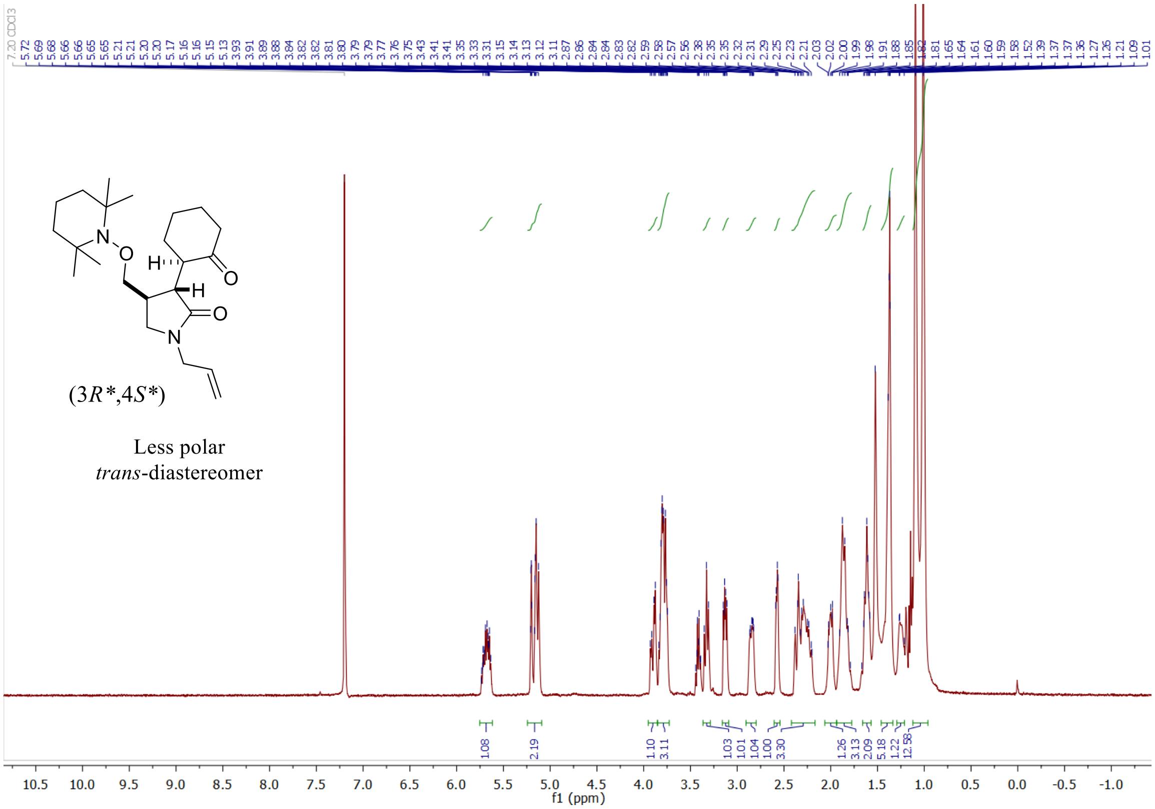

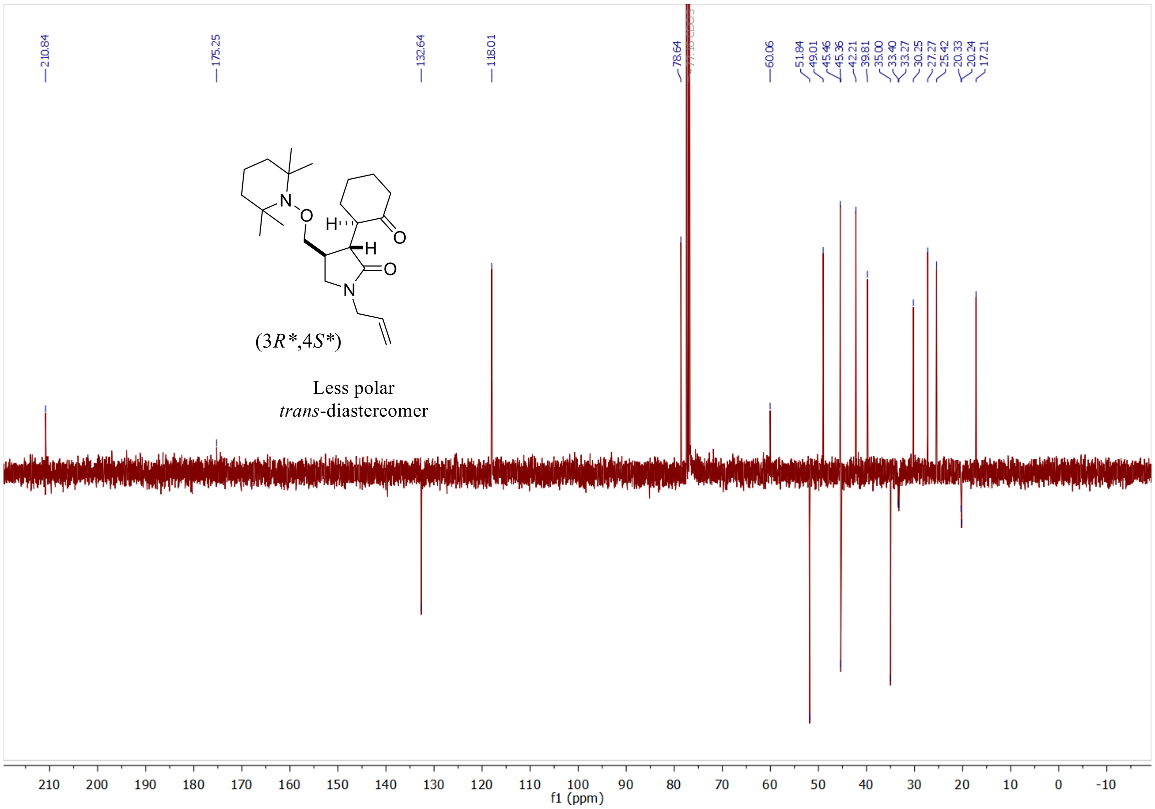

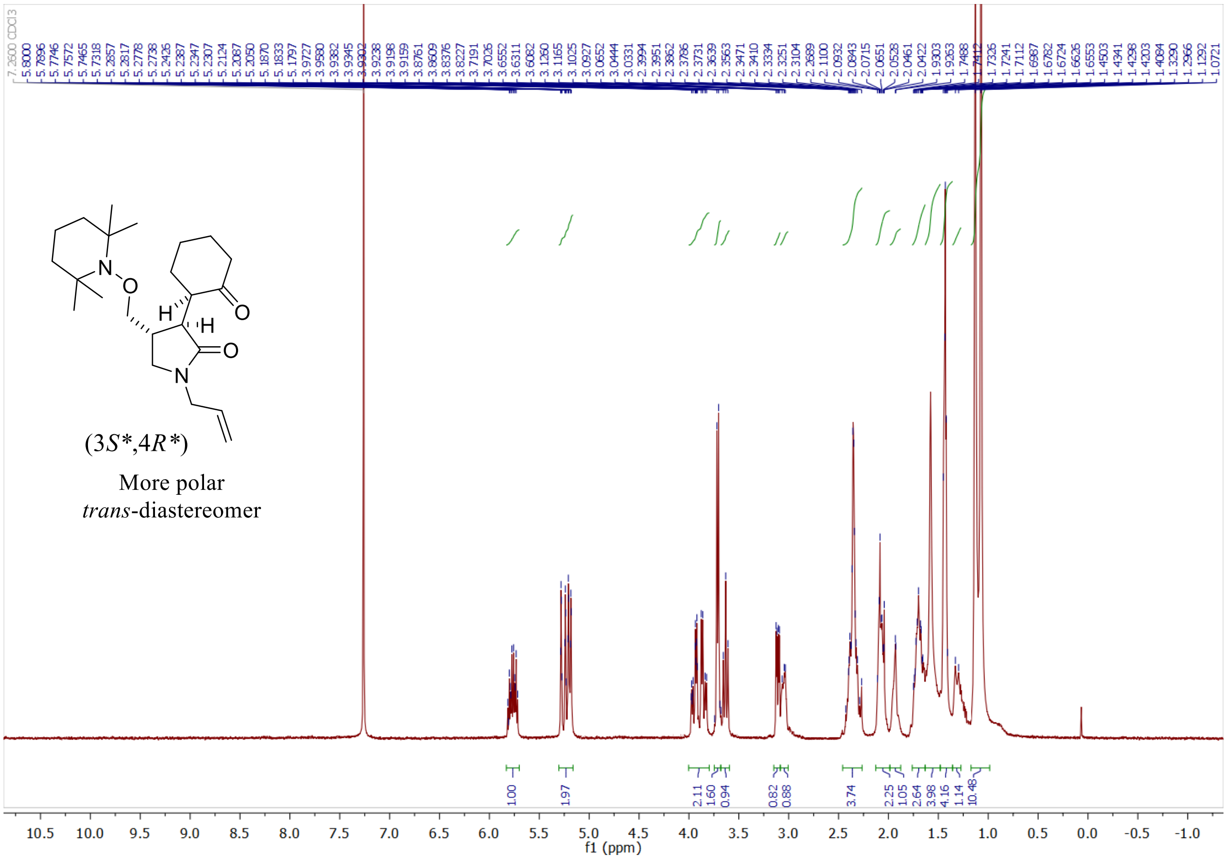

(3S*,4R*)
More polar
trans-diastereomer
f1 (ppm)

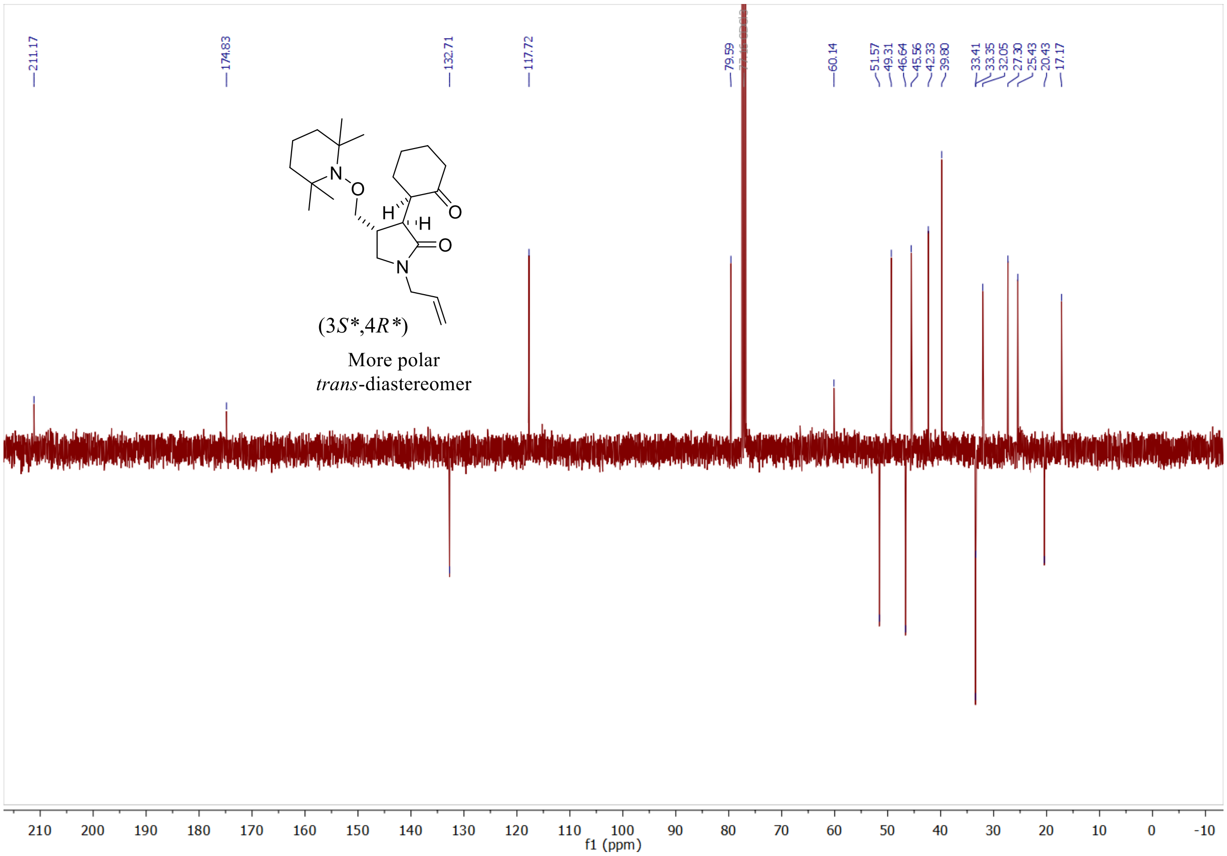

211.17
174.83
132.71
117.72
79.59
60.14
51.57
49.31
46.64
45.56
42.33
39.80
33.41
33.35
32.05
27.30
25.43
20.43
17.17
(3S*,4R*)
More polar
trans-diastereomer
f1 (ppm)

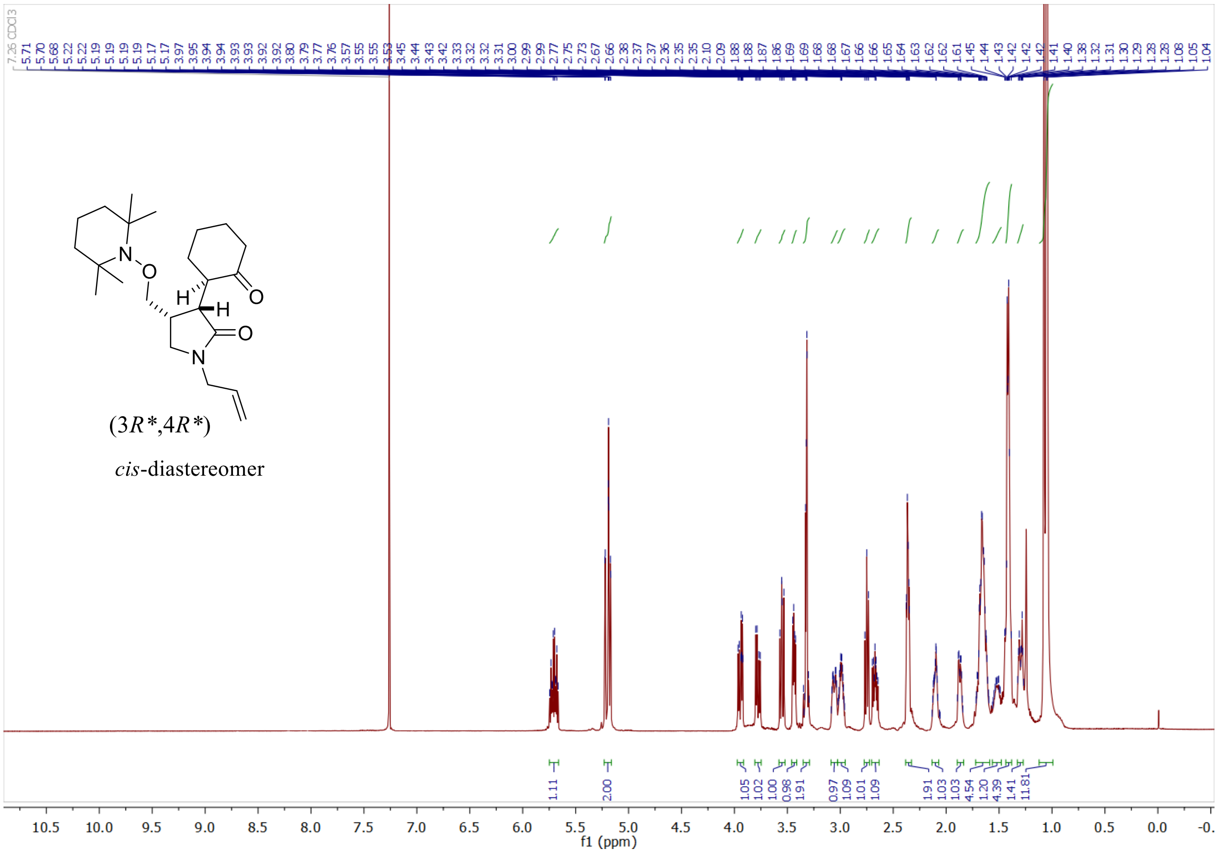
7.26 CDCl3
5.71 5.70 5.68 5.22 5.19 5.19 5.19 5.19 5.17 5.17 3.97 3.95 3.94 3.94 3.93 3.93 3.92 3.92 3.80 3.79 3.77 3.76 3.57 3.55 3.55 3.53 3.45 3.44 3.43 3.42 3.33 3.32 3.32 3.31 3.00 2.99 2.99 2.77 2.75 2.73 2.67 2.66 2.38 2.37 2.37 2.36 2.35 2.35 2.10 2.09 1.88 1.88 1.87 1.86 1.69 1.69 1.68 1.67 1.66 1.66 1.65 1.64 1.63 1.62 1.62 1.61 1.45 1.44 1.43 1.42 1.42 1.42 1.41 1.40 1.38 1.32 1.31 1.30 1.29 1.28 1.28 1.08 1.05 1.04
1.11 2.00 1.05 1.02 1.00 0.98 1.91 0.97 1.09 1.01 1.09 1.91 1.03 1.03 4.54 1.20 4.39 1.41 11.81
(3R*,4R*)
cis-diastereomer
f1 (ppm)

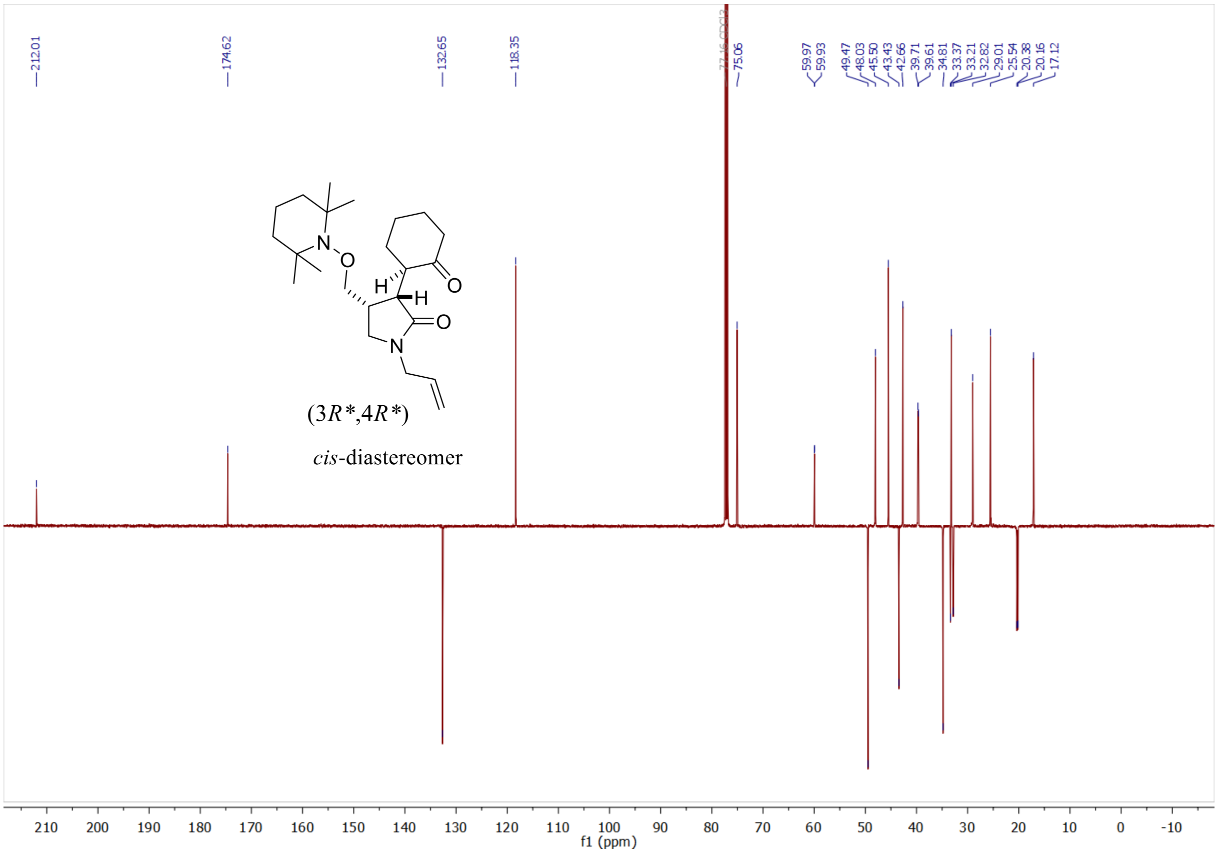
212.01 174.62 132.65 118.35 77.16 CDCl3 75.06 59.97 59.93 49.47 48.03 45.50 43.43 42.66 39.71 39.61 34.81 33.37 33.21 32.82 29.01 25.54 20.38 20.16 17.12
(3R*,4R*)
cis-diastereomer
f1 (ppm)

(4*S,5*S**,6*R**)- and (4*S**,5*S**,6*S**)- and (4*R**,5*S**,6*R**)- and (4*R**,5*S**,6*S**)-2-Benzyl-4-(2-oxopropyl)-6-((2,2,6,6-tetramethylpiperidin-1-yl)oxy)-2-azaspiro[4.4]nonan-3-one (13m)**

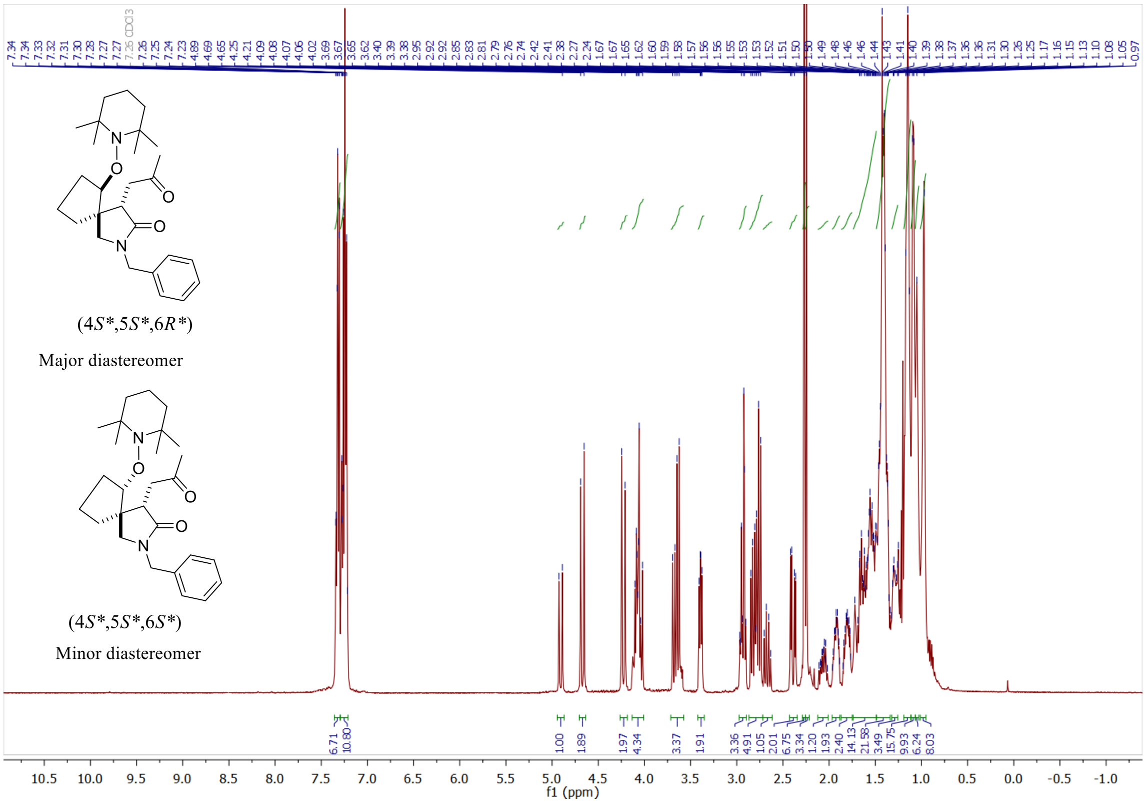

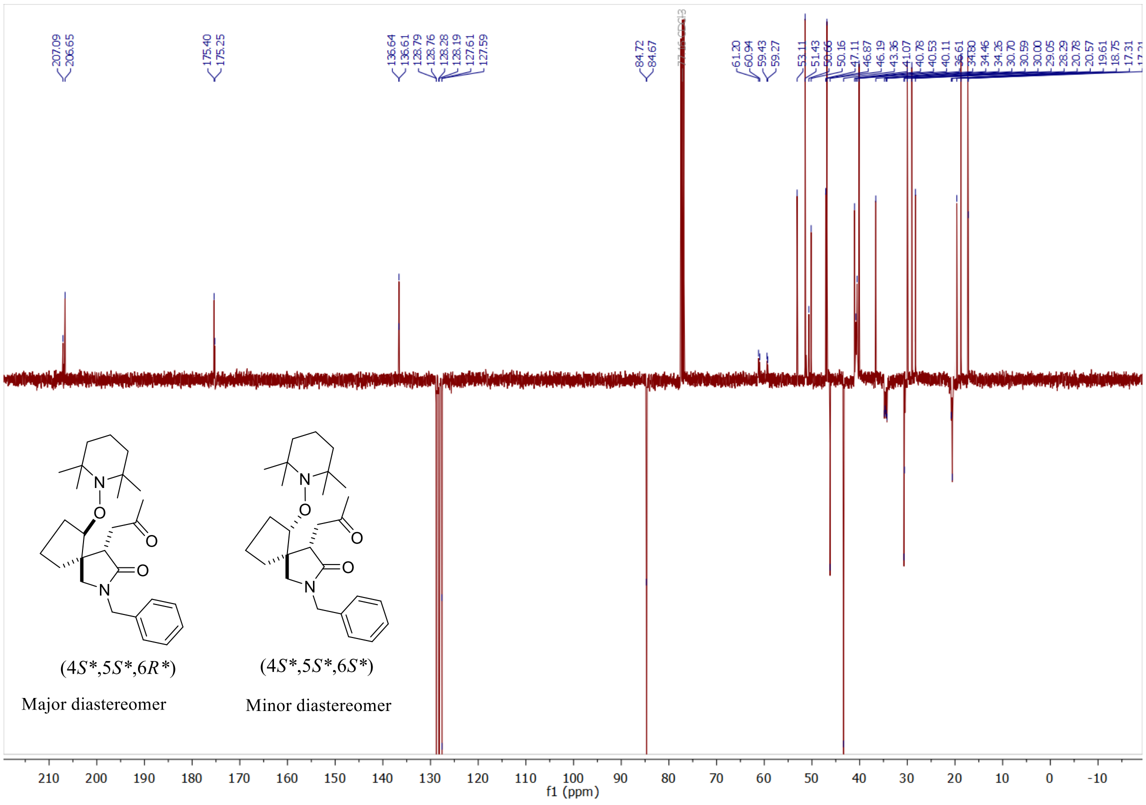

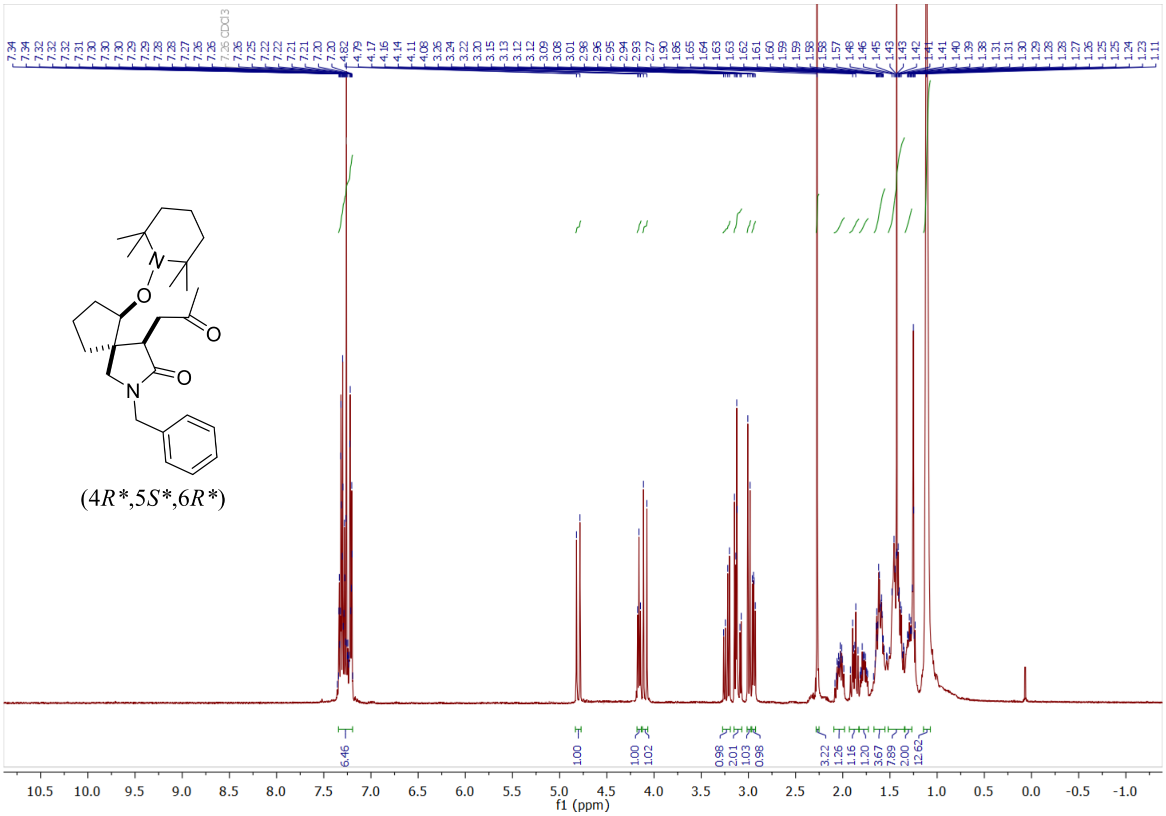
7.34 7.34 7.32 7.32 7.31 7.30 7.30 7.29 7.29 7.28 7.28 7.27 7.26 7.26 CDCl3 7.26 7.25 7.22 7.22 7.21 7.21 7.20 7.20 4.82 4.79 4.17 4.16 4.14 4.11 4.08 3.26 3.24 3.22 3.20 3.15 3.13 3.12 3.12 3.09 3.08 3.01 2.98 2.96 2.95 2.94 2.93 2.27 1.90 1.86 1.65 1.64 1.63 1.63 1.62 1.61 1.60 1.59 1.59 1.58 1.58 1.57 1.48 1.46 1.45 1.43 1.43 1.42 1.41 1.41 1.40 1.39 1.38 1.31 1.31 1.30 1.29 1.28 1.28 1.27 1.26 1.25 1.25 1.24 1.23 1.11
(4R*,5S*,6R*)
6.46
1.00
1.00 1.02
0.98 2.01 1.03 0.98
3.22 1.26 1.16 1.20 3.67 7.89 2.00 12.62
f1 (ppm)

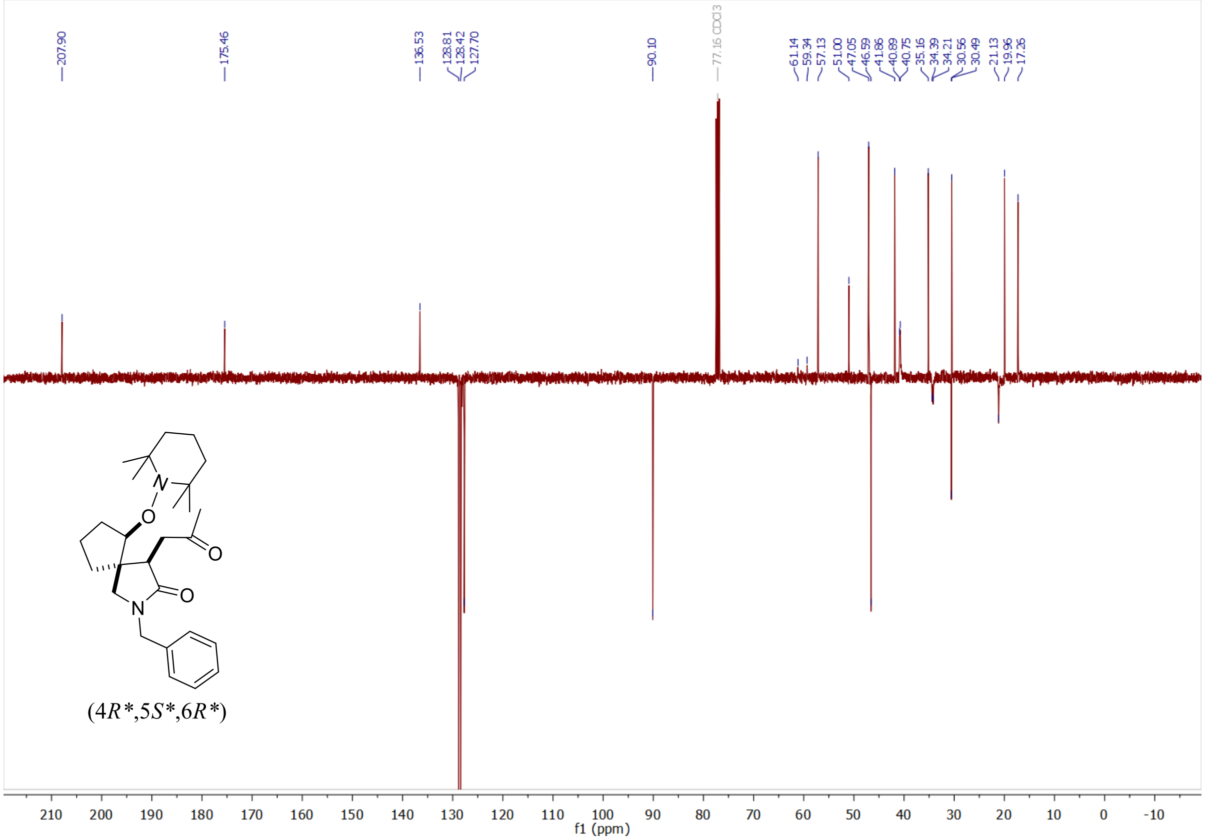
207.90
175.46
136.53
128.81 128.42 127.70
90.10
77.16 CDCl3
61.14 59.34 57.13 51.00 47.05 46.59 41.86 40.89 40.75 35.16 34.39 34.21 30.56 30.49 21.13 19.96 17.36
(4R*,5S*,6R*)
f1 (ppm)

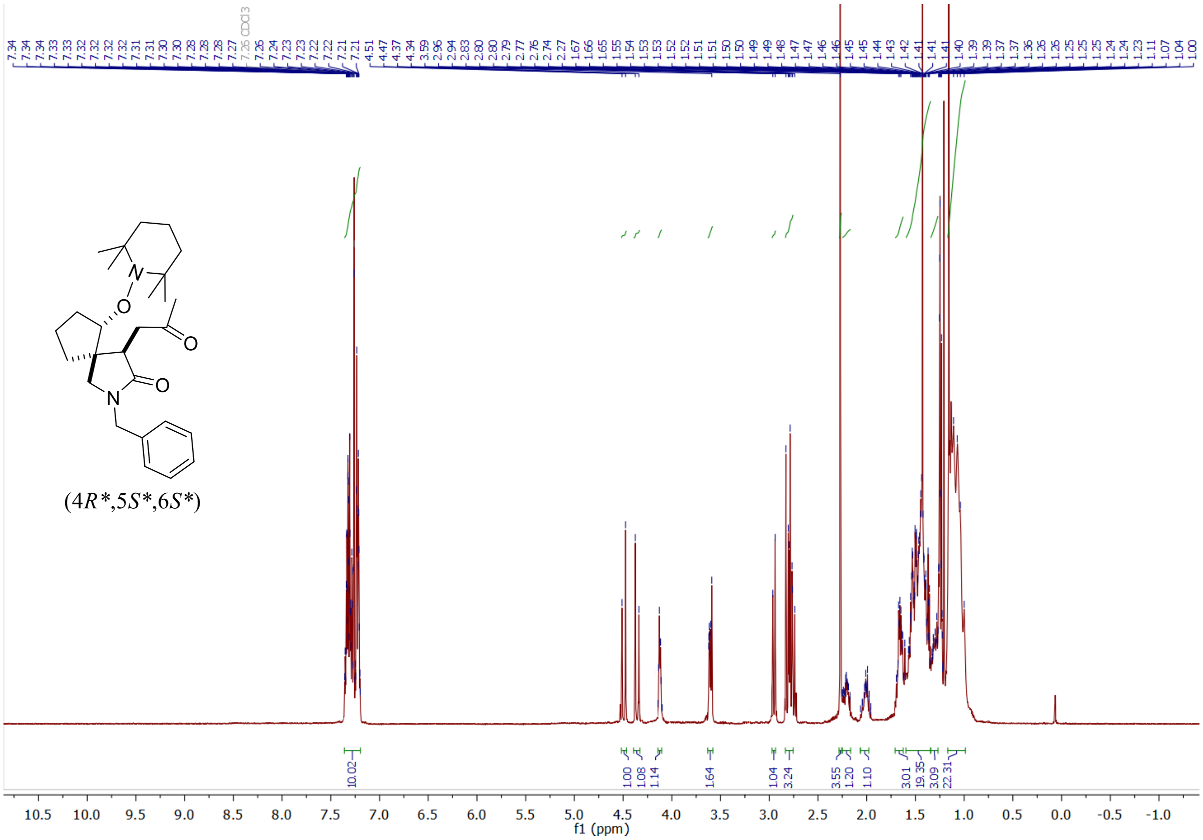
7.34 7.34 7.34 7.33 7.32 7.32 7.32 7.32 7.31 7.31 7.30 7.30 7.28 7.28 7.28 7.27 7.26 CDCl3 7.26 7.24 7.23 7.23 7.22 7.22 7.21 7.21 7.21
4.51 4.47 4.37 4.34 3.59 2.96 2.94 2.83 2.80 2.79 2.77 2.76 2.74 2.27 1.67 1.66 1.65 1.55 1.54 1.53 1.53 1.52 1.52 1.51 1.51 1.50 1.50 1.49 1.49 1.48 1.47 1.47 1.46 1.46 1.45 1.45 1.44 1.43 1.42 1.41 1.41 1.41 1.40 1.39 1.39 1.37 1.37 1.36 1.36 1.35 1.25 1.25 1.25 1.24 1.24 1.23 1.11 1.07 1.04 1.00
(4R*,5S*,6S*)
10.02 1.00 1.08 1.14 1.64 1.04 3.24 3.55 1.20 1.10 3.01 19.35 3.09 22.31
f1 (ppm)

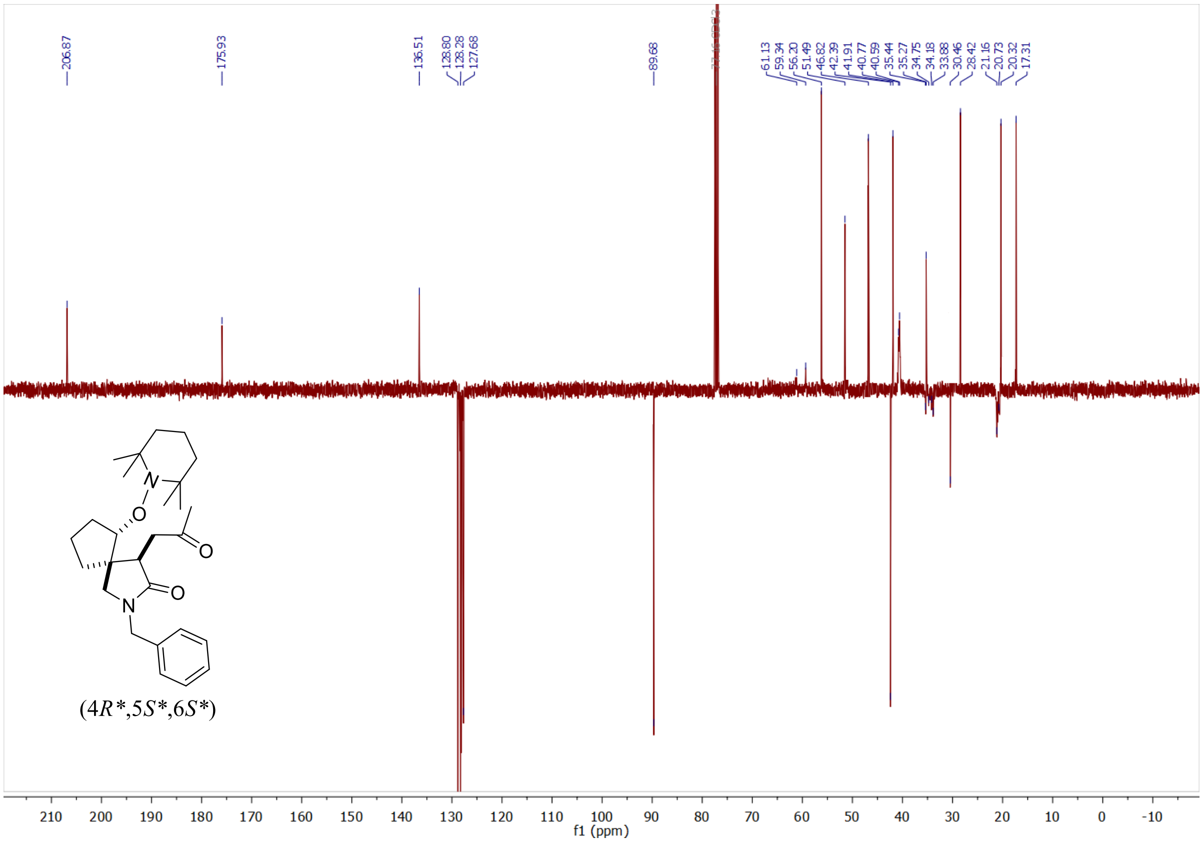
206.87 175.93 136.51 128.80 128.28 127.68 89.68 61.13 59.34 56.20 51.49 46.82 42.39 41.91 40.77 40.59 35.44 35.27 34.75 34.18 33.88 30.46 28.42 21.16 20.73 20.32 17.31
(4R*,5S*,6S*)
f1 (ppm)

(4*S,5*S**,6*R**)- and (4*R**,5*S**,6*R**)- and (4*S**,5*S**,6*S**)-2-Benzyl-4-(2-oxopropyl)-6-((2,2,6,6-tetramethylpiperidin-1-yl)oxy)-2-azaspiro[4.5]decan-3-one (13n)**

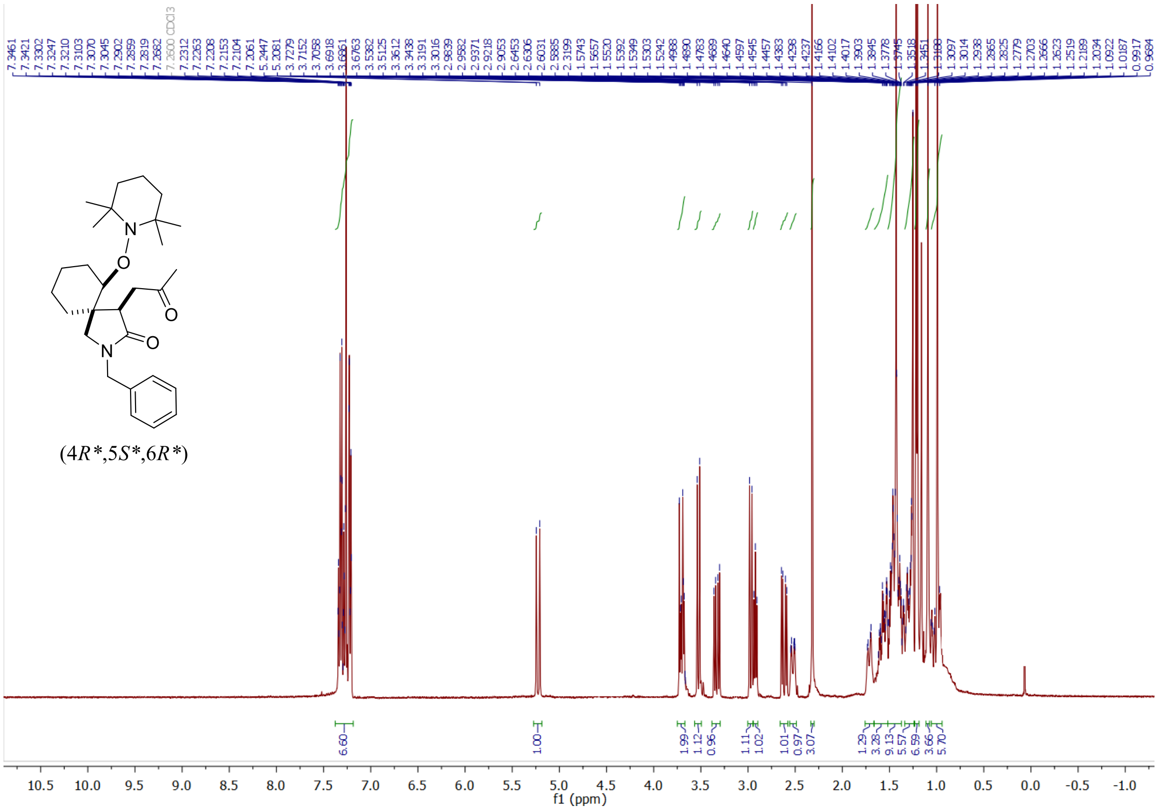

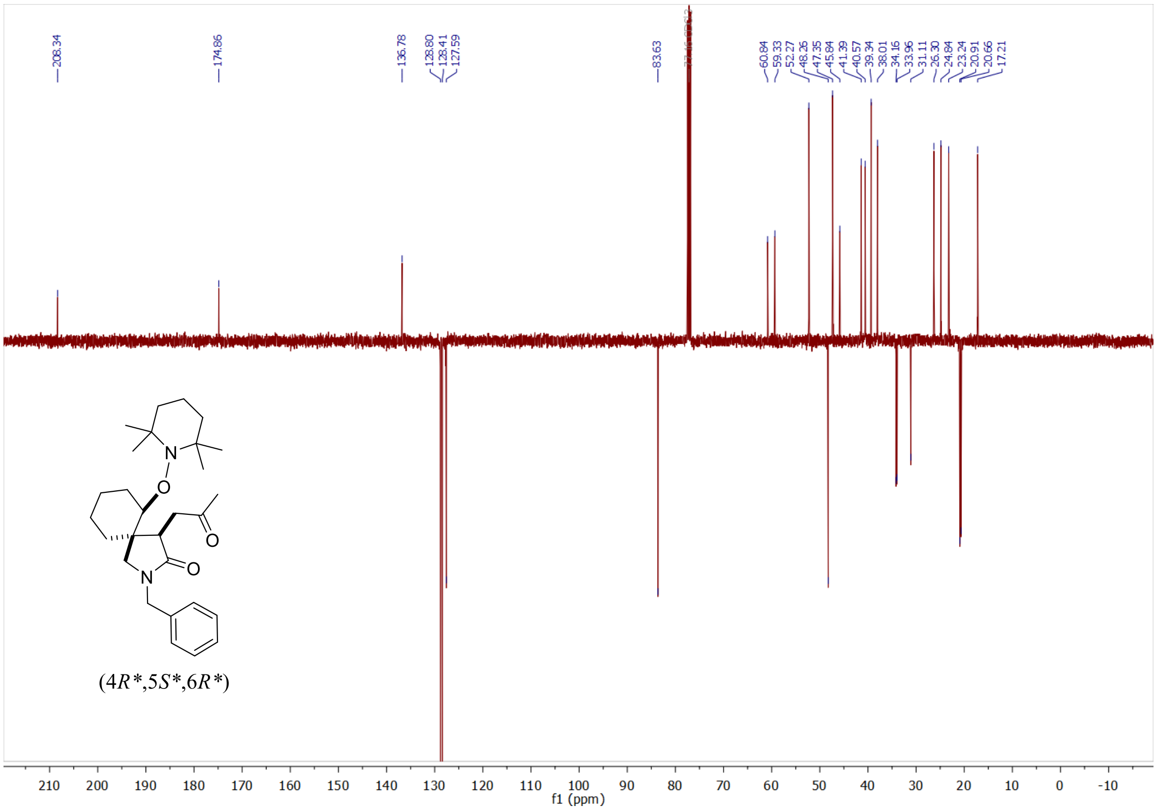

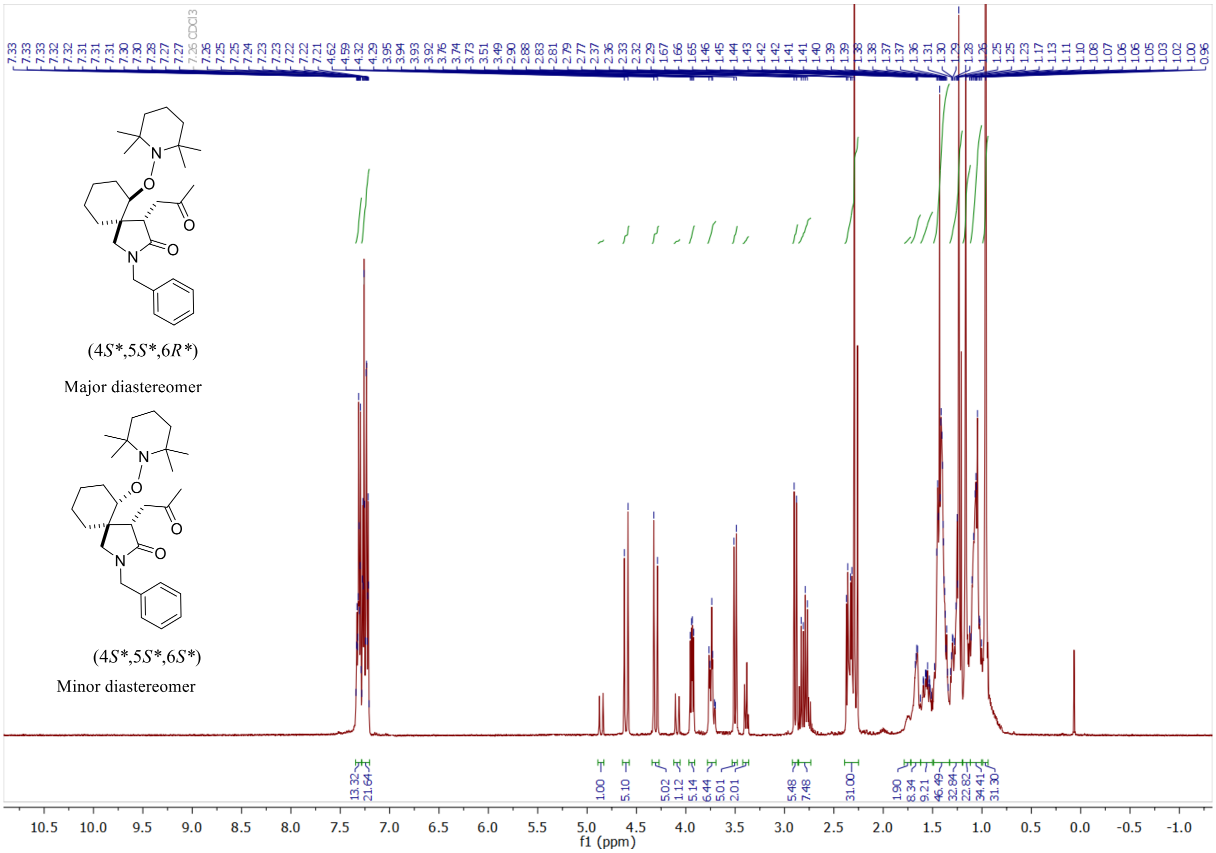
7.33 7.33 7.33 7.32 7.32 7.31 7.31 7.30 7.30 7.28 7.27 7.27 7.26 CDCl3 7.26 7.25 7.25 7.24 7.23 7.23 7.22 7.22 7.21 4.62 4.59 4.32 4.29 3.95 3.94 3.93 3.92 3.76 3.74 3.73 3.51 3.49 2.90 2.88 2.83 2.81 2.79 2.77 2.37 2.36 2.33 2.32 2.29 1.67 1.66 1.65 1.46 1.45 1.44 1.43 1.42 1.42 1.41 1.41 1.40 1.39 1.39 1.38 1.38 1.37 1.37 1.36 1.31 1.30 1.29 1.28 1.26 1.25 1.25 1.23 1.17 1.13 1.11 1.10 1.08 1.07 1.06 1.06 1.05 1.03 1.02 1.00 0.96
(4S*,5S*,6R*)
Major diastereomer
(4S*,5S*,6S*)
Minor diastereomer
13.32 21.64 1.00 5.10 5.02 1.12 5.14 6.44 5.01 2.01 5.48 7.48 31.00 1.90 8.34 9.21 46.49 32.84 22.82 34.41 31.30
10.5 10.0 9.5 9.0 8.5 8.0 7.5 7.0 6.5 6.0 5.5 5.0 4.5 4.0 3.5 3.0 2.5 2.0 1.5 1.0 0.5 0.0 -0.5 -1.0
f1 (ppm)

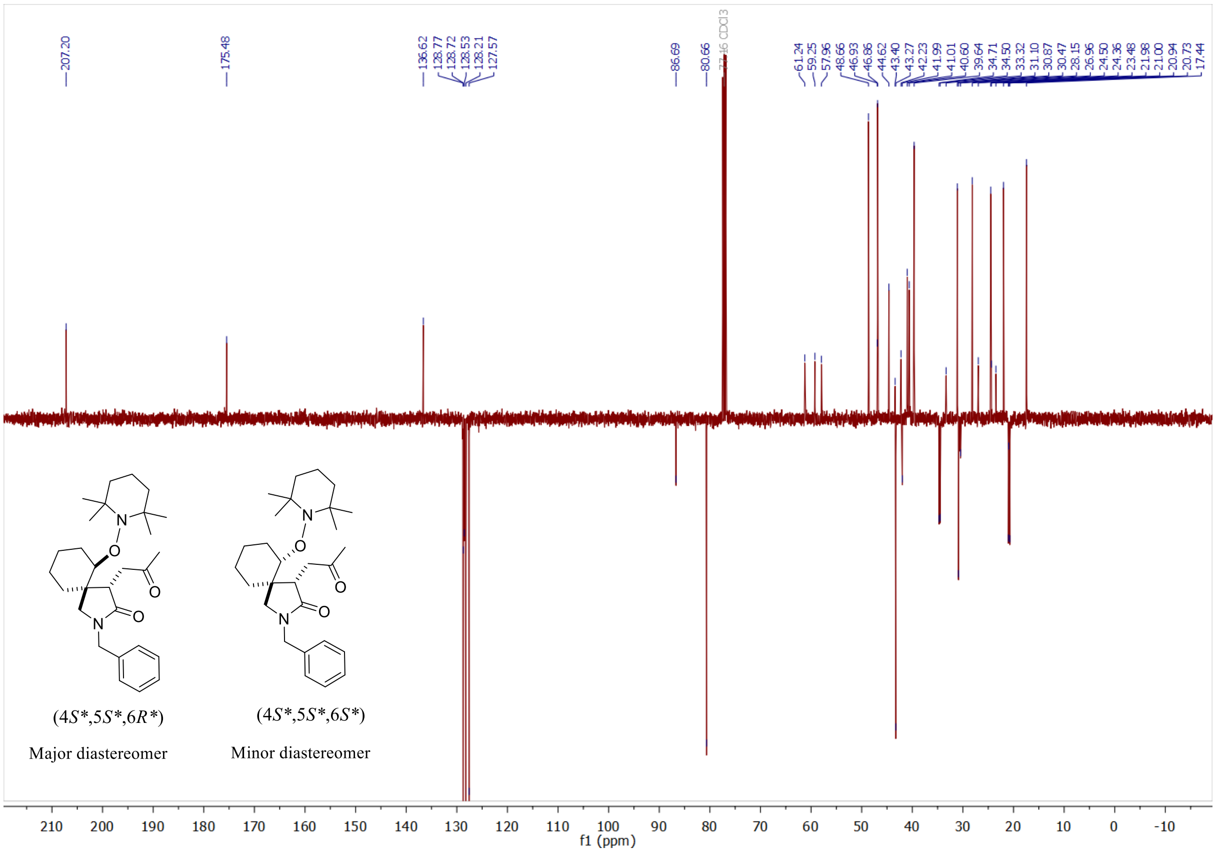
207.20
175.48
136.62 128.77 128.72 128.53 128.21 127.57
86.69
80.66
77.16 CDCl3
61.24 59.25 57.96 48.66 46.93 46.86 44.62 43.40 43.27 42.23 41.99 41.01 40.60 39.64 34.71 34.50 33.32 31.10 30.87 30.47 28.15 26.96 24.50 24.36 23.48 21.98 21.00 20.94 20.73 17.44
(4S*,5S*,6R*)
Major diastereomer
(4S*,5S*,6S*)
Minor diastereomer
210 200 190 180 170 160 150 140 130 120 110 100 90 80 70 60 50 40 30 20 10 0 -10
f1 (ppm)

(3*R,3a*R**,4*R**,6a*R**)- and (3*S**,3a*R**,4*R**,6a*R**)-1-Benzyl-3-(2-oxopropyl)-4-((2,2,6,6-tetramethylpiperidin-1-yl)oxy)hexahydrocyclopenta[*b*]pyrrol-2(1*H*)-one (13o)**

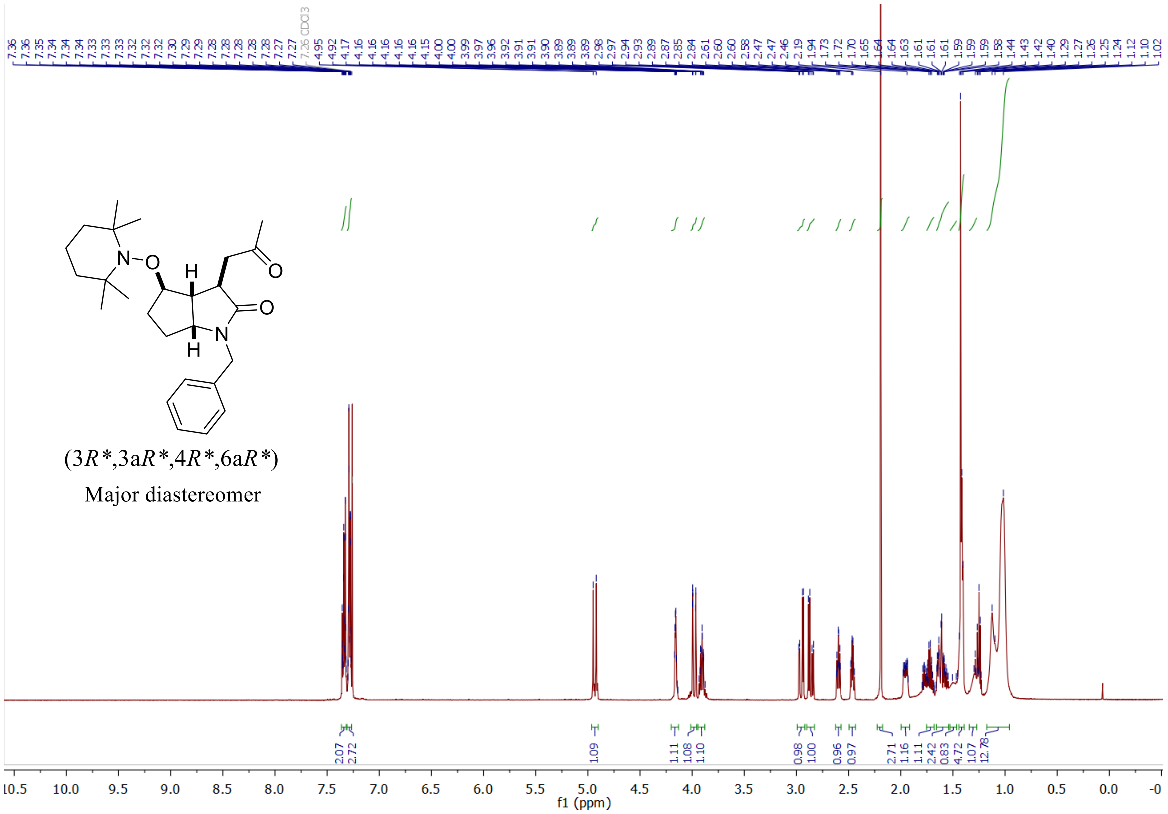

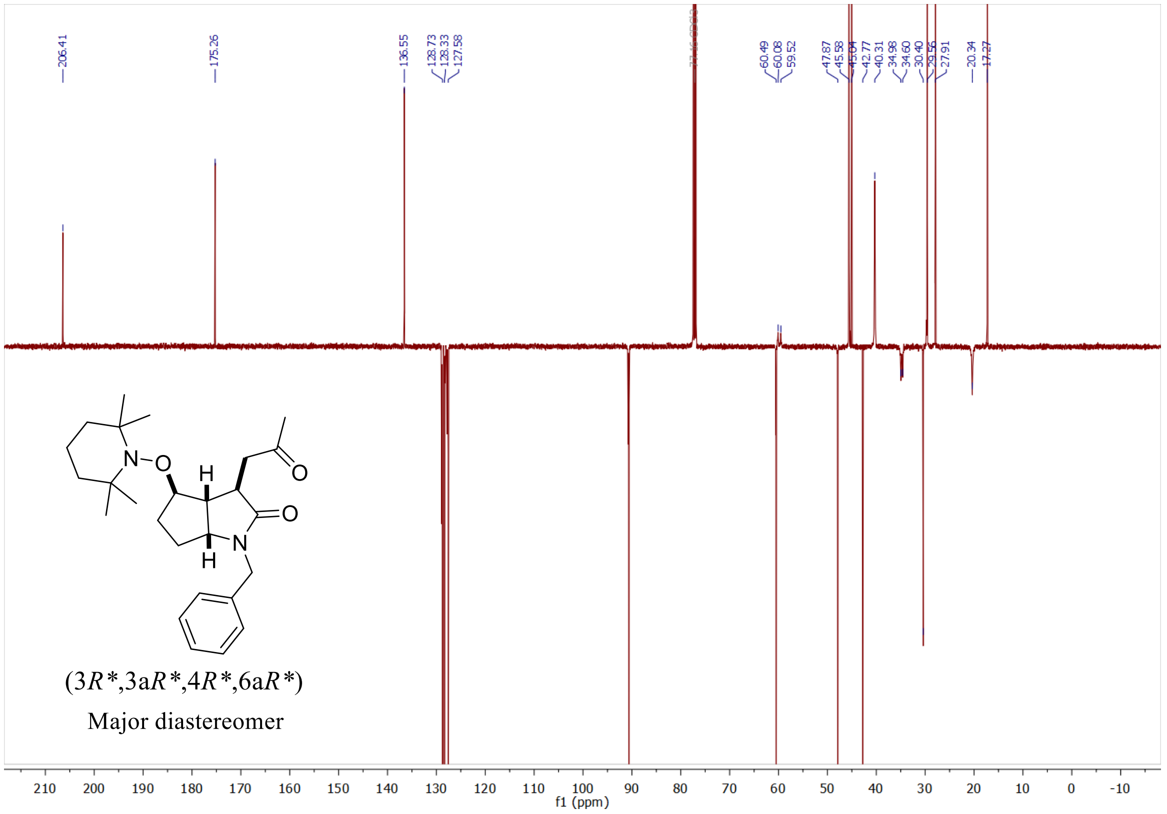

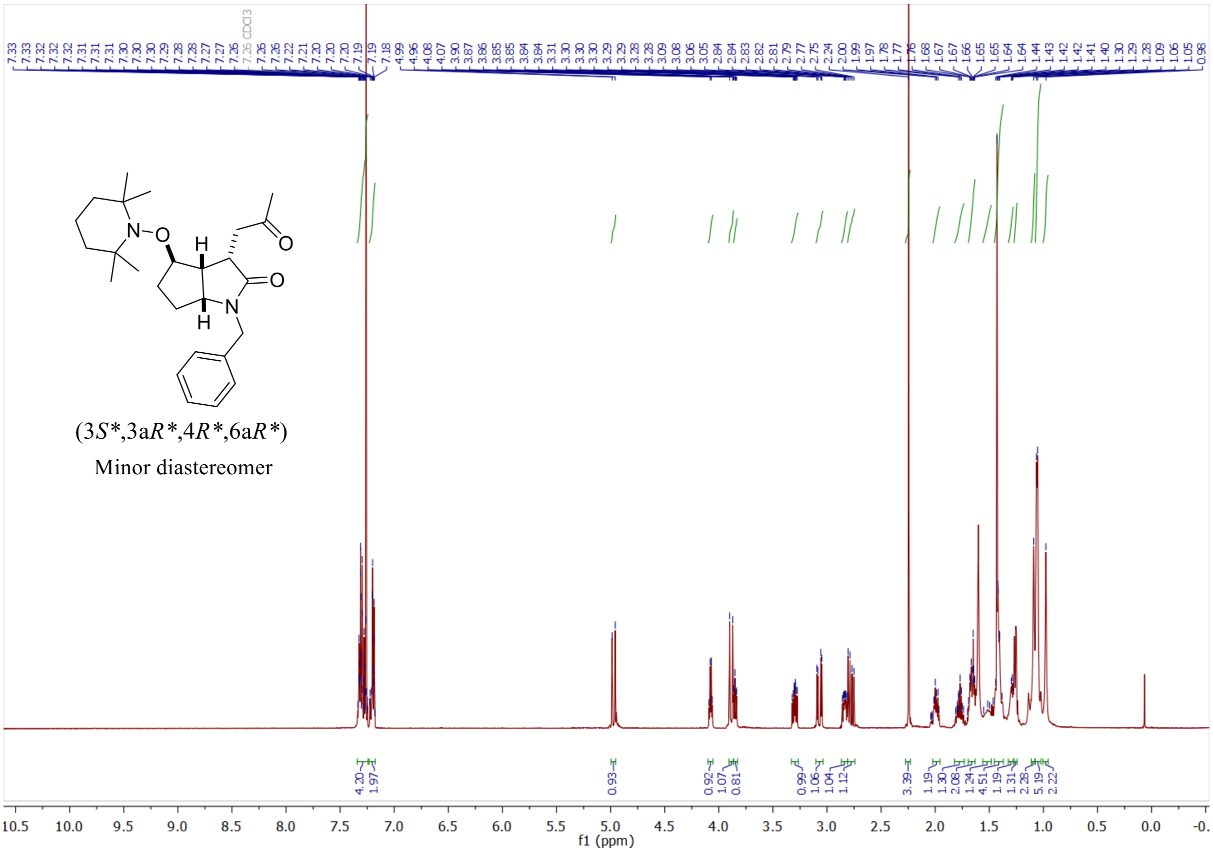
(3S*,3aR*,4R*,6aR*)
Minor diastereomer
f1 (ppm)

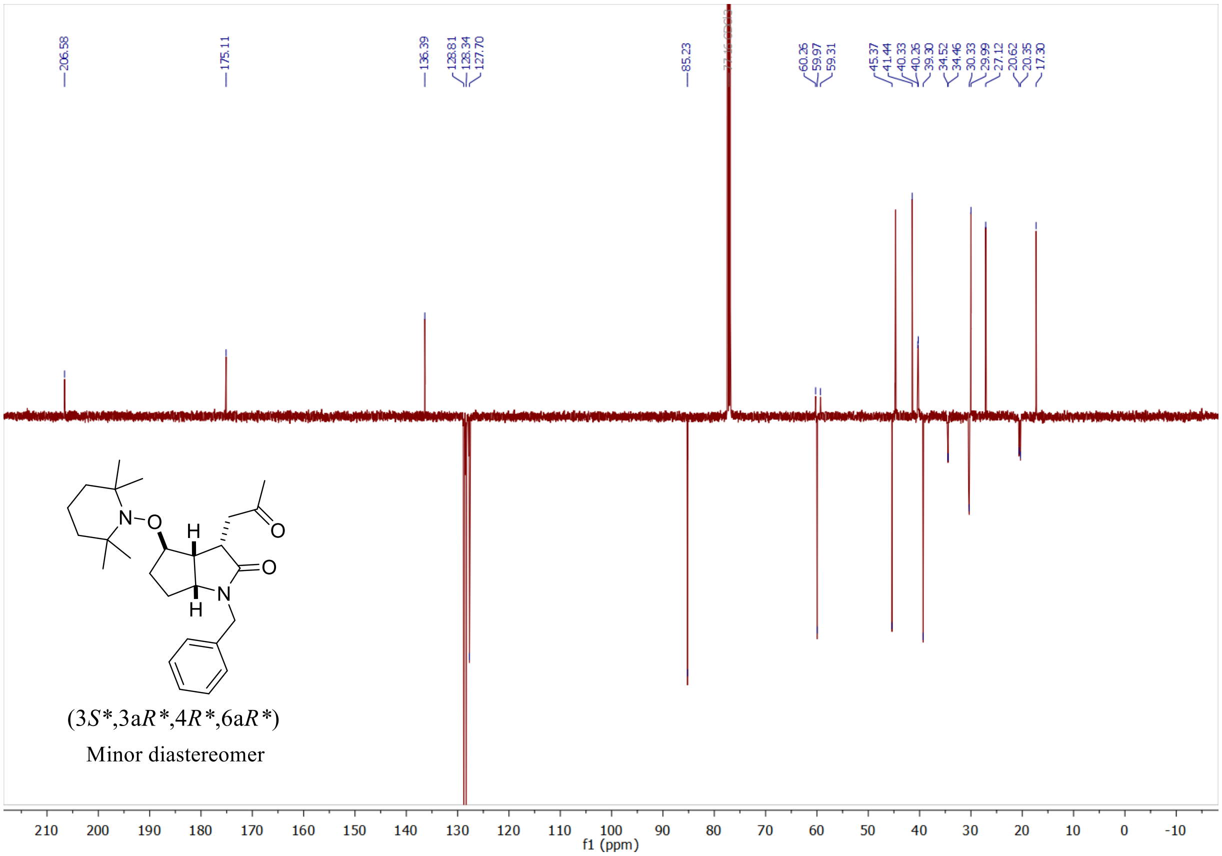
206.58
175.11
136.39
128.81
128.34
127.70
85.23
60.26
59.97
59.31
45.37
41.44
40.33
40.26
39.30
34.52
34.46
30.33
29.99
27.12
20.62
20.35
17.30
(3S*,3aR*,4R*,6aR*)
Minor diastereomer
f1 (ppm)

(3*R,4*R**)-1-Allyl-4-(hydroxymethyl)-3-(2-hydroxypropyl)pyrrolidin-2-one (*trans*-14)**

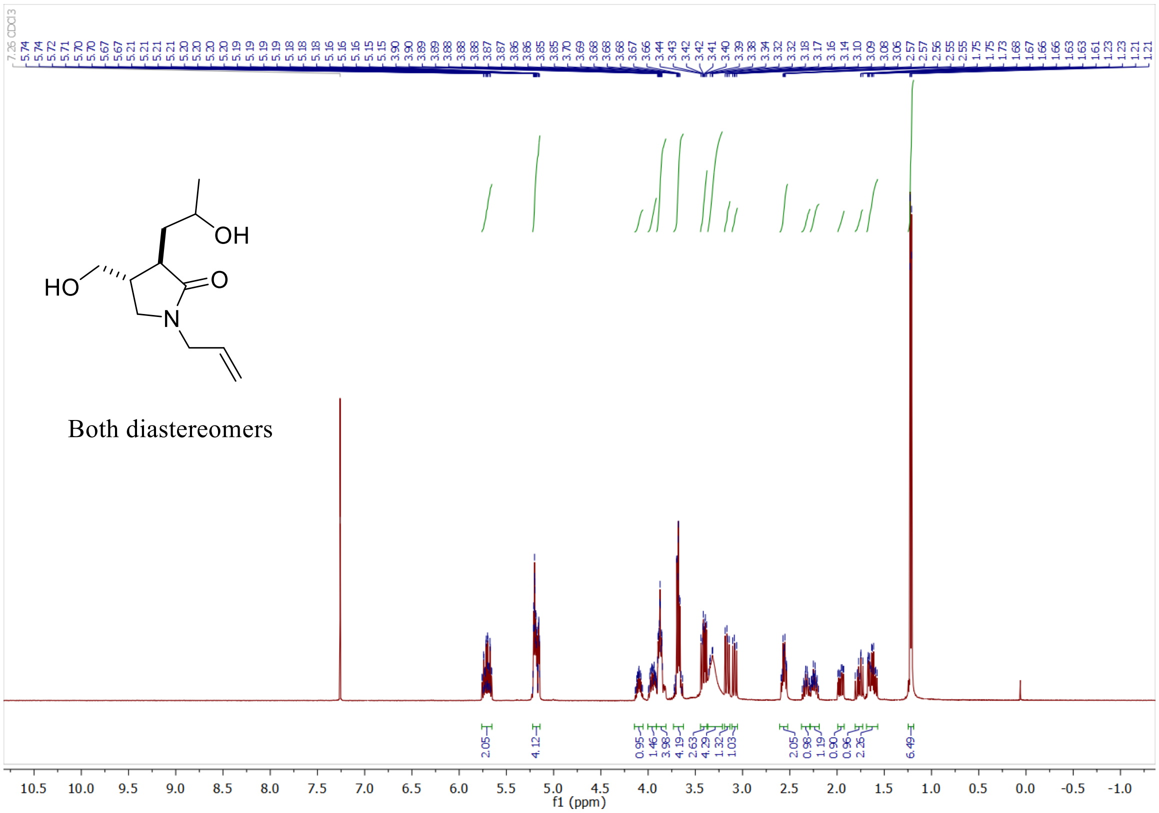

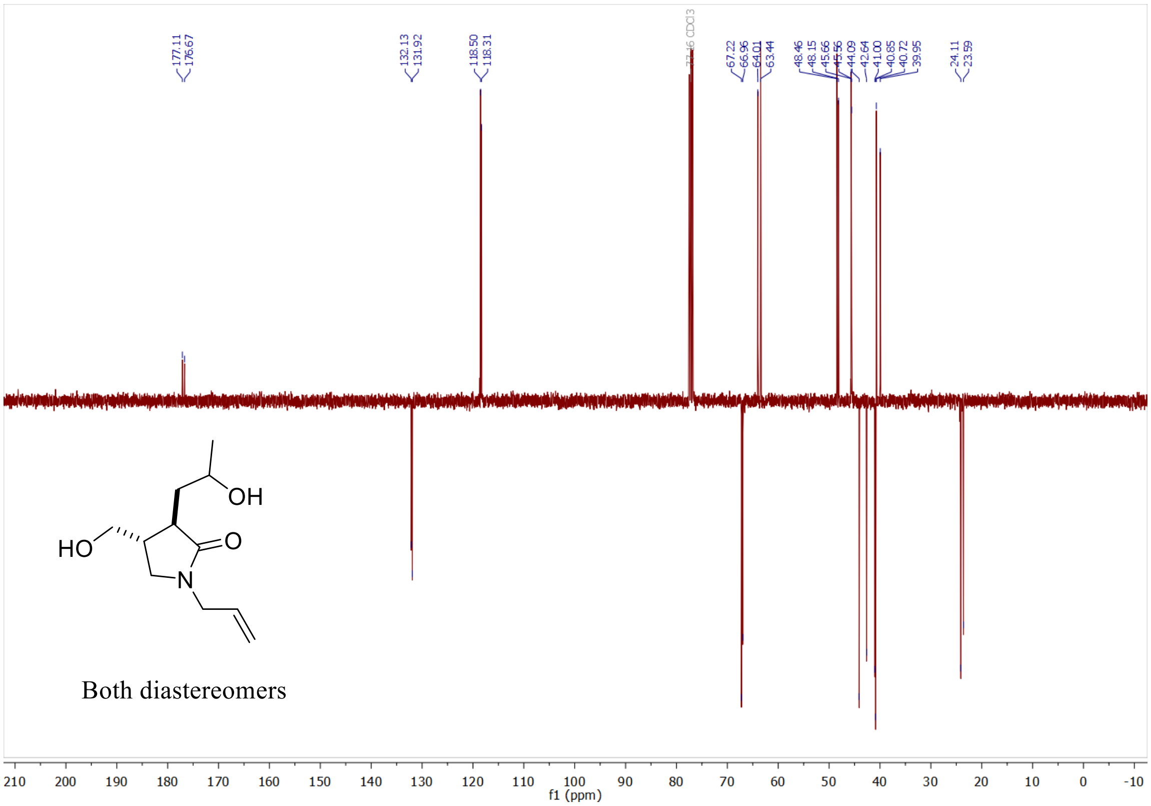

((3*R,4*R**)-1-Allyl-3-(((2,2,6,6-tetramethylpiperidin-1-yl)oxy)methyl)-4-(2-hydroxypropyl)pyrrolidine (*trans*-15)**

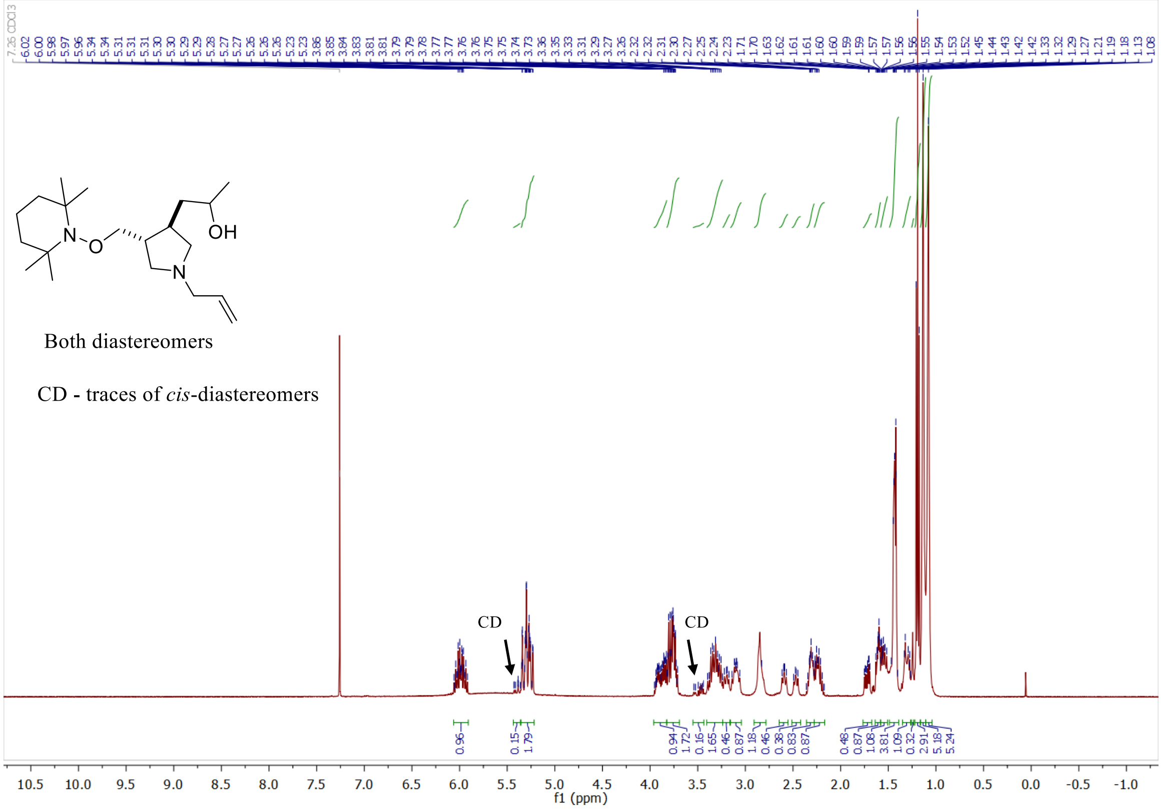

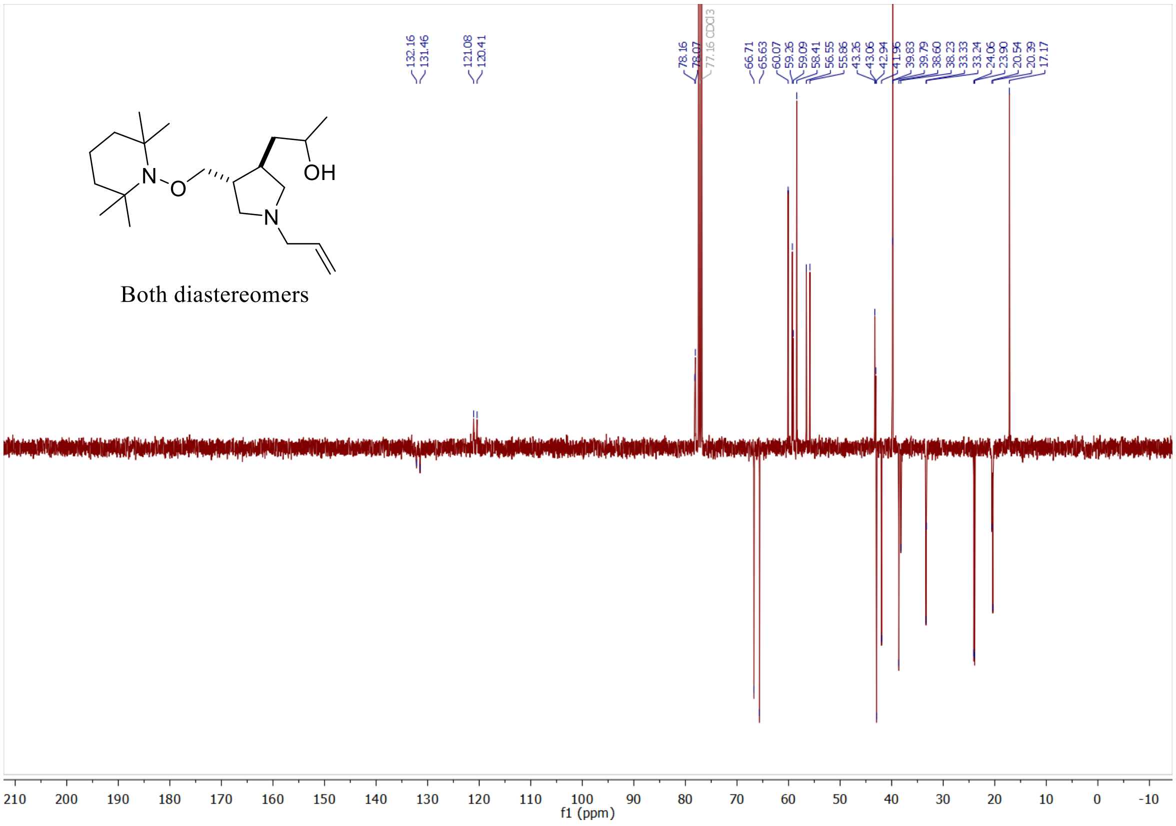

(3a*R,4*R**,6a*R**)-6-Allyl-2-methyl-4-(((2,2,6,6-tetramethylpiperidin-1-yl)oxy)methyl)hexahydro-2*H*-furo[2,3-*b*]pyrrole (16)**

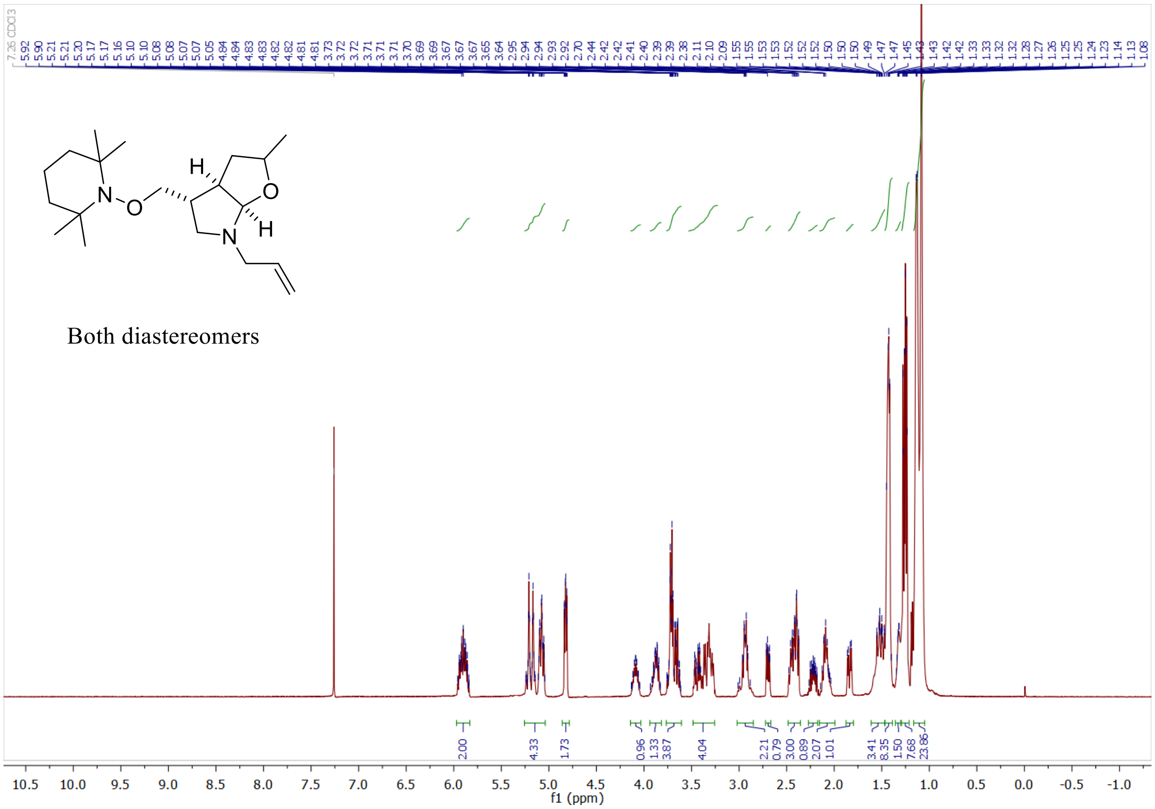

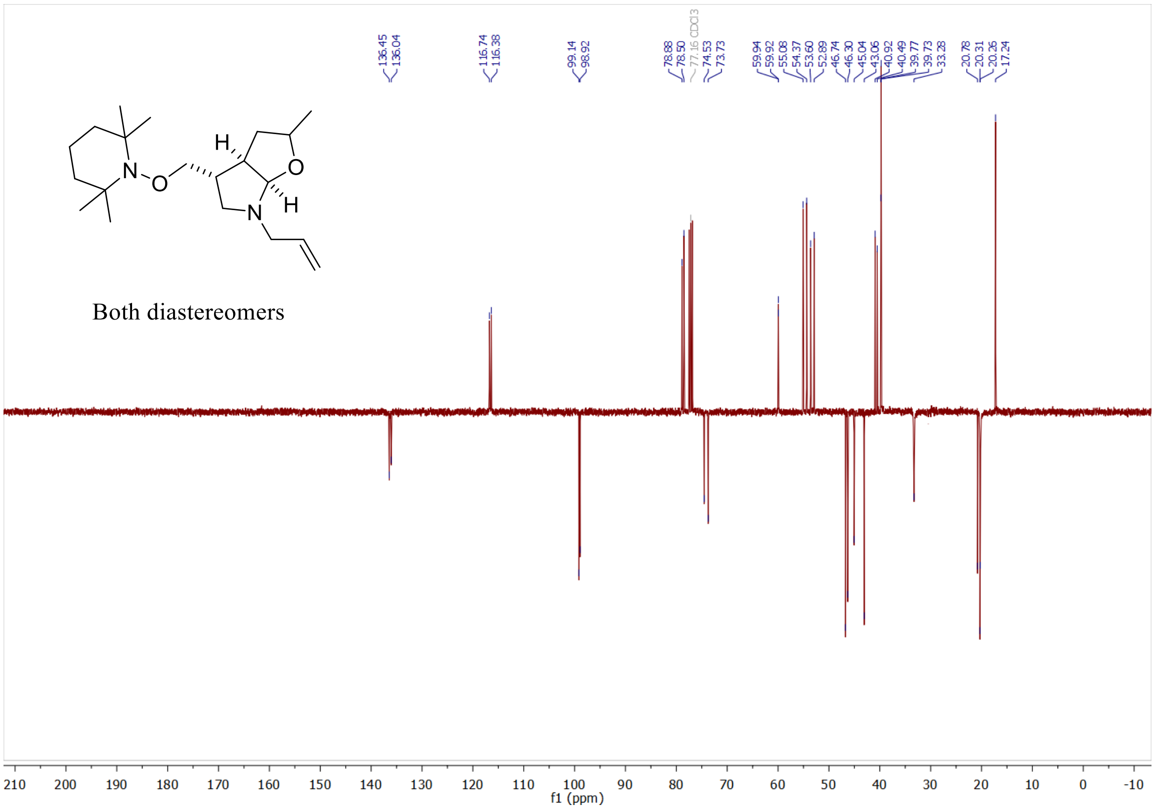

(3*R,4*R**)-3-(2-Hydroxypropyl)-1-((*E*)-prop-1-en-1-yl)-4-(((2,2,6,6-tetramethylpiperidin-1-yl)oxy)methyl)pyrrolidin-2-one (S15)**

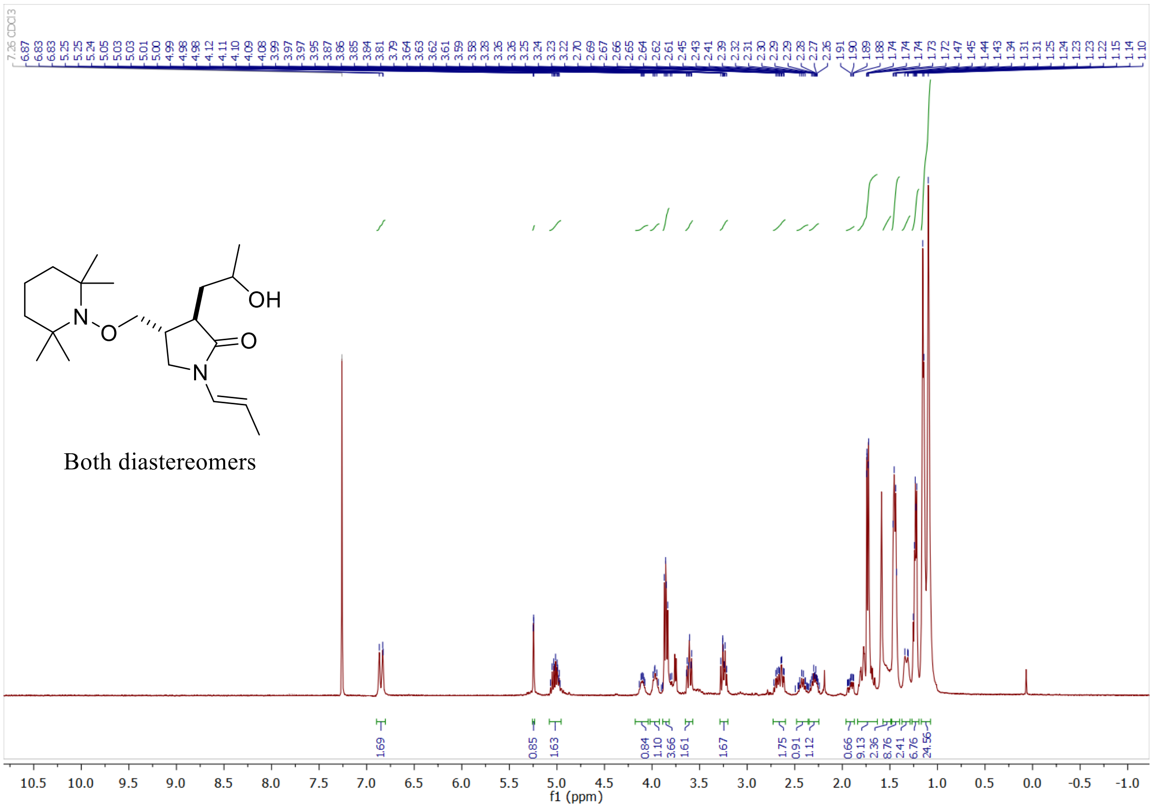

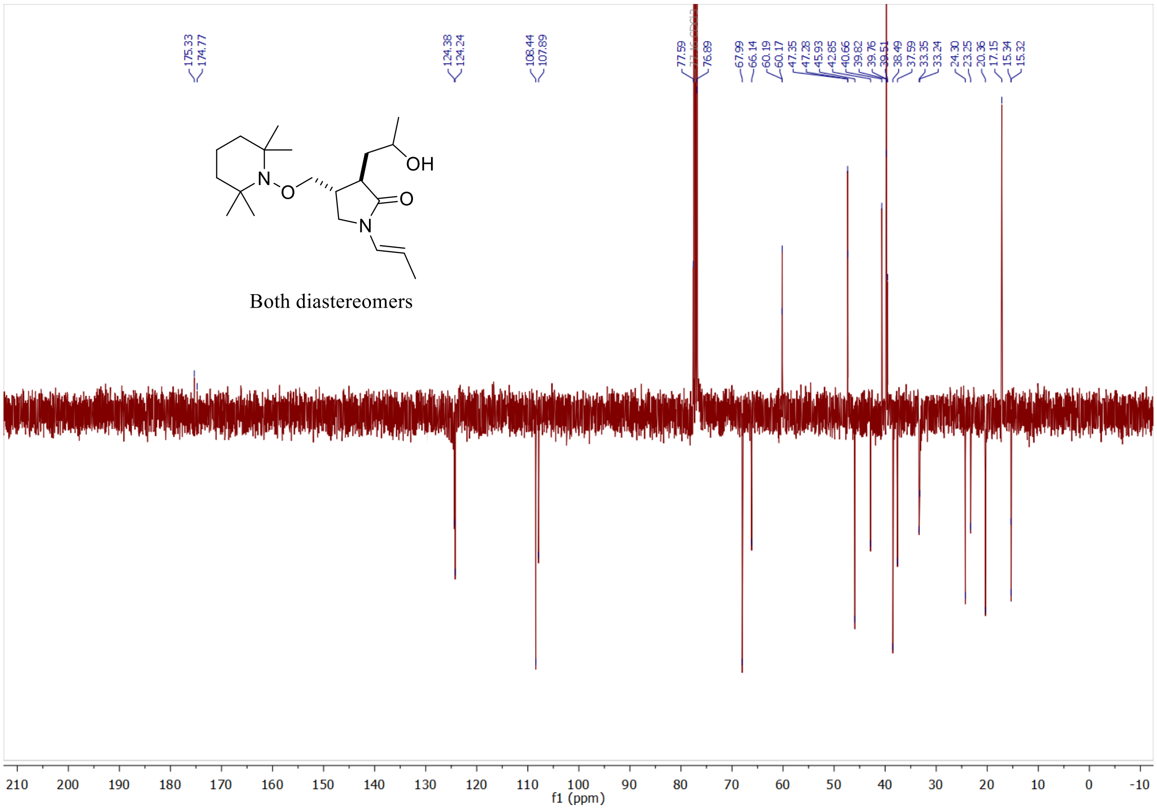

(3*R,4*R**)-3-(2-Hydroxypropyl)-4-(((2,2,6,6-tetramethylpiperidin-1-yl)oxy)methyl)pyrrolidin-2-one (*trans*-17)**

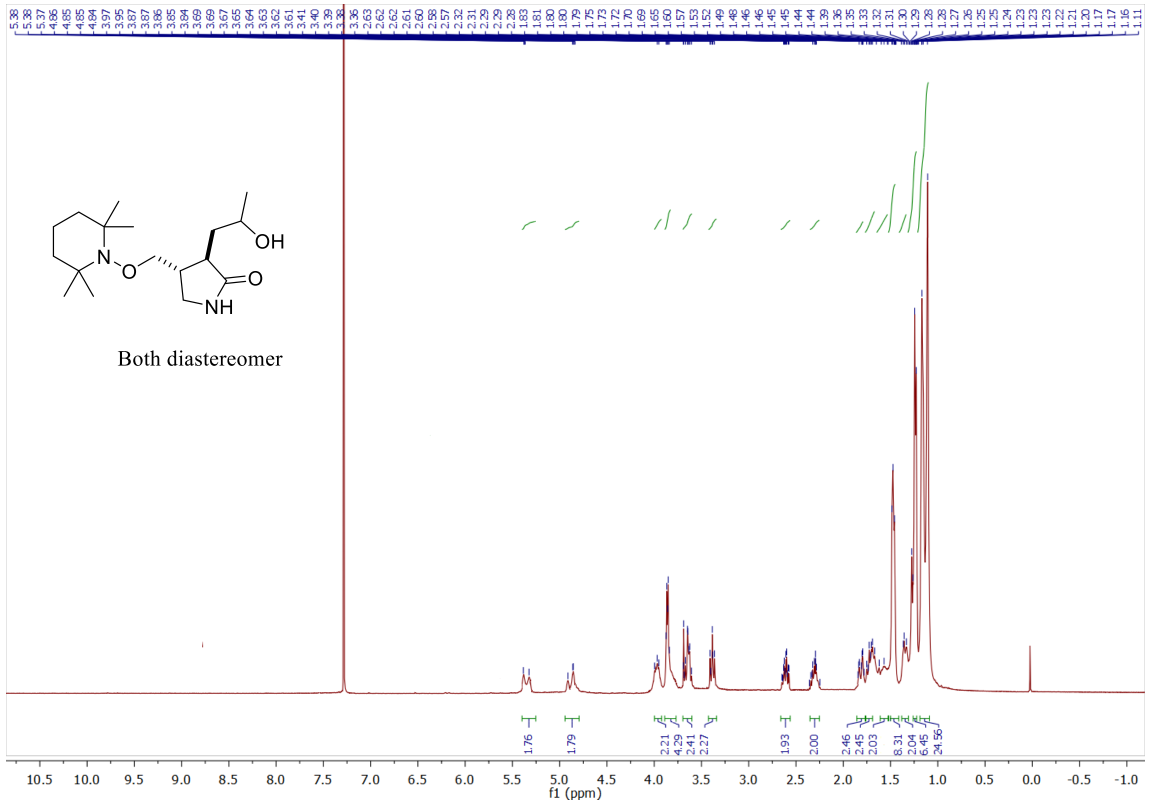

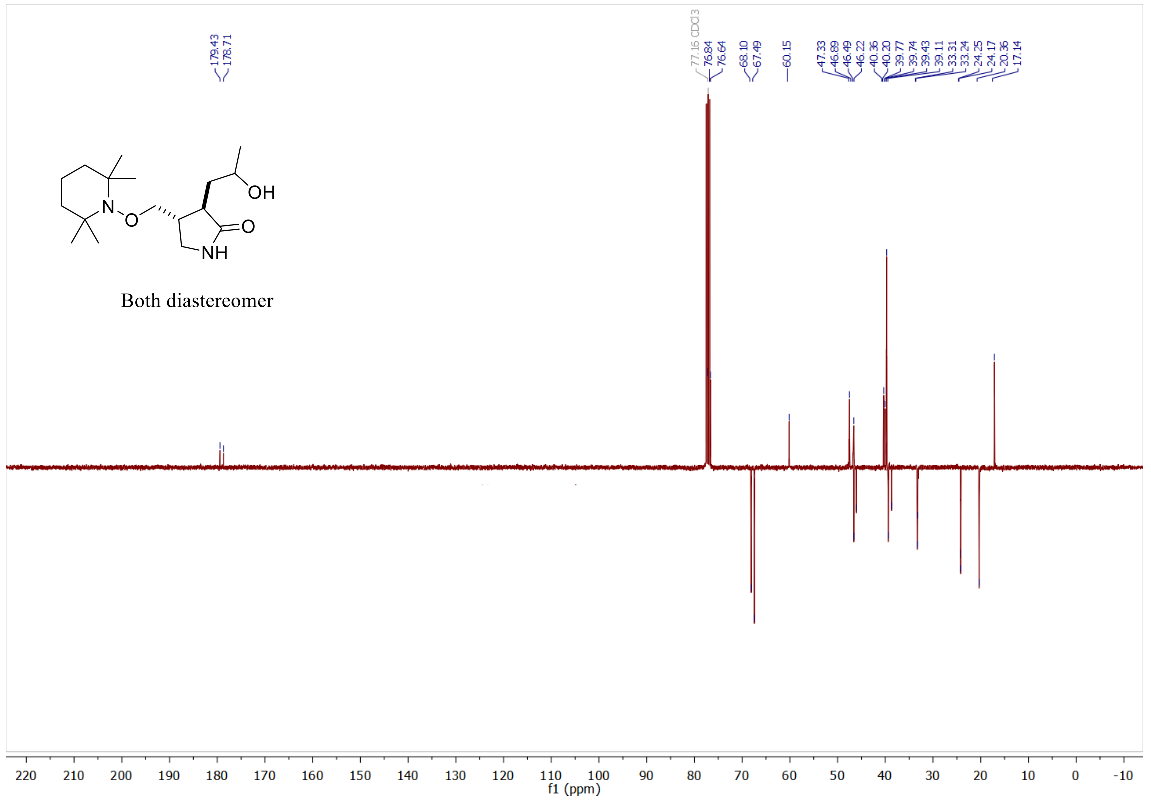

(3*R,3a*R**,6a*R**)- and (3*S**,3a*R**,6a*R**)-1-Benzyl-3-(2-hydroxypropyl)hexahydro-cyclopenta[*b*]pyrrole-2,4-dione (18)**

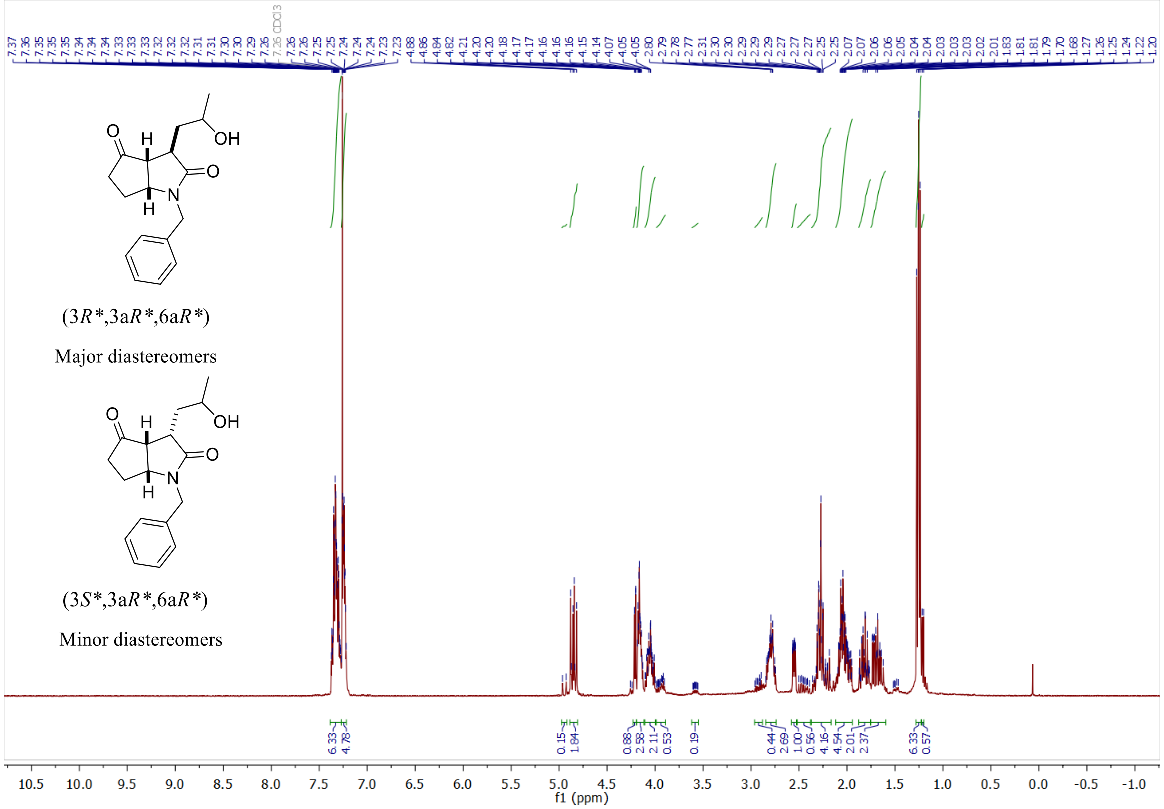

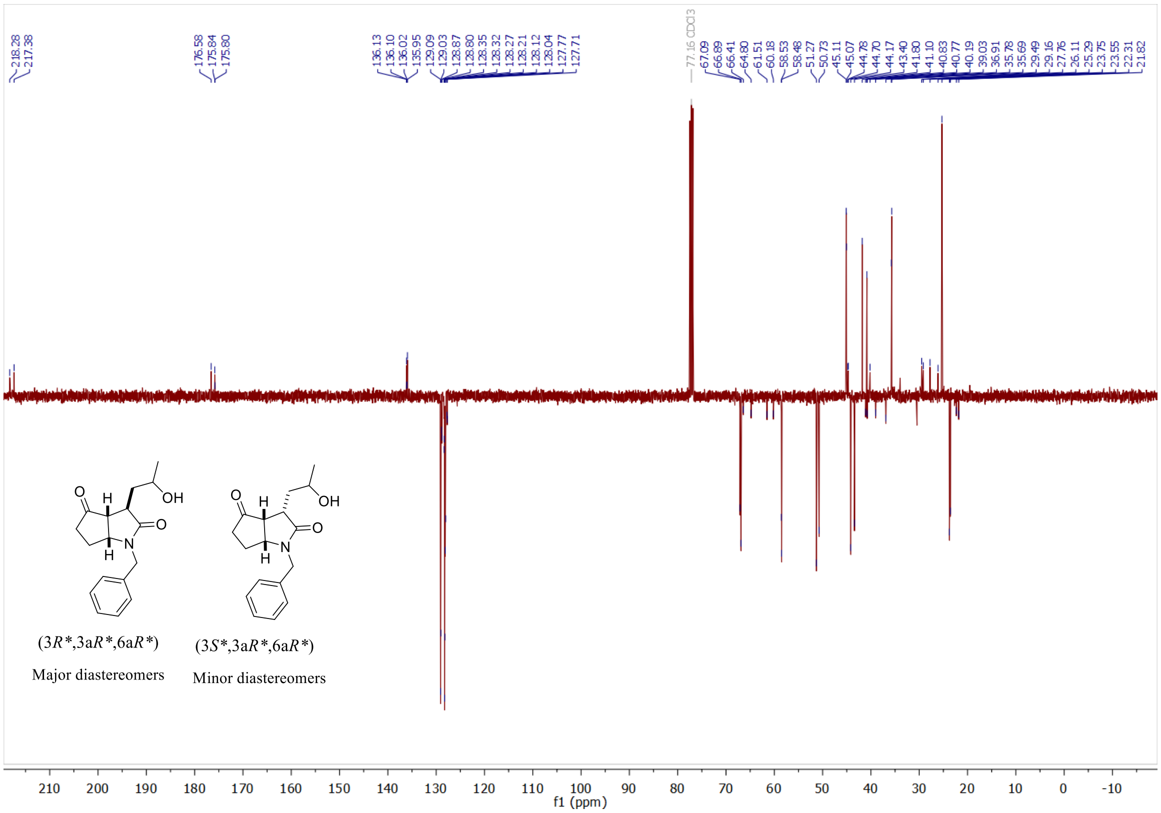

1-(((2*R*,4*S*)- and (2*R*,4*S*)-1-(Allyl((*S*)-1-(naphthalen-2-yl)ethyl)amino)-4-hydroxy-1-oxo-pentan-2-yl)oxy)-2,2,6,6-tetramethylpiperidin-1-ium chloride (S13·HCl):

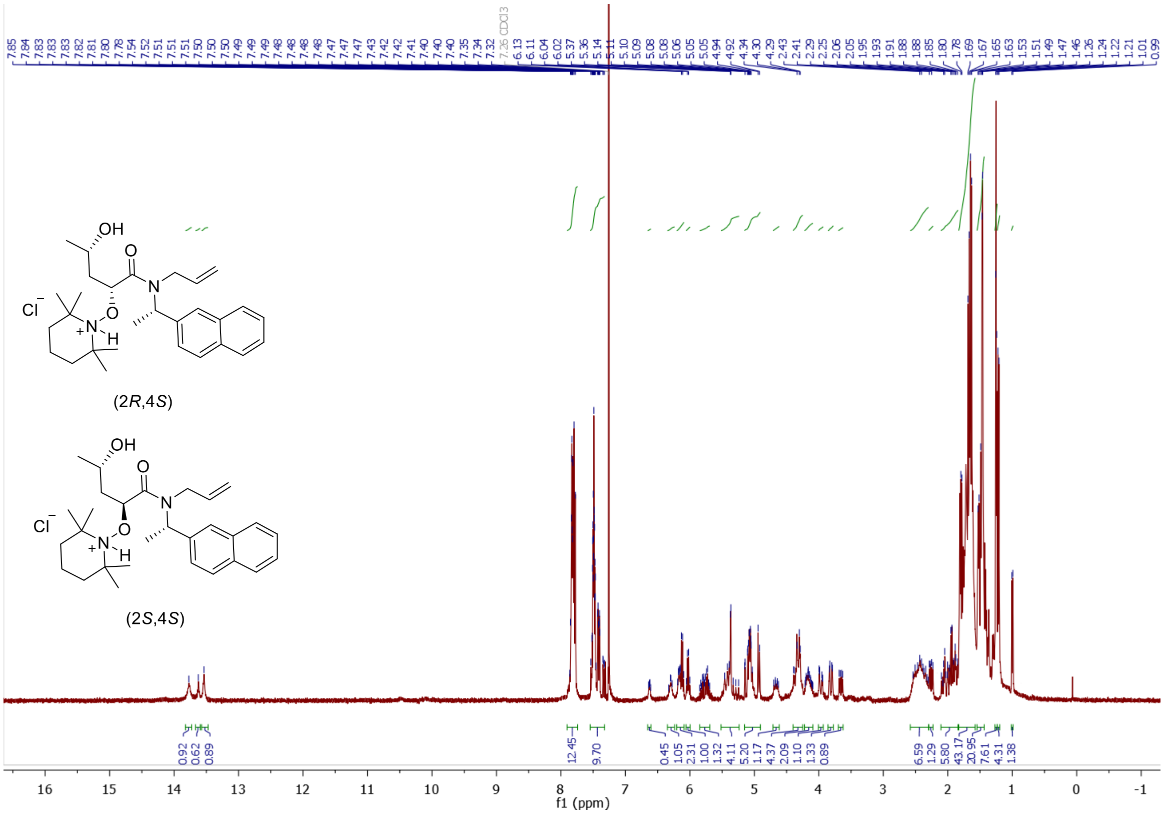

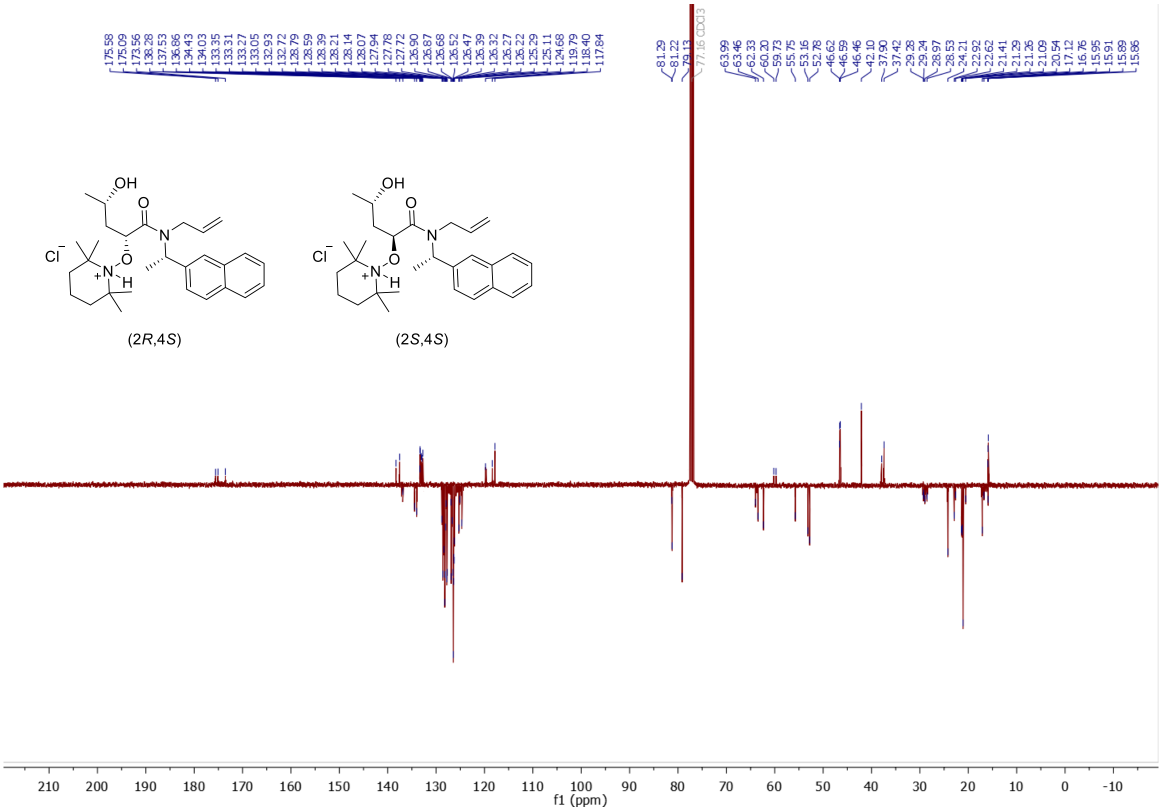

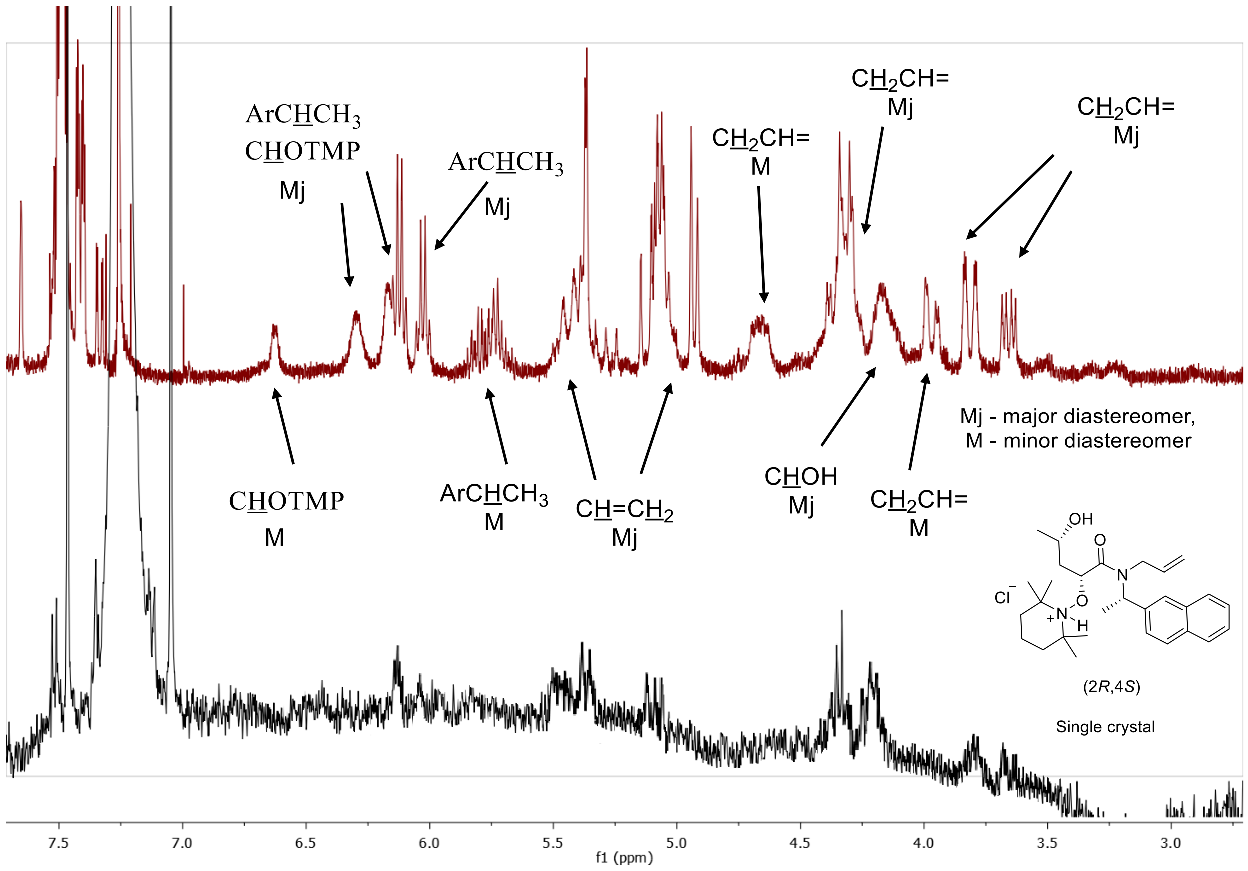
ArCHCH3
CHOTMP
Mj
ArCHCH3
Mj
CH2CH=
M
CH2CH=
Mj
CH2CH=
Mj
CHOTMP
M
ArCHCH3
M
CH=CH2
Mj
CHOH
Mj
CH2CH=
M
Mj - major diastereomer,
M - minor diastereomer
OH
O
N
Cl
+N
H
O
(2R,4S)
Single crystal
7.5
7.0
6.5
6.0
5.5
5.0
4.5
4.0
3.5
3.0
f1 (ppm)
